# The large repertoire of conifer NLR resistance genes includes drought responsive and highly diversified RNLs

**DOI:** 10.1038/s41598-019-47950-7

**Published:** 2019-08-12

**Authors:** Cyril Van Ghelder, Geneviève J. Parent, Philippe Rigault, Julien Prunier, Isabelle Giguère, Sébastien Caron, Juliana Stival Sena, Annie Deslauriers, Jean Bousquet, Daniel Esmenjaud, John MacKay

**Affiliations:** 1 INRA, Université Côte d’Azur, CNRS, ISA, 400 route des Chappes, BP167, 06903 Sophia Antipolis, France; 2 Department of Plant Sciences, University of Oxford, South Parks Road, Oxford, OX1 3RB UK; 3 Forest Research Centre and Institute for Systems and Integrative Biology, Université Laval, 1030 rue de la Médecine, Québec, QC G1V 0A6 Canada; 4 Gydle Inc., 1135 Grande Allée Ouest Suite 220, Québec, QC G1S 1E7 Canada; 5 Center for Organismal Studies (COS), University of Heidelberg, Im Neuenheimer Feld 345, 69120 Heidelberg, Germany; 6 Natural Resources Canada, Canadian Forest Service, Laurentian Forestry Centre, Québec, G1V 4C7 Canada; 7 Département des Sciences Fondamentales, Université du Québec à Chicoutimi, 555 Boulevard de l’Université, Chicoutimi, QC G7H2B1 Canada; 8 Canada Research Chair in Forest Genomics, Université Laval, 1030 rue de la Médecine, Québec, QC G1V 0A6 Canada

**Keywords:** Biotic, Drought

## Abstract

The NLRs or NBS-LRRs (nucleotide-binding, leucine-rich-repeat) form the largest resistance gene family in plants, with lineage-specific contingents of TNL, CNL and RNL subfamilies and a central role in resilience to stress. The origin, evolution and distribution of NLR sequences has been unclear owing in part to the variable size and diversity of the RNL subfamily and a lack of data in Gymnosperms. We developed, searched and annotated transcriptomes assemblies of seven conifers and identified a resource of 3816 expressed NLR sequences. Our analyses encompassed sequences data spanning the major groups of land plants and determinations of NLR transcripts levels in response to drought in white spruce. We showed that conifers have among the most diverse and numerous RNLs in tested land plants. We report an evolutionary swap in the formation of RNLs, which emerged from the fusion of an RPW8 domain to a NB-ARC domain of CNL. We uncovered a quantitative relationship between RNLs and TNLs across all land plants investigated, with an average ratio of 1:10. The conifer RNL repertoire harbours four distinct groups, with two that differ from Angiosperms, one of which contained several upregulated sequences in response to drought while the majority of responsive NLRs are downregulated.

## Introduction

The NLR (nucleotide-binding, leucine-rich repeat) gene family is the main group of cytoplasmic receptors involved in the recognition of specific pathogens as part of effector-triggered immunity in plants^1^. NLRs may confer resistance to biotic aggressors including viruses, bacteria, fungi, nematodes and insects^2–6^, and abiotic stresses such as drought in some cases^7^, and present a wide range of variation across flowering plants^8^. This fundamental role of NLR proteins in resilience to biotic and abiotic stresses has encouraged genome-scale studies of their evolution, primarily in Angiosperms^9–14^.

The NLR protein family may have originated in green algae^15^ and was well-defined early in the land plant lineage^16^. It has evolved through gene duplication, domain fusions, and acquisition of amino-acid sequences that enable the detection of pathogens, protein dimerization, the activation of immune responses and possibly the triggering of a type of programmed cell death known as hypersensitive-like response^17^. The central nucleotide binding NB-ARC domain binds ADP or ATP molecules, which induces a protein conformation change and the switch from an ‘OFF’ to an ‘ON’ state. The NB-ARC domain, which is often used to perform phylogenetic studies, contains highly conserved motifs (Ploop, kinase, RNBS, GLPL, MHD) involved in intra- and extra-molecular interactions^18,19^. Among these motifs, the RNBS-A, RNBS-D and the MHD motifs show some variations that are correlated to the N-terminal domain identity and can be used to classify the sequences into subfamilies. The C-terminal LRR domain mainly supervises the recognition function through interactions with effectors or molecules modified by effectors through residues in its horseshoe 3D-structure^20^.

The conserved Toll interleukin-1 receptor (TIR) domain at the N-terminus defines the large TIR-NB-ARC-LRR (TNLs) subfamily^21^, many members of which display a C-terminal post–LRR domain (PL) of unknown function^22^. The two other subfamilies are made up of non-TIR containing NLRs with a diversity of N-terminal sequences and structures: (1) the CC-NB-ARC-LRR (CNL) protein subfamily, which displays a coiled-coil structure in their N-terminus^9^ and (2) the RPW8-NB-ARC-LRR (RNL), which shares homology with RPW8-only proteins (RESISTANCE TO POWDERY MILDEW8) from *Arabidopsis thaliana*^23^. In addition, partial NLRs such as RBA1 (TIR-only protein), TN2 (TIR-NB-ARC-only), RPW8.1 and RPW8.2 (RPW8-only) were recently shown to confer resistance to pathogens^23–25^; therefore, we considered truncated versions in our study of the NLR gene family.

The occurrence of the different NLR subfamilies is variable among the major plant taxonomic groups. For instance, Monocots have many CNL encoding genes and lack the TNL subfamily^21^. The RNL subfamily is found in primitive plants such as *Physcomitrella patens* but has few or no members in other plant lineages, except in the Rosaceae, where it underwent a major expansion^26^. The RNL subfamily have been classified as a sister clade to the CNLs and TNLs^11^, but its evolution remains poorly resolved owing to the variability in both CNL and RNL subfamilies and its sequence motifs remain poorly defined. In Angiosperms, RNLs are subdivided into two identified subclades^27^ based on the homology of their RPW8 domain to the one of (1) the N-required gene 1 (NRG1), co-responsible of the Tobacco Mosaic Virus resistance in *Nicotiana benthamiana*^28^, or (2) the activated disease resistance gene 1 (ADR1), involved in both pathogen resistance and drought tolerance in *Arabidopsis thaliana*^7^. Functionally, RNL genes function as helper proteins acting downstream of sensor NLRs^27,29–31^.

The recent analysis of *Pinus taeda* L. and *Picea abies* (L.) H. Karst. genome drafts recently suggested the occurrence of numerous RNL sequences^26^ but a comprehensive analysis of conifer NLRs is lacking. A few conifer NLR sequences have been described^32^ and linked to resistance against pathogens such as *Cronartium ribicola*, which is responsible for the white pine blister rust^33,34^. Conifers possess very large genomes sizes and abundant pseudogenes, which hinder genome assembly and analysis of large gene families, but transcriptome sequencing provides an effective alternative to rapidly acquire large datasets of expressed genes^35–37^.

The overall goal of this study was to investigate the makeup of NLRs in conifers with the aim of improving our understanding of the RNL subfamily in plants. We introduced 3816 expressed NLR sequences in seven conifers in the *Pinaceae* and the *Cupressaceae* families. Our specific objectives were to: (1) identify the repertoire of expressed NLR sequences through the analysis of seven conifer transcriptomes; (2) classify the poorly known conifer NLRs based on amino acid domains and motif analyses; (3) investigate the diversity and the drought responsiveness of conifer RNLs, testing whether or not conifers form a single group external to the Angiosperm groups as recently proposed^27^. We found that conifer RNLs presented a level of sequence diversity that is unparalleled in tested Angiosperms and possibly in other plant taxa. Our results also suggest that they play a key role in drought response.

## Results

### NLRs are abundant and diverse in the *Pinales*

We developed a dataset of 3816 expressed NLR sequences (Supplementary Data S1) by analysing the transcriptomes of seven conifer species in five genera of the *Pinaceae* and *Cupressaceae*. The sequences were identified using the Pfam database (31.0) in the predicted amino acid sequence datasets obtained from the assembled mRNA sequence. The expressed sequences contained at least one of the canonical NB-ARC, RPW8, or TIR domains while CC-only and LRR-only were excluded as they may be mispredicted or not specific to NLRs. We filtered the full dataset to eliminate potentially redundant NLR peptides of a same encoding-gene. The number of expressed NLR genes thus identified ranged from 338 in *Abies balsamea* to 725 in *Picea mariana* (Table 1), in line with the total of 679 NLR genes identified in the genome of *P*. *taeda*^15^. The total number of distinct NLR transcripts represented 0.73% to 1.35% of the assembled transcriptome depending on the species, which are substantial proportions of such genes when comparing with other Angiosperm transcriptomes (Supplementary Table S1).

**Table 1:** Distribution of NLRs and their subfamily assignation in seven conifer transcriptomes.

		*Abies balsamea*	*Larix laricina*	*Picea glauca*	*Picea mariana*	*Pinus banksiana*	*Pinus strobus*	*Thuja occidentalis*	Dist^†^ (%)
	Number of transcripts	46178	50712	37491	58751	47473	45447	38767	
	TOTAL NLR	338	633	506	725	486	560	486	
	Ratios	0.73%	1.25%	1.35%	1.23%	1.02%	1.23%	1.25%	
Non-TIR-NLR	CNL								
	NB_CNL_–(LRR)	65	146	115	166	82	103	51	22%
	Ratios	0.14%	0.29%	0.31%	0.28%	0.17%	0.23%	0.13%	
	CNL2								
	NB_CNL2_–(LRR)	47	18	17	17	36	21	34	6%
	Ratios	0.10%	0.04%	0.05%	0.03%	0.08%	0.05%	0.09%	
	RNL								
	RPW8	5	5	6	13	8	12	3	9%
	NB_RNL_–(LRR)	11	8	6	10	21	12	13	
	RPW8–NB_RNL_–(LRR)	16	33	19	16	26	16	15	
	*Total RNL-related*	32	46	31	43	55	40	31	
	Ratios	0.07%	0.09%	0.08%	0.07%	0.12%	0.09%	0.08%	
TIR-NLR	TNL								
	TIR	43	100	121	144	94	89	72	63%
	NB_TNL_–(LRR)	52	97	99	128	104	122	91	
	TIR–NB_TNL_–(LRR)	55	143	58	105	52	126	154	
	TIR–LRR	0	3	1	2	0	2	0	
	*Total TNL-related*	150	343	279	379	250	339	317	
	Ratios	0.32%	0.68%	0.74%	0.65%	0.53%	0.75%	0.82%	
	Atypical NLR	15	26	8	14	7	14	14	
	Undetermined NLR	29	54	56	106	56	43	39	

^†^The distribution is calculated using the total sequence number of each subfamilies in proportion of the total of assigned sequences (CNL, CNL2, RNL and TNL).

### Domain and motif specificities

**Figure 1:**
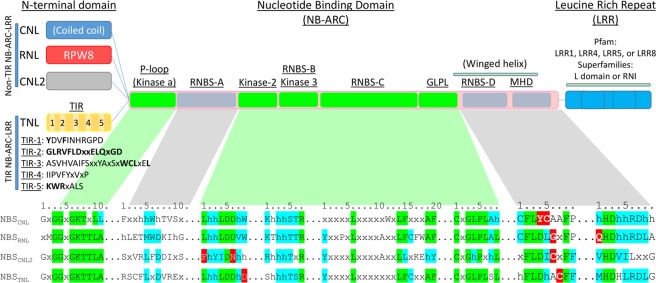
Schematic representation of the four subfamilies of NLRs in conifers. The detailed motifs of the NB-ARC domain are split into conserved motifs through NLR subfamilies (green) and discriminative motifs (grey). Pfam and Superfamilies entry identifiers are indicated above the green bar. Discriminant amino acids are highlighted in red, ‘h’ indicates various hydrophobic residue.

The predicted NLR proteins followed the same domain organisation in conifers as described in Angiosperms, which included the differential N-terminal domains TIR, CC or RPW8, a conserved central NB-ARC domain, and a polymorphic C-terminal domain (LRR) (Supplementary Data S2). The TIR domain of conifer and Angiosperm TNLs was generally conserved, unlike the non-TIR N-terminus of the other NLRs, which was highly polymorphic and mainly made up of conformational domains (coiled-coil). The conifer TIR domain sequences were similar to those observed in Angiosperms and were characterised by a site displaying hydroxyl groups (poly-serine or threonine) close to the initial methionine, followed by the TIR-1 to TIR-5 motifs found in Angiosperms (Fig. 1). The TIR-1, 2 and 5 corresponded to the human TLR boxes 1 to 3, respectively. Within the NB-ARC domain, highly conserved sequence signatures and variations were observed in conifers as found in Dicots. The conserved elements included the P-loop, Kinase 2, Kinase 3, RNBS-C and GLPL motifs, which are similar in all NLR subfamilies, and the RNBS-A, RNBS-D and MHD, which were specific to each of the NLR subfamilies (Fig. 1).

We report two specific signatures in the RNBS-D (CFLDLGxFP) and MHD (QHD) motifs that enabled an accurate detection of RNL sequences in conifer sequences. Among these motifs that have been previously identified in Angiosperms^38^, the RNBS-D motif was particularly useful to discriminate between different NLR sequences. It harboured conserved aspartic acid, glycine and cysteine residues that characterise each of the NLR forms (Fig. 1). We found that the QHD motif exclusively contained a glutamine (Q1) residue before the conserved histidine (H2) and aspartic acid (D3) in the conifer RNL sequences, in contrast to the conventional methionine (M1) or other hydrophobic residues found in CNL and TNL proteins. We also identified an unconventional non-TIR NLR subfamily (CNL2), which had a unique combination of features in the NB-ARC domain, including a conserved cysteine in the sixth position (C6) after the conserved aspartic acid (D4) in the RNBS-D motif, a VHD motif and a polymorphic RNBS-A motif (Table 1, Fig. 1). A blastp analysis based on the RNBS-D motif only identified sporadic NB-ARC_CNL2_ sequences in recent Angiosperms.

We scanned the conifer sequences for conserved C-terminal signatures downstream of the LRR domain but none were clearly identified, in contrast to Dicot TNLs where post-LRR (PL) domains are ubiquitous. None of the known Dicot PL motifs were detected in the entire dataset of predicted peptides by using blastp or FIMO analysis. We also searched in datasets from ancient plants among Embryophytes (*P*. *patens*, *Sphagnum fallax*), *Ginkgo biloba*, another conifer (*P*. *abies*), and an early-diverged Angiosperm (*Amborella trichopoda*), and only found complete PL motifs in *A*. *trichopoda* and a few partial ones in *G*. *biloba*. These observations suggest a partial acquisition early in seed plant evolution, and a potential adaptive expansion in early flowering plants (Supplementary Fig. S1).

### Conifer sequences are broadly represented in all NLR subfamilies

We assigned each of the transcripts to specific subgroups CNL, CNL2, RNL or TNL based on N-terminal sequences and specific motifs in the NB-ARC domain when possible, considering only the sequences containing *bona fide* NB-ARC domains. The TNL sequences were most abundant in all of the species and represented 63% of the total (Table 1), except in *A*. *balsamea*, which harboured as many CNLs as TNLs. The respective proportions of the different subfamilies were similar in the two *Picea* species and *Larix laricina* (*χ*^2^, *P* > *0*.*999*), the proportion of TNL sequences was slightly higher in *Thuja occidentalis* and *Pinus strobus* and lower in *A*. *balsamea*, while RNL sequences were most abundant in *Pinus banksiana* (0.12% of all the transcripts) (Table 1).

### Retracing the origin and distribution of RNL identifies a potential link to TNL evolution

The analysis of NB-ARC motifs is crucial to assign sequences to particular subfamilies. We reliably identified motifs in the NB-ARC_RNL_ domain including specific signatures that were absents from all of the NB-ARC_TNL_ or NB-ARC_CNL_ sequences, owing to the large and relatively uniform numbers of RNL sequences in conifers (Table 1). We used these results on RNL motifs to compare conifers and ancient plants to gain insights into the formation of RNL, and to investigate RNL sequence contingent sizes across land plants, aiming to explain differences observed in the seed plant phylogeny.

In early land plants, we identified two, four and ten putative RNL sequences in *Marchantia polymorpha*, *P*. *patens* and *S*. *fallax*, respectively, by using RPW8 and NB-ARC signatures. Although we had detected RPW8 domains in these sequences, their adjacent NB-ARC domains did not possess the NB-ARC_RNL_ motifs and clearly displayed CNL signatures in the RNBS-A, RNBS-D and MHD motifs (Supplementary Fig. S2). Among all the plant proteomes tested, this peculiarity was only found in the early land plants. These same species also harboured an RPW8-containaning sequence associated with other domains such as Ubox/Arm, PLAC8 and PKinase (Mapoly0115s0019.1.p, Pp3c12_21520V3.1.p, Sphfalx0341s0009.2.p). The data showed that the RPW8 domain fused to a core NB-ARC_CNL_ or connected to other domains already existing in early land plants.

**Figure 2:**
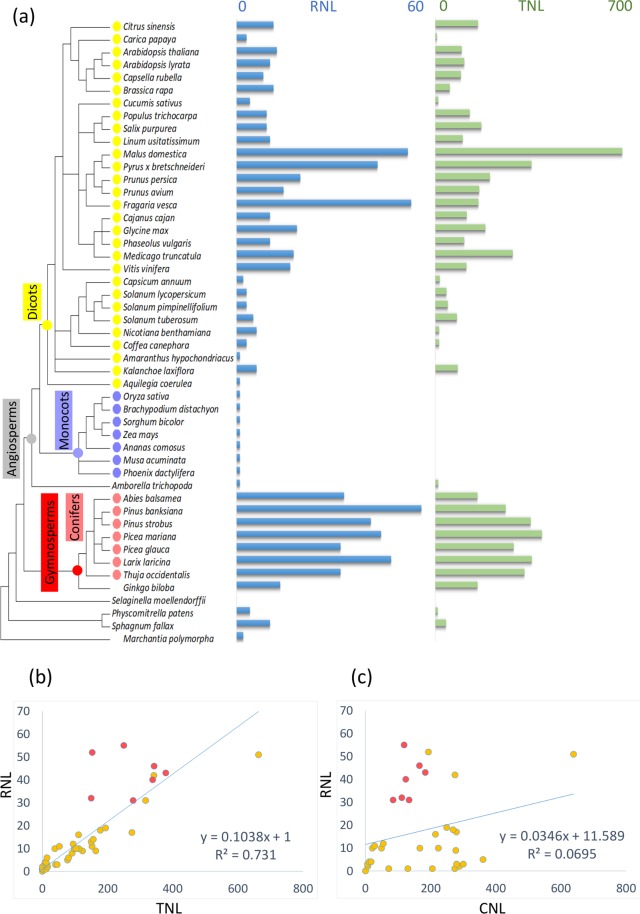
Distribution of the RNL and TNL genes in 49 land plants. (**a**) RNL- and TNL-related sequence numbers are mapped to the species tree created from the NCBI Common Taxonomy Tree (https://www.ncbi.nlm.nih.gov/Taxonomy/CommonTree/wwwcmt.cgi). Abscissa axis represents the protein numbers situated above the diagram. (**b**) RNL versus TNL numbers. (**c**) RNL versus CNL numbers. Light red dots represent the conifer species used in this study. The linear regression equation and the R^2^ coefficient are indicated.

The abundance of RNLs was then investigated in 42 plant proteomes distributed among diverse taxonomic groups of land plants in addition to those of the seven conifers (Fig. 2 and Supplementary Table S2). Zhong and Cheng (2016) identified the expansive RNL subfamily in several of the *Rosaceae* using blastp and also suggested that the *Pinus* and *Picea* had numerous RNLs^26^. Using the same blastp procedure, we also found large RNL contingents in both *Pinus* and *Picea* as well as in the two other groups of the Pinaceae family, namely Abietoideae and Laricoideae, and in the Cupressaceae family (Fig. 2a). We also detected thirteen RNLs in the early-diverged Gymnosperm *G*. *biloba* (Fig. 2a and Supplementary Table S2), suggesting that significant RNL contingents may be a common feature of Gymnosperms.

The data showed large variations across the land plants ranging from a single RNL sequence in Monocots to more than 30 in some of the Angiosperms without a clear pattern related to phylogeny. We retrieved TNL sequences from the same 42 plant proteomes either from prior surveys of NLR sequences or by using blastp searches (Fig. 2a and Supplementary Table S2) and performed a regression analysis between the numbers of RNLs and TNLs among the major groups of land plants. A significant relationship was uncovered, showing a 1:10 ratio (RNL:TNL; Fig. 2b). The overall regression model was significant, *F* (1, 47) = 130.36, *P* = 3.75e-15, R^2^ = 0.73 (Fig. 2b). A similar analysis between RNLs and CNLs failed to detect any quantitative relationship (Fig. 2c).

### Conifers present a high diversity in RNL gene sequences

**Figure 3:**
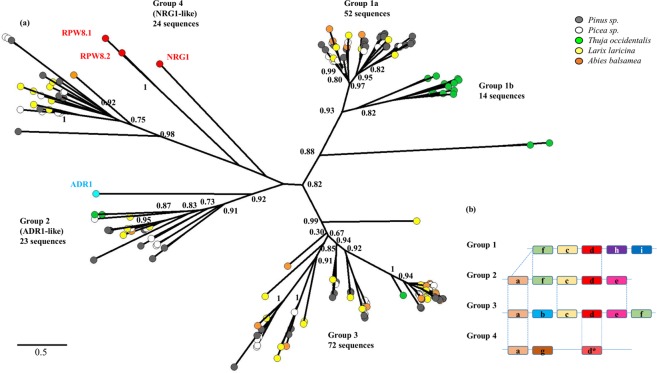
Unrooted ML phylogenetic tree and motif analysis for the RPW8 domain. (**a**) 185 RPW8 domains identified in conifers in this study together with RPW8 domains of ADR1 (blue dot), RPW8.1, RPW8.2 and NRG1 (red dots) were selected to build the ML phylogenetic tree. The number of conifer sequences contained in each of the groups are indicated below group numbers. For clarity, only main support values >0.70 are displayed (full data available in Supplementary Fig. S6). (**b**) Motif pattern diversification within the RPW8 domain associated with each group. The details of the motifs are available in Supplementary Table S3.

We tested whether the large number of conifer RNLs was associated with increased sequence diversity, which could represent an ancient sub-group expansion. We extracted and trimmed 185 full-length RPW8 domains from the seven conifer sequences to identify potential subgroups. We only retrieved RPW8 domains from RPW8-only and RNL peptides. Following the approach described by Zhong & Cheng (2016)^26^, we produced an unrooted tree including all of the full-length conifer RPW8 domains and four Angiosperm reference RPW8 domains to delineate the diversity of conifer RPW8 and assign sequences to specific groups. The resulting tree contained four distinct groups of unequal size (Fig. 3a). Two of the groups clustered with the Angiosperm sequences ADR1 (Group 2) or with the weakly supported NRG1 group (Group 4). The tree contained two other large groups (1 and 3) entirely made up of conifer sequences (Fig. 3a). Each of the seven species investigated here appeared to be homogenous in diversity among the four conifer groups. Considering the group 1, the *Thuja* sequences were entirely contained in subgroup 1b, suggesting an ancient divergence followed by recent duplications specific to this group of sequences (1) in the Cupressaceae (Fig. 3a). The group 2 represented by ADR1 had the most uniform representation, with three sequences on average per conifer species (ranging from two to five in *T*. *occidentalis* and *P*. *banksiana*, respectively).

Each of the groups was submitted to a clustering filter, which showed strong sequence clustering in groups 1 and 3 using a cut-off value of 80% identity, further suggesting multiple duplications for some of the sequences (Supplementary Fig. S3). The tree topology obtained with the representative sequences by using an identity-based clustering (cut-off of 60%) did not differ from the groups delimited with the full set of sequences (Supplementary Fig. S4). We carried out a motif analysis of the RPW8 domain in the four groups separately and found increasing polymorphism in the N and C-termini from groups 1 to 3 and a conserved central section in the RPW8 domain (Fig. 3b and supplementary Table S3). Some motifs show signs of recombination (f), loss (a, e) or *de novo* formation (b, h, i). The group 4 appeared to be more diverged compared to groups 1 to 3 (Fig. 3b).

**Figure 4:**
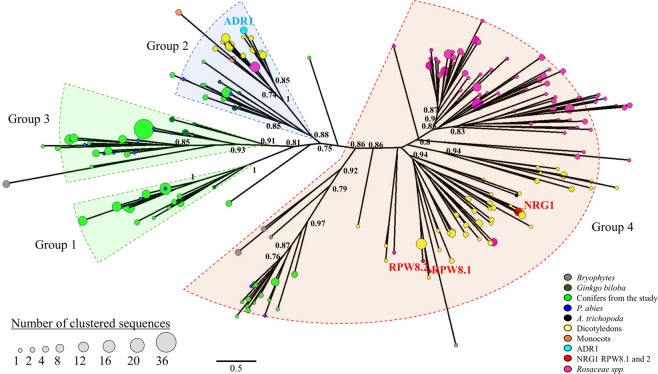
Unrooted ML phylogenetic tree of the RPW8 domain in land plants. 197 representative RPW8 domains retrieved from 50 land plants (49 species used in the Fig. 3 and *Picea abies*) were used for the phylogenetic analysis. The size of the circles reflects the number of sequences that are clustered (sequence identity cut-off of 0.60) with the representative sequence. For clarity, only main support values >0.70 are displayed (full data available in Supplementary Fig. S7). Colours are used to reveal taxonomic groups and reference sequences.

Next, we examined the conifer diversity of RNLs in view of the RNL land plant sequences. We retrieved the RPW8 domain from other plants (counted sequences in Fig. 2), together with *Picea abies* peptide sequences obtained from an independent dataset^39^, and applied an identity-based clustering (cut-off of 60%) on all of the sequences to examine the extent of RPW8 domain diversity. We thus obtained a final set of 197 RPW8 sequences to build an expanded unrooted tree. The RNL sequences generally clustered according to their taxonomic clades within each of the groups (Fig. 4). The Gymnosperm sequences *P*. *abies* and *G*. *biloba* clustered together with the conifer sequences reported in this study (groups 1–4). All of the Angiosperm sequences clustered into groups 2 and 4 (Fig. 4). All the tested Monocot sequences only clustered in the ADR1 group (2). Most of the sequences of the early land plants, presumably the ancestors of RNLs, were located in NRG1 group (4). A few sequences of *S*. *fallax* also clustered in the Gymnosperm group 3, indicating a probable onset of RNL diversification. The phylogenetic tree also highlighted some particular sequences that underwent large expansions in conifers (group 3) or Rosaceae (group 4) (Fig. 4).

### NLRs are differentially expressed under drought conditions

We investigated the drought responsiveness of NLR genes by RNA-seq analysis of samples from an independent study in *P*. *glauca* by Stival Sena *et al*.^40^. The study monitored plants of three unrelated genotypes that were well-watered (controls) or not watered at all (drought treatment) over a 22-day period, with sampling time points at 0, 7, 14, 18, 22 days. A principal component analysis of the entire dataset of foliage transcript levels showed that the drought treatment was the main factor that explained 53% of the observed variance (Supplementary Fig. S5) and supported samples groupings by treatment and genotype. The data showed major effects of the drought treatment after 18 and 22 days, independently of the clone used, in addition to smaller and more variable effects between clones at 14 days (Supplementary Fig. S5).

**Figure 5:**
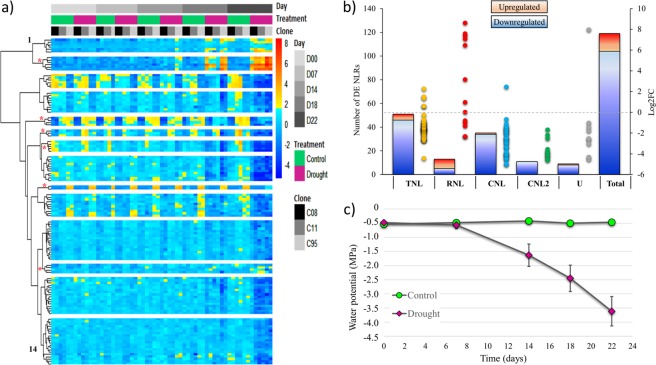
Expression patterns and distribution of drought responsive NLR genes in *Picea glauca*. (**a**) Heatmap illustrating the 14 expression groups formed of the 119 NLR sequences with drought responsive transcript levels for each genotype, treatment and sampling day. Red asterisks indicate expression groups with a single subfamily of NLR. (**b**) Number of drought responsive NLR genes and Log2 Fold Change observed in the four subfamilies and undetermined (U). (**c**) Measurement of water potential in needles for control and water-stressed plants at five sampling times. Each point is the average of 12 biological replicates (3 *P*. *glauca* genotypes with 4 replicates each) with standard deviation (adapted from Stival Sena *et al*.^40^).

We identified 119 differentially expressed (DE) NLR genes (adjusted p-value < 0.05) in drought-treated plants compared to well-watered controls across the NLR repertoire, independently of the genotype. The major changes in NLR expression were observed after 14 days of treatment and increased at 18 and 22 days, thus following the physiological trends in water potential (Fig. 5). The data showed that a large majority (>87%) of the DE NLR genes were downregulated, from slightly (the TNL, PG_019785_T.1; Log2FC = −0.25) to strongly downregulated (the CNL, PG_022220_T.1; Log2FC < −5) (Fig. 5b and Supplementary Table S4). We found that all NLR subfamilies were represented in the pool of DE sequences. A clustering analysis based on expression patterns identified 14 groups including six expression groups that only contained members of one subfamily (Fig. 5 and Supplementary Table S4).

While the DE CNL and TNL sequences were almost exclusively downregulated, 62% of the RNLs were upregulated. In total, 8 out of all the 15 upregulated NLRs belonged to the RNL subfamily (Fig. 5).

### Gymnosperm-specific RNLs have drought responsive expression

**Figure 6:**
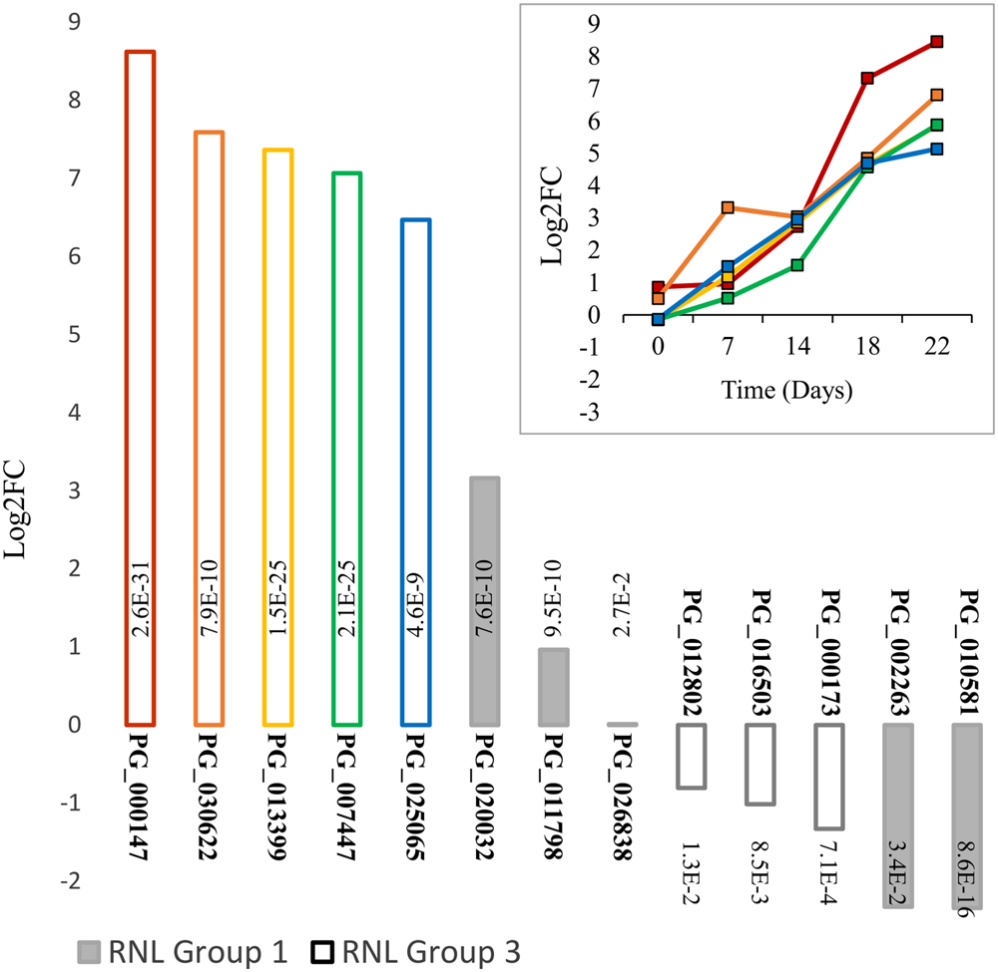
Expression of the 13 drought responsive RNLs and their RNL group assignation. The main figure shows transcript levels (Log2 fold change) after 22 days without water. Adjusted p-values are the result of the statistical tests for differential expression between drought-treated and control groups. Time course of expression profiles (Log2 fold change) are shown for the five most upregulated RNLs (top).

We identified 13 drought responsive RNL genes, including 8 upregulated (up to Log2FC >8) and 5 downregulated sequences (up to Log2FC <−2) (Fig. 6 and Supplementary Figure S9). Examining the set of five most upregulated RNL sequences, we observed a steady increase in transcript levels starting from the day 7, which was before any noticeable change was observed in the leaf water potential (Fig. 5). All of the 13 drought responsive RNLs belonged to the group 1 or 3 of sequences that are specific to Gymnosperms. None of them clustered with the ADR1 or the NRG1 group of sequences (group 2 and 4) that are in common with Angiosperms. This observation suggests that group 1 and 3 likely harbour gene sequences specifically involved in the response to drought in conifers (Fig. 6).

A genomic cluster of group 1 RNL sequences was identified based on the *P*. *glauca* linkage map^41^. The sequences were identified based on similarity (99% identity) between the full set of *P*. *glauca* RNL sequences described here and the sequences that were positioned on the linkage map^41^. Many of the RNL sequences did not produce any matches but four RNL sequences with matches were localised within 0.4 centi Morgans (cM) of each other (positions 3.86 to 4.18) on linkage group 6. Their patterns of expression ranged from downregulated (PG_010581_T.1), upregulated (PG_011798_T.1) to not differentially expressed genes (PG_012538_T.1; PG_008655_T.1) (Supplementary Table S5).

## Discussion

Most NLRs are constitutively expressed in healthy tissues and some are stress responsive in plants^20^. We produced a transcriptome-wide repertoire of NLR sequences from seven species in the Pinaceae and Cupressaceae families by RNA sequencing of several tissues and species in normal and stressed conditions. We found a large and varied NLR gene arsenal in these conifers compared to Angiosperms that have reduced or even eliminated either TNL or RNL subfamilies. We discuss interspecific variations and evolution in non-TIR NLRs including their ratio to TNLs, which may depend on their involvement in successful stress responses, resulting in their selection followed by expansion through duplications in some cases.

### Motif usage in conifers and implications in NLR evolution

Extensive studies on NB-ARC domains have highlighted their motif flexibility involving the reorganisation of pre-existing building blocks that can be observed at a domain or motif level^16^. In our dataset, we observed conserved motifs (P-loop, Kinase 2/3, and GLPL) involved in crucial ADP/ATP binding and shifting^42^. These motifs are conserved in all NLR subfamilies and in NB-ARC of prokaryotes, which do not display upstream N-terminal domains^16^. Thus, these motifs are not influenced by the N-terminal domain identity. The over-representation of RNL sequences in several conifers allowed us to define reliable NB-ARC_RNL_ signatures. We showed that RNL proteins strictly required a glutamine, first residue of the MHD motif in Fig. 1 (Q1) in place of the methionine (M1) (i.e. QHD). This glutamine residue was first identified in *Arabidopsis* ADR1 homologs^43^ and probably possesses a functional role through its ability to create hydrogen bonds possibly involved in ATP processing. Indeed, mutations on H2 or D3 residues in the MHD motif (Fig. 1) favour ATP binding leading to the auto-activation of NLR and cell death^44,45^. The shift in sensitivity between the ON/OFF states is crucial to confer a finite lifetime to active forms and reliably unlock OFF states initiating safe cell death^46^. The RNBS-D motif is shaped according to the N-terminal domain identity but its exact function remains unclear^19^. By contrast to other NLR subfamilies, RNLs did not harbour a conserved cysteine (C5, C6 or C7) but a conserved glycine in the sixth position (G6) of the RNBS-D motif (Fig. 1). The ability of cysteine residues to form disulphide bonds may play a role in intramolecular interactions whereas the glycine in RNLs could reduce the steric hindrance. Our motif analysis showed that CNL2 sequences display polymorphic motifs and an intermediate RNBS-D form that resembles that of the archaic kinase-NB-ARC-LRR in *P*. *patens*^10^. These findings and the near absence of CNL2 sequences in plants other than conifers suggest that CNL2 could represent an intermediate form in the evolutionary swaps between NLR subfamilies.

### Diversity and drought responsiveness of RNL genes

The data reported here showed that conifer RNLs include sequences that are distinct from those previously described in addition to members of the well-known ADR1 and NRG1 groups^26,27^. The presence of ADR1-like sequences among all flowering plants and the absence of NRG1-like representatives in some taxonomic groups reinforced the previously confused state of knowledge^27,43^. We showed that conifer RNL genes split into four groups, two of which correspond to the only known Angiosperm groups (groups 2 and 4) while the two others (groups 1 and 3) are Gymnosperm-specific (Figa 4, 5). Our results are at odds with the suggestion of a single independent Gymnosperm group^27^ and places the NRG1-ADR1 differentiation before the divergence between Angiosperms and Gymnosperms, at more than 300 Myr ago^47^. RNL sequences from *G*. *biloba* and *P*. *abies* support this interpretation (Fig. 5).

Although they are usually found in low numbers, we detect that ADR1-like sequence group has a few sequences in all of the seed plants tested to date, which contrast with other RNL groups. Taken toghether, the absence of plant species lacking ADR1-like sequences, the involvement of ADR1 in Arabidopsis thaliana drought tolerance and its identification as a cornerstone in NLR signalling^31^ clearly suggest that ADR1-like sequences are crucial for plant survival and fitness. Considering the phylogenetic distance between species, the compact aspect of the corresponding branch in the phylogenetic tree supports a significant sequence conservation previously documented in flowering plants^43^. It has been suggested that ADR1-like proteins carry out a general function in disease resistance or play a critical role to control common pathogens^27^. Its characteristics may also fit with a role in adaptation to abiotic stresses that affect most plants in their natural habitats, or a hub function acting downstream of a sensing network. In contrast, the heterogeneous NRG1-like group is made up of more polymorphic sequences, which is reflected by its scattered distribution in the phylogenetic tree (Fig. 4).

Most importantly, our data identify two Gymnosperm-specific groups (1 and 3) of RNLs that may harbour many recent duplications based on their lower levels of sequence divergence. The RPW8 domain motif organisation in these latter two groups shows relatedness with ADR1-like sequences, suggesting a shared origin with the ADR1 group. The extensive duplication and diversification of RNLs in conifers argues in favour of a role in adaptation to ubiquitous stress such as harsh environmental conditions.

Our RNA-Seq analysis in *P*. *glauca* suggests that the majority of differentially expressed NLRs are downregulated after a water deprivation of several days, which is in line with increased susceptibility to pathogens in stressed conifers^48,49^. Interestingly, only RNLs from groups 1 and 3 appeared to be responsive to drought. Specifically, a subset of RNLs from group 3 were strongly overexpressed after drought treatment and few sequences were slightly downregulated, as observed for a majority of stress-responsive NLRs. In *Arabidopsis*, ADR1 was shown to be actively involved in drought tolerance^7^. Here we report for the first time in conifers, RNL sequences from a nearby group of ADR1 that respond significantly and early to drought stress. Their genomic localisation indicates that some of them may be clustered in tandem arrays. The comparative study of drought-responsive RNL clusters among clones or species that show differential resilience to drought should be of interest. Analyses of copy number variation, gene organisation, sequence diversity or expression among these clusters may reveal molecular mechanisms acting in adaptation to drought in conifers^50^.

### RNL origin and distribution in plant lineage

The recent sequencing of ancient genomes has shed light on the origin of NLR proteins^15^. The RNL subfamily arose before the Gymnosperm - Angiosperm split^27^ putatively *de novo* from a non-coding region of an NLR-encoding gene or following domain fusion^26^. No RNLs are mentioned in green algae^15^; nevertheless, we identified a few RNL sequences in the tested early land plants, including the liverwort *M*. *polymorpha*, and in the mosses *P*. *patens* and *S*. *fallax*, which harboured typical NB-ARC_CNL_ but no NB-ARC_RNL_ motifs. In these three species we also identified other RPW8-containing proteins. NB-ARC_RNL_ signatures were detected in all tested seed plants, including ancient plants such as *G*. *biloba* and *A*. *trichopoda*. It is possible that a pre-RNL emerged from a pool of CNL sequences in which the fusion of the RPW8-encoding exon occurred early in land plant followed by a transition toward RNL forms that arise before the divergence between Gymnosperms and Angiosperms. However, a study aiming to confirm this hypothesis and to decipher the RNL evolutionary history should include more sequences from ancient plant lineages (e.g. green algae, mosses, lycophytes, and ferns) but also other Gymnosperm sequences from Cycads and Gnetophytes. Expansion of the RNL sequence family is observed in groups 1 and 3 in the conifers and in group 4 in the Rosids. By contrast, taxonomic groups such as the Monocots have only a single ADR1-like RNL and have lost any other group representatives. A striking co-absence of NRG1-like and TNL sequences has been highlighted in Lamiales (a subgroup of Asterids), early-diverged Dicots (*Aquilegia coerulea*), and Monocots^27^.

We identified a high abundance of both RNL and TNL genes in conifers. We also observed that the number of TNL genes correlates well with that of RNLs but not with that of CNLs in diverse seed plants. The quantitative link between TNLs and RNLs (approximate ratio of 1:10, RNLs:TNLs) is based on 49 plant species, despite potential variations in determinations of gene numbers among species. This asymmetrical ratio linking the abundances of RNLs and TNLs is surprising especially given that the two subfamilies are probably not physically linked, e.g. the chromosome 7 of *Prunus persica* contains 80% of the RNL pool but only 5% of the TNLs (Supplementary Table S6), and the dynamic NLR family has undergone several gene expansions and losses during evolution^11^. In Angiosperms, the functionally characterised RNLs NRG1 and ADR1 act as downstream helpers of conventional sensor NLRs^29,51^. NRG1 acts downstream of the activation of the TNLs N, Roq1 and RPP1, but not the CNLs Bs2 and Rps2^28,30^. ADR1 and its paralogues are also thought to act in conjunction with TNL and CNL sensors^29,31^. Recently, an extensive study revealed a larger NLR network involving numerous TNLs, including pair TNL pairs, and a few CNLs that signal via NRG1 and/or ADR1^31^. In this context, the proportionality between TNLs and RNLs may be explained by the structure of their functional relationship (*i*.*e*. multiple sensors that signal via few helpers). RPW8 domains recruit TNL gene-signalling components such as EDS1, PAD4 and EDS5, but do not recruit CNL gene-signalling components^52^. New findings showed that signalling machineries, including EDS1, PAD4 and SAG101, co-evolved within plant species and clades for regulating downstream pathways in TNL immunity^53^. A recent study that highlighted the loss of numerous NLRs and their gene-signalling components in aquatic Angiosperms paves the way to decipher the complex interactions between the plant immune system and drought tolerance^54^. All together, these results support the hypothesis that RNLs may function redundantly in network, together with TNLs to activate NLR-triggered immunity. By contrast to the complex network recently identified in Solanaceae that is exclusively composed of sensor-helper CNLs^55^, the complex TNLs/EDS1/RNLs might have evolved independently to provide a parallel machinery that triggers immunity especially in Rosids and in Gymnosperms.

## Methods

### Plant material and RNA extraction for transcriptomes

The study relied on four year-old saplings and germinated seeds of seven conifer species, including two spruces (*Picea glauca* and *Picea mariana*), two pines (*Pinus strobus* and *Pinus banksiana*), a fir (*Abies balsamea*), a larch (*Larix laricina*) of the Pinaceae family and a representative of the Cupressasseae family (*Thuja occidentalis*). All of the individuals were obtained from open-pollinated seed lots. The saplings were cultivated under standard greenhouse conditions^56^ and included four individuals per species. Before the sampling, we deprived half of the saplings of each species of water for 14 days and watered the other half normally. We sampled six vegetative tissues from these greenhouse grown plants. Total RNAs were extracted from the different tissues by using the protocol described in^57^ with modifications^58^, and the MasterPure™ Plant RNA Purification kit. The detailed protocol is available in Supplementary Methods.

### RNA-Seq library synthesis, sequencing and assembly

We sampled different conifer tissues and applied a drought stress to generate a maximum number of RNAs. We pooled all of the RNAs from the different tissues for each species separately (Supplementary Methods). We used 500 ng of total RNA from each of the species pools to synthetize mRNA libraries using TruSeq® Stranded mRNA kit following the supplier’s instructions. Second strand synthesis and post PCR clean-up were performed using Axygen® AxyPrep™ Mag PCR Clean-Up Kit. Each library was quantified using a Nanodrop ND-1000 and characterised with an Agilent Bioanalyzer 2100 using High Sensitivity DNA chips. The Genome Quebec Innovation Centre at McGill University (Montreal, Quebec, Canada) carried out the sequencing of each pool using the “rapid run” procedure (paired-end 250 bp) with an Illumina HiSeq 2500 sequencing system.

The transcriptome assemblies were performed with Gydle software NUCLEAR version 3.2.16, RESOLVE version 2.6.12 and VISION version 2.6.12, and guided using the white spruce gene catalogue obtained by the sequencing of full-length insert cDNAs (FLICs) from white spruce cDNA libraries as described in^59^. The detailed protocol of assembly is available in Supplementary Methods. The accessions numbers for all of the transcriptome assemblies and the sequence reads are mentioned in the additional data and a detailed list is available in Table 2 of the Supplementary Methods.

### Functional annotation and motif discovery

For each of the seven conifer species, we applied a first filter by analysing the entire predicted protein dataset using the Pfam database with a cut-off value of 1.0. Sequences displaying the Pfam entries NB-ARC (PF00931), TIR (PF01582), RPW8 (PF05659) and LRR 1 to 6, 8, 9, and LRRNT (PF00560, PF07723, PF07725, PF12799, PF13306, PF13516, PF13855, PF14580 and PF01462, respectively) were extracted. Then, we characterised the domain organisation using HMMER web server version 2.19.0 with the selection of Pfam, TIGRFAM and superfamily HMM databases with significance e-values set to 0.01 (model) and 0.03 (Hit)^60^. Atypical NLR sequences were also verified with InterProScan by using the entire database available. Motifs analysis and scanning were carried out using the MEME suite^61^ with maximum number of motifs set to 10, minimum-maximum motif width set to 5–30 and 5–50 and minimum sites per motif equal to 2.

### Additional data sampling and extraction data

The full list of additional plant protein datasets and the versions that were screened is available in Supplementary Methods. RNL sequences were extracted using BlastP searches with (i) RPW8 domains of ADR1 (BAF00531.1), NRG1 (AAY54606.1), RPW8.1 (ACJ05907.1), RPW8.2 (ACJ72031.1) and a representative of each of the four conifer RNL clades detected in this study and (ii) NB-ARC_RNL_ laying between the RNBS-D and the QHD motifs as queries with broad e-value < 1. When TNL numbers were not available in the literature, we produced our own estimates by using BlastP (e-values < 10^−8^) searches with N (Q40392.1), RPS4 (CAB50708.1) and Ma (CAR94514.1) sequences deprived of LRR, as queries. BlastP (e-values < 30) with PL motifs^22^ were used to retrieve PL domains. All extracted sequence identities were verified using HMMER with default parameters and NB-ARC and PL motifs were aligned to be evaluated.

### Sequence alignment, clustering, and phylogenetic reconstruction

Sequence alignments were performed with Muscle^62^ using default parameters and trimmed using MEGA7^63^. Clustering analyses were conducted with CD-HIT^64^ using default parameters and variable sequence identity cut-off values: 0.97 for elimination of redundant sequences (allelic sequences or alternative transcripts) leading the total number of sequences to be reduced to 3734, and 0.50–0.90 for the conifer RPW8 domains. To identify RNL groups in conifers, 185 full-length RPW8 sequences (>100 amino acids) were extracted and trimmed from our dataset. These RPW8 domains together with RPW8 domains of ADR1, RPW8.1, RPW8.2 and NRG1 were aligned to undertake the first phylogenetic analyses of conifer sequences. Then, the conifer sequences were clustered using a cut-off value of 0.60 to obtain 45 representative RPW8 sequences. Simultaneously, a second dataset was prepared using 351 Angiosperm, 13 *G*. *biloba* and 17 *P*. *abies* RNL sequences. These sequences were used to similarly extract full RPW8 domains (>100 amino acids) and clustered (cut-off values: 0.60) to obtain 152 representative RPW8 sequences for this second dataset. The 197 representative sequences were included into the phylogenetic analysis conducted using the web service http://www.phylogeny.fr^65^. The phylogenetic trees were reconstructed using the maximum likelihood (ML) method implemented in the PhyML program (v3.1/3.0 aLRT). The WAG substitution model was selected with an estimated proportion of invariant sites and 4 gamma-distributed rate categories to account for rate heterogeneity across sites. The gamma shape parameter was estimated directly from the data (gamma = 3.981 and gamma = 4.611). Reliability for internal branch was assessed using the aLRT test (SH-Like). The phylogenetic trees were then drawn using Dendroscope 3^66^. Similarly, the JTT substitution model was used (with Gamma shape parameter: 3.648) resulting in the same tree topology (Supplementary Fig. S8).

### Transcriptomic analysis of drought stress in three *P. glauca* clones

Foliage samples from the drought experiment in *P*. *glauca* described in Stival Sena *et al*.^40^ were analysed by RNA-sequencing. The samples were collected at 0, 7, 14, 18 and 22 days from the beginning of the watering treatments. We used three unrelated and clonally-propagated genotypes (clones 8, 11 and 95) each with two replicates, in two conditions (well-watered control and drought with no watering) with five different sampling time points, and obtained 59 RNA samples to analyse (lacking one control replicate for the clone 95 at the time 7 days). The detailed protocol for RNA extraction and RNA-seq library synthesis modified from Stival Sena *et al*. (2018)^40^ is available in Supplementary Methods. The pooling of 59 libraries into one lane for sequencing produced variable numbers of sequence reads per library (Supplementary Fig. S5). In order to limit the effect of size library, we added a filtering step keeping only genes with counts >5 in at least two of the libraries. A principal component analysis (PCA) also showed that replicates clustered mostly together, indicating that plants from the same treatment had similar profiles and suggesting that variation in library size had little impact on expression profiles (Supplementary Fig. S5). DEseq2 v1.20.0 package in R software (Core Team 2018) was used to normalize count data. We calculated the differential expression between the treatment and the control foliage samples with the DEseq2 v1.20.0 package and the LRT approach. A lists of differentially expressed NLR genes with a FDR adjusted p-value < 0.05 are shown (Supplementary Table S4). Cluster v2.0.7., factoextra v1.0.5, and pheatmap v1.0.10 packages were used to generate heatmap with gene clustering analyses using manhattan distances and the ward.D2 clustering method^67–76^.

### Accession numbers

The raw data from the seven conifer transcriptomes are deposited in the European Nucleotide Archive (ENA) as part of the study SRP134160, bioproject PRJNA437248 (SRA accession SRR6816977 to SRR6816983). A list of accession numbers is available in supplementary methods.

## Supplementary information

Supplementary Information

Dataset 1

Dataset 2
