# Supplementary material for: The large repertoire of conifer NLR resistance genes includes drought responsive and highly diversified RNLs
Source: Sci Rep. 2019 Aug 12;9:11614. doi: 10.1038/s41598-019-47950-7 (PMC6691002; doi:10.1038/s41598-019-47950-7)
Supplement: Supplementary file 1 — Supplementary Information [file 41598_2019_47950_MOESM1_ESM.pdf]

The large repertoire of conifer NLR resistance genes includes drought responsive and highly diversified RNLs

Cyril Van Ghelder, Geneviève J. Parent, Philippe Rigault, Julien Prunier, Isabelle Giguère, Sébastien Caron, Juliana Stival Sena, Annie Deslauriers, Jean Bousquet, Daniel Esmenjaud, John MacKay

Supplementary files

Supplementary Methods: Detailed methods for the plant material, RNA extraction, RNA-Seq library synthesis, sequencing, assembly and additional data sampling.

Supplementary Data S1: NLR peptide sequences from the seven conifer transcriptomes.

Supplementary Data S2: Functional annotation of the NLR peptide sequences from the seven conifer transcriptomes (excel file).

Supplementary Figures:

Supplementary Figure S1: Alignment of Post-LRR domains
Supplementary Figure S2: Alignment of representative RNL sequences
Supplementary Figure S3: progressive clustering of the RPW8 domains in seven conifer species.
Supplementary Figure S4: Unrooted ML phylogenetic tree of the RPW8 domain in conifers after using 60% sequence identity cut-off and clustering using the WAG model
Supplementary Figure S5: Library size of the transcriptomes and Principal component analysis of the data from the study of Stival Sena et al. (2018)
Supplementary Figure S6: Detailed ML phylogenetic trees corresponding to the Figure 3
Supplementary Figure S7: Detailed ML phylogenetic trees corresponding to the Figure 4
Supplementary Figure S8: Unrooted ML phylogenetic tree of the RPW8 domain in land plants using the JTT substitution model
Supplementary Figure S9: Count number average of the five most differentially expressed RNL sequences

Supplementary Tables:

Supplementary Table S1: Ratio of NLR to the total number of transcripts in our study and other plants
Supplementary Table S2: Details of the NLR subfamilies numbers in 49 land plant species used to build the figure 2
Supplementary Table S3: Details of the RPW8 motifs identified in Figure 3

Supplementary Table S4: List of the 119 differentially expressed NLR genes

Supplementary Table S5: Position of the RNL genes on the *P. glauca* composite linkage map of Pavy *et al.* (2017)

Supplementary Table S6: RNL and TNL genes present on the chromosome 7 of the peach genome.

Supplementary Figure S1. Alignment of Post-LRR motifs in *Amborella trichopoda, Ginkgo biloba* (Gb) and five reference TNLs in dicots.

```
                                                 Motif 1                                   Motif 2
                                   10        20        30        40        50        60        70        80        90       100
                          ....|....|....|....|....|....|....|....|....|....|....|....|....|....|....|....|....|....|....|....|
motifs                    --------------------------P-GSEIPEWFSHQSVGD------------------------~~~~~~KWMGLALCAVFE---------------
Gb_20740                  SGSSVSVCNLNKWIREV-PSLQELNIY-GSSVPLWFRHQMHGQLDGKE--------HARDLCLDVPIRVKPNVKIEAVIFCFVSHD--QGWSDYEWDHGR
Gb_06500                  SGCGVLKVDFLKWIKGM-PLLRELSFY-ASEIPEWFDHRMKTKFFVK---------DFPRLCMDRVSMIFDEGRYAGTDVGYM-----------------
A.trichopoda00001.586     -GCSPHTLRGQSLAKETFTSLDKLSIP-GSKVPDWFMFQTP--------------------SFTVPRLSDVGLHIREVLLCFSYS-----TYDTSP----
A.trichopoda00109.130     -GCSPHVWRGQNLAKEMFTSLVQLSVP-GSKVPNWFACKCTPRCSLIQDGE-----MLQQVSCALPRPSHAGRRIKEIMVCLVIY-----VHHSSYTR--
A.trichopoda00056.119     -GCKPHVLRGQSLLEEMFTGLYSLSVP-GSKVPNWFMCKCTSRYIRIQDIENPDWFMYQTLSCTVSRISEEGLHIREMMVWFVIY-----VNELSCTH--
A.trichopoda00085.83      -GCGSYVSNSPGLGKETFIQLRHFGVS-GSKVPSWFMFQTL--------------------SCVIPRVSDEGLKVRGVILCLVLS-----VDSEMPIDDG
A.trichopoda00085.87      -GCSPRVSRS--IAKETFNHLWCFGIS-GSKVPDWFMFQKL--------------------SCTVPRLSDQDLKIRGVILCFVIS-----HNSEIPIDFV
A.trichopoda00085.80      -GCT-HPSRS--IAKETFEHLRCFGVS-GSNVPDWFMCQSL------------------~~SYMISGMSDHGVEIRGVILCLVIS-----LDNEIPTEID
N(N.g.)Q40392.1           --QNISSMRHDISASDSLSLTVFTGQPYPEKIPSWFHHQGW----------------DSSVSVNLPENWYIPDKFLGFAVCYS----------RSLIDTT
Bs4(S.l.)AAR21295.1       -FLNISSFQHNISASDSLSLRVFTSL--GSSIPIWFHHQG----------------TDTSVSVNLPENWYVSDNFLGFAVCYY----------------G
RPS4(A.t.)CAB50708.1      --QLLSYARKRYNGGLVSESLFSTCFP-GCEVPSWFCHET----------------VGSELEVKLLPHWHDK-KLAGIALCAVVS-------CLDPQDQV
Gro14(S.t.)AAP44390.1     -TSMVDSLLKQMLEALYMNVRFCLYVP-GMEIPEWFTYKSW---------------GTQSMSVALPTNWFTP-TFRGFTVCVILDKKMLFILGRFNTHKV
Ma(P.c.)CAR94514.1        -----DAIRR--------DERFEYGYR-SNEIPAWLSRRS----------------TESTITIPLPHDVDGKSKWIKLALCFI---------------CE

                                  110       120       130       140       150       160       170       180       190       200
                          ....|....|....|....|....|....|....|....|....|....|....|....|....|....|....|....|....|....|....|
motifs                    ----------------------------------------------------------------------------------------------------
Gb_20740                  YNSNTWMEATIIRGE--EEVHNTRILR----NRHADRRDQLHLSVYREG----HPFVMN--LQNGDRIRV--------------------------WARS
Gb_06500                  -------KATIVRGG--QEVFETVFIRI----REANRRDQLYLSMYLEN----DSFVKR--LHSGDRIRI--------------------------RVCS
A.trichopoda00001.586     --GSPRSMVSIKRED--RGIRIEEQVDIQYFPLS--HQNQVFFRRLRHGHMP-RLWFKW--FKDENQIEL--------------------------LTKF
A.trichopoda00109.130     --NRHVVGLWIKRED---ETL--PQIPLQIYPVTNLNQNEIYFYRFREGQYY-GNRKFW--LEDGDEIEVEYDPIQCRCQAADVDEPTECEDQVEDIEEL
A.trichopoda00056.119     --QKHVVNLLIKRKG-----REKDRLPLGIYPGT--HRNQIYIYHFRDGIGF-EFQMNW--LKDGDQIEF-------------VSESTYYEDQIEVINES
A.trichopoda00085.83      L-DIPHVQFKVMNNG---MQRMSPTLKLGN-PKA--NQDQVYLYHFQED----DALVLN--LEDGDEVKI--------------------------INNC
A.trichopoda00085.87      P-DIPDVLFMIIRNG---TRRFSTMLRLGV-PKA--HQDQVYLFHFQEG----GHILPM--LEGGDQIEV--------------------------AKRS
A.trichopoda00085.80      DWGIPDVELTITRDG---IERFSSTLRLEMLPKA--RQDQVYLFHFQEG----DDVLLM--LGDGDQIEV--------------------------LNRS
N(N.g.)Q40392.1           AHLIPVCDDKMSRMT--QKLALSECDT----ESSNYSEWDIHFFFVPFA----GLWDTS--KANGKTPND--------------------------YGII
Bs4(S.l.)AAR21295.1       NLTENTAELIMSSAG-----MPCITWKLLLSNHSECTYIRIHFFLVPFA----GLWDTS--NANGKTPND--------------------------YKHI
RPS4(A.t.)CAB50708.1      SRLSVTCTFKVKDED---KSWVAYTCPVGSWTRHGGGKDKIELDHVFIGYTSCPHTIKC--HEEGNSDEC----------------------NPTEASLK
Gro14(S.t.)AAP44390.1     YGLENMIWLNLKRYDGLRQKISTSFGPIG--SEKPGGLGDTLITHIAFE----RSWK----LENDLDYYR--------------------------NNAF
Ma(P.c.)CAR94514.1        AAQKHDSLEDVPEFDEELGLKFTRNHRIEL-CTTEDPHERLLALDYRDCNFA-GPFIHWCFIPQSDLAES--------------------------SNKR

                                         Motif 3
                                  210       220       230       240       250       260       270       280       290       300
                          ....|....|....|....|....|....|....|....|....|....|....|....|....|....|....|....|....|....|....|
motifs                    ---------~~~~~VKKCGVRLVYEQDVEELNQT------------------------------------------------------------------
Gb_20740                  IYA------AWRIWVKEGGMHILYREGEEKHEQDSLCTNIILYNHPSYTIHSNSAHKYAISL------------------------
Gb_06500                  QSI------SHQICVKQGGMHLLYTKEETTTSNNKKDLNDTVFERLSKDLTWLHWWNVVWDKHVPIHQMDIKSFIAYDLQSCFIHRQTATLKPSVMHASR
A.trichopoda00001.586     ---------MVCL---KGAMHLVYKPADTDESIQERLANFFNPLLSE-----------------------------------------------------
A.trichopoda00109.130     RAS---SLRLPCVRLKKVGIHFIYESEDTEDSTEEKLANFFNSL--------------------------------------------------------
A.trichopoda00056.119     GPSLTPSLGFPCYHLKQAGIHLVYESEDTEDSIEEKLADFFNSLHVQWQLTASLYVRYPFWFRIATPYFLCGIWILK---------
A.trichopoda00085.83      PPL------TPSIHIKKVAIHLVYKPEDTKDSIQEKLAEFFNSMDAHEDALEIVQFHSLQAHNLQTYHPERRMRSGILLLVVILCCFYFWMM--------
A.trichopoda00085.87      PPM------FPSIQLKKGGIHLVYKPEQTQDYIQEKLAEFCNSMDCHENLPPHYLRRKVQAGILLWVFVLLYFVWMRSNSENYSPFLPSLMTYSALYALK
A.trichopoda00085.80      PPE------FPCVQMKKGGIHLVYKPEDTHESIQEKLADFFNSLNCHDSS--------------------------------------------------
N(N.g.)Q40392.1           RLS------FSG-EEKMYGLRLLYKEGPEVNALL-QMRENSNEPTEHSTGIRRTQYNNRTSFYELING--------------------
Bs4(S.l.)AAR21295.1       MLS------FPQ-ELKECGVRLFYEDESVLETTNDELTIGVRRIRYDDDDSEHYEEAGCSSSKKQRS---------------------------------
RPS4(A.t.)CAB50708.1      FTVTGGTSENGKYKVLKCGLSLVYAKDKDKNSALETKYDMLIGKSFQETSEGVDGRVKKTKGKYVMPVEKNFQETTEGVDGRVNKKKKTRMDNGRPKKKQ
Gro14(S.t.)AAP44390.1     QLEFSACDHYQKDVVKGLGVRLVYEN--------------------------------------------------------------------------
Ma(P.c.)CAR94514.1        LIQATITPDSPGTRVTGCGVSLIYLEDVPKFVRKLNKHYSYCYHGYQPEQEEDGMRSILSTSGIQTEQEEVQEQETTTSTRIVGQLRRNVESLLEKLFE-
```

Supplementary Figure S2: Alignment of representative RNL sequences from basal land plants, gymnosperm, angiosperm, monocots, dicots and the reference RNLs (ADR1, NRG1, RPW8.1 and RPW8.2)

```
                                     10        20        30        40        50        60        70        80        90       100
                                 ....|....|....|....|....|....|....|....|....|....|....|....|....|....|....|....|....|....|....|....|
P.patens_Pp3c10_50V3.1           -MGDPLVAPALVGFGVNL--LLTEVIKAGRMAYRCKDECNELVAKLESVRPLVEQV-NDNSSKGNEAC-----TLWLSG-FQSLLERAKVVTNECKLE--
M.polymorpha_Mapoly3272s0001     ---MAELVSVVIGFGVEL--LLQGVIDEVKQAVDRNKNCKELLVLLEGLAPLVQFL-CDSSKKGQGQNKSTPVTEWLEK-LRSRLEEADKVVRECISQGS
S.fallax_Sphfalx0171s0003.1.p    ---MLIEPGLVFGILGQLQPLLNRVIDAVEITLKYDKSCTKLSSLLLRLQPLLQHI-ANQAPNLSPNV--SLVHTWLTE-LEKRLKAAETTLNVSNEQHP
G.biloba_Gb_38648                --MGDLLSQTLAGVVATE--LLSVVKDVAKKCYGCKSYCKRLEKTLEGIIPILDEI-FQTGAEVSQHR-----QRELKE-LYEKLQKGIELVENCSKV--
A.trichopoda_scaffold00033.264   ---MDLVSGAIIGAIAGE--ILKALLDATNIVYECKSQCEKLKSTLQHIVPKLEDI-EQLRLQLTDCE-----PADFKE-LKSRLESGKVLVYKCMKL--
A.coerulea_Aqcoe5G206900.1       ----MAVTDFFAGEIATE--LLKQLFQITRKCSLCRASAEQLMISINELLPIITEI-KYSGVELPQGR-----QNHLDR-LSETLTAGRELAVKVLSS--
P.dactylifera_XP_008792596.1     ------MDTFFAGEIATE--LVKELVKVIRRTVLCRPTAEQLKRNVDALLPIIQEI-RHSGVELPQAR-----QSQLSD-LAEHLHLALELARKAAAS--
A.comosus_Aco004926.1            ------MDTLFPGEVATE--LLRELAKVVRRAYGCRATAEQLKRGVEALLPIVQEI-RYAGVELPQPR-----QRQLSE-LAERLARGLELARAAAAA--
M.acuminata_GSMUA_Achr7T08250_   ------METFFAGEIATE--LVKELLKVVRRTYLCRPAAEQLKRSVDALLPIVQEI-RHSGVELPQHR-----QSQLSE-LADQLRLALDLARKAAAS--
N.benthamiana_NRG1_AAY54606.1    -MAATLLGGAALGPVFDI--LLKAVLDVGIKIATFRSKFQSLIKTLNDIKPVFDDI-ERLNKALDGRD------YEIEM-FKKQLFAGEELVRKCSKT--
A.thaliana_ADR1_BAF00531.1       ---MASFIDLFAGDITTQ--LLKLLALVANTVYSCKGIAERLITMIRDVQPTIREI-QYSGAELSNHH-----QTQLGV-FYEILEKARKLCEKVLRC--
A.thaliana_RPW8.1_ACJ05907.1     MPIGELAIGAVLGVGAQA--IYDRFRKARDISV-----VNRLCATIISIRPLLVQIDKRSKVEGSPLR-------EVNERLTCFLELAYVFVEAYPKL--
A.thaliana_RPW8.2_ACJ72031.1     -MIAEVAAGGALGLALSV--LHEAVKRAKDRSVTTRFILHRLEATIDSITPLVVQI-DKFSEEMEDSS-----SRKVNKRLKLLLENAVSLVEENAEL--
                                                                          RPW8 DOMAIN

                                    110       120       130       140       150       160       170       180       190       200
                                 ....|....|....|....|....|....|....|....|....|....|....|....|....|....|....|....|....|....|....|....|
P.patens_Pp3c10_50V3.1           SRWSRRKRMVKFWRWKKLPSLVMKI---------------SSDIEEARSNTSLVTLGIVLSMHIPKQVEAFSFVKQ------------------------
M.polymorpha_Mapoly3272s0001     RPWNIVKNRSTSKKLVEVTRGITDLVGQAPLVQLGYTKETTDEQNKILADLQESMKNANQLHSDLIHEVHCA--DLSQITRVTVNTPTEIFGRSYYGTIT
S.fallax_Sphfalx0171s0003.1.p    RLTAPVSRYRAASEINKLTESVEELLKQADLVTVDLALSISAKVENATEDTKHALQSVENQLSIVTRQLPNVRRQLTDVTRQCLKPSIAKSWNIEEATVA
G.biloba_Gb_38648                SCLNMYQRYKFAKRILALEKYISSF------SDKQGWAHVMSDLHHLRVDTGRRLEYIEKQVEGNHECILFEQMMKDQMPLFGIGSEASYPNLQIP----
A.trichopoda_scaffold00033.264   RFWKVHKKYSYGKKLIELDEYLVRF------FDLNLAADTNLDTKQLRIE----FKEMRRLLDGRGIASESSYALP------------------------
A.coerulea_Aqcoe5G206900.1       NRWNVYKNLQYARKMEKLEKNVSRF------INGPLQFHVLADVHHLRVG----LDRLEEKIGIDKLEERIGGLRI-----------EGNGWVEESMRRK
P.dactylifera_XP_008792596.1     SRWNVYRSMQLAHRMEKLDRWIARW------LERQMPAHVLADVHHLRVESAVRLDRIERHLQNGAPATAAAAAAMTMMKPAAVGVAQMMEGLELGGGG-
A.comosus_Aco004926.1            PRWNVYRSVKLAREMEKVERWIARW------LQRQMPAHVLADVHHLRVDAEVRMERIERRIDHVEAAAAAAAAAAAPGPVVGVRAPSSAAKGAGEMMMM
M.acuminata_GSMUA_Achr7T08250_   PRWNVYRSMQLAHRMERLDRWISRW------VERHMPAHVLADVHHLRVDYSARLDRIERTLDMTAASAALAAARV----PVAVGSVPFSGSPLTEMMDG
N.benthamiana_NRG1_AAY54606.1    KCYDALKKWNYSRKLTKLENSLVRF------CQVHGFIQVCRDSKIILVNVIEHGKKLDQITSMLRGISLRNGSSI-----GFTNSNGSSGWMNGNSFGS
A.thaliana_ADR1_BAF00531.1       NRWNLKHVY-HANKMKDLEKQISRF------LNSQILLFVLAEVCHLRVNGDRIERNMDRLLTERNDSLSFPETMMEIE---------------------
A.thaliana_RPW8.1_ACJ05907.1     RRRQVLRKYRYIKAIETIELALRSIIVVDFQVDQWDDIKEIKAKISEMDTKLAEVISACSKIRT
A.thaliana_RPW8.2_ACJ72031.1     RRRNVRKKFRYMRDIKEFEAKLRWV-VGVDVQVNQLADIKELKAKMSEIRTKFDSTS
                                                                          RPW8 DOMAIN

                                    210       220       230       240       250       260       270       280       290       300
                                 ....|....|....|....|....|....|....|....|....|....|....|....|....|....|....|....|....|....|....|....|
P.patens_Pp3c10_50V3.1           -----------------------------------------GTPSLIVGVDEHFKKLKMSILQSHGGDKASHFGLQGRGGAGKTLLVQMLNNDDDIQMKY
M.polymorpha_Mapoly3272s0001     LERAFEDIRAQADEHFLTQTQGSATGSSADVFPSPQHQFERQISARVFGGDELLDSARSILL---EEDGGQWVGLWSMGGAGKTLLASRLSNDLPLKQHF
S.fallax_Sphfalx0171s0003.1.p    IITAFQNLNASDDHDNAMEAVPLTLATIDPQQPSPKL----HVPQFVFGLDDSIGRLQQLLLSRHSDPDPHSIGVWGKGGVGKTLLARKVHNSMEVRQHF
G.biloba_Gb_38648                -----------------------------------------EMPEHPVGLKISVEELKKRLF----PQDVSLISVKGMGGSGKTTLAVALCSDQQVQEFF
A.trichopoda_scaffold00033.264   -----------------------------------------GLPDCPVGLSDSLRELKAKFF----ADDVSVLGICAQGGYGKTTLASMLCREEDVKGRF
A.coerulea_Aqcoe5G206900.1       VEEE-------------------------------------EKVVNLIGVGMVLAKKKMMEV-VVGRDDLKVIGICGIGGSGKTTLATEISKDPQVRSHF
P.dactylifera_XP_008792596.1     -----------------------------------------GEAAAPVGAAVRVGKERVKEMLMATGGSAEVVGICGIGGSGKTTLAKEICRDPRIQNYF
A.comosus_Aco004926.1            MQGLEMGVVVGGGE---------------------------AVGPPMIGAGARVGNERVKEV-LMGGAGWGAVGICGIGGSGKTTLAREICRDPQIRHYF
M.acuminata_GSMUA_Achr7T08250_   -----------------------------------------GEGEKPVGVGIRVGKERVKEMLMAGGDRAAVVGISGIGGSGKTTLAKEICRDPQIRSYF
N.benthamiana_NRG1_AAY54606.1    TNGSGFSGWSDVP----------------------------QFSDSVVGFDLPLQELKVKLL----EEKEKVVVLSAPAGCGKTTLAAMLCQEDDIKDKY
A.thaliana_ADR1_BAF00531.1       -----------------------------------------TVSDPEIQTVLELGKKKVKEM-MFKFTDTHLFGISGMSGSGKTTLAIELSKDDDVRGLF
A.thaliana_RPW8.1_ACJ05907.1
A.thaliana_RPW8.2_ACJ72031.1
                                                                          NBS DOMAIN
```

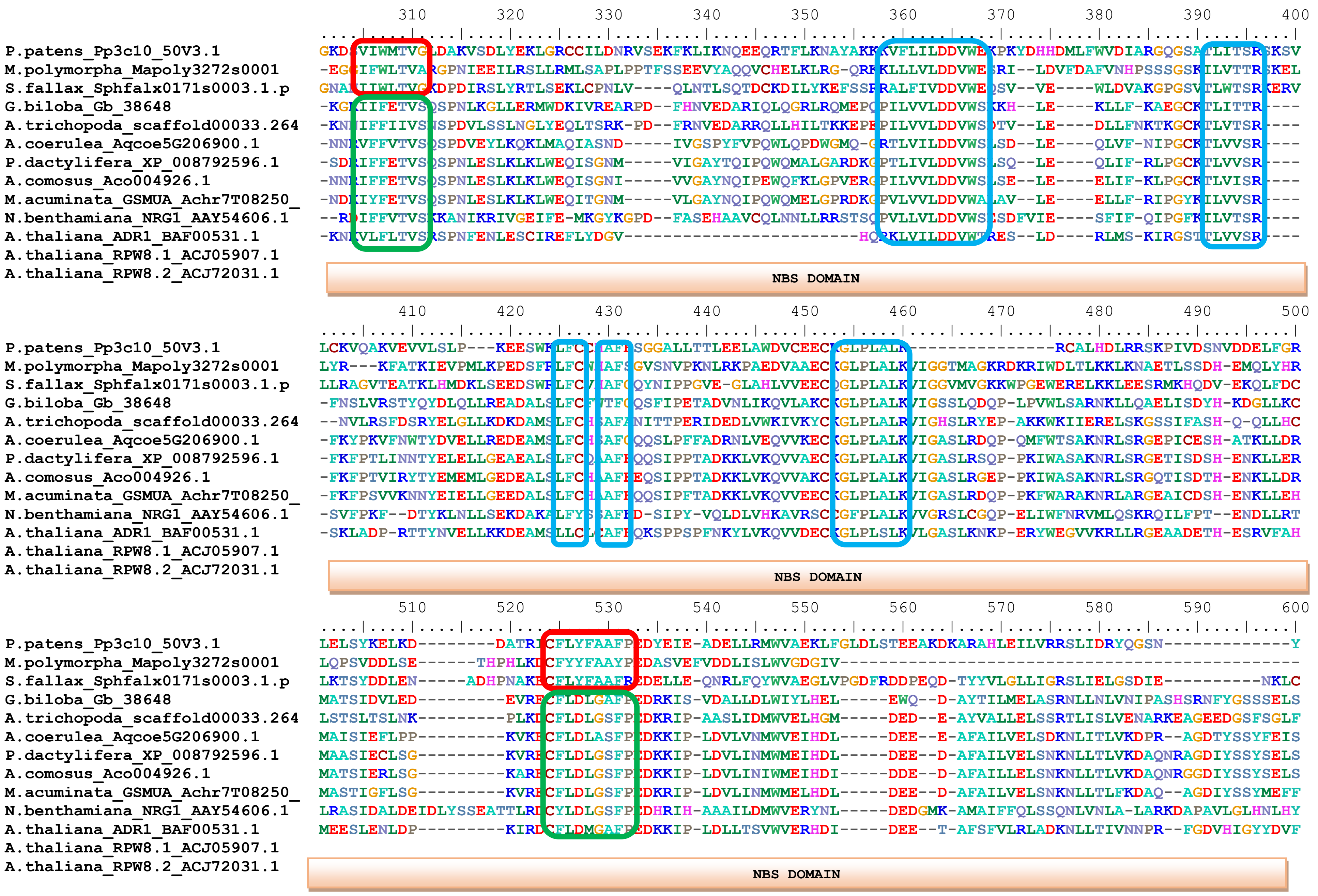

310 320 330 340 350 360 370 380 390 400
P.patens_Pp3c10_50V3.1
M.polymorpha_Mapoly3272s0001
S.fallax_Sphfalx0171s0003.1.p
G.biloba_Gb_38648
A.trichopoda_scaffold00033.264
A.coerulea_Aqcoe5G206900.1
P.dactylifera_XP_008792596.1
A.comosus_Aco004926.1
M.acuminata_GSMUA_Achr7T08250_
N.benthamiana_NRG1_AAY54606.1
A.thaliana_ADR1_BAF00531.1
A.thaliana_RPW8.1_ACJ05907.1
A.thaliana_RPW8.2_ACJ72031.1
NBS DOMAIN
410 420 430 440 450 460 470 480 490 500
P.patens_Pp3c10_50V3.1
M.polymorpha_Mapoly3272s0001
S.fallax_Sphfalx0171s0003.1.p
G.biloba_Gb_38648
A.trichopoda_scaffold00033.264
A.coerulea_Aqcoe5G206900.1
P.dactylifera_XP_008792596.1
A.comosus_Aco004926.1
M.acuminata_GSMUA_Achr7T08250_
N.benthamiana_NRG1_AAY54606.1
A.thaliana_ADR1_BAF00531.1
A.thaliana_RPW8.1_ACJ05907.1
A.thaliana_RPW8.2_ACJ72031.1
NBS DOMAIN
510 520 530 540 550 560 570 580 590 600
P.patens_Pp3c10_50V3.1
M.polymorpha_Mapoly3272s0001
S.fallax_Sphfalx0171s0003.1.p
G.biloba_Gb_38648
A.trichopoda_scaffold00033.264
A.coerulea_Aqcoe5G206900.1
P.dactylifera_XP_008792596.1
A.comosus_Aco004926.1
M.acuminata_GSMUA_Achr7T08250_
N.benthamiana_NRG1_AAY54606.1
A.thaliana_ADR1_BAF00531.1
A.thaliana_RPW8.1_ACJ05907.1
A.thaliana_RPW8.2_ACJ72031.1
NBS DOMAIN

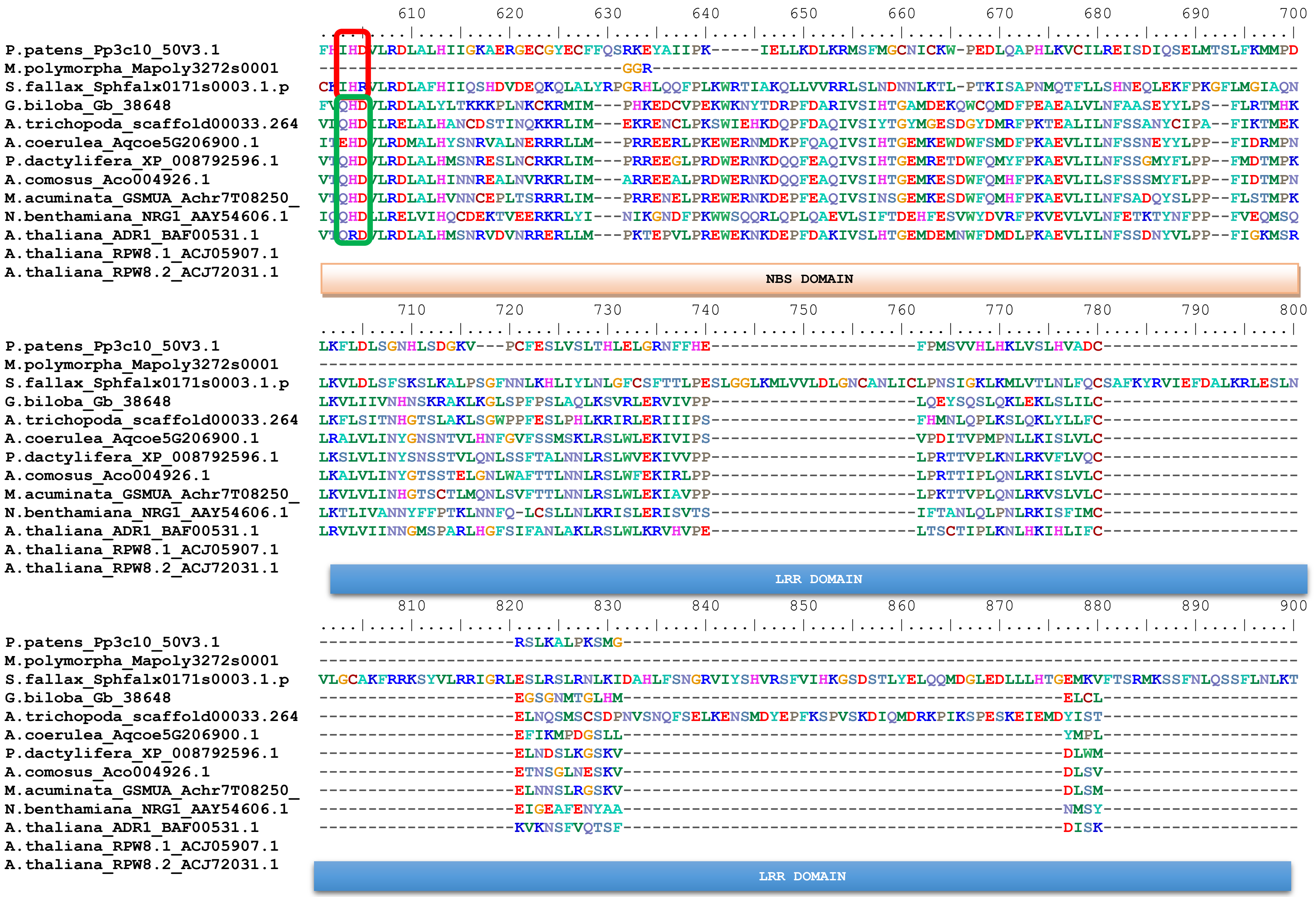
P.patens_Pp3c10_50V3.1
M.polymorpha_Mapoly3272s0001
S.fallax_Sphfalx0171s0003.1.p
G.biloba_Gb_38648
A.trichopoda_scaffold00033.264
A.coerulea_Aqcoe5G206900.1
P.dactylifera_XP_008792596.1
A.comosus_Aco004926.1
M.acuminata_GSMUA_Achr7T08250_
N.benthamiana_NRG1_AAY54606.1
A.thaliana_ADR1_BAF00531.1
A.thaliana_RPW8.1_ACJ05907.1
A.thaliana_RPW8.2_ACJ72031.1
NBS DOMAIN
LRR DOMAIN
LRR DOMAIN

```
                                         910       920       930       940       950       960       970       980       990      1000
                                 ....|....|....|....|....|....|....|....|....|....|....|....|....|....|....|....|....|....|....|....|
P.patens_Pp3c10_50V3.1           ----NLNSLVKLDLYGCRSLKALPESMGNLNSLVELDLGGCESLEAFP--KSMGKLNSLVKLNLGGCESLEALPESMGK--------------------
M.polymorpha_Mapoly3272s0001     ----------------------------------------------------------------------------------------------------
S.fallax_Sphfalx0171s0003.1.p    LRLSSLPNLQSLQLTDCPNLVNLTLNDC--PNLENGALTNCPNLVSLPTLDTLPRLHSL-ILRL----SIKKLPQSFTHRGAFPALNLFNLGQSKIVEFP
G.biloba_Gb_38648                ----NLPRLHEINFDHCSNLEELAASICNMTSLQRLSVRNCHDLYKLS--DDLGNLSSLQMLRLYACPSLEELPHSICK--------------------
A.trichopoda_scaffold00033.264   ----IFPKLKEIEMDYCGDLRDFPSWVCRLTHLKKFSITNCHDLCNLP--EEVGGLRYLEVLSLHACTRLSELPRSITR--------------------
A.coerulea_Aqcoe5G206900.1       ----ILPRLLELTMDHCTNLTELPSSVCSFSSLRSLSITNCHDLQNLP--IELGQLKSLQILRFCSCPALEKLPQSLCG--------------------
P.dactylifera_XP_008792596.1     ----TFPRLSSLTIDHCIDLNELPSSICEVSSLESISISNCHDLSELP--HEFGKLGSLQILRIYACPALKRLPQSICQ--------------------
A.comosus_Aco004926.1            ----TFPKLSDLTIDHCIDLKELPSSICEVRSLNSISISNCHDLYELP--SELGKLESLEILRLYACPSLKRLPQSICR--------------------
M.acuminata_GSMUA_Achr7T08250_   ----TLPRLSHLTIDHCIDLTKLPSSICNIGSLQCISISNCHDLSELP--GEFGKLSSLEILRVYACPSIKRLPQSICR--------------------
N.benthamiana_NRG1_AAY54606.1    ----MWPKLVEMNIEYCSDLVEVPAETCDLVGLKKLSICYCHELVALP--EELGKLSNLEVLRLHSCTNVSKLPESVVK--------------------
A.thaliana_ADR1_BAF00531.1       ----IFPSLSDLTIDHCDDLLEL-KSIFGITSLNSLSITNCPRILELP--KNLSNVQSLERLRLYACPELISLPVEVCE--------------------
A.thaliana_RPW8.1_ACJ05907.1
A.thaliana_RPW8.2_ACJ72031.1
                                 [                                LRR DOMAIN                                                       ]

                                        1010      1020      1030      1040      1050      1060      1070      1080      1090      1100
                                 ....|....|....|....|....|....|....|....|....|....|....|....|....|....|....|....|....|....|....|....|
P.patens_Pp3c10_50V3.1           ------LNSLVQLNLYGCGSLEALPESMGNLNSLVELNLGSCESL-------------------------------------------------------
M.polymorpha_Mapoly3272s0001     ----------------------------------------------------------------------------------------------------
S.fallax_Sphfalx0171s0003.1.p    EVEEDAMPKLQWLDFNDCISLHTLPASISLLTSIQTINLGSKNEKLMTSCQTNFSNSPIRKSFIMDGKPLIPEEEVFELVIPMQEGTTRVQGNDKRPFQK
G.biloba_Gb_38648                ------LRQLEFLDISLCECMKQLPEALGQLSSLREIDMRECSRV-------------------------------------------------------
A.trichopoda_scaffold00033.264   ------LSSLKLLDISECLNIVGLPNDLHGLSGLEKIDMSRCSQI-------------------------------------------------------
A.coerulea_Aqcoe5G206900.1       ------LENLKYLDISQCVNLTSLPERIGGLIMLEKIDMRECSQI-------------------------------------------------------
P.dactylifera_XP_008792596.1     ------LKRLKYLDISQSLNLRDLPEELGNLKNLEKIDMRECS-L-------------------------------------------------------
A.comosus_Aco004926.1            ------LRRLKYLDISQCINLGDLPEELGHLTSLEKIDMRECSQL-------------------------------------------------------
M.acuminata_GSMUA_Achr7T08250_   ------LKRLKYLDISQSFNLRELPEELGHLTSLEKIDMRECSQL-------------------------------------------------------
N.benthamiana_NRG1_AAY54606.1    ------LNRLGFLDVYDCVELDFLPREMDQLCSLRTICMGSRLGF-------------------------------------------------------
A.thaliana_ADR1_BAF00531.1       ------LPCLKYVDISQCVSLVSLPEKFGKLGSLEKIDMRECS-L-------------------------------------------------------
A.thaliana_RPW8.1_ACJ05907.1
A.thaliana_RPW8.2_ACJ72031.1
                                   [                                LRR DOMAIN                                                     ]

                                        1110      1120      1130      1140      1150      1160
                                 ....|....|....|....|....|....|....|....|....|....|....|....|...
P.patens_Pp3c10_50V3.1           ------EAFPESMGNLKNLKVFK----------------------------------------
M.polymorpha_Mapoly3272s0001     ---------------------------------------------------------------
S.fallax_Sphfalx0171s0003.1.p    VHGDDEERMFKRGGSIMGSDLFIPSSPTRFVFRGSSSLAEPTKSEKEHGLCEAL---------
G.biloba_Gb_38648                ------KQLPQSASDLRSLVHVICDEKIGHHWSAIKASTIPQLCVEVAEKHFHLDWLED----
A.trichopoda_scaffold00033.264   ------RRLPGSVAELRGLKNVICDEEIANQWEEIKLQSLSGLHVRVVEEEATLDWLER----
A.coerulea_Aqcoe5G206900.1       ------SSVPASAVSLRRLSRVICDEEASWLWKEVE-RVVPDLRVEVAEECFNLDWLAE----
P.dactylifera_XP_008792596.1     ------RNIPRSLSSLKSLGHVICDEEMALLWKEAE-RHIPDLRIQIAEQCYNLDWLVE----
A.comosus_Aco004926.1            ------RNLPRSSSSLKSLGHVVCDEEMALFWKEAE-RNIPDLRVQVAEVCYNLDWLVE----
M.acuminata_GSMUA_Achr7T08250_   ------RTIPRSSSSLKSLGHVICDEEVALLWKEAE-RCIPDLRVQVAEECFNLDWLVE----
N.benthamiana_NRG1_AAY54606.1    ------TELPDSVLRLVKLEDVVCDEETASLWEYYK-EHLRNLRITVIKEDINLNLLHKSLFI
A.thaliana_ADR1_BAF00531.1       ------LGLPSSVAALVSLRHVICDEETSSMWEMVK-KVVPELCIEVAKKCFTVDWLDD----
A.thaliana_RPW8.1_ACJ05907.1
A.thaliana_RPW8.2_ACJ72031.1
                                  [                                LRR DOMAIN                                                      ]
```

Supplementary Figure 3 : Progressive clustering of the RPW8 domain in seven conifer species. Y-axis shows the respective number of sequences of each clade and the X-axis represents sequence identity cut-off.

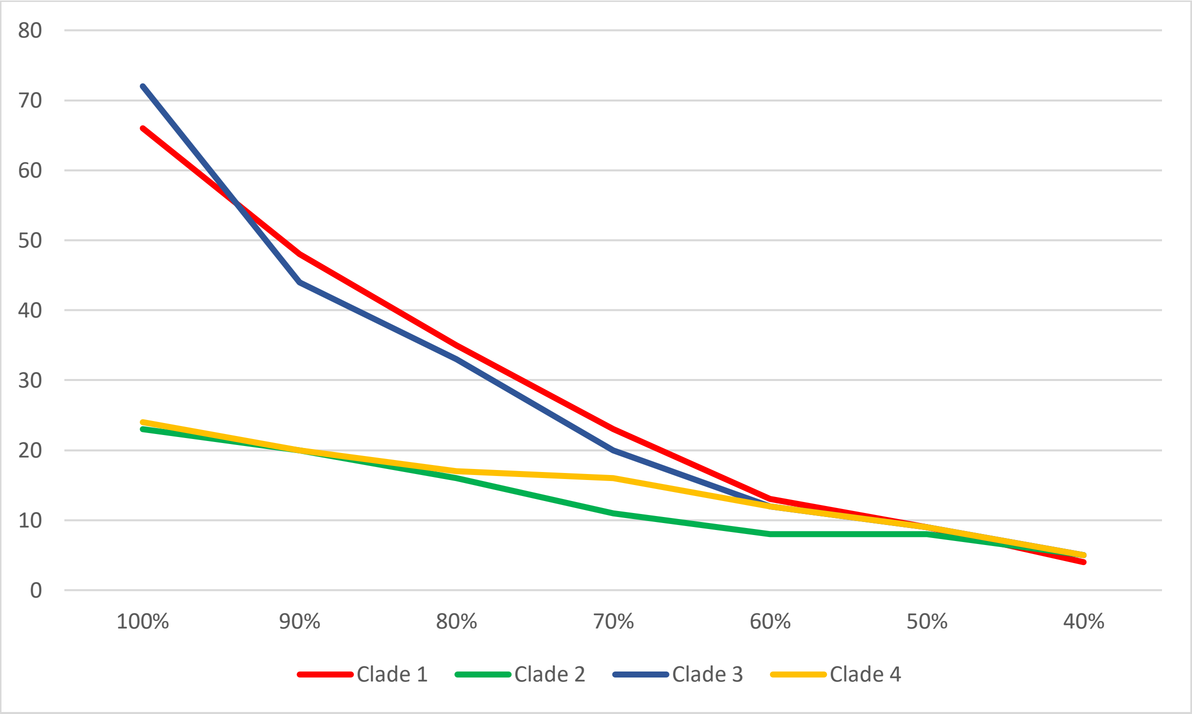

Supplementary Figure S4: Unrooted ML phylogenetic tree using the WAG model of the RPW8 domain in conifers after a 60% sequence identity cut-off

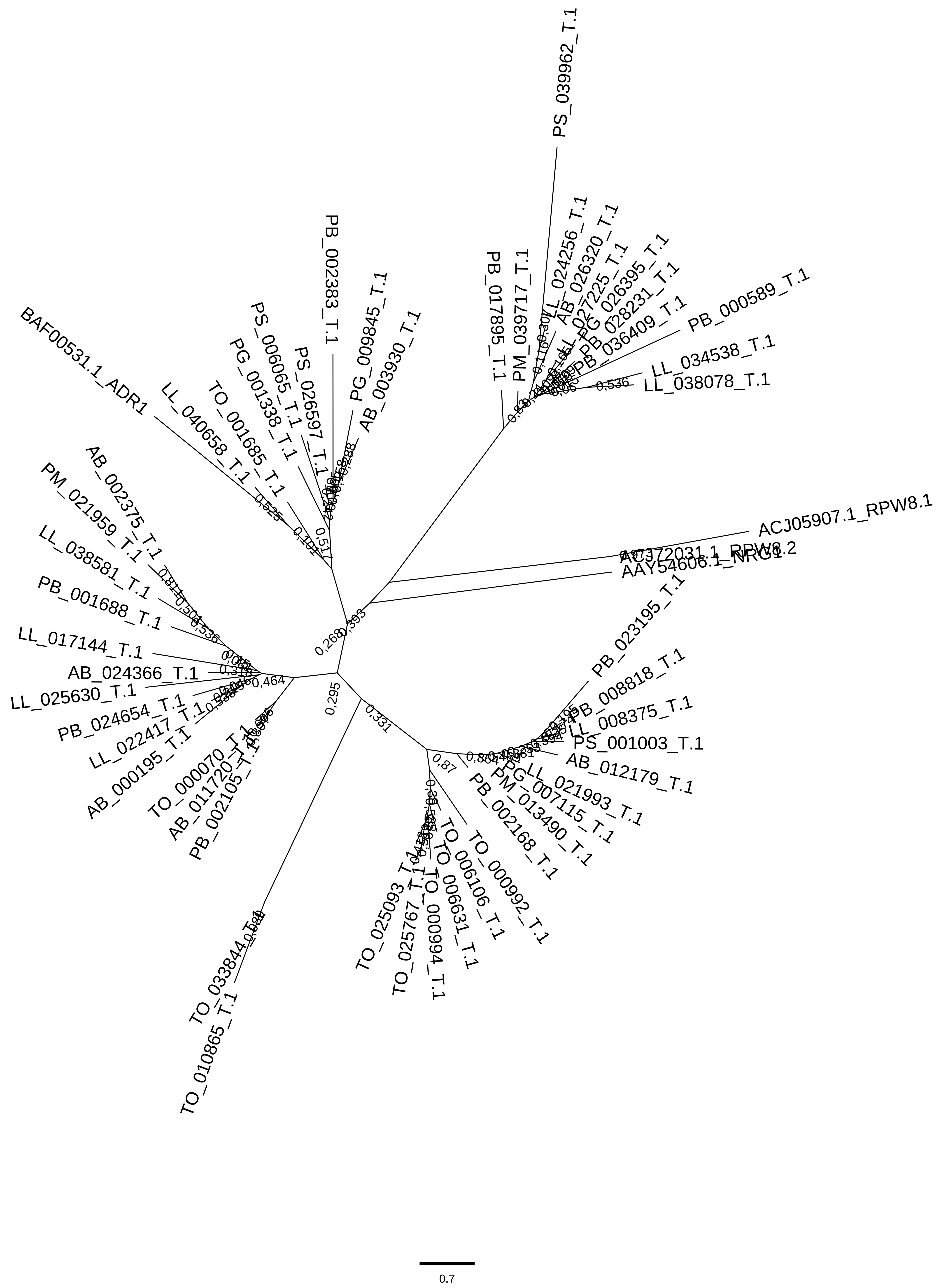

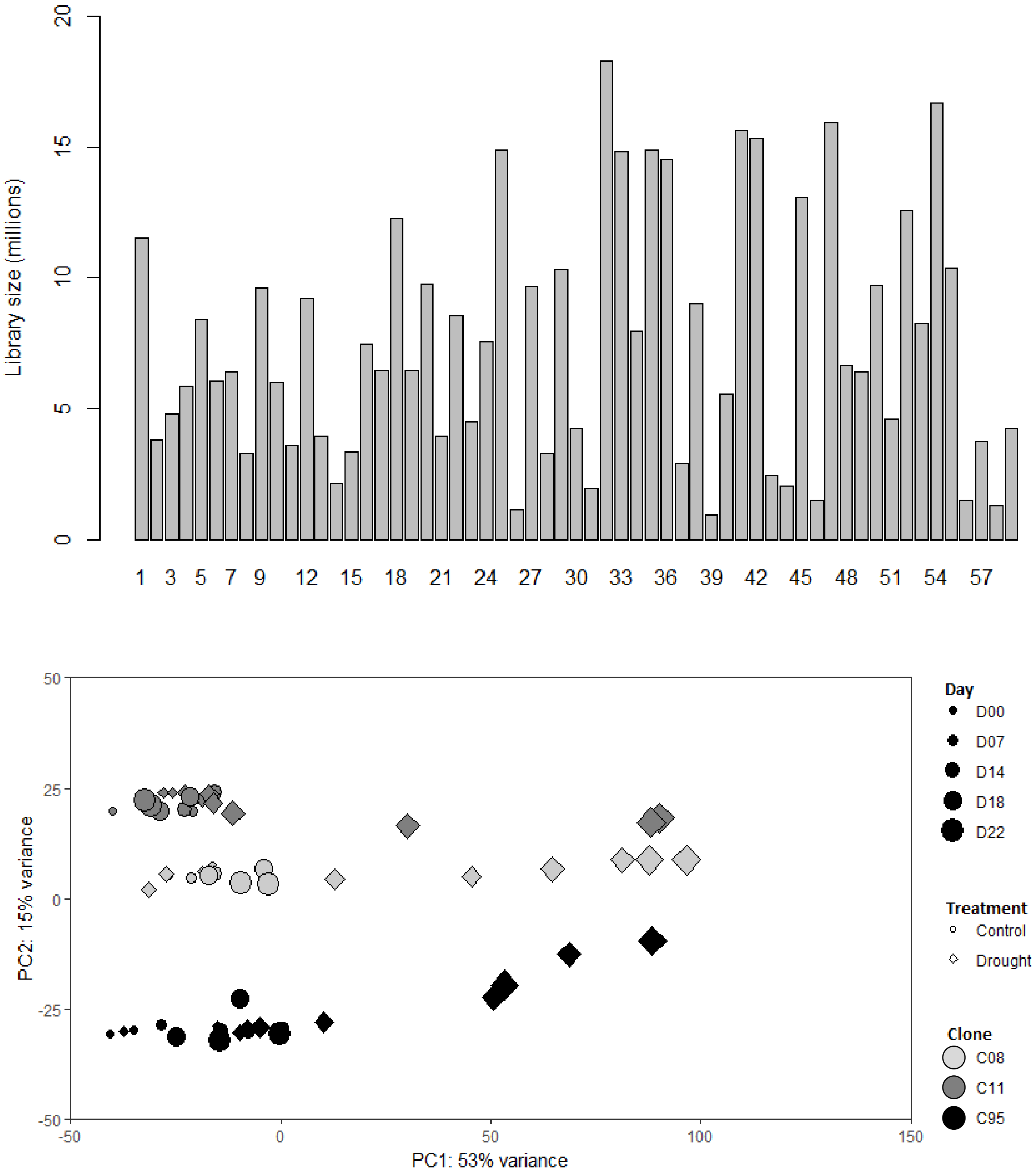

Supplemental Figure S5: Analysis of the RNA sequencing data from the study of Stival-Sena et al. (2018). Library size of the transcriptomes for the 59 samples (top). Principal component analysis based on the expression level of all genes in each library (below). The three *P. glauca* clones C08, C11 and C95 are depicted in light grey, dark grey and black, respectively. Control and drought stressed plants are shown in circle and diamond-shaped. The different time points are illustrated by increasing shape-size from 0-day post treatment (J64) with small-size shape to 22-days post treatment (J86) with large-size shape.

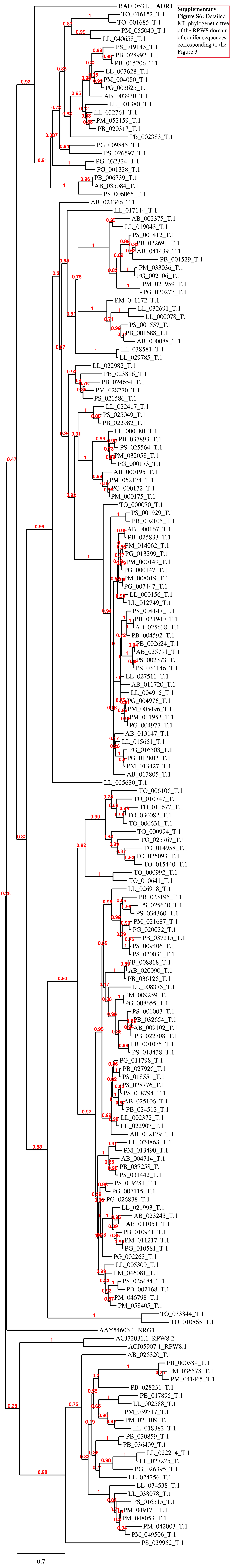

Supplementary Figure S6: Detailed ML phylogenetic tree of the RPW8 domain of conifer sequences corresponding to the Figure 3

Supplementary Figure S7: Detailed ML phylogenetic tree of the RPW8 domain of clustered land plant sequences corresponding to the Figure 4.

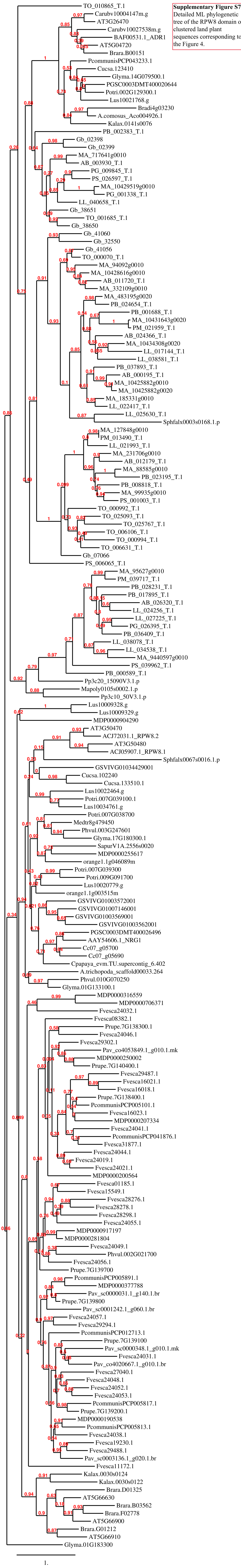

Supplementary Figure S8: unrooted ML phylogenetic tree of the RPW8 domain in land plants using the JTT substitution model

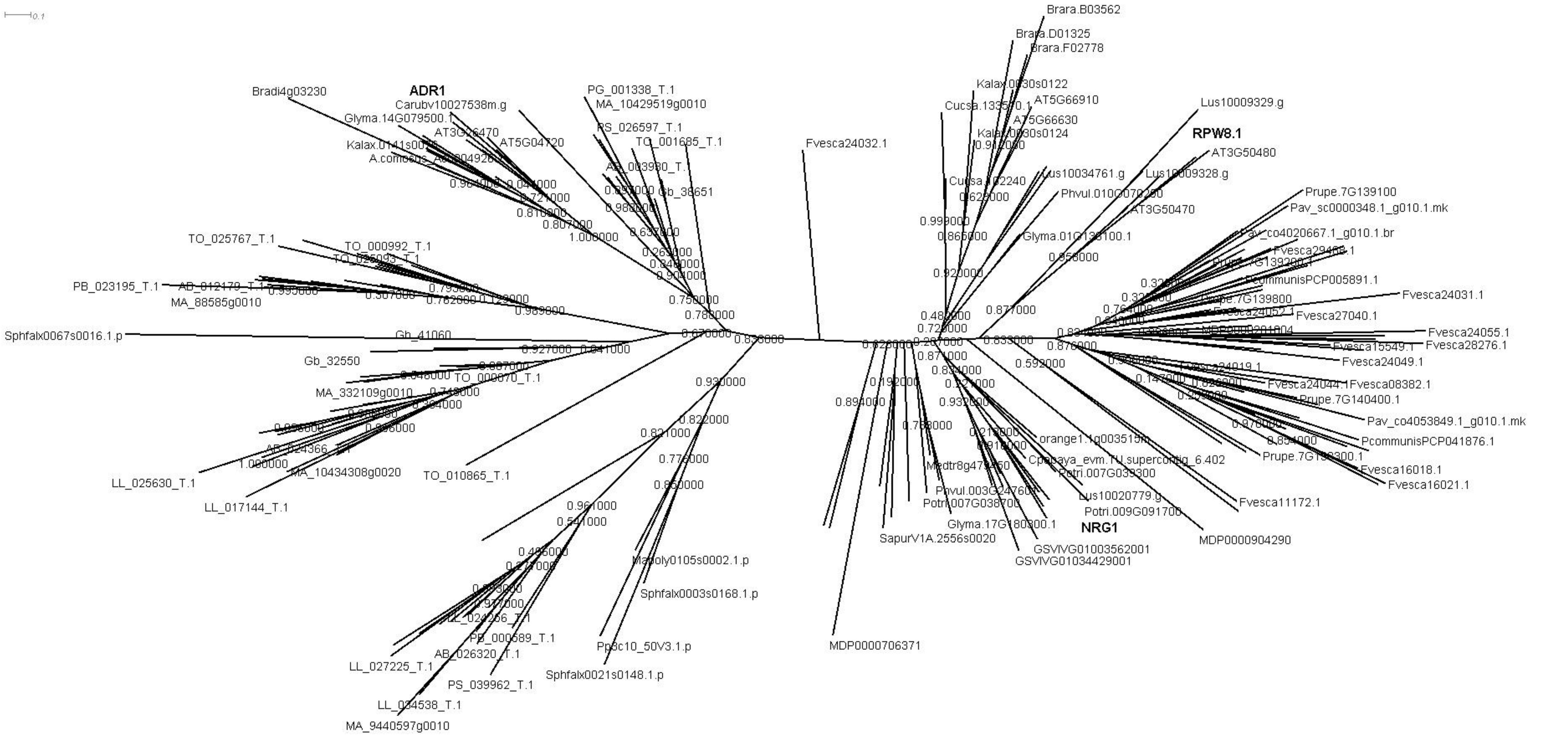

Supplementary Figure 9:

Count number average, obtained from six biological replicates (two replicates for the three genotypes C08, C11, C95; with SEM), of the five most differentially expressed RNL sequences (Adjusted P-value <0.05) over the five time points of sampling.

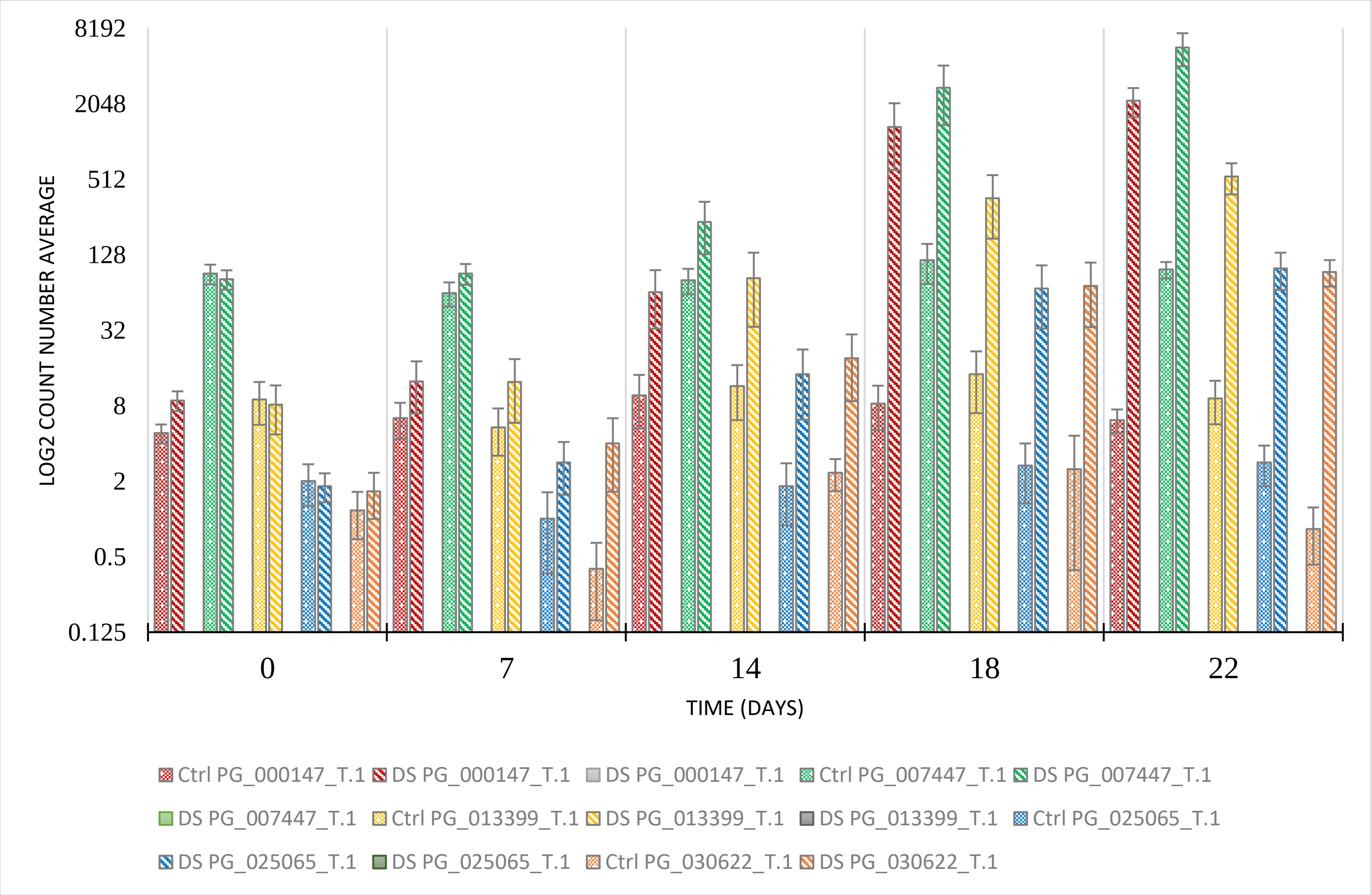

Supplementary Table S1 : Ratio of NLR-related sequences to the total number of transcripts in this study and in other plants.

Species	Coding genes	Gene transcripts	TOTAL NLR	NLR /coding genes ratio	NLR /gene transcripts ratio	Annotation Genebuild by EnsemblPlants	References
Abies balsamea	-	46178	338	-	0,73%		
Larix laricina	-	50712	633	-	1,25%		
Picea glauca	-	37491	506	-	1,35%		
Picea mariana	-	58751	725	-	1,23%		
Pinus banksiana	-	47473	486	-	1,02%		
Pinus strobus	-	45447	560	-	1,23%		
Thuja occidentalis	-	38767	486	-	1,25%		
Arabidopsis thaliana	27655	55442	205	0,74%	0,37%	TAIR10, INSDC Assembly GCA_000001735.1, Sep 2010	Meyers et al. 2002
Glycine max	56679	90023	319	0,56%	0,35%	Glycine_max_v2.0, INSDC Assembly GCA_000004515.3, Nov 2015	Kang et al. 2012
Medicago truncatula	50444	60059	571	1,13%	0,95%	MedtrA17_4.0, INSDC Assembly GCA_000219495.2, Jun 2014	Shao et al. 2014
Oryza sativa	40745	89723	458	1,12%	0,51%	ASM465v1, INSDC Assembly GCA_000004655.2, Jan 2005	Li et al. 2010
Populus trichocarpa	41377	46745	317	0,77%	0,68%	JGI2.0, INSDC Assembly GCA_000002775.2, Jan 2010	Kohler et al. 2008
Prunus persica	26873	47323	354	1,32%	0,75%	Prunus_persica_NCBIv2, INSDC Assembly GCA_000346465.2, Sep 2017	Zhong et al. 2015
Solanum Lycopersicum	33810	35216	326	0,96%	0,93%	SL2.50, INSDC Assembly GCA_000188115.2, Oct 2014	Andolfo et al. 2014
Vitis vinifera	29971	30609	459	1,53%	1,50%	IGGP_12x, INSDC Assembly GCA_000003745.2, Jun 2011	Yang et al. 2008
Zea mays	39498	138270	95	0,24%	0,07%	B73 RefGen_v4 (Zm-B73-REFERENCE-GRAMENE-4.0), INSDC Assembly GCA_000005005.6, Mar 2016	Li et al. 2010

Supplementary Table 2 : Details of the NLR subfamilies numbers in 49 land plant species. Numbers in blue are retrieved from the mentionned surveys and numbers in green are extracted from the related peptide dataset (using BlastP analysis and verified with HMM models).

**common name**	**name**	**NLR**	**TNL**	**CNL**	**RNL**	**NLR survey**	**Source for RNL search**	**Peptide dataset**
common liverwort	*Marchantia polymorpha*	39	0	7	2	Xue et al. 2012	Phytozome v12.1	Marchantia polymorpha v3.1
flat-topped bogmoss	*Sphagnum fallax*	252	38	NA	10	-	Phytozome v12.1	Sphagnum fallax v0.5
spreading earthmoss	*Physcomitrella patens*	115	9	11	4	Xue et al. 2012	Phytozome v12.1	Physcomitrella patens v3.3
spike moss	*Selaginella moellendorffii*	2	0	0	0	Yue et al. 2012	Phytozome v12.1	Selaginella moellendorffii v1.0
Gingko biloba	*Gingko biloba*	NA	150	NA	13	-	Guan et al. 2016 - Gb.pep.fa	http://gigadb.org/dataset/100209
Northern white-cedar	*Thuya occidentalis*	NA	317	85	31	-	Transcriptome	-
Eastern larch	*Larix laricina*	NA	343	166	46	-	Transcriptome	-
white spruce	*Picea glauca*	NA	279	134	31	-	Transcriptome	-
Black spruce	*Picea mariana*	NA	379	184	43	-	Transcriptome	-
White pine	*Pinus strobus*	NA	339	124	40	-	Transcriptome	-
Jack pine	*Pinus banksiana*	NA	250	119	55	-	Transcriptome	-
balsam Fir	*Abies balsamea*	NA	150	112	32	-	Transcriptome	-
Amborella	*Amborella trichopoda*	NA	11	NA	1	-	Phytozome v12.1	Amborella trichopoda v1.0
Date palm	*Phoenix dactylifera*	NA	0	NA	1	-	NCBI genomes assembly DPV01	Phoenix dactylifera DPV01
Banana	*Musa acuminata*	NA	0	NA	1	-	Phytozome v12.1	*Musa acuminata v1*
Pineapple	*Ananas comosus*	NA	0	NA	1	-	Phytozome v12.1	*Ananas comosus v3*
Maize	*Zea mays*	95	0	71	1	Li et al. 2010	Phytozome v12.1	Zea mays Ensembl-18
Sorghum	*Sorghum bicolor*	184	0	130	1	Li et al. 2010	Phytozome v12.1	Sorghum bicolor v3.1.1
Brachypodium	*Brachypodium distachyon*	212	0	205	1	Li et al. 2010	Phytozome v12.1	Brachypodium distachyon v3.1
Rice	*Oryza sativa*	458	0	274	1	Li et al. 2010	Phytozome v12.1	Oryza sativa v7_JGI
Aquilegia coerulea	*Aquilegia coerulea*	NA	0	NA	1	-	Phytozome v12.1	*Aquilegia coerulea v3.1*
Milky Widow's Thrill	*Kalanchoe laxiflora*	NA	80	NA	6	-	Phytozome v12.1	*Kalanchoe laxiflora v1.1*
Prince-of-Wales feather	*Amaranthus hypochondriacus*	NA	1	NA	1	-	Phytozome v12.1	*Amaranthus hypochondriacus v1.0*
robusta coffee	*coffea canephora*	NA	14	NA	3	-	sol genomics network	Coffea canephora protein sequences v1.0
Nicotiana benthamiana	*Nicotiana benthamiana*	NA	14	NA	5	-	sol genomics network	N. benthamiana Genome v1.0.1 predicted proteins
Potato	*Solanum tuberosum*	438	77	361	5	Jupe et al.2012	sol genomics network	Potato PGSC DM v3.4 protein sequences
currant tomato	*Solanum pimpinellifolium*	355	45	300	3	Andolfo et al. 2014	sol genomics network	S. pimpinellifolium protein sequences LA1589
Tomato	*Solanum lycopersicum*	326	40	280	3	Andolfo et al. 2014	sol genomics network	Tomato Genome proteins (ITAG release 3.20)
Pepper	*Capsicum annuum*	306	16	288	2	Qian et al. 2017	-	Cpsicum annuum cv CM334 Genome protein sequences (release 1.55)
Wine grape	*Vitis vinifera*	535	111	215	16	Yang et al. 2008	Phytozome v12.1	Vitis vinifera Genoscope.12X

Barrelclover	*Medicago truncatula*	571	275	279	17	Shao et al. 2014	-	Medicago truncatula Mt4.0v1
common bean	*Phaseolus vulgaris*	337	103	224	10	Shao et al. 2014	-	-
soybean	*Glycine max*	465	178	269	18	Shao et al. 2014	-	Glycine max Wm82.a2.v1
pigeon pea	*Cajanus cajan*	289	112	167	10	Shao et al. 2014	-	-
strawberry	*Fragaria vesca*	NA	153	193	52	Jia et al. 2015	Genome Database for Rosaceae	Stawberry Genome v1.0 ab initio gene proteins
sweet cherry	*Prunus avium*	NA	156	NA	14	-	Genome Database for Rosaceae	Sweet cherry genome v1.0 proteins
Peach	*Prunus persica*	443	194	249	19	Van Ghelder et al. 2016; Jia et al.2015	Genome Database for Rosaceae	Peach Genome v2.0.a1 all transcript peptide
pear	*Pyrus bretschneideri Rehd*	617	342	275	42	Jia et al. 2015	Genome Database for Rosaceae	Pear draft genome v1.0 hybrid proteins
apple	*Malus domestica*	1303	664	639	49	Jia et al. 2015	Genome Database for Rosaceae	Apple Genome V1.0 predicted peptides
Flax	*Linum usitatissimum*	147	98	49	10	Kale et al. 2013	Phytozome v12.1	*Linum usitatissimum v1.0*
Purple willow	*Salix purpurea*	NA	164	NA	9	-	Phytozome v12.1	*Salix purpurea v1.0*
Poplar	*Populus trichocarpa*	402	123	277	9	Kohler et al. 2008	Phytozome v12.1	Populus trichocarpa v3.0
Cucumber	*Cucumis sativus*	53	11	17	4	Wan et al. 2013	Phytozome v12.1	Cucumis sativus v1.0
Mustard	*Brassica rapa*	80	52	28	11	Mun et al. 2009	Phytozome v12.1	Brassica rapa FPsc v1.3
Pink shepherd's-purse	*Capsella rubella*	NA	91	NA	8	-	Phytozome v12.1	Capsella rubella v1.0
Lyre-leaved rock-cress	*Arabidopsis lyrata*	138	103	21	11	Guo et al. 2011	Phytozome v12.1	Arabidopsis lyrata v2.1
Thale cress	*Arabidopsis thaliana*	151	94	55	11	Meyers et al. 2003	TAIR	TAIR10 proteins
Papaya	*carica papaya*	54	6	6	3	Porter et al. 2009	Phytozome v12.1	Carica papaya ASGPBv0.4
sweet orange	*Citrus sinensis*	NA	152	NA	11	-	Phytozome v12.1	Citrus sinensis v1.1

Supplementary Table S3: Details of the RPW8 motifs identified in figure 3.

motif	MEME	Nb of sites	e-values	Width
a	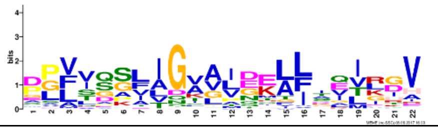	84	2.8e-332	22
b	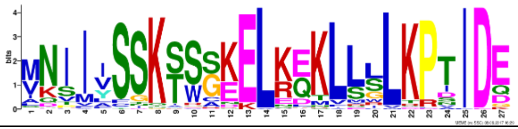	71	5.0e-1038	27
c	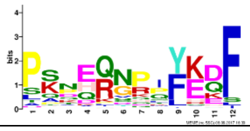	132	5.4e-418	12
d	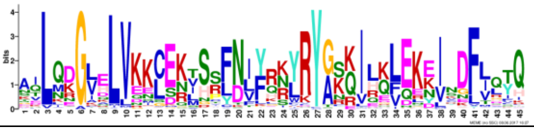	157	1.2e-3475	45
d*	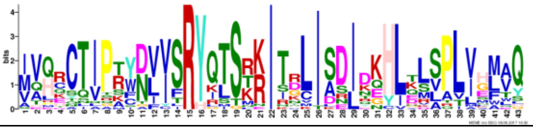	24	1.5e-402	43
e	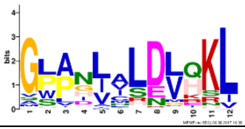	82	1.1e-257	12
f	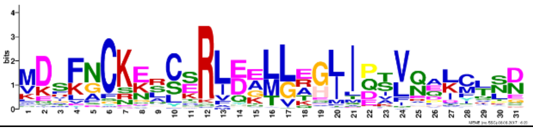	122	7.5e-1295	31
g	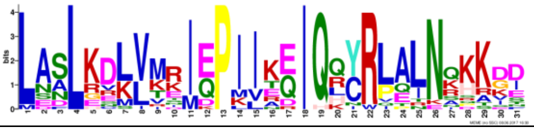	19	1.1e-178	31
h	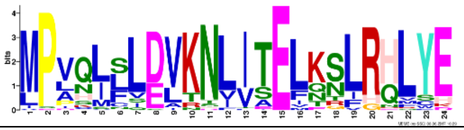	49	4.2e-598	24
i	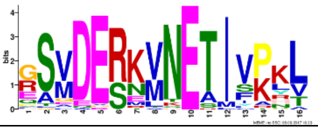	48	2.2e-347	16

Supplementary Table S4: List of the 119 differentially expressed NLR genes (Adjusted Pvalue <0.05) with their subfamily and expression group assignations.

Differentially Expressed NLR	baseMean	log2FoldChange	lfcSE	stat	Pvalue	Adjusted Pvalue	NBS-LRR Subfamily	Expression group
PG_001471_T.1	13.66855083	2.450547425	0.865418942	18.94433341	0.000805985	0.002115279	CNL	1
PG_011798_T.1	1154.483568	0.962059964	0.168405879	53.39583691	7.04E-11	9.52E-10	RNL	1
PG_018623_T.1	68.07760019	0.589946431	0.317997279	13.13450767	0.010636928	0.020782289	TNL	1
PG_020032_T.1	119.6129448	3.158357801	0.574544738	53.9227415	5.46E-11	7.60E-10	RNL	1
PG_021812_T.1	196.7851559	2.257576457	0.311751807	80.21065113	1.57E-16	7.77E-15	TNL	1
PG_026838_T.1	62.81941299	0.010673059	0.847345107	12.44157594	0.014352604	0.026941669	RNL	1
PG_000147_T.1	454.6327066	8.617440953	0.820836302	166.7146012	5.30E-35	2.60E-31	RNL	2
PG_007447_T.1	1203.423106	7.066495126	0.688865522	135.8840669	2.15E-28	2.11E-25	RNL	2
PG_013399_T.1	157.8220042	7.364781125	0.733023068	136.6303383	1.49E-28	1.54E-25	RNL	2
PG_019724_T.1	44.00186757	7.965987357	1.11145701	75.09382209	1.90E-15	7.41E-14	U	2
PG_025065_T.1	34.07984528	6.466260061	1.059575018	49.7327906	4.11E-10	4.61E-09	RNL	2
PG_030622_T.1	26.17277113	7.590556448	1.21711405	53.82779567	5.72E-11	7.92E-10	RNL	2
PG_001975_T.1	126.5367603	-4.642758124	0.651369846	62.22673798	9.87E-13	2.10E-11	CNL2	3
PG_003813_T.1	303.1528528	-1.160856559	0.503404947	23.71939465	9.09E-05	0.000301984	TNL	3
PG_016168_T.1	69.93504192	-3.78489389	0.651036855	41.65805645	1.96E-08	1.52E-07	CNL	3
PG_030758_T.1	65.90984909	-4.537298352	0.707877044	54.53546445	4.07E-11	5.90E-10	U	3
PG_030759_T.1	66.36499118	-4.47384608	0.696530501	51.72759587	1.57E-10	1.95E-09	CNL2	3
PG_032601_T.1	23.12206662	-4.201030728	0.761142056	34.47644297	5.95E-07	3.24E-06	CNL	3
PG_023129_T.1	104.6630694	-2.531400929	0.415916275	52.86661926	9.09E-11	1.20E-09	U	4
PG_023520_T.1	42.27907251	-2.144389575	0.595158543	24.60194623	6.05E-05	0.000209127	TNL	4
PG_023535_T.1	94.93489561	-2.538803176	0.479327493	35.70120308	3.33E-07	1.92E-06	CNL2	4
PG_023965_T.1	21.24298972	-1.816117862	0.531362906	13.5470234	0.008890343	0.017761685	TNL	4
PG_025567_T.1	60.22093264	-3.019162588	0.510433424	55.44525513	2.62E-11	3.98E-10	CNL	4
PG_026514_T.1	55.30180247	-1.303832876	0.331295901	17.14696092	0.0018099	0.004364347	TNL	4
PG_027609_T.1	42.23665206	-2.194485926	0.490044659	28.78175933	8.66E-06	3.62E-05	CNL2	4
PG_030965_T.1	19.98854145	-1.602304019	0.4825323	14.01007491	0.00726297	0.0149126	TNL	4
PG_032035_T.1	36.32098043	-2.543761889	0.479207083	49.29935164	5.06E-10	5.58E-09	CNL	4
PG_000915_T.1	63.38803716	-2.759108663	0.679969234	22.29136321	0.000175353	0.000544333	TNL	5
PG_018964_T.1	26.76858277	-2.628668648	0.576219602	31.52195183	2.40E-06	1.14E-05	TNL	5
PG_030046_T.1	100.9200444	-1.730344318	0.675588051	10.79058881	0.029021106	0.049747933	TNL	5
PG_031163_T.1	49.7212265	-2.686052791	0.76808363	14.24888412	0.006541656	0.013592214	TNL	5
PG_022851_T.1	112.1294323	-3.868486953	0.637975857	37.77128262	1.25E-07	8.00E-07	CNL2	6
PG_025525_T.1	108.4130107	-4.069264073	0.789541005	26.84974793	2.13E-05	8.18E-05	CNL2	6
PG_036715_T.1	20.5698958	-4.28233979	0.753577594	35.47274244	3.71E-07	2.12E-06	CNL2	6
PG_002086_T.1	64.85685646	-4.147860455	0.70437581	42.80282853	1.14E-08	9.27E-08	CNL	7
PG_005099_T.1	63.33578162	-4.554452585	0.802123073	35.21336138	4.20E-07	2.36E-06	CNL	7
PG_005233_T.1	53.39038078	-4.064269404	0.727301141	34.38237824	6.22E-07	3.37E-06	CNL	7
PG_013121_T.1	96.62762652	-4.471619134	0.808072645	34.49306931	5.90E-07	3.21E-06	CNL	7
PG_033031_T.1	25.55387424	-3.91731213	0.856219097	22.5020579	0.000159189	0.000499048	CNL	7
PG_001161_T.1	54.10061107	-2.130084277	0.577936715	17.5661263	0.00149982	0.003689131	TNL	8
PG_002418_T.1	252.8121285	-2.618591326	0.458933465	48.21657648	8.51E-10	8.96E-09	TNL	8
PG_013663_T.1	38.19068774	-3.456289223	0.527170406	60.43620572	2.35E-12	4.55E-11	CNL	8
PG_020429_T.1	101.0200953	-4.94932683	0.683020599	80.05027739	1.70E-16	8.32E-15	CNL	8
PG_022220_T.1	94.96966871	-5.050136833	0.800594648	60.90102294	1.88E-12	3.73E-11	CNL	8

PG_022823_T.1	44.71730791	-3.568524874	0.635262345	34.12287305	7.03E-07	3.75E-06	CNL2	8
PG_023416_T.1	89.60857445	-2.006389623	0.587944315	21.48811531	0.000253357	0.000755875	TNL	8
PG_024393_T.1	81.6559838	-4.331841513	0.647269824	62.05616348	1.07E-12	2.25E-11	U	8
PG_027550_T.1	32.04023514	-2.975991853	0.63282373	31.05784588	2.98E-06	1.38E-05	CNL	8
PG_030072_T.1	57.63111363	-4.180792975	0.613595477	69.6134584	2.74E-14	8.59E-13	CNL2	8
PG_032528_T.1	132.5588063	-1.175167371	0.436738839	23.0530549	0.000123573	0.000397487	TNL	8
PG_024369_T.1	10.34221701	-1.726291057	0.707336991	11.8502798	0.018500054	0.033692787	TNL	9
PG_030268_T.1	27.16756693	-2.580465791	0.785817968	18.45491226	0.00100539	0.002578626	TNL	9
PG_010062_T.1	18.82308515	-2.435621672	0.686564156	30.18465372	4.49E-06	2.00E-05	CNL	10
PG_019615_T.1	32.96847495	-1.686565957	0.485751922	24.66200819	5.88E-05	0.000204041	CNL2	10
PG_021165_T.1	62.44948818	-0.919657288	0.575615773	13.00444501	0.011254095	0.02182623	TNL	10
PG_024997_T.1	40.69575945	-2.124083976	0.440362373	32.60236133	1.44E-06	7.14E-06	CNL	10
PG_027305_T.1	17.06346923	-3.314147182	0.695703658	25.05561518	4.90E-05	0.000172961	CNL	10
PG_027423_T.1	31.24823132	-4.447745951	0.657873666	64.51863955	3.25E-13	7.95E-12	TNL	10
PG_028579_T.1	40.29778808	-3.822941478	0.661760161	32.70628245	1.37E-06	6.84E-06	CNL2	10
PG_029570_T.1	122.4037371	-1.612713909	0.537969686	20.05200386	0.000487731	0.001357722	TNL	10
PG_031236_T.1	12.75522078	-2.21678313	0.64747364	23.24363588	0.000113196	0.000366752	TNL	10
PG_032108_T.1	17.46866298	-1.865769178	0.563457152	24.95263731	5.14E-05	0.00018066	TNL	10
PG_001027_T.1	161.5715712	-1.031062234	0.363706762	11.44447221	0.021997747	0.039168958	TNL	11
PG_002977_T.1	65.00590861	-0.67777785	0.252211026	18.10512926	0.001177058	0.002966979	CNL	11
PG_003080_T.1	41.14477415	-1.954068217	0.349801845	51.61383177	1.66E-10	2.05E-09	CNL	11
PG_005613_T.1	41.72700027	-1.880404521	0.412871922	36.7457954	2.03E-07	1.24E-06	CNL	11
PG_009545_T.1	34.78267968	-1.481033871	0.391503444	17.58808829	0.001485105	0.003657062	TNL	11
PG_011665_T.1	34.68241541	-1.253654738	0.346787633	25.15549246	4.68E-05	0.000166068	TNL	11
PG_013569_T.1	47.66285744	-0.916186698	0.371909318	11.57333464	0.020822788	0.037337609	TNL	11
PG_014034_T.1	90.69627304	-1.591659617	0.387987418	26.61009712	2.38E-05	9.03E-05	TNL	11
PG_014262_T.1	38.177421	-1.36031812	0.377384386	15.14860842	0.004402713	0.009602228	CNL	11
PG_024247_T.1	51.1213908	-0.959916607	0.40026069	10.9908364	0.026667194	0.046188784	TNL	11
PG_026136_T.1	47.89023568	-1.497486026	0.475179719	10.98336543	0.026751602	0.046318635	TNL	11
PG_026526_T.1	31.32844764	-1.614285564	0.386154877	21.57084417	0.000243947	0.000730799	CNL	11
PG_028266_T.1	84.82777644	-1.541650801	0.418689027	18.31051559	0.001073033	0.00272965	TNL	11
PG_028823_T.1	21.27612319	-2.630351034	0.483195303	47.36376561	1.28E-09	1.29E-08	CNL	11
PG_029130_T.1	35.03753941	-1.635264917	0.493262809	14.29919818	0.006398892	0.013352111	CNL	11
PG_030244_T.1	42.72735024	-1.777911641	0.380729803	23.97659572	8.07E-05	0.000271041	U	11
PG_031683_T.1	96.35560312	-1.776782864	0.384168178	25.52315451	3.95E-05	0.000142998	TNL	11
PG_003292_T.1	94.89065323	0.565879191	0.351994239	15.04870974	0.004601249	0.009977589	TNL	12
PG_010112_T.1	31.04581951	1.484548949	0.49360684	14.73020848	0.005294772	0.011290643	TNL	12
PG_026810_T.1	20.95937916	-1.934545381	0.592122454	13.52907765	0.008960119	0.017879254	TNL	12
PG_033929_T.1	30.20519774	1.346815978	0.471286512	15.82813764	0.003258664	0.00735466	TNL	12
PG_000173_T.1	60.68427734	-1.329337119	0.349102487	21.63603528	0.000236777	0.000712089	RNL	13
PG_000389_T.1	75.69006458	-2.715578045	0.465288902	38.56151786	8.58E-08	5.73E-07	CNL	13
PG_003633_T.1	58.58000727	-2.074512023	0.648566474	12.54688614	0.013715711	0.025914642	CNL	13
PG_003976_T.1	31.05365071	-1.602981113	0.432981079	18.93394418	0.000809781	0.002123821	TNL	13
PG_006607_T.1	43.07682985	-2.652887188	0.556816468	22.11757739	0.000189903	0.000584417	U	13
PG_007137_T.1	24.19273955	-4.415076688	0.653452545	56.79017683	1.37E-11	2.23E-10	CNL	13
PG_010581_T.1	1247.26345	-2.347340021	0.278682408	85.22064331	1.36E-17	8.62E-16	RNL	13
PG_019409_T.1	39.61601954	-1.966184761	0.52100778	19.3782101	0.000662242	0.001778693	TNL	13
PG_022962_T.1	43.29620255	-2.884348498	0.550496513	29.90588836	5.12E-06	2.25E-05	CNL	13

PG_027346_T.1	114.4947097	-2.682939906	0.602321885	21.14510885	0.000296365	0.00086847	TNL	13
PG_028426_T.1	27.22517091	-1.661573711	0.496468878	21.96234787	0.000203909	0.000622831	TNL	13
PG_029059_T.1	37.14968146	-1.409700204	0.372162057	26.02668853	3.13E-05	0.000115636	CNL	13
PG_029408_T.1	35.7229738	-4.078132414	0.753797523	30.7849868	3.39E-06	1.56E-05	CNL	13
PG_029409_T.1	83.51748434	-2.443438316	0.767259599	14.99019329	0.0047216	0.01019689	TNL	13
PG_030995_T.1	14.84798199	-3.366374962	0.79233794	19.78984998	0.000549431	0.001508092	CNL	13
PG_032342_T.1	38.56739212	-3.036606397	0.567025025	32.68832488	1.38E-06	6.90E-06	U	13
PG_002263_T.1	89.78943946	-2.329554751	0.762927717	11.83514554	0.018620208	0.033880185	RNL	14
PG_002809_T.1	41.19800658	-1.83551034	0.516296252	17.30213343	0.001688359	0.004100987	CNL	14
PG_010841_T.1	42.71141734	-1.034107428	0.362064416	11.40745909	0.022346948	0.039707905	U	14
PG_011683_T.1	54.07302573	-1.104980905	0.33933562	17.17473289	0.001787531	0.004319425	CNL	14
PG_012075_T.1	171.9369843	-1.344107138	0.239120268	54.36591039	4.41E-11	6.32E-10	TNL	14
PG_012802_T.1	376.1679886	-0.808815432	0.247471407	14.39266465	0.00614175	0.012870271	RNL	14
PG_013996_T.1	182.7643113	-1.195285507	0.378602913	23.12230687	0.000119697	0.000386223	TNL	14
PG_016503_T.1	90.50226088	-1.015731267	0.283381251	15.46284356	0.00383152	0.00848754	RNL	14
PG_019732_T.1	72.16617877	-1.559483228	0.44358853	15.38932295	0.003958254	0.008741645	TNL	14
PG_019785_T.1	44.98527101	-0.249587365	0.35077238	12.65842411	0.013071199	0.024864409	TNL	14
PG_020200_T.1	159.7914378	-1.168589846	0.319886811	14.88062609	0.004955319	0.010655918	TNL	14
PG_020291_T.1	62.67867129	-1.637604927	0.410999878	22.78754465	0.000139621	0.000442724	CNL	14
PG_021434_T.1	38.13073435	-1.793628192	0.455378934	18.49638759	0.000986752	0.002536124	TNL	14
PG_023840_T.1	57.64926664	-1.563447034	0.440550379	14.2394534	0.00656876	0.013638781	TNL	14
PG_024538_T.1	78.90344311	-1.652324331	0.415549383	18.88019809	0.000829701	0.002171136	TNL	14
PG_026017_T.1	27.25549826	-2.489154522	0.421522743	39.50007944	5.49E-08	3.83E-07	CNL	14
PG_026413_T.1	50.51917933	-0.668992338	0.308376356	13.71326107	0.008268744	0.016703407	TNL	14
PG_027685_T.1	38.47900144	-2.347668898	0.613558749	19.98185702	0.000503535	0.001397157	TNL	14
PG_028025_T.1	30.89383893	-1.427341915	0.40181666	21.70792093	0.000229112	0.000691316	U	14
PG_035021_T.1	17.37134006	-1.74716938	0.811981421	14.42315996	0.006060066	0.012712668	TNL	14

Supplementary Table S5: List of RNL sequences with, when available, their corresponding *P. glauca* gene catalogue accession number and genetic position (Rigault et al. 2011, Pavy et al. 2013).

RNL (reference transcriptome accession number)	RNL sequence group assignation	DEG	Expression group	Cluseq accession (Rigault et al. 2011)		Composite map linkage group (Pavy et al. 2018)	Composite map position (Pavy et al. 2018)
PG_001338_T.1	2	-	-	GQ0168_L19	GQ02747_D08	LG12	2,09
PG_004977_T.1	3	-	-	GQ02905_L23	GQ03418_F13	LG12	43,32
PG_004976_T.1	3	-	-	GQ02905_L23	-	LG12	43,32
PG_020277_T.1	3	-	-	WS00725_G10	GQ03715_H16	LG12	46,54
PG_016503_T.1	3	downregulated	14	GQ04109_A14	-	LG08	129,52
PG_010581_T.1	1	downregulated	13	GQ03702_K04	GQ03321_G12	LG06	3,86
PG_012538_T.1	-	-	-	GQ03612_J11	GQ0133_H03	LG06	4,13
PG_008655_T.1	1	-	-	GQ03222_P15	-	LG06	4,16
PG_011798_T.1	1	upregulated	1	GQ03512_J05	-	LG06	4,18
PG_003625_T.1	2	-	-	GQ02813_L10	-	LG02	75
PG_002263_T.1	1	downregulated	14	-	-	-	-
PG_007115_T.1	1	-	-	-	-	-	-
PG_020032_T.1	1	upregulated	1	WS0011_L03	-	-	-
PG_026838_T.1	1	upregulated	1	-	-	-	-
PG_009845_T.1	2	-	-	GQ03308_B18	WS00747_F12	-	-
PG_032324_T.1	2	-	-	-	-	-	-
PG_000147_T.1	3	upregulated	2	GQ0025_J06	GQ03619_N18	-	-
PG_000172_T.1	3	-	-	-	-	-	-
PG_000173_T.1	3	downregulated	13	GQ0031_B13	GQ03316_N22	-	-
PG_002106_T.1	3	-	-	GQ0205_A13	-	-	-
PG_007447_T.1	3	upregulated	2	-	-	-	-
PG_012802_T.1	3	downregulated	14	GQ03701_F14	-	-	-
PG_013399_T.1	3	upregulated	2	GQ03714_K21	-	-	-
PG_025065_T.1	3	upregulated	2	-	-	-	-
PG_030622_T.1	3	upregulated	2	-	-	-	-
PG_026395_T.1	4	-	-	-	-	-	-
PG_000071_T.1	-	-	-	-	-	-	-
PG_000073_T.1	-	-	-	-	-	-	-
PG_002264_T.1	-	-	-	-	-	-	-
PG_005390_T.1	-	-	-	GQ03007_O23	-	-	-
PG_017209_T.1	-	-	-	-	-	-	-
PG_018001_T.1	-	-	-	-	-	-	-
PG_024644_T.1	-	-	-	-	-	-	-
PG_029492_T.1	-	-	-	-	-	-	-
PG_029972_T.1	-	-	-	-	-	-	-
PG_035029_T.1	-	-	-	-	-	-	-

Supplementary Table S6: RNL and TNL genes present on the chromosome 7 of the peach genome.

	**RNLs**	**TNLs**
Sequence accession numbers	Prupe.7G138300	Prupe.7G012000
	Prupe.7G138400	Prupe.7G017000
	Prupe.7G138500	Prupe.7G026100
	Prupe.7G138600	Prupe.7G037000
	Prupe.7G138700	Prupe.7G037600
	Prupe.7G138800	Prupe.7G065300
	Prupe.7G138900	Prupe.7G065400
	Prupe.7G139100	Prupe.7G065500
	Prupe.7G139200	Prupe.7G065600
	Prupe.7G139500	
	Prupe.7G139600	
	Prupe.7G139700	
	Prupe.7G139800	
	Prupe.7G140100	
	Prupe.7G140400	
number on the Chromosome 7 / total number	15/19	9/192
percentage	79%	5%

Supplementary Methods

Reference transcriptome development

Plant Materials

RNA-sequencing was carried in seven Pinaceae species including white spruce (*Picea glauca*), black spruce (*Picea mariana*), estern white pine (*Pinus strobus*), jack pine (*Pinus banksiana*), balsam fir (*Abies balsamea*), tamarack (*Larix laricina*), eastern white cedar (*Thuya occidentalis*). We isolated several different tissue samples including four to six vegetative tissues (for details, see Table S1) from 4 year old plants grown under greenhouse conditions with natural light; we sampled the tissues from two well-watered plants and two plants that had not been watered for 14 days. The tissues also included several megagametophytes and whole embryos isolated from germinating seeds.

RNA extraction

Total RNA was extracted by grinding tissues in liquid nitrogen to a fine powder and by utilizing either 1) the cetyltrimethyl ammonium bromide (CTAB) extraction method as described by Chang et al. (1993) with modifications (Pavy et al. 2008) for the tissue preferential analysis and the drought experiment or 2) MasterPure™ Plant RNA Purification kit (Epicenter, Madison, WI, USA) for the RNA-Squencing. The total RNA concentration was determined using a NanoDrop 1000 (Thermo cientific, http://www.thermoscientific.com/) and assessed for quality with an Agilent 2100 Bioanalyzer and Agilent RNA 6000 Nano Kit LabChips (Agilent Technologies Inc.,http://www.agilent.com/), and stored at −80°C. Only RNA samples that display RIN score >7 and 1.9<A260/A280<2.2 were used for subsequent experiments. The minimum RIN score for RNA extracted from foliage was set to 6.5 due to chloroplast content.

RNA-Seq library synthesis and sequencing

Total RNAs from all of the tissues were combined to form a single RNA pool for each as shown (Table 1). We used 500 ng of total RNA to synthetize mRNA libraries with TruSeq® Stranded mRNA kit (Illumina Canada Inc, Victoria, BC), following the manufacturer's protocol with a few modifications. First, Illumina technology suitable custom adapters were synthetized (IDT, Coralville, Iowa, USA) and their concentration was reduced by half at 25nM per reaction to avoid adapter dimer molecules and tris-NaCl (10mM, 50mM) at 25nM was used to complete the volume. Second strand synthesis and marking clean-up and library amplification clean-up were performed using Axygen® AxyPrep™ Mag PCR Clean-Up Kit (Axygen Biosciences, Union City, CA, USA) whereas post ligation clean-up was done using a ratio of 0.85 of PEG/NaCl SPRI® Solution over Beads with adapter-ligated DNA. Quantifications of each library were determined by a Nanodrop ND-1000 (Thermo Scientific, Wilmington, DE, USA) and library fragment size distribution and the absence of dimer molecules were verified with an Agilent Bioanalyzer 2100 using High Sensitivity DNA chips (Agilent Technologies Inc., Santa Clara, CA, USA). Four equimolar pools of 17 to 18 libraries each were prepared and purified following the Library Amplification Cleanup protocol as described in the library construction protocol. The concentration and the fragment size distribution of each purified equimolar pool was evaluated with a Nanodrop ND-1000 and an Agilent Bioanalyzer 2100 respectively. Each of the pools were sequenced by using the rapid run procedure (2×250 bp) at the Genome Quebec Innovation Centre at McGill University (Montreal, Quebec, Canada) with an Illumina HiSeq 2500 sequencing system.

Transcriptome assemblies

The transcriptome assemblies were performed with Gydle software NUCLEAR version 3.2.16, RESOLVE verison 2.6.12 and VISION version 2.6.12, and guided using the white spruce gene catalog

obtained by the sequencing of full-length insert cDNAs (FLICs) from P. glauca cDNA libraries as described in Rigault et al. (2011). Raw sequence reads were filtered by trimming adapters and retaining segments with 50 or more consecutive Q20+ bases to produce high-quality (HQ) sequences. HQ sequences were assembled using reference-initiated read-mapping, followed by iterative rounds of consensus resolution, extension, remapping and de-novo assembly of unmapped reads, until the assembly captured 95% of HQ sequences. The assembly process represented each gene by a single transcript sequence defined by its maximal set of exons, clustering isoforms showing a nucleotide similarity of 97% or above in order to capture splicing and allelic variation. Representative sequences were oriented using RNA-Seq strand information and coding sequence annotation. Quality control and curation of each transcriptome assembly was performed by coverage analysis, visualisation and interactive edition.

Transcriptomic of drought stress modified from Stival Sena et al. (2018)

Plant material

We used young trees from three genetically unrelated *Picea glauca* genotypes (clones 8, 11 and 95) that were propagated in vitro by somatic embryogenesis and grown in containers for 2 years at the Vegetative Propagation Centre of the Saint-Modeste tree nursery of the Ministère des Forêt, de la Faune et des Parcs (Saint-Modeste, Québec, Canada). The plants were 40 cm on average, were potted in pots of 5 l containing a mix of peat, perlite and vermiculite (3:1:1, by weight) and grown in a greenhouse with day temperature of 23 °C, night temperature of 20 °C, 16/8 h (day/night) photoperiod and watered three times per week, for 2 months prior to the experiment. For the experiment, one-half of the plants were watered and for the other half, water was withheld; the plants were arranged in a completely randomized design. Plants were destructively sampled and the newly formed foliage (needles) was collected at 0 (Julian day, JD 64), 7 (JD71), 14 (JD78), 18 (JD82) and 22 (JD86) days from the beginning of the watering treatments. Two plants per genotype (replicates) in both watering treatments were sampled at each sampling point (a total of 150 plants). The watered plants were sampled 2 h after the last watering. The sampling time was at midday. At each sampling day, the midday water potential (branch) of four plants per genotype in both watering regimes was measured using a Scholander pressure chamber (Model 610, PMS Instruments, Albany, OR, USA). Foliage samples were frozen in liquid nitrogen immediately after removal from the trees and stored at −80 °C. The needles were ground to powder using a MixerMill 300 (Retsch, http://www.retsch.com/) and steel grinding balls cooled in nitrogen. Powdered foliage tissue was stored at −80 °C until RNA extraction.

RNA extraction and cDNA synthesis

RNA was extracted by grinding tissues in liquid nitrogen to a fine powder and by utilizing either (i) the cetyltrimethyl ammonium bromide (CTAB) extraction method as described by Chang et al. (1993) with modifications (Pavy et al. 2008). The total RNA concentration was determined using a NanoDrop 1000 (Thermo Scientific, http://www.thermoscientific.com/) and assessed for quality with an Agilent 2100 Bioanalyzer and Agilent RNA 6000 Nano Kit LabChips (Agilent Technologies Inc., http://www.agilent.com/), and stored at −80 °C. Complementary DNAs were prepared from 500 ng of total RNA using Quantitect Reverse Transcription Kit (Qiagen, Germantown, MD, USA) and then diluted 1:4 in RNase-free water.

RNA-seq library synthesis

We used 500 ng of RNA per sample to synthetize mRNA libraries with TruSeq® Stranded mRNA kit (Illumina Canada Inc., Victoria, BC, Canada), following the manufacturer’s protocol with a few modifications. First, Illumina technology-suitable custom adapters were synthetized (IDT, Coralville,

IA, USA) and their concentration was reduced by half at 25 nM per reaction to avoid adapter dimer molecules and Tris-NaCl (10 mM, 50 mM) at 25 nM was used to complete the volume. Second strand synthesis and marking clean-up and library amplification clean-up were performed using Axygen® AxyPrep™ Mag PCR Clean-Up Kit (Axygen Biosciences, Union City, CA, USA) whereas post ligation clean-up was done using a ratio of 0.85 of PEG/NaCl SPRI® Solution over Beads with adapter-ligated DNA. Distribution of library fragment size and the absence of dimer molecules were verified with an Agilent Bioanalyzer 2100 using High Sensitivity DNA chips (Agilent Technologies Inc., Santa Clara, CA, USA). DNA quantification was performed using Quant-iT™ PicoGreen ® dsDNA Reagent and Kits (Molecular Probes Inc., Eugene, OR, USA).Three equimolar pools of 20 libraries each were prepared and purified following the Library Amplification Cleanup protocol as described in the library construction protocol. The concentration and the fragment size distribution of each purified equimolar pool was evaluated with a Nanodrop ND1000 and an Agilent Bioanalyzer 2100, respectively. Each of the pools were sequenced by using the rapid run procedure (2 × 250 bp) at the Genome Quebec Innovation Centre at McGill University (Montreal, Quebec, Canada) with an Illumina HiSeq 2500 sequencing system.

Read assembly and annotation

Raw RNA-Seq sequence (FASTQ) files were filtered into High-Quality (HQ) sequences, trimming adapters using the NUCLEAR version 3.2.4 software (Gydle Inc.) and retaining only segments of 50 consecutive Q20+ bases. The HQ sequences were mapped to ribosomal and gene sequences using the *Picea mariana* Gene catalogue (Rigault et al. 2011) using NUCLEAR. A total of 22532 gene sequences (i.e. cluster representative clone sequences) were annotated by performing protein-level similarity searches with BLASTX (E-value # 1e-10) against proteins of Arabidopsis (Arabidopsis thaliana; The Arabidopsis Information Resource [TAIR] version 10) (see Rigault et al. 2011 for more details).

Table 1: Tissues and RNA used to prepare the pools used for the preparation of RNA-Sequencing libraries.

Plant	Tissue type	Amount of RNA used						
		Picea glauca	*Picea mariana*	*Pinus strobus*	*Pinus banksiana*	*Abies balsamea*	*Larix laricina*	*Thuya occidentalis*
Well-watered, 4 year-old tree, 2 trees	Young foliage	100	100	100	100	100	100	100
	Stem Apex (1 cm)	0	0	0	100	0	0	100
	Stem secondary phloem and bark	100	100	100	100	100	100	100
	Stem secondary xylem	100	100	100	100	100	100	100
	Root apex (1 cm)	0	0	100	100	100	100	100
	Root secondary xylem	100	100	100	100	100	100	100
Not watered (14 days), 4 year-old tree, 2 trees	Young foliage	100	100	100	100	100	100	100
	Stem Apex (1 cm)	0	0	100	100	0	0	100
	Stem secondary phloem and bark	100	0	100	100	100	100	100
	Stem secondary xylem	100	100	100	100	100	100	100
	Root apex (1 cm)	0	100	100	0	100	100	100
	Root secondary xylem	100	100	100	0	100	100	100
Seedlings at 1 cm root stage	Megagametophyte	200	200	200	200	200	200	200
	Embryo	200	200	200	200	200	200	200
Total amount of RNA in species pool		1.2	1.2	1.5	1.4	1.4	1.4	1.6

Table 2: Accession numbers related to the seven transcriptomes

study	bioproject_accession	biosample_accession	sra_accession	tsa_accession	taxid	organism
SRP134160	PRJNA437248	SAMN08646834	SRR6816980	GGJG00000000	90345	Abies balsamea
SRP134160	PRJNA437248	SAMN08646875	SRR6816981	GGJA00000000	3326	Larix laricina
SRP134160	PRJNA437248	SAMN08646877	SRR6816983	GGJI00000000	3330	Picea glauca
SRP134160	PRJNA437248	SAMN08646878	SRR6816977	GGJJ00000000	3335	Picea mariana
SRP134160	PRJNA437248	SAMN08646879	SRR6816982	GGJH00000000	3353	Pinus banksiana
SRP134160	PRJNA437248	SAMN08646880	SRR6816978	GGJK00000000	3348	Pinus strobus
SRP134160	PRJNA437248	SAMN08646881	SRR6816979	GGJL00000000	3317	Thuja occidentalis

Table 3: Additional data sampling:

name	Resource	Peptide dataset
Amaranthus hypochondriacus	Phytozome v12.1	Amaranthus hypochondriacus v1.0
Amborella trichopoda	Phytozome v12.1	Amborella trichopoda v1.0
Ananas comosus	Phytozome v12.1	Ananas comosus v3
Aquilegia coerulea	Phytozome v12.1	Aquilegia coerulea v3.1
Arabidopsis lyrata	Phytozome v12.1	Arabidopsis lyrata v2.1
Arabidopsis thaliana	TAIR	TAIR10 proteins
Brachypodium distachyon	Phytozome v12.1	Brachypodium distachyon v3.1
Brassica rapa	Phytozome v12.1	Brassica rapa FPsc v1.3
Cajanus cajan	Shao et al. 2014	Shao et al. 2014
Capsella rubella	Phytozome v12.1	Capsella rubella v1.0
Capsicum annuum	sol genomics network	Cpsicum annuum cv CM334 Genome protein sequences (release 1.55)
carica papaya	Phytozome v12.1	Carica papaya ASGPBv0.4
Citrus sinensis	Phytozome v12.1	Citrus sinensis v1.1
coffea canephora	sol genomics network	Coffea canephora protein sequences v1.0
Cucumis sativus	Phytozome v12.1	Cucumis sativus v1.0
Fragaria vesca	Genome Database for Rosaceae	Stawberry Genome v1.0 ab initio gene proteins
Ginkgo biloba	Guan et al. 2016	http://gigadb.org/dataset/100209

Glycine max	Phytozome v12.1	Glycine max Wm82.a2.v1
Kalanchoe laxiflora	Phytozome v12.1	Kalanchoe laxiflora v1.1
Linum usitatissimum	Phytozome v12.1	Linum usitatissimum v1.0
Malus domestica	Genome Database for Rosaceae	Apple Genome V1.0 predicted peptides
Marchantia polymorpha	Phytozome v12.1	Marchantia polymorpha v3.1
Medicago truncatula	Phytozome v12.1	Medicago truncatula Mt4.0v1
Musa acuminata	Phytozome v12.1	Musa acuminata v1
Nicotiana benthamiana	sol genomics network	N. benthamiana Genome v1.0.1 predicted proteins
Oryza sativa	Phytozome v12.1	Oryza sativa v7_JGI
Phaseolus vulgaris	Phytozome v12.1	Phaseolus vulgaris v2.1
Phoenix dactylifera	NCBI genomes	Phoenix dactylifera assembly DPV01
Physcomitrella patens	Phytozome v12.1	Physcomitrella patens v3.3
Picea abies	Congenie.org	Picea abies v1.0
Populus trichocarpa	Phytozome v12.1	Populus trichocarpa v3.0
Prunus avium	Genome Database for Rosaceae	Sweet cherry genome v1.0 proteins
Prunus persica	Genome Database for Rosaceae	Peach Genome v2.0.a1 all transcript peptide
Pyrus bretschneideri Rehd	Genome Database for Rosaceae	Pear draft genome v1.0 hybrid proteins
Salix purpurea	Phytozome v12.1	Salix purpurea v1.0
Selaginella moellendorffii	Phytozome v12.1	Selaginella moellendorffii v1.0
Solanum lycopersicum	sol genomics network	Tomato Genome proteins (ITAG release 3.20)
Solanum pimpinellifolium	sol genomics network	S. pimpinellifolium protein sequences LA1589
Solanum tuberosum	sol genomics network	Potato PGSC DM v3.4 protein sequences
Sorghum bicolor	Phytozome v12.1	Sorghum bicolor v3.1.1
Sphagnum fallax	Phytozome v12.1	Sphagnum fallax v0.5
Vitis vinifera	Phytozome v12.1	Vitis vinifera Genoscope.12X
Zea mays	Phytozome v12.1	Zea mays Ensembl-18
