# Supplementary material for: The large repertoire of conifer NLR resistance genes includes drought responsive and highly diversified RNLs
Source: Sci Rep. 2019 Aug 12;9:11614. doi: 10.1038/s41598-019-47950-7 (PMC6691002; doi:10.1038/s41598-019-47950-7)
Supplement: Supplementary file 2 — Dataset 1 [file 41598_2019_47950_MOESM2_ESM.docx]

>AB_000038_T.1

MRETLRNKGLQAVQTMLLKDLLHVDWNVRSPSEGKELLRKRLNVVEDILIALDDVDQHEQ

LADLLDLNALHPNAVILVTTRDKGVLQRFRGCLEYEVKRLEATEAEELLCWHAFGKEEAD

ENLRDLVVKLVGMCHGVPMLLEQCGERLYGKHERSYWDFQLAKLSKQLESHVEDVQIADG

STEQEGVYTYELC

>AB_000088_T.1

MALSLIEGAALGAALQVALEKGLKQLGGVSDPIISGKTWGEKLEETVSFLRPIIDEYIET

SSYPDLSDLSARRSEQFKDFQALLQSGRDLVRESDQIHSLDIKGKYVYGNKILEFNDEIK

DFIAIQGPPNLALDLQKVIAEVRNLGRRFERMERLILQTINPTQRSQTPIDRLQGATAVQ

QSDSFKSQVPAMPNEVVGLYNPVN

>AB_000089_T.1

MGGSGKTTLASALCHDPGVQATFQNNIHFITVSQLHRNENGLLEILDTLGDRVIGSDRPR

FRSIEDARNQLQNKINRIAESTHQPTLVVLDDVWSKSNLETLLFTAEGYKTIITTRYNST

IPDINGTQLYSMPELKEADALSLFCFWAFSQTSIPATAKEDLVRQVAEGCKGLPLALKVI

GSSLHCKRRPVWEFYKDKLSRSEPISKDHKDVLLSRLETSIDILSDKEKQCFLDLGAFPK

GRKFSVDSLLDIWVYVHGMEWPEAFEVLLEFASRNLINLTGYPGSAAIDYSCASELAFSQ

HDVMRDLAFHLSSQNSDIHCKRLFMPRKEAKIPTKWQSTLKDQSSSARFVSIHTGAMEER

DWCQTDFPQVEVLTLFFSASQYCLPTFLQSMTKLKVFIIYNYSSKRAIISGLPCFPSPVQ

VRSVLLHKLIVPPPLYKNCRSWAKLEKLSVCLCEGLGNITLLDKELEALDLPNLLEINFD

HCSDLKELPVKLCKSTSLERLSVTNCHLIENLPDDLGSLSSLRVLRLSACPSLSRLPPSI

CKLGQLEYVDISMCRSLKVLPAEFYWLSSLETLDMRECSGLKKLPIVKLRSLKHVIISDS

DKESQEVWLSIKESGIHNLIIDVVAELFSLDWLYD

>AB_000167_T.1

MNIFVSSKSSSEEVKQKLLLLKPAIDEISKLSSDVDVPAPQRGHPFKDFQAQLQDGLHLV

KKLEQLSSFNLYRRYRYGKQILKIEKNVNDFLLTQGLANLILDVHKLNVDFKNCSERSER

VEEMGRHIIESVNAKFTSDASFNSFMLQQMSSIQLFHSSIDGLHDTTMDEQSTSICNSQI

PGIPSFVVGLNIPVDDVKQILFQNEVNIIGVEGMGGSGKTTLALALCNDLQVKDFFQNNI

VFITVSQSPNVKALLETMWDKIIGAGRPDFQSIEDAHNQLQKNLSLKGNRRTLVVLDDVW

SRSNAEQLLFDAKGYKTVITTRQDYSIPRTNSSHVYHIPMLQRADALSLFCFWAFGQPSV

PETEDEYLVKQVEAMCMGLPLALKVIGSSLRNEPQPVWENARKKLSRAEPISQYHREELL

DCLETSIDVLDDESK

>AB_000195_T.1

MAALLDPITGKLLDKALDKAFTEVGGVVQIILSSKTSGKELKEQLSWLKPTIDQIIKVDV

SADSDTSPDIIKQYKNFQADLKEGLDLVERLLKLRSFDFFKKYRYGNKVLNLRKKLYDFI

IFHGPPDVILAGKKLDADLRVYFERLIGIVNAGMTNDPNSNTVMLQQIDVTQVFQTPNQV

FQNLSDHGMHEATAAQQSVSCTYRVPKMPTEYVVGLNNSVHAVKKILLQNDVNIVGVTGM

GGSGKTTLALALCNDTNVKDFFKKNIIFITVSTFLNENGLLEILGYMWDEMTGDHRSHFQ

SIQEARFQLQTNLSSRTNHPTLVVLDDVWSRSYLENLLFEAKGYKTIFTTRDESTFPIRN

STCRQYKMPMLENANALSLFCFWAFGQPSIPTTQEEDLVKQVAAECHGLPLALKVIGSCL

SNEPRPSWESARKKLSRAESPW

>AB_000396_T.1

MKGNTSLHDWHLAFNQMRNVDPDFLASTRIDEGLYQRLKLSYDRLPHSNIKNCFLYCAAY

SEDYVISVDYMVEIWIGEGLVNSRETSGLMDVGRRYVKLLEERCLFDTLDDGDVWRVKLH

DVVLDMATYIAEKEEKCFFRTRQNLQKFPSEKGIENCKRIAIGENNISVLPTDFRCPNLL

TLILSRNESLREVPNGFLVHLTSLRVLDLEGTKIEALPTSVWHLRQLEFLRLSHTLIKDL

PEDICNLSQLQFLHLNGCRQLESLPCKIGELQNLKTLNIGECCSLTGIPREISQLTSLNR

LSLATSEGWEKSIMDVEEVKSGVCSLKDLANCPNLMELLVHVKPGIEVEGIRSGIQVGIM

GSWVEMRDLRLAFGVEEHDVVEDLPQDMQNMKKLQMFALQNYHGRGLPNCISEFPQLEKL

ELYSCFQIRELPPLEKLPNLRSLSLNNCIKMKELGITGGFPMLERLELSNLPELESMGSS

SSSDVVWNEETMPKLQVLSITNCDSLKGLPMGIEKLPKLKQIEVQEDWWKESIWVETDAG

IQSENDA

>AB_000444_T.1

MGPEETIPDTRKYEDGQRELGYMLEKVTAFIYIDNVLGENELRQLLPGDLSKAKKLRLLV

TARDTNVGRAFPRKTSLEIYSMKRVLNSTSMRFLKREVLDDMEEKLNTSQIDSLIDICGG

VPLMLTLVAKVLSFGQDKQKAYNMVIQHKQNLKGQMFERIGGEEQYSFAAGEAQTFTSLK

APSGSSKPAALSCTAEDLKGKTILLEIRPLHLGCDLRLASLIQLYNTKKETFNLEIISIP

LVQRSSSSTLDVFEKFSRDIPWLVIQNPWAVPSAVKYFLMELFAPDELDADCPYGIKIIE

SNGRIFTPSKPVMGLLDRCGAKAYPFTVQKIEELKKEEWNQMETMPTLEFLFNGLESVSD

QVKEMMLHQKVVCLYGGIESENTMKEFTALIRNAFISVKDNIHIIYIPSFTLKAEEKSQV

TDDIHWSYQPLEMSTLAQAEMEGMLLLNPSQHDAFRFWRRIALLEEEMPEMHNRDTEQIS

VMKKIFQCWNNENPWMILMDEEGKTITESGRKIVWSCKGSMEDEAKAWIELLIKGSPEQR

KEALSELEQWPDDSN

>AB_000550_T.1

MASSSSFSASASASNYDVFINHGEPDVKRTLGSYLYHRLVSYGMRVFLDVTEMKLGSNLS

SQIKGAIATASVHIAIFSPRYAESTLCLNELVMMLESGAPILPVYYRVTPNELRRKVKRG

QGVYGRALHLHERKKRLDGKTNQEMPRYDSNSIENWRNTLSVVSDRSGFELQEFNGDEGE

LLDKVVERVLELVRRAGPLHEERGARVHPIGLDEEVDYFENIVLSQHQTGKPLVAWIVGK

AGIGKTTLAKEIFNRKRSFYSRSCFLSNIREKARTCSLHFLQRLLCEDLINKNIGSGPEA

IKLFKELASYIYGSSALVVLDDIDDVDQLHALLPSQNYPPSDGLILITSRHEDVIRSFRV

EKPTVYKLTGLSPRHSRELFYSKAFGQSLRSGGFEDLDLIDRILEACDGIPLYLNVYGSL

VCPEKGTSYWRDQVETLKQLDLNQRLPEWDLEQLERLPRDIENIKKLSRKEAIDILKIDL

LQRKRGKVNVVGIFGLDGVGKTTLVKELFESKKLSYSRSCFLYEVRASYLQSLFSKLLKD

LIPLDRPVESAEECIALFKEAKQVFPSLVVLDNVDHWDQLQALLSVLQNVLPSSSLILIA

SRDEHVLNRSGVQGSSIYKLECPDWQVSRELFCSRAFGQPSPLPEFEGIVDEFVTACNRM

PLLLKVFGAILGAKTDRSYWQDQLDRLQRSRDIQEKLEVSLDTLNREEKHVFSYIACCCI

GEKSDLARRLLEAFGPAVLSGFESLQKRCLVEVDSEDCIIMYKYLRDLGRTMADERGLRP

LLWPPTENNSSLLQQSSNEQIITEPRRNIDDSLQQSSVTREVRVFLKMIFVMMIIVAMMM

AMINWSKGLTIILITMITAMMIWMG

>AB_000665_T.1

MYRLAKHVKELKERPRLRVGMGDIIQEALEMIVKASTMCCVQIDSSKFSRFFKTTVDKEE

LLKFQRELENMYKHMKNQFDICLYDAIDCRKVRPRTTLEREYPVYAVGIEEPVKEVTKLL

DWESEKNAVAVIVYGFGGVGKSTLADAVFARLYIEGRKYSMVRLFDDITSTPNITKLQKC

ILTDLMRGTEEEKKPMPEFTTFEVGQREISRILETEEAFIYIDNVLDGEWLQRLLPRDMQ

MAKKLR

>AB_000701_T.1

MLHKLVERVEKMAKKTALAVAQHPTGLDEKIKDFETTVLLSNQRTGKLQMVGIVGLGGMG

KTTLAKQFFNIKRSEYNKSCFLQDVRENSKSSLHSLQNKLLKDLNPSNGHNIDKVSIHEG

TEMLRKFLSSSQVFVVLDDIDHEDQVVALLPGRDGLRMDSLILITSRDKDVLARSGVEES

SIYELKGLDSKHSQELFCSYAFCHPYPLGGFEDLVEEFLRACDGLPLSLKVFGALLFCQK

EKSQWKDILDRVQLPTDILHRLRISYDALSREEQEIFLDIACFFIGKDKNTAIKFWKGSL

GFRNLQGKCLV

>AB_001303_T.1

MPIKDVLHSESLFLVTSRYKDVLTSSGIADSSIYKLIGLNKQHSRQLFCSHAFLQPFPLP

GFEYLVGEFCKACDGLPLSLRVFGALLCGKNEKPYWEGQLIKLRQIKLPNEIKERLKISY

DALEEDEKQIFLDIACYFINKERDTAIKVWDGSGWNGLVGFRNLQDKCLVDVHVANVNDY

DDFGVFNVIKMHDHLRDVGREIADLEMSSGVTPHRLWRVIEDIDDLSKQSSVITEVRGIR

TAASEFIDFNEISESEELFDQVLGKRKLTDRIPYCFTKLQLVETQGRALKTILRMVESPN

LKWLSWTNCPYTSLPKWIPMKNLRVLDVQGEFLETLWKEESQVPLELRELYIRTYSWFQF

PEYSWFQFPKSIERLKHIEKIVFKYNLQGLPDSFGCLTNLQHLDLSQCESLQGLPDSFGC

LTNLQHLDLSYCMSLQGLPDSFGWLSANLQYLGLIGVSTKSYLGLIGVPKSTSAIRFQHL

CNSCGRRRKNDWLCDIADLEEPNLCICIGVEELPRLESFEDHCSSCGRLRKIDWLCDLTD

LKEPNICMCLGVEELERLKSLKKSAGRY

>AB_001433_T.1

MLKKHLSSLDRVLIVLDDVDHIDQLDKLFSPVKDTIHSSSLILVTSRNKDVLTSAQIDES

SIYMLKGLNQQYSRELFCSHAFNQPYPDIGFEQLVEKFLGACHGLPLSLKVIGALLCGEN

LTYWEEQFHKISRVVPTDIQSRLKISYDGLDEEEKQ

>AB_001495_T.1

MGGSGKTTLATAFCNDKEVQDHFKERIVFETVSRSPNIIEILGSMWLQLAGKEKPEFRDI

ENARRRLQNKLCLKESRPTLVVLDDVWEKCDLDKLMFEGAGYKTLITTRNNQILISDQQY

ENQLLKPSDAVSLFCHFAFRQPSIPETADGDLVKEVIAKCDGLPLALEVIGRSLNGLLSE

AWVTAKKKLSEGQPVSDDHKKALRVMGTSVDILEKEVRECFLDMGIFEEARNISADMLLD

LAVYVHGLERAGAYTILVELASRNLVKLVNNQKDTLWDSYEWQELSFTQHDILRELAINL

TNEEETIVNRRKRLTMTRKAGVSFGKMWKKHADDVPVFNAEIVSFNTGQRFHAGGGLVLH

GLS

>AB_001885_T.1

MYKHMKNQFDICLYDAIDCRKVRPRTTLEREYPVYAVGIEEPVKEVTKLLDWESEKNAVA

VIVYGFGGVGKSTLADAVFARLYIEGRKYSMVRLFDDITSTPNIAKLQKCILTDLMRGTE

EEKKPMPEFTTFEVGQREISRILETEEAFIYIDNVLDGEWLQRLLPRDMQMAKKLRVLIT

AREKGVRTVCTGCGMEPRDYALTGLPNSEAMNLLKKEMKEGQIQSSQLDQIIKISGGIPI

LLTMVARFISSEEDKQKAFRIVMEDKEHLEGQAFGDIEHYVFAYDHLPERCKEPFLDICV

FFKGMNWDSVADIVGESELDMLEKRALVSKDTHMNITVHDVILKIGIKRSEETRFNSTNA

TRSETEEFLQKEIPGIRGLWLRGISEIKGLKDQWDIPAIKLDSMSNSLKILRLGNSKVKG

ECNKRLEKLVFFEGKVPLFPFNISQMSSSLKYMELNGRGHSHVFEISPSDCQRLRNLRIL

RLGNFDGLKKLPEELGELIQGLRELTLSLCKSIEELPRSISKLQYLRILKMTYCPTLTRL

PEDLGSLSSLQELDLSFCEYLKELPESTSKLESLKKLKMTACESLKHLPKDFELLNLQVL

ELQDCKSLKVLPRNFEKFQNLKPFVETFSLDWLDDDEEDY

>AB_002226_T.1

MGIGHMVTTSFVVVANMLERFEEFGDNKIECLGLLKKMLSLAKLVKQFQERPTLKESEGV

GDVIKKATGLIVEGSVLCCTQMKSSKFSNFWKAPAMKEEMSKFRQKLNDMYTHMNAQMDI

TIYDVIAPKKPRLSRGYPEHAVGIDDAVKEVLQLLEWESGKKAVAVMLHGFGGMGKTTLG

DAVFSKADFEGCKYSEVQLFQNIGSKPDTVELQKCILKDLMGPEETIPVIRKYQDGQREL

GNILEKAPTLIYIDNVLGESELRHLLPLDLNKAKKVRLLLTARDVNVGIACPRKTSIKIH

GMKRFSNDEAMSYLKREVLDDMEGKLNSNQIDNIIEICGGVPLMLTLVAKVLSFEEDKGK

AYNMVMQDKQSMKGQMFAMLFVKEDYFFAYDSLPEKCKDPFLDVCAFFKGWEWETVADVV

GEFELDILEKRALVTKGIDGVAYVHDVILTIGQEKAEGTRFKVTSAIEVEEIFHANEENE

DQGIKGIWLNEIKDPYTIPASNLDRVCKSLRVLSLGNFTKVEGQCNKIFEKLIFFQGVIT

HLPFDISRLKKLTYLAYEPEDLTLPKMPSSLRHVELDGRLHSLAFQISSTCFQQLGNLRI

LRLIRFSELKNLPQEIGDRVKALQELSLSYCKSLEELPISISRLQDLRVLRMDACSSLGQ

LAEDFGSLKSLQELNLEGCLSLRQLGEGFGSLNSLQELNLKGCTALKELPNNFEKLSSLR

SLNLSYCENLEDLPHGLGKLSSLVALHFHHCHKLKSIPDSIGQLKLLDSFGFNVVSFCSS

LTELPDGFCNLSFITKLSLSDCKSLHRLPNRLGELASLRILFLDECSSLQKLPESFGQLK

YLEHLDLSGCVSLEELCNEFHCLSSLHVLAISGCKSLEKLPEDFHCLISLQHLGLNVCPK

LEGKSMDKVIKINALKVVAIAGSGRLEDRWAEIERESDKSWPFKVFTRQGISTDENEKIQ

KDMLTQLEFLLSEGRGKPFHLSGLPLVIILDTLADYNHNFPWPLIEETIDDIQINFEMVY

VGDDFDKLPPKAKNNIIVGHFNGTEFMNFFKHAIKIR

>AB_002334_T.1

MLQTNAPIIPVFYGVKPDQVRWTRGDYAEALKAHEKKRRHNSKTIEDWRNALSKVANKSG

FELETCNGEELEMELVNKVVKRILEMVKRPDLINF

>AB_002375_T.1

MTRDFPFNNQVYQSLPPCCNTCENLLYGCERLGISILKQYFLWYQFPELGMEFIETAQTA

LTELGGVIEIIYSSKTWGKELKEKLLLLKPDIDGAIELISRSDSSLQRQRQFADFQAVLQ

DGLNLVDEVDNLHRFDIYRKWRYGRRIREFEEKIKDLISIQGSPILAHDLQRLGSDVTDL

RRRFEAATIADQSISYTSHDVPDMPKFVFGLENRIIAVKQILLQKGVDRLGVIGMGGSGK

TTLASALCKDTVVKADFKNNIIFIPVKQTNPNVKGLLETMWDKIICTQRPDFQSIEDAHD

QLQTNLRLRTCRPTLVVLDDVWSTSDLEYLLFEAEGYKTIITTRQDSIIPITDSTRLYNI

PLLDDANALSLFCFWAFGQNSIPTTQKEDLVKQVAAECNGLPLALKVIGSSLRDKPRPVW

VNADRKLSKAESISAYHRERLLKRLETSIDVLDDKHKQCFLDLGAFPRGKKFSVESLLDI

WVYVREMEWTDAFEVLLELASRNLLNLTGYPGSVAIDYNCASELSFSQHDVMRDLALHLA

SQASIISRERLFMPRKENKIPTEWLSTLKDQSSRARFVSIHTDAMQEQDWGHIDFPEVEA

LALFFAANQYCLPTFLHSMSKLKVLIIYNYSSMRAILSGLPRLPSPVQIRSVLLNKLIVP

SPLYENCRSWERLEKLSVCLCEGLGSITLLDMEIEPLNFPKVMEINIDHCSDLRELPVKL

CTLTSLQRLSVTNCHLIQNLPDDIGGLRSLQVLRLSACPSLSRLPHSICKLGQLEYLDIS

LCRCLEGLPTEFDQLSNLETLDMRECSALKKLPGIKQRSLKRVIMSDSDKESERAWLFIK

ETSIHNLTLDVVAEQFSLDWLDD

>AB_002572_T.1

MILQQMGAADMFDEAFYDSPCCFDGLGNSDFVVGLEKNMWNLKRILLQKEVSIVGLHGMG

GVGKTTMALALCNDQEIKGVFRNNIIFLTISQSPNLKGILETMWEKITHTKNPKFQNVED

AHRQLQQLLQRQDKPTLVVLDDVWSRANLEELLFEGEGYKTLVTTRDCSTIPRETSTQIY

ELPLLDDGDALSLFSFWAFGQRSIPSTADEHLVKQVKEKCKGLPLALKVIGSSLHGEPRP

TWESAKNKLLN

>AB_002734_T.1

MLETSLKTGATVIPVFYDVEPYEVQCAQPGNKGRYSESLRRLEDQMKRRYHQTHYDSNSI

GKWRDALSKAGDLSGFERTAYNGDEQELVDEQELVKNVVERVLEILGGTRSKITEHRAGL

DEKVKDFENKVFLGHHQSAKPQVVGIVGIGGIGKTTLAKELFDQKRSHYKVSRFLSNVRE

NANKGSLPSLQRQLLKSLTGADVVIDNIDKGKEELKKHLSCYHILVILDDVDHMNQIDAL

LP

>AB_002735_T.1

MAASSSTAASTSVARNANTKYYHVLLHCRGVDVQKTFARSLYLRLISNGLTAFLHQEPDE

IQQGYTITSQIKHAIGTASAGVVIFSERYAESTWCLDELVMMWDSNKPIIPVFYGVTPAE

VRWPRGKIGKYAQALENLERKRTFEGEPRYDSNTIEKWRNALSNVADINGFELEASDIGG

EVEMELVDKVAQRLLELLEQLPQHSLNFAQYRPGLAEKLKDFEEEVLFKHHQSGNARVLG

IFGLSGVGKTTLAKEFFDRKKSDYSNICFLYNVRENAVEGFLTSLQRQLLESLTRSDVPI

DMSIDQGKEALKKHLSSNRVLVILDDVDDKNQIEALLPVRTDLPSDSLLVMTSRDEMVMR

NSGVEKASIYKLTGLNYQHSRELFCNHAFSQPLPYIPFECLVDKFLEYCDGLPLLLKVFG

ALLFNKYEQSDWEDLLDKLRQQLPTEIQEKLKISYDSLTMEQKEIFLDSACFFIGHKRDT

AIRIWDGSGWKGSLGFRTLQSRCLLELDSENNIHMHDHIRDYARTVADTSLPRRLWQCNA

IKDMLEQLSDRS

>AB_002827_T.1

MAEAVFISGFAGAGIDIVVKQILHQIENAIRYKKALRSLESLVKNIQPIIKEIQRYRPAL

NRKNGIPTSQCYNKASAVSEWLKQLDLLLRQASEMVNQCTLPWYKGHLTSRKVSSLISNI

DEHLKVSPLVGLAQIPELLEEQTQVLLQGFGQIMNESVASSSSSSATASQSLASTGFFIA

EPLIVGQERAFAALEKQVIDSELSSVGVVGKGGSGKTLLLKRIFNSEKVRNVFS

>AB_002839_T.1

MATSSSTADFRDSSSNYHYDVFLNHRGTDVKNTFASHLFRRLLLHGFQVFLDKHGMQVGE

NLTCQIQGAIRNASVHVAIFSPGYAESKWCLDELIIMLESKAPIIPVFYGVKPAELRSTR

GKCGVYAQALDNLEKKKNHEEKPRYDSNTVEKWRKALCDVAEISGFDLQDACNGDEGELV

EKIVQRLVKLVKHTLMYVAKYPSGLDEKVEDIEKTVLLKQQQIGKAHVVGIVGMGGIGKT

TLAKELFNRKHSDYSRSCFLSDVRESASKTSLNVLQSKLLKGLTQLDIQIEHIGEGRERL

KDHLSFSRAMVVLDDVDHPDQLDALLPLEVKEVLRSDSLILITSRDRNVLARLGVEDSSI

YNLTGLSTQHSRELFCSYAFSQRHPLSGFEDLVDDFLRACDGLPLSLLVFGALLYGNEDK

SFWEDQRDRVQIPGDIQQKLKISYDTLNREEQQIFLDIACFFIGEMRDMAIKIWDGSRWK

GLLGFRNLENKCLVEVDSKSFIKMHHHLRDLGRDIAENSGLPLRLWGPSEKLADLLKQSS

EISDVRGIRMAPSVDRDDVQYEILRSDMPWYKRKSHELFLNRGMSLRNNRTHYSGSGRTK

LHFLETEDALLEHILRRVQSPNLVWLSWSKCPYSSLPSWIPMENLRVLQVAGSELKTLWR

GQLQAPLQLRELQIIAPLSKIPKSIGKLTHLERIEVIYRPRLPGQIKLKKLPKEFCHLRS

LKALVLRGCSRIKSLPDSFGDLTNLEHINLSGSNNLEILPNSFGDLIRLKYLDLNDCSNL

TISKETFGNISTLEYINLSYCEKIQVLPSQVAHQRSLENLYLRNTKLRDLPSAIGNLSDL

EILELGSPLLETLPSSIGDLRNLKELTLWNCKRLKNLQDSVGLLAQLTKLRIGFCPLREL

PFKMDEGERETITEPRGLRTLGNLDSSIGKYIFRLQHLELFSTKISKVSFPDGAFPNLQH

LLIRFAYDLVEVGTLPNTLIQLELIGCCNLRKLEGLCGLPKLQSLNIGGCKKVEQLPSIE

TLVSLQELWAYECVKLKRIQGLAQLTKLRKLDASDCTELEELEGVEHLISLEKLHTYGCP

QLHWDWAVLEQLRQRLMKEGMLVI

>AB_002971_T.1

MKPGSLIVVTSRNKDVLLKDIPQSSIYTLTPLNLQYSRELFCSHAFRPGDSVEGFDKVIE

EFLDACKGLPLSLKVIGALLRAGKGKLNFWEAQLRKISKELPLDIQSTLKISYDSLDESE

KQIFFDIACFFIGEDKDTTIRIWDGSGLEGLLGFWKLENRCLVDVDSGNLIRMHDHLRDL

GRDLAEKEHPGCRLWRPTDNLLNNLSGQSHVRGISMVGLNDPEQSFENLAGRHLVTNMSS

LQLLRAEGYFVERLFSLGNLPQLIYLRWDNCPFSSLPSSLSIKNLRVLYLQGQNLETLW

>AB_002974_T.1

MHVGDTLTSQIPAAINTASVHVAIFSPKYAGSYWCLEELQLMVESGKTIIPVFYNVQPTE

LRRTHGDGVYARALGILEGKKGSDGQPRYNSTTVQNWRAALSKVADISGLELERFNGDEG

ELLDKVVESVLETLNKTLKLNKRFDVAEHPTGLDEIITGFENTVLMQQQSGKPKVIGIVG

LGGIGKTTLVQELFNRKNSDYSRSCFLCEVRENARKHSLEYLQMELLKDLTQLDQQIKSV

SEGKQMLRNFVKSNCNCKALVILDDVDHKDQLDALLLPIRDVLQSDSLVLITSRDRDVLR

KSKVEESSIYKLTGLNPQQSKELFCLHAFTQRHPHPGFEDLVEKYLNACGGLPLSLKVFG

GLLYGNDDDKSFWEDHLERLEQILPDDIQKRLQISYDALQWDEKHMFLDVACFFIGENKD

TVIRIWDGCHWKGSLGFRSLQNKNLVDVVGTENEIRMHDHLRDLGRDLAKNPDLPLRIWR

REGMDDLPHQSSEVTEVRGIRMGRTEYEDLSSFLGGKMKKLQLLVMEDFNHLGDFNIDIE

SILDVVQSPNLIWLCWKSGGHSSLPWIRMRELRVLEVCGTHLETLWQEERQAPLELRELR

IKARRLTSLPESFGKLINLQ

>AB_003015_T.1

MGDPGFVSGSIGVVIDRLIQVIQRHINIAVCCSKELRLLEKLVKKIQPVNMEIQQYQIAI

RQSQSDSLKKIEPVAVNNWLKTLNGILEDASKLVHQCQVPVYNIVSRYLLSKKIRKSIDS

LQTHLEGVPLIGFTQLLRNELQNMLQSQEAAPNRSSIPSTSQPSLASYRQQPIQEACIVG

QDEVFIILKRLLIAAPDSSRIGVFGMGGAGKTLLLKRAMNDKQVQQYYEKDLILWLTVSQ

NLSITNLRNNLGRQIDLHINEGFDIRWSEDDVQRWIYEKTSSRRFLLFLDDIWDESENLL

EKLGVPDNQVNGSSKIVVSTRDRRVLSKMRVESPIGMENLSPEESWRLFCFHAFVGNQHT

PHLGIEKIARDVCAECDRLPLALKVIGAAMAGITALSGWRLTLEKLQNADKLDPQIEKKL

YNRLRLSYDALCTSQYISLQLCFLYLGAFKEDEEMYVQDVIKLWIGEGLLPTEEGKDPLE

VGRTYANFLVDRCLIEATITDADGQVVIFSVHDVLHDLALQIAEKEDDCCFRAGKGLEEF

PFKHCIGKSRISLRANNLKSIPGKYRGQDLRAMLLSENKFLEYIPSSVITTLTSIRLLDL

EGTSIKSLPDSFGALKHLVCLRLAKSPIGKLPDSVTCLKKLEVLDLSECYQLSELPRELH

KMTSLIYLDLSFCQQLTCMPRGISALSSLQYLKMEGCWKAWQETAKPRKLVRESRPRARF

QDLHTLKHLKWLALEGSQDMMPITDGIVGDMIEMRTLILRMSQMKELPEDMGQMAKLTTL

FVESDVLGKIPSWICGFEQLSCLILRGCTELKEIPAGLEKLEWLRRLDIVKSSSLKELPD

AFGETGAFPRLESFWLEETEVVEAFPQIREGALPLLKKLGLVLCSNVRTIPPGLENLHNL

RELHVYSSPLILESMEEVGGEAWCAVRAMEGERHVEILRNLIHDHRHKDGMGCSSICKRV

F

>AB_003230_T.1

MYEISAIHFIFSFSCVSHGKPFGIILQLCIPLLLVVYPMANPLASSSNSSANNSPYEVFI

NHRGPDTKESIAGHLYSSLTPHGLRVFLDKEEMEKGDFLTSQIAAAIRTASVHIAIFSPR

YADSEWCLNELLLMLESMESRRSTVIPVFCRVKPADLRWTLGENGVYAKSLTSLQKKKTT

DSQTHRKKPRYQKDTIEKWRNALSKVSHITGFDLEEAYNGVEWKLVEEVVQLVLQTVEKP

PLDVSKYPTGLDQKVKAFEELLQQQSGGP

>AB_003599_T.1

MENSTFVAMNNGLLSWRADVHLCICILVALCFALKHLICWILFRSLRASISKNATECIAS

SLDILEKRRFPRDGHDIQYDVFINYQNNETSANRFVDNLYEVLKSCGVRAFVNSREAAEE

NSEEVLDGRTKHAISTAKLHLAIFSKGYAESPKCLSELYEMVKIGKKVFPLFFDIEPSDA

RWIRRSYAEAFKRHERQQRFQIETLNSWKDTLHAVSLITGWELVQFKGQEGKLLKKVVQD

VLVEVNEVPLEVAKFPVGLSERTANLEALLLSNPDGNKATVVGIVGMGGVGKTTLAKATY

NRIRPNFRAATFLHDVKEFIDRDGLTTVQEIILGDLLRFDCKIRSVDEGKVFLRRRLDKA

KNILIVLDNVDSFDQLDALVVTKVLGPTCTVLITTRDKRILELAQISMVYEATGLNTDQA

TELFCRHSFLSPRPSVGFDDLVLKFVEALDGLPLSLETFGSHLYGKTDRKIWEVILGKIT

RILPWNIKERLKITVEELDEEDKSMFLDAACYLVGESKDTAIRIWDASKWSGWLGFETLE

QKCLIRVDTKNRITMHDHLRDIGRDIIDHESKHFPGRRSRLWRPNEIIKVLTENSGTEAV

RGLSFASRSSNLSSNNESGVPITWQAESLSRMKDLKLLLLQGTSLDGDFSQLSKNLLWLR

WWDFPYQCIPPNLPVGRLEVLDLGRGRVVKLWDENDCSQLPLNMRELNLTECNQLQRVPK

NIRQMRVLQKVVLKRCRLLSTLPEEFSDLHFLEHLDLANCRSLRSLPNNFGGLKHLRHLD

LSFCSKLKMLPDSFSQLLLLKYLTFEKCKILNIGPNILGMSSSLEHLDFTGCDKVQVLPC

NISSQRHLVRLYIHCRGLKQLPESLGELLGLRYLTMECPQIKEIPDSLGNLIQLKSFTLG

SSRLRHLPESVGRLKLLQQLGIKCHRLSYLPNAIGQLNNLQRLSLRGCKALQNLPPSFGN

LTKLETLDIYDAPNLRIPPGILDGLRSLEVLSLYGCKSLAEGCIISLCQNTQALRSLRLR

KLEVENCLRIPEHTCSSLETLKVYACKNLVTAEICSTISREVEFIACLQLRTISGFSADT

RLTKLCLRNCPELSEVTNLGDLHCLETFDVGGSLKLFSEGGLYLLKELKELDISVTHEAL

QGQCEWLQKLPSPGQLRISADSICPDTTEGLSHVLKPFHPMEKGRDGHGLEFKIPSDTEM

PCGAVILCFVSSHLVDGRDDHEIMLELYHEHNNRIRPFSYCYIKYADLICLHIFSADTFL

VRVLRSGDVIIIKPSETMNDSPMVLGGGWAHIVQRGEEFEIDYVRNAFLMEVGKQLGKSK

SGM

>AB_003655_T.1

MATPAVSSTAKNTSSHLYEVFINHRGPDTKKSFAGHLYNRIRSHGIRVFLDNPELRQGDN

LTSQIVSAVETASVHVAIFSPRYAESNWCLDELVLMVKSMKSGATIIPVFYNVEPADLRW

SKNENRRYTKDLDILGEKKTDEGRRRFDHDTVEKWRAALTRVADITGFDLKAFNGEEGEL

VEKIVEHVSKRVKKTRFSVPEYEIGLDAKVKDFEDKVLSQQQHNGKPQVVGIVGLGGVGK

TTLAKEFHRKSSSYINSCFFPVSQIARRTSSQFLFGEVLKRLTRPASDLSLNNVTEVKNL

LEVNEPSLEMIKKYPSLVVLDGVDDAEKRDGLLPIKEVLHSNSLILITTRDIDLLTGAGV

EAASIYNLNGLDTHFSRKLFCFHAFGVATPLQGYEYLVDEFLTFCEGLPLSLKVLGAIFY

RENDRTYWENQLHRFQQILPHTIEEKLKFSYDALKKEEKHIFLDIARIFIGEKADTAIRI

WKGSGWGGFLEFQSLMEKRLVEVDSENYIDMHDHLKDLAKTIAEQASSPRPLWLKTDNNA

DSLQSLQESSLIAELRRNIDDLLNQSSVIKELRSFMLTIIIMMATLLMMTAMLIWSMPRR

H

>AB_003780_T.1

MDTLFLPVKNAMKPGSLIVVTSRNRDVLISSDIPPSSIYTLTPLNPQYSRELFCSHAFRP

GDSVEGFDKVIEEFLDACKGLPLSLKVIGALLRAGKGKLNFWEAQLRKISKELPLDIQST

LKISYDSLDESEKQIFFDIACFFIGED

>AB_003930_T.1

MASGLVLQSLAGAVPSELLAIIKDVLKKSFACKESCKRLQKTLEKIIPVLGDDLNSDVEL

PQHSLKMLNDLYQELQKGIKLVQNCSKVNRLNIYKSYNYANRIQGLELYIVNFTQTEGWA

HICSELHSLRVHQHKLETTVHKMEDKLEHIDHKMENKLENIEHKIDAIREESDIKFIADQ

MDRWEIGKKASQSSVPDLPDFRVGLKGLVHELKKRLLRKDVPLLGVIGMGGSGKTTVAIA

LCNDTEVKGHFKERIVFERVSQSPNLKGLLERLWDKIVGGNRPDFHDIEDAHLQLQQRLR

RMEPQPTLVVLDDVWEIDHFEKLLFEGKGYKTLITTRNNNVVKSDYRYDLPCLREQDAVS

LFCFSAFDRKSIPETADYNLVKEVIAECKGLPLALKVIGRSLHGQPHAAWMTAKDKLSQG

QIISEYHKTEVLKRMATSIEILEDEVRDCFLDMGIFPEARKISADPLLDLGVYVHKLKWT

AAYTILIELVSRNLVTLVNSQKGTLGNPYGSSSELSLIQHDVLRDLAIYLTEQESTFNKR

KRFIMPQKADSIRKMWKKHAGCDFDAEIVSFHTGLMEEKDWCQMNFPNAKALILNFSASE

YFLPPFMQSMHKLKVLIIVNHNSKRATLKGISVLSSLTKLKVVRLERVIVPPLQEYCQSW

QKLEKLSLVLCEGCGNMIGLHMGVCLNFPRLLEFAIDHCSDLRELPTSICNLTTLKQLSV

TNCHDLHKLSDDIGNLSSLQMLKLYACPSLKELPYSICKLRQLQFLDISLCGYLKQLPDQ

LGHLSNLKELDMRECSRVKQLPKSASDLRSLVHVICDEKIGQQWSYIKASTIPRLFVEVA

EEHFHLDWLED

>AB_004081_T.1

MGAAHLTCTGLLIAAHVLERIETVSANVDKCLLVLDEICNLAQHVKKLRQRDGLRKGMED

SIKEALGLIVEASIMCSNQIGSSKGWRFLTALENKEKLENFQQSLQRMYRHMDNEIPICL

YDAIDCRQVRPQKPRERPYPDYAVGIEKPLEEVKELLEWESEKNAVAVILHGFGGMGKTT

LADAVFARVYVEGCKYSMVRLFDDVTSTTSTADIVKLQTYILKDLKMETPEETPAEIRTP

EDGQREIGRILQKEKAFIYVDNVLQRYPFEQLLPRDMDTAKKLRLLITTRDTDVKRECRK

LETKIYAMKGLPLTEAKSLLEKEMYTSYTDNKEKQLSPTQLDEMVAMCGYIPKLLTVVAG

FICCEEDKQKAYIRVKEERERNWKGEIGKDIAHYVFAYEFLDEGLKDPFLDICSFLKGCE

WSDVANIVGDGVLNSLEKRALVTKKTETLWVHDVILAMGHQQAEGTRFNSADQIEEFLDE

KEDEDIRKIKGIMLRRGKADLRPLCISASKLDLMCSSLRVFSIQHYAKVEGTCEKIFEKL

VYVEAKVSDLPFDVSRSKELKYLDVWDDWDDSNQDIEKLIGSISKFRYLMVLILRNLRQL

PEGIGSLKFLQNLYLENCESLEALPSSLTDLSSLRLLDLSECQSLKYLPCNLGKLTSLTE

LFLNGCQELISIPSSLEDLSSLRLLDLSWCQSLKNLPYGIGKLTALANLRLEGCKELSSI

PEGIGQLKSLESSINLSGTGLKELPDGFCNLSFITHLRMDECYCLEKLPCRFGEVTSLKE

LSLTSCVRLKKLPESFGQLKYLERLDLSKCTILEELCINFSSLQFLSLDCCEMLKKLPED

FHCLASLNDLRLRDCHMLEGKWMESMVKMKTLQDVDITGSPLLEQRWEEIQRQGDESWSF

VLYTGRQFSSESKEHIFKQLNSEFDSGDESWSFNLYRGRKKMVLKVAIEDEKSKRKALKT

VAGIKGVESVAVDMKERKMTVIGTADPVFLTTELRKIGFTELLGVGPAKEEKKAEGEKKE

GPKKEEKKAEPYPYTVVTDDYNPNSCAIC

>AB_004116_T.1

MEHVKWHPLEMPLLAVGLDQAWKDLHDQIKKQSSKKVVVIAGLGGIGKSTLAKHTFNELS

RSDFRRASYLFNVRERDDHDLQRQLCRDLLGKNMFDSWREGKTTLRKSLKGLKVLIVFDD

VDDREKIKSVVDLDAVEPGSLILITSRYKDILGGSSASTFLYDLKLLKGNHARELFCHHA

FHQLNPPEKYGDLVEDFLKICGGLPLCLQVLGEQVCSNSDRAHWERQLKLYSQGASLPEA

NDVIKKLKWSCEDMDSEERRMLLDVAYFLVGEDKELAVQVLEGLDYMLDVHYSLKRLCET

YLISFKNDVDYHHDETKREQLRKRQIPPKFSDWE

>AB_004268_T.1

MLNEHEGRMKAKVVGIVGFGRVGKTTLAKEFFNRKRSHYRESCFLFDVREMSARMALSYL

QNKLLKDLKHTNNIKIESPDQGIGILNRYFSSCPALIILDDVDNASQLEMLLPVKILLAL

KARF

>AB_004269_T.1

MNLKSCEALIILDDVDHRNQLNAFLPVKHVLHPDSLILVTSRDKQVLLSEGIGESSIYQL

RGLDPPNSLKLFCWHAFFQPHPLPQFVDLVDGFVEGCDGLPLSLEVFGAMLCGENEKSNW

EERLTGLQELPREIQARLRISYDSLETEEQQIFLDIACFSIGQDKNKWIRIWSGSGWKGS

IGLR

>AB_004349_T.1

MFEGDESIDDRKMKLYASLENKRFLLILDDMWSPIVDLDQVGVNFGHDNRSRVLISSRFR

DVVETFAVKKYCMMIRPLSSEEGWELFSRRAFTNGALIPEHIAKEVASECQGLPLAINVV

AAAMARKETADEWSRALDLMRNVDPSFPSTHRTIDKELYQRLRLSYNLKMCFLYCAAFPE

DDWIHVETLVEMWTAESLVPQKGRTYFMDVGREYIDALVHRCFIEYDDRCAEKEFIKVDD

RYDEKGFIKVHDVLRDMGIYIGYF

>AB_004696_T.1

MAGVSTGTGCAANVGSDVMSGLWNKIVQVVQMIRDLPRAVRDMNVQVQRLKYVRDDIKAV

LAQQQRNPKQVVQNWLMKAGEAIESSETISNEYEEHKNCLGCCPDCVLRYRISKDIRSWK

STVDQLHLEKQSDFPSSGEYGDPTPCTQIPIQSTESGFASNAMLSAQTKLVETWLTDKES

DIRRIGIQGMGGVGKTLLLDTINDSQKVRNSFELIIKVTVSKNHILDMQDCVADRLNIRR

EFPDKSNFEGRKDRLRSYLKNKKYLILLDDMWVEDMWRSDQLQNLGVSLNDTGFKIVLTT

RDKQVCARMNVQETITVEPLLEDDGWELFRSRAFENRNGVVPQEIEEAARKIAKECKGLP

LAIIVVAAAMTHHTDVHDWEVALDQMRNVDETFYDLHPGVDTELFQRLKWSYDALKSDNL

KVCFLYFAAYPEDRQIHCGEVIDMWIAEGVVKGSGNSYLNDIAYSFISHLRDRCLIEVVH

KDEVGRIITVKMHDVLRDLAIRIAEKDHMCYFKAGQGVSHFPVQEEVHAKGCARLSLMYN

RLDSLPTTFPCSSISVLLLGENRGIKEVPGSFLRELPSLKVLDLSKTGITSLPPSIGDLK

HLASLQLRQTWICELPETIGDLKELQFLNVHGCEKLRCLPERISELKHLRALNISYCLGL

RQLPRGISELTSLERLEMWCSHISLHFEEDGNEERKYGCLKDLQSLRRLRDLSVRMKSPV

KEGVIRNWSKMRTLWLEFDGGVNQDYLPQDMQAMKDLECFTLYGCDVERLPTWVSEFRRL

GYLRLYWCNKLRELELGIGFPKLGTLMLDRLQSLECVGAVAARASGSGGLGEGASPLPPM

LKSLEVFRCNKLKRLRGEWDKLKCLEKIKGEREWWDAIEWEDTNIKTSLESKFNSR

>AB_004714_T.1

MDIFNCKNACSRLDGLLTGLIQALQQIRLFNLDAPQNEQNPGDEKFLTTLMKGVELVKEC

EKASHFTVSRNLTYASRIHQLEKEVCGFVQYQMPPHILLDVNNLITELKSFHHLYELGSE

DESEMNETIFKYAAKVTNDPHEKAMMLQQMVADEMFDGAFDEAPCSYNLSVKSDFVPGLE

KNIWNLKKILLQREVSVVGVQGMGGLGKTTLAL

>AB_005234_T.1

MNPEGVKIWTNQSMQAKRFALFLDDVWEEGAKLLEELGIPLLTNPSNSKVIVSSRDYRVL

REMGVNADRSTKEMEDLIEDESWRLFAHHAFPYNDRNTPASIEERTARLVCAKCGGLPLA

IKAVGRAMAGITDPKEWEFAVGRLPETNSQGLRQALYDPLRWSYDALGRYDINLQLCFLY

LSVFTEDQVIWIEHAIRVFIGEGLLARKNHNPFEVGRFYINFLADRCLIEPTIRDVDGSV

VLFRVHDILRELAIQIAEREEGFYCRSGKGLTELTENENERPARTRLFLSFNNLSSLPES

LRAPEISSLLLVGNPIREIPRKVLGSMVSLKVLDLSFTSLQSLPESVGRLKDLVCLFLVK

VPITRLPGSLTDLPCLEVLDVSLTKITELPSDIHRLRSLRYLGLQCCEDLQSLPRSMSLL

TSLRYLKMCGCSSLWTKCAGKTSHKKVASINDLPSLTQLKKLHVDNNEEIISEGTFGRME

ELEALQLTCTAMEILPDDIIKMSKLRILWLECPSVVKIESKFSEFLCLTNLKLYRCKMLE

ELPDLHKIIHLKKLEINACPKLKKFPKKFGEKGAFPSLKILSLVDLSGLEELPIIEEDAM

FSLTTFTMLSCEALKMLSSSYLNIKSLKKVRVWGCSMVLENLEKVERTNTIVEVVTMTTT

EVINILEKKRFGQDHYLYGEFW

>AB_005266_T.1

MASSSEIEIENQPSPATPHASSASSSAAADPTTSSVFINHRGPDTKKTFASHLYHRLLSD

GFRPFLDHQELQEGLDFPSQIEHAIRSASVHVAVFSPKYADSDWCLRELLLMLESGARII

PVFYHVQPADLRWTGEDKTGVYAKA

>AB_005318_T.1

MGGSGKTTLALALCNDPQVKDFFQNNIIFITVAHSPNVKGLLETLWDKIIGGRRPDFQSI

EDAHNQLKNGIRLKGHPKTLVVLDDVWSRSNVEQLLFEAEGYKTIITTRQDYTIPTSNST

RVYNIPMLQKADALSLFCFWAFGQPSIPITEDEDLVKMVEAECKGLPLALKVIASSLRGE

PRPVWENAKKRLSRGQSISEYHRDELFHCLETSIDVLDEESKQCFLDLGAFPKGRKFSAD

ALLDIWVYTRGMEWQDAFVVLLELASRSLLNLTSDPGCRAISFGCASELSFSQHDVMRDL

AFRLANQDSTIHCKRLFMPSKEDNIPTKWLTLKDQASKAQFVSIHTGPMEEQNWCQINFP

EVEVLTLFFAASRYCLPTFLHTMPKLKVAIIYNYSSKRSTLHGLPSFPAFPQIKSVLLGR

LIVSPLYEYCRSSESLEKLSVCLCEGLGNMTSLDKGHDFPESPKFQEINFDHCSDLEELP

ENICNLTYLQKLSVTNCHLIQKLPDNLGRLRSLRVLRLSACPSLSMLPPSICALQQLEFL

DISLCRSLKDLPMEFDQLSNLKILDMRECSGLKKLPKVLAKLRSLRCVICDESTERQWLA

IKASTMPNLVVEVIEERFNLDWLDD

>AB_006721_T.1

MPMETMQSLPSNMSLSIQLLISLSEKARHNRSNCNNLVGQLKFLKPLFEDIEKSNITMTP

PVLRVLEALDSNLNKAKELVERCGPKGSKIYMVLCSGQFQPKFDSISLKISHALSTLPFS

SLHVSEQTQAQVEHCIKELKRVKYTREPYYEQTAADLEKALKDHRDGVKVDNDKLRTIAE

GLELTSNQEVLREASYLEKEKDYARAEKDKQEEDCINQIIGLVTQMCDYMVELKQSQTEA

GVQIPADFRCPLSLELMSDPVIVASGQTYERGYIQQWLEQGMITCPKTRQTLSHRNLIPN

YTVKALIANWCESNNVPLPEPAKPVTYTSQRNQSTKIDSDDGMQPSSLGLVSANSSLVNF

SASQTAFTQKEICSEDHGSKKESLGFSPRSDLEGGESSTQRDPLINGEHQIQAVEENSIT

NRVSNQTEGHSRNVSLSSIASSLDDGQTSAGGDAPISEGNSNTALSDGSPYSSDVSGELA

APSSATHSQRSDIGPSRLPERVFHGGRLSNLWGRRTDHRSTMPLVISSQVSDTSANESSI

RQQVERLVEDLLSGSVEIQRAAAGELRILAKCNMENRIIIANCGATRPLVALLSSPDFET

QEVAVTALLNLSINDNNKNEIAAAGAIDPLINVLTIGNSEAKENAAATLFSLSVMEENKI

AIGQSGAIPPLVDLLMNGTPRGKKDAATALFNLSILHENKARIVRAGAVKHLISLMEPAA

GMVDKAVAVLANLATIQEGRAAIGEERGIPALVEVVEIGSQRGKENAAAALLQLCINSSK

FRATVLQEGAIPPLVALSQSGTPRAKEKAQALLRHFRDQRHASMGRGGADRHR

>AB_007534_T.1

MMLQQMGPDNMFDGAYEEAPPCSYNGSFKSDSVMGLEKNISNLKRILFQREVSVVGVQGM

GGVGKTTMALALSEDQEIKVAFRNNIIFLTVSQSPNLKVILETMWEKIVRRKKPEFQNVE

DAHRQLQQQLRRQARPTLVVLDDVWSRANLENLLFEGEGYKTIVTTRDRSTIPTTTSTRI

YELPLLGEADALSLFCFWAFGQKSIPSNADEHLVKQVQAKCKGLPLALKVIGSSLHGEPR

PAWESAKNKLLNGQSISDYHKDGLVKCLETSIDVLDEEARECFLDLGSFPEDRKISVDAL

LDIWVYVRKMDWHDAFVILLELASRNMLNLTSNLRGQAINYGSASEIYFSQHDVMRDLAI

YLASRDRIVNRKRLLMPIKEDSLPRKWELFKDQAFDAQIVSIH

>AB_007857_T.1

MGGSGKTTLAIAVCNDPQVKDFFQNNVVFITVSQSPNVKAILETMWDKLIGAGRPVFQSI

EDAHNQLQKNLSLKGSRPTLVVLDDVWSRSNVEQLLFEADGFKTVITTRQASAIPNTNSR

RVYSMPMLQKADALSLFCFWAFGQPSIPATEDEDIVKQVEAVCKGLPLALKVIGSSLRNE

PKPVWENAKKKLSRAEPISQYHTEELLNCLETSVDVLDDESRECFLDLGAFPKGRKFSVD

SLLDIWVHVRGMEWEDAFVILLELASRNLLNLKSDPGSLAISFGCACELSFSQHDVMRDL

ALRLATKDSTNRCKRLFMAGKEDNIPTNWLTLKDHTSRVQFLSIHTGSMEEQDWHQINFP

EVEALALFFSASQYCLPTFLHTMPKLKVLIIYNHSSKRAILHGLPGFSSVPQIKSLLLEK

LIVPPLYEYGKSWESLEKLSVTLCEGLGNMTLFDKAQVLMFPKLIEINFDHCSDLEELPG

KICSLTSLERWSVTNCHLIEKLPDDLGSLSSLRLLRLSACPSLSLLPPSISKLQQLEFLD

ISLCRSLKDLPVELGQLSNLKMLDMRECSGLKMLPKALAKLRSLKRVICDEHTEQQWLAI

KAKAIPNLTVDVVEERFSLDWLDD

>AB_008861_T.1

MLDKLVVHVVKMAKKTPLHVAKYPTGLDEKVEDFEAKVLLRHKRTSKVQMVGIVGLGGVG

KTTLAIEIFNKKHSEYNKSYFLSDVREKAKSSLHTLQSELLKGLNLFNGPIGNKHQGTEM

LKKSLSSSQVFVILDDIDHRDQVDALLPDRDGLTNDSLILITSRDRGVLEKSGVEESSIY

ELKGLDSKHSQELFCSYAFCQPYPLRGFEDLVNMFLRTCDGLPLSLKVFGSLLFGEKEKS

HWKDILDRVHEVPDDILDRLRISYDALSGEERQIFLDIACFFIGKNKNTAITLWDGSGWK

GSLGFRNLQGKCLVEVDSKNVIHMHDHLRDMGRQIAKDESPRRLWHPTENIDDWLQQQSS

VISEARGISMDGNNEVANVLIRSPMFSWFQRKIDKVFGNRMKKRPGISAMKLQLLSSEGH

LLERILRRVHSPNLIWLSWRYCPYSYLPSWIPMKNLRVLRVQGGVLNTLLHLSTFVDYS

>AB_009102_T.1

MMLSYLGSFGVENHILERMDMFNCRKLYSRFDGLVEGLIPKVQELFLLEQRKPRKQQNPI

YEQFLNILNNGVALVKKCEKTSRFNVFQKLRYASQIHQLEKEIQNLVQYQMPVQLSLDVT

NLVTEIKRIRQLSEQSMDERMVNEAIVAKLTNDPLKNAMMLQQMSSDDMLDGDLEEAPAC

SYNGSVKSDFIVGLEENIWNLKRILLRRDVSVLGVQGMGGVGKTTMAMALSDDQEIKGAF

GNNIIFITVSQSPNLKVILETMWEKIVRRKKPVFQSVEDAHRQLQQQLLRQAKPTLVVLD

DVWSRANLENLIFEGEGYKTLVTTRISSIIPTATSTQIYELPVLDDANAQSLFCFWAFGQ

KSIPTDADEHLVKQVQAECKGLPLALKVIGSSLRGESRPVWESAKNKLLNGESISDYHKE

GLLRCLESSIDVLDEEVRECFLDLGSFPEDRKISVDALLDIWVYVRKMEWHDAFVILLEL

ASRNLLNLTSNLRSCAINYGSTSELYFSQHDVMRDLALYLASRDWIVNRKRLFMPKKEDG

LPGKWELLKDQAFNAQVVSIHTGAMEEDQWCEMNFRQAEALILNFSAGSYFLPSFLSSMT

KLKVLIVLNYGSKRATVNGLPSPSSLPQLKTIRLERLNVPSLQEQSKLFQNLEKLSLSLC

EGLGNMSRFNTTQSSLKLPIMLDFNLDHCCDLDELPSDICDMSSAVNWSITNCHLLQKLP

DDMEKLSSLRMLRLSACLGLKELPASIGKLGKLEYLDISLCECLKELPEEIGQLKKLQVL

DMRECSRLRKLPKSVEGMKSLRHVICDEKIGQQWLRVKSSVLNELKVEIVDAHFSLDWLD

G

>AB_009316_T.1

MYLYQMTPASPLPSQDEDSTSEDQAGSSQLQTSTEGPSLFSEEQPAPLESGGLASASNNK

IDVIEWMSAMGRKLFPCLSVVEEENVFKVAGVNDMKGLIEDISKYLDLIRQGEPTTGHDG

HDRNLPESLARLLTFLSTGRKDMTVASNFRNFSEAITVVKELLEVIGKASYVTAGFLVVA

YGLKRFQDVSDNKEECWSILEEMNNLNRLVKQCTERDKLKEGMQYEIEAATELIMDGSLK

CLTQIRSSPFSKLCHTSRNQAELSNIKEELKNKQKNIYLKLQISIVDTVVALSKKPRLSR

EYPKHAVGIEEPIKEVIDLLELGREKTAVAVILYSFGGMGKTTLADAVFNKVEATDWKYS

HVELFKSIESTPDVINLQKKILEDLGASEEIRKYQDGQQKLEKVLQNVSAFIYIDNVLDK

DVLRRLLPINFDGAKNVRLLITARDINVRKVCQMKTQAYRMRGTPYAAAKSMLLKGLEKG

YSSSAARKSLFENSKSDNVNRIIDKCGAIPLMLNLVAQELSSVEDEQGVREVIEELEKWE

GEEFGRNLESYFFFYDKLPDDCKEPFLDICSFFQGWDWDAVADIVGKDVLEWLERRALVV

KGTNGVVTVHDVILEVGRRKAKGTSVRFAATSELMSFLGVDQKVEQLRYLRYKPAMDLNQ

LIKGNLKMPPNVKYMEIDGKLHCDNLEIFPTRLLRLQDLRILKLINFQGLKELPSALADL

VKGLRELTLSNCKSFKELPYSISKLKSLRVLRMQHCACVQKLPNDLGNLVSLVELDLSNC

ESLNVLPGSFRELVSLRVLNLSSCKALEDLPDGLGNITSLHQLDLSHCHKLTKIPKSIGL

LKKLRSIDISWCSNLTGLPIEFCDLSITYLKLTRCGRLKNLPNEFGNLQSLRTLKLKSCK

GLEELPESFSKLESLVHLDLSKCSGLEELSKDFYRLLSLRTLSLDGCEKLERLPEKFHLL

SSLQHLDLSNCEMLEGEVMHNVLKSESLEMVYITNSDNLKKRWVEEAGRYPSWAFKVDAG

QDLLEEEKENEWKNMSSNFDRLLQNGRGEQFQLSKLPPNTILLVMFDGQFDFNQDFPWPL

IGETIEDIEINFEIVYIGKHFNKLPKTVANKITGRALDNTDARLLIEKLFFTLKRNGVNL

WHQKMNKFFSMSAKIVGEENGGKYPSCWRLLSNSQMEEFVLSKCGRYIWMKQLAAKTQES

NIQLLRELFVHAGEDYRLYRKSVEVEIEELKDKTILLEIRPANVSFDIERLQYLKDLYES

RRSDIEIISIPIGQLPVKFEQFSADIPWLVLQNSWQMTRAVKYFLIDECQLEKGHWESSE

SWYPGRVRKIEPNGKIATYEPVLSMMQRWELEVYPFTTEIINQLKKEEWNNIQRKSMSSL

KFLFKPFHGELDKVKQKKMIGLLCDGKDPRANRMRIKTFAGLIKGAMKDVIDSIHFIYVP

HYDEKKKTVETVMDAISMAYFPMQDSKITELGLQHFSSLSQWAAMRFWMRVCDLRREIQG

MDENGKQLDGLRELLNSLDLRYLSLAFIDEDGEVLTTKGREIVGIFEEARGPGEDEAKKL

VADMIKGSPEERKEALRELESRLT

>AB_009675_T.1

MADLLTGTAGNTISSAIIVPLIQRLIEQIDDLRRLDENRELLKEQLDRMRDLLLDIKTQF

QDQQREVPVSLKRCFERIQSKVERGTELIRRSQRPWPQQCIDNLLCKPKVYTEVREWRTS

FQQNFGDLQTDISLIHHTQQIVTAAPQQEDVLLQDQPDTGLIGMEINAAETQLHSWVSEA

PDVRIIAVYGMGGVGKTTLLKKVYNNFKVGRIFHHVIWVTVAQFPVLQMQNDIASAINLD

LAIYSADMRKIKLSAYLKNTKFFLVLDDMWSAFDLKELGVEFGENKGSKVVFTTRNRDLI

REMNAKLSMQIQPLLPEEGWELFRKVAFEDDHVPENIENIARQVAKECQGLPLAIKVIAS

TMLGSTDVDEWKFALKQMQKVDPNFPLTHPRIDRDLYQRLKYSYDCLPDANMKNCFLYC

>AB_010011_T.1

MAESTSSSTATELENNGYSHDVFINHRGPDVKKTFASHLYRRLIPYGLRVFLDYQELQVA

ENLTPQLKRAIASASVHVAIFSPDYAGSNWCLDELLLILKSGAPIIPVFYGVRPADLRWT

NEQSEGVYGRALDNLAKKRTFGCQQPRYSSVTIENWRNALSRVADISGLNLEAFNSDEGE

LLDKVVECVLKRVKKPQLYVAKYPTGLDEKAQDFESTVLSQQHSGRVQVVGIAGLGGVGK

TTLAKELFNRKSSNYTKSYFLFDVRENAKSSLHSLQRKLLNGLIQLNLGIDSVDEGKGLL

RKYLSYSQVLIVLDDVDHVDQVDALLPVDVLHPDSLIFITSRDKNVLLRSGIQESSIYQL

TGLDAQHSTELFCSYAFCQPHPLSGFEYMVDQFLRACNGLPLSLKVFGGLLYGNNDNSYW

EDQLDTLQQTLPHDIQKSLQISYDALHKDEKQIFLDIACFFIGERRDTAIKIWDGSGWKG

SLGFQSLQRKCLVEVDMENRIIMHDHLRDLGRDLAKESRLPRRIWRGIENIEEFFQQSYG

ITDVRGIRLFRSEYKDDDDEYSFFSCYKIRKLELLDVDILSEHILSGVQSPKLIWLRWHE

CHLSFLPSWIPMKNLWVLEVHGEELKTLWQAESQAPLQLRELQIFAPLSKIPKSIGKLKH

LERIVVDCPSSQNNFDFEELPEEFGHLRSLKALVLKRCSKMRSLPDSFGNLTNLEHIDLS

YCMDLERLPNSFGNLIRLRYLDLSCCFDLTISSETLGNISTLRYIDLSYCNKIEVLPSQV

AHQRGLEKLNLKLINLKELPSDIGELSALEALELESPLLETLPASIGDLRSLKYLVLRRC

EKLKRLADSIGLLNQLTMLTVGDCSLQELPFKTVEAESETFVESRGRRRCSDLDSFVGKC

MLRLQHLELYNTQILDEVSFGEGVCPNLQHLLIKSCNDLVVVGTLPNALVKLELNYCCKL

SKIEGLGGLPKLQTLIIKGCKEVEELPSLETLASLEDFDASVSLKLKSIRGVEQLTKLQK

LEVSNCSELEELPDVENLRSLEKLDVSQCVKLQSIEGLVQLTKLRTLKVSDCSELKELAG

VEHLKSLEVLEAFNCVKLKNILGLEQLTKLRKLDVSNCSELEEMPGAENLRSLKKLEIFG

CVKLQGIQGLAQLTKVDRPGLGQLTKLRRRALTQLPKLHRLDVSDCFD

>AB_010936_T.1

MATDIGTGLATNATNATNAIVGLLFQQLKDVQDLGENVQLLQNELARMDESLRHIHTQFE

AQQKKLPEMVEMCLDQRMKGALEYAKALISRANRQRERCLGCCLVSCSKIPAEIRGWKTR

FNELFRELESALSIIANTKEIVESAQPQAKVLLQPLPESGLVGSGIAESAEKVLTWLKEP

HPQARVIGVYGMAGVGKTSLLKLIYNKCKVSSDFDYVIWFTVSQNYNVGSLQDCIAQSLG

LNVEGISSIEMRTMKLYESLKSKRFLLILDDLWSPVDLNQVGVNFGHDNRSRVLISSRSR

EVVEIMAANAGYCMKIQPLSIEEGWELFRRRAFTNGAAGDKNVEGIAEKVALECKGLPLA

INTVAAALARKKTADEWNRALELMKNADPSFPSTHNTIDKELYQPLRWSYNDLPDHNLKM

CFLYCAVFPEDARINVETLVEMWTAESLVPQKGRTYFMDVGREYIDALVDRCLIEYVDGY

NKEEIKVHDVLRDMAIYIGQRDHKWLFSASQHLKKFPSEEQTSDRNRISVFDNGISDLPA

DLKSAPTLVSLVLAQNEDLKEIPESFLVNLKSLKVLDLSHTSIQTLPTSVGQLGQLVFLN

LSYCERLKDLPDDICNLSHLQFLNLKQCYSLKSLPARIGALKNLKHLQFSSYIGERVEVI

SHQISQLTSLNKLILPDGSNPMSVEHLTNLSSLIELTVRVKPKMKGGSMSSWLEMRELTL

SFCPEEEDGDDAAVDFVPQIMESMKKLQTLSLCGYEGVSLPDCIYQFQNLKNLDLSRCSA

LKELPAMEMGSDAASGGYPMLERLHLWSLDSLESIVRDEGTMLKLQSVSIASCP

>AB_011051_T.1

MDRFNCKNTCCRLEGLITGLIDIFQQLCVSNLRKPKNQQDFMYEQFLKTLMKGVELLKKW

ENSSTLKFFHSLRYGFQIRQLEKEISDFLQHQMPVKILIEVKNLVAELNNLRQLYELGSM

DESKMNETIFKHVSKLTNDPQQNAMMLQQMGADDMFDEAFVEVPCNYNGLGKSDFVVGLE

KHMWNLKRTLLQRDVSVVGVQGMGGLGKTTLALALSNDKEIQDVFQNSIIFITVSESPNL

KVILETMWEKLVQRKRPEFQNVEEAHRQLQQQLLRQSKPTLVVLDDVWSRANLEKLLFEG

IGYKTLVTTRDCSIIPKAISTQLYELSLLDDADALSLFCFWAFGQSSIPSTADAHLVKQV

QSQCRGLPLALKVIGSSLHGEPRPVWQSAKNKLLNGESISDYHKEGLFKCLETSIDVLDE

EARECFLDLGSFPEDRKISVDAVLDIWVYVRKLEWQDAFVILLELASRNLLNLTSNLRSQ

AINYGSASELYFSQHDVMRTLALYLASRDRIVCRKRLLMPQQEDSLPMKWKLLKDQAFDA

QIVSIHTGAMEENQWCKMNFCEAEALVLLFSASNYFLPSFLSSMRKLKVLIVINFGSKRA

TVNGLRVLTSLTHLKTIRLERLIVPPLQEHSKVLQNLEKLSLSLCEGLGKVSKFNSTESR

LKLPIMLDFNLDHCSDLEELPLGICDMPSVQKWSITNCHLLQKLPVDSGKLSSLRMLRVS

ACLGLQELPASIVKLGKLEYLDISLCECLKELPEEIGQLKKLKELDMRECSRLRKLPKSA

GGLTSLKHVMCDEKIGQQWNRVKSSSALVDLRVEVIEVHFSLDWLDG

>AB_011084_T.1

MAILQSVSTPSSGKQINGSPFTKSSTSSSQSTFYDVFVSHRGPDVKKSLASILYHSLQHM

GLRVFLDSGEFELGDYFPDKIERAICSASVHIVILSENYAHSPWCLTELAIMLGTDAKIV

PVFYHVPPSVPRHCTGAYADAFRQHELKGRYREEEINKWKEALHKASLHSGWEFNEHNDD

QGKLWKNIVDTVLTEVRRRSSLEVAEHPVGLEEAVDDFNKRMSEFAQEREKNPNLVGIVG

MGGSGKTSLARHLFNINHSAFHASCFLFDVREASANNKLPTLQRKLLKELLHVDYEIDSV

SQGKEFLRSGFATAETYRFLIILDDIDHTDQVDALLAKNVLRSDSLIIITSRDRSLFRRN

PEAMLYEMKSMDENHARELFCLHAFRLGSPVSGFENLVEGFLKACDGLPLSLKVLGGHLF

GISDKKEWALELQNLSKVPNKDIKHKLQISYEALERDEKKMFLDIACFFIGYHKNTAIQI

WNASGWSGEKSIQTLKYKSLIEVHNGGTLRMHDLLRDLGRDMAAAEENVSRRLWKLQDIN

PLQRKGFRRILSSSHSFRSFLHLRCPHEKPQISYKNSRELTKNSSAKDHSSPLGEFSRVN

FFLGNDCLQPEVLKTSTDLLWLKWENCSHKSIPSWIPMKSLRHLEIIQGELKELWQGNLE

APLQLRELIIRWNPLTSLPMRFGSLTNLEHIDFNGCNRLEMLPKLFNSLTRLQYLDLSDC

ANLRIEPCNLSFGQTKSTYQDSLRTLRLLGTNVSKEVVNQIVKLNSLECLTIGSDSLTSL

PSSLENLKRLSVLCIGGCKRMRFLPASVRKLSLLTNLTIRTSGVEYLPEGIGELTNLQAL

EVEYCPITELTFTNSKAERFEGAEEEIPSNGAVNNLPSSIQDSMCMLSLQNITLRNTKVR

KISISEDFCPTLKTLNLGENTDLVEVDLTLPSKLECLCLSGCGRLKRISGISDLEKLKEL

GISGCCELEELPSLARLSALERFTADKCWKLQNTGGVEQLEGLKDFRLLADNGAVWNCFH

DLQRIPSHQMILGGRAGAVAGEAGVESINLSASDFCDGTAMDSFTVIQQDCVIGGDIINV

CNSSCAASVVIVICFVIESFSENSWVRFWGFPGVLNLDGAVWIKAGEWIIASVFAIEEAS

AWMKDIVNLKRLQVLLAKSPHYKLKKAFITMVNNEGGDKITEILNKIFVSMKDTRAPEQS

GSTFKLQDGVKGRHCYR

>AB_011119_T.1

MLKEFLSSFQVLLVLDDIDHEDQVDKLLPERDFYCSNSLILITSRDGNVLKRSRLDESSI

YKLTGLSAQHSQELFCSYAFCQPYPLEGFEDLVDKFLMACDGLPLSLKVFGGLLFGKNDK

SSWEDTLNQVHEVPNDILERLRISYDALHEEEQQIFLDIACFFIGEKRDMTIKVWDGSGW

KGLLRFQNLQSKCLVD

>AB_011134_T.1

MVILDDVDDVEQIEALLPPQTVLPSNSLILITSRNKDVLTSARVEESSIYQLTGLSTEHS

RQLFCSHSFNQRHPRAGFENIVEEFLKACDGLPLSLIVIGALLCGQKMAYWTEQLVILER

ELPEKINKRLRISYEALNNEEKNIFLDIACFFTRQNRDTAIRIWDGVGHGGWLGFEKLRS

RCLVTVNSENGIHMHAHLRDMGRDVAAESLSPLSPLRVWRWKNEDINLLLQPSGIFVRGI

KLDDSDCINLDDDMDCEISIKRLQLLDTEGGILERVMKWAKRPNLIWLRWKNCPHSSLPS

GISMENLRVLQVNGRRLKTLWQEVEAPMKLRELEIYAPLRNIPKSIGRLKDLERIVVGRF

LQGRVDLTELPEEFGDLQSLKALVLKECSKIRSLPDSFCKLTALEHIDLSFCRNLERLPN

SFGNLGNLQHMDLSNCYDLEMLPDTFGSLRSLQHVDLRGCHSLRRLPDSFGDLSNLQHIN

LSNCNGLKSLPDSFCGLSKLQDIDLSGCHNLKSLPDSFGNLTYLQHVNLSNCHDLETLPD

SLDNLRNLKCIDLSRCHNLERLPNALRDLSPPNICLEGCSYLIN

>AB_011720_T.1

MNMDPVSRSIIETAVGEWFIVTRIVTNTIISNGSSSKELREKLLGLKPNIDEISRLSSDS

DPSSLRGQRFKDFQAQLQDGLHLAKKLERMNSFNLYRRYRYGKRILKVEKNVNDFLLTHG

LATSLVLDVHKLNVDSKGCDERLKCVEEMGRQIIDSVNAKLTNDPSSNSLMLHQMNTTQL

FETSVDESNDTIMVDLSTSSSHPQVPDMPNFVVGLNNLVNDVKHILIFQDGVSIVGVKGM

GGSGKTTLALALCNDPQVKDFFHNNIIFIAVTQSPNLKGLLEIMWDKIIGGIRPAFQSIE

DAHNQLQTKLSLKRYQKTLVVLDDVWSRSNVEDLLFKADGYKTIITTRQDYTIPVSNCTR

EYNIPMLQKTDALSLFCFWAFGRPSIPTTEDEALVKMVEAECKGLPLALKIIGTSLRGEP

QPVWENAKKKLSRAESISEYHRDNLLHCLETSVDVLDDESKQCFLDLGAFPKGRKFS

>AB_012061_T.1

MEPVSAAYHVFINHRGPDVKKTLASLIYHRLTTCHGLRVFLDQKELHTGDGLSPAIRGAI

ESASVNVAIFSERYAESRWCLDELCWILRCNTKIIPVFCDVEPTDLRYIDSESGRYREAF

EEHQRKGRVTMEDIQKWKTALGTASEISGVPFKTNESDFGEVVEKIVKAVLKGVKWEPLE

VAKYLVGLDPAVKEITDEIKKQGELNGTAIVGIAGMAGVGKSTLAAYLYNMKRSDFSRSC

FLSEVRKKDLTSLQRKLLRDLLGCDFDIEDTRQGKGIFRDRLRGLQVLIVLDDADHIEQI

NSLLVTDVLGSRSLILITSRDQDLLTRFSQKTVVYKVKPLQGKYAHELFCWHAFHQSQPY

EGFEHLVEEALKICGGLPLTLQVLGGQFLGRHEKQFWEQQLEMFPRNPPRDIINTLTVSY

EALSREEQEIFLDIGCFFVGEEKEVAVGVIKGLGYNYNDICACFENLRHKCLVEYHDEAQ

CDDYENKSRSEEPSKNLSVCDSDSNGNYDYLNLPKRNKITMHDQIRHLARRIAREEFGVL

PKPTPARLSCSSDIEELMLKLQGGADSCLIRGIRTPKGQNPPDCTNYIKGVGVRLFAVEF

PIDLSSLFGLWDISGDLVWLRLRSF

>AB_012179_T.1

MERIQLEVMEKLPIMFSPPLASDGNRILHTFGFNCKELSSRLEARLACLIQAVLSLCSSD

LNCKNEQNPIYQQFMSILMDGVELVKNCEKTSRYNIYQNLRYASKLQQLEKEIRDFVQYR

LPVQLFVDVNDLIIEMKSLRHLYESPSVDERKVNETIVPKLTNDPFENATMLQHMGADGM

FDGAFDEAPPCRYDGFVNTDFVVGMEKNICNLKEILLQREVSVVGVQGMGGVGKTTMALA

LCNDRDIKGAFRNNIIFLTVSQSPNLKGILESMWEKIVPRKKPEFQNVEDAHRQLQQQLL

RQAKPTLVVLDDVWSRANLENLLFDGEGYKTLVTTRDRSIIPKSTSTQLYELPLLDDADA

LSLFCFWAF

>AB_012418_T.1

MNQIDALLPTRTDLISDSLLVITSRDKEVIKSSGVENAAIYKLTGLNEQHSRELFCAYAF

NQLYPRPSFESLVDNFSKACDGSPLLLKVFGAHVNGKKNQFEWKNLLDRLQQFPPSEIQE

RFQISYQSLDEEGQQIFLDTACFFNGQKRDTAIRIWDGSGWKGRRGFENLQNKCLVDVDR

ENNIVMHDHLRDFGRHVAKNSLPRRLWHCTTINDMLEQLSVATVRGIRMVLNEDSDNDDA

FGGIDMSTLQLVDTENAGFLERVLTRPKHLRPSLIWLRWNNCDTRSLPSWIPMKSLRVLQ

VSGSKLNTLWEDESQAPLELQELEINAPLSNIPKSIERLRDLEHMVIGRFLSGQVNLTKL

PDEICYLSSLKTLVLTECSKMKFLPNSFGRLGNLQHVDLSFCRNLETLPDSFCDLLRLRH

INLSDCHDLERLPDSIGRLSSLQHIDLRGCHNLERLPEFFGNLRDLRHINLSVCHDLKRL

PNSFGNLKYLQHIDLQGCHKLENLPKSFGNLMNLQHINLSNCHGLEKLPDSFGDLSNLQY

MDLSGCHNLER

>AB_012547_T.1

MFLWSWKTVFADKCEGLPLAIATAMKGKSSVDEWESSLSLMKTSDPSFPHTHPPVDRELY

QRLRWSYDALPHSNAKNCFFFCAIYPGDADLMWTNQYKCRLPKGSLGARKKSI

>AB_013055_T.1

MTASSSTSANLHINSEYDVFINHRGPDVKKTFASHLYRALRSFGLKVFLDQQELQQGDYF

APQIEEAIRTSSVHVAIFSKGYADSIWCLNELLLMLESKAPIIPVFYQVKPAELRWTHGQ

HGVYAQALRKLEEKTTYDPQTQEEKPRYSSTDIENWTKALSNVAGISGFEALEACNDDEG

ELKDQVVESVLRHVKKTPLNVAKYPTGLDEKVTHFENEVLLQHRQSGRKPQVVGIVGLGG

AGKTTLAKELYNRKRSDYKRDCFLFDVRENVGNGSLILLQKNLLKCLTGSDLHINNKHGG

IEELKKHLSSSPILLILDDVDSVDEVDALVPNKTVIHSDSLVLITSRSVDVLTNSGVEKS

SIYKLNGLNKQHSLELFCWHAFNQRDPLPGFHDLADQFLKACHGLPLSLKVLGALLCGNE

DTPYWYEQLDRLQNILPSEIRKSLLISYEALNEEEKHIFLDISCFFIGHHRDKAITIWKG

SGWKGWLGFQSLQNKCLVEVDSWNVIQMHDHLKDMGRDVAKGLSPHRLWRWTDEVIDNLL

GQSSAIIVRGIRMLPRDCNDNDVFSAIRMMRLQLLHTDGGLLDRILKRVELPNLIWLRWT

CCPYSSL

>AB_013147_T.1

MDPVSQSIIGSAACELFTVTRTIMNIMISSKSSGEELKQKLLRLDPTIDKISKLSSDSNP

SSHRGRPFKDFQTQLQNGLHLVKKLKRMSSFSLYRRYRYGKQILKVEKNLNDFLSVHKLD

VDFKGCSEQSERIEEMGRHIMDSVNAKMTNDAGSNSLMLRQMSTTQLFQSSIDGLHDTAM

AEQSTSSGNSQIPGMPKFVVGLNTLVNDVKHILFQNGVNIVGVKGMGGSGKTTLALALCN

DPEVKDFFQSNILFIAVSQSPNVKGLLETMWDKIVGARRPVFQSIEDAHYQLQKNLSLKG

NRPTLVILDDVWSTSNVGQLLFEAEGYKTVITTRQDYTIPSTNGSRVYNIPMLQKADALS

LFCFWAFGQPSIPTTEDEDLVKQVEAVCMGLPLALKVIGSSLHNEPQPVWENAKKKLSRA

ESISQYHREELLKCLETSIDVLDDESKECFLDLGAFPKGRKFSVDSLLDIWVYVRGMEWQ

DAFVVLLELASRNLLHLTSDPGSLAIPFGRACELSF

>AB_013167_T.1

MDSVSQSIIDKFFIVTRTILNSMVSNKSSCEELRQNLLLLKPSIDEIAKLSSESSPAPHR

GHPFKDIQAQLQDGIHLVKKLERMSSFNLYRRYRYGKKTQKIEKNVNDFLRTLDVHNPDV

DFKGFGEQSELIEEMGRLIMERVNVK

>AB_013507_T.1

MWKILKLESLGVSLKDTASKIILSTRSRVVCTQMNVDESIRVEPLSEEEGWQLFCKGAFR

SGNVPQEIEDVARKIAGECKGLPLAINVVAAAMKDDAYRYQWEVALDQMQTVNNTFYDLH

NEIEEDLFQP

>AB_013805_T.1

MNTIVSSKSSSEELKQRLLSLKPTFDEISKLASDKDSAHYRGHPFKEFQAQLQDGLQLVE

KLEQVSNFNLYRKYRYGKQILKIENNVNNFLRTQGLAYLILDVHKLNEDFKGCSERSERI

EEMGRHIIDSVNAKMTNDATFNSLMLQQMSTIQLFQSSIDGLYDTTMAEQSIASCNSLVP

GMPSFVVGLNNLVNDVRHILFQNEVSIVGVEGMGGSGKTTLAIAVCNDPQVKDFFQNNVV

FITVSQSPNVKAILETMWDKLIGAGRPVFQSIEDAHNQLQKNLSLKGSRPTLVVLDDVWS

RSNVEQLLFEADGFKTVITTRQASAIPNTNSRHVYSMPMLQKADALSLFCFWAFGQPSIP

ATEDEDIVKQVEAVCKGLPLALKVIGSSLRNEPKPVWENAKKKLSRAEPISQYHTEELLN

CLETSVDVLDDESRECFLDLGAFPKGRKFSVDSLLDIWVHV

>AB_013978_T.1

MLRRHLSSTKALVIIDDVDHVDQLDALLPHQTNLQSNSLVLITSRNKHVLTSRIEKRSIY

KLTGLNRQHSGELFCSHAFHRPYPIPGFESLVEKYLETCNGLPLSLKVFGALLYGEDISY

FEEQLESLEQIL

>AB_014128_T.1

MLPHDPGFVSGFIGVGINVAVDQIVHHVSIALRYNQELNSLRDLVMSIKPIISQIQQCRL

ALNRKRGIPISQSGITMKASAVNDWLKRMDSLLRQASVMAQECTIPSCDLISRYRTSRRI

TRLISVISKHLDQVSLMGWASVLEGLGQIKQSVEALAASSSSATTSATTSEHVASTGIVI

NEPLIVGQHNALERLEKLVADAETDFLGVAGMGGAGKTLVLKTLFNSQKVRDLFCDGLML

WLTVSQSPSFSSLRNELSTQIAMQTKVDPHTIMNRPLNESMQGKRFALFLDDVWENGSKL

LEDLGVLRQIHLSNSCKIIVSSRNKGALSEMGVAKDSTITMGDLNEDDTWRLFSYYAFPY

NKGNVPANVDQATAKDVCHKCGGLPLAIKAVGRAMARITDAKEWEFTVQRLPNANRKDDQ

AVYDRLRLSYDALGTYNVHLQLGFLYLAAAFLEDEVVRVDGEVTLLWMGEGLLEAGHDPF

EMGRVYANVLADRCLIEPTLRDVDGCVVNFRMHDVLRGLAIQIAEEEENFYCRAGKGFTD

KDLNINEFSPYTRIMLNDNNLSSLSESFRAPEISSLLMARNKNFTEIPKRVVGSMMSLKI

LILTRTSLRSLPESVGCLKQLVYLNLANVPIKRLPASFTNLAALQILDLEKSEITELPSD

LYRLRSLSYLNLDKCKDLKCVPSSISSLTSLQSLKIRYCSSMMWTSGKKRCQKAASINHL

GTLTQLKSLHLDNHGETINDGMVGSMVDMDTFELTLTKVESLPGGIFNMSKLRKLQLECP

DMVRMESKFCEFRKLTILRLWWCEKLEELPDLQNLKSLKQLDIFGCDKLKKLPKEYGKRG

AFPLMENFSMVELKELEELPIIEDGAMPSLKTFTIMLCEALKSFPESYLNLKQLQKLRVY

GCSMILENIESVKKTNTRVEVVTISCADTKEVRKQYLQVRNTISGWLYNEFSRSEIFLFL

GDLYRQKK

>AB_016581_T.1

MWTPLDLQELGVEFSNDRASKLIFSTRNRNLIEEMKAEECMKIEAMPTDEAWDLFSKVAF

GGGHAPNEEIENIARQVSNECKGLPLAINLIASSMIGKRAANEWEVALRQMQKLDFNNPI

AHHPRIDRGLFQLLRWSYDCLPDANLKICFLYCAMFPQETAFRVKPLVQTWMAEGLVQTK

DTGHSYVKILENRYLFQVEPQQNVITVHDAVRDMAIYIGETEEKCLFRAGQMLQQFPETE

IDCKRISILGNSIRSLPAKELRCKNLVTLYLGKLELKEIPELKEIPEAFLRGLTSLKVLD

ISGTQLKSLPLSLWELKQLEFLNLSWTEIGILHEGIGNLSSIQFLNLSHCMKLKSLPSQI

GKLKSLKYLDF

>AB_016933_T.1

MWDKIIGGRRPVFQSIEDAHNQLQKKLSSKGNLQLLVVLDDVWSKSDVEQLLFEAKGCKT

VITTRQDLTIPHTDSSHMYNMPMLQNADAMSLFCFWAFGQPSIPTTNDEVIVKQVEAVCK

GLPLALKVIGSSLRNEPQPVWEQAKKKLSSGESISKHHREKLLHCLETSIDVLDDETKEC

FLDLGAFPKGRKYCVDSLLDIWVYVRGMDWKDAFVVLLELASRNLLNLTSDSRNLAISCD

CASELFFNQHDVMHDLALRLASRDSAIDKRLFMPVKEDAIPTKWLALKHTSKVQFVSIHT

GSMEEKGWSQIDFSEVKALALFFDGSQYCLPTFLHTMPKLQVLIMYNYNSKRAELKGLSV

FSSVTELKSVFLEKLILP

>AB_017562_T.1

MDVFSSGAPSTSTAADPSSSNYDYDIFISHRGPDTKKTFASSLHRRLLKNRYRVFLDREE

MQKGETITPQIEGAIATASVHVAVLSPRYAESKWCLDELVQMLESKGTVIPVFYKVKPDE

IRRTRGTDGQYAQALKQLETKMTDGPHGETPRYDSTTVKKWRSALSEVADISGFELEECN

GGEGELLDKVVECVLQKVKTPGLNVAKYPIGLNEKVKEFEDLVLLPQQQSKKPQMVGILG

SGGVGKTTLAKEIFNRKSSYYSRSCFLFNVREEAAKGLNILQSELLTNLT

>AB_018086_T.1

MLRNHLFSSQVLLVLDDVDDVDQVDALLPHKEEYLRPDSLILITSRDKDVLARSGIQDSS

IYELTGLNPHHSKELFCQHAFKRPQPLPKFEYLVDMFIRICYGFPLFLKVFGALVYGNNE

IYWENQLNRLWCGDIKQRLKIIYEALNNEEQNMFLDIVCFFRGHGSDWTIRIMNASGWQG

SFGFQNLQEKCLVGVDRNNDIRIHDHIRDLGREIVAEGSTQFPFCFRPWPEDIESSLITE

VRGIRLFPSEFVCSKILWYRGILHLKYTLDLLERILGTVQRHSRNLVWLSWFDCPYSSLP

SWFPIKNLKVLYVGGRELKTLWQSESQAPSQLLQLEINAPIQIIPRSIGQLKHIEHIRVV

YSEAGKLLSVPMITIPEEICHLWSLKILMLRGCSKVKSLPDSLGNLTNLESLVLDFCQSL

GRLPKSFGNLIRLKHLDLDGCHSLTISTETFGNISALQYLRLSNCSKIEVLPPQIANQWC

LESLDLSGTNIKSLPSDIGKLNNLKYLTIQSCNGLVEIGTLPNALIELDLFGCLKLRKIE

ESLCSLEKLQMLNISGCVELEELPSIETLASLEKLWAFRCAKLKRIRGLASLKRLQILDV

SDCLDIEELPGVENLRWLEKLDASRCPKLQWDEGVVERLRKQLKESMLIL

>AB_018131_T.1

MKDFETTVLMRQRQRGKPQILGIVGLGGVGKTTLAKEFFNRKNSDYAQSSCFLSDVREKT

AKGSLHLLQRKLMKSLTGGTDIQIDNIDEGTGMLREHLSSSKALVVLDDVDEVQQVDALF

PVRTVPHPDSLILITSRSRDVLRGSRVEESSIYNLSGLNAEHSRELFCFHSFSQPHPLPG

FEGLVDKFLKACDGLPLSLIVFGALVYGNNEPSYWEEQLHKLQRILPAEIKQRLKISYDA

LDVEEQHIFLDIACFFIGQNRDTAIRIWEGSVWNGRLGFRNLENRCLVKVDSRNCIHMHD

HLRDLGRDIAEAPGLPLRLWRGMENILQQSSNEEVIREVRGIRMIG

>AB_018159_T.1

MFESGAPIIPVFYEVEPAELRWTRVNANGVYAQALHELEKKTTCDPQTQKEKSRHDSATI

EKWRDALSRVADMSGLSLKEFHGDEGELLDKVVENVLKRIKSAQVDVAKYPTGLYEKIKD

FETTVLLEQKVGKVQVVGITGLGGVGKTTLAKELFNRKSSIYSKTYFLSDVRDNAKISVH

LLQRKLLKGLIQLDLHIDNSHQGIEMLQKHLSSSQVLLVLDDVDHVDQVDALLPVHVLHP

DSLILITSRDKDVLTRSGVQESNIYKLTALNTQHSTELFCWHAFSLPHPLSGFECLVESF

VKACNGLPLSLKVFGGLLYRNGDKSYWEDQLDKLQRKLPSDVQRSLKISYDSLDRQEQQI

FLDIASFLTGENRDTAIRLWDGSGWSGSLGFQSLQSKRLVELESVNRIIMHDHLRDLGRE

IVEAAGLPRFVSITREGIDDLLQQSSVIAEVRGIKLLASTDKTNGGELASFSCNKRRKLE

PLHLRKLKLLDVEVGHLKQFFKKVHSPNLIWLRWEKCSYSSLPSWIPMKNLRVLEVHGDK

LETLWQSESQLPLQLRELKVGARLSEFPKCIGKLIYLEVIYLYLKGENNVKTLPEELCNL

RSLKTLVLRGWSKIKSLPDSFGNLTNLQQIDLSGSSELERLPNSFGKLTKLKDLNLYACP

NLTISTETLGSIRTLENVNLGSTEKI

>AB_018242_T.1

MAASSSTAASPSTSTSAAGNPNNKVFLNHRGVDVKKTFARSLYLRLVQNGLTAFLDNEEI

KAGYDISPQLEHAVRTASVHVAIFSPRYAESDRCLNELILMLQTNAPIIPVFYGVKPDQV

RWTRGDYAEALKAHEKKRRHNSKTIEDWRNALSKVANKSGFELETCNGA

>AB_018343_T.1

MQFDPSKVLLHELLAEARVLLARTCVRKCPNIIECCASFVKLLESFGPLYITDSNCSCEE

VNSNSASESSWVDGRGILTTADSLQMGFFQDLEILCILFELTQQIMSSLQGWSLNLEAHH

SSTGGKFDGYLVFKKRSFVSAFSVLQDNEGNAAPNTQFTKFDWRVPSSSFWMPNCNFLRK

RRHSPSSSDEALRIPIQGSEVDAAEKKKMVQEALTIGDDNLKQLRLAIMDLKGQLCRAAE

SLKAIFFSTEPGFPNFLVRDRLKRLVHSFLTVENYYVGSSDGSVDEEDPLHDYCHCHQIH

CPIGSRTGTPMAEKLVSELLDTGGSLVVGICGPRGLGKTSLASCVASNCKIKRRFIGGVY

WITAGRKTDVKSLQCCLWKAITGSQAVFTSVDEGLTALKKKFSESLLPSLLIVDDVWEAS

QVEGLLCFDGKERGRILVVTNNSQILSSADACIYTLGCLKEEDAVHLFQWVVGWDGPLTL

EQHDLASNLARKCNGSPLFIQALSSAMYGAEAHQKLVLEILLNEGGEVAGEQMNTVDQST

TNQVTGEECMDLAESSISGKQNFATTANCLYSVPYACFSALGFIHPLLQECFLDLAAFPE

GEWVSLSTLEKVWLAFSNGLSQEEIVAILSILVFRSMVDWRLNVLDNELEFKLPEPFYNL

ASNIICQDIKEYSKKKDCNVHKIQSRLLKRKKEMENIKISSKCMGVMIKFFGFGNVYMNC

TEDKELKSGELVTYSKHNDSPFRLNHLKSESTLVSEAYQGKNSLNNTDRNCQVTRFEKEC

RLFVTDYNNNFFQGTSLDETLYKATKFSIYSSNIDQLLPSLGCPFLRVGLFGGNTRLSLP

SPAVLTNMTQLLVLDLSGCTSCTHLPKAISALKSLQLLDLSFCKSLKSLPSSFGCLKNLR

FLKMSGCTSLTHLPKSTGMLTKLEVLQLSECVKLKTLPSTIGQLSLLKMLDLKGCFSLCS

LPPSAGLLRELRFLDLSKCSNLSYTLPYHSQLLLLITGSCKTENMAVPTVLWNIDQVEDL

RLAYLKNMTYLPHTVRNMQHIQKLDLSNCRRLEYLPEGIGNLWLLQELDLSHTAIRKLPD

SVGQLRNLLCLCLQGCSRLLCIPSSISSLPKLRCLDMSECWDLVLLPSSIGNSYSLCELV

ELDLTCCSFKSLPSFSLHALPKLQYLKVVGCDTLVSLPSTFEQLTSLKKFDLEGCHALTS

LKDIGFGKLDKLTWLSIRNCTSLTSLPVEEMVKSSSLKYLDVSGCTGLSPFPQALLTRAL

EGNLTLIRWGAVWPEQRNFNQQHSGAHIE

>AB_018386_T.1

MLEKPKLDVARYPTGLDEKLKHFEDTVLMQQYQIGKPQILGIWGSGGVGKTTMAKEFFNR

KKSAYRKSCFLFDVRENAAKGSLQSLQRKLLKDITGLDKTIDNVSEGTGMLKKHIEDLKS

SNEVKGMSIVSKGMSIVSNALVILDD

>AB_018416_T.1

MVKKPDLHVAKYPTGLDMKLKHFEDTVLLHQHQRGKPQIFGIVGLGGIGKTTLAKEFFNR

EKSNYHKSCFLYDVRESSLTSLQRKLLKSLTSIDVPIESVDEGIGMLIKHLDEDKMMQYK

KHPKSSNAPENVGHVDEGKSSNGL

>AB_018741_T.1

MEERVDKVIKLLKTDSEDSLITVAEDMNKVINLLKTDSEDSLITIAISGMAGVGKTSLAK

AVHNHIYHKFDATCFVYDVRHQAQHTNGIAKMQRHILKDLVKFKDKVNDEAHGKSLMRDR

LRSIRALVILEYVGDNKQLDSLRGDWYGPGSRILVITLDPQLLNDGQVDFVYEMQGLDRE

HALKPFSWHAFMRDKPVEGYEELSQRAVNICNGILL

>AB_018996_T.1

MKLSVSLEMKNILLILDDLWSPIDLDEVGVKFGDHNNSRVLISSRYRDVVEIMAENGYCM

MIQPLSTEEGWELFRRRAFRDGVVPDKSLEAIARQIALECKGLPLAITTVAAALARKKSA

DEWSRALVLMKNVDPSFPSTHRTIEAEIYQRLRWSYNDLPNNLKMCFLFCSAFPEDTEMD

VETLVEMWTAEGLVTRKGTTYFMDVGREYMDILVDRCLIEYVDLEKEEIKVPDVLRDMAI

YIGQREDNWLYEASQHPKKFPSEQEIKDCKRISLSENEISDLPLDLKCPTLVSLVLAQNR

ELKEIPERFLVNFMSLKVLDLSNTSIQALPTSVGQLGQLEFLNLKRCSQLKELPDSICNL

SSLQFLDLEDCFQLKEFPDRIGELKTLKHLNFRSKSTSQCKSFKVVSHDICQLTSLNKLV

LPKRSTAMSMEDLTNLSNLIELEVVVKPEIKKGSMSTWSAMRKLKLLFDNKDANRDEAAL

DILPQNMENMKELQSLHLSDYLGVSLPNCICQFQNLKKLCLYDCSKLKELPALEIGSEAL

SGGGFPVLERLELWLLNVESIVWNEGAMLKLQSLEISCCEDLKTLRMEKLPNLRDLNILE

CDELEEFFIGSGGFPMLETLKLNKLPNLDTVPPSMWNKGTMPKLQFLHIIECPLLTRLSL

EKFPNLEEIQICNWNCHEEVQIEIGSLPMLERLTLSGLQMLESIARPSTIWNETTMPKLQ

ILRIIDCPLLRRLPVGMDKLSNLREIVGKLDWLGRIIWEDDDIKIMLSQLFREYEDWEYY

EYIT

>AB_019200_T.1

MAVSTSTAADSNHNHCYDVFINHRGPDVKKTFASHLYRRLISYGLRVFLDYQELQEGEGL

TCQIQGAIRTASLHIAIFSPTYADSNWCLNELLLMLESGAPIIPVFHHVNPSELRWTQGK

GVYARSLHNLEKKRTYDSQSRHNSSTIEKWRNALSRVADISGLDLEAFNRDEGELVDKVV

EVVLKKSRKTKLNVARYPTGLDEKLEEFATTSLLQQHSGRVQVAGITGLCGVG

>AB_019370_T.1

MAHHITASVSASSSSSAYSASTHYDVFINHHGQDVKKTFAGHLYRSLSSRGLRVFLDQPE

LQAGHTIISQLEAAIQVACVQIAIFSPSYAESRWCLDELVLMVESEMSGATIVPVFYKVE

PSELRWTGKDKDGAYAVALRKHEERYDSHKIQSWRDALSHVAGISGFVLGACNGDEAELL

EKVVQRVLKKVPEPALHVAKYATGLREKIEDFEKIVLSQQERRVKVLGIVGSGGVGKTTL

AKEFFNCKKSNYDGCSFLGDVREETARSFLHSLQSKLIKDLKGINVEIDSCEQGLGLLEK

NLRSHHALIILDDVDDADQLDAFLPIKDVLHSRSLILVTSRNKHALISAGIAKSSIYHLK

GLDTPHSQELFCSHAFFQPYPRPGFENLVLKFVNGCGGLPLSLKVFGALLCEKDESYWEE

KLNELQKLPCKILASLKISYDSLDREEQQIFLDIACFGIGEEKDKWVRIWGGLAGLQSLE

DRCLIEIDTWKMMRMHDHLRDMGRNIAEEISMPRRLWRSIEDVQDLFEEQSSSSAIPKVR

>AB_019374_T.1

MAASSTTAGSASTSVTGNVNSKSYHVFINHRGIDVKKTFARSLYLRLLSNGLTAFLDQDE

LQQGYNFPSQIEHAVRTASVHVAVFSPRYAESYWCLNELLLMLESNAPIIPVFYRVTPAE

VRWTQEGKDGQYARALQELAKKKTYDRQTHGKKLRYDSSTIESWRNALSTVADNSGFELE

VRNGDELELELLDKLVERLLKMVEKPDLHVAKYPAGLDEKLKDFEETVLLQQQESGKPHI

VGIVGFGGVGKTTLAKEFYNRKKSGYPKSCFLHDVRDNASKGSLNSLQRKLLNSLNGSNE

SVDSVDEGKKMLINPLKSSNVLIIIDDVDN

>AB_019455_T.1

MAASSSTNSNECYQVFINHRGVDVKETFARSLYLRLRENGLRVFLDKPEMQPGYNFSSQI

EHAIRTASVHVAIFSPRYAESYWCLDELVMMSKSEAPIIPVFYRVKPADVRWTQGNKGKY

GEALKTLTAKRTHEGKPRYDSSIIDSWRNALSLAADNSGFELEVCNG

>AB_019516_T.1

MASSSTAASLTSNSYHVFINHRGPDVKKTLASHLYHRLSSAHNGLRVFFDKQELRVGEEF

PSQIKDAIRTASVHVAIFSPRYAESQWCLNELLYILDSGSPIIPVFYHVQPADLRWKQGE

DGVYAKGLKNLEPRHDTITIGKWIEALSRVSRINGLNLHETCNGDEGELVEKIVQRVLQM

VKSFFPAGKSPEFYCSQLYGGHGGSDKSDGTYSGIRRIAMTATDHDMLTSIQLHYGLEGD

ESLKDKYIQGCLHGTAAGSLKEYNLKYPEEFVTKMSGYFERYSPSPEFGVVIKSLTFHTN

KGKYGPCGLEKG

>AB_019533_T.1

MARKPDLYVADYPTGLDYKLKDFEKKILLQQQQSGKPQILGIVGLDGVGKTTLAKEFFNK

KKSAYQKSCFLYEVRDNAAKGSLHLLQSKLLKDLTGLDRTIGSVDEGKGILKNHINHFKY

SNASLHKKLHKNL

>AB_019602_T.1

MISCLGYRKLINPLLCLDPIGYGIPTISLFTQYQAEIERKPFPLNYRRAFTGASSASAST

TAYLHNTGPFHRRGPDVKNIFAIHLYRRLISHGLQIFVDSHEKQEGDRLTSQIEGAIENA

SVHIAIFFLRYAESKWCFNELLLMS

>AB_019633_T.1

MGIVGPGGVGKTTLAKQYFSQRRSHYRRSCILYDLREKDVNSCQKQRQLLKDLTGLDLQI

DSVHEGIEKLITHLSSSSSHLLIILDDVDDARQLEPFLPIKDIVDSNSLIVITSREKKVL

KKAEWTEQHIT

>AB_019684_T.1

MELELLTREDGWLLFERVAFKGSHAEGEIMECAKKIAEECQCLPLAITVVAKAMIPKKTL

AEWEESLSQMKKSYFPDIHGSVYKELYQKIGWSYKELAQTPNSKLQLCFLYCAMYPEDEK

IWVDELVRIWIGEGFIEDMQHGHSYVKLLIDRGLFQSVNPVKQGMGILVNKPVNVWKKRF

ITVHDVIRDVAINIAEKQNNYLCRAGLQLTAFPRNAQQCQRVSVRCNQIESVRKDILCIQ

LVSL

>AB_019698_T.1

MTARVEAHVASTSTSSASTAQAGAHAPTGTARTKLIDEPLIVGQDKALANLENLVIDDAE

AKNIGVLGKGGSGKTLLLKKLFNSQKVRNHFRDGLLLWLTVSQNPFLKSLRDELGTQIAI

QKNIDLGKNMDEEDVKIWLNETLLQSRCFALFLDDIWGGNAAEMLE

>AB_019699_T.1

MGVADKHTITIENLIEEDSWKLFTYHAFPYNSGIPPSSIDEEKAKVVCEKCGGLPLAIKV

VGRAMAGSTHQQQWEWAAQSLPNTDSVHDCLRLSYDALCNEDVHMQFCFLYIAATFLEDQ

IIHADWVIPLWVGERLLSRKMLQDRHQIPYDPLEMGRIYLDLLADRCLIEPIRRDVDGHV

YLFKMHDVLRDLGIRIAEEEENFYCRVGWGLTELNENECSARTRIFLTANQVSSLPKSLR

APEICSLVMYANFNLTKLPKRVTGSMISLKVLDLSCTSLQSLPESVGDLKQLVCLRLRNM

PIKRL

>AB_019712_T.1

MLHKLVERVEKMAPRALAVAQHPTGLDEKIKDFETTVLLSNQRTGKLQMVGIVGLGGVGK

TTLAKEIFNRKRSEYNKSYFLSDVRENAKSSLHTLQSKLLKGLNLFNGPIDDIHQGTEML

RKSLSSSQVFVILDDIDHEDQVVALLPDRDGLRMDSLILITSRDRGVLARSGVEESSIYE

LKGLDSKHSRELFCSYAFCQPYPLPEFEDLVKEFLRACDGLPLSLKVFGALLFGEKEKSH

WKDILDRVHEVPNDILDRLRISYDALSGEERQIFLDIACFFIGKNKNTAITLWDYSGWKG

SLGFRNLQGKCLVEVDSENVIHMHDHLRDMGRKIAKDESPLRLWHPTENIDDWLQQQSSV

ISEARRISMDGNNEVANVLIRSPMFSWSQRKIDK

>AB_019749_T.1

MLHGFGGLGKTTLADAVFSRLDIQGCKYSTVRLFENIDSTPKITKLQKLILGDLAEPGET

IPEIRKPEDGQRELGRMLEKVPTFIYIDNVLGQDELEQLLPRDINKAKKLRLFLTARDQD

IVRRALKVKPVVYAMKGLPHTEALRLLERKIYDETDDKDGQINSTKLNNIVQLYGGIPKL

LELVGAFIRVAEDKQNAYETLMKEREVCDSAIGEIEPYLFSYNHLPKRAKDPFLDICSFF

KGWDWETVADIVGQTELDMLRSRALIIKDTNGAVIVHDVILSIGLQKTSGMRFRFTEAKQ

IKELLDDR

>AB_019769_T.1

MADLQLSQAPDHTSSASTSNLASTSYANNNYYVYDVFLNHRGPDCKKTLATDLYNRLSKH

GLRVFLDQQELQEGDRLTSQIEGAIRTASVHIAIFSPRYAESSWCLKELVLMLESRATII

PVFYGVRPSELRWTRGNNGVYDRLLSMLIPWVLCLLCCTRSQNGVYARDLRLLEGKKTFD

SQTGQKKPRYDSNTIGKWRKALSAVADISGFDMETYNGDERQLIEQVVERVLKKVPRKLH

VSKYPIALDQKVEDFERDMLLQQQNGQARVIGIVGLGGIGKTTLAKEIFNRQGSNYDRSC

FLFDVRDNSLKSLQSMLLKDLTLWNGQINSTDEGIEMLRRYLSSSSKALIILDDVDHLHQ

MDALLLPAKDALPSGSLIIVTSRDKDVLISSGIVEASIFKLTGLQPQHSQELFCWHAFHQ

SHPIVGFENIVNEFLNACNGLPLSLIVIGAHLHGKIDLKYWEAQLRKISKALPNDIQKSL

KISYDGLDEEEKHIFLDIACFFIGENRDRAI

>AB_019818_T.1

MANYQTSASCFTSTSATPNSSDYNCDVFLNHRGPDVKKTFASHLYRRLLSYGFRVFLDRL

EMQAGDGLTCQLKGAIAAASVHIAIFSPRYAESKWCLEELVLMLESGTAIIPVFYHVKPS

DLRISTGSGEDGVYGRAIRDLEKKRRYEPNTVKKWREALSRVAEISGLELEACNGDEGEL

VDKVLQLVQKRARKPPLDVATYPIGLDEKLQEFERTAVLQHFGGHWVVGIVGVGGVGKTT

LAKQFFNRKRSDYDRSSFLFDVREVAARGSLTSLQSKLYEDLTGQDQVMNSIDEGISRLR

HHVRRCQALVIVDDVDSVEQLRVLFSPVKDVLSSGSLILVTSRNKDVLTVSGIKESSIYY

LAGLNREHSQELFCLHAFHQPYPVVGFDKPVREFLEVCQGLPLSLKVIGALLYSESPDFR

YWKTQLLKISKVLPTDIQSRLKISFDSLDEQEREMFLDIACFFIGEDKDKAIRVWDGSGW

EGSIGFQNLLNKYLVEVDSKNCIRMHDHLRDLGRALAKKDPRLRLWHLTDEILSNVIHQP

YVRGIRIQTSALSSD

>AB_019820_T.1

MGLPLALKVIGSSLHNEPQPVWENAKKKLSRAESISQYHREELLKCLETSIDVLDDESKE

CFLDLGAFPKGRKFSVDSLLDIWVYVRGMEWQDAFVVLLELASRNLLHLTSDPG

>AB_019826_T.1

MLKKQINNLKSSNSSLVILDDVDNANQVYALLPVDLPLGSLILITCRDKHVLRTSGVQNS

SIYRLTGLNDHYSRELFCSYAFSQTYPLQGFKSLVDSFVKTCNGLPLSLKIFGANLYGRD

IPYWEDQLRSFPSDIGDRLKISYNALKIKEKQIFLDTACFFIGQNRDTAISTWDASGWEG

RL

>AB_019833_T.1

MAASSSTVASSNNNYYYEVFINHRGPDVKKTFASHLYRRLLSHGLRIFLDQPELKRGEDL

TPQIKDAIRSASVHVAIFSAGYAESSWCLNELLQMLDSKAPIIPVFYHVKPTELRWTRAR

REAKGTKGSYAEALENLEEKTTCDPQTHEQQLRYDPSTVEKWRNALSRVAEISGLELDAC

NG

>AB_019836_T.1

MKLLPFAQNQNMTHGGNNELQSPDLTPDFVRGEILKELKTSGCLFVLDDIWNKDVFHKID

WLLGKTKIVVTTRDSRVVNTNSSYKVHKYSMKCLSDKDSMKLFCIHAFPDTADTQQQGRL

PKVLEKVADQIVQKCGQLPLALKTIGASMARVKRLPNYWESIVNSLNEEEAMSDQVMPSL

RLSYHALPYRLKLCFDYCSSFPKNSRIKSEYLVNAWIAEGYTPQTTENPYDVGLSYIDEL

IDRCLLEVSKVGGDSRVKYCKMHDILHDLALSES

>AB_020090_T.1

MFCSPLACAGDHILNQLDVFNWKKLCSKLHGLLAGFIQAVEPLCLQKPKKEQSPIYEQFL

SILNNGVELVEKCEKAGRFNVFQNIRYAYQINQLEKEISDFVRFHLPLCLLSDAKNVITE

LQSLRHLYESGSGEERNVNETIVPKLTNDPHENAMMLQQMGPDNMFDGAYDEAPPCSYNG

SFKSDSVIGLEKNISNLKRILFQREVSVVGVQGMGGVGKTTMALALSEDQEIKVAFRNNI

IFLTVSQSPNLKVILETMWEKIVRRKKPEFQNVEDAHRQLQQQLRRQAKPTLVVLDDVWS

RANLENLLFEGEGYKTIVTTRDRSTIPTSTSTNTSTRIYELPLLDEADALSLFCYWAFGQ

KSIPINADEHLVKQVQAECKGLPLALKVIGSSLHGEPRPVWESAKNKLLNGQSISDYHKE

GLVRCLETSIDVLDEEARECFLDLGSFPEDRKISVDALLDIWVYVRKMDWHDAFVILLEL

ASRNLLNLTSNLRGQAINYGSASEIYFSQHDVMRDLAIYLASRDRIVNRKRLLMPRKEDS

LPRKWELFNDQAFDAQIVSIHTGAMEEDQWCEMDFGKAEALVLNFSASNYFLPSFLSSMR

KLKVLIVLNYGSKRATVNGLPALSSLTQLRTLRLERMNFPTLQEHSKVLQNIEKLSLSLC

EGLGNMSRFNSTHLSLKLPIMRDFYVDHCCDLEELPYGICDTSSAENWSITNCHLLQKLP

DDMGKLRSLRMLRLSACLGLKELPASIGKLGNLEYLDISLCECLKELPEEIGQLKNLQVL

DMRE

>AB_020229_T.1

MVAKFLHACDGLPLSLKVIGALLRGKEDLKYWKAQFSKISKVLPRDIRSKLKISYDSLDE

EEKNIFLDIACFFVGEDRDTVKFVSEGSGWEGSLGILNLENKCLVEVDSGNCIRMHHHLR

DLGIYLAENEPPGCLRRLWRPIDNLALQNVPDQLSVRGICMVQKHSESAVELRNSRVVDM

IRLQLLRAKGDCMQSIFSQGYSPDLIWLCWDNCPYSLLPSWLPIKNLRVLQVSGGQPFQS

LWQSETQAP

>AB_020233_T.1

MAHLTAVAPHHTFASTSTSTYNYNIFINHRGPDVKKTFASHLYRRLLLHGFRVFLDQQEM

RAGDGIVSQIGNAIATASVHIAIFSPRYAESKWCLDELLLMLKSGATIIPIFFNVKPSDL

RWVGKDSVYHQALRKHEEERRYDPQTLENWRKALSDVSSLSGFELEGCKGDEGELVDKVV

QRLLKKGEKGLLNVAKYPTGLNEKLEDLENTVLLLQQQQRGNAQVVGIVGLGGVGKTTLA

KELFNRKSSDYGGSCFLFDVKEAAANGSLHSLQSKLLNGLIQLDRQIDSIAEGIEMLRTS

LKSFHALVIIDDVDHVDQLDAFLSVKDTLRSDSLILVTSRDKHVLTSSGIGESSIYKLTG

LNRQHSSELFCSSAFCQPYPLPGFEYLVDEFLTACDGLPLSLKVFGALLCGENDKSCWKD

QLDRLKQVNLPTEIQKTLQISYDALNGEEKQIFLDIACFFIGEDRDMALRIWNGSGWKGS

LGLRNLKNKCLVEVDSKNRLRMHDHLRDLGRDIVEHEKSSGLSHRFWRPTKNITDLFQGS

SVIVDVRGIRMVPRVDPLDVYTDLHLHSKMSPEKSWRDKFFGNRVKRLYNRHNNMLGMRR

LQLLITEDGYLEGILRRVQSPNLLWLCWYQCPYYSLPSWIPMENLRVLEVAGNKLKRLWR

HGSQAPLKLQELNICAPLLKFPKSMGQLKLLEKIVVDYPPMWTDLKTLPDESFHLKTLPE

EFCDLQSLKHLELKRWSTMKSLPDSFGNLTNLEYIDLSESYNLEMLPNSFGNLIQLKHLC

LQSCYKLTILRETLGNIKTLEYLSLSDCPNMEVLPPQVTLQSLEELYLLGTNLKELPSAI

GDLSNLEFLELGSPLLEMLPSSLGYLRNLQKLGLSNCQELKYLPDSVGLLTKLTTLQIEC

CGLCYLPSDVMKMNNLESLQVVDCPLHELPFSRALTKFDSIDKCMFRLKFLQLYNTQISE

VSFHEGVCPNLQYLKITSCDDLVEVGPLPPSLITLDLSSCLALRRIRGLCGLAKLQILNI

NGCKEIEELPGLETLKLLEDFRPDIYYKLIKAYSVGIVDKAEG

>AB_020323_T.1

MWLTETDNVHIIGVWGMAGVGKTSFLKVVYNHYIEATNVFQHVIWVTVSADYKVLDLQAR

IAKAINLELPSNSDIDMQKMLLSANLKEKRFLLVLDDLWRPLNLQELGVAFGEDKQSKVL

LSTRNRDLIPAMRAEKSIQIQPLSKEEAWMLFRTIAFNGDTVSVSMDIMECARKIAEECK

GLPLAITVIAAAMIGKTSVDQWEVSLDLMKTADPSFPDTHPRVDPELYQRLRWSYDALP

>AB_020405_T.1

MKPGSLIVVTSRNKDVLLKDIPQSSIYTLTPLNLQYSRELFCSHAFRPGDSVEGFDKVIE

EFVDACQRLPLSLKVIGALLRAGECDLNFWKAQLRQISKNI

>AB_020456_T.1

MTKVRRLPNDWERTLNRLLEVDAMNDSVMASLRLSYQVLPNHLKLCFVYFSVFPKNTEIK

FDYLLHAWTSEGFTPETTQEAFDVARSYIDELIDRCLMEVSKVSGDGRVKYCKMHDLLHD

LALSESHKQTKCLLMPGEELEKLPQRDCVDLRRISLIKNHISEINETIQCRGLRTLLLWN

NRNLTSISVSFLNNVRYLAVLDLSQTSIKSLPDTVGNLKHLRFLNLSQTKIQKLPKSLAH

VRSLQFLDVSLCEDLCELHSGIGEHKSMVYLNVKGCGKLKSLPVGISKLICLQSLKGAVL

KRKMATNAKDLQLKDLKGMTLLRQLTLIIDASASSPQDNIQLEKGTFGAMTKMRDLSVKY

INSRSLLHLPEDIEAMKRLEILHLGGCVVPKWIFQLQNLMELKLLGDNDSAHYSGLENIP

NLRKLQLGGNGNCIEFPVEFGQTGAFPNLEKFILENFKCSENFPSLRDEAMPMLKHLEMK

NCSQLKDMPEALAKLSSNLKEIEMAECCRAWEDSLWEGGRTWHLLKEHTIKLTIDGYKIC

WEMSAGENRREIEALREK

>AB_020611_T.1

MTSQIEAAIQMAFIQIAIFYANYAQSNWCLDELDPVVKSRVTILPVFYRVKPYPPCKLGR

EVYKTLRWIAIIIESVWSACLGENDIRLWE

>AB_020755_T.1

MGVLGKGGSGKTLLLKRLFSTQKVRNFFNDGFLLWITVSKSPSVKSLRDELCRQIAMQTK

EELVQNMMEEDAKLWLSQKMQGKRFALFLDDVWGEGGKLLEELGVALLTDHSNSKMIISS

RDRRALLEMGVSENATITMGDLVEEKSWDLFAYHAFPYNRGNCPANIEMTAKVVCAKCGG

LPLAIKVVGRSMAGITDAREWESVAQRLPNEKKQDLQALYDRLRW

>AB_020786_T.1

MKNCFLYCAMFQEDVKIEVQAMVEMWIAEGLMKTKEDAEYKYLLETGHSYVRLLQNRCLF

YSSYGDDGGPCISVHDVLHDMAIYIGENEENCVLRAGQSLQHFPHIPASDNCKRISVREN

HIASLPTKELKCPKLVSLFLGFNLLKKIPEAFLLNFTSLRVLDLAMLPIKSLPTSLWQLT

QLEYLCLSYTQIEHISEEIGNLLNLQLLHLRCCRNLKSLPSQIGELQNLKYLDIRRCTNL

NVIPDEIKACKILRGWI

>AB_020954_T.1

MADTVASSSSTSASLSSRSSYDVFINHRGSDVKKNFASHLYRSLLSHGLRAFLDQPELRV

GDNFPSQIIDALTNASVHVAIFSPGYADSEWCLKELVYMLDSRAPVIPVFYNVQPTVLRW

TQSKGVYAEALRKLEGKKTHDEQTTRYDPTTIKSWRDALSKVSEISGLELQGCNGDEGEL

VDKIVTRVLEIINSRAVQISGFYCSQPYGCPGVVDWSDGTYSGIRRITVRENDDYLISLQ

LTYGLEGDETVKDTLICGLQHGSGNGALKEYNLNYPEEIVTKISGYSGSFNTCEVITSLT

FHTNQRKLGPCGQEQGIPFETEERGKIVGIFGSTTGPYLDSVGVYTLKPRTSTYEEFYRS

QPYGGIGSSYWNDGTYSGIRRIIMTEKDNCLTSIQLYYGLEGAESVKNKYVMCPRHGCAG

GIRRVYDLNYPEEFVTKISGCLGRYGPFDVIISLTFHTNKGKHGPCGQETGRYFETEMGG

KIVGVFGSSGDLVDSVGVYMLKPRASDDKEFHRSQLYGGTGGRYWNDGTYSGIRRITMTA

NKMNWLTSVQLHYGLEGDESLKDGYFPGLRHGVLAADARKKYNLNYPEEFVTKISGYFGR

YGPFDVIISLTFHTNQGKHGPCGHEGGRYFETEERGKIVGIFGSSGDLVDSIGVYTLKPR

ESKDEEFYRSQPYGGFGGAYWNDGTYSGIRRN

>AB_021312_T.1

MAASSSTVSTSNNNYHYEVFINHRGPDVKNTFASHLYRRLLSHGLRIFLDQPELKRGDGL

TSQIKDAIRSASVHVAIFSAGYAKSIWCLNELLQMLDSKATIIPVFYKVKPSELRWTRAR

TEEATGEATGEGTEEATGEATGNYAEAFAKLEKKTARDPQTHEQGPRYDPATVEKWRNAL

SCVADISGFELDACNGDEGELVDKVVERVLKKVKKPALDVAKYPTGLDDKVEDFES

>AB_021314_T.1

MEAPSVGSSSYAVEEGNADVDLDGLIDYISQLLEPIKEGRKSSQKKTKTAQRYLPATSKT

FNKLRDFLSETRNFMKDITRGDAIKTVASGILQGMGMPNLVITGLVVVAAILERFDDVGA

NKIECLRLLQEMIFLAQLVKQFQERPLLNKGMDNVIKEATELIVESSIKCLSQMKSSKYS

KFFSTSANKEDLERFTKQLDKQYKHINAQMGIAIFDVHSSKNAPLSRRYPDHAVGLEDSV

KEVIHLLEWASDKKAVAIIIHGIGGMGKTTLANAVFSRLDLKGCKYSKVQLFKYIGSIPD

IVNLQSLILKDLMEPQKSVPDIRTPEDGQKELELALEKVTALICIDNVMDVDALRQLLPH

NMSKANKVRLLITARDLNVRKACPAMTVVPYPIRGMSSTEAMNLLQKEMSNGTAAKLNDS

QLRRIIEICGGIPLMLTLVGGVIRSEENKEKAYRIVMQDRQKLKGQTFGDKEHYLFAYDY

LPDTCRDPFLDICSFFNGWDWNTVKDIVGESEVDVLEKRALVTKNKDGMACVHDVILTIG

LQKTERTRFKFTSASEVDKLLKEEQGNQPIKGIWLSDNKDPLHINTSKLDSMHSSLRVLA

LGSSVKVEGKSNKVFEKLAFFQAGIPHLPFHMSKVKELRYLSYHPETLDLPEISSNLKHL

ELHGRLQSHATEISSRGLRQLPNLRILRLLSIPELEELPIDLGDLVMDLEELSLSHCESL

EELPRSISRLRSLRVLRMDYCSSLEQLPEEFSELQSLRVLRMDYCSSLEHLTEDFGLLNS

LQELNLQGCEALQALPNNFENLSSLKLLNLSSCTSLEELPHGLGNLTSLTALDLNKCDEL

RSIPESIGQLQLLSSSMDMSHCSSLTELPDAFCDLSFLTNLSMAGCESLDRLPDRFGKLA

SLRVLNLEGCQSLEKLPEGFGLLEFLEDLNLRNCYKLEELCNDFHCLSSLRDLILSGCLC

LKKLPEDFHRLVSLQHLFLCRESLKTLPEDFHLLASLQQLRLDDCPMLEGKCIDNVIKTK

NIQHVSIDGSTMLLERWAEIKRDGNQSWSFTVYTGQDLSEEEVNNMQKTFLSNLDGLLIY

GQGEPFHLSNCPPNTSILFIFDERPNLIFEYPWPLIEETLNDIEIKFEMIYIGKHFEKLP

GKVANKILGHAPLNTEVKTIVSNLLVILKDEDEDEDEDDDDDDDDVDVVYGIMSVTMMGD

GKDGKYLSGFKALSERNIEEFVVGKCEKYIRLKKIVEASQESNTEILREMFTPREEDSTD

RIFQKNCASHKAPSEVSKPLVTCGVDELKGKTILLEIRHVEVCYDIRLESLTEMYVTTKE

RPDFEIISIPIIQPGTQVDLDIFQRFSREVPWLVLRKPWMITTTVKYFLVKQCAMWEEDE

ELETCYPSVIWIIEPNGRITIYKPSVLSMLDRWGAKSFPFADEKIKELRKEEWEKQKNMS

SLEFLFIHSEKVSKQVKEILLQRKMICLYGGNRGMFEFTYFISAALTELRDSIHIIYIPT

YGFECKEKLWLTYGTSMKYYPLEKPYMSQAEMEGMPLLNLSEYEAMRFWTRLSSLKKEIN

EMDNTGMQLRGLQRLLHSLPAWGDKQMQLRRLMNSWMILMDKDGEMVTGRGKEIVHKLRY

ITEGKEEGLLLQGLLSEFEQQESEEERRFSEEAVDKEEQREIQKKIEPWNSEADAITGDL

ESESKNESGLLSAKASPTTRKEAYVASSSSSAPCWSSVSRSITTWDATNIEVEKMQERRA

TLKKEDKKIEKERGKEKQS

>AB_021662_T.1

MEQAYQVFINHRGPDTKKNLASLIYHRLTSCRGLRVFFDQNELHTGSAVYPSIKSAIESS

SVHIALFSKRYAESRACLDELCWMIQRSSHQRKIIPVFCDVEPSHLKGIDRPPYADAFEK

HQGSVDAGKVSMEVVEGWKNALREAAEISGLVFKTDESDYGQLLEKIVKTVLKALKWDLL

YVATYPVGLDEAAEDFKNKIMNQSELNATKIITVIWGMGGVGKSTLARHLYNVRRSDFSR

SCYLFNIREKDLLSLQQKLVRDLLGYDVDNTSEGKRILGNGLRGLRILVVFDDVDDKEKI

KSLLDVDAVEPGSLILITSRDKDILRGFSENTFLYHVKPLKRKHSQELLCRHAFRQEKPF

QRYGTLVVEFLEICGGLPLCLKVFGEHLAANLEIKYWKQQLENYFHQGRPLLETEAVINM

LKVGYEVLDPEEKGIFLDVGCFLVGEDTELVVRVLEGLGWLNARVALESLCTKCFLDFCT

HEESVDHNPYIHPIPNVLSKGCSKIIMHEQVKELARHIARNDSTGPLRVSCSYDIQQILQ

LQRPSIPFQIRGIRIPKGQKPPLLTIEITGVRLLVVDNPIDFSSFLGSSIRSSECMWGDL

VWLRLGSCSLRSINSSTITISLQKLRVLKLEGFDFDLEYFFERVVKDGYPAHLQEFNIQY

SEATTSLSRTVKMSHWTSDMFKGSRTSDMLRILRLSNHLNKIDFQNNRHIRSFPIDSFDF

KNLRYLNLSHSTNLIRLPKSFSKCLRLQYLALRDCQNLSFATDFLGDISTLEYVDFFGCS

KLVHLPKGMAYQKSLRCLDLRGTSLFHLPDNLKLLDKLEDLRIGSPELKELPHSIGDLIG

LKQLILLDCHRLNHISTSIKRLSRLERLVIDNSGVQSSLMQVERLPSPFMQVEGLPSSLV

NLMLMNCSKLKALTNLSNLVNLKILNIYNCKELETVNVEGLIQLEEIQAYSCRKLQRIGA

LTQLERLNFLELQIYNENGVNCDDILQLLTSRSQISTAKLYAITDDPMVGIEFEKVVQRV

LSNFENISVLDVPATTTSESPVNLENLHSYGAILMCFIPKRLRFRIRFEAANNPGKYLEC

GSKNGTESGMLDIFMWTEDSKLFEYKNFYNNVSVYCPSHGSGEDSDKGWIVPLARKADGL

EILKQFLVELRSMNGFPIV

>AB_021665_T.1

MSFTRYNPLERTVSSATGKYDVFLSFRGEDVRKTFVDHLYASLENAGVNAFLDSEKLEKG

DEISSTIKNAIENSAIRIPIFSMNYAESHWCLEEVSHMCKSGGLIIPLFYDVTPTEVRHP

DRGAFAKAFQQKKGRYSQAKINEWKRALAKVSSFSGWSRDDALGFEAGLVKLVVQHVFKT

LQNVIPDEDIPDKLPDVAEEIPPEEIPVEIRNEPLRISKSVFGMEEDMNKVINLLKTDSE

DSLITIAISGMAGVGKTSLAKAVHNHIYHKFDATCFVYDVRHQAQHTNGIAKMQRHILKD

LVKFKDKVNDEAHGKSLMRDRLRSIRALVILEYVGDNKQLDSLRGDWYGPGSRILVITLD

PQLLNDGQVDFVYEMQGLDREHALKPFSWHAFMRDRPVEGYEELSQRAVNICNGILLSLE

VLGAHLYNKDIGYWVEALQILENSVNQDKYAILRLCYDGLNSEQKEMFLDMACLFIGRKR

ESVISFWEASHCDPNRGLMDMILKSLISLDEDDRFAMHSELRDLGRAMVVEESTKPGDRS

RLWKPQEAELVVMEGKGTEKVRRLQYLQQEVTLQTDNLKSMCNLQLLWLDGALIEGDFSQ

MPQEIKWLRWENCPLKCLPCEWNMKYLAVLDLSFSKDIEAVCTEFSDEEGPSNLKVLKLN

DCTNLRVLPDLSNHTSLIRLELFGCTELKELPESVGRLVELKHLDLSNCYNLTYLPDTIT

NLSSLETLFLSNCHNIRKLPDSFGELTVLKKIRLNGAPLRELPQSFRWLSCLEKLTLQGC

CKLRSLPQTIGELICLRYLDLHGCSSLRELPDSIYDLKSLRHLDMTDCQGITLGEELGNL

VCMERLILSNCPALRSLPTSIGQLNCLRHLNMSNCKSLFRLPEEIGNVVCLEELLLNKCY

NLCELPQSIGNLSKLRILEMEENYSLSELPPSFSRLTSLEHLKSGGCCLPQQIGDFSSLE

VLCLKSYKFSSLSASISRLPRLNKLYLDHCTELIELSTLPKELLELFIQDSLGLRKISSL

SHMKRLKKLRIHNCRELVELPDFACLQSLQDLSISKCKNVKRIAGVEGLKSLRKLHVVGC

RLHVEGYSTSILRPLQLIKETRCLELFSFSANGVPESLEHKMQASGDDLLNAMLEINEPC

TGVILCFMVGFNGSITSLSIEMSIVRDFKEIFNTKLPNHSKNIVGNQIFVHILRKNHPLV

MMVQSGDVIRAKVEGDVARNLVGSGGMQLFSEGEDGRKEDLILGRLGKDLTLLMEKYSED

DDI

>AB_021775_T.1

MGVAKESTITMGDLIKDDSWRLFSYYAFPYNKGNVPANIDQETAKVVCEKCGGLPLAIKA

VGRAMAGITDAKEWGFAVQRLPNANSKDDQAVYDRLRLSYDALGTYNVHLQLGFLYLAAA

FLEDQVVRVNHAMLLWMGEGLVEAGHDPFEMGRVYANVLADRCLIEPTLRDVDGCVVNF

>AB_021819_T.1

MASTSHSSLDTPSTSTSTHSPNSYFDVFISHRGPDVKNNFATHLYRELRANGLQSFLDRE

ELQAGELLSPQIEEAISVAYIHIIIFSPGYADSRWCLDELRLILNKSGNTIIPVFYKVKP

SELRWAGQYAEALSNHEQKGRHDRQTVESWRKALKDVPDRVGFELEAYNGDEGRLLENVL

QCVLKNRRKIGFNVAKYPTGLQEKVREFENTLSLQQRQSGKVKVMGIVGMGGAGKTTLAK

ELFNKKFSNFGVSSFLFDVRLNGGRSSLNSLQTKLIKDLAHRDVQIDNVDGGIESLKTYV

ASKHALVVLDDVDAADQLDAVFLPIMEVLRSDSLILVTSRYKDVLASSGIAESSMYKLTG

LSKQHSQHLFCSYAFCHPRPPPEFKYLVVEFCRACEGLPLSLKVFGGLLCENKNKSYWES

LLDRLRQINLPNEIQQTLKLSYDALEEEEKQIFLDIACFFIGEDRDMAIRIWDGSGWNGL

VGLVKLESRCLVEVDRENNTIKMHDHLRDMGRGIADEKYSSEVSWHRHRLWRLTEDIHEL

WKQSTAIIEVRGIRTVPHLIDYYQIPKTGRFIDNYFIPGFINRNLPITEVRRWCDDVLGT

RLRKLIDTTPYRFRNLQLVATQGLDLESILRSVGSPNLIWLRWNNCPFSYLPSWIPTKDI

RVLQVGGNKLEKLWRAESEASLELRELYIVAPLSKFPKFIGRLKHIEKIVVMQSNLKSLP

EEFSDLRTLKYLLLSGADLEWLPDTFGCLTNLQHLDLRNCKNLRGLPYSFGCLLNLQHLD

LRNCGNLPGLPDSFGCLLNLQHLDLRNCGNLPGLPDSFACLINLQYLNLQGCKGLQRIPD

FFRCLRNLKHLHLLGYLEFLLKKGYFPVTLPFKPNRCDELGAIDPEYYEPADEPCCLKHS

YDPDSFDKLNSYDPDFYFPSSETLLQWNHVRHRTREL

>AB_021910_T.1

METVFIGAASNSISSGVTVAIAPLIQLIEDAIHLDRNTRLLQDKLSSLKALLQGITNQFQ

GDQRSPPEIVNDGLQRLQRSLEEARGLIDRYLQQNQCLNCLFTCKPRPRLSRQVREWNTN

LDQLLSSLQRDFSIFSNTQQIAASTPLRPETLLQPVPEAGFVGLGIQDAETMVESWLNME

DQFRVIGIYGMGGIGKTSLLKRVYNTCKQGNVFQAVIWITVSQNVDILNMQKVIVGKENL

NLGSSSNSESSSDAQMLKMSIFTCLRKMKFLLILDDMWSYLDLEEEVGIPFGNEKGSKVI

ISTRSTDVIRRMRANEYSIQIQPLSEEEGWELFCRKAFKDGVAPTQSIEDVARLIAGECK

GLPLAINVVAAAVMGNETVEQWNFALSQVKNADPEFLTLRGIDEGLYQRLKWSYNRLPNS

NYQNCFLYCGAFPEDAQIKVEILVEMWIAERVVVHTHSRETKSLMDKGRGYVQLLVERSL

FEYASSDKSRIKVHDVLRDMAINIGEEEENCLFTTTQNLQNLPVEKGIDNLKRIAVHSHN

DIPFLPTELRCPNLLALFLARNKSLTQVPNGFFLNVISVRVLDMSRCTKIESLPESMWHL

TQLEFLDLSETSIKNLPEDICNLVRLQFLHLSWCEELESLPSKIGELKTLKHLDLRGCPK

LTEIPREISQLSSLYKLGLKGIALKTEEARSGVCSLKDLTNIPNLMELSVEVMPGIEVDG

IRSGIKVGIMSTWLEMRHLSLSFHVGGLGDDVVMEDLPADMHNMKELQSFELFDYYGRRL

PNCICDFKQLERLELFRCSQLSELPLLERLPNLKSLWIQDCEKLRELGIGRWGSAAGFPM

LEELRLLFLPMLESMRSSSSNIMWSEGMMPNLRRLTIMGCPSLKRVPEEIVKLPKLKEIQ

IAASEDWWENLIWEDDNVPKLLKDKLKKMSW

>AB_021940_T.1

MPAKTVVRDFSKGIGQVHWVFAGLLVVANTLERFETLSANRNECLSILEEMSNLAQHVKD

LKELPGLEKGMETSIKRATEMIVEGALMCCTQMSASRISKFVSSTVNKEDLVKFQQKLKK

TNDFMSQQMGKCTYEAIQSIFDMIQQMGTFHAIQSKNVRPPVKGRERPYPEYAVGIEKQV

EEVIRLLEWESPKNVLAVMVHGFGGTGKTTLADAVYARHCKDGCKYSKVRLFEDINSTPN

IIDLQKCILKDLMLGSREEDQKLMLEDIRTFEDGQRAIGRMLEKELVFIYIDNVLDKDPL

AKLLPKHKDMEKSRKCRLVITTRDAEVRKGCSMDEVKPYPVKCLPSTEAMILLKKELDYE

KTDHRKRLNSDQLHRIVKLCGGVPLLLSLLAKFIRYDGRGQEKAFSILMEDMEEKKEWNE

IGDIEKKSYFFAYDHLPERVKDPFFDICSFFNGWDWDEVTDIVGESNLDVLEKRGLVNKD

RDMTVTVHDVILTIGCKKTKGTRFRFNTKSEIEEFLKKDIQGIKGIWLNDIQGIEDIQGI

HTISIPATKLDCMSHSLRILALGDSAEVDGTCKEIFEEIVFFQGEVPYTPFDVSHSKELK

YLRYEPHDWNLLEMPSNLKHMELNGRLHSEDAFKILSSDLQQRRSLRTLKLVNFARLKNL

PEDFGSLTSLQKLNLSFCNSLEELPGSISKLQSLTELDMKFCSSLKRLPEDFGSFSSLEK

LNLSFCHSLEELPGSISKLQSLTELDMRYCSSLKRLPEDFGSLTSLHNLDLSGCDSLEEL

PGSISKLQSLTELDMRYCSSLKRLPEDFGSLTSLQELDLSNCNSLEELPGSISKLQSLT

>AB_022155_T.1

MADLSLTAPASVASGSSSQVYSHFDVFINHRGPDVKKGLASHLHTRLRVHGLRVFLDDRE

MEPGHPMTPQIQSAIRTASVHVAIFSPGYAQSRWCLDELLMMLDSGSTIIPIFYGVNPSV

LRWTQGGDGVYARDLWKLERETTTDSQTNKKKTRYDSNTIEKWRKALSDVSHISGFELQA

FNSDEGLLLEQVVHQVLNKVTKPPLVVAQYPTGLEDKVKEFETTVLSQQQSGKTGSVGVV

GLGGVGKSTLAKEFFNRHRSSYDRSCFVYDVRENAAKNSINLLQATLLKELAQRDEQING

ANEGIDKLKHHLSSSHRALIVLDDVDHIKQLEALFLPVKDAMKPGSLIVVTSRNRDVLIS

SGIPQSSIYTLTGLNPQHSRELFCSHAFGPAASVEGFDEVIKEFLDACQGLPLSLKVIGA

LLRAGEGDLNFWKAQLRKISKILPDDIQSTLKISYDSLDKWEKQIFLDIACFFIGEDRDT

TIRIWDGSDWEGLLSFWKLENRCLVDVDSENRIRMHDHLRDLGRDLAEKEHPGCRLWRPT

DNLLYNLSGGQHVRGVSMVPWIEQSFENLAGRHLVTNMSSLQLLRAEGYFVEHLFSLGNL

PQLIYLRWDNCPS

>AB_022856_T.1

MFCSPLACAGDHILNKLDVFNLKKPCSKLHGLLAGFIQAVEPLCLPKSKKEQSPIYEQFL

SILNNGVELVEKCEKAGRFNVFQNVRYAYQINQLEKEISDFVRFHLP

>AB_022909_T.1

MAHRDFSPASQDTPIPVSSTSVPASSTSVFINHHDLKVQKTLASHLHRRLRDHGLEVFFK

WLQLAGGDNPLTCEIEGHIREASVHVAVFSPGFAESRWCLNELMLRLESMKNPESGLKII

PVFYGVEPSELRWTKGVDAKGVYAEALRNFQEKNPSESNIEKWRKALYEVAEKTGFELRA

FNGDEGKLVDKIVKRVLKMLENHLYYHVFINHRGPD

>AB_022939_T.1

MGTAHLACTGLLVVALVLERFDTVSTNKEECLCVLDEICKLGKHLNDIKQRSRLRKDMEA

SIREATDLIMEACITCCTQIGYSKGRRLLSASGDKEELVKFQQKLQRMYKHMDNKMSICL

YDATECRKVRPQKPRERSYPDYAVGIEKPLEEVKKLLDWESEKKAVAVILHGFGGMGKTT

LADAVFARVYVEGCKYSMVRLFDDVTSTPSTSDIVKLQKYILEDLKMGTPEETTHEIRTV

EEGQREIGRILEKEKVFIYVDNVLQRYPFEQLLPRDMDKAKRLRLLITTRDTDVSRECRK

LETKIYAMKGLPLTEAKSLLEKEMYTSYTDNMEKQLSSTQLDEIVEMCGYIPKLLTVVAG

FICCEEDKQKAYIRVKEEREKNWKGEIGKDIAHYVFAYQTLDEGLKDPFLDICSFFKGKK

WSTVADIVGDSELNSLEKRALVTKKRTEDWTKFYRNETIETVWVHDVILAMGYQQTEGTR

FTSAKKIQEFLDEKEDKDIQNQKIKGIMLSEYKDCLSISASKLDLMCSSLRVFQVGKWSK

VDGNCTKIFEKLIYVEVDIPNFPIDVSRSKELQYLRIFPIDVSRSEELQYLRWEGEGDLN

EGEGYLNEELLRSISKHQYLRVLKLHSCNNLTHLPKEMGSLNSLQKLHLDMCTSLEALPS

SLENLSSLQELHLHYCDRLKALPSSLGKLSLLRSLVLRKCKRLEHLPKDIGSLNSL

>AB_023159_T.1

MIVSIYTSVYQTIYNLPHNLSALLKQVANLLLQSLPISVSIFTWQMDASTSTSAHANKNC

YDVFLNHRGPDVKNTFASHLYHRLISYKLRVFWDKEEMQTGEKLTPQIKGAIESASVHVA

IFSPGYAQSKWCLNELLDIVESKAPIIPVFYNVEPTDLRCTHAQSQGVYAQALRDFEQKR

RYDSTIIEEWRKALSSVRETSGLDLGAFNGDEGALLQGVIQRVLKTVEKIPLNVAKHPTG

LDEKAQDFEEKVLSKQHSKRARFVVIAGMGGVGKTTLAKKLFNEKISDYSNSYFLFDIRK

NYKSSLLSLQSKLLNGLTHLNLHIDSIDQGIEMLRKYLEKMRVLIVLDDIDDVDQVDKLL

PMKDFVNVLQHESLILITSRNKNVLARPGVQESSIYQLTGLNERNSRELFCSYAFDQPHP

P

>AB_023243_T.1

MDSFNCKKTSSRLEGLLEGLIAIFQQLCLLNLRKPKNEQNLIYEQFLKTLMKGVELLKKR

ENTSCLSFFHSLRYGFQIRQLEKEISDFLQYQMPLKILLEVKNLVAELNNLRQLYELGSM

DESKMNETIFKHVSKLTNDPQQNALMLQQMGADDMFDEAFVEVPYNYNGSGKPDFVVGLE

KNIWNLKRTLLQRDLSIVGVQGMGGLGKTTLALALSNDKEIKDVFQNNIIFITVSESPNL

KVILETIWEKIVRRKRPEFQNVEEAHRQLQQQLLRQAKPTLVVLDDVWSRASLEKLLFEG

IGYKTLVTTRDCSIIPKATSTQLYELSLLDDADALSLFCFWAFGQSSIPSTEDAHLVKQV

QAQCRGLPLALKVIGSSLHGKPRPVWDSAKNKLLNGESISEYHKDGLFKCLETSIDVLDE

EARECFLDLGSFPEDRKISVDALLDIWVYVRKLEWRDAFVILLELASRNLLNLTSNLRSQ

AINYGSASELYFSQHDVMRALAIYLASRDRIVCRKRLLMPQKEDSLPMKWKLQKDQAFDA

QIVSIHTGAMEEDQWCTMNFCEAEALILLFSA

>AB_023296_T.1

MGTAHLATTGLLVVANIVERFEDVSKNRDECLNLLKEMIFLAKVVKQINERQELRERMHY

EVKDALELIVEGSIKCCSQITSSKFSKFFSTSVNKEQLGELARQFDAKYKHINTEMGIQI

YDVIQYRKANLSRLYNEKAVGIEGGIREVTDLLEWGSDHNAVAVILHGFGGMGKTTLADA

VFSRVNIEGCQYSKVQLFENIDSPPKITKLQKLILKDLTGSENILEIRKHEDGQLELTRV

LKKVTAFIYIDNALGERELGQLLPEDMTEAKKVRLLITARDINVRKACPVKTATNEYRMR

GISSMEAKNLLNTEMFDEVERKLHFSKVDHIIKKCGGIPLMLKLVAKALHVAKDTQEVLE

VLDELDKLKGEDFGPDKIESYFFAYEKLPTECKDPFLDICSFFDGWDWDIVANIMGKSEL

EMLARRALVTKHTSGVVSVHDVILKLGRRKSHGVRFIFTGAIQMKKFLDEKEEKDLHNIK

GIWLEENRDLFSISAAVLDSMHESLRILKFGNLTKIEGKCNKIFKDLIFFQGAVPYLPFN

TKELKYLSYQPKDLKLLEIPRSLKHMEFDGRLHSHAFEISSRELEQFQNLRVLRLTRFAK

LKKLGNLVNILKELTELTLSYCQSIEELPSSISMLPSLRVLRMDYCLNLKHLPDDIGSLN

SLQELYLQGCTSLEVLPNSFEKLSLLKSLDLSSCEKLSEFP

>AB_023524_T.1

MAQHQASASVASGSSSQVYSHFDVFINHRGPDVKKRLASHLHRSLRDRGLKVFLDDREME

QGHHITPQLHWAIRTASVHVAIFSPSYAQSRWCLDELLMMLDSGSTIIPVFYDVNPSELR

WTRGEDGAYARVLRWIQGVLGWIQGGDGVYARDLWKLERKKRYDSNTIENWRKALSDVSH

ISGFELKACNGDEGLLLEEVVRQVLNKVTKPPLYVAQYPTGLEEKVKEFETTVLSQQQSG

KTRAVGVVGLGGVGKSTLAKEFFN

>AB_023538_T.1

MRESGAPIIPIFYKVKPAEIRWTDENKGVYAKELEQLQMKKTYDSETDQEKPRYDSVTIQ

KWKNALSSMANINGFDLVDGRFNGDEGQMLDELVERVVSIMAKKKRPYVAKYPTGLDEKV

KEFETTVLRQQHSGQVQVVGIVGLCGAGKTTLARELFNRMRSKYEKSYFLSDAEANAKKT

SVHSVLSDLLKGHDIDVGIRKLKWDLKSSRALIILDDLDHVNQLEAVLTVKDVLSFDSLI

LITSRNRDVLISSGVDEFSIYKLTGLNWQHSLELFCSYAFCQAYPQQGFEELVDRFLEAC

DGLPLSLKVLGAHLFGRDDKSYWKEILNQVKVPADIQTKLKTSYDSLSVEEQQIFLDIAC

FFIGEDRNSAVRVWEGSGWKGSLGFKSLQDKCLVEVDRENIIQMHDHLRDYGREIAKDLL

PRRLWHSTEKIDDLLHQHSSARTEVRGIKMLPDTDGDDETLSWYQRSQELFGNRLMKLHN

ALFHGVGVGVMKLQLLHTKG

>AB_023625_T.1

MDKAKKLRLLITTRNTTVSRECSKLKTEIYTMKGLPPIEAKSLLEKEMYSDADNMEKITV

SQLDQILEMSGYIPKLLTVIARFIRCAEDKHKAYSRLKEEREKNWKGEIGDDITHYVFAY

QTLDERVKDPFLDICSFFNGEEWSTVADIVGDSELNSLEKRALVTKKRTKGIDFYTVKTI

EKETVWVHDVILAMGHQQTEGTRFSSAEKIQEFLDEKEDKDIQKIKGIRLSKYVSNCLSI

SASKLDLMCSSLRFFQIGKWSKVDGKCTKIFEKLIYVAVHIPNFPIDVSRSRELKYLSWE

GDLNEELLRSISKLQYSIFKSVKTTQL

>AB_023839_T.1

MGIVGMGGIGKTTLAKELFSKKSSNFGESCFLFDVREYASRGSLNSLQTKLIKDLVHIEK

RIDHIDEGTEVLKTSLKTSLHALVVIDDVDDADQLNAFRPLMDVLRSDSLILFTSRYKDV

LISSGLVESSIYRLTGLNEQHSQQLFCSYAFCDPHSPSLFKDLVDEFCRACDGLPLSLKV

FGSLLCGKRDISYWEGQLNKLSQIKLPDKILQTLKLSYDALEEEERQMFIDTACYFIGEN

RDMAIRIWDGSGWNGLVGLANLQDRCLVEVDTENNTIKMHDHLRDLGRQIVDQQKPCGVI

PHREWRVMEDIPELWRQYSGITEVRGIRTVHRLNAD

>AB_023850_T.1

MTRLAKHVKRLKERERLKQEMEDIIKETTVLIVKAAIMCSSQMDVSRCSKFFKSQSNMIK

LQKFREKLQEMYSRMDKKMDMCVFDAIEYGNKRSLLTRGRPYPEYAVGIDKGVKEVLRLL

DSESEIQALAVILHGFGGTGKTTLADAVFARAYIKGCKYSMVRLFDDIRSTPNIVGLQKL

ILQDLIGQTEGVKKKIPDIGTFEEGRREIGYILKKEAVFIYIDNALHRDALEQLLPSDMT

KAKNLRLLITARDKDVLRGCRHLKKKVYLMKFLDAEEAKELLNKEIHHEIDDLHEQINSS

QLDNIVQICGGIPRLLISVAGFIGLEEEDMQKAYRTIIQEKEKLTGQLLADVEHYVFQYD

SLPVMCKDPFLDICAFFKGWDWNTVSDIVGDSELDMLEKRALVTKDTNMTLQLHDVILAI

GNQKGRGSRFIITNAKQLEELLVKDMRGIKGLWFKENKDLLDMPATKLNSISDSLRILAL

GKFTRVKEKCIEKFEKLLFFQGEISDLPFDVSHLKELRYLNFQPQNLDLFKEIQSDVRHM

ELNGKGLSDVWKIPSRDVQQLQHLRVLKLVGFKEVGKLLDQLGNLANGLQELIISDCSSV

QELSESISNFQGLRIVDIEGCESLKVSLGVSEWKRLILNEKGCQNLRMYLSSLDVLSLND

EDCENEKVYLEPFNVPQWKTLLFDFDEEEGCESVPVYVDSLDRSQWKTLLFDFDEEEGCE

SVPVYVDSLDRSQWEALVSNIVEEIQ

>AB_023856_T.1

MAASSSSTFSATNPNISYCPDVFLNHRGVDVKKTFARSLYIRLSSNGLKAFLDQEELQAG

YNFPSQIEQAIKTARVHVAIFSPRYAESEWCLRELVMMLESKAPIIPVFYRVQAAEVRWI

KDKYAGHLQRLAEKKTEEGKPRYDSKDIDNWRAALSHVADNKGFEFNGEELELELLDKVV

KCLLEMVKKPDLHVAKYPTGLDEKLKDFENTVLLSQQQKGKSQILGIVGLGGVGKTTLAK

EFFNRNNSNYHKSCFLSDVRENAAKGCLTSLQGKLLKSLTSIDVPIDSVDEGKGMLIKHL

KSSEAPAEILDDAIDSVDEGKGMLIKHLKSSEAPAEILDDVDHASKALIILDDVDHLDQV

DALLPVDLSSDSLILITSRDRHVLSSKAENSSIYNLTGLKDQHSRELFCSHAFNQTDPLA

GFKSLTDRFLKACNGLPLALKVYGANLFGRDRSYWVDELESFPGEIKDRLKISFDALNNE

EKQIFLDIACFFRG

>AB_023934_T.1

MASSFTMASSSGPASSSVHDVFINHRGPDVKNTFASHLYRRLRDCGVTAFLDREEMEKGE

EFFSQIVHAISGASVHIAIFSQRYAESIWCLKELVLMKNSRATIFPVFYKIEPSVVRWTD

KKGAYAEALRGLEQKKIQDAETGEEKPRYSSDTIQEWRNALYYVAGLDGFTLRGDEDEVK

FLDEVVESVLKKVPKPPLHVAQYPTGLDLKLQELEKILLSEQQQEERVEAKVLGIVGVGG

VGKTTLAKEFYNSHRLRYNASSFLSDVREVAVRMSLEHLQSQLIKDLKHVETRIGRHEGI

EILKRHLSFCQVLIIL

>AB_024366_T.1

MPPLLVDPLTGAAIELLLSGLLGVLKIIYSSKTSGKELKDKLSSLKPIIEQISRKFESDS

DSFLDRGKHFKDFQKAVVEDGLKLVEKLEKLQSFNFYRKYRCGRKIQKFDKKIRAFIDIQ

GSPLVVLDLHNIFEKICDLGERCDRIQCSVQKMTDKPTFNTRTSITQLNQSFIHGMPEAT

AAPQSVSCTSQVTVTPNEFVVGLDNLINGVKQILFRNDVNVVGVAGMGGSGKTTLALALC

KDTQVQVSFPDRIIFITVSQLSDQKGLLKSDQKGLLKILETMWDEIIRSERPHFQSIEDA

HRQLQEKLSLRTYPQILVVLDDVWDRFDLEYLLFEAKGYKTIITTRDNSIILKTKNAGLY

SMPMLEDAHALSLFCFWAFDQPSIPSTHDENLVKQVEAECRGLPLALKVIGSSLFNEPQQ

VWENARKKLSRAESILGYHKKGLINRLETSIDVLDDKPKQCFLDLGAFPKGKKFSVDLLF

DVWVYVRGMEREEAFVVLYELASRNLLNITSHPGSRAINFGCALEISFSQHDALRELALH

LASKDSSILFMPHREDRIPPKWLEHKDQASKAQFVSIHTGPMKEQDWCQIDFSEVEVLAL

FFVARQYCLPTFLHSMPKLKVLIIYNYSSKRAKLDGLPGFPSFTQIKSVILERLIVPSLN

EYCRSWESLEKLSACLCEDLGNMTPLDNEPVLTFPKVVEINLDHCSDLEELPGKICNLTF

LQWLSVTNCHSIQKLPDDLERLRSLRVLRLSACLGLSMLPASICKLQQLEFLDISQCSYM

KVLPEKFDQLTNLKTLDMRECSRLKKLPEAFAKLRSLRHVICDENIEKQWLHIKASAMPN

LTVDVVKESFNLDWLYD

>AB_024372_T.1

MKKCAGLPLAIKTIGSYLARVESKDWKSKLNSLPEAEAMTDSVMHSLRLSYEALHDRVKP

CFLYFSVFSKNTLIQSESLVHAWIAEGFISTSTQEAAEDAYDVGCSYLDELVDLCLVEVS

QFDGNGRVKYCKMHDLLHDFAHSESLKQRKCLLETGGELGELPVERCLGLHRISLVKNNI

SKIKDTVQCRGLRTLLLSYNYSLQSISSSFFNSLRYLSVLDLNRTSIRSLPGSIGDLKFL

KYLNLSGCTSISMLPKSLSNLRRLEFLDVSYCQKLRKLHSGIGKHKFLVHLNLKGCERLI

SLPVGISKLISLHTLKGARISMSKKAANALQLRDLKGLTLLQQLLLLIVDGDGRANQLEE

GIFKGMTKMRILEVLNHNSSILHLPQDMSVMERLEIVRLHHCKLPEWIFRLENLNELTSC

RDDSLDYTGLQKIRNLRKLQLSENKACVEFPEVFGEAGAFPKLQKLSIEDFRALKIFPPL

KDDAMPSLKYLQITECNQLVNKPKRFEELKGMEEEQDLELCDLPSEIDAYNLTAKTMRSL

QQLQLPLPLPLQQLLQQQAAIH

>AB_024409_T.1

MASSSASTAASPSINSNSNYDVFLNHRGPDVKKTLASHLYHRLTDRGFKVFLDKKEMREG

ENLNSQIKGAIATASVHIAIFSPGYAQSRWCLDELVLMLQSKDTVIPVFYHVEPSDLRIR

TGNYGMYARVLTKVGWTDKSEVYANALRIHEKKKRYEPKTIAEWKQALFSASSLSGVELK

EYNGDEGELLDRVVQLVEDKVRKPELDVVKYPVGLDEKLRDFEETVLQQQRHRRKSKVVG

ILGTAGIGKTTLAIEFFNRKRSAYHQSCFLQDVRDETCLMSLQNKLHRGLTSRDLKISSI

ADGRGQLSHRLSGSKALIILDDVDHAHQLDALFEPVKDVLDSDSLILITSRNKDVLSRIE

ASPIYKMTGLNKEHSLELFCLHAFHKPQPVVGFEEVVAKYLDACDGLPLSLEVLGSELYQ

KDLEYWKARLLKISHDLVLPPDIQSCLKISYDSLDSQEREIFFDIACFLIEEDRDLAIRI

WDGSGWKGRLNLQNLEHKCLLEIDGKNCIRMHNHFRDLGRNFSEKELPTCHRRLWRRTEN

LLYNAFRQPTVRGITMVQETSGQSIHNHVELGNFSVDMSKLHLLKAEGDCLESISNFLRH

SDQLLWLCWNKYPYNSLPSWIRTRNLRVLQVAGCQLKKLWNSESEASLKLEHFEMKDAIV

PLPEDFCHVRTLKHLRLQTTMVSLPDSFRNLTNLKHIDFSGCADMHMLPESFGDLRDVQH

VSLNGCFRLEMLPPCFGNLPLLQHLDLQETAIKKLSFPEGLYPSLQHLNISFCRQLEEVG

PLPTALISLDLRGCSALEKIIGLPGLERLRLLDISGCNKIEELPDLQNVYIVR

>AB_024425_T.1

MPAKTVVRDLLKGIGQAHWAVAGLLVVANILERFETLSANQNECLSVLEEMSNLAQHVKE

LKERSGLKKAMDTSIERATEMIVEGALMCCTQMSASRISKFFSSTVNKDDLAKFQQSLQK

MYRRMDNQMLICILDAVESKNVRLPVKGRERSYPEYAVGIEEQLEEVIDLLEWESHKNVV

AVMVHGFGGMGKTTLADAVFARLYIEGCKYSMVRLFDDINSTPNIIELQKCILKDLMLGS

REE

>AB_024497_T.1

MGRDGALLGCLPCRCCSATQEPDDDAPSAHSQRPDRSAHRVQEVPSSRLVPMPRQRAYPH

HAVGIDDQVNRVIQLLEWENETKVAVAVVLHGLGGMGKTTLADAVVARLDIQGWSCSTVL

EPRAGFVPVRNFEDHQQEIQDMFGKEQAFIYIDNVTVRVEHLDKLLPNNSCNKKLRLLLT

ARDEMVADKLKECGIHSHVYPVKPLSLDAMELLWKNIRVPGTIPTPQINEMLKICDGVPL

VLERVGAYICQSEDKDEAYRRIIEWGKDGRPFSFAEEYSIEKKGLLFDLDELPVSTKETF

LDICSFFNGWDWDKVSCILAEDD

>AB_024500_T.1

MLTMFCNDILLSNFCQEIWIRQKKLRLLITTRDTDVSRECRKLKTKIYAMKGLPLTEAKS

LLEKEMYTSYTDNMEKQLSSTQLDEIVEMCGYIPKLLTVVAGFICCEEDKQKAYIRVKEE

REKNWKGEIGKDIAHYVFAYQTLDEGLKDPFLDICSFFKGWEWNDVANIVGDSELNSLEK

RALVTKKMTLVTKKRTERGKIYRKKTIETVWVHDVILAMGHQQTEGTRFSSAKKIQEFLD

EKEDKDIQKIKGIMLSVDDPLSISASKLDLMCSSLRVFQVGKWSKVDGKCTKIFEKLIYV

AVHRIPNFPIDVSRSKELQYLRWEGEGDLNEELLRRSISKLQCLRVLKLHSCNNLPKEMG

PLNSLQKLHLGMCTSLQALPSSLENLSSLQELHLSNCDRLKALPSSLGKLSLLRSLVLWN

CESLKHLPKDIGSLNSLRTLNLYECKSLEEIPSSIENLSSLQKLRLSSCDRLKALPSSLG

KLSSLRILDLSQCGSLKDLPCDLGKLTSLTELFLYGCKELISIPSSLWDLSLLRILDLSE

CRSLKDLPCDLGKLTS

>AB_024513_T.1

MGKTTVADAVFARLYIEGCNYSMVRLFDDVTSTPRTSDIVKLQQYMLKDLKMGTLEETEV

SDIRTLEEGQREIGRILQREKAFIYVDNVLGRYAFEQLLPKDMDKAKKLRLLITTRDTDV

SRECRKLKTKIYPMKGLPHEAAKSLLEKEMYSETDNVQKQLSSDQLDHIVQMCGYIPKLL

TVVAGFISCQDDKQKAYRIVKEEKEKNWKGEIGKDITHYVFACQNLDERLKDPFLDICSF

FKGRNWSEVADIIGDGELNSLEKRALVTRDTTSRVWVHDVILAMGLQQTEGTRFTSKSQI

KEFLDQKEDKDIRKIKGIELSGYDYGQSDPLYISASKLDLMCNSLRVFIAKSMVKMDGKC

TNIFEKLVYLEAEVSNLSFDVSRSKELRYLYLVDLNKELTPSNVEELSRSISKLQSLKVL

RLYGWKSLRQLPEDCGSLTSLQNLYLDTCESLEALPSSLGKLSALQVLDLRWCKRLKVLP

SSLGKLSLLRSLGLCGCESLKDLPYDLGKLSSLAELGLNGCEELSSIPESIGQLKSLGSS

INLWRTGLKELPDGFCNLSFITDLQMGYCVGLEKLPVRFGEVTSLRKLGLRYCKRLKKLP

ESFGQLKYLEHLDLSECTILEELCKDFNGLSSLRVLSLDSCKMLKKLPEDFDCLTSLEVL

SLRDCHMLEGKWMEGMVKMKTLQIVNIRGSPMLEESWEEIQREGDQSWTFVVCTGQGSTN

EHVLKQLHSKLDSILTNGRVTDLPLNTLLLVMFDGNQKFIRNSYFSTSNFIEENIDDIQT

NFEMIYFGNNFDKLPKKMADRILDHAPANSNAQALFQKVLSAIGAIGAESNWRDPSGRCF

EMSAKIVSDEKGDKYLSCWELLSLRSVEEFVIKKCGRYLKLKQMVEKGGESNIELLRELL

ASGEEESTLTFVKNDAAVRVEELEGKTILLNIWPTYRFYDIPLQSLMEMYVAEKDRLNFE

IITIPIVQFRSTEPEVLQVPWLLLQNPRQLKSVIKFFLVAECAEWFRESLEVGRSKPNWD

RWHPSIMRVIETNGTIAPHNPAALRMVNTWGAKAYPFTEEKIKELEGEERNQMKAMSNLM

FLFNNQESLWDKVLMLFIGAGSRIYFIGMESDSIQQRIS

>AB_024600_T.1

MASSTISRIHSDKIEMENRSYPPATATGASCYYDVFINHCGGDVKKTLASHLYRRLLAHG

LKPFLDQQELQQEGLDFPSQLEDAIRSASVHIAIFSKNYAESRRCLDELLLMRESGAPII

PIFYKVKPAEIRWTDENKGVYAKELEQLQMKKTYDSETDQEKPRYDSVTIQKWKNALSSM

ANINGFDLVDGRFNGDEGQMLDELVERVVSIMAKKKRPYVAKYPTGLDEKVKEFETTVLR

QQHSGQVQVVGIVGLCGTGKTTL

>AB_024653_T.1

MITGLNKQYSRQLFCMHAFSQSHPLLGFENLVEEFLEACDGLPLSLKVFGALLWGRNEKS

YWQDQLYGLHQIRLSEIKGKLKMSYDSLNKEEQEIFLDIACFFTGENKDTCIRIWDGSGW

NGLRGFQNLQNKCLVEVRSDNEIHMHDHLMELGRGIAKASGSRLRLPCETNNIDELLQPL

SAITDPRGIVMRPYIVYDDHTDFVHSFLRSDMPWYRRMIHELLGKWMMKIRKARYNTLQL

LDIEGVLLERILKRVQFPNLIWLRWLKCPYSSLPSWIAMDNLRVLHVFGRELKTLWQDEL

QAPLYLRELEIWGSLSKFPKSIGQLKHLERIVVNWVPESKNIGNLIKLPEEFCDLRSLKL

LELTWSNLKSLPESFGNLTNLEHINFYGSKNLERLPNSFSQLIRLKYLDLGSCNNLTISS

ESFGNISSIEYINLLNCEKIEELPLQVTRQLSLEQLILKGTRLRALPSEVGALNDLEVLK

LSSPLLKALPDSLDRLRKLKKLSLYDCEQLKCLPQSAGRLSQLTKLTVKDCPVLKWLPIS

MMTQLIKLKVIGCSNLKNIEGLWGLVKLQTLNVRRCEKMEKLPSFDTLVVLEELQASQCV

KLKRIQGLAQLPRLRTVDVSKCSNLKALEGVEQLKLLVRLDVSGCPKMQRALSRDGGLME

HLRQRLKKELVM

>AB_024884_T.1

MHTKENPDKNMSQEAVQIWLNERLQGKRFILILDDVWEKGAELLEELGLLRLTHSSDSTV

IVSSRNRGALLGMGVPEKSIIKMGDLDEDDSWRLFAYHANVPTNIDQGTAKVVCGKCGGL

PLAIKAVGRAMADIPYAQEWEFAARRLPIANSQGQETVYQCLRLSYDALGYDVNLQMCFL

YLAAFIEDEIIHAHYQVIPLWIGEGLLARKKPEDQCGHDPVSMGKMYLNILADRCLVEPI

RRDVDGQVYSFRMHDVLRVLAIEIAEHEENFFCRAGK

>AB_024933_T.1

MAGSGKTTLASALCHDPGVKDTFQSNIHFIIVSQFHGNENAVCKILEIMCHSIFGGEKPK

FQSIEYGRNQLQNNLNRNAHPKTLVVLDDVWPGLSVKNLLFTAEGYKTIITTRHNDIIQY

IRGSLYEIPVLNAADALSLFCFWAFGQASIPPTANGDLVREVAAQCNGLPLALRVIGSSL

HSKPLQLWEMVKEKLLKSETISTDHEAELFGRLKTSIDILSEKERQCFLDLGAFPKGRRF

NVGSLLDIWIYVRKMTWLEAFDVLSELASRNLMHLTYDEGNAAIDYHCASEFSFSQHDVM

RDCALHLSNQDSNILCKRLFMPRKEANIPTEWLRAAQSSSAQFVSIHTGAMKEEDWGQIN

FPKVEALTLFFDAEQYCLPTFLQSMSNLKVLIVYNYSSQLVTLSGLPSFPSSVQIRSVLL

YKLIVPSFYENCRSWERLEKLYICRCGDLGNITPVDKDKESEALNFPKLLEINVDDCNES

RELPIKLSNITSLQMLSVTNCYLLSNLPVDMETLSSLRVLRLSGCTGLSRLPPSISKLAQ

LECFDISFCLYLRDLPAGFDHLPNLKTLDTRGCLTLKLPNVKPGSLERVIIFEDEKES

>AB_025106_T.1

MLSFPLASEGNHILHAMDGFFCKKSCSRLDVLLAGLIQTVQPLCIANLKAPKNEQDPKYE

QFLGILLDGVELVKNCEKTSPYNIYQNLRYASKIHQLEKEIRDFVQYQMPLQLCLDVKNL

VAELTSLRHLYESRSVDERRVNETIVPKLTNDPLENATMLQQMGPDMLDGAFDETPCEYN

GYVKTDFVVGLEKNIRNLKKMLLQTESSVIGVQGMGGVGKTTIALSLCNDRDIKGAFQNN

IIFITVSQSPNLKGILDTMWGKIFPRKKPEFQNVEDAHRQLQQQLLRQAKPTLVILDDVW

SRANLENLLFEGQGYKTVVTTRDRSIISAAASTQLYEVPLLEDGDALSLFCFWAFGQRSI

PNNADEHLVKQVQAECKGLPLALKVIGSSLHGAPRPAWESAKKKLVNGQLISEYHKESLL

RCLETSIDVLDEEARECFLDLGSFPEDRKISVDALLDIWVYVRKLDWQDAFVILLEFASR

NLLNLTSNLRSQAINYGSASELYFSQHDVMRDLALYLASRDGLVCRKRMLMPRKEDSLPV

KWELLKDQAFAAQLVSLHTGAMEEDQWCEMNFRQAEALVLVFSASNYFIPTFLSSMKNLK

VLIVFNYGSKRATVNGLPGLSSLTQLKAIRLERLHVPALHEHSKVLQNLEKLSLSLCEGL

GSKSRFNNIQSSLKLPSMLDFNLDHCCDLEELPP

>AB_025142_T.1

MDASTSTSAHANSNHYFDVFVNHRGPDVKKTFASHLYRRLLPYNLRVFLDYEELQAGENL

TPQIKGAIESASVHVAIFSRGYAESKWCLDELIFMLESGVPIIPVFYGVEPADLRWTNAQ

KKGVYAQALCDLEQKRRYDSTIIEKWRTALSSVPETSGLDLKAFNGDEGVLLDNVIECVL

KKVKKTPLNVAKHPTGLDEKAKAFEETVLSKQHSGRVQVVGITGMGGVGKTTLAKELFNR

KSSNYSKSHFLSDVRENAIKSSLLSLQSKLLKGLTHLNLHIDSIDQGIEMLRKHLENMRV

LIVLDDVDKVDQVDSLLPMQDVLQSESLILITSRDKNVLARSGVQESSVYQLTGLNERHS

TELFCSYAFCQPHQPSEFESLVSQFLKACNGLPLALKVFGGLVYGKDKQYWEKQFEKIEK

APHIHKDIQESLKISYDALDEEEKHVFLDIACFFIGEKKDKAIRIWDGSDWSGLLGFQTL

QEKCLVEVDIQNKIRMHDHLRDLGRDLARHPSLPRRLWRGKKDIHDLR

>AB_025210_T.1

MQSDVAEAINLNLESSSNAERRKMKLFTSLKEKKFLLVLDDLWSPIELEERVGIPVRNEK

GSRVIISTRSRDVIRRMGGSNEYSIHIQPLSEEEGWELFCRKAFKDGVPPAKSIEEVARL

IAGECKGLPLAINVLSAAVMGKETVHEWNLSLSDIKNVDPVFVGYSGIDEDLYQRLKWSY

NRLPDSNFQNCFLYCAAFPEDAQIFVEGLVEMWIGEGLVVQSRETQSLMDKGREYVKLLV

ERCLFEYVKLSMEYIKVHDIVRDMAIYVAEKEENCFFRTSQNLQSIPRENGIVNSKRISI

GYSDILPTELRCPNLLALLLSGNESLTQVPNDFFLNATSLRVLDMSHCTKIESLPESMWH

LTQLEFLDLSRTSIKNLPEDICNLVRLQFLHLSDCNKLESLPSKIGELKTLKHLDLRGCP

KLTESPREISQLTSLHKLNMWATGIELKTEEARSGVCSLNDLRNIPNLMELSVEIMPGIE

VDGIRSGIKVGIMSTWLEMRCLRLEFHLDGRGDDVVMEDLPADMHNMKELQKFRLVSYCG

RRLPNCICDFKQLERLELFMCYQLTELPLLERLPNLKSLWIWDCKKLRELGIGRWGSVAG

FPMLELLILHHLPMLESMRSSSSNIVVWSEGMMPNLQRLTIQRCTSLKTVPEEIVKLPKL

KEIGASEEWWENLIWEDENVPKLLKDKLNKLPL

>AB_025320_T.1

MLELMKSDRSTIIPIFYHVKPADLRYGVYAEALTTLQKKKTKDSQTQQKKPRYESDTIKN

WRNALSSLEKDGFDLDGAFNGIESKLVEEVVQLVLQKVEKRPLYVCKYPTGLDQKVEAFE

ELLQQAFEELVQQQSG

>AB_025457_T.1

MATSSSASTSTSHGNTNTNIYNVFINHRGPDVKEGLATLLYQGLREKGLGVFLDREEMQA

GEKLSTQIEGAIRQASVHLAIFSKGYASSRWCLHELVLMQETNRTIIPIFYGVNPSALRW

TQAADGVYARALRKLEERHDSNTINKWRNALFNAAGISGYELSAYNNNEELLISEEELLV

SKEELKKLEEQLVSKVIKRVSEIASKTPLSVAKYPTGLADKVKELQTKVKSAQQQTQNLA

TAVGIVGLGGVGKTTLAKQFFNSHRSNYVSSCFLMDVREKVARNDLNSLQVQLIKELAQR

NEQINTPDEGIEMLKKHLSSLDRGVLIVLDDVDHIDQLDKLFSPVKDTIHSSSLILVTSR

NKDVLTSAQIVESSIYMLKGLNQQYSLELFCSHAFNQPYPVIGFEQLVEKFLDACHGLPL

SLKVIGALLCGKDLTYWEEQFHKISRVVPTDIQSRLKISYDGLDEEEKQIFLDIACFFIG

EKRDTALRIWDGSSWLGRPGLRHLENRCLVELDNENCIKMH

>AB_025629_T.1

MAEIAVVSKATENLTSILVDKLFREVSLLATFRQDFEFVCEELISIKDLLNDVGDKRSSS

SVCSWLDRLEEFLLDAVDTVEECGAGRCKLWNPIFRYKMGRRIRILQERISKIHRSTKYL

ISLTSVLHVNERMQRFNEYNTQDKRERSNAIISESQTVGMEHQIHDITELISHENGPKVI

AIVGIGGQGKTLLLQHVFNIQKDRKYFNHMVWLTVSQKFSVERLLLDIFKQLSKNPKVIK

EMEEEIVDDMKKVSKIRKFTTDSNDQEEVQREVKQKLRDEISKHLEGKRSLFAIDDVWDQ

HFLDSIKLPLESENKTVKIVITTRDRKVVGQVAESHLCHMDGLSPVDSWKLFSIHAFPPN

RRESAPEAPKTPAHNVLMKKCAGLPLALKTIGSYLARVENKDWELKLRSLPEAEAMTDSV

MHSLRLSYEALPDRVKPCFLYCSVFSKNTLIQSESLVHAWIAEGFISTSTQEA

>AB_025634_T.1

MGKTTLADAVFARVYVEGCKYSMVRLFDDVTSTPSTSDIVKLQKYILEDLKMGTPEETTH

EIRTVDEGQREIGRILEKEKAFIYVDNVLQPYPFEQLLPRDMDKAKKLRLLITTRNTTVS

RECSKLKTEIYTMKGLPPIEAKSLLEKEMYNDADNMEKITVSQLDHILEMSGYIPKLLTV

IARFIRCAEDKQEAYSRLKEEGEKNWTGEIGDDISHYVFAYQTLDERVKDPFLDICSFFN

GREWSTVADIVGDSKLNSLEKRALVTKKRTEGRSIETVWVHDVILAMGHQQAEGTRFSSA

KKIQEFLDEKEDKDIQKIKGIMLSKVDETLSISASKLDLMCSSLRVFQLGKWSKVDGKCT

KIFEELIYVAVDIPNFPIDVSRSKELKYLSWEGDLNEELLRRSISKLQCLRVLKLHSCNN

LPKEMGPLNSLQKLHLGMCTSLQALPSSLENLSSLQELHLSNCDRLKALPSSLGKLSLLR

SLVLWNCESLKHLPKDIGSLNSLRTLNLYECKSLEEIPSSIEN

>AB_025638_T.1

MNSIISSQSSSKELRQKLLGLKPTIDEISRISSESDPSCHKGPPLKDIQDKLQDGLHLVK

KLEGISSFNLYRRYRYGKKILQVENSVNDFLLTQALAGILDVHKLSVDSKACEERLKRME

EMGRHIIDSVNAKMTKDAISNSFMLHQMSTGQLSETSIDESYDTTISKEPTSSSNSQVPD

MPNFVVGLNTLVNDVKQSLIFQNGVNVVGVKGMGGSGKTTLALALCNDPQVKDFFQNNII

FITVAHSPNVKG

>AB_025710_T.1

MPIIEDIAVGSAGNVTATAIIEIVKYVKTVAGSEKNLQFLVTDLARMENILSDVRNQFED

QQKTLPTLLQNCLGSIKNALQEVTDLIARAKQHRRRCLGCCFFCHPKLPAQISEWRRTRF

NELFQQLESALSISASTAQIVESAPPRADLLLQPLRESGFVGSGIAESAQKVLTWLNEPH

PQAQVIGVYGMAGVGKTSLLQLIHNKCKEKVSGNFDYVIWFTVSQTFNVESLQNTIAESV

SLDVKGISSTETRKMKLYASLENKRFLLILDDMWSPIVDLNQVGVNFGHDNRSRVLISSR

FRDVVERFAVNKYCIMIRPLSPDEGWELFRIRAFRNGAVIPEAIAREVASECQGLPLAIN

AVAAAMARKETADEWSRALDLMRNVDPSFPSTHRTIDAELYQRLRWSYNDLPNNLKMCFL

YCAAFREDAEISVQAVVEMWTAESLVPQKGRTYFMDVGRGYIDALVDRCLIQYVDRYDDR

YAEKEFEKEFIKVHDVLRDMGIYIGQGDEEKWLFLATQHLKNFPSKEETRDCKRISLMDN

EITDLPAADLQCPTLVSLMLARNRELNEISEGFLVNFMSLKVLDLSYTSIEALPASVGQL

GQLEFLNLKGCERLKDLPDSICNLSHLQFLNLEECGSLNSLPDRIGELKNLKHLHVSGSK

EGFKVIPHQISQLTSLNKLVLRSLSTPMTAEDLSSLSNLIKLNVEVKPEVKGGSMSTWSE

MRELTLNFCGSEDENRDDAALDILPQNMQSMRKLQSLELSFYLGVSLPNCICQFQNLKML

NLNNCRKLKELPALANGSACFPMLETLELNWLESLESIVWNEGTMLKLQYLMINGCEVLE

TFRTEELPNLSNLCITDCVKLKELFIRSDGFPMLESLALHYLPELENVAIPSSVWNEETM

PKLQVLSISNCLLLRRLSMEKLRRLPNLK

>AB_025714_T.1

MLKGLNKHHSEELFCWHAFKQPNPVKGFEQLVQKFLDACHGLPLSLKVIGALLFQERDMK

YWKEQLDKISNILPEEIQSSLKISFDKLDEQEKNIFLDIACFFIGTDRDTAIRIWDGS

>AB_025728_T.1

MQAGKTILSQIEAAIEVASVSIAIFSPGYAESHWCLNELLSMLKSGNVIIPVFYNIRPND

LRRTATNNEGQYAEALWEHEQKQRHSMGTIEQWRKALSEVANISGLELKAFNGDIFELLD

KVVERVLTRLPKTPLAVSEKYPFNEAKYPPGLGQKLQKLENKVLSLQQQKAKVVGIVGVG

GVGKTTLTKAYFNLKSSHYTESSFLFDVRENAQRMSLNNLQRKLIKDLKHIEIEFQSHQE

SIGILRQYLSGCHALIVLDDVDDIDQIDAFMPIKDVLHPKSLIVVTSRDKNAIRIPESAI

YTLEGLTKEQSKELLYSHAFPESTHESPDESPEFENLVNEIVSVSDGLPLSLRVLGALLS

GKSKDYWEEQLEEFHGLLPRKIEDCLKISYNSLNRGEKEIFLDIACFFKGGDRDRAIRIW

GLVGIQNLENKCVLGVDIDNKIKMHDHFRDL

>AB_025811_T.1

MGSENIPDIRKHQDGQRELGNMLQEKRAFIYIDNVLGENQLRELLPKNMNQSKKVRLLLT

ARDENVGIVCPMETAHVKMYHMRGISPQEATNLLKKDIKDDIDPSQLNEIVEVCGGIPLM

LTLVGPFIGHARNKQQAFRRLMQEKGRLKNKPFDGIERYLFAYDDLPDQCKDPFLDICLF

FKGWDWDTVADIVGDSPLEMLESRALVTKNTNGVTSLHDVILTLGCQQEEELRFRFRFTS

ASEMTKCLDRMEEKDIEKIKGIWLSDNKDLFLISAPKLDSMYKSLRILNLGNLTKVVEGN

CNKVFKELIFFQGAVPYLPFDVTELDLKYLSYQPRDLKLFEAKMPPNIRYMELNGRLQSC

DFQISSKDLQQLKNLRILKLTGFAQLTEVAQELGDLVNGLQELNLSYCKSIENLPPSISK

LQFLRVLKMDGCSSLLHLPEDFGSLNSLQELNLQGCTNLRSLPANIEKLSSLRLLDISSC

QELRKLPDGIGKLTSLVNLSLNNCVTLQSIPESIGRLKSLAFSMDMSGCSGLWTLPGQFV

DLGNLRELNLRGCQNFMKLPEGFGRLKYLVKLNLSECINLQGLCNDFHCLSSLRILNLSG

CRQLSELPPDFHRLTSLQNLYIKGCSYLESLPEGFHDLPSLQHLYLCDCRDIKEEWMDNL

VKMKTLQIINIEGSWSLVRRWRKIQREGNHSWSFAVYTGQDLSKDEKENILQKLLSELDC

LLFDMRGKPFNISKLSPNTLSLVMFHSNPDDDFPWPLIEETSEDIETEFQMIYIGKHFNK

VPSNVKERISGYNHENIGSTVVKLLLTLDEGVRESWLLTIDENVRESWLERYNAEEPIHF

LMKMVEGSKDDKCSPCWKVLSNREMEEFVLSKCEKYFKLKQLAETPQESNLRLLTELFAT

REEENIFLRNSASASNKDPSEKPKDFETCGLSKLKGKTIILEMRSADLCNDIRLSSLIEM

YVTEKEQLDLEIISIPIVQSSTQLDVLDAVEQYAKEVPWLVLQNPLTISRAVKYFLIAQW

EWGDVLHYPSIIWIIEPKGRISICKPSSLFMLYTWGPKAYPFFTHEKFEELRKNERNALN

SMSSLKFLFNHLESDPDQVKEAMFQGKMICLFNELDEEVTRSFSNVMTELRDSIHTIYIP

RYRYTDMEMLRVTDTIYKSYYLLENVELPEASEMEMEGMSVINLSEHEAMRFWMRLLYLK

EEIYGMEDGGDKKLCERKRFSPYFQLIDEKDDRHGGCMIFMEEDGKMVAACRGETMKWIF

RNNNEDRAKQLIKRLVKVSKEEGKQTDDDSASVLDLTKKMQTADQENVDVDLDSLSKYIS

ELLEVEKLYHLLEFKKERKKKSRPVGAGHNWPTLKNLLTEGESGFIDNSSLHGGEEDVLH

LFMIFLKSLLKEIGKAQWAAAGLQVVAELLEKFETLTANQNEWLLVLEAMYRLAIYLK

>AB_025921_T.1

MASSSTSSSTAASPSINSNSNYDVFLNHRGPDVKKTLASHLYYRLRDRGFQVFLDRQEMQ

EGGNLNSQIKGAIATASVHIAIFSPRYAESTWCLDELVMMLQSKGTVIPVFYHVKPSDLR

IRTSKDGVYARVLTNVGWTEKSEEYANALRIHEKKKRYEPKTIAEWKQALFSASLLSGVE

LKEYNGDEGELLDRVVQLVEDKVRKPELDVAKYPAGLDEKLQDFEETILQQQRHRRKTKV

VGILGTAGIGKTTLAIEFFNRKRSAYHQSCFLQD

>AB_025984_T.1

MVRGEHLRKLLPNESYNKNFRLLLTARDESVADVLSACGIHTCVYPVRPLPYQDARKLLC

QKMGVPGRMQTPPQINEILEICDGVPLIVERVGAYICQSQDKDEAYKRVIEWSKEGKPFS

SAEEYNIEKKGLLFDLDELPPSARETFLDICSFFNGWDWNQVSCMMGEDELNSLRKRALL

LKKEECENKVMVRLIILTVSRNMTKGKRFTSAHELSKVLEGNELDDIGGIKGIWLEDNIS

EPLFISAKKLDLMHDSLRVLALGDMAIVVDGQCTQKFKQIRYFQAGLIPCIPFHPKTSQE

LRILDWLPHNNNDLQLRKMSSKLKVLNLNGARYDRRFEIENVFERLRGLKTLKLTQFKTL

KNLAEELRLLTQLEELDLSGCTIAQLPKGLGDLNALSKLSLRSCRKLRELPDSFEKLRSL

KYLDLHSSGLKQLPKDFGSLSSLEELDLEHVDLKELPSSFGKLSSLRVLNIKHCSSLNHL

P

>AB_026138_T.1

MEVGTGAAGGVAADIINKGLVAPIIRKLDDLINMDTNNQFLQVKLQRMKHLLQDISNDFE

NQERVPAQSITYCLGRMRDAIQEATDLSARFQRKQNSPYWLISKASISGQIAELNAKFDQ

LIHDLDTDFRIFQHAQQIVSASPLRSSAPLQSPALGQPSASAPPQETDVLLQPVPDGGFV

GSKIKSAETRLQAWIDAESPQVRVIAVFGMAGVGKTSLLRRVYNSYKVGNVFSVVIWVTV

SQDYQIFNLQGQIAEAIQLDLSAISNIDTRRMKLCAKLRNEKFLLILDDIWRPLDLEQGI

GVGFGDDKASKVVLSTRNRDLASTEAHKLLKVEPLSMEEGWELFQRVAFKDGHVPEQLKE

CARDFADECEGLPLAITVVAATMKGKPPFVDEWNTCLSLMKIADPSFTDTHPRVDKQLYR

RLRWSYDDLPNSNLKNCFLYCAMYPEDWHIWVDELVQLWIAEDFVKSKIGTHSTDFDLGY

RYVKLLIDRGLFQSVNFEKRRMDIIVNKPVDAWKRRYVTVHDVIRDMAIYIAEEEENYFC

RGGQNLTEFPESQTQSCQRISLLTNQIEYLPKEFICPRLVSLILYKNPLTCNIPETFLIN

LTSLRVLDLSSSRIQSVPTSLTQLTSLKFLDLNNTDIKELPQEISNLSELRFLNLCRCIN

ITSLPYTIGKLTQLQCLILEGCYNLVLPAEISELTSLKRVGLGIGTPKEVLKDPTNVIAL

NGLTNVKKLSLGIFLEFEAHEQIGTWLEMRHLRLSYNNYMNNTLPHGLQNMKKLESLVLN

NIIPVSLPNWICEFEQLERLELSDCGKELPSLERLPNLKFLKLTECNELIDLGIGSSQKP

GGFPMLETLVLKYMHELRSITCPSCEGGALQEGTLSKLCILKIKHCPALTTLPMEMEKLL

NL

>AB_026182_T.1

MLRSYLKKKKFLLILDDMWSSLELQSLGVSLNDKDYKIVITTRNKAVCTSMNVEEMIPMD

PLPADEGWQLFCSRAFQNGNVPADIEDVAREIAGECKGLPLAIIVVAAAMKHHPYRHEWE

LALQQMQAIDETFYDTHEEIDEKLLQRLKWSYNVLPNNLKTCFVYFAAYPEDRVINCEEV

IEMWIAEGLEGLVKSSEHSYLHDTAHSFVSFLRDRCLIVATKTDIAGRIEQVKIHDVLRD

LATLIAHKEQKCFFKVGQSVSVFPIEEQSKGWLRMSFMYNSLRSLPEAFACSSISVLTLK

QNAGIKNVPGSFLQGMPSLRVLDLSDTHITSLPSCIGDLKHLVSLQLYRTDIRELPESIG

DLNKLQFLNVGFCGQLGYLPKRITELKCLIALDIWGCKRLSFIPRGISELVSLERLRMKM

SIPLDFEEAPDANAERSYAWLKDLQSLRRLRNFAVEIKSRVKDGVIGNWSRMRDLWLDFN

LANQDYLPDDMKAMKDQLERFCLCRCNVELLPNWVREFRKLSYILLKSCKQLKELPAELY

ELPGLRGLSVEECDSLKELELERRGCFPKLEKLTLQNLKALESLQSSSGGLGEGALPMLK

SFTVKKCEKLKTLPLGWDKLKCLEEIRGERDWWNAIQWQDVNLKTYLETKFRSV

>AB_026190_T.1

MEKGDFLTSQIAAAIRTASVHIAIFSPRYADSEWCLNELLLMLESMDSGRSTVIPVFCRV

KPADLRWTLGENGVYAKSLTSLQKKKTTDSERHRKKPRYQKDTIKEWRNALSKVSHINGF

DLEEAYNGVEWKLVEEVVQLVLQTVEKPPLDVSKYPTGLDQKVKAFEELLQQQSRGPRVM

GIVGLGGVGKTTLA

>AB_026198_T.1

MEASIREATDLIMEACITCCTQIGYSKGRRLLSASGDKEELVKFQQKLQRMYKHMDNKMS

ICLYDATECRKVRPQKPRERSYPDYAVGIEKPLEEVKKLLDWESEKKAVAVILHGFGGMG

KTTLADAVFARVYVEGCKYSMVRLFDDVTSTPSTSDIVKLQKYILEDLKMGTPEETTHEI

RTVEEGQREIGRILEKEKVFIYVDNVLQRYPFEQLLPRDMDKAKRLRLLITTRDTDVSRE

CRKLETKIYAMKGLPLTEAKSLLEKEMYTSYTDNMEKQLSSTQLDEIVEMCGYIPKLLTV

VAGFICCEEDKQKAYIRVKEEREKNWKGEIGKDIAHYVFAYQTLDEEFKDPFLDICSFFK

GWEWSEVANIVGDGKLNSLEKRALVTKKRTEARTQFYRNETIETVWVHDVILAMGHQQTE

GTRFSSAKKIQEFLDEKEDKDIRKIKGIRLSKYEDCLSISASKLDLMCSSLRVFQVGKWS

KVDGKCTKIFEKLIYVVVDIPNFPIDVSRSKELKYLRWEGERDLNEELLRRSISKLQCLR

VLKLHSCNNLPKEMGPLNSLQKLHLSNCDRLKALPSSLGKLSLLKSL

>AB_026203_T.1

MAQGNTIGHDIGTGAAGGTLGGVAADFLNKGLVAPILQQFDDMINMDRNTKLLQVNVERM

RNHLQHISNMYEDQQRVIPENIRSWLRRMGVAITEAIDLIKTFQRVQVDLEELKIYRQVR

EMYTKFENLLRDLDTDFSVLRAQNIVSFAPLRADVLIQPMPTAGLVGRAIESAATQLQTW

INAVNTPPRVIGLYGMPGVGKTTVLGKVHNFYRARNVFDVVIWLTVSRDYQFSALQGQIA

EAIGLDLSSISNPDIRTMMLLESLKKKEFLLILDDVWSSLVLKELGVLFGANECSKVVFS

TRDRNLAVTEANESTKLEALIMQEGCELFEKVAFSGRPVPENLKERARKIADECKGLPLA

ITVVAKAMRGEKTVDEWDDCLSMMKATDPDFRNTHPCVQDELYKILTWSYNEMGKTLGLQ

SCFLYCAMYPEDEEISVEDLVRMWIAEGFVQSKKGSYSESMKRGYRYVKLLIDRGLFESV

NFEKQSKDTNKPVNVWKERFIRVHDVIRDMAIFIAEGNNYFCYAGLELKKFPSSQTQACK

RISLRRNQVKSLPEDFPCTQLVSLMLSENPLVSLPTSLTHLRVLDLSNTLIESVPTSLTH

LRFLDLSNTLIESVPTSLTQLEFLTMRHTRIKELPKDIGNLSNLQFLDISECKYLTSLPL

EMCQLTSLK

>AB_026320_T.1

MADPGFLSGFIGVAIDTAITQILYHVGIAVSFKQELESLRELVTRIQPMVTDIHQCRLEL

NKKRETSTGGSDVNSWVTYLQKRLQEALAIVQECASIPRSAVCFRYQSSKRIITLISDID

KHLKDLPILLLQQQQRQTQMLIEIRSESTQASTASTSATGTSEVLAPTAGGFLIQEPHIV

GQKKYFTALEKRVIDADVEPLFRMGVLGKGGSGKTLLLKRLFSTQKV

>AB_026333_T.1

MANPLASSSNSSGNNSPYEVFINHRGPDTKESIAGHLYSSLTPHGLRVFLDKEEMEKGDF

FTSQIAAAIRTASVHIAIFSPGYANSEWCLYELLLMLELMESDRSTIIPIFYHVKPADLR

YGVYAEALTTLQKKKTIDSQTQQEKPRYESDTIEKWRNALSKVSYIDGFDLDGAFNGFEW

KLVEEVVQLVLQKVEKPPLDVSKYPTGLDQKVKAFEELLQQQSGVLRVMGIVGLGGVGKT

TLATEIFNRLRSNYKQSCFLFDIRENSLTTLQSHLLKDLSQSNVQIRSIAEGKAMLRRNL

SSKLALIILDNADHAQQLEALLLPAKDVLHSNSLILVTSRDR

>AB_026366_T.1

MTEAKKVRLLITARDINVRKACRVKTAPNEYRMRGISSMEAKNLLRVEMFDEVERKLHSY

KVDHIIKKCGGIPLMLKLVAKALHVAKDTQEVDEVLDELDKLKGEHFPLDEIESYFFAYE

KLPRECKDPFLDICSFFDGWDWDIVANIMGKPELEMLARRALVTKHTSGVVSVHDVILTL

GRRKSHGVRFRFTGAIQMKKFLDEEEEKDLHNIKGIWLEENRDLFTISAAVLDSMHESLR

ILKFGNLTKIEGECNKKFKDLIFFQGAVPYLPFKTKELKYLSYQPKDLKLLEIPRSLRHM

EFDGRLHSHAFEISSRELEQFQNLRVLRLTRFAKLKKLGNLVNILQELTELTLSYCQSIE

ELPSSISMLPFLRVLRMDYCLNLKHLPEDIGSLTSLQELYLQGCTSLEVLPKSFEKLSLL

KSLDLSSCEKLSELPQGMRKLTSLVNLSFNKCSRLTSIPESIGGLKSSTFSMDMSFCSSL

KEFPDLFVELENLRELNLCGCSELMKLPKSFSQLKCLVKLNLSKCISLERLCDEFHHLSS

LEILDLSGCGSLQELPQDFHCLTSLQNLYLSGRNRLKELPQNFHCLPSLQHLYLCDCSLE

GKCMDNMVEMKKLQIMNIEGSYYLEKRWEEIQMQGNQSFSFAVYTGKDLSKEEKENILEK

LLSELDCLLIDKRGNPFHICDLAPNTLSLVMFHSDPNDDFPCPLLEETIDDIQINFKMIY

IGRHFNQLPGKVKDKISGYSREVPKITLMVAKFLLTLDADDRKSWLSTLEKNVRESWLER

YNAEEPLCFKMEMIGDGKDGKNHSCWKVLSKCEMEDFVLSNCGRYLKLKQLVETSQESNI

VLLRELFATHDKESNSIFQRNCTSNKDPTENSQPFVCGVDNLKGKTILLEMRSVEDSCND

SGLWTMCEMYLRDKETLDIEIISIPIVRPRTIVDVLERFSRKVPWLVYQNPWTVIRAVKY

FLVKQCTDWERERDDRWNPNIFWIIEPNGKISLGNPYMLLRWNNWGSNAYPLTYEKLEEL

RECEWTQWQNMSNLEFLFSHLDSTVTDQVKRLMFQGKLICLYAGKGKLEVTSAIRNALIE

LRDKIEIIYIPLFDIERTETLKLTDNIYKDYFVLETSEILPVEMEGMSLLNLSQHLSKHE

ILRFWMRLFYLEKEINGMDGSDNQLCEMKRLFSSFQLMDKDEDFGSFQFMDEDEFAAVDK

ESVMFLSLMKKEILEVGDGGMTFMDEDGKMVAACRGETMGWIFKDNNRAKRLMQLLVKVS

RKEGKQKDQYSSVPDFPENMKTAGAAADQENVDVNSLSEYTSELLGMERLYGLLKFNKEG

GKSPRPKAAAPHYWPTSRGFSMLKNFLSDGTNANWVKNIILPQPKEEDEEYHVTVISEDE

EDPKEDEGDRGSIMSPRPKEDEDEVDVDDDMMLKAIINSLLEGIGEANWATAGFLMFADI

LERVESLYANREEHLRLLVLLHEMYRSVMCLEQSNQMHELMVWILMAALSCCTGTDHSSL

LVRLYFTFVYQFDDWNLESRLPLLQGLWGLIYRELKRVCDWEFFLRHMREARFSPATSSG

GSDNPATP

>AB_026398_T.1

MHLPACPLLGLFFFLLITMIFLLLFVLFPPIPLSLHRLFIVVTSCSGCQERFTIPFLMAP

GREWIASSLRLVEGGNITSQIKTAIQVTSVHVAIFYVNYMQSNWCLDELDLMAKSRAPII

PVFYKVKPHPLPGFVDLVYKDM

>AB_026403_T.1

MGKRICCCICPCFSAPDEFEAVRTDPNSVVAVSGKGDSSSETGGPSAHHDHDSANSNNKR

GGEGCSSSVSQEAPSGHAHVTDGHGGEDYANNSNSVVGDSAEGGSSSETGGPSAHKGCSS

PGSAHAHAHGGQDSSSSSNVNLDDLIKHISQRLDQIKEKSPPQRQQTAHYIWPTNSPGLL

KLQEVLSRGIDFASAVKDVIPSQHSTEAKAVVLSLLKGIGKAHVVTAGLLVVANILERFE

DVSDNRAECLSLLDEMNRLAKHVKQLKERPGLRQEMENIIEEAVELIVEASLMCCTQIDS

STFSRFFTTSVDKGELVEFQKKLKKTYKHMKNQMAICVYDANVDWRPLKPRERGYPNYAV

GIEEPTQEVIKLLEWESEKNAWGVVLHGFGGMGKTTLADAVFARLYKEECKYSMVSLFES

ITSTPNIVELQKFIVKDLMVGTAAAEVDGLLANIATYSDGQREIGRLLEKEVAFIYIDNA

LEIDPLEKLLPWDMSSARKLRILMTARDTNVCRGCRIKATIYDIRCLPDTAATTLLEKEM

RTLEQLNRPQLTQLVKICGGVPKLLSLVARFIFCADDKQDAFRTVMEEQEKWEGQIGEDI

NRYLFAYKYLPDSSKDPFLDICSFFNGWQWDEVASIVGKPALDVLEKRALVTRGTNMTAN

VHDIILKVAQQSEKGMRLTFTSASEIQGFLDNTDKEDLQGMKGLWLREIKIEDPLQISAK

KLDFMHRSLRILSLGDITVEGKCYKKFENLVFFQSGLGSIDLPFDVSKSEELTYLAYHQP

EELNVYKMPEDLKKDAIDRMRRSQVLQISSRCLRPLRNLRILRLIGLTQLEKLPADLGDL

NGLHELTLSRVPCIEELPPTISKLQSLRVLRLDRCSRFRKLPEELGGGLVNSLHKLILSG

CNLIEELPISISKLQFLKNIEMDSCTKLSRLPDDFGSLKSLQELNLSQCNRLVAFPSSFK

NLSSLRSLNISGCESLQYLPPDLGSLTSLVELNLSFCKNLISIPDSIGQLKLLAFSMDIR

YCESLTQLPDQFCQLSFIRHLELDGCHRLKKLPDAFGKLESLGVLSLFHCVGLKNLPEGF

GQLKSLVELNLSDCSDLEELSVDFHRLSSLRILDLWFCKDLEKLPEDFHCLESLEVLSLD

GCDGLQRLPEGFGQLGSLRVLSLRWCINLKKLPEGFGQLNSLVELNLGFCDRLKDLCIDF

HCLNSLEYLRLGQCRKLQGKWMDDVVKLKALGNLDVTESPMLVQRLTEIQRECHQSWRFI

VETGQGLLEESNSKNREDALSKLSSKLDTLLTDGVGETFNVSALPLNTVVLVMFDGDRKS

RDASALVEYAIKHRPINFETIYIGRHFNSLPRKIAHRISAHAACSSEVLLVVEKITSTSS

LTDALFSGWWESYFQMSAKIVEDGKGGKYLSCWEYFYNLYEGIISKEFVLSNCEGYLQLK

QVEETIQSSNINMLRELFACGEKEETMATDIFLKDYGAHKVGVDKLQGKIILLHLGPLKE

IDILRDVLTEGYVTEKERSNLEIISIVKGSRPTEVERFAKEVPWLVYPRTITKAKRYFLY

QKCSLKWKSEESGWVIQESEYSTAIMVIERNGRIPSYEALPLRTILDRWGWKAYPFTEEK

IEELKEEEWNDLPHFSPLMFLVKHLGEVKERMLSQKMILLCGGVKDEREMTEFTSFLRDE

FKGQWDSIHLIYVPRVYKCKVSEVAMRDAEMEGMTLLTLSKHDTIRFWERVLYLRQEIVG

MDKQRKGVGTMKTLLNFCDQCIDWATLMGKDGELISYMQQTITRTRIMKKHEEA

>AB_026439_T.1

MLLKEVAFLYIDNALDKDVLEKLLPKDMEKFKKCRLVITTRDTEVRNGCLMDEVKLYPVK

YLPNTEAMIFLKQELNYEKTDDRKRLNSGQLHRIVNLCGGIPKLLSVLARFIRYEDRGQE

KAFRLVMEEMEEKKKWNVRIGDIGSYLFVYDLLPEIVKDPFLDICLFFNGWDWDKVSDIV

GESNLDVLEKRGLVKRDKYMTVRVHDVILTVGYEKTQGTRFRFNSKSEIEEFLKKDIQGI

KGIWLNDIQGINHIRLNHIHTISIPATKLDCMSHSLRILALGDSAEVDGTCEKIFEEIVF

FKGEVPPFHVMPSNLKHMELNGRLHSEDAFKILSSDLQQRRSLRTLKLVNFAMLKNLPEV

WAILSHCLQNLYLSGCYSLEELPGSISKLQSLRRLDMSNCSSLKRLPEDFGSLTSLQNLV

LSGCNSLEELTGSISKLQSLTQLDMNDCSSLKRLPEDFGSLTSLQKLHLSYCNSLEELPG

PISKLQSLTELNMWNCSSLKRLPEDFGSLTSLQNLVLSGCKSLEELPGSISKLQSLTELD

M

>AB_026487_T.1

MGMAHLACTGLLVVALVLERFDTVSTNEEECLCVLDEICKLGKHVNDLKQRPRLREAMLP

SIREATDLIMEASIMCSTQIGSSKGRRLLSASKDKVELVKFQQELQRIYRHIDSKMGICL

YDAAECRQVRPQKPRERPYPDYAVGIEKPLEEVKKLLEWESDKNAVAVVLHGFGGMGKTT

LADAVFARLCIEECKYSMVRLFDDVTSESSTSDILKLQRYILKDLNMGTHEETPAEIRTP

EEETPPTPAEIRTPEEETPPDIRTPEEETPPDIRTPEEGQREIGRILQKEKAFIYVDNVL

QRYPFEQLLPRDMDKAKKLRLLITTRDTDVSRACNKLKTEIYRMKGLPHTEAKSLLKKEM

YTSYTDNMEKQLSDSQLDEIVGMCGYIPKLLTVVAGFISCAEDKQEAYSRLKEEREKNWK

GEIGDDISHYVFAYQTLDERVKDPFLDICSFFNGEEWSTVADIVGDSKLNSLEKRALVTK

KRTEGRGLYTKGEIETVWVHDVILAMGHQQTE

>AB_026547_T.1

MDASTSTSAHANSNHHFDVFVNHRGPDVKLTFASHLYRRLLPYNLRVFLDYEVLHAGENL

TPQIKGAIKSASVHVAIFSPGYADSKWCLNELLDILESKAPIIPVFYNVEPTDLRCTHAQ

SQGVYAKALCDLENKRTDDGQLRYSSTTIGMWKGALSRVAETSGLELKAFKGDEGALLDK

VIECVLKNVKKRPLDLATHPTGLDEKAQDFEDKVLLKQHSGRVQVVGITGMGGVGKTTLA

LELFNKKSSNYSNSCFLSDVRENAIKSSLHSLQSQLLNGLVQLKPPAAIDNIYQGITMLK

THLEKKRVMIVLDDVDNVDQVDSLLPMQDVLHPESLILITSRNKNVLARPGVQESSIYQL

TGLNERNSRELFCFYAFGQPHPPSEFVSLVHQFLNACNGLPLALKVFGGLVYGKDKQYWE

KQFEKIEKTLHKDIKESLKISYDALDEEEKQVFLDIACFFIGEKKDMAIRIWDGSDWSGL

LGFQTLQEKCLVEVDIENKIRMHDQLRDLGRDLARHPSLPRRLWRGKKDIDDFRQHSSVH

GIANVRGIRWTNQFNGESSYLTRKWSQLTEEVKVTKLQLLDVKVRRLQLLDVDILSENFM

RGVDSPNLIWLRWESCHISSLPPWIPMENLRVLEVHGSDLETLWEGQGKSEVPSQLRELR

IFAPLSKIPESIGRLKHLERIEIRHSNFEDLPTNFGRLRSLEELHISECVKLKSIRGSAE

LIKLRIVKVSYCRNLEELPGFENLRSLEELYVYRCLKLKSIRGSA

>AB_026557_T.1

MASPSMASSSTLASSYYYEVFINHRGPDVKNTFASHLYRRLLHCGLRAFLDREEMEKGDK

ISPQIEHAIRGASVHIAIFSRSYAQSTWCLNELVLMKNSGATILPIFYKVEPSVLRWTVE

GAYAESLRDLQQKTIHDSETGEEKPRYGFKTIQDWRNALKDVADLSGFTLGEDDDEGELL

EKVVETVLKKVPKTSLDVAKYPTGLDLKLEKFEETVLSQQQGTEHLEAKVVGIVGVGGVG

KTTLAKEFFNRNRSCYAASSFLSDVREVAGRMSINYLQSKLINDLKHTDIKIDRHEGIEI

LKRHLSLCQVLIILDDVDNVNQMDAFLPIKDVISPNSLILVTSRDKQVLKSSGVLESSIY

NLEGLDKTQSRELFCSYAFNQPLPLPPPEFPDLVDQFVAACKGLPLSLKVIGALLSGEND

KCCWQDLLERLNHTLPEEIFETLRISYDSLDKQDRSIFLDIACFFIGEDIDKGIRIWGKA

GVKTLENKCLLEVDTDNTIKMHDHIRDMGRKIAEERTLPCRLWDSTTNSIDNFLEQPSTV

ITEVRGIKMVTTSSDDFRYTGRNWFGFYNYGSSQISQSKYLFSNSHFLCMRNLQLVATEE

DQLEDILRRVQRPHLIWLRWYRVSHSSLPDWIPMNNLRVLEVEGNRLNTLWSHQSQDQVP

LHLRELNICAPLTEIPKSIGQLKYLERIAIYCPESLRALERLPDEFCRLGSLKYLVFKGF

SEMVSLPHSFGELTTLQHIDLEEASSLQTLPDSFGKLTRLKHLSLSHCRSLTISDATLGD

ITTLEYLNLSCCEQVNELPPQVARQLSLQELKLVGTMLKELPNLCCNLEVLHLGGDLLEM

LPPNLGELNCLKMFFFN

>AB_026618_T.1

MEQVTEAASYEVFINHRGPDVKRTLASLIYSALGNCGVTAFLDSEEIRTGDTLTPAITAA

INSASVHVAIFSERYAESSWCLNELDSILKVSDEHNTKIIPVFWDVKPSDLRYIESGRYA

DAFKNHKQKRRVDLEDLEKWKTALGKASHISGLEVKTNESDYGKIVNKIVEMVNEEIFKR

VALKVTKFPVGLDLAAKDFENKYLKSGHSNATTIVVIAGIPGVGKSTFAKHLYNLRRPHF

RRSSILSEVRSHLELRYLQEQLLHDLLHSQSLPRISSISQVKQILQQRLPRLPPVLVVLD

DVDDIAQIERLLDVESVPSGSLILITSCDQDLLRHSSVKTEVHVLKPLDKNYARELFCLH

AFSPSEPPEPPEELKDLVEEFLNICGGLPLSLKVLGGLFVGCRDKVYWERQLEKFSTTLP

NSITNTLKISYEALDPKEKEVFLDIGCFLVGEAEELAIHVLEGLYEDNTIGECLQSLQKK

CLVGFCDRTRKMPSVDGRRKYYIECDDTCKSITMHDQLRKFARHIAREEFRVFPARNPLR

LSSPSDIHEVCVHSQAGSTLQVRGFRLSTPPRRYNKHPALSKGLTYHFLRDIAVKVEAVK

VEGVRLLVVDYERPCLAPIPTSNLWGELVWLRLHGDSKWFYEYSISLRRLRVLEIQSTGS

SVEGFFDHHHNEVRTF

>AB_026641_T.1

MGKTTLADAVFSTIFQQNYTSTNICRYSKIRLCEDKKSSLSTITTLQKTIRDYLVDHDDQ

RSAHISTFEDGQRQIGNILKKKSVFLCIDNVLNKERLDQLLPLRSTLDQAKKLRLLITAR

DKGIGTVLGHNMSCKFHEVLASSSSQAEKILKKHMDTKKLDKGLVDDIVRTSGGVPLILS

VLGQSIGAEKDKQRAWRIFREERESWSGEIFDRLDIAYKHLPDDLKDPFLDICSFFNSEW

YWEAVEDIVGISILDRLQKRGLLTKDLNSMLTIHDVILRIGLQRSKGSRIMDANEVSQLD

GKGIKGLWFTTDQGREPIPAKKLDQVARSLRILALQNLKVEGDYNENFPELIYLNAGMNP

LPSNVSAFKKLCYLSYSPQKPEDLNLSQMPSKMKRLKLNGKSYSRLFRRCSLDLHGLQNL

RVLKLLEFKHLTKLPKEFAFLKCLQELQIIDCDGFEGFPTDLGDLNQMVTLTIEKCPKLR

KLPESVSKITSLQILKLPHCAGLRQLPEDFGSLSSLRTLDLKGTGLKMLPSSFAQLSSLR

SLYLSSCNKLEDLSKDFHSLPSLQYLKLSSCPMLQGQWMDVIKKVKTIKEVDIRKSEKLL

QKWQGITNPPRTFSVKVN

>AB_026655_T.1

MGIAGPRGVGKTTLAKTFFDSKRSEYSGSCFLSNVKESSRDSLNRKLKEELPQNDTIVEG

IYKILKLIPLSCIAKILKLIPSSFSHRKVLIVLDDLDQADQMDVLSSHSHRLGSNSLILV

TSRDKNVLEGRIRKEAIYNLTGQKHHTPSPRTNLILAGSWLEELPAWLTHLTSLKELWIR

DSPNLKRLSSGAFQLPNLPVSYQLLNRLTVLTIMDCDIRSLLQELEKMNNLQVLRLCNCP

FSELSFRQVQIPTKVLLLRESRRLDGPSKTKAPFPVGVNCHFQHLWINSCRDLVEVGSFP

YTLKTLDLRYCNRLRKIAGLRDLGNLEQLDISGCEEVEELPGLESLKHLESLKASRCYKL

RSIIGLTHLIKLRELYVRQCHEILHLPGVEHLLCLKKLDASGCLKIQWTTEVLEKLRERL

NEDFSYDMK

>AB_026741_T.1

MAKKSCCSCATFLSCFSRNTTDASPDALSFSAIVNNNVDSTTVIAEAGAGSRPDEIQTPP

LNPDTSFSHPPAAAASSSVEGTSSAAAPKASLNSNAASANGNTNAASPLIDTQEALDVDN

IPKDAAAVEGDNVEVDLDGLIKHISQLLEHIKDEGKSSSLPKLVHRSYLPTSKGFSKLRE

FLTEKDIIPGDAKTIVRSLLQGMGTEHLAAAGLLVVASILERFEDISNNRDECLHLLKEM

IFLAKVVKQINERQQLQERMQDEIKDATELIVEGSIKCSSQMGFSQFSKFFSSSVNKKDL

GELAQQLDVKYKHIYIQMGIEIYDVSQCHKANLSRRYPEHAVGIQEAIREVVDLLEWGSE

QNAIAVILHGFGGMGKTTLADAVFSVLNIEGCQYSMVPLFDNVDSPPKIIELQKLILKDL

MGSENIPQIRKHEDGQRELSRMLEEKPAFIYIDNALSERELAQLLPEDMTEAKKVRVLIT

ARDLNVSKACPLKTVREYRMRGISSVEATNLLKMEMFDIDDMEGILHSYKVNQINHIIER

CGGIPLMLKLVARALLDAEDEQEVDAVLDELKKLKGEDFGPDKIESYFFAYDKLPRECKD

PFLDICWYFEGW

>AB_026804_T.1

MDVRENVAKNDLNSLQVQLIKELAQRNEQINTPDEGIEMLKKHLSSLDRVLIVLDDVDHI

DQLDKLFSPVKDTIHSSSLILVTSRNKDLLASAQIVESSIYMLKGLNQQYSLELFCSHAF

NQPYPVIGFEQLVEKFLDACHGLPLSLKVIGALLCGKDLTYWEEQFHKISRVVPTDIQSR

LKISYDGLDEEEKQIFLDIACFFIGEKRDTALRIWDGSSWLGRPGLRHLENRCLVELDNE

NCIKMHDQLRDLGRELAKNSEYPLRLWTLRDSFDDLYNAFQIQRPNSQFIPHLSFVRGIH

TFHTNDAKISFEDLAASCNMSRLQLLWAEGDFVIRLFQVPHSPHSPQLIYLHWDYCPYSS

LPTSIPLKNLRVLYIQRGFLKTLWRHESQAPLQLRELVLNAEIRRIPKSIGKLEHLEKFV

LTSECIKTLPDSLGNLRNLQELNLSG

>AB_026805_T.1

MGGVGKTTLAKKLFNEKISDYSNSYFLFDIRKNYKSSLLSLQSKLLNGLTHLNLHIDSID

QGIEMLRKYLEKMRVLIVLDDIDDVDQVDKLLPMKDFVNVLQHESLILITSRNKNVLARP

GVQESSIYQLTGLNERNSRELFCSYAFDQPHPPSEFESLVHQFLKACNGLPLALKVFGDL

VFGRNKQYVEKLFEKIETALPKVIKESLEISYDALEKKEKDVFLDIACFFIGENKDMAIR

IWDGLLDFQTLQEKCLVEVDIENKIRMHDLLRDLGRDLARHTSLPRRLWRGKEDIDDLRQ

HSSVHGIANVRGIRWINQFNEDDGDEGDDTEEEDGDEDDDAESSYLTRRLPLTGEVKVSG

LQLLDVDILSDNFIRGVDSRNLIWLRWKSCHFSSLPSWIPIKNLRVLEVHGSGLETLWKG

KLKAPSQLRELRIIAPLSEFPESIGNLKHLERMVIRHSNFGFRDPLSKNLRLNGSFKMTP

LPESFGNLTNLEHIDLDQCTLLERLTNSFKDLSKLRTIKVGDCRELEELPGFENLRSLEE

LHISECVKLKSIRGSAEKLRIVVVYDCPELEELPGFENLTSLEEFHISECLKLKSIAWLA

ELTKLRILNVRDCPELEELPGFENLRSLEEFHISECVMLKSIAWSAQLTKLSIVDVHHCP

ALEELTGFENLRSLEELHVFGCVKLKSIRWSAHLCSWMCEAEELTGVGSADRASNSTCY

>AB_026817_T.1

MKEYNIISFRFNPIFHFRLQSFVPTICSSTSAYSLLFQLFALQFQYPHNSHLLFAILRIF

SGTSAHPTPMVEITEITAVARHNDFASSSTASGSSLSPFDHYDVFINLCDYNHSGRDVHT

FATKLYDQLHFLQLQVFMDKPDLLFNNNILPSKIEHAIIAASVHIAIFSPNYAQSKWCLD

ELVLMTKSSAKSRSTIIPVFYYAKPSELRRTGKDHEGGYAKALCSYENQNLYDSLTIRKW

RDALSQVAEISGFVLDDGHGGEEDLLNNVVQSVTSDFPFGARYDVFINHRGPDVKDTFAR

SLYDHLVSRGLRVFLDKPELEAGIDIVPQIEVAIQFAYVNIAIFSPNYAVSHWCLDELVL

MVSSMNTILPVFYEVSRSDVLSPDDGVYKKALLEHERLQRCDSETLQDWRDALSIVAKLP

GHKLDTCGSSLILDPELDRKKKELLNKTVQSVLENLAKRSEAVRNSEVQTSELMKIVLKE

NVRKCNVRKCAVGGAGNVTATTIIEIVNNVKTVAGSENNLQFLITDLARLEDILSDVRNQ

FEYRTPPQFLQNWLEGMKDALQEVTDLIARAKQHRRRCLRGFLFCNLKLRAQITEWRRTR

FNELFRQLFVPMSIFEQIGVGVAPKLASTVIVELVKYLKTIDSNKNLKFLITDLLFRLED

MEDILSDVRNQFEYQQWTPPQFLQNWLEGMLEGMKDALHEVTYLIVGAKQQISERRRTRF

NEICQQVEYALSISARSISAKLRKNGLVAKHNLGGSQCQNQAYSALRIN

>AB_026939_T.1

MESMKQESKRKESYLWRGYPTRAVGIDKSITEVLDMLKCESEEKAVTVILHVFGGMGKTT

LGDAVSSRVGGCKYSHVQLFEDVNAKPDIKDLQKKILKDLMGTEEPVPEIRKYQDGQQAL

ELVLQRVEAFIYIDNVLEGNALKNLLPDMGTAKKVRLLLTARDENVTKYCKRLHTNPKIY

RMKGIPPPDAMNILKREIKEEINENQFKDIIEMCGEIPLMLILAAAIIGCADNKQEAYST

LMLDRGKWEREEFEELHYAFAYEALPDKCKDPFLDVCSFFKGWDWETVANIVGEKELRML

RSRAVVIKGTNGAVIIHDVILSLGLLKAEGTRFTAATQIKKLLDQKDEKKFEGVKGIW

>AB_026994_T.1

MASTSYYSYRSNDPVFDVFINHRGPDTKETLAKDLYDQLKHSLRVFLDYGELKEGEEITP

QIQSAIRTASVHIAIFSKRYCESSWCLEELFLMLQSGSPILPIFYGVKPSELRWGTQGEN

GVYAQALHTLAKQRTFDSQPRYDTSTIEKWRKALFDCAEISGFDLETFNGDERQLLQRVV

ERVLKIAKIPLHVSEYPTGLDEKVKDFEGRVSLQQQSSETRVIGIVGLGGVGKTTLAKEI

FNSRRSNFKKSCYLSDVRETADRSSLTSLQGRLVEDLTQSNVQIGNTDDGIEKLKRYLLS

SHAFIVLDDVDRVQQLHALLSPVKNVLQQGSLIIVTSRNMDVLIKWGIVESSIFKLTGLQ

KQHSRVLFCRHAFDQPYPVREFEQVVEQFLAFCGGLPLSLKVLGALLRGEDLKYWKAQLH

RISQILP

>AB_027009_T.1

MGKRICCCICPCFSAPDEVEAVRTDPSGAQEASSANNRNSVVADSGKGGSSSETGGPSAH

HDHDSANSNNKRGGEGCSSSVSQEAPSAHAHVTDGHGGEDSANNSNSVVGDSAEGGSSSE

TGGPSAHHDHDSANSDNNRGGEGCSSSGSAHAHAHGGQDSSSSSNVNLDDLINHISQLLH

QIKEKSPPQKKQTAHYIWPTSSPGLLKLQEVLSRGIDFASAVKQVIPSQHSTEAKAVVLS

LLKGIGKAHVVTAGLLVVANILERFEAVKDNRAECLSLLDEMNRLAKHVKQLKERRGLRQ

GMENIIEEAVELIVEASLMCCTQIDSSTFSRFFTTSVNKGELVDFQQKLQKMYNHMKNQV

DICIFDAIDAIACRNGRPRKPRERGYPEYAVGIQEPTQEVIELLEWESEKNAWGVVLYGF

GGIGKTTVADAVFARLYIEECKYSMVSLFESITSTPNIVELQKCILRDLMVGTAAQEKEE

QVLESIVTFRDGQREIQGLLEKEMAFIYIDNVLEIEPLEKLLPRDMTKARKLRILMTARD

TEVRTACKFKTKVYDIKGLPDVEAVSLLKKEMYSDGGTDVTGEQMSHRQLSELAQICGGI

PRLLSLVAGFIRCEDDKQRAVRIIMEEKEKWKGRIGKYIEHYVFAYENLPPLLKDPFLDI

CKFFNGWQWDEVADILGESNLDMLQKRALVTRDTNMTTQVHDVILKVAEESDKGMRLTLT

SASQIQDILYQMEQKDLQGMKGLWLREIKIEDPLQISASKLDFMHRSLRILSLGDITVEG

KCDKKFEKLVFFQSGLGSIDLPFDVSKSEELTYLAYHQPEELNVYKMPEDLKKDAIDRMR

RSQALQISSRCLRPLRNLRILRLIGLTQLEKLPADLGDLNGLHELTLSRVPCIEELPPTI

SKLQSLRVLRLDRCSRFRKLPEE

>AB_027131_T.1

MAKGFLQGMGSAHLVTSGLLLLANVIEKFEEVDANKTKCVRLLKEMLFLGKLIKQYNARA

LLMEGPMHDLIKETTESIVERSLLCFSQLDSSKYSKFIKSSVNKDHLDNFREEIDRMYRQ

INAQMGIYILDGKKAPLSRRYPEHAVGIEESLKEVTDLLEWGSQKNVVAIMLHGFGGLGK

TTLADAVFSRLDIQGCKYSTVRLFENIDSTPKITKLQKLILGDLAEHGETIPDIRKPEDG

QRELGQMLEKVPTFIYIDNVLGQDELEQLLPRDINKAKKLRLLLTARDQDIVRRALNVKP

VVYSMKGLPHTEALRLLERKIYGETDDKEERINSTQLNKIVELYDGIPKLLEVVGAFIRA

AEDKQNAYGIWMEEKDKWDSEVGKIESYLFAYNLLPEVSKDPFMDICSFFKGWDWDAVAD

IVGHIELDMLRSRAFVSKGTNGAVIVHDVILSIGLQKTSGTRFRFTEAKQMKELLDDRDE

KEIQEIRGIWLSENKDLLSISASKLSLMYSSLRVLALGNIIKVQGKCDKTFEKLVFLQAA

IPYLPFDVSKSKGLRYLKYEPKDLNFPQIPSTLRHMEFDGRLHSRAAFEISFDDLQQLGS

LRILSLTNFKALKTLPEEVGDLVNGLEELTLSKCTSVRELPGSISKLSKLKVFRLDYCSS

LSQLPTDFGSLSSLQELNLKGCKNLKALPDNFEKLTALRLLDLSFCERLLDLPHGLGNLT

SLAELYLTNCSQLTSIPESIGQMKSLALTMDMSDCSSLKELPNGFCNLSFIKRLILDRCS

TLEKLPDRFGELESLTDLDLAECTSLKKLPESFGQLKNLTTLNINDCSNLEELCNDFHSL

ASLRILCLCDCPMLEGKEMDNVVKIKTLQHVVIEGSQMLLKRWGEIQREGKPGKPLVHTG

EVLPHDIKE

>AB_027255_T.1

MAGIPDAQEWEFAARRLPIGNSQAQQAVYQCLRLSYDALGYDVNLQMCFLYLAAYIEDEI

IHAHYQVIPLWIGEGLLARKKPEDQCGHDPVRMGKMYLNILADRCLVEPIARDVDGQVYS

FRMHDVLRDLAIQIAEHEENFFCRAGKGLTALEQNDCSGRIRMLLNNNKLSSLPQFSRPE

EICSLTVRENNIREIPERLIAGMSSLRILDLSTTSLQSLPESVGCLKQLVYLNLLFLPIT

TLPASITNLVNLQVLVLRGSRITELPSEINKWRSLKFLLLDFCQHLKCLPYSITGLTSLQ

YLYISNCPSIWTKHPEKQYRAASINDLGRLTQLKSLHLDNNGQTIREGTLGNLAEMEALK

LNLSGMAGLPRDLTSLLKMRRLSLNCPSVVKMESNFCEFQKLTRLKLWYCHMLEQLPLLH

TMKCLKRLDIVGCGKLEKLPKEFSEAGAFPSLEIFSLVYLDKLQELSTVEDGAMSALEVF

SVMGCKEVKMLPESYLNLKTLKKIRVYDCPMRLENLENANTRIKIVTGSATVDTKEFHKR

IIESKRVKDYIYDEYWCNEIPVFLFDLLYGQP

>AB_027280_T.1

MTGLDKGHSQVLFCLHAFGQRGPVVGFEELVGNFLDVCAGLPLSLKVLGAGLRGEKSKSW

KGHLDKITEAGLPTDIQRTLKSSYDSLDKEERDIFLDIACFFVGIKRDTAIRIWDGCGWR

GWLGFWNLEKKCLVQVDSNKCIRMHDHLRDLGRHLAEREPPECGRRLWRPTDELFPNVLT

SSTVRGISVVQQRSGESFQSPVEFANRIVDMSGLQLLRIPGDYLRSISMVTEQPDLLWLR

CESYTYSSLPSWPIPLRNLRVLEVEGREGEVEAASCLETLWETEIEAPLELRELNIRVPL

LRVPESIWQLKYLEKIELHNIIETLPEEFSHLRSLKHLKLCGRMVSLPNSLGNLTNLQHI

DLSGCHNLQMLPPSFRRLSRLKHLSLEYCLNLTLSDETFGNITALKRLNLTSCKKIERLP

SQVTRQQSLEELILNDTAVKELSNAIGDLRKLKILEIAGPPLKILPPSLGMLQSLEQLTL

KFCEDIKYLPSITNLKELRMLRMVDLTVECLPVGVTELDNLESLVVENCPIREVPFASVE

KVQGGIEELLDVRGRRLLDPIHNYMLG

>AB_027369_T.1

MGDAILTGAAANIISSVVSVVIPPLIQLIDDVLHLDRNTQLLEVQLNRMRSLLLDITNRF

QHDQRSLPDTVKNWSERLRHSLDDAKRVLRRIQQQKRCVDCSGLRRPRLSTQVREWNANF

DRLFIDLERDYSIFVNAQQIVLSAPLQSEALLQAVPESGYVGSGIKYGEMQLQAWLENED

LHFRIIGVYGMGGIGKTSLLKTVYNAVKKKGKLFEAVIWTVVSQNSISVMQTDIAKQINL

NLGSSSTVGMELSACLRGKKFLLILDDIWEHFNLEEDLGIPLGNDKGSRVVISTRSRDVV

RRMGADDYSIQIQPLSTDEACRLFCRGAFKAGTTPTKDIEDVATQIAGECNGLPLAINVV

AAAMKGNTSLHDWHLAFNQMRNVDPDFLASTGIDKGLYQPLRLSYDRLPHSNIRNCFLYC

AAYSEDYLISVDNLVEMWIGEGLVNSRETSGLMDVGRGYVKLLEERCLFD

>AB_027371_T.1

MPESSIYTMTGLDREHSRELFCLHAFDKRLPVVGFETSVTKFLDVCNGLPLSLKVLGALV

YQKDLKYWKAPLRKISQLVPTDVQSILQISYDGLDDQEKEVFLDIACFLINEDRDMVTSI

WDASGWVGWLNLQNLVNKSLVEIDSKNCIRMNDHLRDLGKDLAEKDPPSFHRRLWRPIDN

HFHNASEKSTVRGINMVEGRLGSSLDNPAELKNSLIDMSRLQLLSAEGDCLESISNLLKH

SDLLWLRWSCCPYTSLPSWIPIRNLRVLQVAGDNLENLWQSESEAPLELRELNIAAARLR

LPESIGQLKHLEKIELRCAIETLPEEFWHLSSLKHLRMPTNMRILPNNFGNLTNLQHLDL

SGCSNLRWLPDSFGNLTNLQHLDLSGCSKLDKLRDSFGNLTNLQHLDLSGCSYLAHLGDS

FGYLTNLQ

>AB_027644_T.1

MGRDANAASSALVTTKGPSSDNIKASSSAPSQEYTASYARRVQEVPSSRLEPLPQQRAYP

QHAVGIDDQVNRVIQLLEWENETKVAVAVILHGLGGMGKTTLADAVVARLDIQGWNCSTV

EPRAGLVPVRIFEDLQQEIQDMFGKEQAFIYIDNVTVRGEHLHKLLPNNSCNKKFRLLLT

ARDEMVADVLKECGIHVHVYPVKPLSLDAMELLCRNIRVPGTIPTPQIDEMLKICDGVPL

VLERVGAYICQSEDKDDAYRRIIEWGKDGRPFSFAEEYSIEKKGLLFDLDELPVSTKETF

LDICSFFNGWDWDKVSCILAEDDLNRLRKRAMLLKKEECENKVTVPHIILRIGLNMTKGK

RFTNAHDLSKALEGDEQGIRGINGIWLQDNRSEPFILSARKLDLMHNSLRVLALGDMTIV

VDGQCHEQEFKQLIYFQAGLIPYIPFHPTTPQELRFLDCRLPHNINNLQLTKISSKLNVF

NLNGGRCDCQFGTQNIFGRLRGLRTFKMTQFKTLNIFREDIRSLTQLEELDLSGCKNLVQ

LPEGLGYLE

>AB_027704_T.1

MGVLENSTITMGDLVEEKRWDLCAYHAFPYNNGNRPANIDEMTAKLVCAKCGGLPLAIKV

VGRSMAGITSI

>AB_027738_T.1

MAQLCTDTIPRYAVISRYKTSRRISRLISDITQHLQLVPLIGLVQTEGQTQELRERLARV

EELGTSSTSGTTSATTSSQPDALRGFFINEPLIVGQDAAFAELEKLMIDAEVHSPFSCIG

VLGKGGSGKTLLLKTVFNSAKVRDFFCDGFLLWLTVSESPSFTSLRNQLSSQIAMQSQVD

LDKDMNEEGIKIWLHESLQGKRFSLFLDDVWEEGAKLLEELGLLRLTDQSKCKVVVSSRI

QKALLEMGVNADTSTLIRMGDLIEDDSWRLFLYHAFPHMNGNLPENIEEGRAKVVCAKCG

GLPLAIKAVGRAMAGIIDAQEWDLAVQRLPNANNQDHQAVYDRLRLSYDALGNYNVNLQL

GFLYLAVNLPED

>AB_027836_T.1

MAYPTPAYSSEIEIEVENQPSPATPHDSSVASSAAAAYPTPASSSASSAAADPTSSSSGD

YGYDVFINHRGPDTKKTFASHLYRCLLSHGFRPFLDHPELQEGLNFPSQIEHAIRSASVH

VAVFSPKYADSDWCLRELLLM

>AB_027922_T.1

MANLTTHLYPSSSSTASPSASTSASQYHVFVNHRGPDVKTTFASHLYRALIRHGFRVFFD

REEMKEGDNISSQIEEAIRGASVHIAILSPTYAESSWCLEELVRMKNSGAIIIPVFFKVS

PSDVRYADKVNGKYAKGLQLLQQKKTYDSATTGEERCSSITIQNWKNALSYVANLSGFEH

RDNDDEGELLDKVVQCVLKMVPKTKTPLDVAKYPTGLDLKLKEFETTVFSQQQETERKEA

KVVGIVGVGGAGKTTLAKAFFNLTRSRYNSSSFLFDVRETARKMSLPYLQGMLLRDLKPQ

EKDIKIESRDEGVGILKRHLSSSHALIVIDDVDDMDQLEALLPIKKILRPDSLILITSRN

NDVLRTSGVLGSSIYILRGMNIPHSKELFCCYAFDRPDPLPEFADLVDHFVTACDGLPLS

LKVFGAQVSVQVERLFWEEILENLHKILPTNIHDRLKISYNSLEPEVKEMFLDIACLFLG

EDRDKAIRIWGNVGVRNLQDRCLLEVDSENKTKMHDHIRDMGRKIAEEGSMPRRLSHINM

IDDLFEQSSPVRGIKMVNFYGDFFRENSDISFQRLFDNSFFNPQIPPLEMKHLVSNFHFR

KLQLLATEEDHLDDMLRAQRPHLIWLRWYRCSHSSLPDWIQMENLKVLEMEGASLNLLWP

DQPQTAPLQLRELHVCAPLSHIPKSIGHLNYLERIVIRTNEYGTSLLDRLPDEFCNLCSL

KYLILEGFSRIVSLPDRFGCLTNLQYITLEGAHSLQMLPNSFGNLTSLKTLNVSNCEKLQ

SIQGLRQLTKLTSLFVHQCSEILELPGVEHLTSLEKLNVRRCKKLQSIPGLGQLTKLTFL

YVNECSEILELPGVEHLTSLEDLHVIHCQKLQSIPGLGQLTKLTHLAVKGCSEILELPGV

EHLTSLDTLSVSGLE

>AB_027923_T.1

MRESGAPIIPIFYKVKPAEIRWTDENKGVYAKELEQLQMKKTYDSETDQEKPRYDSVTIQ

KWKNALSSMANINGFDLVDGRFNGDEGQMLDELVERVVSIMAKKKRPYVAKYPTGLDEKV

KEFETTVLRQQHSGQVQVVGIVGLCGTGKTTLARELFNRKRSKYEKSYFLSDAEANAKKT

SVHSVLSDLLKGHNIDVGISKLKWDLKSSRALIILDDLDHLNQLEAVLTVKDVLSFDSLI

LITSRNRDVLISSGVDELSIYKLTGLNSQHSLELFCSYAFCQAYPQQGFEDLVDGFLEAC

DGLPLSLKVSGAHLFGRDDKSYWEEILNQVKVPADIQTKLKISYDSLSVEEQQIFLDIAC

FFIGEDRNSAVRVWEGSGWKGSLGFKSLQDKCLVEVDRENIIQMHDHLRDYGREIAKDLL

PRRLWHSTEKIDDLLHQHSSARTEVRGIKMLPDTDGDDETLSWYQRSQELFGNRLMKLHN

ALFHGVGVGVMKLQLLHTKGHILERILRKVHPPNLLWLSWSDCPYSYLPSWIPMENLKVL

KVEGKALKTLWRDESQAPLQLRELEIIDSFDL

>AB_027987_T.1

MATSGDANNNKKFDVFLNHRGPDVKKTIASCLYRWLTSRSPGLQVFLDQQELEGGRILET

QIKEAIASASLHVAIFSPRYAESRWCLEELYLMTESGKPIIPIFYQVTPRDLREARYNKG

AYAADLDKHERKIAWDGQPRYNSTTVLNWRNALNRAADFIGFDLATLNGDLGVLLLKVVE

CVCKRVEKLSSYEMRHRSGGILDTVKAIGMHDQLRALGRALGRMLLNYIER

>AB_028019_T.1

MGMAHLACTGLLVVALVLERFDTVSTNEEECLCVLDEICKLGKHLNDLKQRSRLRKDMEA

SIREATDLIMEASIMCSTQIGSSKGRRLLSASGDKEELVKFQQELQRMYRHMDSNMSICL

YDATECRKVRPQQPRERSYPDYAVGIEKPLEEVKKLLDWESEKNAVAVILHGFGGMGKTT

LADAVFARVYVEGCKYSMVRLFDDVTSTPSTSDIVNLQKYILEDLKMGTPEETTHEIRTV

EEGQREIGRILEKEKAFIYVDNVLQRYPFEQLLPRDMDKAKKLRLLITTRDTDVSRECRK

LKTKIYAMKGLPLTEAKSLLEKEMYTSYTDNMEKQ

>AB_028034_T.1

MGAAHLTCTGLLIAAHVLERIETVSANKDECLLVLDEICKLAQLVKKVRQRDGLRKGMED

SIKEAMGLIVEASIMCSNQIGFSKGWRFLTASGNKEKLENFQQSLQRMYRHMDNEIPICL

YDAIDCKQVRPQKPRERPYPDNAVGIEKPLEEVKELLEWESEKNAVAVILHGFGGMGKTT

LADAVFARVYVEGCKYSMVRLFDDVTSTPSTADIVKLQTYILKDLKMETPEETIHEIRTV

EEGQREIGRILQKEKAFIYVDNVLQRYPFEQLLPKDMDKAKKLRLLITTRDTDVSRECRK

LKTKIYAMKGLPLTEAKSLLEKEMYTSYTDNMEKQLSSKQLDEIVEMCGYIPKLLTVVAG

FICWAEDKQKAYIRVKEEREKNWKGEIGKDIAHYVFAYQTLDEGLKDPFLDICSFFKGRE

WSDVANIVGDDELNSLEKRALVTKKTQTVFVNDVIKVWVHDVILAMGHQQAEGTRFTSAD

QIGEFLDEKEDKVPNLLTVLFHTMLILVILSHTHNDMQDIRKIKGIMLRSGRLDLSLCIS

ASKLDLMCSSLRILSIKHYAKVEGRCEKIFEKLVYVEDKVSYLPFDVSRSKELKYLHVWD

QASSL

>AB_028114_T.1

MATAHLACTGLLVVALVLERFDTVSTNQEECLCVLDEICKLGKHVNDLKQRPRLREAMLP

SIREATDLIMEASIMCSTQIGSSKGRRFWTASKDKVELDKFQKELQRIYRHMDSKMGICL

YDAAECRQVRPQKPRERSYPDYAVGIEKPLEEVKKLLDWESEKNAVAVILHGFGGMGKTT

LADAVFARVYVEGCKYSMVRLFDDVTSTPSTSDIVNLQKYILEDLKMGTPEETTHEIRTV

EEGQREIGRILEKEKAFIYVDNVLQRYPFEQLLPRDMDKAKKLRLLITTRDTDVSRECRK

LKTKIYAMKGLPLTEAKSLLEKEMYTSYTDNMEKQLSSTQLDEIVEMCGYIPKLLTVVAG

FICCEEDKQKAYIRVKEEREKNWKGEIGKDIAHYVFAYQTLDEGLKDPFLDICSFFKGWE

WNDVANIVGDSELNSLEKRALVTKKMTLVTKKRTERGKIYRKKTIETVWVHDVILAMGHQ

QTEGTRFSSAKKIQEFLDE

>AB_028140_T.1

MVGIVGLGGVGKTTLAKEIFNRKLSEYNKSYFLSDVRENGKSSLHTLQSKLLKGLNPSFN

GPIDDIHQGTEMLRKSLSSSQVFVILDDIDHEDQVAALLPDRDGLTNDSLILITSRDRGV

LKKSGVEESSIYELKGLDSKHSQELFCSYAFCQLYPLRGFEDLVNMFLRTCDGLPLSLKV

FGALLFEEKEKSHWKDILDRVHEVPDDILDRLRISYDALSGEERQIFLDIACFFIGEDKN

TAITLWDGSRWKGSLGFRNLQGKCLVEVDSENVIHMHDHL

>AB_028169_T.1

MAHSNFPQRSSAASSGSASTSYAHDTHYVYDVFVNHRGPDCKETFATDLHKRLSGYGLRV

FLDEQELQRGEDLTSQIKGAIREASVHIAVFSQNYAGSKWCLEELELMLESKKIILPVFY

DGINPSDLRWTRGDRGVYARALAELENKKTVGTETHPEKPRYPFDTIEKWRQALSDAADI

SGFELAKYKGDRHQLVEVVVEEVLRKVKRPVHVSKHPIGLDQKVRDFETTVSLQPQSRET

RVIGIVGLGGVGKTTLAKEIFNRQQSKYDRSCRLSDVRDTDLKSSQSKLLADLARWNGQI

YDTDDGIGKIKDYLPRDPPTLALLIIDDVDDIKQLNALLEPVKDNVKSGSLILITSRSKD

VLISCKIVRSSIYELKGLNPQHSRELFSWHAFGQSHPFERFARLVDGFLNACNGLPLSLE

VIGALLCGKDDLREWEEQLSEVSKSPPEDIKSRLKISYDGLSDQEKKIFLDVACFFIGED

SNTAVRIWIGSGWSGWLGLRKLENRCLAEVDSESCIRMHDHLRDLGRDLAEKEPLHRLWR

QTDTLAQDLYGQSPVRGISMVHRYGSVWPEPQLFKTLSEDRDFQLIKNMSNLQLLRAEGD

CVRSIFSIVESRKAFSPKLVWLCWQSCPFRSIPRWITLNKLTVLEITGNKLETLWQDESQ

APVQLRE

>AB_028258_T.1

MANLPAAASCSTASSSASAPSSGYDVFINHRGPDTKKKIASHLYFGLLRCGLRVFLDKQE

MEKGDEIQSQIVQAIRDASVHIAIFSRSYAESHWCLAELVLMKNSGATIIPIFYEVDPSV

VRWTENGAYAEALRGLEQKTKKNPETGEVKPRYGSDTIQNWRDALSCVADLSGFTLLGDQ

DDEGEVLDKVVENVLKKIPKTPLDVAKYPTGLDLKLQEFEKIVLSQQQQEEERVEAKVVG

IVGVGGVGKTTLAKEFFNSHRSRYNASSFLSDVREVTGRISLEYLQSQLLQDLKHMETKI

GRHEGIGQFKKHLSLCHALIILDDVDHATQLEAFLPLKNIMNPNSLILVTSRNKHVLKSS

GILESSIYNLEGLDMTQSKKLFCSYAFNQPLSLPELADLVDQFVTACKGLPLSLKVIGAL

LCGENDKSYWQEQLDRLNHALPEEIFETLKISYDSLDKQDKPIFLDIACFFIGEDIEKGK

GIWGKAGLTNLRNKCLLEVDTVNKIKMHDHIRDMGRMIAEEPTMPRRLWNSTTISNILVE

QQSALITEVRGIKMVSSYHDVFQKKDWHTWSYSGMRSGISPSKYTFKNSHCPGMKNLELV

AAEGDHLEDMLRRVQRPNLRWLRWYRSSHPFLPDWIPMENIRVLEVEGMRLNIPWPHQSQ

GVPLDLRELNILAPLSDIPKSIEQLKYLERIAIYNPELLGALKRLPNEFCRLGSLKYLVL

RDFSRMVSLPESFGELTNLELIDLGGASSLQTLPGSFGKLTRLKYLSLRGCKNLTISSGT

LGNITTLEYLNLCSCEHVNKLPPQVAHQLSLQQLYLSGTKLKELPSVGCNLKVLELGGDL

LEVLPPGLGDLKMLETLSLFNSRQLKSLPDSIGELSRLTKLVIYSCGIEYLPRSLLKMKI

LKVLDVRNCPLLEYPFKKEVEEERETVLKKLNSSNYRCMSGLKSLHLSGTKIREVCFDKG

VCPNLQQLTLNNSELRQIGGLCALPKLRDLKLASCAKLEELYGFETLTSLETLYIWRCEK

LQSLPGLGQLTKLRTLQVDSCREIQGLPGVEHLTSLETLNVSRCEKLQSIPGLGQLTKLR

TLQVSSCREIQGLPGVEHLTSLETLNVSQCEKLQSIP

>AB_028310_T.1

MTKKCCNCCCFTSFSCFPCFSARNTTDSSPLIAIETGGLERHDDDTRSVPSIPPASTSTS

HVASSSGEVASHPNASADALSSSSPNVANNNTNSVSTSQVASSSAEGTSAPNASSSAEGT

SAPNASSSSSANVANDNTNAAFPAVATPTPASIHPHASSSFSTAVAQTPSTSTGGNSSSS

VQTMSDAINNILQVAAATNVDLDGLMKHISELLEQVKGEGKKSSLPKAVQNYLPTSKSWI

KLKEILSEATNFAKDIIPGDAKNVVRGLLQNIGTAHLATTGLLVVANILERFEDVSNNRE

ECLHLMKEMSFLAKVVKQFEKSSQLKEEMQSVMEEAIGLIVESCIMCCSQMASSKYFKFS

SASVNKEKLGEYRKKLDVMHKHIYIEMGICIYDKKAPLSRRYPEHAVGLQDSVNEVIDML

EWGSEQNALTVILHGFGGTGKTTLADAVFSLVQVEECQYSHVQLFKDIKSSPDILELQKL

ILKDLMGSENIPDIRKHQDGQRELGKMLQKKR

>AB_028336_T.1

MDASTSTSAHANSNHHFDVFVNHRGPDVKKTFASHLYRRLLAYNLRVFLDYEELQAGENL

TPQIKRAIESASVHVAIFSCGYAESKWCLDELIFMLESGVPIIPVFYGVEPADLRWTNAQ

SQGVYAKALCDLENKRTDDGQLRYSSTTIGMWKGALSRVAETGGLELKTFKGDEVALLDN

VIECVLKNVKKRPLYLATHPTGLDEKAQDFEDKVLLRQHSGRVQVVGITGMGGVGKTMLA

KELFNRKSSNYSKSYFLSDVRENAIKSSLPSLQTKLLIGLTHLNLHIDSIDQGIEMLRKY

LEKMQVLIVLDDVDNVDQVDSLLPMEDVLQSESLILITSRDKNVLARSGVQESSIYQLTG

LNERHSTELFCSYAFGQPHPPSEFESLVHQFLNACNGLPLALKVFGGLVYGKDKQYWEKQ

FEKTEKTLHKDIRERLKISYDALDEEEKQVFLDIACFFIGEKKDMAIRIWDGSRWSGLLG

FQTLQEKCLVEVDIENKIRMHDHLRDLGRDLARHPSLPRRLWRGKKDIDDFRQHLSVPGI

ANVRGIRWRN

>AB_028504_T.1

MEKKRCRCRLFSCFSCFSAENTTPSAAQPDHGSEIAPVYHSSAVPSTAAPNATDHGPQLI

VPPDVSSSSTDVLPESNEIAPVDHFSVTSTAAPNATDDALKHPQHGPQPILPPDASSSST

DVLPDSNEPQPIVHPDLSSSSPLAFPAHDEVYQVSEAASSSNAATDNHKVADDQLQGTPS

SSTVSNAPHLINSILTAANALDTENVNADLDGLIEYISNLLETIKEKKMTGKKSPKTESG

KGYWQIPSDGSLRKIREILSDGINVFKDIFPGEAKTAIQGLFKGMGMAHLACTGLLVVAL

VLERVDTISTNKEECICVLDEICKLTHHVNDMKQRPGLRGAMDASIKEAINLIVEASIMC

STQIGSSKGRRFFSASGDKEELVKFQQKLQRMYRHMDNKMGICLYDATECRQVRPQKPRE

RPYPKYAVGIEKPVEELMELLEWESETNAVAVIVHGFGGIGKTTVADAVFARLHVEGCKY

SMVRLFDDVTLKPSPSDIVKLQTYILKDLKMGTPEETSSEEIRTPEEGQREIERILEKEK

AFIYVDNALERYSFEQLLPKDISKAKKLRLLITTRDRDVGRECSKLKPKIYPMKVLPHIE

AKILLEKELYGETDNMEKPLSSSQLDQMVEMCGCIPKLLIVVAGFIRCEDDQQEAYGRVK

EERERNWKGEIGKDIAHYVFAYQNLDETLKDPFLDICSFFNGVEWSEVADRLGDGELNSL

EKRALVTKKTQWSATVTVWVHDVILAMGLQQAEGTRFSSANKLQEFLDDKEDKEIRQIKG

IMLPFKDRSMRFKDPLYMSASKLDLMCTSLRLFDLRDSAKVKGKCEKIFEKLFYFKGQIF

DLPFDVSRSKELKYLDFSYSPDQEFSNYEELLGSISKLPYLRVLKLSCCKNLGQLPKYFG

SLNSLQKLHLDHCESLEALPSSLGNLSSLKLLDLWGCRSLKDLPCDLGKLTSLAGLRLRN

CSKLSSIPDSIGQLKSLDSEMDLSCTGLKELPDGFCNLSFITHLSMDSCERLKKLPETFG

RLKSLEDLDLSKCTTLEELCRNFNGLSSLRRLSLNSCKMLKKLPEDFHCLASLEVLSLRD

CHMLEGKWMESMVEMKTLQFVDITGSPMLEERWEEIEREGDQSWSFAVQVIIISFIYYTS

LFQCLDYQTYNIENISIFL

>AB_028604_T.1

MDVLQPDSLILITSRDKDVLLKSGVQETAIYQLTGLDEYHSRELFCSCAFRKPSPPPSEF

EFLIHQFLKACNGLPLSLKVLGALVHGKDHRFWKIQLRKLRKTLHQDIKRSLQISYDALD

EDQKQIFLDIACFFVGEEKDMAIKIWDESGWVEFQALQEKCLVEVDFENRISMHDHLRDL

GRHLARDPTLPCRLWRGKEDIDDL

>AB_028642_T.1

MHPRVNTGICDENHALYAPLCGGVFLNHRGPDVKATFATDLYRCLVACGFNVFLDEPEMQ

PGHDITCQIKQAIENSSIHIAIFSPTYGESSWCLNELEHMWEMHESDRAIILPIFYDVQP

SQLRHTGKGQTGMYAKALWNHEEKGTNFQTIQKWRKALRGAAGISGFELKKYNSDEELLG

EVVGHVLDIREKKRLKYVTKDEFERTIHNCKEFGRIVDSCKAKVMGIAGPRGVGKTTLAK

TFFDSKRSEY

>AB_028661_T.1

MDKAKRLRLLITTRDKDVSRECRKLETKIYAMKGLPLTEAKSLLEKEMYTSYTDNMEKQL

SSTQLDEIVEMCGYIPKLLTVVAGFICCEEDKQKAYIRVKEEREKNWKGEIGKDIAHYVF

AYQTLDEGLKDPFLDICSFFNGEEWSTVADIVGDSELNSLEKRALVTKKRTEGRDLYTRR

TIEIETVWVHDVILAMGHQQTEGTRFTSAKKIQEFLDEKKDKDIQKIKGIMLSKVDETLS

ISASKLDLMCSSLRVFQLGKWSKVDGKCTKILEKLIYVAVDIPNFPIDVSMSKELKYLSW

EGDLNEELLRSISKLRPISKLQYLRVLKLHSCNNLPKEMGPLNSLQKLHLDMCTSLQALP

SSLENLSSLQELRLSNCDRLKALPSSLGKLSLLRSLDLWNCESLKHLPKDIGSLNSLRTL

HLYQCKSLEEIPSSIENLSSLQELRLSSCDRLKALPSSLGKLSSLRILDVSQCGSLKDLP

CDLGKLTSLTELSLYHCKELISIPSSLWDLSLLRILNLSWCQSLKNLPCDLGKLTSLTEL

SLSFCKELISIPSSLWELSSLRILDLSYCRSLKELPCDLGKLTS

>AB_028673_T.1

MDNKMDICLYDATECRQVRPQKPRERPYPDYAVGIKKPVEELKELLEWESEENAVAVIVY

GFGGIGKTTVADAVFALPHVEGCKYSMVRLFDDVTSKPDIIKLQTYILKDLKMGTPEETT

PEEIRTPEEGQLEIGRILEKEKAFIYVDNALERYPFEQLLPKDMGKAKKLRLLITTRDRD

VARECRKLKTKFYPMKVLSHTESKTLLETELYGEVDNMEKRLSSIQLDHIVEMCGSIPKL

LIVVAGFIRCQDDPQDAYRRVKEEREKNWKGEIGKDIDHYVFAYQNLDETLKDPFLDICS

FFNGWKWSEVADRLGDGELNSLEKRALVTGKTEGTWRTVWVHDVILAMGLQQAEGTRFNS

ANKLQEFLDEKEDKEIRKIKGIMLLFMRFKDPLYNISASKLNLMCTSLRHFELGDSAKVK

GKCEKTFEKLLYFRGQIFDLPFNVSRSNELKYLDFSYSNKEFSNDTNIEELLGRSISKLG

YLRVLKLSGCYKLRQLPKDFGSLNSLQKLHLDRCFNLEALPSSLGKLSSLRLLDLSWCRS

LKDLPCELGKLTSLAQLRLIYCSELSSIPDSIGQLTSLAQLHLDGCSELSSIPDSIGQLK

SLDSSISLSHTGLKELPDGFCNLSFITHLQMESCVRLEKLPDRLGEVTCLRYLNLYNCKR

LEKLPETFGNLSFITRLHMHRCERLEKLPETFGQLKYLEDLDLSECTILKELCRNF

>AB_028683_T.1

MLKQLKGSENIPDIGGYEDGQRELGAMLKEVPALIYIDNVLEVEALRNLLPPDMNKAKKV

RLLITARDSNVRKACPLKRVPELYHMRGMASTDAVSLLQKEMYDDPRGILDSEQINIIIK

KCDGIPLLLKLIAASLRVAKDKPQEVRGVMEQLENLKGEDFGPDKIEPYFFAYDKLPKDC

KDPFLDICSYFHGWDWDTVANIVGESELSMLESRALVTKDTNEVVSVHDIILELGRHKSK

GVRFRFTHANEAELLFDKKKEDIQAIKGVWLSEIENPLDIFAWQLDLMYGSLRVLALGNS

TTVKGVCTNKFEELVFLKARITDLPFQVSKSKRLKYLNYKSKDLKLSQMPSSLRHAEFDG

RLHAHAFQIYSSDLRQLKELRILKLINFEELTNLPENMGDVMSGLRELSLSGCTSLKELP

LSISKCGSLRILRMDGCHSLTELPADFGSLNSLQELHLPYCRNLKALPDSLEKLSSLKLL

ELSGCHELLNLPSGLGKLTGLVKLDLYSCSSLESIPESIGEFKRLQPTLELSFCRSLKEL

PNGFCNLSHITHLSLVYADLENLPDGFGTLTSLKVLDLRLNYRLEKLPEGFGQLKYLEEL

KLGSCFWLTELCNDFHCLSSLQNLDLSGCGRLQKLSPDFHRLASLQHLDLLSCETLEGKW

MDDVVKLNRLQFVCIEGGEQLVKRWAEIQTEGHQSGSSFVVNSGQDMSMSEDQKENIEKT

LLSDFERLLTNGRGESIRLPPNTLFLVICDLSQFFFSDEFPWPLIEETIDEIEKEAPTII

YIGPHFKKLSKKVQERILAWKMTGSGAVQVVTKMFLTLDISTAHLCEGVINYYFQMHTKM

VGDGKSDKYLPPWKHLTKTETEELLLGNCRRYLQLKEFVKTKQESNLELLRELFAHGEEE

STSIFQKYYPSQQDGNEDSEPFTCGVDKLKGKNILLVMRPTDSCYDIRLQSLKELYVKEK

EILNLEIISIPIVGQGYYTSEAYKKFLKEFSREVPWLVLRTPAIARSVKFFLVKECGWVG

GDEFEKCYTNRICLIESNGRIKTYNPSLFSMLDKWEFFFNHLESVSYQVKW

>AB_028916_T.1

MGHRMEVKLENIEHKVEDKLEEIGHKIDAVREESDLKFIADQMDQLEIGKKASQSSVPEL

PDFRVGLKGLVYELKKRLLRNDAPLLGVIGMGGSGKTTVAIALCNNNEVKGHFKERIVFE

RVSQSPNLKGLLERMWDKIVREKRPEFHDVEDAHQQLQQRLRQMEPQPTLVVLDDVWEID

HLEKLLFEGKGYKTLVTTRNNNVVKSDYRYDLPCLREQDAVSLFCFSAFDRKSIPDTADG

NLVKEVIAECKGLPLALKVIGRSLHDQPPAAWITAKDKLSRGQTISEYHKTEVLKRMATS

IDILEEDVRECFLDMGIFPEARKMSADPLLDLGVYVHKLKWTAAYTILIELASRNLVTLV

NNQKGTLGNPYGCSGELSLIQHDVLRDLSIYWTEQEPIIEKRKRFIMPQKVDSIRKMWKK

HADHDFNAEIVSFHTGSMEEKDWCQMKFPNAKALILNFSASEYFLPPFMQSMDKLKVLII

VNHNSKRATLKGISVLSSLTRLKVIRLERVIVPPLQEYCQSWQKLEKLSLVLCEGCGNTT

GLHMGAYLNFPRLLELAIDHCSDLRELPTSICSLTTLKRLTVTNCHDLQKLSEDIGNLSS

LQMLKLYACPSLKELPYSVCKLRQLQFLDISLCGHLKQLPDQLGQLSSLKELDMRECSRV

KQLPKSASDLRSLVHVICDEKIRHQWSFIKVSTIPSLRVEVAEEHFHLDWLED

>AB_029068_T.1

MADLLASSSGSNISPYDVFLNHRGPDTKSSIATDLYDCLARYRLRVFLDKPEMEKGDFVS

SQIAAAIRGASVHIAIFSPGYADSVWCLDELLLMVESIKWTPGENGGVYAEALRRLQEKT

TTDSKTREEKPRYESDRIEKWRNALSQASDIMGFEYDGVE

>AB_029143_T.1

MLERLLSSTNALVIIDDVDHVDQLDALLPHQTGLHSDSLVLITSRNKDVLTSRVVDTSIY

KLTGLNTQHSLELFCSHAFQQSYPLPGFESLVEKYLETCNGLPLSLKVFGALLYGKDISY

FEEQLESLEQILPTTEIHQ

>AB_029244_T.1

MDATDIVTGLAKDTASTIIAAVTKQIKDVVELEENLELLNTDFTRMEVFLRYIDRQFQEQ

QRRLPDPVELCLIRMNHALTQARRLVESAKRQRRRYFGCCLLCNPKLTTEVRNWETRFDR

LFQELQNDFSITANTQHIASTSAPQAEVLLQDVPDDIVGSAIYSAQMKLQTWLTEPAHCV

IGIHGMGGVGKTSVLKVIHNHYKEEVSGVFDVVIWSTISQYKNEGEDDKIKELQISIAQT

LSLDLKDSHTPDMRKMKLSAYLEKKRFFIILDDMWRFIDLNKIGVKFGHDKGSKVLISSR

SSDVIRSMGANDYSFKMQPLSEKDGWELFRR

>AB_029245_T.1

MTNVDPSFPETHTTIDAELYQRLRWSYNDLSDDLKSCFLYCAAFPEDTEIDAETLLEMWS

AEGLVKRRGITNLIDVGHHYIDFLASRCLIEYVGFKYMLIPRSGGGNVIVNREVIKVHDV

LRDMAIYIGQRDGQWVFASAQHLHNFPVEEVTRNCKRLSLGHNDIQHLPTDLECTGLVSL

VLAHNTKLTEVPAPSLLNMMSLKVLDLSCCTLITSLPTSVGQLGELEFLNLQGCYRLKEL

PESICTLSRLQFLNLYFCESLISL

>AB_029563_T.1

MNPPAVMQNHTPSIARSDVPSSSTPSSSVLTQSDYTERITGADDPVDPGNVNADLDDLSN

YILQYLAFIKHEGEKPSPVSAAECDWPGSKSAIDLKELMNGVAETAKDLCPDAKTIAQKF

LQGMGGLHMLATGFLVLGNIMERYETISSNKEECVCLLERMNNLIQDVKQLQEAKIKEAI

ELIIEASLACCTQIDMPKYRSFFSTTVNKKELQRFQTRLTKLHREIQLQKEFVLHDLLLR

VPPCPRASEEFRRENKVQIEKVTELLQQKSEKRAVAVILYGLGGTGKTALADAVYWSLYH

EKVLPYRNHEEVLECKYSFVRLFDSITPVPDIPKLQIQILEDLTSRTADIRRYEMGRDAI

KEVLEKEAVLIYIDNVLFPGKVEQGLDAIEKLLPRTTTKVKQLRLLITTRDKGAAVDACH

QVGIETELYPIETLEDAKAKQIVEEELNRGEDDHVNEQQNGTDKKLDSNQINQIVQICGG

IPRVLKEVAAYIGVSENRVEAQERYHQVIWERENNWNGRVRWNKIAHYAFAHEHLDEHLK

DPFFDICSYFQGWDWEELSNIVGDFELKSLEDRALVTKDVKSMTAKVNNVILAIGIHNKG

RRFKFTSASEFQRVLNPNNDEDLRRIKGIWSNDNIGPLDIPASKLDLMCGSLRVLALQNS

TKVSGRCNRTFEELYFFSAQISDNPFDPSKVKKLRHSRYRPDDFHNLCQGSSNEESVERN

NDMQE

>AB_029633_T.1

MELSACLRGKKFLLILDDIWKDFDLEEDLGIPLGNDKGSRVVISTRSRDVVRRMGADDYS

IQIQPLSTDEACQLFCRGAFKAGTTPTKDIEDVAKQIAGECNGLPLAINVVAAAMKGNTS

LHDWHLAFNQMRNVDPDFLASTGIDKGLYQPLRLSYDRLPHSNIRNCFLYCAAYSEDYLI

SVDNLVEMWIGEGLVNSRETSGLMDVGRRYVKLLEERCLFDTLYYNDDGRWYIKVHDVVH

DMATYIAEKEEKCLFRTRQKLQKFPSEKGIENCKRIAIGENNISVLPTDFRCPNLLTLIL

RLNESLREVPNGFLVHLTSLRVLDLDRTKIEALPISLCHLRRLEFLQLSNTLIKDLPEDI

CNLSQLRFLNLRGCRQLQSLPCKIGELQNLKTLDIGKCCSLTGIPREISQLTSLNRLFLA

TSEGGEKSIMDVEEVKSGVCSLKDLANCPNFMELQVHVKPGIEVEGIRSGIQVGIMGTWV

EMRKLTLVFDVEEHDVVEDLPQDMQNMKKLQIFMLYKYQGRGLPNCISEFPQLEKLDLYS

CFQIRELPPLEKLPNLRSLSLDNCIKMKELGITGGFPTLEWLKLRKLPKLESMGSSSSSD

VVWNEETMPKLRVLSITNCDSLKGLPMGIEKLPNLKEIEVQEDWWKESIWVETDAGIQTE

NDA

>AB_029670_T.1

MRGISASRIGHSLPSISMAVNYPYYHVFINHRGPDTKDAFAVPLYRSLTSRGLRVFLDKP

ELDAGNKIISQIEAAIEVASVSIAIFSPGYAESSWCLNELLSMLKSGNVIIPVFYNIRPW

ELRVTEGTYAEALRKHEDKKRHNIQTIEQWRKALFDVANISGLVLEAFNRDIFELLDKAV

QCVLRHIPKTPLAVYD

>AB_029750_T.1

MSLNNLQRKLIKDLKHIEIEFQSHEEGIGILRQYLSGCHALIVLDDVDDIDQIDAFMPIK

DVLHPNSLILVTSRDKNAIRIPESAIYTLEGLTKEQSKELFSSHAFPESTHESPEFENLV

NEILSVSDGLPLSLRVLGALLSGKSKSYWEEQLEEFHRLLPRKIEDRLKISYNSLNRGEK

EIFLDIACFFKGGDRDRAIRIWGLVGIQNLENKCVL

>AB_029766_T.1

MHLSACPLPGLFFLLIRMIFLFLFVLFPPIPLSLKRLFIVVTSCSGCQERFTLPFLMAPG

REWIASSLRLVEGGNITSEIKTTIQVTSVHVAIFYVNYMQSNWCLDDLDLMAKSRAPIIP

VFYKAKPHPLPGFVDLVYKGM

>AB_029868_T.1

MKNVPKRSRVVQSSEVQSVVNCVLKGNDPKSCNVVQSEVEHCFGLKVKLKEFEKAVLLER

QEEHAVAKVVGVVGAGGVGKTTLAKEFLKCKKLNHNESFFLFDVREHACRLPLEDLHEKL

IEELGIHTLNISMYYGFAYKHILSCRALVILDDADDVHQLNALLPIKHLLHPKSLVLVTS

RDKDVLERSGIVDSSIYSLTGLNKLHSPLLFCSYAFLAGYPSPGFADLIDEFVTACDGLP

LLLKVLGALLCGEDRSFWQMQLNKLHKCIEPAKIYRTLKISYDSLNQEEQQIFLDISCFF

IGKDKDTAIRILGMTALEKLEKRCLLEVDSKNKIQMHDNLRDLGRNIANELMPGRLWHQT

TKNIYDLLEQSSSVPFQFRGITVIRSAYSDEPEACLRSKMPYDKRTGVIFPNQIRGPTNL

KFVAAEGGYLEDILRVGSPDLIWLQWYECPCSNLPSWIPMENLRVLEVAGSKLTELWGDG

SE

>AB_029905_T.1

MVKKPDLPVAKYPTGLDMKLKHFEDTVLLHQHQRGKPQIFGIVGLGGIGKTTLATKFFNR

EKSNYHKSCFLSDVRENAVKGSLTSLQRKLLKSLTSIDVPIESVDEGKGMLIKHLDEDKM

MHYKKHPKSSNAPENVGHVDEDKSSNGLIILDDVDNVDQVDALLPFDRACSGWLILITSR

DKRVLEGSKVEISSIYILNGLDDHHSRELFCSHAFNQTHPLPEFASLTDGFLKACDGLPL

ALKVFGANLYGRDVSYWEEELESFPSEITDRLKISFNALNSEEKQMFLDIACFFRGLKRD

TAISIWNESRWKGRRGFQNLQDKCLVDVDSWNVIHMHDQLRDMGRDVAEKWRPHRIWRWR

DNVDQQPSVKTARGIKMDLSDYTAYSAVLRDIGTGSLRILDTQDFVLERILRGVELPDIL

WLRWVECPYSSLPSWIPMKNLRFLEVSGSVLNTLWQRESESQAPFQLPVFLEVLNTNNCV

RLKSITELEQLTKLRQLVVVGCSELEELPSMKTLVSLEELHAEGCVKLKSIRGLGQCTRL

RKIDVDGCSELEELPSMQTLVSLEELWAKGCMKLKSIRGLGQCTRLRKIVVSGCSELEEL

PSMQTLVSLEELWAKGCMKLKSIRGLGQCTRLRMLDVEGWSELEELPSMQTLVSLEELWA

KGCMKLKSIRGLGQ

>AB_029921_T.1

MDVREKVARNDLNSLQVQLIKELAQRNVQINTPDEGIEMLKKHLSSLDGVLIVLDDVDHI

DQLDKLFSPVKDTIHSSSLILVTSRNKDVLTSAQIVESSIYMLKGLNQQYSLELFCSHAF

NQPYPVIGFQQLVEKFLGACHGLPLSLKVIGALLCGKDLTYWEEQFHKISRVVPTDIQSR

LKISYDGLDEEEKQIFLDIACFFIGEKRDTALRIWDGSSWLGRPGLRHLENRCLVELDNE

NCIKMHDQLRDLGRELAKNSEYPLRLWTLRDSFDDLYNAFQIQRPNSQFIPHLSFVRGIH

TFHTNDAKISFEDLAASCNMSRLQLLWAEGDFVKRLFQVPHSPHSPQLIYLHWDYCPYSS

LPTSIPLKNLRVLYIQGGFLKTLWRHESQAPLQLRELVLNAEIRRIPKSIGKLEHLEKFV

LTSECIKTLPDSLGNLRNLQELNLSGCFGLQMLPDSVGNMTNLRELNLTSCSGLQMLPDS

VGNMTNLRELNLTSCSGLQKLPDSVGNMTNLRELNL

>AB_030163_T.1

MLFRTIAFNGDTVSVSMDIMECARKIAEECKGLPLAITVIAAAMIGKTSVDQWEVSLDLM

KTADPSFPDTHPRVDPELYQRLRWSYDVLPNSNVKNCFLYCAMFPEDYKIPVRNLLQIWI

AEGLVRSKQGSHFMDMAIGRSYVNLLVDRCLFQNVSRDEKISDGWDVLNGQRIRVHDCLR

DMAIYIGEKDENCVFKAGQQLQNFPEIQNEDCKRISVSSNQIQNLPTLFTRPKLVSIFLN

DIPIKEVASGFLVSLTSLKVLDMSNSRISSIPSSVGELKQLEFLNLSGLGDIQDLPAEIC

HLSSLQFLDLSSCTGLLSLPSEIGELKTLKYLNLSRCRSLRVIPHEISQLTSLSILDAKE

AELSVEAAELEASIWSLKSLRNLTMLRTGVKVDVQEGMTSSGMKVGIMGNWLEMRYLCLY

FTKGDYVMEDLPRDMQNMNKLQSFTMCFYNGLSLPDYIGNFQHLEFLRLSICPHLSQLSP

LEHLPNLKYLYLYKWPHLKELGIGNSGNARGFLMLERLVLENLERLESIAEPSNNGVWNE

SVLPKLHTLTIRGCPSLKRLPRGMEKLSSLNTLIGEEDWWQSIEWEDDNMKCHLQNLFRT

L

>AB_030182_T.1

MGMAHLACTGLLVVALVLERFDTVSTNEEECLCVLDEICKLGKHLNDLKQRPRLREAMVA

SIREATDLIMEASIMCSTQIGSSKGRRLLSASKDKVELVKFQQELQRIYRHMDSKMGICL

YDAAECRQVRPQKPRERPYPDYAVGIEKPLEEVKKLLDWESDKNAVAVILHGFGGMGKTT

LADAVFARLCIKECKYSMVRLFDDVTSEPSTSDILKLQTYILEDLNRGTHEETPKIRTVE

EGQREIGRILEKERAFIYVDNVLQPYPFEQLLPRDMDKAKRLRLLITTRNTTVSRECSKL

KTEIYTMKGLPPMEAKSLLEKEMYSDADNMEKITVSQLDQILEMSGYIPKLLTVIARFIR

CAEDKQKAYSRLKEERETNWKGEIGDDISHYVFAYQTLDERVKDPFLDICSFFNGQEWST

VADIVGDSELNSLEKRALVTKKRTEGLSLETVWVHDVILAMGHQQTEGTRFSSAKKIQEF

LDEKEDKDIQKIKGIMLSKVDETLSISASKLDLMCSSLRVFQLGKWSKVEGKCTKIFEKL

IYVAVDIPNFPIDVSMSKELKYLSWEGDLNQELLRSILKLRPISKLQYLRVLKLHSCNNL

PKEMGPLNSLQKLHLDMCTSLQA

>AB_030350_T.1

MGDPGFISGLIGVVVDRLIQLIEKEVMIAVCCREELTLLGNLVKEIQLVNMEIQQYRIAI

RQSQSDVVKNIEPVTVNNWLTTLNSILEYAREIVLKCQVPAYNLVSRYMLSKKIRKCIDR

LQGHLERLPLIGFTYQLHSHLTNRLQNQEAARNQSSIPSTSHPSLESYRQQPIQEPCIVG

QHEVFNNLKSLLMDDKEIAASTPSRIGVFGMGGAGKTLLLKRALNDKQVQQHFEKDLILW

LTVTHNLSILNLRNDLGRKINLHISEGFDDRWGEEDVRRWIHEKMSSRRFLLFLDDIWDD

SETLLEMLGVPDDADHVNGSSKIVVSTRDRRVLSKMRVSAQISMDYLSPDESWRLFCFHA

FADNQHTPPVGIEKIAEDVCTECDRLPLALKVIGAAMAGITAPSEWREALRKLRNADKLD

PQVEKKLYNRLRLSYDALCTPQLFALQLCFLYIGAFKED

>AB_030496_T.1

MGTAHLAFTGLLVVALVLERFDTVSTNEEECLCVLDEICNLGKHVNNLKQRPRLREAMLP

SIREATDLIMEASIMCSTQIGSSKDRRLFSASEDKVELVKFQQELQRIYRHMNSKMSICL

YDATECRKVRPQQPRERSYPDYAVGIEKPLEEVKKLLDWESEKNAVAVILHGFGGMGKTT

LADAVFARVYVEGCKYSMVRLFDDVTSMPSTSDIVKLQKYILEDLKMGTSEETTHEIRTV

EDGQREIGRILQKEKAFIYVDNVLQPYPFEQLLPRDMDKAKKLRLLITTRDTHVSRECRR

LKTKIYVMKGLPLTEAKSLLEKEMYTSYTDNMEKQLSSTQLDEIVEMCGYIPYLLTVVAG

FICCEEDKQKAYNRVKEERERNWKGEIGNDIAHYVFAYQTLDEGLKDPFLDICSFYKGWE

WSKVANIVGDGELNSLEKRALVTKEIETVWVQNVILAIGHQQAEGTRFTSADKIGEFLDE

KEDKDIRNIKGIMLDKGCISASKLDLMCSSLRVFSIQEYAKVEGTCEKIFEKLVYVEAKV

SDLPFDVSMSKELKYLDVLDDSNQGLTYSKCKYIEKLLGSISKLPYLIVLKLSHCKNLRQ

LPKDIGSLKFLQYLYLDNCESLEALPSSLTDLSSLRHLYLFRCQSLKDLPCDLGKLTALA

NFRLEGCKELSSIPSSLGDLLSLRVLDLSGCRRLKDLPCDLGKLTSLTELFLIGGKELIS

IPSSLGDLSSLRLLDLSGCQSLKNLPDLGKLTSLIELRLEGCKEPSSIPEGNYFKI

>AB_030633_T.1

MEIVTGVAGQAISNIVGRVIGVMKDVIEKEDNCQILHTDLKRMENLLLDISTLFKDQQKE

APETVKNWLQRLENSLTEAKSLLHRSTPSLRCLDCLICNPVRLSKEIKAWKVTFDNLFQG

LQNDFSTFVSAQQIAKSAQQKALLQDVPPSEFVGSGINSDKAQVEMWLTKTDNVHIIGVW

GMAGVGKTSLLKVLYNHYIEVSNVFQHVIWVTVSADYKVLDLQARIAKA

>AB_030695_T.1

MACSSSSSPPDTIGTSSSTPSTSTSTTEHHHIYDVFINHRGPDVKKTFASHLYHRLLSSG

LRVFLDQEELQQGENLTSQIQGAIETASVHVAIFSPTYAESKWCLDELVLMLNSGTPIIP

VFYGVKPAELRWTQSKDRVYGNLLRTLVWWPLGIHGMFAKIRRILGWSTRRKDGTYEHAL

LNLGKKKTIDPQTNKEKPRYEANAIENWRNALSRVAEISGFELEACNSDEGRLLNKVVEQ

VQRKVRKTPLNVATYPTGLKEKLEDLDKMISLQQQSGGAQVVGIVGLGGIGKTTLAKEFF

NIRRTSYSASCFLFDVREAAGKGSLPSLQTELVRCLKQLDLSQVRSIDEGIGLLKAYLSF

SRALIILDDVDHPSQLEALFLPVKDILFPGSLIVVTSRDKDLLTVSGIVESSIYKITGLN

RRHSLELFCSHAFNQPYPSVGFEEVVAQFLHACEGLPLSLKVIGALLRGKEDLEYWKYQL

SKISKVLPSDIQSRLKISYDSLDEEEKEIFLDIACFFVGQDRDMVIRIWEGSLGIRNLEN

KCLVQVDSGNRIRMHDHIRDMGRSLAEKEPPGYLRRLWRPIDNLTLQNVPHQSSVRGICI

VQRNSESSVELNRPVVDMSKLQLLRAEGDCLQSIFSQGRSPDLIWLCWDKCPYPFLPSWL

PIRKLRVLQMTGFRGEITEPLQSFWQSESQLPLELRELSVDVPLSKLPDSIGQLKQLERI

VLSQARMKFLPNSFGNLTNLQYLDLSWSFKLRELPHSFGNLIQLKHLRLAGCSELSISQG

TLGNITTLVSLDLSCCLKMNELPPQVARQRFLKELNLLGTSLRVLTGDIGELGRLESLKL

GSPFVKMLPLSLGDLISLKELTFSECTSLECLPESVGKLTSLKELTFFECTSLECLPESV

GKLTSLKELTFSECTSLECLPESVGKLTSLKELTL

>AB_030835_T.1

MASSTSTSTSAASPYNYDVFINHRGPDVKKNFASYLYHRLLIHGLRVFLDQQELQEGDDL

TPQIHGAIDTASVQVAIFSPTYAQSTWCLNELVLILETLEKGATILPVFYNVKPSDLRWN

RGEKGPYAEALHNLENKKTDEGKSRHDPEIIENWRNALSRLSDKSGFELAKYNGDEGELL

EEVVQRVLKKVKKSGLEVAKYPTGLDDKMKDFETTVLMRQRQRGKPQILGIVGLGGVGKT

TLAKEFFNRKNSDYAQSSCFLSDVREKTAKGSLHLLQRKLMKSLTGGTDIQ

>AB_030866_T.1

MLHKLLESVEKMAKKTALAVAQHPTGLDEKIKDFETTVLLSNQRTGKLQMVGIVGLGGVG

KTTLAKEIFNRKLSEYNKSYFLSDVRENGKSSLHTLQSKLLKGLNLFNGPIDDIHQGTEM

LRKSLSSSQVFVILDDIDHEDQVAALLPDRDGLRMDSLILITSRDKGVLTRSGVEESSIY

NLKGLNSKHSQELFCSYAFCQPYPLRGFEDLVNMFLRTCDGLPLSLKVFGALLFGEKDKS

HWKDILDRVHKVPNDILKRLRISYDALSPEEQEIFLDIAYFFIGENKNSAIKVWKGSLGF

RNLQGKCLVEVDSENVIHMHDHLRDMGRQIAKDRLWHP

>AB_030881_T.1

MAQGNTIGHEIGTGAAGGTLGGVAADFLNKGLVAPILQQFDDMINVDRNTKLLQVNVERM

RNHLQHISNMYEDQQRVIPESIRSWLGRMGDAIIEAIYLIKTFQRVQGDLEEFKIDRQVR

EVNTKFDNLFRDLATDFSVLSVQNIVSFAPLRADVLIQSMPTAGLVGRAIESAATQLQTW

INAVNTPPRVIGLYGMPGVGKTTVLGKVHNFYQARNVFDVVIWLTVSREYQLSALQGQIA

EAIGLDLSSICNPDRRTMMLSASLMKKEFLLILDDVWSSLVLKELGVLFGANECSKVVFS

TRDRNLALTEANEYSTKLEALIMQEGCELFEKVAFSGRPVPENLKERARKIADECKGLPL

AITVVAKAMRGEKTVDEWDDCLSMMKATDPDFRNTHPCVQDELYKILTWSYNEMGKTLGL

QSCFLYCAMYPEDEEISVEDLVRMWIAEGFVQSKKGSYSESMKRGYRYVKLLIDRGLFES

VNFEKQSKDTNKPVNVWKERFIRVHDVIRDMAIFIAEGNNYFCYAGLELKKFPSSQTQAC

KRISLRRNQVKSLPEDFPCTQLVSLMLSENPLVSLPTSLTHLRVLDLSNTLIESVPTSLT

HLRFLDLSNTLIESVPTSLTQLEFLTMRHTRIKELPKDIGNLSNLQFLDISECKYLTSLP

LEMCQLTSLKRLHLSMGMENAVKDPGNVMALKGLTNLIELHFFLFYKFEEYEIMGDILGS

WVEMKHLYLYYNHMNPSKCNLPRGMQNMKKLESLVLFHYRGTNLPDLICEFQHLERLVLD

SCSTVTELPLLEKLSNLKYLKLQNYSNVRDLRIGKPSGFPKLEMLHLSEMSRVRSLGMLE

TGTLPSLKVLKIKKCPRLRLSTGMKNLTSLRALYGHRISWWEKLIWEDGEMKEWLRTLFK

EI

>AB_030886_T.1

MVKSTATKRATILPVFYIVKPSDLRHNEKGAYAKALLELEQKKTKDCETGEEKPRYPSCT

IQNWRSVLSQAAEISGFVLEGDKEEELLTKVVEGVLENVPKTPLYVDKYPTGLEEKLQDF

ERTVLNEHEMRMRAKVVGILGFGGVGKTALAREFFNRNRSHYNESSFLLDVREKSARMSL

EYLQSKLIEDLKHIRNIKIESVHEGIEKLKRNLSSCHALIILDDVDDAWQLEALLPVDNL

SPQSLILVTTRDRHVLEQSRVSSIYNLTGLNTTHSKELFCSYAFHQSLPPPEFKDELVDR

FVTACNGLPLSLKVIGALLCGENEEYWEEQLDRLRQTLPEEIQKRLRISYDSLSKQDQPI

FLDIACFFLGQDKDKAIRKTPLVNKDKAIRIWGLVGIRNLENKCLLEVDSNNNTI

>AB_031033_T.1

MASSSTCTSTSTSHGNVYDVFLNHRGPDVKKTLASQIYHRLTERGVRVFFDIEEMQVGEE

LTTQIKDAIRTASVHVAIFSPRYADSRWCLDELLLMLETEKTIIPIFYDVSPSDLRHARE

RQAKEGVYAGALRVLEKKRRWDQKTPRYDSNIIENWRKALSSAADKSGFEHTSNSDDEGL

LVNKVIQRVLEKVPKTPLHVAKYPTGLDGKVEELERIVSSRQQSGNLATAVGIVGLGGVG

KTTLAKQFFNSHRSNYEGSCFLSEVREKASSNSLNSLQAQLLRDLTPQNKQIHIDTAAEG

IEVLKKYLPSSTRALIVVDDVDHYDQLQELFFPVKNTIHSNSLILVTSRNKDVLRSAEIE

ESSIYMLKALNKHHSKELFCWHAFKQPNPVKGFEQLVQKFLDACHGLPLSLKVIGALLFQ

ERDMKYWKEQLDKISNILPQEIQSSLKISYDKLDEEE

>AB_031143_T.1

MDVREKVARNELNSLQVQLIKELAQRNEQINTPDEGIEMLKKHLPSLDRVLIVLDDVDHI

DQLYKLFSPVKDTIHSSSLILVTSRNKDVLTSAQIVESSIYMLEGLNQQYSLELFCSHAF

NQPYPVIEFKQLVEKFLDACHGLPLSLKVIGALLCGKDLTYWEEQFHKISKVVPTDIQSR

LKISYDGLDDEEKQIF

>AB_031193_T.1

MAEALILGAVASAAASAAGDDIVSLIQVLIDRIDNVRRLDENRELLEDQLDRMKRMLRGI

ETQFRDQHREVPDSMRNCLERMQRKVEESEELIRRSQCSWRQKCIDFLCCNPNVYTEIQE

SKTSFDDFLSGLEKDIAAIHHVQQIVAAAPQEAGVLLQDEPATGLIGVDIKSAETRLQGW

LSEATHIRTFAVYGMGGVGKTTLLKKVYNNYCNAGHIFDRVIWVTVAKFTITNIQDQIAS

AINLDLAKFSDDIRRMKLCASTKNIKFLLVLDDMWSAIDLMRDLGVEFGENKGSKVVFTT

RNRELIGEMNAKLCMQIQPLLPEEGWELFSKIAFEDGRVPEEMEDIARRVAKECEGLPLA

IQVIASTMRAKTTAVEWMHALKQMQKVDPNFPRNHPRIDRELYQKLKLSYESLPGDDLKN

CFLHSAMYPEDTQIEVKYLVQRWIAEGLVNTNGDEEYDYIFETGQSYVKLLQNRGLFSVG

WADVAIRVHDVVRDMAVYVGEKEENCVFRAGQSLRDFPHIRSDNCK

>AB_031285_T.1

MEETIGLIVESCIMCCSQMASSKYFKFLSASVNKEKLGEFRKKLDDMHKHINMQMGICIY

NISERKKAPLSKLYPEHAVGLQDSVNEVIDMLEWGSEQNALTVILHGFGGTGKTTLADAV

FSLVRVEECQYSHVQLFKDIKSSPDILELQKLILKDLMGSENIPDIRKHQDGQRELGNML

QEKRAFIYIDNVLGENQLRELLPKNMNQSKKVRLLLTARDENVGIVCPMETAHVKMYHMR

GISPQEATNLLKKDIKDDIDPSQLNEIVEVCGGIPLMLTLVGPFIGHARNKQQAFRRLMQ

EKGRLKNKPFDGIERYLFAYDDLPDECKDPFLDICLFFKGWDWDTVADIVGVYPLEMLES

>AB_031424_T.1

MASASSSRPASLSESSYQIFINHRGPDVKKTFASHLYRWLVSRRFTVFLDQEGLQVGESL

TSQIQTAIGNASVHIAIFSRGYAESSWCLKELVLMLNTGKPILPVFYHVTPADLRWTLDE

NFKYTRAIRDLQMKKTYDSQTLEEKP

>AB_031500_T.1

MSSTRSSDYRYHVFISHCGQDVKDKLASHLYRVLELRKFRVFLDKVSLQVGENSTTKIEE

AIASAFVHVVIFSPNFASSPYCLNELLQMLESGAPIIPVFYHVKTSDLRWTSGDKGWFAH

VLEIIPWLTTSEDGVYASALRDLRRKRTYDADTHRNIPLHAPQIIKKWTEALSAAANIEN

LDLENCDKGVEAKLINEIVQRVEKFVRKAAFPMPKYLIGLNNWLDEFKNTISRAPQLPGE

AKVVGILGSGGIGKSTTAKEFFNRYRSDYDRSSFSCDVRKYPLIDLQRNLVKDLTSCDLK

IDNIADGIEKLSRHLLDIQALVVLDDVDNVEQLNALLPPMRAVLHSDSLIVLTTRNQNLL

SRLKDSSIYEMRGLNPEQSKKLFCWYAFHQACPFKGFEKIVEDFLDGSTGLVLSLKVLGA

FFFGNNLRNWEDKCFNISKMLHIRIPRWALLDIACVFLGEDRNTSIQISLQGRSLVFTNH

EKLCLINQKHLRMRDHLIYFDR

>AB_031527_T.1

MNERHFTSQICPRNLLLNLYLHLISLFIPLKIMPPTLDSASASTSASPRGFDVFINHRGP

DVKKTIASHLYYRLLSLGLQVFLDDPKLERGEDYPPQIAGAIATASVHVAIFSPGYAQSR

WCLDELVDMLESKESRGATIIPVFYKVDPSELRWTDKGKYG

>AB_031528_T.1

MVAQKVLKKVSKPPLPVSEHLTGLDEKLQDFEKTVLLEQQHQRRKANVVVIVGPGGLGKT

TLATIFFNRKKPDYQQACFLHDVREYAVKNCLNTLQMKLYKDLTGRDQVMDTVYEGIAML

KNRLLCGQALIVLDDVDDDKQLDALFSPVRDVLPPGSLIIVTSRNPEVLARSGIEKSSFY

KMTGLDKEHSRILFCSYAFGQRGPHEGFEEIVTKFLDVCAGLPLSLKVLGALLRGEKLKS

WKAQLDKISEAGLPTDIQRTLKSSYDSLDKEEREIFKDIACFFVGIKRDTAIRIWDGCGW

RGWQGFRNLENKCLVQVDSNKCIRMHDHLRDLGRHLAEGETPECERRLWRPTDEHFPNVL

TSSTERGISVVQQRSGESFQSTVEFAHRIVGMSGLRLLRIPGDYLRSISMVTEQPDLLWL

RCENYKDSSLPSWPMPKLRVLEVEGREGEVEAASCLETLWETEIEAPLELRELNIRVPVL

RVPQSICQLKYLEKIELHNIKETLPEKFSDLRSLKHLKLCGQMVSLPNSLGDLTNLQHID

LSLCRNLKMLPPSFGRLSRLKHLSLEYCLNLTLSDETFGNITALKRLNLTFCKKIERLPS

QVTRQQSLKELILNDTAVKELPNAIGNLRKLKILEIAGPPLKILPPSLGMLQSLEQLTLK

FCKDIKYLPSITDWKELTMLKMVDLTVECLPVGVTELENLESLVVENCPIREVPFASVEK

VQGGIEELLDVRGRRLLDPIHNYMLGLRYLYLRGTRIIELSFAEGVCPNLQTLDLGGCRI

LVEVGSLPTTLLSLRLRGCRALKKLTGLSRLAKLQTIDMRECTKVRALPGLEALISLEEF

KTSKCNNMKRIRDLAWKSKPESHILKKLIKIMETQEEEEDEEQQQERNHALECLMLLQQL

QQEQGHALE

>AB_031540_T.1

MRVAGAETLLLNSEAIAGYMNERHFTSQICPRNLLLNLYLHLISLFIPLKIMPRTVDSAS

ASTSASPRGFDVFINHRGPDVKKNIASHLYHRLLSHGLQVFLDEPELERGEDFPPQIAGA

IATASVHVAIFSPGYAQSRWCLDELVDMLESKESRGATIIPVFYKVDPFELRWTDKGKYG

EDLRHLETKRRYDRQTVAKWRGALAQVSDISGFKLEDCNRCTWIYNGCSFEWSCICRRMA

TRSFHF

>AB_031669_T.1

MANLPAAASCSTASSSASASSSGYDVFINHRGPDTKNTFASHLYRGLLRCGLRVFLDKQE

MEKGDEIQSQIVQAIRDASVHIAIFSPRYAESHWCLHELVLMKNSGATIIPIFYKSDPSV

VRWTENGAYAEALRGLEQKTKKNPETGEVKPRYGSDTIHNWRDALSCVADLNGFTLLGDQ

DDEGEVLDKVVENVLKKIPKTPLDVAKYPTGLELKLEEFEKIVLSQQQQEEERVEAKVVG

IVGVGGVGKTTLAK

>AB_031765_T.1

MLAVHEKFNNAKEIQGYLEKIDDFIRIEGPPNLALDSQQLRLEVGFLRRQLAQQELLSRQ

NISPTQTNIDGIHGAQAAERSNSINSQVPPKPEKALGLYERIDEVKQILNQRDVNIIGIT

GMGGSGKTTLASALCHDPGVKDAFQSNIHFIIVSQFHGNKNCVCEILEIMCHSIFGGEKP

KFQSIEYGRNQLQNNLNRNAHRKTLVVLDDVWPGLSVTNLLFTAEGYKTIITTRHNFIIQ

YIGGSLYEIPVLNAADALSLFCFWAFRQASIPPTANGDLVREVAAECKGLPLALKVIGSS

LRSKHPSTWEIVKEKLSKSEPISPDHEAELVCRLKTSIDILSDKERQCFLDLGAFPEGRR

FGVGSLLDIWIYVRKMTWLDAFDVLLELASRNLMRLTGYGEGCAAINYHCASELSFSQHD

VMRDLALHLSNQDSNILCKRLFMPRKEANIPTEWLRAAQSSSAQFVSIHTGAMKEEDWGQ

IDLPKVEALTLFFDAKQYCLPTFLQSMSNLKVLIVYNYSSQRVTLSGLPSFPSSVQIRSV

LLYKLIVPSSFYENCRSWERLEKLYICLCGDLGNITPVDKDKESEALNFPKLLEINFDHC

SELRELPIKLSNITSLQMLSVTNCHLIKNLPVDLETLSSLRVLRLSACTSLSRLPPSISK

LDQLEYIDISICTSLEGLPAEFDHLPNLKTLDMRECSRLKKLPNVKPRSLERVIICEAVK

ESPAAWSSIFVAVAQSFSLDWLDEH

>AB_031775_T.1

MVGIVGLGGMGKTTLAKEFFNRKCSEYNKSCFLSDVRENAKSSLHTLQSKLLKGLNLLNG

PIIDDIHHGTEMLRKSLSSSQVFVILDDIDHEDQVVALLPDRDGLTNDSLILITSRDKDV

LARSGVEESSIYELKGLDSKHSLELFCSYAFCHPYPLGGFEDLVEEFLRACDGLPLSLKV

FGALLSCQKEKSYWKDILDRVQLPTDIQHRLRISYDALSREEQEIFLDIACFFIGKDKNT

AIKFWKGSLGFRNLQGKCLVEVD

>AB_031823_T.1

METKIGRHEGTGQFKKHLSLCHALIILDDVDHDTQLEAFLPLKNIMNPNSLILVTSRDKQ

VLKSSGILESSIYNLEGLDMTQSKKLFCSYAFNQPQFAPPEFSDLAPPELADLVDQFVTA

CKGLPLSLKVIGALLCGENDKSYWQEQLDRLNHALPQKIFDTLKISYDSLDKQDKPIFLD

IACFFIGEDIEKGKGIWGKAGLTNLRNKCLLEVDTKNKIKMHDHIRDMGRMIAEEPTMPR

RLWNSTTISNILVEQPSALITDVRGIKMVGTYPDYPLMRSGISPSHCPGMKNLELVAAEG

DHLEDMLRRVQRPNLRWLRWYGSSHSSLPDWIPMENIRVLEVEGERLKIPWPHQSQGVPL

QLRELNILARLSDIPKSIGQLKYLERIAIFNPNSLGALKRLPNEFCRLGSLKYFAFRLFS

KMVSLPDSFGELTNLEHIDLEGASSLQTLPDSFGKLTRLKHLSLRGCKNLTISSGTLGNI

TTLEYLNLSSCEHVNKLPPQVAHQLSLQHLYLSGTKLKELPSFGCNLKVLELGGDLLEVL

PPGLGDLKMLETLSLFNSPQLKSLPDSIGELTRLTELVISRCGIEYLPRSLLKMNILESL

KVLDCPLLEYPFKKEVEEERETKEETALNKLNSSNYRCMSGLKRLSLSGKKIREVYFDKG

VCPNLQRLTLGGSELRQIGGLCALPKLRDLQLGDCAKVEELYGFETLTSLESLDVSQCEK

LQSIPGLGQLTKLRTLRVSSCREIQGLPGVEHLTSLESLDVSQCEKLQSIPGLGQLTKLR

TLRVSSCREIQG

>AB_031842_T.1

MAGVGKTSILKMVYNHYLEVDSDAFQHLIWVTVSTGYNPLDLQARIAKEIKLDLGPHGAD

SDTHKMLSAHLKEKKFLLVLDDVWDPLDLQELGVVFGEGKQSKVLFSTRRRDLILGTRSA

EKSIEIQPLSPDEAWKLFRRTAFNDDTVSVSMEIVNCAREIAEECKGLPLAITVIAASMI

GKTRADQWKNSFDLMKTADPYFPKTHERVDPELYQRLRCSYHALPSLEVKNCFLYCAMYP

EDYHIPVDDLVQMWISEGLVGRGRESHLLDMAIGRSYVDILVDRYLFQNAGYEKYDRPWR

FVKQRKIIVHDVIRDMAIYIGEKDENCVFKAGQQLQNFPKIPGDCKRISVFDCDIENLPT

EFTSPKLASLLLCCNQIGVVPLGFLNSLTSLKVLDLSATSHSTSRGLMIPSSVGQLKQLE

FLRLNDFRRMEDLPEEVCNLSSLQFLDLSRCGLRSLPSQIGELKTLKYLNLSYCQSLRVI

PHEISQLTSLSTLNTYASGVRFNVEAESEASMWSLKGLVNLAMLTVNVWVDVQEGMTSSG

IKVGIMGTWLEMRHLWLHFRVENDVNVVLPEDMQKMSKLQSFQMSDYNGLSLPDYICNFQ

QLEHLELSRCDHLTGLSPLERLPNLKYLMLDRWYNLKELGIGNSRKASGFLMLEELVLSR

LDGLESIAGASNKGVWNELILPKLRVLYIHRCPSLKRFPKGMEKLVSLNRINGSNLWWDR

IEWEDVNMSSHLQNLFQR

>AB_031910_T.1

MATKPSPPAASKKASVLRRLNAFKMADSTKSVIKNINEDIAENEDVDLEKLMEHINQLLK

LREQQNQLYKKPSTVSKWTWPKSSGLKKLKDWLQSGIDVCSALKDIISGPVKEILKGIGH

ASLATTVLLVVAYSLERYEEFSSNKEECLKVLKEMCYVANLVKIIKESPKGLQNELEEAT

TLIVEGSIICCAQIGSSSWKRFLIASENKDELSNVTKKLDRLYQKINLQIGFRVLTAIES

KRQESYLWRGYPTHAVGIDKSITEVLDMLKCESEEKAVTVILHGFGGMGKTTLADAVFSR

VGGCKYSHVQLFEDVNAKPHIVDLQKQILKDLMGTEEPVPEIRKYQGGQQALKSVLQRVE

AFIYIDNVLEGNALKNLLPEDMGTAKKVRL

>AB_031921_T.1

MVTKPKPDLHVAKYPTGLDEKLKHFEDTVLLQQQQRGKPEILGIVGLGGVGKTTLAKEFF

NRKKSKYQKSCFLFDVREHATKDKLTSLQGELLKSLTSTEVPISSVDEGKMMLIKHFKDP

EILDDVDPEILDDASNALIILDDVDNLDQVDALL

>AB_031934_T.1

MVPLFDNIDSPPKIIELQKLILKDLMGSENIPQIRKHEDGQRELSRMLEEKPAFIYIDNA

LSERELAQLLPEDMTEAKKVRVLITARDLNVRKACPLKTVREYRMRGISSVEATNLLKME

MFDIDDMEGILHSYKVNQINHIIERCGGIPLMLKLVARALLDAEDEQEVDAVLDELKKLK

GEDFGPDKIESYFFAYDKLPRECKDPFLDICWYFEGWDWDTVANIMGESEVEMLARRALV

TKDTNGVVSVHDVILTLGRRKSHGVRFIFTNASQIKRILDQEEELKHVQKIKGIWLGENK

DLFTISATILDSMHKSLRILKLGNLTRIEGKCNKIFENLIFFQGAVPYIPFDTRELKYLS

YQPKDLKLKEIPQSLRHMEFDGRLHSHAFEISAKELKHFENLKVLRLTRFARLKKLGDLV

DGLRELTLSHCQSIEELPSSISMLPFLRILRLNYCSNLKHLPH

>AB_032090_T.1

MVRLFDDINSTPNIIELQKCILKDLMLGSREEDQKSMLENIRTFEDGQREIGRMLEKEVA

FIYIDNALHKDPLEKLLPKGMEKSRKCRLVITTRDKAVRKGCSMDDVKSYPVKYLPSTEA

MILLKQELNYEDTDNRKRLNSDQLHRIVNLCGGVPKLLSVLARFVRYDDRGQQKAFSIVM

KDMEEKKEWNVEIGDIGSYLFAYDHLPERVKDPFLDICSFFNGWDWDEVTDIVGESNLDV

LEKRGLVKKDRDMTVTVHDVILTISYKKTEGTRFIFNSKSEIEEFLKKDIQGIKGIWLND

IQGIKDIRLNHIHTISIPATKLNCMSHSLRILALGDSAEVDGTCENVFEEIVFFQGEVPH

LPFHVSHSKELKYLRYEPHDWNLLKEMPSNLKHMELNGRLHSEDAFKILSSDLQQRRSLR

TLKLVNFARLKNLPEVWAILSHCLQKLDLSDCGS

>AB_032279_T.1

MTGLNKEHSLDLFCLHAFHKPQPVVGFEEVVAKYLDACDGLPLSLEVLGSQLYQKDLEYW

KAQLLKISDHLVLPRDIQSSLQISYDSLDPHEREIFLDIACFLIEEDRDLALRIWDGSDW

DGSLNLQNLEHKCLLEIDGKNCIRMHNHFRDLGRDFSEKELPRCHRRLWRRTDNLFHNAF

IQPIVRGINMVQETSGQSIRNHVELGNLSVDTTKLHLLKAEGDCLKSISKFLERSDQLRW

LCWNKYPYNSLPPWIPTRNLRVLQV

>AB_032328_T.1

MPAKSVVQDLLKGIGQVHSAVAVLLVVANILERFETLSANQNECLSVLEEMSNLAQHVKE

LKERSGLKKAMETSIKRATEMIVEGALMCCTQMSASRISKYFSSTVNKEDLAKFQQRLQK

TYRRMDNQMLICILDAVESKNVRPPVKGRERSYPEYAVGIEEQLEEVIDLLEWESHKNVL

AVMVHGFGGMGKTTLADAVFARLYIEGCKYSMVRLFDDINSTPNITELQKCMLKDLMLGS

TEEDQKSMLENIRTFEDGQREIGRMLQKEVAFLYIDNALHKDALEKLLPKDMEKFRKCRL

VITTRDTEVRKGCSMDEVKPYPVKYLPSTEAMIFLKQELNYEKTDHRKGLNSGQLHRIVN

LCGGIPKLLSVLARFIRYEDRGQEKAFSLVMEEMEEKKKWNVRIGDIGSYLFVYDLLPEI

VKDPFLDICLFFNGWDWDKVSDIVGESNLDVLEKRGLVKRDKYMTVRVHDVILTVGYEKT

QGTRFRFNSKSEIEEFLKKDIQGIKGIWLNDIQGINHIRLNHIHTISIPATKLDCMSHSL

RILALGDSAEVDGTCEKIFE

>AB_032404_T.1

MGGVGKTSLLDTINDSQNIRNSFELIIKVTVSKNHILDMQDCVARRLNLTGFPDKSNFEE

RKDMLRSSLKDKRFMLLLDDIWADSALWTTDDLQNLGVSLNDRGFKFVLTTRDKGVCTRM

NVQEMITVEPLSEEEGWQLFRSRAFQNRDGNVPQEIEEIARQIAKECKGLPLAIIVVAAA

MTQHTDRLDE

>AB_032540_T.1

MKKYTSRHQWEDALEQMQTVDKSFYDMHNEIGLKWSYDDQREEGLKICFLYLAACPKGKV

IRYEQVIDIWMAEGMVSGREASHLFIDSLGGRCLI

>AB_032583_T.1

MLTTFWVDMLLSSFCLKIWIKQKKLRLLITTRDTDVSRECRKLKTKIYPMKGLPHEAAKS

LLEKEMYSETDNVQKQLSSDQLDHIVQMCGYIPKLLTVVAGFISCQDDKQKAYRIVKEEK

EKNWKGEIGKDITHYVFACQNLDERLKDPFLDICSFFKGRNWSEVADIIGDGELNSLEKR

ALVTRDTTSRVWVHDVILAMGLQQTEGTRFTSKSQIKEFLDQKEDKVRNL

>AB_032639_T.1

MASSSEIEIEVENQPSPATPHASSASSSAAADPTTSSRNVFINHRGPDTKKTFASHLYHR

LLSHGFTAFLDLEELQEGFHFPSQIEHAIRSASVHVAVFSPKYADSEWCLRELLLMLESR

APIIPVFYHVQPADLRWTGEDKTGVYAEALHRLQLKKTYCSQTHQDKPRYDPATIQKWRD

ALSSVADIS

>AB_032701_T.1

MIFLAKVVKQIKERPQLQERMQHEVKDATELIVEGSIMCYSQMGFSQFSKFFSTSVNKEQ

LGELAQQLDAKYKHIYMQMGIQIYDVSQCKKANLSTRYPEHAVGIQDAVREVIDILEWGS

EQNAIAVILHGFGGMGKTTLADAVFSVLNIEGCQYSKVQLFDNIYSPPKIIELQKMILQD

LMGSENIPQIRKHQDGQRELSRVLEEVTAFIYIDNALGERELLQLLPDNLSKAKKVRLLI

TARDLNVRKACPLKTARKEYRMRGISSVEATNLLRMEMFDSRERKLRKLHSYKVDHILKK

CGGIPLMLKLVGRALQFAKDTQEVDDVLDGLDKLDFGRDKIESYFFSYEMLPTECKDPFL

DICSFFDGWDWDTVANIMGESELKMLAGRALVTKHTTGVVSVHDVILTLGRRKSHGSRFI

FTSASQMKKILDEKEEKDLHSIKGIWLSENKDSENKDIFSVTTPLLDSMHQSLRILKLGN

LTRIEGKCNKIFTELIFFQGVVPYLPFETRELKYLSYQPEDLKLLQIPRGLRHMEFDGRL

HSNAFEISSKELDQLKNLRVMRLTRFAKLTKLGDLVNSLRELTLSYCQSIEELPPSISRL

PFLKILRMDYCSNLKHLPEDLGSLNSLQEFNLQGCTSLEELPNSFGKLLSLKLFNLQGCT

SLEELPNSFGKLSSLKLLDLSFCENLSGIP

>AB_032718_T.1

MLRKSLSSSQVFVILDDIDHEDQVAALLPDRDGLRMDSLILITSRDKGVLTRSGVEESSI

YNLKGLNSKHSQELFCSYAFCQPYPLRGFEDLVNMFLRTCDGLPLSLKVFGALLFGEKDK

SHWKDILDRVHKVPNDILKRLRISYDALSPEEQEIFLDIAYFFIGENKNSAIKVWKGSLG

FRNLQGKCLVEVDSENVIHMHDHLRDMGRQIAKDRLWHPRENIDDWLQQQSSVISEARGI

RMDGNNEVANVLIRSPMFSWFQWKFDKVFGNCMKWFQSMIDNVFGNCMKKRPGISAMKLQ

LLSSEGHLLERILRRVHSPNLIWLSWRDCPYSYLPSWIPM

>AB_033008_T.1

MLDKLVVHVVKMAKKTPLHVAKYPTGLDEKVEDFEAKVLLRHKRTSKVQMVGIVGLGGVG

KTTLAIEIFNRKHSEYNKSYFLSDVREKAKSSLHTLQSELLKGLNLLNGPIGNKHQGTEM

LRKSLSSSQVSQVFVILDDIDHPDQVDALLPDRDGLPNDSLILITSRDRGVLVRSGVEES

SIYELKGLNSKHSEELFCSYAFCQPYPLRGFEDLVNMFLRTCDGLPLSLKVFGALLFGEK

DKSHWKQILDRVPDDILDRLRISYDALSGEERQIFLDIACFFIGKDKFTAITLWKGSLGF

RNLQGKCLVEVDSKNVIHMHDHLRDMGRQIAKDESLRRLWHPTENIDDW

>AB_033048_T.1

MAQHQASASVASGSSSQVYSHFDVFINHRGPDVKKGLASHLHTRLRVHGLRVFLDDREME

QGHPLTPQIQSAIRTASVHVAIFSRRYAKSSWCLNELLMMLDSGSTIIPVFYGVNPSELR

WTRGEDGVYARVLRWIQGGDGVYARDLWKLERKKRYDSNTIENWRKALSDVSHISGFELK

ACNGDEGLLLNQVVRQVLNKVPKPPLVVALYPTGLEDKVKEFETTVLTQQQSGKTGAVGV

VGLGGVGKSTLAKEFFNRHRSNYDRSCFVYDVRENAAKNSINLLQATLLKELAHRDEQIN

GADEGIYKLTQYLPSSHRALIVLDDVDHLKQLEALFLPVKDAMKPGSLIVVTSRNRDVLI

NSGIPQSSIYRLTGLNPQHSRELFCSHAFSPAASVEGFDEVIKEFLDACQGLPLSLKVIG

ALLSGEGDLNFWKAQLRKISRILPDDIQSTLKISYNSLDEWEKQIFLDVACFFIGEDRDT

TIRIWDGSEWEGLLSFRKLENRCLVDVDSENRIRMHD

>AB_033070_T.1

MASSSNSSGNNSSPYQVFINHHAPDTNHSIATHLYDRLHDDNGLQVFLCNEEMEKGDFLT

SQISAAIREARVHIAIFSPGYADSEWCLDELLLMLESMNLRQSTVIPVFYLVKPEDLRWA

LGENRKYAEALRRLQEKKTTDFKTHQEKPRDDSDAIEKWINALNTVSYISGFEYDGVEGN

LVDNLVEVVLGKVKKPLHVCNYTTGLDQKVKDFEVLLQQQSGGPIVMGIVGLGGVGKTTL

AKEIFNRLRSNYQHSSFLFDIRQNSLTTSQIDLLKDLSPKSNVQIRSEDAGKEILKKELK

SKEAVILLDNVDSAQQLEA

>AB_033154_T.1

MAGVGKTSLLKVLYNHYIEVSNVFQHVIWVTVSADYKVLDLQARIAKAINLDLPSNSDID

TQKMLLSANLKEKRFLLVLDDLWHPLNLQELGVAFGKDKQSKVLLSTRNKDLITQMRAEK

SIEIQTLSKEEAWMLFRTIAFNGDTVSVSMDIMECASKIAEECKGLPLAITVIAAAMIGK

TSVDQWEVSLDLMKTADPSFPDTHPRVDPELYQRLRCSYDALPNSNIKNCFLYCAMFPED

YEIRVRNLLQIWIAEGLVRSKQGSHFMDMAIGRSYVNLLVDRCLFQNVRSGENIYDGWDV

LDRQRIRVHDCLRDMAIYIGEKDENCVFKAGQQLQNFPEIQNEDCKRISVSSNQIQNLPT

LFTRPKLVSIFLNDIPIEEVASGFLVSLTSLKVLDLSWSNISSIPSSVGELKQLEFLNLS

RLEYIWDLPAEICHLSSLQFLDLSECRCLQSLPSRIGELKTLKYLNLTKCKSLRVIPHEI

SQLTSLSILDAYSLEAAELEASIWSLKSLRNLTMLRTRVKVDVQEGMKSSGIKVGIMGNW

LEMRHLCLIFTKGDYVMEDLPRDMQNMNKLQSFTIWDYNGLSLPDCIGKFQHLEYLNLSY

CTHLSHLYPLEHLPKLKHLELWWWPNLKELGIGSSGNARGFLMLEKLVLHMLKRLESIAG

LSNNGVWNESVLPKLHTLTIFNCPSLKRLPRGMEKLSSLKFKYDNGGRRLVAKHRMG

>AB_033230_T.1

MASTSYYSYRSNDPVFDVFINHRGPDTKETLAKDLYDQLKHSLRVFLDYGELKEGEEITP

QIQSAIRTASVHIAIFSKRYCESSWCLEELFLMLQSGSPILPIFYGVKPSELRWGTQGEN

GVYAQALHTLAKKRTFDSQPRYDKSTIEKWKKALFDCAEISGFDLETLNGDERKLLQRVV

ERVLKIAKIPLHVSEYPTGLDEKVKDFEGSVSLQQQSSETRVIGIVGLVGLGKPP

>AB_033380_T.1

MKVVQDLLTAIGQAHWVAAGFLVVANILNRFETVSANQDECLSVLEEMNNLAQHVKDLKE

RPWLKTAMEASIKRATEMVVEGALMCCTQMKSSTFSRFISSSVNKDDLDKFQQKLQKMYR

CMDNQRFICVLDAIECMNVRPPVKERERSYPEYAVGIEKQLEEVIELLEWESNKNALAVM

VHGFGGMGKTTLADAVFARLYIEGCKYSMVRLFDDINSTPNIIKLQKCIVEDLMAGSREE

DQKSVLKNIRTFEDGQRAIGNLLEKEEAFIYIDNVLGKDALENLLPRNVEKAKKLRLLVT

TRDKEVRKGCSMNAVEFYPMKFLPDTEAMNLLKEEMHYGKDEDMALLNADQLYQIVSLCG

GIPKLLSVLARILRFEEGGQQKAFSIVMEEQNKWNRQIVDIECYLFAYDRLPERVKDPFL

DICSFLNDMDWNKVSDIVGSLI

>AB_033412_T.1

MKTLFNRDKVRDLFRDGFLLWLTISGSPSILSLQKELFTQISMQRKVIDPNTNMNEEAIA

IGQLNEALQGKRFALFLDDVWEKGAKLLEDLGVLRLIDQPPYSKIIVSSRDSRVLRGMGV

AKESTITVGDLIEEDSWKLFRFYAFPYNNGNVAVGIDE

>AB_033454_T.1

MYRRMDNQMLICILDAVESKNVRPPVKGRERSYPEYAVGIEEQLEEVIDLLEWESHKNLV

AVMVHGFGGMGKTTLADAVFARLYIEGCKYSMVRLFDDINSTPNIIELQKCILKDLMLGS

REEDKKSMLENIRTFEDGQREIGRMLEKEVAFIYIDNALHKDPLEKLLPKDMEKSRKCRL

VITTRDIEVRKGCSMDEVKPYPVKYLPSTEAMLFLKHELNYEKTDDRKRLNSGQLHRIVN

LCGGIPKLLSVLARFIRYEDRGQEKAFNLVMQEMEEKKKWNVQIGDIGSYLFAYDLLPEI

VKDPFLDICLFFNGWDWDEVSDIAGESNLDVLEKRGLVKRDKYMTVTVHDVILTVGYEKT

EGTRFRFNSKSEIEEFLKKDIQGIKGIWLNDIQGINDIRLNHIHTISIPATKLDCMSHSL

RILALGDSAEVDGTCEKIFEEIVFFQGEVPSPPFHVSHSKELKYLRYVPHDWNLLKEMPS

NLKHMELNGRLHSEDAFKILSSDLQQRRSLRTLKLVNFARLKNLPEVWAILSHCLQNLDL

SGWNSLEELPGSISKLQSLTELDMSNCSSLKRLPEDFGSLTSLQYLNLSGCNSLEELPGS

ISKLQSLTKLYLWNCSSLKRLPEDFGSWTSLQNLDLSGCNSLEELPGSISKLQSLTELD

>AB_033480_T.1

MLDKLVQRVEKMAKKKRALDVAQHTTGLDQKIKDFETTVLLSNQRTGKVQMVGIVGLGGM

GKTTLAKKFFNIKRSEYNNCCFLPDVRENSKSSLHSLQNKLLKDLNLLNEPIDNIHEGKE

LLRKSLSSSQVFVVLDDIDHEDQVVALLPDRDGLRNDSLILITSRDKYVLARSGVEESSI

YELKGLDSKHSRELFCSYAFCHPYPLGGFEDLVEEFLRACDGLPLSLKVFGALLFCQKEK

SQWKDILDRVHEVPNDIVDRLIISYDALSGEERQIFLDIAWFFIGKNKNSAMKLWKGSLG

FRNLQGKCLVEVD

>AB_033616_T.1

MAHSNFPQRSSAASSGSASTSYAHDTHYVYDVFVNHRGPDCKETFATDLHKRLSGYGLRV

FLDEQELQKGEELKSQIKGAIREASFHIAIFSQNYAQSQWCLEELVLMLESKKTILPVFY

DGVNPSNLRWTRGGVYAQALQELENKRTVDNETHEETPRYPSDTIGKWRQALSVAADISG

FELAKYKGDRHQLVEAVVEEVLRKVKRPVHVSKHPIGLDQKVRDFETTVSLQPQSRETRV

IGIVGLGGVGKTTLAKEIFNRQQSKYDRSCRLSDVRDTDLKSSQSKLLADLARWNGQIYD

TDDGIGKIKDYLPRDPPTLALLIIDDVDDIKQLNALLEPV

>AB_033631_T.1

MSLVNLQMKLIKDLTQREDIKIESPDEGISELKKHLLYCHALVIVDDVDKADQLEVLLPI

KDVLSSNSLILVTSRDKQILVSSGIVDSSIYKLAGMNRAHSQDLFCSYAFYQPHPLPGFT

DLVELFVTACDGLPLSLKVIGALLCGRNERSYWQERLNELKILPPDDILQRLKISYHSLN

QMEQKIFLDIACFFIGEEMGRAIRIWGQVPVLGIEDKCLFEVDSVNKIKMHDHVRDMGRC

IAEESSMPRRLWLPLTNNSIDTLFEQPMSGITEVRGILTVHPYPYPWYFYRSKMSRYKRW

SDELLGNRKRTLFNKDSHYDGRSFQILASEDGYIKAILRRVRSPHLILLRWYKCPYSSLP

SWLPMEHLRVLQVAGERLNKLWQHESQAPLQLRELNIRGPLSHVPKSIGQLKHLEKIEIV

GIVGLDPRVKTLPEEFFDLRSLKYLDLSWSLCLKKLPDSFGKLTNLQHINLTGASRLKVL

PNSFGNLTRLKYLSLRGCSDVTISNETLGNISALEYLDLSRCNEVKELPPQVARQRSLEK

LYLQGTMLKELPSGIGNLCNLEVLEIGGPLVELPFKKAEGERETRVALNDLDSASNNKYM

FGLEKLSLSRVAIRELTFSEGVCPNLQALELSLCHHLRQIGRLCGLEKLQHFKIHFCNEV

EELPSLETLTSLEEMDVYECRKLKRIQGLGQLTKLRKLIVRYCPEIEELPDLEHLISLEV

LNADGCHKLKSIEGLGQLTNLQTLLLKGCHQIQALPGVEHLMSLERLNVRGCHKLQWGEG

VVGQLRRRMEKGFEYDKQSKIRRFWNALD

>AB_033714_T.1

MGKTTLADAVFARVYVEGCKYSIVRLFDDVTSTPSTADIVKLQTYILKDLNMERPEETPA

EIRTPEEGQQEIGRILQKEKAFIYIDNVLQRYPFEQLLPRDMDKAKKLRLLITARDTDVK

RECRKLETKIYAMKGLPLTEAKSLLEKEMYTSYTDNMENRLSSAQLDEIVKMCGYIPKLL

TVVAGFISCEEDKQKAYIRVMEERERNWKGEIGMDIAHYVFAYEFLDEGLKDPFLDICSF

FKEWEWSKVANIVGDGVLNSLEKRALVTKKTQTVWENDVIQVWVHDVILAMGDQQAEGTR

FSSAKKIQEFLDEKEDKDIRKIKGIMLQTGKADLRPLYISASNLDLMCSSLRVFRIKHYA

KVEGTCEKIFEKLVYVEAKVSDLPFDVSTSKELKYLDVWDDWEEVKELLEDDSNQGLTYS

KCKYI

>AB_033763_T.1

MPNFVVGLNNIVNDVKQILFQEGVSIVGVEGMGGSGKTTLALALCNDPKVKDFFQKNIVF

ITVAHAPNVKTLLEIMWDKIIGGRRPVFQSIEDAHDQLQKKLSSKGNLQLLVVLDDVWSK

SDVEQLLFEAKGCKTVITTRQDHTIPHTDSSRVYNMPMLQKADALSLFCFWAFGQSSIPI

TQDEVVVKQVEAVCMGLPLALKVIGSSLRNEPQPVWENAKKKLSRAESISKHHRGELLHC

LETSIDVLDDETKECFLDLGAFPKGRKICVDSLLDIWVYARGMDWKDAFVVLLELASRNL

LNLTSDSRNLAISCDCASELFFNQHDVMHDLALRLASRD

>AB_033893_T.1

MLKKHINNLKSFNSSLMGIVSKALVILDDVDNANQVYALLPVDLPLGSLILITCRDKHVL

LTSGVQNSSIYMLTGLNHHYSRELFCSYAFSQTYPHQGFKSLVDSFVKTCNGLPLSLKIF

GANLYGRDIPYWEDQLGSFPSDIGDRLKISYNALNFEEKQIFLDTACFFIGQNRDTAIST

WDASGWKGRLGFQILQDRCLVEVDEWNSIHMHDHLRDLGREIAEVRLWRWTEKVIGNLLQ

QPSVIRVRGIKM

>AB_033914_T.1

MMLLESLKKKEFLLILDDVWSSLVLKELGVLFGANECSKVVFSTRDRNLAVTEANESTKL

EALIMQEGCELFEKVAFSGRPVPENLKERARKIADECKGLPLAITVVAKAMREKKTVDDW

DYCLSMMKATDPAFPNTHLCVQKELYKILRWSYKEMGETLGLQNCFLYCAMYPEDEEISV

EDLVRMWIAEGFVQSKMGSYSESMKLGDRYVKLLIDRGLFESVNFEKQSKDTIKPVNVWK

ERFIRVHDVIRDMAIFIAGENNYFCYAGLGRTDFQFKRTRGCKRMSLRSNLLKRLPDNFP

CTELVSLILSKNPLGSLPTSLTRLRVLDLSNTQIESVPTSLTDLEFLKMSHTYLKELPED

IGNLSNLQFLDLSYTQIKSVPTSLPNLEFLTMRDTCIKDLPTDICNLSKLQYLDISECKY

LTSLPIEICQLTSLRRLHLSMGMENAVKDPRNVMALKGLTNLIELHFFLFFKFEEYEIMG

DILGSWVEMKHLYLYYNHMNPSKCNLPQGMQNMKKLESLVLFHYRGTNLPDLICEFQQLE

RLVLDSCSTVTELPLLEKLSNLKYLKLQNYSNVRDLRIGKPSGFPKLEMLHLSEMSRVRS

LGMLETGTLPNLKVLKIKKCPRLRLSTGMKNLTSLRALYGHRISWWEKLNWEDSEMKEWL

RTLFKEI

>AB_033943_T.1

MGTPEETSEEIRTPEEGQREIGHILEKEKAFIYVDNALERYPFEQLLPKDMGKAKKLRLL

ITSRDRDVATECRNLKTKFYAMKVLPHTESKILLETQLYGEADNMEERLSSIQLDHIVEM

CGCIPKLLILVAGFIRCEDDPQEAYSRVKEERERNWKGEIGKDIAHYVFAYQNLDETLKD

PFLDICFFFNGKEWSEVADRLGDGELNSLEKRALVTGKTEGIVWVHDVILAMGLQQAEGT

RFSSANKLQEFLDEKEDKEIRKIKGIMLPSKDWSYDEFMRFKDPLYISASYISASKLDLM

CTSLRLFDLRDSAKVKGKCEKILEKLIYFKGH

>AB_034007_T.1

MAYPTPASSSEIEIEVENQPSPAMPHASSASSAAVDPTTRSYDVRCYDVFINHRGPDTKK

TFASPLYHSLLSHGFRPFLDQEELEEGFHFPSQIEHAIRSASVHVAVFSPKYADSEWCLR

K

>AB_034058_T.1

MEASIMCSTQIGSSKGRRLFSASGDKEELVKFEQKLQRMYRHMDSKMSICLYDATECRKV

RPQKPRERSYPDYAVGIEKPLEEVKKLLDWESDQNAVAVILHGFGGMGKTTLADAVFARL

YIEECKYSMVRLFDDVTSEPSTSDILKLQRYILKDLNMGTHEETPADIRTPEEGQREIGR

ILQKEKAFIYVDNVLQPYPFEQLLPRDMDKAKKLRLLITTRNTTMSRQCSKLKTEIYAMK

GLLPIEAKSLLEREMYTSYTNNMEKQLSDSQLDDIVEMCGYIPKLLTVVAGFISCAEDKQ

IAYRRLKEEKEKNWKGEIGDDITHYVFAYQTLDERVKDPFLDICSFFNGEEWSAVANVVG

DSELNSLEKRALVTKKRTGWGSDFVWVHDVILAMGHQQTEGTRFSSAKKIQEFLDAKEDK

VPNLLTLKSDIS

>AB_034094_T.1

MASSSMASSSTSASSCYYEVFINHRGPDVKNTFASHLYRRLLHCGLRAFLDREEMEKGDK

ISPQIEHAIRGASVHIAIFSPRYAQSIWCLNELVLMKNSGATILPIFYKVEPSALRWTVK

GAYAESLRDHQQKTIHDSETGEEKPRYGFDTIQDWRNALNDVADLSGFTLLGEDDDEGEL

LEKVVETVLKKVPKTSLDVAKYPTGLDLKLEKFEETVLSQQQGTEPLEAKVVGIVGVGGV

GKTTLAKEFFNRNRSCYTASSFLSDVREVAGRMSINYLQSKLIKDLKHTDIIIDRHE

>AB_034222_T.1

MAHRDFSPASQDTPIPVSSTSVPASSTSVFINHHDLKVQKTLASHLHRRLRDHGLEVFFK

WLQLAGGDIPLTCEIEGHIREASVHVAVFSPGFAESRWCLNELMLRLESMKNPESGLKII

PVFYGVEPSELRWTKGVYAKALRNFQKKNKSESNTIEKWRKALYEVAEKTGFELRAFNGD

EGKLVDKIVKRVLKMLENHLYYHVFINHRGPDTKRTLASHLYHCLLAHGLRVFLDTEELQ

KGAKIFSQIEGAIATASVHIAIFSPRYAESKWCLDELILMLKSRSTIIPVFYHVKPSEVR

WTRRDCHGPYSQALEKHERRGRSAAIERWRDALSQAASISGFVLEDCNNDEGELVHKVVQ

RVLSRFT

>AB_034289_T.1

MAGVGKTSILKMIYNHYLEVESDAFQHLIWVTVSTVYNLLDLQARIAKEIKLDLGPHGSD

IDTHKMLSAHLKEKKFLLLLDDVWDPLDLQELGVAFGKGKQSKVLFSTRRRDLILGTRSA

DKSIEIQPLSPDEAWKLFRRIAFNDDTVSVSMEIVNRAREIAEECKGLPLAITVIAASMI

GKTRADEWSLSFDLMKTADPDFPKTHPRVDPELYQRLRYSYHALPSLELKNCFLYCAMYP

EDYAIPVDRLVQMWIAEGLVGRGRESHLLDMAIGRSYVDILVDRYLFQNTGYGYSPWSFA

KPQSIRVHDVIRDMAIYIGEKDENCVFKAGQQRRNFPEIHNEYCKRISLYCNIENLPTEF

TSPKLASLLLRCNQIGVVPSGFLVSLTSLKVLDLSTPTEFPPLGSMIPKFPSLGSMIPSS

VGQLKQLEFLKLNGFSDLPEEVWHLSSLQFLDLSGCGLRSLPSQIGELKTLKYLNVMGCV

NLKVIPHEISQLTSLSTLNTDWTGVRLNVEAESEASMWSLKGLVNLAVLTVTVWVDVQEG

MTSSGIKVGIMGTWLEMRRLCLNLEGNVVLPEDMQKMSKLQCFEMSGYNGLSLPDYICNF

QQLEHLKLSRCDHLTGLSPLERLPKLKCLVLLRLFNLKELGIGNSPSGFLMLEELELQNL

NRLESIAGASNKGVWNKLILPNLRVLYIDRCSSLKRFPKGMEKLVSLSRIRGSNLWWERI

EWEWEDVNMRSDLQKKFFVWCEEVV

>AB_034300_T.1

MGKTTLAEAVYSSFSRKDCKCSWIRLCKDTDSCSDIAILQMAMLKDLMGADKQVPDIKTF

EDGRRFIGHILEKEPAFICIDNVLSEKRLQQLLPLPSDLDKAKKLRLLITAHDTNIRNVL

GTKMCTKLYKVKPIPYEHASDLVKGLMHTQKKDPAVIKEIVEKSGGVPLLLEKAAKFIGA

ADDKQKACRIFREESEKLSGQIFGEGDDLLDYNRLPEDLKDPLLDICSFFHGWRWEAVED

IMGISILDRLEKRSLVTREARNKVAVPDVILRIAIQKSKGTRITGANAIQEFLNATGNKE

EGIKGLSFTEDPGVVSISAKKLDRVLSSLRVLALKNLTIGNYNKIFQNLIFLDAGKSPLP

SNVSTFKELRYLSYLPQKQEDLNLSKMPPNIKRLKLNGRMYSYSNVCSVDLHGLQNLKTL

QLLEFKHLAKLPKELGFLKCLEFLNITDCCNFEELPTNLGDLNALKFLTVEKCPKLQNLP

ESISKLTSLNVLELCGCDSLKRLPKGFESLCSLRTLYLNGTGLEVLPISFGSLSSLQELN

LKGTSLQELPSSFEKLSKLKVLNLASCLRLMDLPDGLGKLTSLVELNLIGCGELKRIPKG

NRNRKLTWLFAAMEKSISGHHKKPHAA

>AB_034343_T.1

MSQSEMIPPSLHEPPLGDHDCSSSSSKPTEHVLTQNVKNAATAVDAIDDGENVDKLIQDI

HLLLEFHRKKSDTKPQHRIGFYWDAASDDVKQLKVFLTNAVKCASALGNIISCKDSTQVI

SGLLRNIAHFNLATAALLTVADVLDKFIDVSDNKDGCISLLDDMHKLGILITRLKEDYLL

MDEMGTDIKDATTMKIVEGSLICSKQMDMCMVSRFLLSSVNKKKLQKLGEELKGTRMQID

SQMSYCILKALDCRLMRPLRKRERECPTYKGLEDQLEKVIQLLDWKSDTKAQAVILYGLG

GMGKSTLADAVFARFYRTDCKYSMVRFDEVSSSEDDVVKLQKIIVEDLMIGAEDEVTSYT

QINSIKNFEDGRQAIGRILHKDGVLIYIDNVSNTDLLENLLPKHMENAKKLRLLITTRDK

DIRRGCRIKKENVKFHRMELVSNGVAENFLNQELAGQVNATQLERIVNLCGGIPQLLNKA

VDCLKYSIDNKKEPFKNVEMERLDNIEKYVFSYKNLPRDCRGQFLDICMFFKGSNWENVK

EIVGKAPLRALKKKALVSKDKNKKILRVDGVVLEMGKEMEKITRFRFEFTSTKGQRSTPN

LDLRYQQRRIVKLGSLTEAKGNDGKIFEKILYVEDPKEVTKSKGNGNKPKRRNRRGNFKS

GG

>AB_034356_T.1

MQNPIMTAEYQVFINHRGPDVKMTIASQIYHTLQYQKLRVFLDNEEIRVGHDISSEIKTA

IESASVHVAIFSEGYAESKWCLDELDAILKSKGMIIPVFWDVEPLTLRYIEGGCYKKAFE

EYTRKGRVTKEVLENWRNALKKASHLSGFVFKTKEGNHGKILKQIVDSVLEVFKGGSARV

AKAETFLRNEILKQSEVPKNSDQLEVYKEFITEEFALTHEPDEGEPGLSDLFEADEDGPS

LSELFEADEDGPDLCELSEDGRAVSEPFES

>AB_034383_T.1

MSQVSTVSHCEGELNLLTFLPIHVSFHFLINMGCMIYLNRRFSIAYFTIFRDEDEVKFLD

EVVESVLKKVPKPPLHVAQYPTGLDLKLQELEKILLSEQQQEERVEAKVLGIVGVGGVGK

TTLAKEFYNSHRLRYNASSFLSDVREVAVRMSLEHLQSQLIKDLKHVETRIGRHEGIEIL

KRHLSFCQVLIILDDVDHASQLEAFLPIKDVISPNSLILIISRNKHVLKSSGVLESSIYN

LEGLDTPQSRKLFCSYAFNPPLPLPPPEYTDLVDQFVTACKGLPLSLKLIGAHLCGENEK

SYWQGQLERLYHNHTLPDEVFKTLRISYDSLLNQDQSIFLDIVCFFIGEDIDKGIRIWGI

VGLRNLQNKCLLEVDTQNKIKMHDHIRDMGRNIAEERTMPRRFWDSTTNIIDNLLVQPST

VITEVRGIKMVSSSYEAFWDSEMIWFPTRRSSRTRLSKYIFNNSHIPVMKNLQLLATEDD

KSEDILRRIQPHLKWLRWYRISHSSLPNWIQMKNLKVLEVEGRRLNILWPHQSQGVPLDL

RELNIWAPLSEIPRSIGQLEHLERIAIHNPGGALERLPDEFCRLGSLKYLMFRGFSKMVS

LPDRFGELTNLEHIDLEGASSLQTLPDSFGKLTLLRHLSLRRCKSLTISNGTLGDITTLE

YLNLYSCEHVDELPQQVAHQVSLQELYLDGMNLKELPCVGCNLKVLELGGSLLEMLPPNL

GDLNCLKILSLRSSPQLKYLPDSIGALTQLTISLCGIECLPRSVLKMNNLESLEVWSCHL

WEHPFKKEVEGETEWETAS

>AB_034575_T.1

MPAHYHFIVLSIHPFFPIPMAELTVAPHHNDHPSSSMASCSGSTSYVHYDVVINHCSEDR

DADTFATSLKSCLEFRGLQVFMGKLEFRDEEEAASQTEATISLASVQIPIFSPNYAAWKW

CLDVLVRMTKSEVTILPVFYKVKPSELRWTDKDHKYGTYALWLADHEEKERYDSQTIQNW

RDVLRHASEISGFELEACNGNEEELLSKIVERVFDACPPRDFDVFINHRGPDTKEQYAG

>AB_034576_T.1

MTKSGATILPVFYEVEPSVLDGVYAEALINHEKKRRYNKEHIQKWRGALFDVAKMRPFVL

DGNDENILDLIVGNIMKNVPKRSRVVQSSEVQSVVNCVLKGNDPKSCNVVQSEVEHCFGL

MVKLKEFEKAVLLERQEEHAVAKVVGV

>AB_034662_T.1

MAASTSTAANSTNTQYYYYDVFINQRGPDVKKTFASHLYRRLLSHGFRAFLYQPELQPGE

NLNSQIEGAIRTASVHVAIFSPRYAESEWCLDELLLMFKSGAPIIPVFYEVEPSELRWAR

VNAKLSCTRVKAGVYAQALHELEKKTKYDPQTQKEKPRHDSATIESWRDALSRVADISGL

SLNAFNRDEGELLDKVVENVLKRVKRTQLNVAKYPTGLDEKVQDFETTVLLEQQRG

>AB_034682_T.1

MAMRRLCCCIPFFQGREDSAGISGIAEAGAPQCVKESAAENVDGAPQCVKESVAENVDGA

LQCDEASSSSSGVIKKILVEGEKVKVDLEHLIEHIDMLLRLRAQADKKILPHKECWTWPD

GSDWKSLNKMLSSGIDFLSDLKDYISGPVKAILKLIGNASLATAGLLVVAYTLERFDYIS

TNNEECFQVLKEMSNVANLVKILKENPKEMKNEIEDATTMIVKGAILCCGQMNSSPWKKF

LKASENKEDLSYITEELILLYTKIGAHGGSSAANDIKGVSNDIKRVLTAIERESKSPLSR

EYPTHAVGIEKSIEEVLDRLECKSEMNAVTVILHGFGGMGKTTLADAVFSKIEGYKYSKV

QLCERIGSAPDIKQLQKTIIEHLGPEEPTPNIITYQDGQAELGKLLKKVQAFIYIDNVLD

GDELKKLLPPDMDTANKVRFLFTARDENVKYSCPRSRTEPKIYHMKSIEPLEAKRILEKK

IHSGISESQFNDIIKICGGIPLMLRLAAPFIALSPDKEDACRKLKQQQGNLEHEQFKEMH

CA

>AB_034836_T.1

MDASTSTAGDANNNYHYDVFVNHRGPDVKKTFASHLYRRLIPLGLRVFLDYEELQVGEKL

TPQIEKAIETASVHVAIFSPDYAKSSWCLNELLHMLKSGAPIVPVFHHVEPADLRWTQAQ

GRGYAQALCDLEKKTTKDGQQRYSSTIIEEWRNALLSVADTSGLELKAFNEDEGLLLDKV

IECVLRKVKKTQLNVAKHPTGLDEKAQHFEEIVLSKQHSGRVRVVGIAGMGGVGKTTLAK

>AB_034844_T.1

MTGKKSPKTESGKGYWQIPSDGSLRKIREILSDGINVFKDIVPGEAKTAIQGLFKGMGMA

HFAGTGLLVVALVLERFDTISTNKEECLCVLDEICQLTHHVNDIKRRPRLREAMDASIKE

AINLIVEASIMCSTQIGSSKGRRFFSASRDREELVQFQQKLQRMYRHMDNKMAICLYDAT

DCRQVRPQKPRERPYPEYAVGIEKPVKELMELLEWESEKNAVAVIVHGFGGIGKTTVADA

VFARLHVEGCKYSMVRLCDDVTSKPSTSDIVELQTYILEDLKMGTPEETTSEEIRKKIRT

PEEGQREIGRILEKEKAFIYVDNALERYSFEQLLPKDISKAKKLRLLITTRDRDVGRECS

KLKPKIYPMKVLPHIEAKILLEKELYGETDNMEKPLSSSQLDQMVEMCGCIPKLLIVVAG

FIRCEDDQQEAYGRVKEERERNWKGEIGKDIAHYVFAYQNLDETLKDPFLDICFFFNGWE

WSEVADRLGDGKLKSLEKRALVTEKTEGTWRPTVVWVHDVILAMGLQQAEGTRFSSANKL

QEFLDDREDKEIRKIKGIMLPSKERFMRFKDPLYMSASKLDLMCTSLRLFDLQNSAKVKG

KREKIFEKLLYFKGQIFDLPFDVSRSKELKYLDFSHSPDQEFSNYEELLGSISKLPYLRV

LKLSCCKNLGQLPKDFGSLNSLQKLHLVHCESLEALPSSLGNLSSLRLLDLSWCRSLKDL

PCELGKLTSLAQLRLSDCSELSSIPDSIGQLKSLDSSMDLSGTGLKELPDDFCNLSF

>AB_034902_T.1

MSDPQTSALSASSYTNCSEYYHDVFISHRGPDVKNTFASHLYYRLRSHGLQPFLDREELQ

AGELLSPQIEGAIRTASVHITIFSPGYADSRWCLDELLFILNKSGDAIIPVFYNVKPSEL

RWTGRYLEALANHEQNGRYDRQTLENWRKALSDVSYRVGFELQAYNGDEGKLLEEVVQCV

LKRGIKIGLNVARYPTGLEENVEDLEFTVSWKQKGKVKVLGSVSMGELCKTTR

>AB_034905_T.1

MADLQLQTPAPDHTSFPSSSTSASTSYANNNYYVYDIFINHRGPDCKNTFATYLYNRLCK

HGLRVFLDKQELQEGDSLTSQIERAIRTASVHIAIFSPRYVESSWCLNELVLMLESGKTI

IPVFYGVRPSELRWTEGENGLCGTLFRILCCIGDQTGVYARDLRKLQGKKTFDSQTGQQK

PRYDSSIIGRWRKALSDVAGISGLDMETYNSDEHQLLEQVVEAVLKIVKRKLHVSKYPVS

LDQKVEDFERKMLLQQQSGQTGVIGIV

>AB_034929_T.1

MGANDYSFKMQPLSEKDGWELFRRKAFTNGAAPEKDIETIAEEIAKECKGLPLALNVVAA

ALRSERDVSKWRDALAFMTNVDPSFPETHPTIDAELYQRLKWSYNDLPDYLKSCFLYCAA

FPEDTEIDVETLLEMWSAEGLVKRRGITSLIDVGHQYIHFLSSRCLIEYVGFKEMSIHTL

QGVEREVIKVHDVLRDMAIYIGERDEQWVFASAQHLQNFPVEEVTRNCKRLLLGHNDIQH

LPTDLECTGLVSLVLANNRKLTEVPASSLLNIMSLKVLDLSCCPLITSLPTSVGQLGELE

FLNLEGCYRLKELPESICTLSRLQFLNLNYCESLLSLPDKIGELKTLKHFSFEKGGEVTH

TVIPRGLFGLTSLTKLCLRLDIEIRMEDLRNLSNLMELCIPMTADTTGNCDCTWSEMRKL

TLVYDDYGPYHDHVDVGEDAEMSVNILPSNMETMIKLQSLILVGYPEESLPNYISKFQNL

ETLELHRCGQLRELPLLEIGSDSARDSFPMLKRLVLKSLDKLESITGVLTMSKLQYVEIM

WCPLLKRLPMGMEKLYNLQKIKGEEEWWSNLIWEAEVTRVKL

>AB_035084_T.1

MEPVGSFIAGQLLAVVKDVANKNIDFKDCCQNLQKTLEGLIPTLEEVVYNQLPDDGQKML

KEFYEKLQTGKTLLQKCSNVSRVNIFRIYKYSRRIDRLDKDISKFIQTTGWALTYREIYE

IRYGRANLGAKVDTCELKIEEIGDKIENLPESFQRIMK

>AB_035127_T.1

MKSVDPSFPSTHRTINAELYQRLRLSYDDLPNNLKMCFLYCAAFPEDAEIVVETMVEMWT

AESLVPQRGKTYFMDVGRECIDTLVDRCLVEYVNGETEKITIHDVLRDMAVHIGQGEEKW

LFEASQHLKKFPSEKETRDRERISVVDNEISDLPADLKCPTLVSLALAKNKRLKVIPERF

LVNVMSLKVLDLSNTSIQVLPTSVAQLVQLEFLNLRGCYRLKDLPDSICNLSHLQFLNLE

RCVKLNSLPDTIGQLKNLKHLKFSGGEAQRIPRQISEVTSLNKLVLPKSSTPVNVEDLTN

LSNLIELDARVKPKTKGGSMNAWSEMKKLILHFEWVEDENRDDAALDILPRSMQKLQTLH

LYDFQGVILPNCICQFQNLKNLCLTSCRVLKGLPALETGSDAASGGFPLLERLELYSIDS

LESIVWNQGTMLQLQILQIENCGSLQSIVWNEGKMSKLQVLHISKCRVLKTFRMEEIPNL

RDVSVSHCDKLELFTVSGGFPMLEALTLHHLPSLQRVAGPSGFPMLEALTLHHLPNLESI

AGPFMWSEETMPKLQSLYIKNCLLFRRLSIENFPNLKQITIIHCPELEIEIGNLPMLERL

ILANLEKLESIARSSSVWNEKTMSKLQILRIINCPLLRKLPMEMDKLCNLREIFGKLGWW

EGLIWEDENVKTKLSQLFINSKKK

>AB_035219_T.1

MDSFNCKNACSRLDTVLTGLIKEFQQLSLSNLRKPKNEQNPSYEKFLKTLMKGVELLKKW

EGTSCFKFFHSLRYGFQIRQLEGEIADFLRYQMPVNIFLEVKNLIAELTNLRQQYELGSM

DESKMNETILKHVSKLTNDPQQNAMILQQMGADNMFDGALVEVPCNYVGLGMSDFAVGLE

KNIWNLKRTLLQSEVTVVGVHGMGGLGKTTLALALSNDKDIKDVFQNNIIFITVSESPNL

KVILETMWEKIVRRKRPEFQNVEEAHRELQQQLLRQAKQTLVILDDVWSRANLEKLLFEG

VGYKTLVTTRDRSTIPKMTSTQLYELPLLDDGDALSLFCFWAFGQKSIPSTANEHLVKQV

QAQCKGLPLALKVIGS

>AB_035322_T.1

MPPMSIRDCLERTRDAIGEAKSLIDRSQLQQRCLDRVFCKPRISRQIREWNERIDQLRQE

LENLFSTFANVQQIASSSAQVPAKELLQPLPEWGIAGVDIKSAEKQLQTLINAPQFRIIG

VYGMAGVGKTTLLRSVYNSYKVSDVFEVVIWVTVSQGYKIRELQSRIAEVLNLDHSRISD

LDTDTLKMMLSASLVKRKFLLVLDDMWSALDLQQELGVQFGDDKASKVVFSTRSRDVALT

EANVSMKVEPLSRHEGWELFQRVAFKERHVPEELKECAREIADECEGLPLAITVVGAAMR

GKGAVADEWKSCLNLMKNTDPSFPETHPRVDQQLYRRLRWSYNDLSTTPNLQNCFLYCAL

YPEDARIDVERLVVMWIAEGFVKSKEETYSTDMQLGYRYAKILIDRGLFQNVNFEKQSKP

DILVNKPVNAWQERYITVHDVVRDMAIYIGEREENYVCRAGQQLQDFPPSRSRADCKRIS

LCGNQIKCLRKDFLSQNVRPRLVSLLLSENPLGYMLVQEDFFMNLTSLRILDLSSTHIEP

VPKSLTQLALLEFLDLSRTNINELPEDIGKLTQLQYLNLSSCDKLVSVPSSIGKLTQLQY

MNLSYCDKLGSVPSSIGKLTQLHCMYLWCRNLVVPPEICQVTSLRKLSLQIPFRIAMGPL

RMGTWSEMTHLRLGLNKNMNI

>AB_035471_T.1

MDSGSHSSNQLQPSKPSAPSLRTIFPFNLHIIPVLLSAIGSMAADIGTGVATNVTNAIIG

LLFQQLKDVMELEENLQLLNTEFARMNEWLHHIQTQFERQQKKLPEVVERCLERMKDALE

DATALIHRVNRQRGRCLGCCLLCFPKIPAEIRVWKTRFGELFQQLESALSIAASTQQIVE

SAQPQAEVLLQPLPDSGFVGSGIESAHKQLLTWLNEPHPQGRVIGVSGMAGVGKTLLLQL

IYNKFCEKKVSSSFDFVIWFTVSQNYKIESLQDTIAESVGLNFKVAASIDQKKMKLYAFL

ENKRFLLILDDIWSPIDLNQVGVNFGHNSSSRVLISSRHRDVVETMAANEYCLMIRPLSS

DEGWELFRRRAFTNG

>AB_035735_T.1

MAEAAVVSTLMQTLTSMLADKLLQEVSLITSFKKDLEFICEELVSIKLLLNDAGKRNTRS

MSNWLDKLEYFLYDALDIVKECGAVRKFGNPIFRYRMACKIRGLKERISKIHRSAKYLKH

LTCVLDLNAFNEENSEDKRERSSAFLKEIQHVGIDNGIKEITDLIVSEDDRHQVLAVLGM

GGMGKTFLVQNVFISQRVEQCFDYKVWLALSQSFMVKQVLVEVCRQIKLSVDEIQDLRAE

DLTTKIHEHLKEACCLFVLDDVWERDVWNKIGLPLQSKYKIVVTSRDKRSVEGVRRYNHD

IYNMEKLSDLDSMKLFCIHAFPDREENSPPEQLKSFVPKIVEKCSGLPLAVKTIGASMTR

VRRIPNDWESTLNRLNEAEAMSRGVMSSLRLSYENLPYRLKLCFLYCSAFPKNTRIKSEY

LVHAWIDEGLSPETAEEAYDSARSCIDDLIDRCLIEVSKVGG

>AB_035760_T.1

MFLEEWERPPWDAVSSRVGGCKYSHVQLFEDVNAKPDIKDLQKKILKDLMGTEEPVPEIR

KYQDGQQALELVLQRVEAFIYIDNVLEGNALKNLLPDMGTAKKVRLLLTARDENVTKYCK

RLHTNPKIYRMKGIPPPDAMNILKR

>AB_035791_T.1

MSMEPVSESTIGTAVGEFFIITRIVMNIIISSKSSSNELRQKLLSLKPTIDQISKISSDA

DSSSYKGQPIRDIQAQVQDGLHLVKKLEKVSSFNIYRKYRYGKQILKVEKKINDFLLTQS

LAGLVLDVQKLNAESEGFKERLERIEEMGQQIIDSVNAKMTNDATSNSIMLHQMSTRELF

GTSIDESNDTVMAEQSTSSSSQVPDMPNFVVGLNNLVNDVKQILIYQKGVNIVGIKGMGG

SGKTTLALAVCSDSQVKDFFHNNIIFITVAQSPNVKGLLEAMWDKIIGGRRPVFQSIEDG

RNQLQKKLSAKGYQPTLVVLDDVWSKSNIEPLLFEAEGYKTIITTRQDYTIPISDSTRVY

NIPMLQQADALSLFCFWAFGRASIPRTEDEDLVKMVEAECKGLPLALKVIGSSLRGEPQP

VWENANKKLRRAESISEYHRDNLLHCLETSVDVLDEESKQCFLDLGAFPKGRKFSVDSLL

DIWVHVRGMEWEDAFVVLLELASRNLLNITSDPGSRAISFGCASELSFSQHDVMRDLALR

LANHDNTIHCKRLFMPSKEDSIPTKWLTLKDQTSQAQFVSIHTGQMEEQNWCQINFPEVA

ALALFFAASQYCLPTFLHTMPKLKVVIIYNYGSKRATLHRLPNFPTFTHLKSVLVERLIV

SSPFEYCRFSGNLEKLSVCLCEGLGNMTLFDKEQVPKFPMFREIIFDHCSDLEELPGKIC

NLTHLQKLSVTNCHLVQKLPDDLGRLRLT

>AB_036214_T.1

MVKKPALYVAKYPTGLDAKVKDFENSVLLQQKLKPQILGIVGLGGVGKTTLAKELYNSKI

SEYDRSCFLFDVRSNPLNSSQRKLVKSLTGADNPVSSVDEGIEMLRRHLSSTKSLVIIDD

VDHVDQLDALLPHQTDLQQSNSLVLITSRNKDVLTRRVVGTSIYKLTGLNTRHSLELFCS

HAFLQPYPLPGFESLVDKYLESCKGLPLSLKVFGALLYGEDISHFEEQLERLEQRLPTDI

YEELKISYDSLSEQEKEIFLDIACFLRGENKNMAIRIWDGSGWNGRLGFANLENKSLVEV

DSLNKIHMHDHLRDMGRQIAERYVLWGDKLPRRLWRWRENTIDDLLLPSPEGHAIPVRGI

RMDLREYNGFVDDDAFGGIRMKG

>AB_037218_T.1

MTELGVEVSAGVINEVIVAPLIRQIDDVIHLDRNCGVLQAQLKVMKLHLQTISEVFENQQ

RTVPRAVQDRLECMRDSIEEANSLIGRSQQQQQCLDYIFRAWDCLFSKPGISAQIREWTI

GMDKLFEELETDISIFVRAQRVDSLSPQEGNALLQPVPEGVFVGSGIISAATELQRLLTE

APQVRVISVYGMPGVGKTSLLQKVYNSHKVSKAFFTDVIWVTVSQDIVISDLQAQIAQEI

HLVNFSSILGDSQKMKLSASLKNKKFLLILDDLWSLLDLEQELGVGFGVDKGSKVVFSTR

FRNLTLMREQDSIHVEPLLPEEGRALFERLAFEGGHVPEELKDCVRETADECKGLPLAIT

VMAAAMRGKTVDELNVSLSLMKNADPFFPNIYPCVDKDLYQKLRWSYEELRQTPNLQHCF

LYCAMYPEE

>AB_037383_T.1

MYKKIAIEMTLRLINNIACYQERHLSRIYPENAVGVEKSIEQVTDLLGWGSKENARAVIL

HGFGGMGKTLLADAVFSLLDIPGCKYSKVKLFKSIESTPDIVDLQKCMLKQLKGSENIPD

IGGYEDGQRELGAMLKEVPALIYIDNVLEVEALRNLLPPDMNKAKKVRLLITARDSNVRK

ACPLKRVPELYHMRGMASTDAVSLLQKEMYDDPRGILDSEQINIIIKKCDGIPLLLKLIA

ASLRVAKDKPQEVRGVMEQLENLKG

>AB_037536_T.1

MRQESYLWRGYPTRAVGIDKSITEVLDMLKCESEEKAVTVILHGFGGMGKTTLADAVFSR

VGGCKYSHVQLFKYVNATPDIEDLQKKILKDLMGTEEPVPEIRKYQDGQQALELVLQRVE

AFIYIDNVLEGNALKDLLPEDMGTAKKVRLLLTARDENVTNYCPRPHTNPEIYRMKGIPP

PDAMEILKKEIKEEINENQFKDIIEMCGEIPLMLKLAAAIIGCADNKQEAYSTLMLDRGK

WE

>AB_037634_T.1

MNDLDKLWFKGEGCKTLITTRNNDIVTSDRRYEKPLLELSDAVPLFCHWAFGQRSIPGTA

DNNLVREVILKCCGLPLALQVIGRSLCEQPHGVWVTAKDGLSQGHPVSDHHEDALLRYLA

TSIEILKKEVRECFL

>AB_038661_T.1

MLHGFGGLGKTTLADAVFSLLDIQECKYSTVQLFKHIDSKPDITELQRLILRDLAEPGET

IPEIRKPEDGQRELRRMLEKVPTFIYIDNVLGQDELEQLLPRDINKAKKLRLLLTARDQD

TVRRALKVKPVVYAMKGLPHTEALRLLEREIYGETDDKEERINSTQLNKIVELYGGIPKL

LEVVGAFIRAAEDKQNAYEILMKEKDKWD

>AB_038906_T.1

MNLESSLDAEMLKVKVNIFTSLKKKMKFLKKMKFLLILDDIWSSVDLEELGIPAGNEKDS

KVIISTRSRDVILRTRANDYSKQIQPLSEEDGWEFCRKAFKDGGAPTKSIEHVARLIVGE

CKGLPLAINVIAAAVMGNATVHEWNLALFPVKNPVFLSYSDSGSGNDEGLYQGLKWSYNH

LLDSNFPICFLYCCAFWEDAEIYVEISIEMWIAERLVHSREANYLMDIRHRYVELLVERC

LFEDIYGNMR

>AB_038949_T.1

MDVSNCSIVSALLSSLIHRVHLHYSPDIETPQNEQNPIYEHCLSMLNKGAQLVKRCEKTS

RFNSFQNLRYASEIHQLEKEIRDSVQYQMPDQLSPDFMNLISEFKNLRHLYELGTLDERK

VNETIVPKLTNDPQLNTRMLQQMGSDGMFDGAFDDTPTCNYNVSGKSDFVVGLEKNIFDL

KRILLQREVSVVGVQGMGGVGKTTTAFALCNDQEIKGAFRNNIVFITVSQSPNLKEILET

MWEKIIRKKKPEFQNVEDAHRQLQQQLLRQSKRTLVVLDDVWSRANLENLLFEGEGYKTL

VTARDRSIIPTTTSTRLYKLPLLEEADALPLFCFWAFGQKSIPSDVDQQLVKQGGHRGHR

FKSCRGFWAAHIGPS

>AB_038960_T.1

MPAHYHFIVLSIHPFFPIPMAELTVAPHHNDHPSSSMASCSGSTSYVHYDVVINHCSEDR

DADTFATSLKSCLEFRGLQVFMGKLEFRDEEEAAYQTEATISLASVQIPIFSPNYAAWKW

CLDVLVRMTKSEVTILPVFYKVKPSELRWTDKDHKYGTYALWLADHEEKE

>AB_039223_T.1

MATSSSNDQWYDLFISHRGPDTKETFARSLYLRLLEKGFKAFLDKDEMQPGYDISSQLNN

AIQTARVHVAILSPGYADSSWCLDELIMMLESKDPII

>AB_039313_T.1

MESLIGPAFTALNAILGAPVTQLFKDVINKEENLQILNTDFKRMVDLLLDVGTLFQHKEP

PQIVNHWFQELLKSLTEANKILDHPSGLRRCLGCLICKPARRASQIREWTAKFDKLFQGL

QNDFSTIVNGQKIAKSAPQSEALLQAVPESGYVGSGIKSGEMQLQAWLDNEDHHFRIIGV

YGMGGIGKTSLLKTVYNAVKKKGKLFEAVIWTVVSQNSISDMQTDIAKQINLNLGSSSTV

GMELSACLRGKKFLLILDDIWEHFNLAEDLGIPLGNDKGSRVVISTRSRDLVRRMGADDY

SIQIQPLSTDEACLLFCRGAFKAGTTPTKDIEDVATQIAGECNGLPLALNVVAAAMKGNT

SLHDWHLAFNQM

>AB_039412_T.1

MEMAASASASTSTAADSNNSYSYDVFINHRGPDVKKTFASHLYRRLILHGLRVFLDYEEL

RVGEDLSCQIHGAIRTASVHVAIFSPTYAESEWCMKELVLMLESKAPLIPVFHGVKPADV

RRTRGEGVYAQALHILEQKRTHDSQPRHNPSTIENWRYAFSRVAGISGLDLEAFNGDE

>AB_039486_T.1

MIDKLVEHVVKMAKKTLYVAKYPTGLDEKVEDFEAKVLRTSKVQMVGIVGLGGVGKTTLA

KEFFNRKRSEYNKSYFLSDVRENAKSSLHTLQIKLLKGLNLLNGAIIDDIHQGTEMLRKS

LSSSQVFLILDDIDHEDQVDALLPERDDLSNDSLILITSRDKDVLARSGVEESSIYELKG

>AB_039736_T.1

MNQSKSETSRIVRITGIGGAGKSTLVKYIFNLRNSDFSKSCYLSTNREEVDIRCLQRQLL

HDLLGIDEFIGNIFEGKRILRDHLAGLQILLVLDGFDHIEQIDSLVDVYALGAGSLILIT

ARDRESSPKTLSYDVKPLNRRHAKELFCRHAFLQFKPLEGYEDLVEKFLEICGGLPLSLE

VVGGLLAGNLDKTYWMLQLKMFSKRLPDQILDTLKASYQKLNKEEKEMYLDIGCFLAGED

KELAVRVLQGSGYTDVWGTLESLRRKCLLDFESDVESSNDINYKYEHPNIGRLIMHNHLT

GFARHIAREEFIEFSEHKPLRLSCSNDMNQILQLQDGSDFCRIRGIRIPKSQNPPQPMNH

RSIR

>AB_039893_T.1

MAASASASTSYSTSTAAHSTNNYCYDVFINHRGPDVKKTFASHLYRRLTLHGFTAFLDQE

ELQVGRNFDPQIKDAIRTASVHVAVFSPTYAQSEWCLKELVLMMQSKAPIIPVFHKVKPE

EVRHTRGEGKYAQALQSLERKRNHYSQPRHDSSTIENWRNALFHVAGMSGLELEAFNHDE

GVLVDKVVESVQKLRTPQLYVAQHPIGLVEKVEDLENTVLSQQQNGR

>AB_039897_T.1

MKLHAFLESKRFILILDDLWSPIGLNQVGVKFGHDNRSRVLISSRSREVVEIMVANAGYC

MQIQPLSRAEGWELFRRRAFTNGAAGDKNVEGIAEKVALECKGLPLAINTVAAALARKKT

ADEWNRALELMKNADPSFPSTHNTI

>AB_040170_T.1

MAASSSTVSTSNNNYHYEVFINHRGPDVKKTLASHLYRRLLSHGLRIFLDQAELKRGDDF

TSQIDDAIQSASVHVAIFSPTYADSSWCLDVLLKMLESKATIIPVFYKVKPSELRWTRAR

TEEATGEATGEGTSNKKRSYAEALAELEKKTVEKGPRYDPATVEKWRDALSRVAGISGFE

LDACNGDEGELVDKVVER

>AB_040188_T.1

METVFIGAASNTISSGVTVAIAPLIQLIEDAIHLDRNTRLLQDKLSSLKALLQGITNQFQ

GDQRSPPEIVNDGLQRLQRSLEEARGLIDRYQQQNQCLNCLFTCKPRPRLSRQVREWNTS

LDQLLSSLERDFSIFSNTQQIAVLGPQRAEALLQTMPKLGFVGLGIQAAEMKVQTWLNME

DHQFRVIGIYGMGGIGKTSLLKRVYNTCKQGTVFEAVIWLTVSQNFNVMSMQSVIARTIN

IDLGSSSNAESSSDAEMRKMQLFTNLKEKKFLLVLDDMWSPLDLEEQVGIPVGTEKGSRV

IISTRSTDVIQNMEANEYSIQIQPLSEEEGWELFCRKAFKDD

>AB_040348_T.1

MFLRTCDGLPLSLKVFGALLFGEKDKSHWKQIHDRVHEVPNDILDRLRISYDALSLEDQK

IFLDIACFCIGKNKNSAIRLWEGSLGFRNLQGKCLVEVDSENVIHMHDHLRDMGRQIAKD

ESPRRLWHSIENIDDWLQQQSSVISEARGISMDGNNEVANVLIRSPMLSWFQRKIDKVFG

NRMKKRPGISA

>AB_040408_T.1

MADSEIEIITVPPHHALASTSDSIAHSPGDFDVFINHRGPDVKNTFASHLYRRLVQRGLR

VFLDKPELQEGLNFTSQIQSAIRSASVHVAIFSPRYAQSKWCLDELLQMFESGAAIIPVF

YHVKPGQLRWTLGDGGVYSKDLRKLEEKKTYDTETHEEKPRHDPSTIIKWREALSRVAEI

SGFELDASYTDEGELLDKVVECVVKKERETRLNTEEAQSSENTILLEEQERKGQLEVRLQ

WSSAQFRVLWWPILRILLPLIGVVVLVFWVKVFVDYYLVNDVMKQQFDQFDKFVHGFDSR

GVPQFSNMDQFGPFEDQFGPFEDQFGPTISFPSNVQIDTEIGSPISFPSNVDMDDQLKKQ

IDKQFGGETITTFVGEP

>AB_040756_T.1

MAASSSTAAASSSTAAHSNNTYCHEVFINHRGPDVKETFARDLYTCLNFSHGLRVFLDQP

ELQAGEKITSQIEDAIRTASVHVAIFSPGYADSNWCLDELVLMLESGATIIPVFYNVKPS

ELRWNRRAGGLYAKSLHNLA

>AB_041421_T.1

MANTLRSLFGRSSRTHSSTAVQNELAPNIPAQSEWSKELSTDCAVGIEQPIQEVIKLLDW

ESPGNAQAVLLHGLGGTGKTTLAHAVIERVQKRVSSKECKYSKVTLFSDITSNPDIIGLQ

RCILKDLMEPDAVPDIRTWEDGRKIIGTILEKVVAFIFIDNVLSEDPLERLLPWDMEKAK

KLRLLMTARDVGVRRGCRLTTVHCYLMQLLTEAEAVTLLKKEIYGGEIDGQEGQINSRLN

EIADMCGGIPLLLSRAGRFIRYGEDQEKGILQVLQEREHFDGEVFSKVEPDIFHYDSLPY

RYQDPFLDICSFFNGWNWDTVACIVGEYELEMLERRALVSKN

>AB_041439_T.1

MDLLLTALTELGGVVQVIYSSKTWGKELRDKLMLLKRDIDDAIEVIADSDSSTHRYSQFR

DFQAVLQDGLSLVNEGDNLHVCDVYRKWRYGRRVREFQEKIKDLIDVQGSPIIARDLQRL

GTEVKKLHQQLEAATIAEQPINSTSQDVPDMPKFVVGLETCINAVKQILLQDDVDRLGIA

GMGGSGKTTL

>AB_041666_T.1

MASSSEIEIEVENQPSPATPHASSASSSAAADPTTGSYDVFINHRGPDTKKTFASHLYHR

LLSHGFRPFLDQEELQEGIDFPSQIEHAIRSASVQVAVFSPKYAESEWCLRELLIML

>AB_041794_T.1

MEKGDFLTSQIAAAIRTASVHIAIFSPRYADSEWCLNELLLMLESMESGRSTVIPVFCCV

KPADLRWTLGGNGVYAKSLTSLQKKKTTDSQRHRKKPRYQKDTIEEWRNALSKVSHINGF

DLEEAYNGVEWKLVEDVVKLVLQKVEKPPLDVSKYPTGLDEKVKAFEELLQQQSGGPRVM

GIVGLGGVGKTTLATEIFNRLRSNYNQSC

>AB_041797_T.1

MATSSSASTSTSQGNTNTNIYNVFINHRGPDVKKGLAALLYQGLREKGLGVFFDCEEMQA

GEKLSTQIEGAIRQASVHLAIFSKGYASSSWCLDELVLMQETNRTIIPIFYGVNPSALRR

TEAADGVYTRALRKLEERHDSNTINKWKHALFNAAGISGYELSAYNNNEE

>AB_041809_T.1

MAGVGKSTLAKRFYNLKRLDFSRCCFLSEVRKSKGELPSLQQKLFHDLVGYCDMHLDDTS

QGRSIFRDRLRGLRVLVVLDDVDQIEQINSLLDIDAAGPGSLILITFRDKHLLRCSSRKT

KTVLYEVKPLQGKHARELFCQHAFLQPNPQEGFEDLVIEFLKICSGLPLSLNILD

>AB_041915_T.1

MAASTSTAAPPNASASAKAPTAASPNTNYNFHYDVFLNHSGSDVKYNFASHLYRLLRDRR

FRVFWDRQETQAGNTVNSQIQGAIATASVHIAIFSPTYAESESCLNELLLMLKSKATVIP

VFYHVKPSDLQIRTSKDGVYAQVLRNLGRTDKTGVYAKALQIHEKKKRCDSQTLAKWRTA

LFDVSQLGGLELDAYNGDEGELVDRVVKRVEEEVPKQQFSVGKHPTGLDEKLQDFEKKVL

LQQQQHRGEVKLVGIVGSGGVGKTTLAREFFNRKRSGYRRSCFLSDVGEAAGRSSLHSLQ

NKFIKELTSLDPEIVSVAEGKGKFAGYLLSNQALVILDDVQHVDPTDAMYLPLKDVLPSG

SLIVVTSRDKELLRKSGMPESSIYTMTGLDREHSRELFCLHAFHNRLPVVGFETSVTKFL

DVCNGLPLSLKVLGALVYQKDLKYWKAPLRKISQLVPTDVQSILQISYDGLDDQEKEVFL

DIACFLINEDRDMVTSIWDAPGWVGWLNLQNLVNKSLVEIDSKNCIRMNDHLRDLGKDLA

>AB_042391_T.1

MEPVSSTYQVFINHRGPDVKKTLASLIYHRLTTCHGLRVFLDNKELNTGDSLSPAILGAI

QSASVHIAIFTEHYAESSWCLDELCSILRSFHERHTSIIPVFYDVQPSDLRHIESGPYAA

VFEVHRRRGRDIEKWKTALKEASQISGLLFKTKESDYGEHLERIVKEVLKEVFKREPLDV

TTHPIVGLEQAVEDFQNEIKKQSDLNETILVGITGMAGVGKSTLAKRFYNLKRLDFSRCC

FLSEVRKSKGELPSLQQKLFHDLVGYCDMHLDDTSQGRSIFRDRLRGLR

>AB_042439_T.1

MAASSSASTFTTGNPNISFCYDVFLNHRGVDVKKTFARSLYLRLVKEGLKAFLDQEELQP

GYNFPSQIKQVIGTARVHVAILSPGYAESVWCLRELVMMLHSKAPIIPVFYGVTPAEVRW

TEGKDGKYGRALQNLAEKKTQGELRYDSKDIENWRAALSHVADIKGFELEVSNG

>AB_042588_T.1

MENKSCCCRLFSCFACFSDENTTPSGAQPEPSEIAPVDAFHVTSTAAPNATGDALTHPHH

GPQPIASSTSSEARTSTDVLIESNDSQPIVQPDLSCSSQPNTSPLALPAHYQEGSSSSND

VTNHHNVADYQLQGTSSSSSVSKASDLTKNTTASGAQPDPPEIAPVDPNSYVTSTAASNA

THDALTHPHHGPQPIVPPFASTSPEARTSTYVPTELNDPQPIVQPDLSSSSQPNTSPLAF

PAHDKDSQEGSSSSNAATNIQNVADNQLQGTPSSSRVSKVSDLINNILTTAKAVDTENVD

ADLDGLIEYISHLLELIKEKEKTGKISSNTEGAKDYWQGHSGSLGKFREILSEGINVFKD

TIPGEAKTAIQGLLKGMGTAQLASTGLLVVALVLERFETVSANRDECLTVLGEICKLAEH

AKKLQQRPGLRERMEASIKEATGLIVKASIMCSTQIGSSKGWRFLSASRNNEKLLSFQQK

LERMYRDMDSAMLVCVYDATACRQVRPQKPRERPYPDYAVGIEKPVEEIIELLDWESDKN

AVVVILHGFGGMGKTTVADAVFARLYIEGCNYSMVRLFDDVTSTPRTSDIVKLQQYMLKD

LKMGTLEETEVSDIRTLEEGQREIGRILQREKAFIYVDNVLGRYAFEQLLPKDMDKEKKL

RLLITTRDTDVSREC

>AB_042600_T.1

MEIQQHRLEFNKKKDTKASAVNVWLKQLEALLQQASKMVQRCTTPSSWDVVSRYQTRRMI

TRLISDTNEVLKLSPLIHMAQTQEVLADALGKMREARLDSASATTSQAGNSPTGYTARTK

LIDEPLIVGQDRANENLQKLVLEDADAKNIGVVGKGGSGKTLLLKALFNSQEVRNHFNGG

LLLWITVSQSPSVTTLRNELYTQISLQKNVDLSTNMKEEDVKIWLNETLQQSKGFALFID

DVW

>AB_042838_T.1

MELVATPSCEPYQIFINHRGKDTKLTLASLIYHALEPYGLRVFLDQYVLSPGDIIETEIR

RAISSSSVHIAIFSKNYAESRWCLKELCWMLKSSHEPRFIPIFCDVEPWELSFVEKGCYA

EAFEKHRKRESMEVVDEWKKALGKAANISGKVFKGKEAL

>AB_042900_T.1

MANSPLASSFPSHTSHSPTDNEAESSRPSTSAGAHPSSNYYYDVFINHRGPDVKKTFASS

LYRRLTSEGLSAFLDQRELHEGESITSQIECAIRTASVHVAIFSPRYAESKWCLDELVLM

RESGSVIIPVFYRVEPSELRWTRGGNGSYAKALRDLETKKTDEEKLRHDSTTIENWRNAL

AHVAGISGFQLEAYNGDEVELLDNVVQGVLRKVNKTSFKVAKYPTGVDE

>AB_043304_T.1

MEQGHNITPQIQWAIRNASVHVAIFSPRYAQSSWCLDELLMMLESGSTIIPVFYDVNPSE

LRWTRGGDGVYARQLQELERKTTTDSQTNERKKRYDSNTIENWRKALSDVSDISGFELKA

CNGNEGLLLDKVVRQVLNKVPKPPLYVAQYPTGLEDKVKEFETTVSQQQSGKTGGVGVVG

YGGVGKSTLVKEFFNR

>AB_043474_T.1

MAQNIGTGTAGQVAADIINKGFVAPIVQQIDDLIHLDRNHQRLQSNLKRMENVLQHISNG

FEQQKKTPADSITYCMERARHAIVEAERLIGESGGEQSSILSKPKISREVRQLNMKFDQL

FPELLRDFSLFLAVQQIVVVTPPQSSGSPQSLPIGQSSASALPQEKNVFLQTVPDGGFVG

SGIESAGTQLQSWITADQAPQVRMIGVYGMAGVGKTSLLKRVYNLYKVSDVFDVVIWLTV

SRDYKIYDLQGQIAEAIDLDLLSTSNIDTRKMKLSASLKLKKFLLILDDIWSSINLDEEL

GVAFGDDNVSKVVFSTRNRELASTDAHKSLKMKPLSTEEGWELFQRVAFVDGHVPEEIME

CARKIADECAGLPLAITVVAAAMRGKTPVADEWNTSLSMMKIADPCFPDTHPRVDKQLYQ

KLRWSYDDLAQTPNLQNCFLYCAMYPEDEKIWVEELVRMWIAEGFLKSKTCSIDMQLGHR

YVKLLIDRGLFQSVNLEKQGRDIIANKPVKVWKKRFIKVHDVIHDMAIHIGEKEEKYLCR

AGLQRKDFPHTETQDCKRMSLRSNQVESLPEDFLSTQLVSLFLSKNPLTYNVLGGFLINL

TSLRVLDLSNTQIESLPTASLTQLRVLDLSNTKIESVPTSLIHLQFLTLRCTCIKELPKD

>AB_043512_T.1

MPVAFRNNIIFLTVSQSPNLKVILETMWEKIVRRKKPEFQNVEDAHRQLQQQLRRQAKPT

LVVLDDVWSRANLENLLFEGEGYKTIVTTRDRSTIPTSTSTNTSTRIYELPLLDEADALS

LFCYWAFGQKSIPINADEHLVKQVQAECKGLPLALKVIGSSLHGEPRPVWESAKNKLLNG

QSISDYHKEG

>AB_043573_T.1

MEAQGDIEIEALDENDAWELFGKVAFRLCDVLPGNNIYDIARKVAGECKGLPLALEVIAS

AMIGKSSQDDWKLVLRQMETVDATFTALHPLVEEKLYRPLRWSYDCLPANLPANLKACFL

FGAVFPEDTIIDWNELVEMWIGEGLIETKTWESLMATGQRCVQFLIDRSLFQQVGL

>AB_043598_T.1

MYPTGLEEKLEDFETTVLSRVSVGSKVVGIVGTGGVGKTTLAKHFFNSRRSNYDGSSFLF

DVRQKTGASESLMDLYRTLINDLKHKDTKILSPDHGLRRLKIELESCRVLIVLDDVDDAT

QLEAFLPVSNSLSFDSLILVTSRDKHVLKGVLESSIYNLKGLKIPSSEELFCSYAFCRRD

PPKEFADLVDQFITACDGLPLSLKVFGALVAGENSRDYWKEMLNRLRETLPTDIEKRLRI

SYDSLTKEDKPIFLDIVCFFIGEDRDTAMRISGIVGLTNLENKCMLEVDSKNKIKMHDHI

RDMGRNIALEGSMPRRLYRMTDNNDDLSDQWSNVITEAVRGIKMVSATDDFFQSPENFSP

VSMTTTVRESWYRRWTDQMSRHPINTLQFHGMGKLQFVATRGHYLEGMLSGEQSPNIIWL

RWYGCPYSSLPSWIPMKNLKVLEVEGRRLNTLWLDESPAPVQLRELKIWAPLSVFPQSVG

RLK

>AB_043895_T.1

MAASASDPDPDPDPASVDPNNNNNNYHRGPDVKKTFPSHLYHRILSDGLRVFLDYQELQE

GENLSSQIKGAIATATVRVAFFSPRYAESNWCINELLLMLDCGAPFIPVFHQVKPTDLRW

TQGEGVNARALHNLERKRTYDSQPRYSSTTIENWRNALSTVAKMTGLDMEAVNGDEGELL

DKVLRMCVDKG

>AB_044065_T.1

MMQMQRSSHERKIIPVFCDVEPRHLKHIDKPPYDVAFLEHQRAVDAGEVEMEVLQGWKSA

LKDAADISGLVFKTDESDHGKFLERIVKIVLKAIKWDLSATYPVGLDEAAKDFQKKIMNQ

SDLNAKKTVAVIAGVGGVGKSTLAEHLYNVRRSDFSRCCYLLNFRRKDLPSLQQELVRDL

VNYDVRNISDQGTSIFRNGLSSLRVLVVFDDVDDKVKIQSLLDMVVLGPGSLILITSRDK

DILRGFSANTFLYEVKPLKRKHAQELFCRHAFRQSKPLQGFETLVVEFLEICGGLPLCLK

VLGERLAAHLENAYWKERLENYFRQGRPLQEAEAVINTLQVSYEALNPEEKNIFLDVGCF

LVGEDTELAVRVLEGLGWHNARIALESLCTKCFLDFSTREESVDPNPYIHPIPNVLSRGC

SKIIMHEQVRELARHIARNDFTNPLRVSCSYDIQQILQLQRTSIPCKIRGIRIPKGQKPP

LLTIEITGVRLLVVDNPIDFSSFLGSSIRSSECMWGDLVWLRLGSCSLRSINPSTITISL

ENLRVLGLEGFDFDLEYFLERVVKERYPAHLQELNIQYSEAASSLSRTVSTSRTANMFKS

SRTSGMFRTICLSKDLNKIDFQNNRHMRLFPSDSFDFTNLRHLDLSNCINLRRLPKSFSK

CLRLQYLALRYCKKLSFAADFLGEILTLEYVDFFGCSKLMHLPRGMAYQRSLRCLDLGST

NLSQLPDNLKLLVKLEELRIGSPDLTEVPDSIGHLIGLKQLILSECPRLRHISASIKRLT

RLERLMINNLGVSSSLMQVEGLPSSLVSLMLMNCSKLKALTNLSNLVNLKILNIFNCKEL

ETVNVEGLIQLEEIQACSSLKLKRIIALSQLKRLNFLKLQIYNENGLICDDICQLLTDDH

REFVTSRSQIS

>AB_044550_T.1

MQPFLDEEELKGGDNLTSTIEAAIRSASVHVAIFSPRYAESIWCLKELVLMLESGAPIIP

VFYHVKPSELRWTRVNKKRGRTRVKNGAFAQALHKLESKQRCDSRTIETWRDVLSRVGDT

SGFELEKYNSEGALLEAVVERLLEMGKGERGTDQRLRV

>AB_044564_T.1

METFLIGAASNTLSSVVTVSIPPLIQFIDDVINFDQNTQLLQDKLRGLKVLLQDVTNQFQ

HDQRSPPETVDYWLQKLHHSLQEARRLIHRSQQQKQCLDCLFRKLRLSTQIREWRTNFDE

LLSDLQKAFSVLHDAQQIAASTPLPPETLLQPVPEAGFVGLGIQDAETMVQSWLNMEDHQ

FSVIGIYGVGGIGKTSLLRRVYNTCKQGNVFQAVIWTTVSQIINIQSMQSDVAEAINLNL

ESSSNAERRKMKLFTSLKEKKFLLVLDDLWSPIELEERVGIPVRNEKGSRVIISTRSRDV

IRRMGGSNEYSIQIQPLSEEEGWELFCRKAFKDGVPPTKIIEEVARLIAGECKGLPLAIN

VVSAAVMGNE

>AB_044655_T.1

MRKMKLYACLENKRFLLILDDMWSPINLNRMSVKFGKKKFILILDDTTTCGVQ

>AB_044945_T.1

MASSSEIEIEVEKQPSPAKPHASSASSSGGADPTSSSSGDYGYDVYINYRGPDTANTFAS

HLYHGLLSHGFRPFLDRRSLEKGLDIASQIEHVIRSASVHVAVFSPHYAESECCLRQLVL

MLESPAPIIPVFYQVQPTDLRWTGEGKTGVYAEALRQLQQKKTYCSQTHQDKPRYDPATI

QKWRDALSSVADISGFDLDGKFNGDEGEMLRKLLKRVEKIPRKTAFHVFISHRGPDTKKT

FASHLYHLLLSHGFRPFLDQEELQVGINFPSQIEHAIRSASVHVAVFSPKYAESEWCLRE

LLLMLESRAPIIPVFYRVKPTDLRWTGGVYAEALRRLQLKKTYCSQTHQDKPRYE

>AB_044987_T.1

MKLCASLKNKKFLLILDDMWSTCPVDLHQELGIGFGDDKGSKVVFSTRSRDIILMPGQKS

VEVKPLLPEEGRVLFNRLAFEGGNVPEDLKDCARETADECKGLPLAITVVAATIRGKTEV

DDWILSLSQMKNADPSFPNTHPRVDKELYQKLRWSYNDLPRADPNLQHCFLYCAMYPEDK

KIDVDALVRTWIAEGLVQSRGAAYSMDMDLGRSYVKLLIDRGLFQNVSFEKRISKGNSKK

PVNV

>AB_045514_T.1

MKLSASLKNKKFLLILDDLWSLLDLEQELGVGFGVDKGSKVVFSTRFRNLTLMREQYSIH

VEPLLPEEGRALFERLAFEGGHVPEELKDCVRETADECKGLPLAITVMAAAMRGKTVDEL

NVSLSLMKNADPFFPNIYPCVDKDLYQKLRWSYEELRQTPNLQHCFLYCAMYPEEEKIDV

DTLVRMWIAEGFVRSKEETYSKDMDLGRSYVKFLIDRGLFQNVSFEKQISKGHVKKPVNV

WKERYITVHDVIRDMAIYTGAKEKFLCRAGQQLKDFPHIQTQDWERISLFRNQIENLPKD

FICPKLVSLFLSENPCSVPDTFLIKLNSLRVLDLSHTQIESLPPSITQLTSLEFLTLSST

NIKELPTAICNLFRLQFLDLSLCRGLRSLPPEMSQLTSLKRLHLGMGMEKALDDPTNVMV

LKGLTNLIELRLFLFSKFDALATIMGTWLEK

>AB_045602_T.1

MANQDSVASSSSASISYPRYDVFINHRGPDVKNTFAAHLYRRLIDNGLQVFLDKSELLPG

HGITPQIKAAIEIASVHVAIFSPRYAESPWCLEELELMVKSTATKRATILPVFYIVKPSD

LRHNKKGDYAKALLELEQKKTDCETGEEKPRYPSCTIQNWRSVLSQAAEISGFELEGRVL

YQAAEISGFVVEGEEEELLTKVVEGVLKNVPKTPLTKIRQ

>AB_045711_T.1

MAHLTASPAPSSSAMAHLTNDNASSSASTSSYAHYDVFINHRGPDVKNTFASYLYRRLLD

HRLRVFLDKPELETGHNILSQIEAAIQAASVHIAIFSPTYAESNWCLDELVLMRNSRATI

LPVFYKVKPSELRWTVKDGAYADALRKHQQKTRSDPQTSEEKPRYDPNTIQNWRDALSHV

ADISGFELEAFNCNEQELLDKVVQRVLKIVPKTPLFVATYPTGLDDKLQDFERSVLSSEK

QESVKARVVGIVGGGGIGKTTLAKEFFNRRSSYYNG

>AB_045757_T.1

MASSSTTLAASPCNPSFDVFINHHGPDVKKTLASHLNRSLLSQGLRVFLDHPQMTPGDKF

PSQIKDAIRTATVHVAIFSPGYVESEWCLNELLYLLESGAPIIPVYYHVQPAELRWTQIM

EDGVFSKGLRKLEPLQDTITIQKWREALSLVAAQKRGLDLQESCNGDEGELVEKIVQRVM

EEIKRILSVGMLDFYRSELYGGLGGSGRSDGTYSGVRRISMTADKNSLTSLQLHYGLDGD

ESVKDIHIEGFMRGTKVGSFKKYKLNYPVEILTKISGYIGSYGSMAVIKSLTFHTNERKY

GPCGPEQGTYFDTEVGGKIVGVFGSSGTLVDSIGVYWLKQRTSKH

>AB_045781_T.1

MTSTQLYELPLLDDGDALSLFCFWAFGQKSIPSTANEHLVKQVQAQCKGLPLALKVIGSS

LHGEPWPVWESAKKKLLNGESISDYHKEGLFKCLETSIDVLDEEARECFLDLGSFPEDRK

ISVDALLDIWVYVRKIEWQDAFVILLELASRNLLNLTSNLRSQAINYGSASELYFSQHDV

MRALALDMASRDRIVCRKRLFMPRKEESVPGKWELFKDQAFDAQIVSIHTGTMEENQWCK

MNFCEAEALVLLFSATNYCLPSFLSKMRKLKVLMVFNYGSKRATVKGLPLLSSLAQLKTI

RLERLIVPPLQEHSKVLQNLEKVSLSLCEGLGNMSRFNGNQSNLKLPIMLDFNMDHCCDL

EELPVGICDMSSVQKWSITNCHLLRKLPDDLGRLSSLRMLRISACLGLKELPASIGKLGK

LEYMDISLCECLKELPEEIGQLKKLEELDMRECSRLRKLPKSVGGLKSLKHVICDEKIGQ

QWNRLKSFSATMDLRVEIVEAHFSLDWLDG

>AB_046081_T.1

MGGICKTSLVKRVYNTCKQGNVFQAVIWTTISQNFSITNLQKVIAGPKKMNLESSLDAEM

LKVKVNIFTSLKKKMKFLKKMKFLKKMKFLLILDDIWSSVDLEELGIPAGNEKDSKVIIS

TRSRDVILRTRANDYSKQIQPLSEEDGWEFCRKAFKDGGAPTKSIEHVARLIVG

>AB_046148_T.1

MAASTSTNNHDYDVFINHCGPDTKKTFASLLYHRLLSFKVRVFLDQPELRKGGDFSCQIV

STIKTASVQVAIFSPKYADSKWCLDELVLMLETLETGATVFPVFYDVEPSELRWTQAGGK

GRYAASLQRHETPRSPGEQPRYDSITVEKWRSALSKVAGITGFELKAFNGDERKLVDAVV

ELVLKKVEKRTLNVATYPTGLDEKVKEFENKILSQRPQSANPQLVGIFGQGGIGKSTLAK

ELFNKKGSEY

>AB_046163_T.1

MACSSSSSPPDTIGTSSSTPSTSTTEHHHIYDVFINHRGPDVKKTFASHLYHRLLSSGLR

VFLDQEELQQGENLTSQIEGAIATASVHVAIFSRGYAESNWCLDELVLMLKSGTPIIPVF

YGVKPAELRWTQSKDRVYGNLLRTLVWWPLGIHDMFAKIRRILGWWTRRKDGTYEHALLN

LGKKKTIDPQTNEEKPRYEANAIENWRNALSRVAEISGFELEACNSDEGRLLNKVVEQVL

RKVKRHH

>LL_000030_T.1

MIESICHTKSSTMVLQMIMGSQLYPILRFSLFAAFTWLVLKQLFLCFISHSIYLSLEDMK

TVSSATQKPLAIGTSGTSRKLSVDDNGAPSRVIHYDVFINHRGTDVKNTLADTLYEILKF

AGVRGFLDREELGRGEALSHTIADAISTASVHIAILSRRYCESSWCLDELCQMLDSGALV

IPVYFEVTPDDCEFIDRGPFAEAFNKHYTQGRTDRALIQQWEKALKMVTLRPGKRLDEYN

GRHGLLAMEVVAEVLKEVNKVTLEEAKYPVCLEQKVEELCTLIHSHETSTRAATIVGIVG

IAGIGKTTLAKALYNRIHSDYKAASFLSNMRDTIRNKGLQALQTMLFKDLFHVDWHVRSP

SEGKELLRKRLNVVEDILIVLDDVGQHEQLADLLDLNALHPNIVILVTSRDRGVLQQFRD

CLEYEVKRLEATEAEKLFCLHAFRKEEADEDLRDLVVKLVGMCHGVPMLLEQCGGRLYGK

DERLYGEDKRSYWESQLAKLSKQLEYHTEDV

>LL_000031_T.1

MLQRFKGCLEYEVKRLEATEAEKLFCFHVFGKEEADEDLRDLVVKLVGMCHGVPRLLEQC

GKRLFGKDKRSYWEFQLANLSKQLEYHAEDVQIADGNSEQEGFFTYEI

>LL_000078_T.1

MGSTILQSLEGAVAQIGLETGFSGVAGFANPILLGKALGEKLKETVSLLKPIIDEYIAII

DEYIEKSSVSDPDLPEHRREHFIVFQAVLQRALDIVRDSDQIHSLDIKAKYDYGNKILEI

NQEIEDFIRIQGPPNLALDLQKIIAEVRNLGRRFELIERLTLQTVNLFTQLVPDMPREVV

GLYKPIIDVKQILMNNDVSIVGIGGMGGSGKTTLATALCNNPEVQASFENNILFITVSQQ

QTENGVLEILKIMWDHIIGGDRPRFRSIEDAHTQLQNNLKRIAERTHRPTLVVLDDVWSR

SNVKNLLFEVEGYKTIITTRDKSIIPHVDGTRLYDMPVLEDADAVSLLCFWAFGQTSIPA

IAKEDLVKQVAAECKGLPLALKVIGSSLHSKPRPVWENAKEKLSRAEPITEEHKDDLLSR

LKTSVDILNHKERQCFLDLGAFPKGKKFSVNALLDIWVYVRGMEWHEAFDVLLESASRNL

INLTGYPGSAAIEYSCASELSFSQHDVMRDLALHLSSQDSNIHCRRLFMPRREAKMPTKW

LNTESSGAQFVSIHTGAMEEQDWCQINFPEVEALTLFFSASPYCLPTFLQSMPKLKVFII

YNYSSKRAILSGLPSFPSPVQIRSVLLHKLIVPPPLYRNCISGERLEKLYVCLCEGLGNI

TLLDKELGALNSPNMLEINFDHCSDLKELPVKLCNLTSLQRLSVTNCHLIHNLPDDLGWL

SSLRVLRLSACLNLSRLPPSICKLGQLEYVDISVCRSLQDLPTEFDELLNLETLNMRECP

GLKKLPKFKFRSLKSVIICDSGNETLRAWLDDIKTPGVHNLTIDVVEQRSSLDWLDD

>LL_000149_T.1

MADQTTRNNDNISGASSSSMASCSCSTSYNSYDVFINHRGPDVKATLGMDLYRSLNARGL

RVFLDNPELEVGQNITPQILHAIETAFIHIAIFSSTYAESKWCLEELLHMLKAFQSRPNE

VTIIPVFYGVEPWEVRWTNKGAYAEALSNHEKKQRFEPETIQNWRRALSEAAEISGFDSK

AYNGNVGRLLDDVLRCVSKIRDRTPLKYVAEHPTGLKEKLEDFESTVLWPGKAKVVGIVG

TGGVGKTTLATEFFSKRRSNYNRSCFLYDVRETAAKGSLTSLQRKLLKELTELDLPIDSI

KEGTSTLVRHLSSSHILIILDDVDEALQLEAFLPLKEIMKPNSLILVTSRDKKVLKKAKI

EQTSIYMLEGLNRKHSEELFCFHAFGQPHPQPKFADLVYQFITACDGLPLSLKVFGALLF

GENERSYWLEILNRLHQLPPEIQERLRISYDSLNSEERQVFLNISCFFLREDRDTAARIW

GFMELLNIENKCLLKVNSENKIEMHDHIRDLGRSIAEEEEIMPRGIWRHTTSTIENLLER

SLRVITNVRGIKTVTREDLISEDLISEAMMVRSDMSWHKNEVLGMESSEMSWYKRWSDEI

FGNCLRDLFNNFRFRGILNLQLVATKEDYPERILRRARSAHLMWLRWYKCGNSCLPSWIP

MESLRVLEVAGAKLKLLWQHESQAPLQLRELKLWAPSFSNFPRSVGQLKQLEKITIFQAL

IKTVPEEFCDLRSLKYLELIKCSKMMSLPDSFGNLTNLQHLDFSGALSLQMLPKSFGNLT

RLEHLGLRDCCSLTFSNETFGNITTLEHIDISGCGSVTELPPQVAHQRSLQILNVCKTNL

KELPSGIGDVCKLEALELGGEPLLEILPSLLGCLKSLKELWLFNCPELKCLPDDFGRLTQ

LMELKIWNCEIEYLPQELMGMKNLEILIIEDCPLRELPFKKAEGELDFSDGKCMLKLKLL

KLAGTQISELFFNGGVCPNLQCLLLVRFSELRGIEGLCRLAKLQYLYVSPRVKVEALPGV

EQHMKSVKESDVNEYSSELEILQRRGHFTRGMIYVNKCHEILEPGSLDSWLLPSAFWVSN

R

>LL_000156_T.1

MNIITSSKSSSEELKQKLLLLKPTIDEISKLSSDSDPARQKKGRSLKGIQAQLQDGINLV

EKLERMSCFDLYRRYRYGKQILKVEKNVNDFLLTQGLANVMLDVHKLNVDVKGCSERSER

IEEMGRHILENVNAKINNDASFNSLMLQQMNSIQLFQSSFDELHDTTMAEQSTGSCNSQV

PGMPSFVVGLNNLVNDVKQVLFQNGVNIVGIEGMGGSGKTTLAFALCNDSQVKDFFQNNI

VFITVSQTPNVKDVLETLWDKVIGAPRPVFQSIEDAHFQLQKNLSLKGNQQILVVLDDVW

SRSNVEQLLFQGEGYKTVITTRQGYAIPSTNSSRVYNIPMLQKADALSLFCFWAFGQPSI

PTTDDEDLVKQVEAVCKGLPLALKVIASSLRNEPKPIWENAKKKLSRAEPISQYHREELL

NCLETSIDVLDDEAKACFLDLGAFPKGRKFNVDSLLDIWVYVRGMEWEEAFVILLELASR

NLINLTSDPGINSAEGGSASTRAI

>LL_000180_T.1

MDPFTSKVVDELLEKAFTEMKGVVNIIVSSKTSAKELKEKLSSLKPTIDEILKVQNPDSD

ALVRPYKIFQTDLQEGLDLVGILEKLHSYDIFRKYRYGKKIQKFQKRVYEFIQTHGPPDI

NLNLKKIEADLRPERLIAIVKAGMTNDPNSNAIMLGQIDATQGFQNPNQVFQNPNGHGMH

ETTAAQLSVSYTYQVTETNEFVVGLNNPVNAVKNFLLRIGVNIVGVTGMGGSGKTTLAYA

LCNDSQIKDFFKENVFFITVSKLSDVNSILGILETMWDKVIGGPRPKFRSIEDARNQLQN

NLTHNAERGDRPILMVLDDVWSASHLEDLLFQAKGYKTVVTTRENYTIPKRSDACHYKMP

MLEDNHALPLFCFWAFGQTSIPTTEVKEDLVKQVAAECKGLPLALKVIGTCLRNEPRLSW

KIARDKLSRAKPISQYYEDTVLHRLQMSIDILDDEPKQCFLDLGAFPEGRKLSVDYLLDI

WVYVRGMEWDDAFMVLLELAKRNLLNLTSDPGSRAIDYSCASEFCFSQHDMMRDLALRLA

SQDSIIQRKRLFMPCKENNIPKEWRSTLTNQSSGAQFLSIHTGAMAEQDWCQIDFPEVEA

LALFFAASKYCFPTFLHTMPKLKVVFIYNYGSKRATFHRLPCFPSFTQIKSVVLERLIVS

PLYEYCKSWESLEKLYVCLCEGLGNMTLLDTEEVLKLPNVVEINLDHCSDLEELPGKICN

LTSLQRLSVTNCHLIQKLPGDLTRLKSLKVLRLSACPSLSMLPPSICKLRQLEFLDISLC

RSLKDLPMEFDQLSNLKMLDMRECSGLKKLPKGLAKLRYLEHVICDCDENTKRQWQSIKT

SAIPNLIIESVEESFNLDWLDY

>LL_000648_T.1

MANKCYRCSCFFSCLPCLNTQPPVGSCENAPHLADANTHAIPNACPEAQSSSPNAAANSN

ASPAVATEKANERRHDPQTFPAPPFQASASHDASSPSAEGASTSAVPAQDAPANGGPSSS

IPTESDVVDEEKGNVDLEGLIQHISELLELIQKNQLDKKGSTDHHWPKSSAFTRLKEVLS

EGIEAASSLKDIIPATRSLLNGIGQAHWALAGLLIVSNILERFEYVSANRDECIGLLDET

YRLAKYVKQLKERSRMREGMEDIIQKAVEMIVEASIMCSVQMDSSKFSRFRSASRDKEKL

LKFQQELEKLYKQMKTRFDICLYDAIDCRRCRPPTPLEREYPTYAVGIEEPLKEVIKLLD

WESEKNAVAVIVYGLGGMGKSTLADAVFARLDIVGCKYSMVRLFDHITSTPNIVELQKCI

LTDLMMGTHEKEKITIPTFESGQQQISRMLEKEAAFICIDNVLGGDRLQELLPRDVLTAK

RVRVLITARDKGVRSKLKMETRDYAMTGLPQSEAMNLLNREMNNKQIDSTQLNEIIKISG

GIPKLLTVVAGFISSEEDKQKAFRIVMEDKKHLEGQAFGDIEHYVFAYDHLSERLKDPFL

DICSFFKGWNWDTVADIVGESELDMLEKRALVSKGRNATVSVHDVILMISSKRAEETRFN

FTDGSQIEEFLEKDISGIKGLWCDGRLDIPTIKLDSMSNSLKILNLGHPLGAKGKCNKRF

EKLIFFRCAFCNLPFNISQMPFHLKHLELDGTSYSNGLFELSSSDLQQLRNVRTLRLINF

AGLKRLPEELGDIIHGLRELTLSGCRSIEKLPRSISKLQHLRVLNIRDCDRLSELPEDFG

SLISLQILDLSSCSSIEELPESTSKLHSLKELKMRHCGKLKHLPENFESLNSLEVLRLYG

CESLKVFPKNFEKFQKLKPFLKTNREKLVDLLEEQNEKLREAGLVYNATLGFLKGVCQR

>LL_000679_T.1

MAYPTPSCSSFPQTHSQIEMENQTSRPATSVGASSSSTAADPSDSCSNILYDVFISHRGE

VKKTLASHLYRGLLSRGLRPFLDQQELQEGLHFPSQLKAAIRSAWVHVAIFSPNYAESRW

CLDELLLMQESGAPIIPIFYHVKPAELRWTDQSKNGAYAQALNQPRMKSRYDSATIQNWR

DALSRVADISGFDLDGLFNGDEGYMVEKIVERVMEMSKNPELYVANYPTGLNEKVTDFET

TILKQQQRSGNLQVVGIVGLGGAGKTTLSLELFNRKCSNYEQSYFLSDVREKAKKNSLHS

LQNELLKGLHPRLDVRINNINEGIGKLKRHLTSSHVFFVLDDIDHEDQLEAFLPVKDVLS

SNSLILITSRDRDVLARSGVEQSSIYKLTGLPRQHSLELFCSYAFHQPYPLNGFENLVDK

FLQVCDGLPLSLKVLGAFLYGNNDKSVWGDILQQVKVPTGIQKKLKISYDALSEDEQHIF

LDIACFLIGEKKNSAITLWDGSGWNGSLGF

>LL_000680_T.1

MAYTTPSCSSFPQTHSQIEMENQTSRPATSVGASSSSTAADTNILYDVFISHRGEVKKTI

ASHLYHGLLSRGLRPFLDQQELQEGLNFPSQLEAAIRSASVHVAIFSPNYADSQWCLHEL

VLMLESRAPIIPIFYRVKPAELRWTDQNKDGVYAKALHKLQMKKTYDPQTDQDKSRYDSA

TIQNWRDALSSVANISGFELDGPFNGDEGHMVHKIVERVMKMSKKPGLYVAKHPTGLNEK

VTDFETTILKQQQGSGNVQVVGIVGSGGAGKTTLSLELFNRKRSNYNKSYFLKDVREKAN

KNSLHSLQSELLKGLTQSDLHINNINEGIGMLKRHLTSSHVFFILDDIDHEDQLEAFLPV

KDVLSSNSLILITSRDRGLLARSGVLSIYKLTGLPRQHSLELFCSYAFHQPYPVKGFENL

VDQFLQVCHGLPLSLKVLGASLYGNNDKSVWGDILLKVPTDIQKILKISYDALSKEEQHI

FLDIACFFIGKKKNGAITLWDGSGWNGSSGFENLEGKCLVEVDAENVIHMHDHLRDMGRD

IAEEKLPRRLWRPTENIDNWLHQQSSVITEARGISIRQRDSSDKSVIINPSKLSWYQTKI

EKVFGNRMMNILYTLYRFLGIGRLMKLMYRCCRFLGIRNRVMELLYTCYRFFGVGAMKLQ

LLSGEGYLLERFFRRVHSPNLIWLSWSDYPYSSLPSWIPMRNLRVLRVQGGVLETLWQDK

SQAPNSFGNFTNLYHLDLSGCNKLERLPDSFGSLTNLRHVDLSNCQNLFRLPDSLGGLTN

LHHMDLSWC

>LL_000730_T.1

MAHLTNASASSSTSTAAPPNTILSSFCDVFLNHHGEDVNKIFARDLYRRLISHELPVFLD

NQEMRRGDSLTSQIRGALATASVHIPIFSPRYAESKWCLEELVLMLESKATIIPIFYHVL

PSALQRTRDEREVYGQALCNLEKMTMKDPQTNEEKPLHERNTIERWRKALKDVSEIRGFD

LQACKGDEGELLDKVMKQTLLKVPRPLLHVAQYPTALNEKVADIERIVLLQKQQHIGEAK

VVGILGLGGLGKTTVAKEFFNRKRSDYERSCFLSDVREHAGRGLLKSLQSKLIRDLTGHD

IRIDTLHDGIQILKKHLSGVQALLVLDDVDHFNQLDAVLSPVKAVLNSRSLIVVTSRDKD

VLLSSSIEESSIYRLSGLNGKQSEELFCMYAFRQPHSIDGFEEVVKGCVNVCGGLPLSLK

VVGALLCGKGLGYWEDQLHKLKRVIPSDIRQSLQISYDALDKDEKEIFLDIACFFIGEDK

DKAMRIWDGSGWEGWLMLHNLEAKCLVEIDNENCLRMHDQLRDLGRYLADKEDERGLRRF

WRPIDNFDLHNVFQHQTSVRGISRVQRRPGQDPVKELCKRFKNLTLSNRFPDNSGLQLLR

AEGDSLGSIYNLLRRSDLQWLCWDKYPYTSLPCWIPKMNLRVLEVGGDELETLWQNETEA

PLQLRELNIGAVLLRVPESIGLLKHLEKIVLKDTIEPLPEGFFHLHSLRHMRIRTKMMVL

PDSLGSLTKLQHIDFSESADLKTLPDSLGSLTKLQHIDFSGCADLKTLPQSMGNLINLQY

INLKKCFRLQMLPGSFCNLIELRHLSLECCDNLKELPFAIGNLCKLEILKLQCPLLETLP

SSLGNLTSIQELTLHFCTNLKCLPDSVGRLKHLTALTIHYADIEYLPEALTELNNLRTLK

VRACPRLLKVPLGTVEKVGLPDSSIEHCMFRLEHLDVQGTAIRELSFPDGFCPTLRLLNT

SFCLELVEIGALPTTLISLDVQGCSSLEKIRGLSGLAKLELLDISGCSEIEELPGLETLS

SLELRADGCDKLKGVGKNLMKIRRKSCNVHHFLGLKKRKL

>LL_000766_T.1

MDIRTAVAADAINKLIVAPVLQQIDDVIHLGRNRGDLQAKLLRMKRLLQGISDAFQNHQR

TPPQAIRDCLESTRDAIGEARSLIDRSQLQQCCLDCIICKPRISRDIREWNSRFDELLED

LQTLFSIFANAHQIASSSAQPPAQELLQPLPEWGIVGFDIKSAESQLQTLINAEAPQFRI

IAVFGMAGIGKTTLLKSVYNFYKVSNAFDVIIWVTVSQDYKISELQRCIAEVINLDLSIT

SDIDNRKIMLSASLKNKKFLLILDDMWTALDFQELGVSFVTDKGSKVVFSTHNRDLALEE

GKVSIKVEPLSREEGWNLFQRKAFRGSHVPEKLKECARKIADECEGLPLAIIVVAAAMSS

KRTVDEWSSCLSLMKNADPSFPETHPRVDQQLYQRLRWSYDELSKTPNLQNCFLYCALFP

EDLQIDVEQLVLMWIAEGFVKSKEATFCTSATGM

>LL_001086_T.1

MEPKPELQGPMPAAYHVFINHRGPDVKNSLASLIYYCLTKYHNLRVFLDKNEIRTGDTIP

DTIRNAIQSASVNIAIFSQRYAESTWCLDELLWMLSSHHERHRKVIPVFYDVKPTDLRHI

DNGCYKNAFEDHQRKGRVSNKEMEKWRNALNEASNLSGIEFKTEESDQGEILDKIVCIVL

EEVFKWGPLEVAKYPVGLEQAAEDFPFNEIQKQSELNQTVIVGIVGMSGAGKSTLAKYLY

NLRRPNFNGRSCFLSEVRKKDLPSLQGKLLCDLLGYNQGLHIDQGKHILRHCRILLVLDD

VDHIDQIDGLLDMDSVGCGSLILITSRDKDLLRRSSPKIVLYDVKPLSRQHARELFCQHA

FRQSKPSQEFDVLVEGFLENCKGLPLSLKVLGGQLLGFSDREHWKRQLAMFSTTLHNDII

KTLKVGYDALDDALDKEAFLDVGCFLTGEETELAIRVLNGLYGSSIVHSLERLHQKCLLD

FHYDVQSNDEIECRKQENRNIGYNDLSLQYRGYKLTMHDQLSRVPKAAYPTAA

>LL_001203_T.1

MHPTGLEEKVEEFEKTLLLQKQQSGKVKILGIVGMGGSGKTTLAKYLFNKERSNFGKSCF

LFDVRENAARRSLNSLQTQLIKDIAHRHAQIDHVD

>LL_001274_T.1

MVSRTPLNVAKYPTGLDDKVKDVERMVLLQQQSEKARVVGIAGLGGVGKTTLAKEFFNRH

RSKYVRSCFVYDVRENAAKSSLKSLQSTLLKELVQVNEQINSTDEGIGKLKKHLSCLRGA

VIVVLDDIDHIDQLDALLSPLKDTIHSTSLILVTSRNKLVLTSSGIVESFIYKQTGLNPQ

HSRELFCSHAFDQTCPLTGFEPLVEKFLDACAGLPLSLKVIGALIRGKDFEYWKAQLQNI

SEILPMDIQTRLRISYDGLDAQEKQIFLDIACFFIGEDKDTAIRVWDGSHWAGRLRFRNL

ENRCLVEVDSENRIRMHDHLRDLGRDLAEKEPPGCERLARMAGSSEPRKQMPC

>LL_001293_T.1

MASTSASPSYAHNNCYVYDVFINHRGPDCKKPFATDLYNRLREYRLQVFLDQQELQEGED

LTSQIKGAISKASVHIAIFSKNYAESKWCLDELGLMLQSKATILPVFFVGVKPSDLKWTG

QDGVYARALRNLEEKTTFDIETQKEKPRYHSDTINEWRQALSVAAGISGFEYKGDEHQLI

EQVVEGVLKIVKRPVYVSKYPIGLDLKVEDFERVVGLGRSSETRVLGIWGLGGVGKTTLA

KEIFNRQLSKYDRSCHLSDVKNIESSQKQLLEELGARSWNGQIYNSDDGIEKIKRYLPST

RALIILDNVDHMKQLNALLVPVKETIQPGSLILVTSRNKDVLTNGGIVESHTYKLEGLDP

QRSQELFCLHAFGQPHPVAGSDFERLVEEFLVFCQGLPLSLEVIGELLHENNDLGFWEAQ

LKEISTKLPDDILGRLRISYAGLDPKEQQMFLDVACFFIGQDINTAIRIWDGSGWNGWLG

VRKLENRCLMEVDSKNCITMHDHLKDLGRSIGETDPSSYRLWRPTENLLRSLLDKFPVRG

IDMVHQYAGSEWPDPQSFKIFAERQGSGLVTNISNLQLLRAVGNFVESIFSVVPSQERVG

LSPKLVWLCWHSCPFTSLPPWIPLNKLRVLEIKGSGLETLWPDDGQAPLLLRELIIDAPL

SKLPVSIGKLTKLEKIILHQNELVEEVNLNSFLDELCHLKSLQYLVLRNCSNMKSLPDTF

VNLTNLEYIDISGCYNLQKLPNSFGKLKKLQLIDLSGCLSCKTFPPRRWKRTSQN

>LL_001324_T.1

MANLLASSSTSSGAYDVFINHRGPDTKLSIASHLYSRLSPLGLRVFLDKEELEKGVFFTS

QIAAAIRAAFVHIAIFSPRYAESEWCHNELLLMSESMESGRSIIIPIFYGVKPADLRWKL

GRDGAYAKALTRFLMKKTIDSQTHREKPCYDCGHIEKWRNALFNVSHISGFDLDGAYDGV

EWQLVNEVVELVLEKVKKPPLYVSKYPTGLDVKVKDFEELVQQQSSGTRVVGIVGLGGVG

KTTLAKEIFNRVRSNYKRSCFLFDVREKSVNFLNSLQSVLIKDLTQLNVQISSIDEGQEK

IRRCLLSCHSLIVLDDVDDIEQLDALLLPAMDVLDTNSLILVTTRNKDVLISLDIPESSI

YRLTGLNPQHSQELFCLHAFGQPHPDVGFDKVVKEFLDSCHGLPLYLKVLGALLRGKRDL

NHWKRQLRKISKILPDDIQRRLKISYDSLDEEEKQIFLDIACFFIGEDMDTAIRIWDESG

WEGCFGLRNLEDKCLVEVDGNNCLRMHEVLRDLGRHIGRKEPPDRLWRLTGNLVHDLPDK

STVRGINMVETPVENLTELGNRFVGMHGLKLVRAEGDCLEESIFRSVQSQHIIWLRWDMC

PYSCLPSWIPMERLKVLQVGKSKNLNTLWEDHNLQAPMQLRELNIIDSGLLEIPKSIGKL

KHLEKFALTSQFSIDLKTLPEEFCHLQSLKHLRLIGCSSMKSLPDSFGNLTKLQHIDLVC

SRQLKMLPNTFGNLGNLQQINLHLCCELEKLPDSWGNLSNLQQINLSNCYKLESLPDSFG

ELKNIQQINFSFCSGLKRLSNSFGELKNLQHINLSSCSELQRLPDSFGALKNLQHINLFS

CSKLERLPDSFGELKNLQHIDLSLCSKLKRLPNSFCNLIHLKHLNLNYCSELNIFPETFG

KISTLECLYISGCPKIEVCPPQVAHNRLLLTLFLVLTNLKELPSNIEELSNLQVLHLGSE

WLEMLPPSLGHLRGLRELVLSNCLQLKYLPDNISSLRSLQELVLSKCERLKCLPDHIGEL

RSLHKLELSGCKKLKCLPDSIRLLTQLRELKIKKSGVEHLPPSVMELNCLEQLEIEDCPL

SGLPFRKVQGG

>LL_001328_T.1

MAVGDLVEEKSWDLFAHHAFPYSKGNLPANIDEMTAKLVCAKCGGLPLAIKVVGRSMAGI

TDAQEWELAVRRLPNEKKQDHQALYDRLKWSYDSLGNYHVNLQLCFLYLAAAFIEDQIVD

VEVELISLWVGEGLLAHNKLGDQAGRNEDPFEIGRIYVNVLADRCLIEPIQRYFDGRVLR

FRVHDVIRGLGIQIAEVEEKLYCRTGMGLTALTPNECSGCTRVMLNDNYLSSLPQLWRAP

QLCSLLMVGNKLLTEIPRKVIGSMVSLKVLDLSWTSLESLPESVGCLKQLVCLRLVGVPI

KRLPASLTTLANLQILDLHRSSITELPSDIDRLRCLWHLRMSKCDDLQCLPCSISGLTSL

QRLDMTYCRKVWAKRDKRRRWKKVASIGLLSTLTQLKALRVHNNGETMSEGTLGSMLQME

TLDLQLTEMMNVPDDIFNMSKLRRFGLECSHMIKMESKFCQFQHLSHLQLWNCPRLEELP

DLHKLSSLKKLDIFKCIKLKKFPKEFTETGAFPSLEIFSLAYLNELEELSLIEQGAMPML

KIFTMMKCEVLQILPESYLSLKALQKIRVYGCSMVSDNLHPLKIVDTKVEVVTMSTTDTQ

EIIEKYLQVRNSKAGWSYGEFWYNELWLFLRELYTYI

>LL_001380_T.1

MASPLVIQSVAGAVTSELLSVVIYVLKKSFACKGACKLLQNTLEGIVPVLADDFNSGVEL

SQNSQKVLSEFYQELQKGIELVKKCSKVNRLNIYKSYRYAKKIEKLDLYIVKFTQTQGWA

HICSELHGLRLHHNKVEDIQHKIVDKLENIEQTIEAIKEESEIKFITDHMDQLEIGEKTS

QLSVPDLPDFQVGLNSVVHELKERLLRHDVSLLGVKGMGGSGKTTLATALCNDPQVKVHF

KERIVFVRVSPSPNLKGLLERMWGKIVGGKTPDFHDIEDAHIQLQQRLHQMEPYPTLVVL

DDVWEIDHLEKLLFNGKGYKTLITTRNNIVVKSDYHRYDLPCLREPDAVSLFCFSAFDQK

SIPKTADCNIVKEVIEECKGLPLALKVIGRSLYGQPPEAWITAKDKLSKGQPISEYHKTE

LLKRMATSIDILEEEVRECFLDMGIFPEARKISADPLLDLGVYVHKLQWAEAYTILIELA

SRNLVNLVNNRKCTVNSYGSSCELSFIQHDMLRDMAIYLTRQDPIINRRKRLIMSQKADS

IPKTWKKAAKSAFDAEIVSFHTGSMEEKDWCQMDFPNAKALILKFSASEYFLPPFMESMP

KLKVLMIVNHNSKRATLKGVSVFSSLTRLKVVRLERVIVPPLQEYCQSWQKLEKLSFILC

EGFGNMSRLHMGLHLNFPRLSEFTIDHCSNLKELPASVCNMASLQRLSITNCHDLLKLSD

NIGNLSSLQMLRLYACLSLEKLPYSICNLRQLQFLDISLCGCIKQLPDELGRLSSLKEID

MRECSRVKQLPKSASDLRSLVHVICDEKIGHQWSKIEASTNPRLRVEVAEEEFTLRWLDD

>LL_001646_T.1

MADKREVAVYAEAMRIHENRYDQQKITNWKRALDEVSLISGFELEACNGDEGELVDEVAR

QVLKKVRKGLLPVATHPTGLDVKLQDLEVILERQQHREGPNVVGIVGLGGVGKTTLAKYF

FNSKTSDYQQSCFLPDVRDAAVRCSLPSLQIKLCKDLTGRDPSKVDNVDEGIAMLKDRLS

CTRQALVILDDVDNVVQLDALFSPVKDALPSGSVVLLTSRNKEVLRKSARIAESSIYNMT

GLGFEHSQELFCSHAFLQPHPAVGYKDLVIKFLDACGGLPLSLQVFGALLYSENREVWEA

ELLKLSQVLPEKIQSRLKISYDSLDKQEKEIFLDIACFFIGTDRDTAIRVWDGSGWGGSL

NLMKLQSKCLVTVDSENRIRMHDHLRDLGRDLAENKPPECPRRLWRPADDLFRNVPPQSA

VRGISLDHPFGVYFQSNSIVEMSGVRLLRGPLDSWESQSIMKVMKRSDLLWLHNKYWLSQ

EIRLGVFELMDAFRIPINNLRVLELGDPGDSLWGGDCKTKLWNEESEAPSELRELTIHMH

LDGLPKSIGQLEHLEKIQLNSFGGSSLPEKFCHLRSLKHLTIWATTSTLMLPDSLGNLTN

LQHIDLRNFSFQTLPDSFGNLTKLQHVSLKDCPSLIMLPGSFKNLIRLKDLDLGGCCCLT

LSGETLGNISTLEMLKLSGCEKIKELPPQVTHQQSLQELILEGAPLKELPSTIEHLGNLE

ILVIGCRLLEILPASLGNLTSLKKLKLCDCKKLKSLQDSVGKLKQLTSFGISNSPIRNLL

VELNNLKELTVKNCPIRGVPFASGRSLDSSIHRCMLQLRSLTMWWTRITEISFGEGVCPV

LQYLDLRHSRKLVKVGALPTTLISLNVKYCHALRRITGLRGRDQTKLIIETNKVLQETRV

YGGVSDLFYDESMSGSLDEWTSEAESMSGSLDE

>LL_001825_T.1

MEQVTGQYHVFINHRGEDTKLNIAGLINRELKRCGLNVFLDREIRKGDENCSAIREAIRS

ASVHIALFSKRYPDSEWCLKELCWMLNSSHQCRTIPVFYDVQPSDLKETDREPYATAFQN

NPVKDKQFVEEWKEALKKASFLTGCIFQTGKSDYAQFLDEIVESVLEVVKWDSPDVAKYP

VGLDQALDDLQNKIQQKCGTKVVGISGFGGIGKSTLAKHFFKARRSEFDRSCYLAAVKDR

DLASLQRLLLHALQANITPNTNFGKGIFQNTVRGLKILIVIDDVNDRRQIKNLLDIDAVE

PGSLILITGRDENILRCSCPANTLLYDMQPLQRQHAQELFCHHAFGRQYSEPPEEYKLLV

GVFLNFCEGFPLCLEVLGEQLNVNRNITYWTETLETASKGMPLRAADPVREILKVSYNAM

GSGDQELFLDIGYFFVGEDADLAVRVLEVLGYHNVQEGLESLRQKCFLKFDNDVDFYHEK

DENPYRENQSLTQTPCKYGDNYLDLEFNLPGVLRRVNSKIIMHNQVRDLAKSIVRQNLGS

RDKPQRLSCEIDIKIILDSQFGSDPYLVRGIRTPKGQDPTHLTNHNPTAVLKGLRLLVLE

HSSHLRSFFDSAISWNLVWLRLRNLGGFKSINPSAISLQSLRVLEFQGSTDHLENSFSHL

KNLFNERNDEIPSRLLELNIDAKGGVSAGTASSHESAMPSFYSFLGWIGTKLTYLNKIVL

KNVLILESLPIDFSKLTDLRHLDLSGSINLTNLPTSMSQLLKLQHLALRDCRNLSIPRDI

LGEISTLEYVDFKGCAQLEHLPEGIASQTRLRSLILLDTNLLEFPLDLDLLKLEQLRISS

KHLTKITGSGASLKGLKELILLGCVKLDNITPIENLNLLERLEIYEAALPPILPSGILRL

KNIKDLKIDFCPIAEFYFQADHVMDALRHFSARSTEISNICIPTDVCPRLEEIDLSYCSR

LMLVQGLPCTLVRLILKGCSGLKTLTNLSNLVHLRFLNLNGCFKLETVNVKGMASLEEIG

VEQCWNLQSIEAFNELERVNTLHVSTNLHGNRFSNASEFDSLMRQCIVSKFENLQVRDVS

AATTVRSPARLHNVRSHHGIVMCFITNRSSGNNFCVSFDPSNSHGLYEVYKTFRESRSHG

RMLHMYMWTEDSKLFKEKNLNTKLAVYHSGSGNEFDVRNLSTNCASDRRNNLDEEKGWIV

TLASKSQGSKVCREIMLEGLFKG

>LL_002070_T.1

MVDTKKTIIPIFYDVDPSELLHMLERKTTWEQRYGSGIIENWRKALSSAAEISGFELNKF

NGDEGLLVKEVIQRVLKTVPKKLLNVAMHLTGLDVKVKELEAIVSSQQQSGSLARAVGIA

GLGGVGKTTLAKQFFNSYISNFERLCFLSDVREKAARNSLISLQAQLLKELAEQNEQINS

SYEGKEMLRRYLPSSCRALICIDDVDHLDQLSALFFPVKHTNSLILITSRNKDVLRSAEI

EESSIYMVKGLNTQHSKELFCWHAFKQSYPVEGFEQLVDNFLVACGGLPLSLEVIGKLLY

GKNDLNNWKARSQMISIILPRDIKSTLEISYNGLDEEERFCVGKGISISGKSN

>LL_002111_T.1

MGTAHLATTGLLIVALVLERLETVSANRKECLCVLDEICRLAQHVKKLQQRPDLKEGMED

SMKEAMGLIVEAAIMCSAQIGSPKGWRFLSASENKDLLVDFQQKLQRMYRHMDSAMSICI

YDAANCRKVRPPKPQERPYPDYAVGIEEALKQVTELLEWESENNAVAVILHGFGGMGKTT

LADAVFARLYIEGCKYSMVKLFDDINSTPSPSDILKLQTYILEDLKMETLDKAIRWVEEG

RGEIGSILQREKAFIYVDNVLLRHPFEQLLPRDMVNAKKLRLLITTRDIDACSECTKMIT

KFYDMKGLPPEAAKSLLKTGMNSQTDDMEDQLNSSQLDRIVEICGGIPKLLEVVAGFIRG

QKNKQRAYNKVIEEKEKNWKGKIGKDIASYVFAYQYLDEMLKDPFLDICAFFNGWDWDKV

ANIVGDDELEMLEQRALVKKDPRRCVTVHDVILAMGLQQTERTRLTITNQSQIQNLEDED

VEKIKGIRLLSGKKGPMCISASIIDLMCSSLRVLELGDSIKVEGKCEKTFKMLIYFVDKG

FHLRFDVSKSEELKYFYGAAEDFNLFQMPSSLVHVELNGKCFNSQPFQISSQCLGQLRNL

RVLTLTNFSQLTQLPQKLVDVAEGLQYLRLEGCEHLEELPEFESKLRCLKELTISQCNSF

RKLPEDVGSLNSLQKLTLSHCESLEVLPSSLDKLSSLQELDVRGCRRLEVLPNGLDKLLS

LKLLDLSFCQNLKNLTFDLGKLTSLVKLRLGGCEKLRSIPQSISQLKLLKLLCENLKNLT

FDLGKLTSLVELRLHGCRELSSIPQSISQLKLLDSTMDLGFTGLKELPDGFCNLSFVTNL

QLGWCHSLKKLPDRFGELSSLVKLSLGWCESLKELPESFGRLKTLEHLDLSGCSTLEELC

RDFKGLLSLRVLSLNSCGMLKTLPEDFHCLTSLEILYLSGCWMLEGKWMESVVKIKTLQI

VDIGASPMLEERWAEIQRQGDQSSWTFAVHTRQGLPNDESKENVLKRLQAEFDSILTNGG

EPFHTSDLPLNTLLLVMFDGKEEFHDYFPLNLIEENIDDIESNVNMIYFGNHFNKLPKKV

ADRILGHAPANSNVQPLFQKVFATLGREEYGVFQSHFEMSMKIASDQKGDKYFSSWKRFT

WRQSVEEFVMKCGRYQRLKKLAEGSAESNTELLRELFATGEGEDIHCCFAKNDGSDKVGV

DKLKGKTILLDIRPVNLGYELPLQALTEMYVAQKETLNFEIITIPVNERNAKWDPLDKFS

SDIPWLVLQSPWQLTNVTKYFLVEGCARWLQAWDERDDERDDDDERDDEWDDDESDDEWD

EWYPNIMRVIDSNGRIAAHNSVGVLRMVNTWGAEAYPFTEEKSKELKE

>LL_002159_T.1

MAMAASSTFAAANPGNKSFAIFINHRGIDVKETFARSLYLRLFENGLTAFLDKEEMQAGY

HICSQIEQAVKAATVHVAILSPHYAESYWCLKELVMMLDSKRPIIPVFYRVTPAQVKWTQ

GDYAKAIQTHEKKGRHDPNTIRKWRDALFQVSNISGFELETRNGEELQLELLDKVVKRLC

EMVKKPADLYVAKFPTGLHEKLKDLEETVLLHQQQSGRDQILDDKPRQQSCKPQILGIVG

LGGLGKTTLAKEFFNKKKSYYDKSCFLSDVRDHEAKGSLISLQRKLLKSLIGLNERVLDS

VDEGKEMLVEALKSSKALVVLDDVDHVNQVDALLPLQTHFDLCTGSLILITCRDRHVLIS

SRVEKPSIFRLTELSTRHSRELFCSHAFNQTYPLPGFESLVDMFLKSCNGLPLSLKVFGA

HLYGKDIAEWEDELESLQQILPAEIQKTFLRSYESLDQQEKQMFLDIACFLIGTNRDTAI

EIWNRSGWRGRQGFQNLQNKCLVEVDSWNAIHMHDHVRDLRINIVPENISPRRLWRRTKN

GIDNVLSFENIAVRGIRMVHGCHDEDDVSDIIGTENARLRKTLQILDAENSLLEGILRRV

ELPNLLWLSWKKCPHSSLPSWVPTKNLRVLKVSESISIRTLWHREGESQALLQSPVCLEE

LWADGSVQLKSIRGLEQCTKLRILNIRGCCELEELPSLETLVWLEGLRAEGCVKLKSVKG

LEKCTKLRILNIRGCSELEELPNLKTMVSLEELRADRCVKLKSIRGLAQCTELRKLIVNG

CSELEELSTMKTMVSMEELWAESCQKLKRIGLAQATKLRYLIVSDSSELEELPSMETFVS

LEKLVADRCVKLKSIRGLTQCTKLTLLSVSGCSELEELPSMETLISLEWLWLNECVKLKS

IAGLAQCTKLRKLIVNECSDLEELPRMETLVSLEELWAEGCVKLKSIGGLAQATKLRYLI

VSMCYELEELPSMETLICLEKLVANGCVKLKIIRGLWQLTKLRKLSVQWCFELEELEDVE

HCTWLETRSVSVSACPKLQCPVFE

>LL_002230_T.1

MAEAAVVSALIDKLTSMVAHKLSQEVSLIVNFREDFEYFRDQLLSIKCLLADAGEKRNSS

NSSSVSNWLDNLEDFVSEAEYQVEKFGAIDNIFTKLIFRFRMGKKIKELRKRLQQIQQNA

QNLNLLRSALDLTAHRQASDADHRYKEKRGRSNALLQESQIVGMDDDIQVITDWILKEDS

RKTVIAIVGMGGQGKTLLLQHIFNSEEVQKHFDCQIWLAISEKFIVIELLREVVKQFQQS

ERVKQFEGNNENLADQLNGKKRAHLPNEKELTDLSEEALRDKIREHLDKKSCLFAVDDVW

DRYAWDEISLPSRLQDKVVVTTRNEEVAKAMGTGDQILHKNSLSEENSWQLFCLHAFPDG

AHHCPEALDKIAHQIVSKCGGLPLAIKTIGAYLARLVRGLPNDWERTSQHLNEAHGMTDS

VMPSLKLSYEALPAHLKPCFLYCSVFPKNTEIESEYLVHAWIAQGFVSAQLQEVYDVGCS

YLEELIDRCLIEVSEVGGNERLQSCKMHDLLHDLAVSEQTKCLVRPGGQLQRIPAEDCQG

LRRISLMKNEISTINNGIGCPGLRSLLLSHNDRLTSISASFFDNMRYLSVLDLSLTGIKS

LPHSIGNLKLLKYLNISGTPITELPKSLSRLRHLQFLDVSYSNVTRLHPGIDQHKHMLHL

NLDNIWADDYPVGISKLIYLQTLKGVRFTVGYATASANALQLRDLKGLTHLQHLSLTLYP

ATHFYRMEDEGTFRGMTKMRTLNVRSRRAALHLPNDMEAMERLEIVHLEFCVVPKWISQL

QNLMELRLVGPFNSSSADLTGLEKIPHLKKLHLDGNATSKLTEFPNEFGEAGAFLRLEEL

LIHRFRYLKKIPSFQEDAMPMLKCVRIKDCCPLENVPEGLTKLKNLKEVEVEMLVDDVEN

FEYDKRHCWQTFKDQQIKIKVEISNRSYSHPRSRRLL

>LL_002372_T.1

MLSLPLASEGNHILHTFDIFNCKKSSSRLDGLLAGLIQTVQPLYLSNLKAPKNEQNSIYE

QFLRILMDGVELVKSCEKTSLYNVYQNLRYASKIHQLEKDICHFVHYQMPVQLLQDVKNV

ITELKTLRNLYESRSVDERKVNETIVPKLTEDPFQNATMLQRMGPDGMFDGACDEAPPCR

YDDSIKNDFVVGLEKNIWNLKKILLQKEVSVVGVQGMGGVGKTTVALALSNDRDIKDAFR

NNILFITVSESPNLKGILETMWEKLFQRKKHEFQNVEDAHRQIQEQLLRQAKPILVVLDD

VWSRANLEKLLFEGKGYKTLVTTRVRSTIPTAVSTQLYELPLLDDADALSLFCFWAFGQR

SIPNSADEHLVKQVQAECKGLPLALKVIGSSLHGEPRPAWERAKKKLSNGEPISDYHKEG

LLRCLETTIDALDEEARECFLDLGSFPEDRKISVDALLDIWVYVRKMEWQDAFVTLLELA

SRNLLNLTSNLRPSRSQAINYGSASEMYFSQHDVMRDLALYLASRDSIVRRKRMLMPRKE

DSLPGKWELLKDQAFDAQIVSLHTGAMEEDQWCEMNFRQAEALILLFSASKYSLPSFLSS

MKKLKVLIVFNYGSKRATVDGLPALSSLTQLKTIRLERLHVPPLQDHSEILQNLEKLSLS

LCEGIGNMSSFNSTKSNLKLPTMLDFNLDHCCDLEELAPGICDTSSVENWSITNCHLLRN

LPDDLGKLSSLRMLRLSACLSLKELPASIGKLGKLEYLDISLCECLKELPEEMGQLRNLE

ELDMRECSRLRKLPKSVGGMKSLKRVICDEKIGQQWNRVKNMAIVELRVEIVEAHFCLDW

LDG

>LL_002504_T.1

MAASSSTAVHSSNNQWYQVFINYRGVDVKPFARSLYFRLLGNGLRPFLDQEELQPGDYIS

PHMQKAISTASIHVAIFSPRYAESHWCLDELLLMLESKARIIPVFYGVTPAEVRLTQGRY

AEAFKVHERKGKYDSMTIENWRKALSRVADITAFELEPGNGDGLEMQLLDKLVERILSLE

GLVESNKYSEKIGLDEKVEDFERTIFSEHRLRAKPQVVGIVGLGGVGKTTLAKEFFDRKM

SHNMKSCFLSNVRENAAKGILTSLQSQLLKMLTGSDVLIDSINKGIEALRKHLSSCSVLL

VLDDVDHLDQIDALLPNRTDLLPNSLIVITSRDKGVITSSGVENSSIYQLTGLNKQHSLE

LFCAYAFTQPSAPEGFESMVDKFLEFCIGLPLFLKVFGALLCGKNDQSEWEEVLDRLQQD

FPIENSLPQDFPIENSLQIIYDSLNTEEREMFLDCACFFRGLKRDSAIRIWDGSGWKGHL

GFQTLQNRCLLEVDIENNIQMHDHLRDFGRALGVTMLPLRLWHCTNIHDIVKPFPRFPVP

VRGIRMVLGDDVDAFGDIDMSTLQLVDTENAILERIPRRPMHFPSLLWLRWN

>LL_002588_T.1

MAEAVLVSAFVGVAIDKVITQILHHIGIAIRYKEELSSLHSLVRDIQPIIKEIQRCRPAL

NRKTGIPTSQCYKKASAVNIWLKKMDLLLRQASQMVHQCTLPRYTIISRYLTSRRITRVI

SDIKNHLELSRVVELAQIPELLEEQTQVLLEGFGQIMKESVEALASSSSSSATTSQSLAS

TGFFIPEIPIVGQDTAFATLMKFVIDSEFSRIGVLGKGGSGKTLLLKRIF

>LL_002751_T.1

MSSRRFLLFLDDIWDDSETLLEKLGVPDDHVNSSSKIVVSTRDRRVLSRMRVSNPISMDY

LSPDESWRLFCFHAFADNQHTPPGVIEMIARDVCAECDRLPLALKVIGAAMAGITTLPEW

RLTLDKLRNADKLDPQVEKKLYNRLRLSYDALCTEELFALQLCFLYIGAFKEDEDMYVED

VINLWIGEGLLPTEEGKDPLEIGRSHANFLLDRCLIEASIKDADGQVVACSMHDVLHDLA

LQIAEKEENCCFRAGKDLEVFPLKDCTGKGRISLMENKFKSIPDEFKGQDLRAMLLSESK

SLEDIPSSVIRTFTSIRVLDLGGTSIKTLPDSFGALKHLAFLRLARAPIENLPNSITRLK

KLQIL

>LL_002803_T.1

MDSLCSNSLILITSRNIDVLTRAGVEESFIYKLKGLTPQHSQELFSLYAFRKPYPVPGFY

ELVDKFLIKCDGLPLALKVFGALLFKQKETFWEKTLSQLEVPIEIKQKLKISYDALNENE

QQIFLDIACFLIGEKRDIAIAIWDEHRFQNLESRCLVEVDSDNEIRMHDQLRDMGQEIAL

ERGLWHPAEDIDDLWKQSSVLTKLRGIRMFPWYQREYEESWVMRLRDRLYNTFCPSRAMK

LQFLSSEGYLLGRILRRLHCPNLIWLSWRDCPYSYLPSWIPMKNLRVLKVEGKVFKTLWQ

GRSQAPIQLRALEIHCTVLLEISKSIGQLKHLKHMKLSGCRSLKRLPESFGNLTNLQHME

LSGCESLERLPESFGNLTNLQ

>LL_002836_T.1

MVDPGFVPGFIGVAIQLAVDRILRHITIAVRCTEELSALKDVVIRIEPLIRHIQQYRLEV

NRKRGISPSHAHNNVSAVNVWLEKLNALLLQASQMAQHCTISSSFNIISRYKTSIRIANL

KSDIDKHLESVPLIQLALTHEVLLGQSMQREGHQASASSSASTSMPAIDPTAGFLIEEPL

IVGQEKAFAILHRFVVDAEREGNSLFRTGVVGMGGAGKTLLLKRVFNNQNVRDLFRDGLL

LWLTVSNDPSFINLRNELCLQIAILTKINFDRNRGDEYAKLWLNQRLQTQRFALFLDDVW

RDGPKLMEELGLDLIRRSDSKIIVSSRNRTVLSGIGVSETSTISMGDLSEEDSWHLFAKK

AFPYNSGNLPAKIDERMAKVVCDSCGGLPLAIKVIGRAMASATQPRQWEMEAKRLSVSER

HEEKSQMYDRLRLSYDALGNYSVNLQLCFLYAAAAFLEDQIVHVAFHVIPLWIGEGLLRP

QNDELAGYDPFEMGRIYADLLADRCLIEPTMRDIDGSVLCFRIHDVLRNLGIQIAEREEN

FFCRVGTGLTALPENDCSGRTRIVLNRNELSCLPESYRGPDICSLLMYGNRNVTKIPKQV

IGSMYSLKVLDLSGTSLSSLPNSVGGLKQLVCLRLRRVPIRRLPPSLVNLVNLEILDLFR

SSIMELPSGLHKLRSLRYLAVRECTDLQYLPWNISRLTSLQHLDIYGCSRLWTKLEAKKR

CKKVASIQNLGSLRHLKKLVLENNGEIVEEGTLGNMVQMETLALKLERMASLPQDMANMI

KLKTLCLECPSLIKMESSFSEFQNMSCLILYGCLMLEELPHLYNLRSLRELQVGKCCKIK

KFSKEFGEAGAFPLLEIFSLVGLNELEELPFVYEGAMSSLKIFTMMECEALKMLPESYFT

IKTLQKVRMFGCSMVLENLDRVKIENTKVEVITMSIIDTRESLKRYNEVKDTIEGWVYGE

FWCNE

>LL_002890_T.1

MKEHGTFLHQWENALYQMRNNTETFDSLHSIVDKELFRRLQWSYDFLNSDNLKACFISFA

AFPEDRVISCEEVVDLWVAEGLVKGTRGSYLPDTADSFINHLRNRCLIEVAQTDFVGSIC

SVKIHDILRDLAIRIGEKKHGCYFKAGQGLSHFPLEEVDSERRAKLSLMSNNIQSLPQTF

RFSTLSVLLSSQNRGIKEVPESFLNELPSLRLA

>LL_002906_T.1

MAASTSTSAHLNINKHYCYDVFLNHRGPDVKKTFASHLYHRLVSHGLTVFLDQEELEVGD

KFSCQIKEAIASASVHVAIFSPGYADSNWCLDELVLLFESGAPIIPVFYHVKPTELRCTP

CKDGRYAQAIQKLEMKKTVSSQPRYDSTTVENWRNALSSAAEISGLDLQEKFNGDEGKLL

DMVVECVLKKRKTLPLNVVKYPTGLNEQFKDFEAAGITRCDCALCEMSFEKVEGETEALR

LSIGKIVLNKLEYSIDMSTVLMQLQHLELYKTEKSEVSFPTVVFSNLRHLIIKCCDDLVE

VGTLPNTLIHLELTSCSELKKIEGLCDLAKLKTLDISECKEVEELQNIGTLVSLEVLKTS

KCEKLKTIWGLAQLRRLRILNISECSEIEELPGVENLRLLKELSAYRCEKLKGIQGLGQL

SKLETLNISECSALEELPGVESLRSLEVLRTWRCVKLKSIPTLAQLTKLRTLDVSVCCQL

EELPGVEYLRSLEELSAYGCEELKRIPRLVQLSKLKTLNISECFELEELPSVEHLRSLEV

LRTWRCVKLKSIQALGQLTKLRIIDVSECSDLEELPCVQHLSSLEELSAYGCAKLERIPS

LVHLTKLKTLNISECSELKVLHGVEHLTSLEVLRAWRCVKLKSIQGLGQLTKLRILDVNE

CVELEELSGVEQLNSLQEFSRGGCVKLRSIRR

>LL_002959_T.1

MANLLAPSSNSSGHNSPFEIFINHRGPDTKSTIASHIYNRLHDLHGLRVFLDKEEMEKGD

FLTSQIAAAIRDASVQIAIFSPRYAESEWCLNELLLMLESMESGRSAIIPIFCGVHPADL

RWTLGKDGVYAKALTKLQKKKTIDSQRHRKKPRYESDTIEKWRNALSTVSHISGFELQGA

YNGVEWRLVDEVVQLVLEKVKKPQLDVSKYPTGLDKKVKEFEELLHQQNGGTKVIGIVGL

GGVGKTTLSKEIFNRLRSSYEKSCFLFDVRERSRNSLHSLQSMLIKDLTGSNVQISSADE

GPEKLRRYLSSSQALIILDDVDHIKQLDALLLPAKAVLHTSSLILVTSRNKDVLISSDIP

ESSIYRLTGLNPHHSQELFCSHAFGQPQPVVGFDKVVEKFLDACNGLPLSLKVLGALLHG

NKDLKHWEAQLRKMFKILPEDI

>LL_003303_T.1

MENPTLGALNCAQPPWCPDVLISICIFVALFLALKHLICWILLRSLRASILNNAIECIAS

SLDIQEKRTSSDVHIQYDVFINYHNNEISANRFVDNLFEVLKSCGVRAFVKSRDAETEEI

LDGRTKHAISTAKLHLAVFSKEYAQSPKCLSELYEMVKNGKRVFPLFLDVEPSDARWIRR

SYKEAFERHGRQQRFHIETLKSWKEALHVVSLITGWELAQFKGQEGKLLKKVLQDVLKEV

NDVPLEVAKFPVGLSERVEKLEALLLLNPDGNKATVVGIAGMGGVGKTTLAKATYNRIRS

NFRVSTFLYDVKELIDREGLTAVQEIILGDLLRFECKIRSIDEGKVLLRRRLNKAKNILI

VLDNVDNFEQLDALMVTKVLGPSCTVLVTTRDKRILELAQISMVYEATRLNHDQATELFC

RHAFLSARSNVGFDDLVIKFVEILDSLPLSLETFGSHLYGKTDRKIWETLLGKISRILPR

NIKERLKITIEVLDEEEKSMFLDAACYLVGEDKDTAIRIWDASGWSGWLGFETLEQNCLI

QVDAKNRIKMHDHLRDIGRDIVDQESKNFPGRRSRLWRTTDIIKVLTENSGTEAVRGLSF

VSRSSNLSSGSEAGIPTTWQAESLSQMKDLKLLLLQGTSFDGDFSQLSKNLLWLRWWDFP

YQCIPLNLPMERLVVLDLRRGRVVNLWDEDGCSQLPLNLRELNLTECNQLQRVPEDIGHM

KVLQRIVFKRCSLLTTLPEEFSDLHFLEHLDLTRCRSLRSLPNSFGGLKHLRHLDLSFCS

KLKSLPDSFSQLLLIKYLTFEKCKILNIGPNILGKSSSLEHLDFRGCDNIQALPCNISSQ

RHLKWLYIHCRGLKQLPEDWGELVGLRNLILECPQITQIPDSLGNLIHLEYIDFRSSRLR

HFPESVGRLKFLKHLSIRCHKLSYLPDAIGQLNNLQRLFLMGCKALQYLPPSFENLTQLE

NLDIYDAPKLRIHSGILDGLRSLESLSLYRCKSLDEGCIISLCQKAQALRQLRLRKIEVE

NCLRIPEHGCSNLETLEVYACKNLVRAEICSTTLTEVELKACHQLTTISGFSADMRLTKL

CLRDCQELTEVTNLGDLHCLKTFEISGCLKLFSIEGLHLLKELDVLDISVTHEALQRQNE

WLQKLPSPLQLRMSADSIFQNPSEGLSHVLKSFHRMEKDEAGYGLKFRIPLETEMPCGAI

MICFVSSHIVDDRDEDDRDIMLELYHEHQKRRRPVSWSYTKNRDLIHLHIFSADYFMSRV

TRSGDAIIIRPSPSMNDVPMMLGEGWGHVVRRGEEFEIDYIRNAFLGEIGKQLGKSD

>LL_003628_T.1

MASGLVIQSFAGAVPTELLAIIKDVLKKSLACKESCKRLQKTLEGIIPVLIHDSKSVVEL

SQHSQKLLSELYAEMKEGIRLVQHCSKLSRLNIYKSYKYANKIEGLELYIVNFTQTKGWA

HICSELRSLREQQVQLETIGHNLEDKLESLEHKMEDKLEKIDSKIDAIREESEIKFIADQ

MDQLEIGKRASQSNVPDLPDFRVGLKGLVYELKKRLLHNDAPLLGVIGMGGSGKTTVAIA

LCNDTEVKGHFERIFFERVSQSPNLKGLLERMWDKIVGETRPDFLDTEDAHLQLQQKLRQ

MKPQPILVVLDDVWEIDHLEKLLFEGKGYKTLITTRNNNVVKSDYRYDLPCLREQDAVSL

FCFSAFDQKCIPETADYNLVKEVIAECKGLPLALKVIGRSLHDQPHAAWMTAKDKLSQGQ

TISEYHKTEVLKRMATSIDILEEEVRECFLDMGIFPEARKISADPLLDLGVYVHKLKWTA

AYTILIELASRNLVTLMNNQKCNLGNPYASSGELSLMQHDVLRDLAIYLTEQEPAINERK

RFIMPRKADSIRKMWKKHAECDFNAEVVSFHTGLMQEKDWCQMNFPNAKALILNFSASEY

FLPPFMQSMHKLKVLIIVNHNSKRATLKGISVLSSLTRLKVVRLERVIVPPLQEYCQSWQ

KLEKLSLVLCEGCGNTTGLHMGVDLNFPRLLEFAVDHCSDLRELPTSICNLTTLKRLSVT

NCHDLHKLSDDIGNLSSLQMLKLYACPSLKELPYSICKLRQLQVLDISLCGYLKQLPDQL

GQLSTLKELDMRECSRVKRLPKSASDLKSLVHVICDEKIGHQWSSIKASTIPRLWVEVAE

EHFHLDWLED

>LL_003661_T.1

MPDPGFLSGFIGKGIDIAVDQIIYHANIAIRCRKELNSLRDLVTSLKPIATQIHQYRLEL

NRKRGTSIAKRDNDASAVDGWTRKLHELLQQGVAIVQNCTIPRFHVLSRYQTSRRISGLI

SEIENHLKLAPTVNLLLHVEKQRESDQASSSTSASTSKALAPTATFFIEEDLIVGQKRAF

ATLENLVIDAEPNSLFRIGVLGKGGSGKTLLLKTVFNSQKVRALFHDGLLIWLTVSESPS

FSSLRNDLCKQISVHTNENHDPNMNEEDVKRWLSRRMRAERFALFLDDVWGEGGKLLEEL

GLSRFSQHSNSKIIVSSRNRRALFEMGVEDPSIMAVGDLSKDESWELFALHAFPYNNGNP

PANIDQTRAKLVCDKCGGLPLAIKVTGRTMAGITDAQEWEFAILRLPGTDRDEHQALHDR

LRWSYDALANYDVNLQLCFLYLAAFAEDQVIDVESDLIPLWIGEGLLERNQRGHDPFEMG

TIYANVLADRCLIEPLLKDVDGRVVRCRMHDVLRGLAIQIAEHEENFYCRAGKGLTELVE

TEYSGCTRLFLWGTELSSLPKSNTASEICSLLIAVNNITRIPRKVIRSMISLKVLDLGGT

SVESLPKSVGHLKQLVCLRLRSVPIKRLPAAVTDLASLQILNLSGSGITELPSGIDKLRS

LRYLDLSYCDDLQCLPRSISHLTSLQYLDTLRSSKVWTKHDKKKAASINDLGSLTQLKTL

RLYNNGAET

>LL_003781_T.1

MEPAMTEPVSATYHVFINHRGCDVKKTLASLIYKRLTEAHGLRVFLDQEELNTGDAFPPA

IFGAIQSASVHIAIFSPRYAESRWCLDELCSILDKRNRKIIPVFYDVEPTDLRHIHSGRF

EDAFKQHQSKGRVTVEIIEKWKTALKEASDISGIKFKSSESDHGEVLEEIVKDVLNGVKW

EPLEVAKYLVGLDPAVKEINDEIKKQWELNGTAIVGIAGMAGLGKSTLAAYLYNLKRSDF

SRSCFLSDIRKRDLPSLQRKLLKDLLGCDFDIEDTRHGKGIFRDRLRGLRVLIVLDDANH

IEQINSLLVTDVV

>LL_003941_T.1

MAQLTSAQRDTFASSSSCSASIPCVPYDVFISHRGPDVKKTFAGHLYHRLKSRGLQVFLD

KPELQAGHDISCQIKGAIEVASVHIAIFTENYAESKWCLEELVLMLNSKGTILPVFYNVK

PFELRWTEKGTYAGALQKHEEQQRYDLQTIGEWKKALSHVAEISGFELEACNGDEVELVD

KVVQRVLKEVPKPPLHVAKYKVGLQEKLEDFERTVLSQLEEEGRLEAKVLGILGSGGIGK

STLAKQFFNNKRSNYDVSSFLFDVRENAATKSLNYLQSKLIEDLIGTDIQINSWEEGIGI

LEKNLKSCHALIILDDIDHISQLDALVLPIKHVLDPKSLILVTSRDKHVLQSARVAESSI

YPLKGLNRLRSLELFCWHAFFQRHPLPEFEDLVDGFLNACDGLPLSLEVFGALLCQENDR

SYWEEQLNGLETLPPEIQVRLKVSYDSLNPEEQQMFLDIACFSIGENKDKWIRIWGGSGW

KGSVGLRNLENKCLVEVDGENKIRMHDHLRDMGRNIAQDRSMPRRLWGVTMKDVYDLLEQ

SSPLTEVRGIKMGPQSEVPFAKRFSRKVFKISKLQLLEAEGDLVESILSKVRSPNLNLIW

LCWTSCPCSSLPSWIPMHDLRVLEVSGSKLKRLWRSECQAPLQLRELTIQFSPALELFPN

SLRQLEHLENIEVVDGMALATLPEELCLPQSLKFLKIRYVSIWESRRSYFGRPYLHSCRR

LKILPNSLGNLINLQDIRLPYCIGLQTLPYSFGNLRNLQHVDLQGCESLERLPDSLGNLT

NMEHINISGCKRLETLPYSLGNLTNLQQMDLHGCESL

>LL_004006_T.1

MQVGHDIPSQIKGAIQVASLHVAILSPRYAESEWCLNELLLMIESGKTIIPVFYDVKPSD

LRWTDKAKDGPYARALYAHEKKKRYDPQTIQQWRTALSRVANISGLELEAFNGEEEKLQR

QEELLGKVVQSVLKIVPPPLDVAKYPTGLGEKLEEFESTVLSEHQREHRVGAKVMGIVGL

GGIGKTTLATYFFKLRRSEYKMSSLLFDVRENAAKISLNSLQIKLIKQLKHRDIEIERWQ

EGTEILKNHLSSCHALIILDDVDHINHLDAFLPIKDVLHPNSLILVTSRDQGVLRRAKIV

ESSIYHLRGLTRAHSQQLFCCYAFSQQVPKPGFKNLVEGFIEACHGLPLSLKVLGALLYG

EDEHSYWKGLLDGLNKLLPDDIEKSLRISYESLNEEEKQIFLDITCFCIGDDRDKWIRIW

EGSGWTSFVGFRNLQNKCLVDVDHQNKIRMHDHLRDMGRNIADSSRSMPRRLWQPIYTLK

DINDLLEESPPVITEVRGFRISMHFERHNQSRNHSRSHEVLVMRKLQLLGAQGDFVESIL

TRVESPKLIWLNWHDCPYPSLPSWISMHDLRVLEVQGCKLKKLWESECQVGKPYMIKFKF

KMKIIVS

>LL_004016_T.1

MPGVGKTALLQLIYNDCKKKVSSNFDFVIWSTVSQIFNIQSLQDTIAESVGLDIKGISSF

DTRKMKLYASLENKRFLLILDDVWSPIADLEQVGINFGHDNSSRVLISSRYKDVVETTAA

NEYCMMVQPLSTEEGWELFRGGAFRNGAAPDHNVEAIARDIALECKGLPLAINTVAAAMA

RKRSADEWRRSLDLMRNVDPSFPSTHRTIDVELYQRLRWSYNDLPSYNLKMCFLYCAAFP

EDAWIPVETLVELWTAESLVPQRRATYYMDVGREYIKVLVDRCLIQYDEEKDVIRVHDVL

RDMAIYVGEGEEKCLFSSSQQLRHFPDEEETRDRKRIFLLNNQISHLLRDFRCQTLVSLV

LAQDVYRGFGGLKQIPDRFFENLISLKVLDLSYTAIEALPASVGQLVQLQCLHLRYCICL

KELPDRIGELKHLQFLNLSDCRSLKYLPDRIGELKHLKHLNLEGVELRVNPHQICQLTSL

NRLVLPLLSTPMSLEDLTNLSNLIVLRAKIKSEIKGGSMSSWSEMRELTLFFKEDDADDI

LEDIRLIFSEDDVEDDAALDILPQNMQSMKKLQRLLLLNYRGVSLPNCISNFQNLKELKL

YGCRKLKELPGLEIGSDAESDGFPMLERLRLESLDSLESIVWNEKTMLKLQHLRIHGCRV

LKSFRTEELPNLRELWIGFCGELSNVSQIGRLPMLETLFLYRLEKLESIAHWNDKTMPNL

QNLKVSGCPMLKELPKGLDKLSNLSKIEGELDWWNGIVWENHHTVYSK

>LL_004332_T.1

MAGVSTATGCAANVLSNVVSCLWNTVVEEVKIVIFLPQAIADMKAEVQSLSGAIYNIRED

LDREQRNPKRQVQNWLDSAGKAIESCVTIVNEYQQHKNCLAWCPNCLYRYRIGKHIRDWK

RTTSQLYSEKAQPDFPRIGEYGDPAPRPQILIQNPFVPNAKRSAQTEHVERLLTDNDSNI

RIIGFLGMGGVGKTSLLDTINDSQKVRDSFELIIKVTVSKNHILDLQNCVADKLKLTRFP

DRLNFEERKDMLRSYLENRKFLMLLDDMWADHDMWTSSKLQELGASLNDRGSKIVLTTRD

KEVCVRMNVQETIEVEPLLQEDGWQLFRSRAFENENGNVPQELEEVAREIAKECKGLPLA

ITVIAAAMKEHGTSLDQWQIALDHMKNVPEMFDGLHSIVDKELFQRLQWSYDVLKDSLKT

CFLSFAAFPEDREISCEEVIDIWVAEGIVKGSGNSYLRDTADSFLNHLRNRCLIEVVRRD

EVGRISRAKIHDILRDLAIRIAEKEHGCYFKAGQGVTDFPLEEVDRDHCAKMSLMSNNIQ

RLPLTFKCSSLSVLLLSENPDIKEVPESFLKELPSLRILDLSRTGITCLPPSIGNLKRLA

FLQLQQTGICELPETIGNLKDLQFLNVNSCRKLRCLPERIIELKRLRVLDTAFCDQLRYL

PRGISELVALERLNTVTSISLSFEDLQSLRRLRVLSVTPKSPVKEGVMGNWSKMRALTLC

YDDDVNQDELPQDMQAMKDLERFHLARSDVERLPSCLTQFRKLAYLWLHGCKQLKELPAE

LPCLRELLIWYCDKLEELELGMGFPKLETVDLFELKSLECVGAGEGAAASG

>LL_004508_T.1

MKKPLHVAKYPAGLDDKVKDFENEVLLQQRQSEKPPVLGIVGLGGVGKTTLAKELFNRES

SNYSRSCFLSDVRECVNKGNLISLQRKLLKHLTGSELEEDSIDENIETLKKHLSSSQIFV

ILDDVDHVDQVDSLLPDQSVVRSDSLIIITSRNKGILTSSGVENSSIYKLAGLNEQHSLE

LFCSHAFMQPYPVSQFEPFVDKFLKACQGLPLSLKVFGALLCGKKNPSYWQDQLDKLQRI

LPSDIKQRLQISFEALDVEEREIFLDVACFFIGEDRDMAVRIWDGSPWKGFQNLEDRCLV

EVDSENVIHMHDHLRDLGREIAKAPGLVCGTDNIDDLLQQPSLVSAQSFKSI

>LL_004790_T.1

MAASTSTSTSTSTSKAMAASISTSTDVYDVFINHRGPDLKNTLASHLYYRLNVHNLRVFL

DRPELRKGENFESQIKGAIASASVHVAIFSPTYAKSKWCLDELHLMFESGSPIIPVFHQV

KPDYLRWAHEQNEGPYAEALRYHKNKRQWDDKPRYDSTTVERWRNALSRAAEISGFELDA

FNGDEGVLVDKVVECVLKMVKKPELHVAKHPTGLEEKVKDFERTVLPQRNSGRAQVVGIA

GMGGVGKTTLAKELFNRKRSNYSKSYFLSDVRENVSKTSLHALQSKVLTGLVQLNVRIDS

KDEGIGMLEKHLKSLQVLLVLDDVDHVDQVDALLPVDVLHRDSFILITSRNTNALASSGA

QELPIYQLSGLNRDHSTELFCSHAFHQSHPQSGFESLVDQFVGAGHGLPLFLKVFGRLLR

RRRDQSYWEEQWETLQQTLHKDIQESLQISYNALDDNEKQIFLDIACFFIGENRDMATKI

WDGSGWRGSLGRFKTLQEMCLVELDIDNTIKMHDLLRDLGRDLAKHPELPRRLWRGIEDI

DGLMQHRYQKTDVRGIKVSIAEYPSDAFLIQEMILNNEYEDQHTDEEDEDEENDDEEDED

EENDKKKNDRRKKFLKPKLQKLTQLNKVNATNLQLLDVDILSEHILRGLQSPNLIWLRWK

ECRLPSLPDWIPLKNLRVLEVHGKEMKTLWHDRSQAPKQLRELRIFAPLSNIPKSIGKLK

HLERIEICTKYDGDGHQIEELPEEFGRLLSLKTLVLKYQPQLRSLPDSLGNLTNLEHIDL

SHCGKLERLPNSFRKLTRLKYLGLSYCRNLTVSSDTLGNISTLEYINLNYCGNIKVLPSQ

VAHQRYLKKLELTLEELKELPGDIGGLSALEELLIKSHWLETLPPSIGGLSNLKHLELDC

LRLKELPSDIGKLSALEKLAIKDTA

>LL_004867_T.1

MAYPTPSCSSFPQTHSQIEMENQTSRPATSVGASSSTAADPNILYDVFITHRGEVKKTIA

SHLYHGLLSRGLRPFLDQQELQEGLHFPSQLKAAIRSAWVHVAIFSPKYAESQWCLDELL

LMQESGAPIIPIFYHQNKNGQIRTMPAELKWTDQNKNGVYAKALHELQMKKTYYPQTHQD

KSRYDSATIQKWRDALSSVAHISGFELDGQFNGDEGHMVHKIVERVMKMSKKPGLYVAKH

PTGLNEKVTDFETRVVCGQAAAGKWKCSGGRHSRLGWSRED

>LL_004915_T.1

MSMDPVSQSIIGTAVDELFVVTRIVMNIMIFSKSSSKELRQKLLGLKPTIDKISRVSSVS

DPSSQRGQPFKDLQAQLQDGLHLVNKLERMSSFNLYRRYRYGKKILKLEKNINDVLLTQG

LAGLLVDVQKLNAESEGCNERLERIEEMGRHIIDSVNAKMTNDASSNSIMLHQMSSTHLF

EPSIDESNDTIMAEQSTSSSSSQVPDMPNFVVGLNTLVNEVKQILIYQKEVNIVGIKGMG

GSGKTTLALAVCSDTQVKDFFHNNIIFITVAQSPNVKSLLEIMWDKIIGGRRPVFQSIED

AHNQIQNKLRLKGNPPALVVLDDVWSKSNIEHLMFEAEGYKTIITTRQDYTIPISNKTYV

YNIPMLQKVDALSLFCFWAFGRPTIPTTEDEDLVKMVEAACKGLPLALKVIGSSLRGEPQ

PVWENANKKLSRAESISEYHRDNLLHCLETSVDVLDDESKQCFLDLGAFPKGRKFSVDSL

LEIWVYVRGMEWQDAFVVLLELASRNLLNLTSDPGSRAISFGCASELSFSQHDVMRDLAL

RLANRDSTIQRLFMPTKEDTIPTKWLTLKDQTSKAQFVSIHTGPMEEKNWCQIDFPEVEA

LALFFAASQYCLPTFLRTMPKLKVVIIYNYSSKRATLHGLPSLPSFTQIKSVLVERLIVS

PLCEYCRYSGSLEKLTVCLCEGLGNMTVLDKEQALKFPNFLEINFDHCSDLEELPGNICN

LTNLQKLSVTNCHLIQKLPDDLGNLRSLRVLRLSACPSLSMLPPSICELQQLEFLDISLC

RSLKDLPMEFEQLSKLKVLDMRECSGLKKLPKALAKLKSLGRVICDENAGRQWLAIKASA

MPNLTVEVVEERFNLDWLDD

>LL_005002_T.1

MDAIWTGAVANTISSVVCAVIAPLVQVVDDIIHLERNTLSLQENLGTAKVFLEGTDTQFH

HQQRSIPPQLNQKLEELRHSVDDAESVISSSQQRQGCLDRRFCNPRLCRQIRKCNMVLQE

RQNNLRDTIPTVRGIQEIVSSAPLRPEAQLQPVPQSGYVGTGIKSAEMQLNTWLEADDHQ

FRVTGIYGMGGIGKTSLLKAVYNTYKQLKGNVFQVVIWLTVSQKFNILDLQSSIAEAINL

QLGSTSNAQSSTSNALETRKMKLFARLQEMKFLIILDDIWDSIQLEELGIPVGKEKGSKV

VISTRSKDVIRIMGTNDYSIQIQPLSPIEGWQLFCRVGFKAGGAPTKSIEDIARQIAEEC

KGLPLALNVVAAALAGNTTAEEWHLALIQMQNVDPGFLTMYSGIDEDLFGRLKWSYDRLN

DCNLQNCFLFCGAFPEDAEIEVETLVEMWIGEGLVHSRDKSQLMYAGRRYVTLLEERCLF

QYVKTDMKQIKVHDIVRDLALYIAEKEEKCLFRTSQKLDEFPAEEVMDDCKRIAIRYYND

DTDAFLPTDFRCPKLGTLILAQNQNLREVPNGFFLNLTSLRVLDLSHTNITSLPESLWQL

KQLQFLGLNDTKINSLPADICNLSRLQFLHLGGCEQLESLPRTMAELKNLKHLVLNGCRS

LAGIPREICQLTSLNKLEYLWMNPSKLGVDEAESGMCSLKDFTNFPNLMELHVCVKPGMI

DGITRGGIQEGTMATWLEMRHLLLEFDLHMEGLEGIEEELVEKMVPDLPRDMQNMKKLQT

FHLWWYGGISLPNCICHFERLEELSLRSCFQLEELPPLERLPNLRELELTRCIKVRKLGI

TGSVGGFPMLERLHIEDMKNLQSISSSMPKLQTFRITDCPALNTFAKEELEEIVHRNREA

LQSKRDRHSGRCMRQINLGGY

>LL_005309_T.1

MNRVNCKNSCSRLDALLTGIIPTLQQICLLELVKPKHEQNPIYEQFLSILMKGVELVKKC

EKSSPFKIFHKFRYASQIQQLEKEICDFLQYQMPANMFLDVKNLVTELKTFRHLYDDRKM

NETISKLTNDPRENAVMLEQRSADDMFNDEAPGNYDENLVKSDFVLGLENNIWNLKRILL

QREVSVVGMHGMGGLGKTTMALALSNDPEIKGIFQNNIIFVTVSQSPNLNGILRTMWEKI

VYGKSPKFQNVEDAHRQLQQQILRKSKPTLVVLDDVWSKSNLENLLFKGEGYKTLVTTRD

LSIIPQTTYTQVYELPLLDDNNALSLFCFWSFGQRSIPSTADKQLVKKVQMECKGLPLAL

KVIGSSLHGEPYFMWENAKNKLLNGELVSNYHKEGLLRCLETSIDVLDEEARDCFLDLGS

FPEDKKISVDALLDIWVYVRKIEWKDAFVILLELASRNLLNLTSNLRPNRTHAINYGSSY

ELCFSQHDIMRDLAIYLASRDNIVHRKRLLMPRKDHNLPKKWEMLKDKKCNAQIVSIHTG

VMEENQWCEMNFHEVEALVLLFSASEYFLPSFLSSMRKLKVLIVFNYGSKRSTLNGLQAL

SSLTHLKSIHLEKLNVHPLQKNSSILQNLDRISLSLCAGLGNMSRFNNTWTSLKLPKMLD

FNLDHCCDLEELPPKICGMNSIQKWSITNCHLLQKLPDDLGKLISLRVLVLSSCLALKEI

PVSIGQLGKLEYLDISLCQCLKELPEEIGNLRKLKELDMRECSQLRKLPKSIGGLISLKH

VICDEKIGQQWMHAKSFTLIDLRVEV

>LL_005598_T.1

MAASSSSAVHNPYDVFISHRGPDTKKTLASHLYRGLSKNGLRPFLDQPELEQGELFPSQI

ENAVRTASIHVAVFSPRYAESSWCLNELLLMLESGKTIIPVFYHVAPAELRWTRGKAGRY

GEALQKLAEKTTCDPQSGKLESRYQSVTIENWGKALSRVADISGFELEACNGDEGELVDN

VIRCVVKKMKKPLHVAKYPAGLDDKVKDFENEVLLQQRQSENPPVLGIVGLGGVGKTTLA

KELFNRKSSNYSRSCFLSDVRECENKGNLISLQKKLLKDLTGSDLEIGSIDENIETLKKH

LSSSKIFVILDDVDKVDQVDSLLPDQSLVRSDSLIIITSRNKDILTSSGVENSSIYKLAG

LNEQHSLELFCSHAFMQPYPLSQFEPFVDKFLKACQGLPLSLKVFGALLCGKKNPSYWQD

QLDKLQRIIPSEIKQRLQISFDALDVEEREIFLDVACFFIGEDRDMAVRIWDGSHWKGFQ

NLEDRCLVEVDSKNVIHMHDHLRDLGREIAKAPGLVCGTDNIDDLLQQPSHVITKLRGIR

TVHTYGRSQMSWHRDILQIRASEAGLLERILTRVHSPNLIWLRWDECRYSSLPSWIPVKN

LRVLEVSGTSFLEGEWQAPLQLRDLRISRLLGNLPNSIGHLTYLERISLSSFIQHEQFMR

FQSLKTLVLPKCTKMKSLPDSFGNLSNLESIDLSSCYYLETLPKSFGNLTKLKYLKLYSC

YKLTMSRDTLGNISTLEHVSLAMCAKIEVLPMQLAHQRSLEKLDLSGTNIKELPNAIGEL

SNLEVLVVGSRSLDTLPPSLGALKNLKTLELLHCQELKWLPASVGLLTQLTELAVWNCPI

SELPFKKVEGETATLRTSSNLDSSTEKCMPRLQLLDLHNTEISDVSFVEGVCRNLEYLHI

SNSKDLVKVGTLPKTLVKLDLHSCWNLRKIEGLSGLARLKTLDIRECKEVEELPGIETLV

ALESLLANGCAKLKSIQRLVQLTKLRELHIGGCSELEELPGIEHLRSLENFEAQDCVKLK

NIRGLAQWTKLRELRVGGCSVLEELPGVEHLRSLRVLQASKCVKLKSIQGLGQLTKLQDL

DVSECCELEDLEGVEHLRSLRVLQASKCVKLKSIQGLAQLTNLQVLNVSDCSELEDLEGV

ENSRSLRELQASKCVKLKSIQGLGQLTKLQDLDVSECCELEDLEGVEHLRSLQYLQASKC

VKLKTIKGLEQSAKLWSLNVSECSELEDLEGVEHLRSLQASKCVKLKTIKGLEQSTKLSG

LNVSECSELEDLEGVEHLRSLFYLEASKCVKLKSIKGLAQLTQLWKLDVSESSKLEGLEG

IEHCMSLRALNASGCPKLQWGGGVVEQLHERLKEKLIL

>LL_006471_T.1

MKSFDLIIWVRVCKDMLCSYLKKKVFLLILDDMWMSLELQSLGVSLKERGSKLVLTTQEI

ARQGVQGFATGHKRI

>LL_006716_T.1

MANLTISHGVAAASSSSKASSSASTSYSQWEVFINHRGPDVKNNFASHLYRRLISSGLRV

FLDREELEEGQNISSQIQQAISGASVHIAIFSPRYAQSAWCLDELVLMIKSGATILPVFY

KVQPSVVRRTFNDGAYAEALRFLESKTTHEADEERPRYGSHTIQNWRNALSYVADISGFV

LNGDEDDEGELLDKVVQRVLEKVPKTPLDVAKYATGLAGKLEDFEKTVLSQQQDEKRVEA

KVVGIVGPGGVGKTTLAKEFFNLRRSEYNGSSFLFEVRENTRTKSVNYLQSKLIKDLKHR

DVNIESRDEGTGILRKHLSFCHALIVLDDIDYVSQFEALLPMRDILSPDSLILVTSRDQS

VLRSSGVLESSIYKLEGLSRLHSEELFCSYAFHQPHPPPEFADLTDRFVRACDGLPLSLK

VFGALVCEQNESYWEDILNELIHNIHPTTEIQDRLKISYDSLSSRDQQIFLDITCFFLGE

SRDTAKRIWGTVGLQNLQNKCLLEVDGENCIKMHDHIRDMGRNISHGVEMPRRLGHATTN

DIDDLLEHSSCVITKVHGISMLGVNHTEFPPMSSEMPWYKIWIDEVFGNCLRHLVNDSRF

SGILNLEIVAVEDGYYLERILKKVR

>LL_007277_T.1

MEPVVVPATYQVFINHRGPDTKKKLASLIYHRLISCGLRVFLDKDELRTGDTVCPAIRRA

IRSSSIHICIFSERYAESRPCLAELCWILKSSHERAIIPVFCDVEPHDLRDINQGPFAVA

FQEHQRKGKVAVAVMNDWKEALRKTAGISGLAFRTNESEHGEFLEEIVKIVLREIKWDPL

EVATYPVGLDQAIEDFQNKIDLKQSATKVVGIVGIGGIGKSTLAKHLFNLKRSDFNRSSY

LSRVREKIDLPSLQRQLFRDLLGVDMHIVDTDFGRSIARNSLGGLKALIVLDDVDDGSKL

KSLLHLDGVGHGSLILITSRDKDILNGSCANPLLYEVKPMGRKHAQELFCRHAFHQPKPF

EGYEDLVEQFLEILGGLPLSLKVLGEQLAGKLDKTHWKRQLEKYFQGLPLPEAKDVINTL

KWSYNAMRMEEKMMFMDIGCFLEGEDTELAVRVLEGLGYYNDVRDCLQTLRQKCLVDVDN

NEIDSSSEGRRNVTGFTYRNMYDSIFFRLRYYLATELHGKTKIRMENQVRDLVRHIVREE

LTSMEKPLRLSCSTDIAKMLELQDGSDFCRIRGIRIPKGQDLPKLNENLNIKGLRLLVVE

HPIDLSRFFCSAIFGDLIWLRLHNCGFMSIPSTISLRSLRVLELQGPRGNIEQIFNRIDE

IPWELSELSVDNRLITSASTSSSSVLPVDFEHPNTLSRREISTSSEKNISKIQKQNISRT

QQRIMPTFISSTKQSAMPSVISSTQQQGEMPSFFRFLQWIGTFLTNLGKIELKNINSLRT

LPIDFSALKILRHLDLSGCIYLTKLPNSFSHLLQLQYLALRNCKNLSIPKDILGEISTLE

YVDFQGCAQLVQLPLGMAHQTSLRYLNLLDTPLLQLPENLKLLDKLEQLSIGSASLTELP

LSVAYLVHLKELILIDCLNLMCISAPIEKLTCLERLEIYESKVPILPARIAMLKHLKVLT

IYHCPIAKFDFSVNNSSMDALRDFTLHTTSISKIAIPNSACPRLEIVDLSCNRNLMQVQG

FPSTVVRLNLEGCSRLKTLTNISNLAHLQFLTINGCVELETLNLEGLRSLEEIRAEECWK

LQNIKALSQLERVNCLQISTQIGTISNSLILDKALQSGVAALTVGVLDATKTIASPLLLR

EVHRRLPILMFFITTNRGRSNSFSVRFKPSNSRGQSEVYETVRGDGSGGRMLHMYMWTED

SKLLKDDNFYDEIVGYHNNYGYDGTETGNNLVAGDVDDGWVVAYSSETKVAQVCNQILRL

CWGLEQ

>LL_007826_T.1

MGNKTRRFKLPHFRRFKLPHFRHGSQPVVDPDLPSSSQPNTSPLAFTAHDNVSEAGEPPS

SNAATIINNVTDDQLQGMPSSSSDSKASDLTNNILAAAHAVDTKDVDAEVDRLIEYIIDL

LEAKKTVKKPSNTEGAKHSWHSSSESLKKFREVLSEAINVFKDVFPEAKTVTQGLLKGMG

TAHLTTTGFLIAAHVLERFETVSSNRDNCMLVLDEICNVAQHVKRLRQWDGLRKEMEHSI

KEAMGLIVEASIICSTQIGSSKRRRFWTASGTKEKLEHLQQSLQKMDVHMARDVAIGLYD

AIDSMKARPQKPRERPYPDYAVGIQEPLEEVLELLEWDSEENAVAVIVHAFGGMGKTTLA

DALFARHNREGCKYSMVRLFHDVTSTPNIIELQKYIIEDLKMGMEMPNIRTFEDGQGEIG

RILQEEEAFIYIDNVVDRRHFEQLLPKYMDKAKKLRLLITTRDSDVCRGFRKLKSKIYPM

KSLPHIEARRLLEKEMYNDTHNMENQLSSSQLDEIVKMCGYIPKLLTVVAGFISCADDKQ

KAYSRLKEEREKNWKGEIGDDIAHYIFAYQTLDERLKDPFLDICSFFNGSRWSKVADIVG

DGELNSLEKRALVTKESTDIVGDGELNSDFPSVWVHDVILAMGQQQAHGTRFTSANEIQK

FLNEKEDEDIRKIKGIMLPYKMQPVKGIMLPSLYISASKLDLASSSLRVLQLQSYAKVEG

KCKKTFQKLVFLEASISEIPFDVSMSNELKYLWWMPGDLDQELTLSRRKYVEELLGSISK

LQYLRVFELHQCKNLRQLSKDFGALNSLQKLHFYRCSNLKALPSSLGNLSSLQVLSLYMC

SSLEALPSSLGNLSSLQVLSLHECSSLEALPSSLGNLSSLQELRLYECSSLEALPSSLGN

LSSLQVLNLRSCNRLKALPSSLGKLSSLRSLFLHDCKSLKFLPYDLGKLSSLRDLELFGC

KELSSIPESVGQLKSLKFSISLSNTGLKELPGGFCNLSFITHLYMEYCDCLEKLPGRFGE

VTGLREIKLRYCTRLKKLPESFGQLNNLERLDLSECTMLEELCIGFSCLSSLQRLYLNGC

KMLKKLPEDFHRLSSVKSLDLRDCHMLEGKWMESMVNMKTLQIVEITGSPMLEERWEEIQ

RQGDHSWSFAVFTGKGSSNKESKEHILKQLHSKIDSILTNGRLSDLPLNTLLLVMFNRWS

CNNPGFAPSLIEENIDDIPTNFKMIYFGNCFNELPKKMADRILGHSPANSNAQALFQKVL

AAFGLKQNSRSFEMIAKIVKDKKGDKYLSCWELLSEKSVEELVMKKCGRYHKLKQLVEKP

EESNLELLRELFASGEEESTLGFLKNDASEVRVEELEGKTILLHIRPSNSRCEILPLQSL

MEMYVAEKERLDFEIITIPIMRKGTTGTTLVFEEILRQVPWLVLQNPWELKSIIKFFLVE

DCAAWLNENNCPGRFNENKLAWDRWQPGIMRVIESNGRIAAHNPALLRMVNTWQAKAYPF

TEEKRKELEEEERNQIKAVSNLEFLFRDQVKEVMFKGKMICFYTSKAGMAGFIRNALIEL

RDNIHVIYIPQFKRSIRPHVNFTSNIRTCEFLLEKPEIPQIEMQEMSVLNLSEHQAMAFW

ARISSLKECINEMDDNDKHLDGIKKLLDFIDQVYSSMTRNFHYSSGYSCLGIMDEDGKLV

TILGKEILKSPKDKIKELVQQLIKCPKECRKEILVELEQLSL

>LL_008375_T.1

MVSLLPFNGGDHIVYCMDMFNCKKSCSRLEGLLAGSIETVQALYSADLGKPYKEQNPIYE

QFLNILNNGVALVNKCEKTSRFNIFQNLRYASQILQLEREILDFAKYWMPVQLSLDVKHL

TTELKSLRQRYELGEAERKVKETIVPKLANDSNKNAIMLQQMDADNMFDETPPCNYKGSV

KSEYVVGLEKNIWNLKRILLEREVSVVGVQGMGGVGKTTMALALSDDHEIKGFFRNNIIV

ITVSESPNLKEILESMWEKIVRRKRPEFQSVEDAHRQLQEQLRRQAKPTLVVLDDVWSRA

SLEKLLFEGENFKTLVTTRIRSTIPINTSYPDL

>LL_008404_T.1

MAGLSDIGTGAAANVLTSLLKKVVQEVKMVISLPQSFTDMEGEVGRLNNMRNDINVELAR

VQMNPKSVVKAWLDKADEAIKSSETIGSDYQQRKNCLSCCPNCLSRYRISKHIRDWKGDV

AQLHLQRQSDFPPSGEYGDYIPPAQIPIEIAESGFVGNAIQSMQSKVEEWLTEDSNIRII

GIFGMGGVGKTSLLQTINNSLMVRNSFDVIIWVTVSKDYDIFNLQDCIANRLKLQNFPDK

SSLEERKHMLSSDLKNKKFLLLLDDLWESFELQSLGVSLNDRGSKIVLSTRSKVVCTEMR

VDEKIGVEPLLEDDGWELFCSRAFRNGSVPQEIEEIARKIARECKGLPLAILVVAAAMAH

YTYPHEWELALHQMQNVDSTFYDIHSRIDRELFQRLEWSYNYLPENLKTAFLHFAAYPED

GEIYCEGVIDIWIAEGVVKGTVESYLPDTAQSFISFLRDRCLIEVVSYDVVGRIEYVKIH

DVLRDLAIHIAEKVHSCYFKAGNGRSYFPSEEVDSEQCAKMSLISNNLQSLPPIFACPSL

SVLLLSRNRGLKEVP

>LL_008456_T.1

MAFPSSSSASSSAPTSYPYYDVFINHRGPDVKNTLATHLYRRLLDHRLRVFLDKPELVEG

RNITSQIEAAIQVASVHIAIFSPNYAQSKWCLDELHLMVKSKGTILPVFFNVKASELRWT

GKGKPGSYAEALSLAEQKKTVDSRTGEEKPRYDIETIQNWRNALSHAADISGFELDGDYE

ELLNKVVGSVLKNVPKTPLYVAKYPTGLEDKLQDFIKTVLLPQQEEGRVEAKVMGIVGVG

GVGKTTLAKEFYNRKSSYYSGSSFLPDVRENAAGRSLNLLQSQLISDLKHTDIKIESLDE

GIGMLKRYLSYCNALIIVDDVDHASQLDAILPIKEILHPSSLILVTSRDKHVLRNAGILE

PSMYKLEGLNSQYSQELFCSYAFCQPHPLPEFVDLVVRYINACDGLPLSLKVFGALVCGE

NDARYWEEQLDRLQHTLPDEIHKKLKISYDSLSKEDQPIFLDIACFFIGEDRDMAISIWG

LVGLRNMENKCLLEVDIENKIKMHDHVRDLGRNIAEEDSMPRRLWQQTTNNSNIDDLLER

SSCAITQVRGIKMVSTLDMRHSLGLGMRSYMSRYKIWMDEVFGNCLRHLFSIFQFRGMRN

LHIVATEDVHLERILARVGSPHLIWLCWYYCPYPSLPSWIPMEKLRVLEVGGSQLKILWQ

HKSQAPLQLRELKIWAPLSDIPKSIGQLRHLEKIQIFTPLPEPLTLKTLPEEFCHLDSLK

YLELLRCSTMRSLPDSFGKLTNLQHIDLSYSHSLEMLPNSFGNLTRLKHLSLRDCPNLTL

SNGTLGRITTLEYLDLDSCSNVKELPPQVAHQRSLKNLNLRGTNLKELPSDIGELCNLEC

LTLGSDLLEVLPPSFGYLRSLNDLSLWELPELNCLPESFASLTQLTQLSLWGLSNLKCLP

ESFGLLTQLTRLIVEDCGLECLPQDLLKMNNLQILDLSDCPLQEHPFIKVEGERETGALD

KCMSELQQLQLSMTEIREVFFSEGVCPNLHELSLSFCKELREIGGLCSLSKLRKLDIQGC

KQLRELDIHGCKQLEELSSLETLVSLEVINATWCETLKSIQGLGRLTKLRTLKVDYCKAI

QQLPGVEHLMSLEQLNACNCPKLHWGQGLVEQLRQQLKGKIPM

>LL_008484_T.1

MGKRVCLKCGGLPIAIKVVGRAMAGSTLPKQWESALQRLPKANSVYDCLQLSYDALGNED

VSMQLCFLYLASACLESQIIYTEKLFPLWVGEGLLARKMLQYENPLSYDPFEIGRIYINV

LADRCLIEPIIRDFEGQVIMFRIHDMLQDLAIRVAEEEEGVYRRVGRYLTTLNENDLSEH

TRIFLSINNLSSLPMSLGAPQIRSLLMMGNKDLTEIPKRVIGSMISLKVLNLNRTSLKSL

PDSVGYLKQLVYLDLIGTPIKKLPASFTNLVNLEILHLARSSITELPYGLHKLASLKYLD

LRHCKDLQYLPCSISKLTSLQYLYLNGCNRLWTKVPHGSRRKKVAFIDDLASLKQLKRLQ

LQNNGKIISKGTLGTMVEMDTLLLTLEEIENLPDGLSSMSKLRRLSLECSHLVKLGSNFC

DLQNMSYLRLYGCLKLEELSHLHILKSLRKLEIISCYMLQKLPKEFGGRGAFPLLEIFSL

VFLSKLEELPILEEGAMPSLRIFSIMSCPHLEIIPESYLNLKTLQKIRIYNNSVFMENLE

IHKINMKVKVVTMSSEDTEKALKAVVDSVQKVIAGGYSMYSADTWGSEFSVFCNGLFMGF

SGLINFG

>LL_008581_T.1

MTDNSLASPSRSLSYEMNPPSVPSNSGDSPSDDQPVSSQRISSQGADLSLEEQAAPSERR

VSASASNNRIHGRESLTEMANKIWRYIWSCFLPLPKEEAVNNIFKDHSENDTEALVNDIF

QLLDRIRQGEPTTSYECRWPDHLSPLLDLLLSGRKEMTSDNRLKDFTDAMSEIESLLEKI

GSASFVTAGFSVVAYGLKRFQDVSDNKEKCLSILEKMNNLNKLVRQCTERDVLKEGMQQQ

IKSATTLIMKGSIMCLHQIRSSPFYKIIKTENNKGDLHRISDELKYQHRQINIQIGICIL

EVIECKGKTLLSREYPVNAVGIEKPLKEVTDLLEWASAKNAVAVIMYGFGGMGKTTLADA

VFNRLNIKEVEGKDCKYSHVESSRDVINLQKKILEDLMGGETIPEIRKYQDGQREIGKAL

EKVAAFIYIDNVLEKDKLRQLLPLNFDKAKKVRLLITARDINVRKACRMSTPPKEYLMRG

ISSKYAKRLLEKEMPSNKKGLLNSNQFNRIIDKCGGIPLMLNLVVQELSSSKHNKEVCDV

IEELEKLEGEEFGVKLEYCFFIYDKLPKECKEPFLDICSFFQGWDWGLVADIVGKAALES

LERRALVAKGPNGIVTVHDVILEVGRRMAKAMRVRFAERSELQNFLQQDEKVEQLRYLTY

KPAKDLNQLIKNNLKLPQNLKYMEIDGKLHCDALEFFPASLLRLPDLRILRLINFEGLKE

LPSVLGHLVKGLRELILSNCNSFKELPYSFSKLTSLRVLKMDHCQSLTQLPKDFGKFNSL

EIVSINHCDSLGMLPDNFEEHSSLTELNLSHCKVLKHLPVGLGKMTSLVTLDVSHCKSLV

GIPESIGKLKSLLSMDISRCSSLVKLPNEFCSLSIRSLKLTGCAALKELPNGFGQLESLR

VLKLKSCTHLEKLPEGFSKLRYLVHLDLSGCSELQELCKNFSGLLSLRTLTLDACKKLSK

LPENLDRLDSLQHLNLSNCELLEGDTMDNVVKAKVLEMVYIRNSDKLKEQWRKIQGMPDQ

LWPFNVDIGQELRQEEKESEWEKLSSAFGRLLQNRQGNPYHISKWSPNTILLVLFDGSPD

FKQEFPWPLIEETIDDGRINFEILYIGKHFNKLPKTVAGRISGRALDNTDARLLLEKVFF

TLKRNGENLWHQNMNKFFSMSTKIMGKRDREASSVKENGDNYLSCWTLLSDCQMEEFVLD

NCERFLWLKQLAEKKQENSIRFLTELFVNMGEDYCLLRKSDKVGLDELRRKTILLEIRPA

NAIYDSRLQSLENLYKKEKEKEKERSNLEIISIPIGQEKERSNLEIISIPIGHQSIKFEQ

FSGNIPWLVLQNEWTITTAAEKYFLSKALHWEDTDGKLYPSRFMKIEPNKEVADIPVLSM

LERWGAEVYPFSDEKIKELREKEWNERKSMSSLKFILKPLMGELDQMKEVMLNRKMTCLV

CDGNHPRTNRLRTGEFEKCISRAMKEVKNSIQFIYVHPPHKKTETERLTVTDTVKVPYFP

LEDSKKKELKLPYSLIVPPWAAMRLRMRVRDLREEINAMDENDEQLRGIKKLLDSLDTGY

SSMAIMDEDGEVFTTQGIQIADIFEDRKEKAKGDAKELVKRQRQEKYRERVRGMINLMIT

KGSKEERKEASLELLELLESQSPDLDVLEELS

>LL_008928_T.1

MAHANRTPAPDAPSSSTASTSNVDNDSYVFDVFINHRGPDVKTTFATRLYESLCKHDLRV

FLDKPELQRGGNTTSQIKSAIRTASVHVAIFSSRYAESRYCLEELAMMVDCMSKSKSTII

PVFYDVEPSELRRTRDKNGVYGQALFKHEEEKTFNSSTIEEWRNALFEASKIQGFDRKTY

YR

>LL_009224_T.1

MLEWKTPIIPVFYHMRPDEIRWTRGEGVYARSLHNLEKKRTYDSQPRYNSKTIENWRNAL

SRVADISGLDLEAFNGDEGELLDKVVEQVMKKSKKTQLNVARYPTGLDEKVKDFETKTLL

QQQSGRVQIVGITGLGGVGKSTLAKELFNRKSANYSKSYFLFDVRENAKISLLSLQRKVL

KGLTQSDLQIDSIDEGIEMLNKHFTLLSSPVLLVLDDVDHVEQADALLPVAVKDVLSPNS

LILITSRDKNVLVRSGIQESSIYKLTGLNTEHSRQLFCSHAFCQPHPLSGFEHLVDNFLR

ACDRLPLSLKVFGALLSGNNDKSYWEDELERLEQILPDDIQKRLQICFDALQRDEQQIFL

DIACFFIGENSYTAIRIWDGSGWKGSRGFQSLQNKCLAELDNENKIRMHDHLRDLAHYCP

VVFGVGQRTLTILLQHSPLITVRGVRILAGEYKGDDGECASLSSYRWRELQLLDIEDCHL

EPILRRVQSPNLLWLRWDNFPYSCLPSWIPMKNLRVLKVDGFGLESLWSDELEAPLQLRE

LEIYGPLSYLPESIGKLKHLERIVVTCGDFLKLQGEFCHR

>LL_009225_T.1

MAMAASASASTSSAADSNNHYCYDIFINHRGPDVKKTFASHLYRRLISYGLRVFLNYEEL

QAGEGLTSQIQGAIRNASIHVAIFSPTYAESNCCLDGLLLMLEWKAPILPVFYHMRPDEI

RWTRGEGVYVRSLHNLEKKRTYDSQPRYNSTTIENWRNALSRVADISGLDLEAFNGDEGE

LLDKVVEQVMKKSKKTQLNVARYPTGLDEKVKDFETKTLLQQQSGRVQIVGITGLGGVGK

STLAKELFNRKSANYSKSYFLFDVRENAKISLLSL

>LL_009756_T.1

MAASSSTSAAANPINKSYEIFINHRGVDVKETFARSLYQRLMERGFMAFLDKGEMQPGYD

FTRQIEEAIATATVHVAIFSPRYAESDWCLKELLMMLETGAPIIPVFYRVSPAQVRWQGN

DGSYAQALDNRANKTTREGTMRYESEVIAKWRNALFQVANNSGFELETRDGEELQLELLD

NLVKHLSERVNKLKSLHVAKYPTGLEEKFTEFEESVLL

>LL_009757_T.1

MLKKPLKNCNALVILDDVDHVDQVNALFPVQTHDLHSGSLILITSRDQDVLRKSRLDKSS

IYTLTVLDTQHSRELFCSHAFSQ

>LL_009765_T.1

MENVEAIGVLGTCGSGKTLYLSTLFNSQEVRDHFRDGLLLWLTVPSITTLPDDIWEQMAN

DLCKQIATKKKIDLTKDIKGKVKIWLYEQLKQSRFALFLDNVCEWNATDLLEKLLKAWDI

MGAVSVSSGSKVIVSSRDRTALSTMGVSDDYTIAVEDFWSFRRKKKLIEEPEMVGQEVAF

TKLESPLQIDDAKVGQETALKNLEDFVIEDAKVEPEMVGQETALKNLEDFVIEDAEVNIN

IGVAGKRGAGKSLLLKKVFNGQKVRNKYSNGFLLWLSLSQNPSVTSLVDDLCKQIAVQKK

IKLNQAMKEEEKKKWLKEQLQQSSRFALFLDDVLFLDGVWGRDARRLFEELGIVGAVGGH

MTKSKVIVRSRDLSALSEMGVRVAEKYTITIQDLNQEDSWKLFKHHAFPYHDGNLPSNID

EQKAKLVCIQCAGLPLAIKAVAGAMVSSTHPQQWEWALHRLQNAQADRLDDFLRLSFDAL

GNDDVNMQLCFQHVAAAFPEDEIIDAQEVIPFWMGEGLLARKMLEHVPQHILADRCLIEP

MIRNTEERPVCLRMHDQFELGKLYLHLLAERCLIEPVVRDTDGP

>LL_009882_T.1

MLRSYLENRKFLMLLDDMWADHDMSTSSKLQELGASLNDRGSKIVLTTRDKEVCVRMNVQ

ETIEVEPLLQEEGWQLFRSRAFENGNGNVPRELEEVAREIAKECKGLPLAITVIAAAMKI

HGTFLHQWQNALDQMKTVPEMFDGLHSTVDKELFQRLKWSYDVLKPDSLKTCFLSFAAFP

EDRVISCKEVIDIWVAEGIVKGSGNSYLRDTADGFLNHLRNRCLIEVVQTDEVGRISSAK

IHDILRDLAIRIAEKEHGCYFKAGQGVPDFPEEVDRDHCTKMSLMSNNIQSLPRTFACST

LTVLLLSENRDIKEVPESFLNELPSLRVLDLSETGITCLPPSIGNLKRLAFLQLRRTGIC

ELPETIGDLKDLQFLNVSRCFNLRCLPERINELKRLRVLDTEFCDQLRYLPRGISELVAL

ERLNTVMSISLSFEDLQSLRRLRVLSVTPKSPVKEGVMGNWSKMRALTLCYDDDVNQDEL

PQDMQAMKDLERFELYRCDVERLPSWLTQFRKLAHLVLWECKQLKELPAELPCLRELWIR

DCNKLEELELGMGFPKLEKLTLTWLKSLQCVGAGE

>LL_010196_T.1

MMDSLSCKNARSRLEEQLTSLIAIFQQMYLSTLSKPQKEQSLILENFLKTLLKGVELLKK

SENTSCFKFFHGLRYWYQIRQLEREISDLLQYQMPVHIFLEVKSLIAELNNLRQLYE

>LL_010197_T.1

MNETIFKNVSKLTNDPQQNAVMLLQQMGADDMLDGSFVECPCNYDGPGKSDLVVGLEKNI

WNVKRTLLQSEVSIVGVQGMGGLGKTTLALALSDDKEIKGVFQNNIIFITVSESPNLKVI

LETMWEKIVRKKRPEFQNVEEAHRELQQQLLRQAKPTLVVLDDVWSR

>LL_010227_T.1

MAILQSFSSPSSGKQINGSLLTNTSSPSSQSTFYDVFINHRGPDVKKSLASILYHSLQHM

GLRVFLDSGECEMGDYFPEKIHHAICYASVHIAILSENYAHSPWCLAELALMLGTDAKII

PVFYRVPPSVPRHCTGVYADAFRQHELKGRYSKEQIDEWKAALHKASLYSGWEFNEHNDD

QGKLWKNIVDTVLTEVRRRSSLEVAEHPVGLEEAADDFNRRLEEFAQEKEKNPNLVGIVG

MGGCGKTTLARHLFNLNNSAFHASCFLFDVREASANNKLPALQRKLLKELLHVDYEIDSV

SQGKEFLRSGFATAETCRFLIILDDIDHNDQLDALLAKNVLRSDSLIIITSRDRSLFRRN

PEAMLYEMKSMDENHARELFCLHAFCHGNPVSGFENLVEGFLKACDGLPLSLKVLGGHLF

GLFDKKEWGLELQNLSKVPNKDIKHKLQISYDALERDEKKMFLDIACFFIGYPKNTAIQI

WNGSGWSGEQGLQTLKYKSLIEVHNGGTLRMHDLLRDLGRDMAAAEQNVPPRLWKPQDIN

PLQRKEFRRVLSSSHCFRSFLHLRCSPEKPQTLYKSSRDLSRNKKSPAKDHSCSSIEFSR

LDFFIGKDCLQPEVLKSSRDLLWLKWEDCPHKSIPSWIPMKSLRHLEIIHGELKELWQGI

SEAPLQLTELIIQWNPLTALPMRFGSLTNLEHIDFHGCNRLSVLPKSFSSLTKLQHLDLS

ECASLRIEPCNLSFRQTQSTYQDFLRTLRLLGTNVSKEVVNQIVKLTSLECLTIGSDSLT

SLPSSLENLTRLSVLCIGGCKRMKFLPASLRKLSLLTHLSIGRSGVECLPEEIFGELTKL

QALEVEDCPITELTFTNSKAKGFRGSEEEIPYLNKSNGAFNSLPSSIPECMCMLSLQNIT

MRNTKIRMISISEDFCPNLKTLNLGENGDLVEVDLTLPSKLECLCLSGCGRLKRIPGISD

LAKLKELGISGCCELEELPSLARLSALERFTADKCWKLQNTGGVEQLEGLKDFRYLADNG

SVWNCFHDLQRIPSHQMILGGRAGAGVESINLSASDFSDGTATDSFTVIQQEYVIGGDMI

NVWNSSCKTSIVMIICFVIESFSENSWVRFRGFPGVLDLDGAVWIKAGEWIIASVFAIEE

ASAWKKDIANLKILQVLLAKSPHYRLKKAFITMVNKGGEDKITEILNKIFVSMKETGAQE

QSGSGSTFKLQDGVEGRECYH

>LL_010265_T.1

MENPTLVSSFPQSHSEIEMENQRPFPPTIDPLSASASSSTAVAYGYDVFINHRGPDTKKK

LAIPLYRLLISRGFRVFIDQEELEMGLDFSYQLEGAIRNASIHVAIFSPTYAESQWCLDE

LQLMHETKARIIPIFYNVNPNDVRWVGQNRNGLYARALQQLETK

>LL_010407_T.1

MGDLQIVATASELVSNIVGAVRALEQASRDFQEAPKKVKTLEELISSLEGLIKHVKEKYS

HKIHHSQLHSQIQSFETLVDRVQPKVKLARKVSSKKGFRRFASVMWNSMVGDHLTKIICS

IKGDLNWWLDSQEFSANMQKVIDSTANRLPAMLKVRLEAGYPVSKKCEMVRVLLDRDDSP

QVILLVGLSGIGKTCLARQVASNPPERFIHGAVELGLGQWCSRMACSGNKIEYHKRLAKK

IFRFLVQIGCSNKIWEETKGDLEDVCCLLQEALIGKSLLILLDDVWEPDIIERFSKLYDN

SCKYLATTRNEAVYEITEAEKVEICKDDTCEVSKAILLYHSQLSESELPDVAENLLNRCG

HHPLTVAVIGKALRKETRPEKWEKALSDLQTYATYAPVPVPYLNEKEAENAATVYGSFEF

SLEAMACHARDLFTAFAALSWVEPVPEVCLEAVWSALGQDSTFLLAASKLIESSLLSKGS

LFTKAETYMMYHVHDMVSLYLENKVHDAIKMLLMDTRSEASAAVAPWLFVFGKEEAKKVA

EQQLKGFLRSVHERQVVVTLEAIVQSLQASKSVTELEASSVNFRCLVGPEIVYLISEGSE

ILIAAVARSMSNFFCLDDYMQYMESLESVGIIEKLTKLLETCDDPLVQTDVAMVLSRLAE

CGKEDMANEVLMKIPMNKLVDLLDPDIEELHDSLLKTLMILAKAGKEKAVEKMFIAGIER

KLIELLENGSEVAQHRAVIALKSFSELGGSDLVHGFLRSGIMEHLPWHARLSLEKFTNSE

RAIPSLPKRHTVEELVSNILDKNNINAMEAMQNLVPIVEKADVPEIREMILQTALVENLE

KQLQHVLSDNNRIKSEAIFVLMKLGSSGGEPCIRKILKFNIVQNLISLMSCKSPELQDTA

YTALHQLMLGAGGNLVVDRILETSQIEKLTQLLESRSLKINEISMQCLEDLVVLGGKACI

ERMLTLNVVEKLAILEKDNSQFNGVVMNFVKGVDKCKYLSGAERRVTKQQIVRKVKTTLK

DANLVARIIGGIECASSSKGSSRSNRRKK

>LL_011162_T.1

MESAVTDPVSATYHVFINHRGCDVKKTLASLIYHRLEEAHGLRVFLDEEELRTGDEFPPA

IVAAIQSSSVHVAIFSPRYAESRWCLDELCWILDKPNRKIIPVFYDVEPTDLRHIHSGRF

EDAFKQHQSKGRVTVEVIEKWKTALKEASDISGIKFKSSESDHGEVLEEIVKDVLKGVKW

EPLEVATYPVGLDPAVKEINDEIKKQWDLNGTAIVGIAGMAGLGKSTLAAYLYNLKRSDF

SRSCFLSDIRKRDLPSLQRKLLKDLLGYDFHIEDTREGKGIFRDRLRWLRVLIVLDDADH

IEQINGLIVTDVVGSRSLILITSRDQDLLRRSSPKTVVYNVKPLQREHARELFCRHAFNQ

SEPSEGFEHLVEEVLKICGGLPLSIQVLGGQFHGRCEKEHWKQQLQKLSRRLPDDIMDTL

KVSYEALKSEEKEIFLDLGCFFVGDEKEFAVQVLEGLGYKDVSDCFENLRQKCLVESHDD

IQSEEEISIHKITMHDHIRHLARHIAREEFRVLPTPKPLRLSCSADIEQMLQMQAGADSC

LIRGIRTQGQHLPDGTNYIKGVGVRLLEVEVPIDLSSFFGLWDMSGDLVCLRLRKFRSIC

FPDTISLKSLRVLELNGHGDMEQLFQRFNAPPRNLSVLDIDARGSHPRAILNPLGHQRFI

WGCARPMTGTHNPVKSGATTSTSSRSFDLSNSQATLDNAEQGFMACFYRFLEWMRTSREI

RSKGVLSRDGARPMPGTNSPVKLEATLDNAEQGFTASYFYRFLEWIGRSIKLPRRIVLKN

IPFIESLPIDFRELKSLRQLDLSGCTNLTQLPNSFSELMHLQYLVLRDCRNLIIPVDILG

NISSLEYVDFRGCRKLEYLPTGIANQKSLVFLDLIGTALKLPLKPALIPEKVQLLKIGNN

ASFIFEAPNIQSAQSAYSAPNIWRPCIPSSFSQELNETRKFESCEQCGSIICTHLSKKKE

LSSCAAGRESLQPTESLQPQLRTQNLEGTELQQYLGDLRRLKELKELNLIEFHSLNHIIW

EEIAAPNMKVLVISYCPISVFTFKEHAVMGVLRDFTLSRTKITEIHIPEGVLPSLETLDL

SENRELMRVEGLPSTLVRLDLQGCSRLEALISLSNLVDLKFLSVNECSQLETLNVEGLTS

LEEIQAEGCRELKSIHGLSQRERLKCLCISTNTDVIWNEICNFLTFMTSREGLSTAFFSG

KSTAVTYGDRAFCNMLWKEMQSMTSKFQLKVVDAVFTWNHFHTKMEGFQSYGAVLMCFLT

RGGDGTFTVKFEASGDGGSVDEYKMVC

>LL_011174_T.1

MAADIGVSSNILDLLIQVTPEDPKDRNWKWSMDYVNKLDTDLLQVLKTDFNRVKGFLIHI

INQFQARQKRLPPPVERCLERIGAPLREAIHLIDDAKRQQDRGGCLPCRREGSVSSKNRV

WKARFDALLQEFQTVFSTSANSEQIVSSAARLLQPVPDSSFVGSGIQSNREKLQKWLGET

GREARVIGVYGTAGVGKTSLLKIIYNSYKKEVSRFFDVVIWLTMSQKYEIEQLQGSIAEA

LNLTLEETANAEAKKMKLSESLGRRKVLLILDNVRQEIDLSEAGVKIEDANGSKLLISSR

NREVIDKMGAKREYSLEIETLSEEDSWYLFSRGAFANGEDITRKIAINAVAAAMKSKEES

EQKWSDASLSINPLSSEEGCELFMRGTSKNGAIAEKIREDTAKEIAKDCKGLPLAINAVA

AAMSFKKTQEEWKGALDSMRSKDLPVRTDDGTINAELYRLLKWTYNELSDGRAKVCFLTC

AAFREDKEIAVDTLVGMWDAEGLLNPAKTPDSTEGLQNRTETADLMDAGRQIINLLVSRC

LVEYVKPIVDDDYHSVEYVKIHEVFRDIAIEIGQRDEKCLFEAGQKLQEFPTRVPKECRR

ISVAHNAIRNLPNDLNCEGLQSLVLASNIPLEKVPDGFFPSVPSLKVLDLSCTSITTLPK

LEQLKQLQFLNLSSCSKLVDLPDSICSVLSLKGLDLSRTSIRTLPGNLNKLSGLQVLNLN

SCSELQTLPDNIVELKSLKHLKLDACFRLEKIPEKLSQLTSLDTLILPLMESTCAMSVQD

LSNLSNLVELTTVVKSDNADMKPWSKMRNLTLEYEDEENADRVEVAQSEHVILQNMRNME

ELQNFHLINYQGGTLPKSICKLQKMKLLLLRSCYHLEELFALD

>LL_011605_T.1

MLFTRIVFREGPVLMELEDCVAGEGEGLLLALTVAAAAMNGKNTVDEWGNSLSLMKPSYP

SFPHTHAPIDQELYQRLRWRYDALPPSNVKNCFLCCAMFPEDAALMWTN

>LL_011941_T.1

MTFFLVQAECKGLPLALKVIGSSLRGQPRPVWESAKKKLANGESISDYHKEGLLSRLETS

IDVLDEEARECFLDLGSFPEDKRISVDALLDIWVCVRRMEWCDAFVMLLELASRSLLNLT

SNARSQAINYGSASELYFFQHDVMRDLAIYLASRDRIIQRKRLFMPKKEDSLPGKWESFK

SQAFDAQIVSIHTGPMEEDQWCEMNFREAEALVLNFSASNYVLPSFLSSMRKLKVLIVLN

YGSKRATINGLPAISSLTELRTIRLERLNVPPLQEHSKVLQNLEKLSLSLCEGLGNMARF

SETQSSLKLPIMIDFNLDHCCDLEELPLDICDMPAVENWSITNCHLLRKLPDDMEKLSSL

RMLRLSACLGLKELPASIGKLEKLEYLDISLCECLKELPEEIGQLKKLQVLDMRECSRLR

KLPKSVEGLESLKHVICDEKIGQQWLRIKSSVLKELIVEIVEI

>LL_012092_T.1

MLESKAPVIPLFYRVKPHELRYGQAIQRLEEKRRHDSATIENWRKALSRVAGISGFELEA

CNGDEGELVDMVVKEVLKKVKKPALHVAKHPTGLDDKVKDFENTVLLQLQQSGKP

>LL_012159_T.1

MLQKADSLSLFCYWAFGVPSIPTTGDGNLVKQVEAVCMGLPLALKVIGSSLRNEPPAVWE

NAKKKLSRGESISKHHREDLLHCLETSIDVLDDESKECFLDLGAFPKGRKFSVDSLLDIW

VHVRGMEWQYAFVVLLELASRHLLNLTSDPGSLAISFGCACELSFSQHDIMRDLALRLAS

KDSTIHYKRLFMPGKENSIPTNWLTLKDHTSRVQFVSIHTGPMEEEDWCQIDFPEVEALA

LFFTASQYCLPTFLHTMPKLKVLIMYNYGSKRAVLNGLPGFSSVTQLRSVLFEKLIVPPI

SEYCRSWENLEKLSVCLCEGFGNMTLFDKAQVPKLQKFVEIIFDHCSDLEELPANICNLT

SLERLSITNCHLIQELPDDLGMLMSLRLLRLSACPGLSMLPPSIGRLQRLEFLDISLCSS

LKDLPMEFDQLSNLKILDMRECSRLKMLPKALANLRSLQR

>LL_012214_T.1

MWEKIVRKKRPEFQNVEEAHRELQQQLLRQAKPTLVVLDDVWSRANLEKLLFKGAGYKTL

VTTRDRSTIPKTASTQLYELPLLDDADALALFCFWAFGQRSIPSAADEHLVKQVQAQCKG

LPLALKVVGSSLHGYPRPVWQSAKKKLLNGESISDYHKEGLFKCLKTSIDVLDEEARECF

LDLGLFPEDRKISVDALLDIWVYVRKMEWHDAFVILLELASRNLLNLTSNIKSQAINYGS

ASELYFSQHDVTRALALNLASESRIVCRKRLLMPKKEDSLPGKWELLKDQAFDAQIVSIH

TGAMEENQWCKMNFCEAEALVLLFSGCNYFLPSFLSSMRKLKVLIVFNYGSKRATVNGLP

MLSSLTQLKTIRLERLIVPPLQEHSKVLQNLEKLSLSLCEGLGSMSRFNSYQSNLKLPIM

LDFNIDHCCDLEELPLGICDMSSVQNWSITNCHLLQKLPDDLGRLSALRMLRVSACLGLK

ELPASIGKLVKLEYLDISLCECLKELPEEIGQLKKLEEFDMRECSRLRKLPKSVGGMRSL

KHVICDEKIGQQWNRVKSSSAIMDLRVEIVEAHFSLDWLDG

>LL_012474_T.1

MAPDAASFASSSHGNTNTSVFDVFLNHRGPDVKKGLASHIYRRLIVHGLRVFLDQEELQR

GENITPQIEGAIRTASVQVAIFSPKYAESPWCLSELVLMLESGKPIVPVFYNVKPSELRW

TLENKATVGQRRQKKPRHDSSTVEKWSKTLSDVSKISGFELNACNSDEGQLVDEVVGQVL

KMVSKTPLNVAKYPTGLDDKVKDVERMVLLQQQSQKARIVGIAGLGGLGKTTLAKEFFNR

HRSNYV

>LL_012475_T.1

MILDDMWKTLELHSLGVPLNDKGSKIVLTTRIKQVCTGMEAGEIIEVKPLSEEEGWQLFC

RRAFPNGNVPQEIADVAREIAGECKGLPLAINVIAAAMRNHTYRHEWEHALRQIQSIDQT

FYGMHNEVEEKLFQRLQWSYNGLEGYLKTCFLSFAAYPEDSEIICGEVIDNWIAEGLVKG

SGNSYLPDTADSFISFLRDRCLIEVVHNDNVGRISVVKIHDVLRDLAIRIAEKEQRCYFK

AGQGICDFPVEEVDGKGWERMSLVRNDLHSLPTTFACSSLSVLLLNMNRG

>LL_012476_T.1

MHSRIDKELFQRLEWSYNYLPEYLKTCFLHFAAYPEDEEIQCEEVIDIWIAEGVVKGTGE

SYLPDTAQSFISFLRDRCVIEVVNYDVVGRIESVKIHDVLRDLAIHIAEKVHSCYFKAGN

GRSYFPLEEVDSEQCAKMSLISNNLQSLPPIFACPSLSVLLLSRNRGLKEVPGSFLNELP

SLRILDLSRTGITCLPPCIGNLKHLASLQLERTDICELPASICDLKELQFLNLRLCKKLR

CLPERISELKRLRALDIENCSTLSHLPRGISELVSLERLNMFGSISLHFEGDANAERMYA

CFKDLQSLRRLRVLSVCLKSAVKEGVMGNWSKMRDLSLSYDDDVNQDELPQDMQAMKDLE

RFSLLTCDLERLPSWLTQFRKLAYLRIMSCKQLKELPAELPCLRELFISVCDKLKELELG

MGFPKLETLMLDDLESLECVGAGEGAAASGSGSGGLGEGASPLPMFKTLLVARCPKLKRL

R

>LL_012665_T.1

MAAARDATSFSSSSTSNTNSSYAYDVFINHRGPDVKTGIASHIYHRLNDHGLRVFLDQPE

LQEGENMTPQIEGAIRTASVHVAIFSPRYAESSWCLKELVLMLE

>LL_012666_T.1

MILQRQSEKARVVGIVGVGGVGKTTLAKEFFNSPKSNYDRSCFVSDVRENAA

>LL_012749_T.1

MNIIASSKSSSEELKQKLLLLEPTIDEISKLSSDSDPARQKKGRSLKDFQAQLQDGLHLV

EKLERVSCFDLYRRYRYGKQILKVEKNVNDFLLTQGLANVMLDVHKLNVDFKGCSERSER

IEEMGRHILENVNAKITNDASFNSLMLQQMNSIQLFQSSFDELHDTTMAEQSTGSCNSQV

PGMPSFVVGLNNLVNDVKQIIFQNGVNIVGIEGMGGSGKTTLALALCNDSQVKDFFQNNI

VFITVSQTPNVKDLLETLWDKVIGAPRPVFQSIEDAHLQLQKNLSLKGNQQTLVVLDDVW

SRSNVEQLLFQAEGYKTVITTRQGYAIPSTNSTRVYNIPMLQKADALSLFCFWAFGQPSI

PTTEDEDLVKQVEAVCKGLPLALKVIGSSLRNEPQPIWENAKKKLSRAESISQYHREELL

SCLETSIDVLDDESKECFLDLGAFPKGRKFNVDSLLDIWVYVRGMEWQEAFVILLELASR

NLINLTSDPGALTLQKVKGINSSEG

>LL_012908_T.1

MASTSSTAASTSSTAADSNHDVFISHRGPDVKKTFASHLYRRLLFHGIRAFLDKPELQPG

DDLTSQIKGAIRSVSVHVAIFSSGYADSVWCLDELLLMLESKSPIIPIFYKDVEPADLRW

KRSDKRSYADALEELEKKTSYDPKAQEFKLRYDSSTIEKWRDALSRVSMISGFELAAFNG

DEGELLNKVVDRVLKMVGKPALDVADYPTGLDDKVNDFENLVLLQQQSEKPHAFGIVGLG

GVGKTALATELFNRKKSDFSRSCILFNVRDSAGKGTLNFLQTQLFKCLTESKEPIVSVPE

GKGILRKHISSSEVFLILDDVDDVDQIYAFLPHQVVLHPDSLVLVTSRNKNILISAGVEE

SSIYNLTGLSKPHSVELFCLHSFNQLHPLPEFESLVDKFVEACGGLPLSLKVFGALLKGK

HTSYWEDQLGKLQRMLPEEIQQRLQISYDSLDREEKQIFLDIACFFIGEDRDTAIRIWDG

GLSGFQNIQSKCLVEVNSKNKIEMHDHLRDLGRDIAKDSRLPRRLWRWTEDDIDDLLEQQ

SSVIPVRGIRMVLKEYNEYIDDDDALGGIRRRLQLFDDDDALGGIRMRRLQLFDDDDALG

GIRMRRLQL

>LL_012993_T.1

MVISTSANAVELVSSAAPRPEVLLQPVPDSGFVGSVIQSHQMQLQAWLTQPHRQARMIGV

YGMGGIGKTSLLKLVYNHYKEVSGVFDVIIWLTVSQQYQIEKLQASIAETLNLKLDESSD

KDIRKMKLSASLGKKKFLLVLDDIWHPIDLIDEIGVKFGDHNCSKVLMSSRSRDVILEME

ASDDYSLRIQPLSTEEGWELFRTQAFTNGALPRDQNIEAIARNIASECQGLPLALNVVAA

AMRRKKTEDEWRRALTLMTYVDPSFRTTHSTIDAYLYQRLRWSYNDLPDRNLKICFLYCA

VFPEDAVISAERLVEMWNAEKLVTQMDAGYDYIDALVGRGLFEYVAGNFENDVKLLGGVL

CRHINGKAVKVHDVLRDLAMYIGRSEDNWFFATGHHLQDFPSEDEIRDCKRISVGHNDIQ

DLPTDLIC

>LL_013064_T.1

MQTLKLDPNRNMNIPLNESLQGKRFIIFLDDVWDHGAELLEELGVLRVIHLFDSKIIVSS

RNYRALLEMGVAQESTITMGDLNEDDSWRLFSYYAFPYNSGNVPPHIHHETAKVVCDKCG

GLPLAIKAVARAMAGITDAKEWGFTVQRLPNANSKGDEAVYGRLRLSYNALASYGVHLQL

GFLYLGAAFLEDEVVQVESQAILL

>LL_013449_T.1

MQHVDPDFLASTRIDEGLYQRLRLSYDRLPHSNIKNCFLYCGTYPEDYNYNSNVEALVEM

WIAEGLLISRETRCSYLMDLGRSYVKLLVERCLFQVVENDYGRELIQVHDVVHDMAIFIA

EKEEKCLFRARQNLQKFPADEKEIRNCKRIAMGFNNISVLPTDFRCPNLLTLLLAYNESL

REVPNGFLVNLTSLRVLNLSGTTIESLPISLWHLRQLEYLALEETLIKDLHEDICNLSQL

QFLHLQSCRQLESLPCKIGQLQNLKTLNVSDCCSLTGIPREISQLTSLNRLMLWTSETTE

NSIMDVEEVESGVCSLKDLTSCPNLMELSVHVKAGMEVEGIRLGIKEGIMGSWVEMRNLS

LAFDVQAHDVMEDLPQDMQRMKKLHRFWLIKYHGRNLPNCICEFPQLEKLYLYECYQIRE

LPPLERLPNLKSLTVEGCTQMKELRIGTWGSVIGFPMLECLNLYDLPMLESMASSSSNVV

WNEQIMSKLQTLGITDCRSLKGLPMGIEKLPNLREIKVQKDWWELN

>LL_014249_T.1

MDKMSNRGFVSGFIGVGIHMTIQHILQLIHIAKRCKKELADLKELVMSIELTIMHIQQNR

MAFKSNEASETASDVNRWLNDLDALLLDASKMVQRCSIPKSHVNFSGRQSKKICSLNKDI

REHLKLSPLLVRIPEEDTQEGKVKVRDTQKVLEGLGKTKDSLEPLASSATTSQAAMKNLV

VGQNQAFQSLSKSAEAVEDFWNFRGKKKLIEEPEIDRGKKKLIKEQEEALTNLENLVIED

AERKTIVVVDPCGSGKSLILETLFNSEKVRSHFSNGLLLWLSLSQRPSVTSVMNHLCKQI

AVQTKIEIRVEQDKKVWLKEQLQQSSSFALFLDDVWGRHARNLVDLGIVAAVADHRSSKL

IVSSMDPAALLEMGVAEEDKIKIQELISQEDSWKLFKHHAFPYHDGNLPSNIDEEKAKLV

CNKCAGLPLAIKAVAGAMVSSTHPQQWEWALHRLQNAQADRLDDFLRLSFDALGNDDVNM

>LL_014250_T.1

MARMQSHRGFISGFISVGIHMTIQHILQHILIANRCKKELAELKELVMSIELTIMHIQKN

RLAFRSDEAKEMPSAVNGWLEDLDSLLLQASKVVQLCSRPKSHCNFSGFQTKKKINLIIS

DIRKHEELSPLLVRIPNDQVGDCQKVLAGLEQTKDRVEAMASCATTSQAATAPRGTARKN

LTVEEQQAFANLEKFLTDNVEATKICVLGKTKRGETLILKNLFESQKVRNHFSDGLFLWL

TVSQRPSVTRLRNDLCNQIDVNLIRDVKDQEELKIRLNQTLQQSISFVLFLEDVWGRNAA

ELLQGLGILDAVCDHKKCKVIVSSTDHAALLKMEDNKYTITIEEPVIVGQKEDLATLEKF

VTDDAEANKIGVVGKCGAGKTLLLKTLFNRQQVRSHFSDGLLLWLSVSQRPSVTSLRNDL

CKQIAIQKRVDLSKAIKEEEDLSEQLRKSSRFLLFLDDVWGNDAETLIQNLGIVDVVSGP

TKSKIIASSRDPSALSKMGVAAKYTITIKDLNRDDSWQLFKHYAFPYNIGNLPSNIDEGK

AKPVCDKCAGLPQAIKVVGKAMAASTHPQQWEWAFDRLTKADSLDDYLRLNFDALGNQDF

NMQLCFLHIAVAFLEDEIIDAQEVIPLWMGEGLLAKKMLQHEPAHILADHCLIEPMIRNT

EGRPVCLRMQDPFQIGMIYLDLLAERCLIEPTARDADGRVLCFRMLKVVRDLAIPIAKNE

ENFYSCVGNRGLDDLPNNEPSKVKRIFLSGNDLRDLEQFSSYKEIRSLLISQETGLTKII

PKQVIGNMRSLKVLDLSGTSARSLPENVGCLKELICLKLSGMPIKRLPKSLRDLGHLEIL

DVSFTGIKELPSDIHMLRSLRYLGLRGCKYLQNLPNSISGLLSLQYLYMDGCSLWTKPTR

NRCKSVASMKSLGDLVQLKQLALQNDSEAINELGILGSMAEMDSLLLILPNMECLTLDIN

NMSKLRSLSLKCCNFTKMEIKMRNEVCSLKNLSYLKFYECQRLEDLQNLHKLQNLRHLEI

ILCQKVRDFPKCEEEAFPLLRIFSLVGLQNLVELPDAMPFLEIFTIMDCPKLKMLPKTYL

NLGTRIRVYGCPEIVADFKMVEMENMDLLQVITISISAEEIIQKYLQVLQKNNEDWLFGE

SW

>LL_015661_T.1

MDPVSQSAIGTTVGELFIVTRTIMNSMLSSKSSSEELKQKLLLLKPTIDEISKLSSDSNP

APHRGGPFKDFQAQVQDGLHLVKKLERMSSFNLYRRYRYGKQILKVEKNVYDFLLTQGLV

NLIPDAHKLNVDFKDRSERSERIEEMGRHIMDSVNAKMTNDASSNSVLLHQMSTNQLFQS

SIDGMHDTIMAEQSTSKYNAQVPGMPDFIVGLNNILDDVKKILFQNGVNIIGVEGMGGSG

KTTLALALCSDSEVIDFFQKNIVFITVSQFPNVKDLLETMWDKIIGGARPVFQSIEDAHN

QLQKNLSLKGNLRTLVVLDDVWSKSIVERLLFEAEGCKTVITTRQDDTIPTTNSSRVYNI

PMLQKADSLSLFCYWAFGVPSIPTTGDGNLVKQVEAVCMGLPLALKVIGSSLRNEPPAVW

ENAKKKLSRGESISKHHREDLLHCLETSIDVLDDESKECFLDLGAFPKGRKFSVDSLLDI

W

>LL_016351_T.1

MDSVSQFTVEKVLDGLLEVGEIIYYSKSEGEEMKEMLSQLKDVIDENMNLISHSDISSDI

LKNLYKGFRAELEKGLVLVGELENISSFNISARLQLGDRVLECRKKIMESIIIQGSPYVI

NTVLNLTATVRDNERQRLDDVEKLKANFRNLERRLMLENVRMDEPADVPPLPNSVVGLNI

ASDAVRKILLQDDFDIVGITGMGGHGKTTLATYLCNDAKVKEHFDDRIFITVSQLDGDGK

DLLNILETMWVKIVGGRRPSFRNTEAAHSQLQQAIEQRTNRRTLVILDDVWTKSNAEILL

FKARGYKTVITSRHDFTIPQKKGSYLHKMEILGEADALSLFCFCAFAGQPSIPKTEREDL

VQQVAAKCHGLPLALKVIGSCLQGQPRPVWESAKADLSTGEYRNDYHEEFLLNQLEKSIK

NLDEELKKCFLDLGAFPLGGKFSVDSLLDIWVYVRGMKWESAFVVLLDLASRNLLDLKGD

SRSGRAPITYHCASELSFSQHDVLRDLALRLARRDSERLFMPCRDHGIPQEWQPPGDRPC

CARFLSIHTGAMEEQDWCQINFCEVEALGLFFASTSSKYCLPTFLHTMPKLKVLIIYNYG

SQRAVLSGLPSFPSLVQIRSVLLHKLIVPPRYENCKLEKLYEDCKLEKLYVCLCEGLGSI

PLVDKEPEALNFPNIIEINLDHCSDLEKLPVKLCNLTSLQRLSITNCHLVPELPDDLGSL

SSLKVLRLSACPSLKEIPPSICKLRQLEYLDISVCRGLHNLPPGFAQLSKLETLDMRECS

LFNNLPTFKLRSLKHLIISDNEKVAKRGEWLSAIPNLTIHDVPEVFTLDWLSDGTFGS

>LL_016385_T.1

MDTVNCNMLYSRLEALLPSLKDTVQLLYSPNIQKPQNEQNPIYEQSLSIINKGVQLVKMC

EKTSPSNIFQNLRYVSEIHQLEKDIRYFVQYQTPANLSPQLSLELKNLISEIKNLRHLYE

LGAVDERKVNETIVPKLTNDPHENAMMLQQMRPDGMSDGVFDESPICNYNGSGKSDFVVG

LEKNIVNLKTILLQREVSVVGVQGMGGVGKTTMALALCNDQEIKGNWGSIYLNHLCL

>LL_016755_T.1

MQVDPLKVVLDKLLEEAQVLLARTCVRERPNINKCCASFVRLLKTLRPLYITDSNSCCER

VNSASDSTCVDGRSFLITAADSLQMGFFQDLEILCILFDLTQQIVSSLQEWSLNLEAHHS

STEGKFDGYLMFKRRSFVSAFYVLQDNEGNATPNTQFSRFNWRVPSLSFCLPSWNFSRKS

RPSPPSDEALRIPIQGAEVEAADKKMLVQEALQIGDDNMKQLRLAIMDLKNQLCRAAESL

KAIFFSTESGFPNFLVRDRLKRLVHSFLTFENYYEEPSDDSVEDEDPLHDFCHCHQIHCP

IGSRTAIPVAEKLVSELLDSGGSLVVGICCPRGLGKTSLASCVASNCKVKRRFIGGVYWI

TAGRKTDAKSLQCCLWKAISGSQAVFTSVEEGLTALKRKFSECLLPSLLIVDDVWEASQV

EGLLCFDGKERGRILVVTNNSQILSSAEACIYALGCLKEEDAVHLFQWVVEWDGPLTLEQ

HDLATKLARKCNGSPLFIQALSSALYGAGAHQKLVLELLLNEGGEVAGEQMNTVDQITTN

QPTGEACMDLAESSVSGKQNFDRTANCSYSVLYACFSALGFIHPLLQECFLDLAAFPEGE

WVNLSTLETLWLAFSNGLSQEEIVVILSILVFRSMVDWRLNVLDTCGLDSELEFKLSEPF

YNLASNIICQDIKEYSKKKDSNVYKIPFELLNRRKEIENKKISSKSMGVMTKFLGSGNVY

MNCTEDEEVNSGELVTYSKHNDSTSRLNHIKSESSLVTEVYQGKNSVNNTDRNCQVTRFQ

KECRLFGTDCNNNSFQEVSLDETLYRATKFSIYNSDIDQLLPTFGCPFLRVGLLGGNARL

SLPSSAVLTKMTQLLVLDLSGCTSCTHLPKAISALKSLQLLDLSFCTSLKSLPSSFGCLK

NLKFLRISGCTSLTHLPQTTGMLTKLEVLQLSGCVKLKTLPSTIGQLSLLKILDLKGCFS

LCSLPPSVGLLRELRFLDLSKCSNLSYTLPYHLQLLLLAIGSCKTESMAVPAGLWNIDQV

EVLRLAYLKNMTYLPHAVGKMQHIRKLDLSNCRRLEHLPEGIGNLWLLQELDLSHTAIRK

LPDSVGQLRNLLRLCLQGCSRLLCIPRSISSLPKLRCLDMSECWDLVLLPSSIGNSYSLC

ELVELNLTCCSFKSLPSFSLHALPKLQYLKLVGCDSLISLPSTFEQLTSLKKIDLEGCHA

LTSLDRIGLGKLDKLTWLSLRNCTSLTSMAMEEMVELNSLKYLDVSGCTGLSPFPQALLT

RALEGNLTLIRWGAVWPEQRNFS

>LL_016925_T.1

MERVQVVGITGLVGVGKTTLAKELFNRKSSSYSKSHFLFDISENAKRSLPSLQSSLLSGL

LHLQRHIDNRHQGIELLRKHLSSSKVLLVLDDVDHVDQVNALLQVDVLRPGSLILIISRD

KNILLRSGVEESSIYKLTGLNRERSRELFCQYAFRQPHPLPGFEYLVDQFLMGCSGLPLS

LKVFGA

>LL_017007_T.1

MAVSSSTAASTSTAAAANSSNKSYAVFINHRGVDVKETFARSLYLRLAENRVKAFLDKDE

MHAGYNITSQIADAVRTATVHVAIFSPRYAESVWCLNELLLMLETGAPIIPVFYRVTPAH

VRWGKDGYYAQALQDLANKRTHEGAMRYNSETIEKWRGALFQVANNSGFELETRNREELQ

LELLDKVVRCLLEMVKMVKTPESLHVAKFPTGLDEKLKDFEDTVLLQQQQSGRPQILGIW

GLG

>LL_017008_T.1

MLIEPLKSSKALIIVDDVDDVNQVDALLPIQNHDLYSNSLILITCRDRHVLSSRVEKSSI

YQLTGLNPQHSRELFCSHA

>LL_017091_T.1

MAVSTSTSANDPNNNYVYDVFLNHRGPDVKRTFVSHLYRRLLPYGLRVFLDHEELHEGEN

LTTQIKGAIASASVHVAIFSNGYADSNWCLDELLLMVESGAPIIPVFYGVKPANLRWTHQ

QDEGVYARALHNLEKKRTHDCRPRYNSTTIGKWRNALSIVAEISGLDLEAFNRDEGELLD

KVVESVLKRVKKPQLDVAKYPTGLDEKVNDFERTVLSHQPSGRVHVVGIAGLGGVGKTTL

AKELFNRKSSNYSKSYFLFDVRENAKSSLHSLQRKLLNSLTQLDLGIDSVDQGKAMLKKY

LSNSQLLIVLDDVDQVDQVDALLPVNILHRDSLILITSRDKNVLARSGLRESSIYQLTGL

NAHHSIELFCSYAFCQPDPLSGFEYLVDQFLRACKELPLSLKVFGGLLHGNNDKSYWKDQ

LDRLQQALPNEIQNTLQISYDALHPEEKQIFLDIACFFVGENRDTAIKIWDGSGWKGSLG

FQNLQRKCLVEVDIENNIIMHDHLRDLGRDLAKEPGLPRRLWRGIENIDDLMQQSFVMRG

IRLFQSEYKDEDDEHAFFSFYKIRKLELLDVEILMEHSLSGVYSPNLIWLRWHECHLSSL

PSWIPIKNLWVLEVHGTVLKTLWQAESQVPLRLRELHIVAPLSNIPKSIGMLQYLERIVV

DCPLHNSFEELPEEFGDLRLLKALVLKRCSKMRSLPDSFGNLTNLEHIDLSYCTDLERLP

NSFRNLIRLRYLDLSCCFDLTISSETLGNISTLKYVDLSYCNKIEVLPFQVAHQQSLEKL

NLKLINLKELPCDIGKLSSLEELELESPLLEKLPTSIGDLRSLKYLVLRRCERLKRLADS

IGLLNQLTRLTVQDCSLQELPFKTVDGESETLTESRERRRCSNLDSSIDQCMLRLQHLEL

YNTKKLNEVSFGEGVCPNLQHLLIDSCTDLARVGTLPNALITFELTWCSKLSKIEGLCSL

AKLQKLDIKGCIEVEVLQGFETLVSLEVLEVTNCVKLKSVPGLEYLTKIRKLEVSSCPEL

RDLPSVENLRSMESLDLSECVKLKSIEGLAQLTKLRTLKVRSCSELEELAGIEHLRSLEV

LEAFNCVRLKIIVGLEQLSELRKLEVSNCSELEDIPGVENLRSLKKLDVIGCVKLKHGQE

RARLIKPDRQGSPKLTKLHRRALTQLTKLHRLD

>LL_017120_T.1

MRESSIFKLTGLDREHSQELFCLHAFQQPCPVLGFEQVVRQFLEVCQGLPLSLKVIGALL

YMESPDMECWKDQLRKMSKIVPTDIQTSLKVSYDSLDKEDKEIFLDIACFFLGEDRDKAI

RIWDGCGWGGSIGLRNLENKCLVEVDTDNCIRMHDNLRDLGRALAEKEPWLRLWRPTEKL

SPFVFHRSSVRGISMVQQRFGLSLAEPVQRKKRFVDMNKLQLLRANGDCLENIANVLGQS

DLLWLCWNDYPYTSLPSWIPTKNLRVLELPKDKCQVQILDDNYPWRSCQLETLWETENEA

PVQLRELLIGHSLLQFPKSIGKLKHLQKIVADYVETLHEEFFHLLSLKHLRMHTHMELLP

ESLGNLTNLQYLDLSGSSYLQKLPDSFGNLTNLQYLDLSGSYFQMLPNSFGNLINLQYLD

LSQSFNLQTLPPSFQNLVGLKHLCLKDCRKLMLSSETLENITTLESLDLQCCGQMEGFPP

QVTSQRYLEKLYLWNINLKELPHAIGNLSNLEILVLECPLLEMLPASLGNLGRLKELTFS

GCSRLKCLPDSIGDLKQLTKLRMRNGIIKYLPMCVMELNNLEDLSVTDCPLLAMTFAGGE

RVAGGKEALTNARGRRMLDSSIGKRMLRLKDLQLWGTQIKEISFPEGVCSNLQHLNIKFC

HELVEVGALPTTLVSLELEDCPVLKKITGLCGLSRLLRWDIRQCQGVKELRGLKTLISNL

EGANLERIWDLAGWSLEMPSSDGDESDFSDDDGAAFGLQSSCPRN

>LL_017144_T.1

MDSAIDFAAEKALDGLLEVAKIIFSSNYESKELKKKLLSLKDVIDEVSKEISKKISDSDS

SSDRVRMYEDFQTELNDALRLVDKLEKKHRLDILHRFRYGNKFIDYRKKIKDFIHIQGLP

YVVHEVQNLTAAVRDRKRPKLNDEEKVTEAIRDLERLILKDVVPSLNDNPKSITPILQQN

NTIQLSQTSNDGMDEPAAAQQSHGYTSQVPDNPVGLDNPIDDVRQILLRDDVHIVGVTGM

GGSGKTTLASALSNDPKVKASFHEIIFIAVSELNGNTNSILNIIETMW

>LL_017156_T.1

MVKPKTPESLHVAKFPTGLDEKLKDFEDRVLWQQQQRGRPQLLGIWGLGGVGKTTLAKEF

FNTKKSDYHRSCFLFDVKANAQKGSLTSLQRELLKSLSGLDKPIKNVDEGTGMLQEHIKK

LISSNASLHRKHLHSLTGLDEPVHGVDEGKEMHINRLKSSDTLASNNNILVILDDVDHVD

QVSALLPVDLPSGSLIVITSRDKHILIASKVENSSIYNLTGLKEQHSLELFCSYAF

>LL_017167_T.1

MLHCLCVLIDQLSRYFIPVPMANLTTPVYHNPKASCASSSSPAAYSASTSSFSHYDVFIN

HRGPDSKKTLASHLYRRLLSSGLRVFLDREELVEGFNLTCQIKAAIRVASVQVAIFSPTY

AESNWCLDELVLMVDSGATILPVFYNVKPNVLRWTLKNGAYAEALLNHQHKRTTDPQTGE

EKPRYQSDTIKNWRKALAHVADISGFELNGDEGELVEKVVQSILKNVPKTPLDVAKYPTG

LDLKLQEFEETVLSQQWETGRVEAKVVGIVGVGGVGKTTLAKQFFNSKRSYYHRSSFLFD

VREDATRMPLNYLQSKLLKDLKQTDIRIGRDEGIGILKKQLSSCHVLIVLDDVDHVCQLE

AFLPIKDILNPNSLILITSRDKHVLKSSGVLESSIYYLEGLNRPFSKELLCSYAFYHPLP

PPEFADLVDRFVTVCHGLPLALKVLGALVCGENDERYWEEQLDRLQHTLPLEILERLRIS

YDSLSKEDQQIFLDIASFFIGEDRDTATRIWGTVGIQNLENKCLLEVDRENKIKMHDHIK

ELGRNIAADTESMPLRLWRRTSNEIDDLLDQSSCMMTRVRGITMVDRFNFYGNKSDLFMT

VVSTESSVIEENECESSVTFLQTLEFQSEYFTGMSPEMSLHMNSEMPWYQKWTDEVLGNC

MRHLVNNSPTCGILNLRLIATEDGHLERILKRARSPHLLWLRWYKCPYSSLPSWIPMENL

RVVEVAGSKLKILWARESQVPFELRELNISAPLSSIPKSMGQLKLLEKIVIFHALLKTLP

DEFCNLCSLKHLELRECSRMISLPESFGKLTNLQHINLACASALQMLPLSFGKLIRLKHL

DLRNCGNLTFSTGTPGNISMLEYLDLSNITTHWNTWTFQAVRK

>LL_017190_T.1

MLVGCISKMLKKTELRVAQYPTGLDEKLKHFEDTVLLPQQQSGKPQIFGIVGLGGVGKTT

LAKEFFNRKNSDYPRSCFLSDVRDAKGSLNLLQTKLLKCLLRPLKQPQPVDSVDSVDEVE

GALIEPLKSSKALIIVDDVDDVNQVDALLPIQNHDLCSNSLILITSRDRQVLRSSRVEKS

SIYQLTGLNPQHSRELFCSHAFNQPYPNPGFESLLDKFLDSCDGLPLSLKVIGAILYGRD

IADWKDELRRLQQILPSEIKERLKISYHSLNKEEEQIFLDIVCFFIGQKRDTAIRIWEGS

GWNGRLGFQNLENKNLVEVDSLNERIHMHDHIRDMGRDIADELELPRRLWHWTEDVIDNL

LQRSSAITVRGVRMALSEYHADDVYSTIRMRRLQLLDTEDALLNRILRRVEFPNLIWLHW

NKCPSSSLPSSIPMKDLRVLQVSGSQLNTLWQDELQVPLQLRELEIFAPLLNIPKSIGQL

KHLERIVVDHIQVDGNFVGVKITKLPEEFCDLHLLKDLVLRNCSEMMSLPKSFGHLSNLQ

HIDLAGCIKLKNIPNSFRKLTKLKYLDLCRCNNVTLWRETLGKISTLQYIDLSDCKKIEI

LPQQVAHQRSLVTLKLRGTDLKELASAIAELRNLEVLELGSPLLDKLPPSLGDLRNLKEL

KIWDCKKLKCLPPSVGDLRNLQELTIQGSEELEYLPASLGDLRNLKKLLISSCKELKFLP

ASVGRLSQLTKLEIYQCPLNKLPLDSSIVKCMPWLRELSLEHLKISEVPFSEGVFPGLEI

LHIL

>LL_017506_T.1

MSFKRYNPLERTVSSNSRKYDVFLSFRGEDVRKTFVDHLYASLENAGVNAFLDSEKLEKG

DEISPTIEHAIENSAIRMPIFSRNYAESHWCLEEVSHMCKSGGHIIPLFYDVSPTEVRYP

DRGAFAEAFHQKKRRYTKAKINEWKKALAKVSSFSGWSRDDTFGFEAGLVKLVVQDVFKT

LQNVMPEEDCPDKPQDVAEEIPLEAMPLEIRNEPLRISKSVFGMEERMNEVIKLLKTDSE

DSLITISICGMAGVGKTSLAKAIHNHIYNKFDATCFVYDVRHKAQHTNGIAKMQRQILKD

LIKFKDKVNSEAHGKSLMRDRLRSIKALVILEYVGDSKQLEALRGDWYGPGSRFLVITID

PQLLNESQVDFVYEMQGLDRDHALQLFSWHAFLRDRPGEGYEELSQRAVNICNGLPFLLE

MLGAHLYNKDITYWVEACQRLEDLVHQDKYAILRLCFDGLNSEQKEMFLDMACLFIGRKR

ENVLSFWKASHCDSNKGLMDMILKSLISLVEEDKFAMHSELRDMGRAIVTEESEELGERS

RLWKPQQAELVVMEGKGTEKVRCLAYLQQEVTFQTTNLQTMCNLQLLWLDGTLIEGDFAQ

MPQEIKWLRWENCPLKCLPCEWNMKHLAVLDLSFSKDIEAVWTPFSDEEVINFLYRE

>LL_017650_T.1

MAYPTPSCSSFPQTHSQIEMENQTSRPATSVGASSSSIAADSNILYDVFISHRGEVKKTL

ASHLYHGLLSRGLRPFLDQQELQEGLNFPSQLEAAIRSASVHVAIFSPNYAESQWCLHEL

VLMLESRAPIIPIFYHVKPAELRWTDQSKNGVYAEALHKLQMKKTYDPQTHQDKSRYDSA

TIQNWRDALSSVANISGFDLDGPFNG

>LL_017672_T.1

MLESKESGRSAIIPIFYGVQPADLRWTLGKDGVYAKALAGLQEKKTTDPQTHQEKPRYDS

DIIEKWRNALSSVSLISGFDLQDYNNGVEWRLVDEVVQLVLKKVKKPQLDVSKYPTGLDQ

KVEEFEELLHQQSGGTKVIGIVGLGGVGKTTLSKEIFNRLRSNYEKSCFLFDVRERSRNS

LHSLQSMLIKELTGLTVQISSADEGPEKLRRYLSSSQALIILDDVDHIKQLDALLLPAKA

VLPTSS

>LL_017673_T.1

MASSSSSASTAYANNNYYVYDVFLNHRGPDCKEPFATDLYHRLRKHGLRVFLDKPEMQQG

DGLTSQIEGAIRTASVHIAIFSPRYAESTWCLKELVLMLQSGATIIPVFYGVHPSQLRWT

HAETGVYARSLRELEEKKTADTQTGQEKLRYDSKTIGEWREALSAAADISGFSMGSICDG

NQSQLIEQIVEGVSKKVKKPLHVSKYPIGLDKTVQDFERAMLLQPQSEQTRVIGIVGLGG

IGKTTLAKEIFNRQLKNYDRSCCLSDVRENSLTSSQGKLLQDLAQWEGQIYDINEGIENI

KKYIRSTLRAFIIIDNVDHIKQLDALLVPLKDTIHSGSLILITSRDKNVLTSSGIVKSSI

YNLRGLNLPHSQELFCWHAFNQPYPVIGFEEEVGEFSVACGGLPLSLQVIGALLHGKDDL

QYWKAQRCKISKIPPKDILGRLKISYDGLDEEEKHIFLDIACFFIGEHRDTAIRIWNGSG

WEGWLGFRNLEHKCLVEVDSQNRITMHDHLRDMGRYIAKKEPPERLRRLWCPTDNIFNLS

EQSPVRGISMVPNDGSELRPQSFKNLEQLADSLQLLRAIGDCVKNILSLTQSLPLIWLCW

YNCPYTSLPPGIRTDNLRVLQIRGGRLKTLWPESQ

>LL_017720_T.1

MAHANRTPAPDAPSSSTASTSNVDNDSYVFDVFINHRGPDVKTTFATRLYESLCKHDLRV

FLDKPELQRGGNTTSQIESAIRTASVHVAIFSSRYAESRYCLEELAMMVDCMSKSKSTII

PVFYDVEPSELRRTRDKNGVYGQALFKHEEEKTFNSSTIEEWRNALFEASKIQGFDRKTY

YRGACAGVLQCLETIFELCNLPLVFRAKAFMGDIVEEVLKNVKRPLHVSEYPKGLEQKLK

DFETRVSLQQESGKVRVIGIAGLGGVGKTTLAKEFFNRQRSNYKRSCFLFDIRENSLTTL

QSILLKDLTQSSVKIRSHDEGKGKLRRYLSSCHALIIFDDVDNTQQ

>LL_017721_T.1

MAHANGTPAPDAPSSSTASTSNVDNDSYVFDVFINHRGPDVKTTFATPLYESLCKHDLRV

FLDKPELQRGENTTPQIESAIRTASVHVAIFSSRYAESRYCLDELAMMVDCMSKSSSTII

PVFYGVQPSELRLRRTRDKNGVYGQALFKHEEEKTFKSSTIEEWRNALFEASKIQGFDRE

TYYRGACTGVLQCLELIFELCNLPLVFRAKAFMGDIVERVLKNVKRPLHVSEYPKGLEQK

LKDFESRVSLQQESGKVRVIGIAGLGGVGKTTLAKEFFNRQRSNYKRSCFLFDIRENSLT

TLQSYLLKDLTQQSEQIVIRSHEEGIGKLRRYLSSSHALIIFDDVNNIQQLDALLSPAKD

VLNTGSLILVTSRNKDVLTSSGIPDTSIYGLTGLNPLHSKELFCSHAFGQSHPIVGFEKV

VGEFLAVCHGLPLSLKVLGALVHGKNDLKHWKDQLRKIIKILPGDILIKILPDDIQRILR

VSFDSLEEKEQHIFLDIACFFIGENSDTAMRIWDDGRLGLWKLEKRCLIEVDSENRIRMH

DHLRDLGRYLAEKEPPGHELRLWHPPDNFLHNISGQSPVRGISLVHGNDPEQSFEIFLGS

CNLSRLQLLMAEGDFVEWVLRVGQFRNLRYLRWQTCLKSSLSSSIPIKNLRVLYIEGEHL

QTLWQDESQVPLQLRELYIDAPISKVPKSIGKMVHLEKIILSSSYLETLPDEFCYMHSLE

HLELNGCQLTLLPDSVGRLTSLRSLHLTECSTLQALPDSVGKLTRLQKLKLRACSALQAL

PDAVGGLTSLQDLYLSSCSALQALPDAVGGLTSLQHLHLGGCSALQALPDAVGGLTSLQN

LYLRGCSALQALPHGVGGLTSLQVLDLDGCSALQALPDAVGGLTS

>LL_017821_T.1

MAVSSVTAASTSAAVHFNNKSYAVFINHRGVDVKTTLARYLYLRLLHNRLTAFLDEGEMQ

PGFSIKSQIAHAVNTATVHVAIFSPRYAESRWCLDELVDMLKSKAPIIPVFYRVTPAQVR

HAQGSYGEALQTHADNGRHDSNTIDKWRNALEEVANNSGFELKTGSGTSEEWELADKMVQ

HLSEMIVMDERRKDFEMIGFDEKVGLDKMVE

>LL_017971_T.1

MHIDNSYQGIETLKKHLSDFHILLVLDDVDHVDQVNALLPVDFLRHDSLVLITSRDKNVL

LSSGVQESSIYRLTGLDRERSRELFCSHAFCQPHPLSGFENLVAQFLTACSGLPLSLKVF

GGLLYGNTDKSYWEDELERLEQILPDEIHKKLQISYDALQRDEQQMFLDIACFFIGKERD

TVIKVWDGLGLKGSRGFQSLLNKSLVEVEIMEHSYYFDERIYKNCIRMHDHLRDLGRDLA

KDSRLPCRLWCGTKNIDDFLQQSFEATEVRGIRMFSSGYGDNEECSGISGYGDNEECSGI

RMLPSVYGDDEVCSTYGDNKVGYGDNEEGYGDEEVCSRYGGNEEGYGDEEVCSRYGGNEE

GDGDDEEWTYFQRCKMKKLQLLDIDMREDLLRCILRGVESPKLIWLRWHNCPSSYLPPCI

PMKKLWVLEVDGKELETLWPEESQAPVQLRVLRIQAPLSNIPDSIGRLKHLEQVVVV

>LL_018070_T.1

MADLEIASTSPHSTTGTSSPPSTNYCSYDVFINHRGPDVKHKFAINLYHRLLSHGLQPFL

DKEELQKGEYIAPQIEGAIRTASVHIAVFSPTYAESKWCLDELVLICKSGAITIPVFYNV

KPSELRWAGRYAQSLSIHEQKGRYDRQTLQSWREALKDVSYRVGFELEAYDSDEGKLLDN

VVRCVLKERRKAALNVATYPTGLQEKVQDFEKTVSLHQKGEKVQVLGIVGLGGVGKTTLA

KELFNRQSSHFGKSYFLFDVRETAVTRSLNSLQSKLIKGLTKWDVQIDSVNEGIEILGKY

LSSCHALVVLDDVDQMHQLDALLPIKDVLHSDSLILVTSRYKDVLTSSGIAESSIYKLTG

LNERHSQQLFCSYAFLQPHPSLGFEYLVNEFCMACDGLPLSLKVFG

>LL_018140_T.1

MAHCTIAPSSSSAACSASRPFDVFINHRGPDVKNAFARHLYRSLNSRGLRVFLDQPELQA

GQTIFSQIQASIQAASVHIAIFSENYAKSKWCLDELCLMVESEKTGATILPVFYNVKPEH

LRWTGTDKDGPYAKALRKHEEKQRYDSQRIQSWREALSHVADLSGFVLEACNGDEAELLE

KVVQRVLTKVTEPALHVAKYATGLGEKIEEFEKTV

>LL_018141_T.1

MQVLDPKSLILVTSRDKHVLISAGLAESSIYHLKGLDTPHSQEFFCWHAFFQPRPRPGFE

NLVVRFLNRCDGLPLSLKVFGAMVCEKDESYWEEKLNEFQKLPPKIMASLKISFDALDPE

EQQIFLDIACFAIGDDRDMWKRIWGGLAGLQTLEDKCLVEVDSQNAMKMHDHLRDMGRNI

AEQLSMP

>LL_018144_T.1

MASCSSTASHSNNNQWYDVFINHRGPDVKETFARPLYLRLLNTGLRAFLDKEEMQPGYNF

PCQIKAAIGTASVHVAIFSRRYAESNWCLDELILMLESKAPIIPVFYRVTPGEVRWTHGK

DGTYARALQQLAQKKTYDPETHKKKLRYDSNTIEKWRNALSSVADNSGFELETCNG

>LL_018192_T.1

MARLHTSSAHCSSDTPSTSTNYPSYDVFISHRGPDVKNGFASHLFRELYVHGLQPFLDRE

VLQAGDLLSPQIEAAIRTAYIHIAIFSPGYADSTWCLDELLLILQKPRDTIIPVFFNVKP

SEVRWTSKDGQYAQALNKLEDKGRYDRQTLENWRKALNDASYRVGFELEAYNGDERKLLD

KVVQCVLKKRRNIGLNVARYPIGLEDKVEDFEKTVLLQQQQSGKVKVLGIVGMGGIGKTT

LAKQLFNKKSSNFGESCFLFDVRENATRRSLNSLQTKLIKDLAHRDVQIDHIDSGVEKLK

TYLSTSHHADADPLHALVVLDDIDHVDQLHAFLPIQDVLRSDSLILVTSRYKDVLTSSGI

AESSIYNLTRLNEQHSQELFCLYSFCEPYPPPEFKDLVDQFCRACHGLPLSLKVFGGLLC

QKNDKSYWEGLLYKLKLPKEIQETLRLSYDALDEEEKEIFLDIACYFIGEDRDMAIRIWQ

GSGWDGPVRLRNLQDKCLVEVDGQNTIKMHDHLRDLGRQIADEKKSSGVIPHRLSHLVEN

IHDLWQDYSVITEVRGIRTASHPNDNADSGVLPWFKSWCDEVLEKCLQRKPRLRKLQLVA

TKHDDLKNILTRVESPNLIWVRWNNCPYSFLPSWMPMKNIRILELQGNKLKTLWRTESQA

PLELRQLNISAPLSKFPKSIGRLKHIEKIVLRWTHLKSLPDSFGCLTSLQHLDLSRCERL

QGLPDSFGCLTSLQHL

>LL_018269_T.1

MAASSSTDAHPHNNQWFDVFINHRGPDVKETFARSLYLRLLSKGLRAFLDKWEMQEGYNF

PSQIKEAITTASVHVAIFSPRYAESEWCLNELVLMSESKAPIIPVFYGVPANEVRRTRGN

YGQALQKLAEKKTHDPQTHEEKLRYDSDTLESWRNALSSVADNSGFELDACNGELELLDK

VVERLLKMVKKTDLHVAKYPTGLDEKVKHFEDTVLLPQQQSGKPQFLGIMGLGGVGKTTL

AKEFFNRKKSEYNKSCFLSEVRDHAVKGSLTSLQTKLLNSFDIRLDQPIDSVDEGKGMLI

EPLKSFNTLIILDDVDDVNQVDALLPIQTHDLRSRSLILITSRDKHVLISSRVEKSSIYR

LTGLNRQHSAELFCSYSFSQPYPDPGFESLVDKFLDSCNGLPLSLKVFGALVYGRDIAVW

EDQLRSLHQILPTEIKERLKISYEVLNKEEKQIFLDIACFLVGQNRDSAIRIWDGSGWNG

RLGFQNLENKNLVEVDSSNEIHMHDHIRDMGREMAEESELPRRLWHWIENVIDGLLQR

>LL_018270_T.1

MASSSTSISMAASTSSNNSYHVFINHRGTDVKNTLASHLYHRVSAQGFRVFLDKKELHVG

DSFPSEIIEAIRTTAVHVAIFSPGYAESEWCLDELVLMLESGAPIIPVFYNVAPNELRWT

QREDKLYSKGLSKLKDKKTLDSETMKEKPRHDSITIEKWRNALSTVANISGLDLHKTCNG

DEGELVEKIVRLVREKVKRPGLSLMTGYPIGLNEKVESFENKVLKNQQSGKALVVGIVGM

GGAGKTTLAVELFNRKSSNYDKSYFLHDVRAGKSSLPSLQTKLLKGLFQLDRPVDNIHEG

MRTLTELLSTSHSKLIVLDDVDDKGQVKALLPDVKSVISSSTSFVLITCRDIDVLRGSGV

EESLIYHLDGLNPQYSLQLFCLHAFSQPHPPPEFKDRVDKFLEVCDGLPLSLEVFGALLH

DVKDMSLWDAHLDRLRKTLRSDIKERLQISYEALSPEDKEIFLDIACFFIGENRDMAIRI

WDGSDWQGSLGFRNLQNRCLVQVDSENRIKMHDHLIDLGRANAEKSGNGEKSASRHCLWP

PTENIHDLLQKTSIRDEVRGIRMRPSDCNDNADLTLPSEFIDCDDEYRSFSRYKMKKLQL

LDIEGGLLDRVLKRVHAPDLIWLRWIKYPRSSLPSWIPMKKLRVLEVQGNELETLWESES

QAPSELRELKIRDAPLSKFPESIGKLKHLQHIYVNLRKGGINVETLPEQFCNLQSLMTLE

LLNCCEMKSLPELFGNLTNLEHIDLMGCCKLERLPNSFGNLIRLKYLKLSKCSNLTISSE

TLGNILTLEYINLSYCSKIEEMPPQIALQRSLGKLYCMDTAFTELPRAIGNLTELEVLYL

GDDSLKALPHSLGDLRNLKQLTLYCSQLKCLPASFGVLNQLIELRLRCPITELPFKGRVD

SSSDNCMFPRLQYIFLEVVRISELSFDEGVCPNLQYLMVHNCTLIKVGTLPNTLKKLELY

RCKLSMSIDLRGLAKLEMLHITDCIGPKSILGLEELTKLRVVEILSPSSQLENILELPSF

KDLRLLERFRACDLRSIQVSEQLTKLRELDVSGCSKLEVLPGVEHLRSLEKLKASACNNL

RSIQVSEQLTKLRELDVSGCS

>LL_018282_T.1

MAASTASISSSTAAHSNNNQWYHVFLNHRGVDVKKTFARSLYLGLLNEGLRAFLDQDELQ

PGYNFPSQIHHAIRIASVHVAIFSPRYAESYWCLNELLLMLESKAPIIPVFYRVTAAEVR

WIPKDGKYDRALKKLAEKRTEEGELRYDPNTIKNWRNALSQVADNSGFELQTCNGEELEL

ELLEKVVKYLLKMVKMVKKPDLHVADHPTGLDQKLKDFENPVLLQQQQSGKPQILGIVGL

GGIGKTTLAKHFFNKKKSDYLKSCFLFEVKENAAKNSLILLQRQLLKSLIGFDKQVNSVD

EGKEMLKEPLKSSNALIVLDNVDDVDQVTTLLPVQAHELCPGSFILITSRDKRVLISSRV

QNSSIYALTGLNREHSKELFCTYAFGQTYPLPGSECVVDKFLESCNGLPLSLKVLGANLY

GREISELEDELGSLPTQVKECLKISYDALNKEEQEIFLDIACFFTVLNRDTAISMWNASG

WKGRRGFQNLQDKCLIEVDSLNVIHMHEHLRDLGGDIAEKSSPFRLWRWTKNVIYDLSQQ

SSNGQGIAVRGIRMDLTEYNDPHMER

>LL_018283_T.1

MGYTFNSGESELELLLNKVVKSVLKVVKKPDLYVADHPTGLDHKLTDFEQTVLLQLQQSG

KPLILGITGFGGIGKTTLAKEFFNKKKSNYFKSCFLSEVKEKAEKGSLISLQIELLKSLN

VLDEQVNSVDEGKGMLIKPLKSLNALVVLDDVDDVDQVTALLPVQPHQLCSGSLILITSR

DKHVLISSGVEKSSIYKLAELNRQHSQELFCFHAFRQTYPSRGYE

>LL_018334_T.1

MDVLTSSDIPDSSIYRLTGLNRQQSQQLFCSHAFHQSHPVLGFEQEVNKFLDVCDGLPLS

LKLFGALFSGNRDRTYWKAQLCEISEVLPDDIHRSLKISYDSLNSREKEIFLDIACFFIG

EDRDTVIRIWDDGSSWRGWLSFRNLENKCLVEVDDKNRIRMHDHLRDLGRDMAKNEHGCP

LRLWRPTEDLNSQSPVRGIKNFGECSRLVADTSKLQLLSAEGKCVESILRVGRSPPLVWL

CWYNCTESSLPSWIPMKKLRVLKINEDDLGLETLWQDESQVPLQLRELDIAATHLLKIPN

SIGKLKHLQKIVLKPGKSDDMHLKTLPDEFCNLQSLKHLELNHCSRLKLLPDSFGNLTNL

QHIMLLSLYSLERLPDSFGNLRNLQHISLSYCKKLHMLPESFGNYFPDLQLIDLSSCFNL

EMLPNSFCKLTNLQHIDLSYCYTLKMSPNSQTFGNFRQLKYLSMKECIGLNISSETLGNI

TKLEYLCLPYCNDIEVLPPQVTHQRSLLELSLLCPGLKELSSDIRELSNLKRLHLGSKIF

ETLPESLGHLRSLEELELHFCNRLKCLPYSIRQLTQLKKLTMVKMANLALLGIEECPSSK

PPFRNVGGEIEILTDRRGRRGLHLCLNIQHLKIENCVQLVEVRALPTTLIKLELEGCSSL

VKIRGLYGLATLQMLKITLSQKLEELTGIETLASLETLSLGDLKKLNSIQGLGQLKQLRT

VDIIKC

>LL_018347_T.1

MAFESAVVSGFLANVASAAIVGGVKAVKDAFGWEYPKHQDLQIVPDSGFVGRGIQSARKK

LQIWLNEPEARVIGVSGMGGVGKTSLLQLIHNDCKEKVSTNFDFVIWSTVSQIFNIQSLQ

DTIAESVKLDLKGVSSFDTRKMKLYASLENKRFLLILDDVWSPVSSYIGRRVESNC

>LL_018382_T.1

MPDPGFVSGFIGVGIDQVIRQILHRINIAVRCKKELESLRDLVVKIQPIVEQIQKYRQEI

NIKKKDMVSPVSGWLKDLEALLQEASQMTQRCTIQSCNVVFRYQTSMKISGLISRINKHL

NLSPLVGLAQIPELLDEQNQKLGQIKKMLEALPSSSSSACTSHGVLAPTGCAQMKLIDEP

FVVGQQKAFPTLESLVIDEAEAKNIGVLGKGGSGKTLILKRLFNSEKVRNLFSGNLLLWL

TVSQSPCFKNLRNELRIQIALQNGVDLGHNLDEEGVKLWLNDSLQKSRNFIFFLDDVWGQ

YAAELLEELGVQRPVRDHSKSKVIVSARDRTVLLKMGVADRYTITMEDLGCDESW

>LL_018423_T.1

MAKELAIGISCQVGGEFMNKGCVARILQGIDDLIHKDQNKQFLQDTLERMKAILHDIIST

AFPHQYTAPSETINYCLKRIGEAIKKANGLIDAQPQPEEGSSSSTSNVLIDIESQQQQQQ

GRRWLPNLLQAKDILLQAKDNLLHAKKIGELNAELKELLQDLERAFSMVSHARLLMVLGM

VLQLLAWRQSSASALPQETDVLLQPMAEAGLLGRSIESGENRLQFVPASLPHSSARPQSS

ETQLPIVLATPSQLSVMPQSSETQFQIDPATLPQSSASVPSQETESGFVGEAIKSAETQL

KTWFETPRVRVIAVYGIPGVGKTTLLESVYNFYQHTNVFDCVILLTVSQSYEIRDLQDRI

AKIVWQDRIAKKLELNFFDRDADTRKRMLSKSLIEKKFLLVLDDVWSALDLKELGVAFGT

DKGSKVVFSTRHRDLALKEAEQYINMEPLSKDEGWELFERVAFKGGDVPEELEECARKIA

EECNCLPLAITVVAAVMRGKGTKVDDWKCCLSLMEKADPAFPETNPSVSKELYQTLKWSY

DDLSTNLQNCFLYCAMFPEDEQISVDELVRMWIAEGFCQSNEETYSMDMAIGYGYVKFLI

DRSLFQSGNFEKQAMDRIGNKPVNVWKNRFITVHDVIRDVAIYIAEGKQEEKHICRSGLQ

LKRFPRNRTQDCKRMSVRRNKIESVPEDFPCKQLVSLILSQNPLARNVGGSFLQNLTSLR

VLDLSNTQIESVPISLNQLRVLDLSNTQIKSVPTSFPQLQFLTLRGTCIEELPSEISNLS

DLQFLDISECKSLASLPTEISQLTSLKRLHLSMGMEKAVKDPKNIMALMGLKHLIELHLY

MFFKFENHEIMGTIMGSWVEMRHLYLHYNHMIPSKCNLPEGMQNMKNLESLVLYDYRGTR

LPNWVCEFQQLQRLEMDSCGNVKELPPLEKLPRLKFLKLRKYLDVRNLGIGTRSGFPELE

MLHLSDMPKLRGIGVLEEGTLPKLHVLKIKKCPLLKKLPIGMEKLPNLKALYGHRISWWE

KIIWEDHNMKICVQKLFKEIQMGN

>LL_018434_T.1

MKLIDEPFVVGQQKAFPTLESLVIDEAEAKNIGVLGKGGSGKTLILKTLFNSEKVRNQFS

DGLLLWLTVSQSPSFKSLRNELRTQIALQNGLDLGHNLDEEGVKLWLNDRLQKTRNFIFF

LDDVWGEHATELLEELGVQRPVRDHSKSKVIVSARDRTALLKMGVADRYTITMEDLTGDE

SWKLFAYHAFPYNHGNTPANIDEDVAKRVCHKCGGLPIAIKVVGRAMAGSTHPKQWESAL

QTLPNANSVYDCLRLSYDALGNEDVNMQLCFLYTASACLESQIIHTELLFPLWVGEGLLA

RKMLQYENPLSYDPLEMGRIYINVLADRCLIEPIIRDFEGQVIMFRIHDVLQDLAIRVAE

EEEGVYRRVGRYLTTLNENELSERTRIFLSVNNLSSLPMSLRAPQIRSLLMKSNKDLTEI

PKRVIGSMISLKVLDLKRTSLKALPDSVGCLKQLVYLNLFATPIKKLPASFTNLVNLEIL

CLSGTRITELPYDLHKLASLKCLDIRYCKDLQYLPC

>LL_018450_T.1

MSKELFNRKLSEYPKSCFLSDVRENAKISLHSLQSKLLKGLGQLDRHIDDIHQGTQMLLE

FLSSAKVLLVLDDIDHQDQVDALLPDRDALHRDSLIVITTRDKDVLARSRVEEASIYKLT

GLTPQHSRELFCSYAFCHPHPLPGFVDLVNKFVNACGGLPLSLKVFGALLFRKEKYYWED

RLDRLHQVELPSDIQNTLKISYDALSGDEKQIFLDIACFFIGEKRNIGIGLWKESRWKSD

FQNLECKCLVEVDSRGYIRMHDHLRDLGRQIGEEQLPRRLWRSPTENIDDWLQQQSAVIT

EARGISIAHMDSSGISIRNHDEP

>LL_018465_T.1

MAHASASASATRHVYDVFISHRGPDVKKTFASHLYRRLLSSGFRVFLDQPELQRGENSET

QIEQAIKTATVHVAIFSSGYAESRWCLDELVLMLKSTESGKATIIPVFYNVKPSDLRWTD

QANNNNGRYTEALLNHEKKGRYDQERLRTWRKALSDVSYISGFELEACDGDEGELVDRVA

QQVHKRVRKAPLEVATYPTGLDEKLQDFERTVILEQQQRRGEPKAVGIVGLGGVGKTTLA

KLFFNTKRSDYQQSCFLPDVRDAAARRLLPSLQSKLYKDLTGRDQLMDNVDEGIAMLKDR

LSCGPALVVLDDVDHVEQLAALFSPVKDVLPGDSLIIVTSRNHEVLAISGIRASSVYKMT

GLDE

>LL_018466_T.1

MLKSRATRRATIIPVFYGVEPFVLRLRGKDDEVYAEAMRIHEKKNRYDQQIITDWKRALY

EVSLISGFELKAYNGDEGELVDEVARQVLKKVRKEPLPVATHTTATIHMAETNPNP

>LL_018467_T.1

MAHHRTVPAFSTTHYDIFISHRGPDVKKSFAGHLYRSLSSRGLRVFLDQPELEPGDKIIS

QIEAAIRAASFHIAIFSTNYAESNWCLDELVLMVESEKSGATILPVFYNVKPSDLRWTGK

DKNGPYAKALLKIEEKQRYDSKTIQNWTEALSHVADISGVELEACKGDESELLEKVVKRV

LKRVQMPLHVAKYSTGLPMKLEDFRRTVLSEEEEERVGAKVVGIVGVGGVGKTTLAKAFL

NSERSNYEGSSFLFDIKEYAVKFSLNSLQSKLIKDLKGIDIKIERWEEGIVILEKNLSDC

HALVVLDDVDHAHQLDAFLPIKRILHPKSLIVVTSRNKQVLRAAKIAESSIYQLKGLNRP

HSLELFCWHAFFQRHPPPEFEDLVDGFLNACDGLPLALEVFGNFLCKHDKFYWEEQLSGL

HNLPPEIEVTLKASYDSLNQEEQQLFLDIACFCMGEDRDKWIRIWGGSGWNGLVGLRNLE

NKCLVEVNSKNKIGMHD

>LL_018477_T.1

MVVKEVLKKVKKPALNVAKYPTGLDDKIKDFENTVLLPLQQSGKPRILGIVGLGGVGKTT

LAKEFFNRKSSDYSKTCFLADVRDNANKSGLILLQKKLLKRLNVSDDIDGVDEGIGMLSK

HLSSSGTLIILDDVDHLNQVDALLPQQSIHSDSLVVITSRDKDVLARSKVENTSIYKLTG

LDTEHSQELFCSHAFNQTYPQPGFEYMVLEFLKSCNGLPLSLKVFGALLYGKDISAWEDE

LESLRQILPRDIQQSLKISYNSLNEDEKQIFLDIACFLRGENMDMAIRIWDGSCWQGRRG

FDTLRNKCLVEVDTENNIQIHDHLRDMGRYIAEEPGLPRRLWRWTKNVTESLLEQSLNGQ

VISVRGIRMAYTDIDDDDVFSCIPMMMGLQLLDTEDVLLERILTKVQRLNLIWLRLRGWT

EFSSPSLISMKNLRVLHVDASNFTTLWEKESQAPIQLRELEVRATLSNIPESIGKLKHLE

RIVLHVNCSNLTKLPEGFCLLQSLKTL

>LL_018489_T.1

MIFLAKVVKQFKERPQLKEGILHDLIKDATQLIVEGSTKCCKQIDSSKISRIIKASVNKE

DLDNFRKEIDTMYWKINSQMGISIFDANTRNKEAPQSRRYPSHAVGIDEPVKEIIDLLDW

GTENPAIAIMLHGFGGTGKTTLADAVFSLVNIQGCKYSSVRLFQNIASTPNIIELQRSIL

EDLTEPGETVPKFTKHEDGQRELGRLLEKVPAFIYIDNVLKQDELGQLLPKDFNKSKKVR

FLLTARDDNVRRAYKMKTKVFTVKGLSDTEAMHLLKREIYDDMDDKEKQIDSSQLNKIVQ

SYGGIPKLLEVVGGFLRAEDNKQNAYEILMKEKEKWDSEIGNIESYLFAYDHLPDLLKDP

FLDICSFFKGWNWDTVADIVGETELDMLKSRALVTNDTNGVVIVHDVILSLGQRKTKGTR

FTFNNAMEFKELLDKKEISEIKGIMLSENNKDLLDISASKLDLMNNSLRVLALGNFIKVQ

GKCNKTFEKLIYFQGAIPYLPFDVSKSMGLRYLKYEPKDLNFLEIPSSLRHMEFDGRLHS

HAFEISSRDLQQLGNLRILRLTNFAELKKLPEDLGDLLNGLRELTLSYCKSVEELPGSIS

KIQSLRVIRMDYCSSLRELPEDFGLLSSLEELNLQGSSNLKALSNGFEKLTALRLLDVSF

CQRLLDLPHGLGNLTSLYLKNCSQLRSIPE

>LL_018503_T.1

MGASFSTASSASASTSTSTFATSTFTSTSPTASASTSTYAAANPNHKSYAIFINHRGPDV

KATFARPLYLRLLQNGLTAFLDEEEMQTGYNITSQLEHVVRTATVHVAIFSPRYAESDWC

LNELVLMLKSKAPIIPVFYRVTPAQVRWTLGDYDRAIQTHEKKGRY

>LL_018504_T.1

MVKTPDLQVAKFPTGLDEKMKDFEDIVLLEQQQSGRPQILGIWGLGGVGKTTLAKEFFNR

NKSNYRFSCFLSDVRDNAEKGSLISLQTKLLKRLTCGLDNPIDTVDEGTGMLIKHIKHLK

YSNASLQTGFYQPGNCVDEGKGMHIKRLKSSHTLASNNVSNVLVILDDVDRVDHVDALLP

VDLPSGSLILITSRDRHVLIVSKVPESSIYLLSGLNTKYARELFCSHAFNQTYPLPKFES

LVAKFVEACNGLPLSLKVLGAHLYGRN

>LL_018508_T.1

MYLRTSGEQYKKLSDQRDFSAVLREIRRVKPLCTCSAVRSTRRSAIPICESMAGLNLASL

ASPSTSSANNNSYVYDVFINHRGPDCKETFARYLYETLSEHGLRVFLDQRELQEGESIWT

PQIQSAIRTARVHIAIFSPRYAESAWCLNELVLMLESRATIIPVFYGVRPSELRWKQGET

GLYARVLRMLPWVLCILCFTREESESGAYARSLRTHEEKKTIDPQTGQKKPRYDSNTIRK

WTETLAQVADISGFDKETYNGDERQLLKEVVEGVLKKVPREYHVAKYPIALDQKVEDFER

KMLLQEQSGRTRVIGIVGLGGVGKTTLAKEIFNRQGSNYKRSCFLFDVRDKSLNSLQSML

LKDLVQLNEQINSIDEGKAKVTRYLPSCKALVILDDVDHIDQMDALLLSREDLLLSGSLI

LVTSRNKDVLTNWGIDESSIYSLTGLVPQHSKELFCSHAFGRPHPDVGFDEVVEEFVVAC

DGLPLSLKVLGALVRGKNDLNHWKEQLLKISEVLPSDIQRRLKISYNSLDEEEKHIFLDI

ACFFTGEDRDTTIRILDGSGWRGCLGLRNLENRCLLEVDNENHIRMHDHLRDLGRSIAEK

EPPDCLRRLWRPTDKLHNLSDLLPVRGISMAPNYGSEWRTPQSFQNLTEFTCKLRLLKAE

GDYVESILRIAQSPQLIWLHWEDCRYSSLPSWIPTENLRVLRINGEHLETLWLHESQAPA

QMRELIIGADLLNIPKSIGNLKHLEKFSLYGNPAALVSLKTLPDEFCNL

>LL_018561_T.1

MAEPPVVGALIETFTSMLADKVLEEVSLITGFKNDFEFLCDELVSVKLLLDEVGQNSTSR

SMSNWLEKLEYFLIDAVDIVDECRTNKFGKSIFSNFEKLIFRYKMGSRIRTLKERINNIH

GSAKYLKHLMSVLQVNALNAHNLEDKRERFSALLRESVHIVGIEENINKLTKFILNEKGP

QVIAVVGMGGMGKTFLVDCVVKSQKVQENFDHVVWLEVPQNFDMKDMSRRIKKKMKLLCD

QDENDKIIEELQRSRCLFVLDDIWKKDDFNQFHWLLGKTKIVVTTRDKGVMSTISPDHEP

KVYELEKLSSENSKKLFCIHAFPDRGVTQQSPAYTNEPPKEITQHSTASTDIQPPKELRN

VTDEIVLKCGGLPLALKTIGASMARVRRLPNDWESILKSLNEAEAMSGKVWASLRLSYDT

LPYHLKLCFDYCSCFPKNGRIKSEYLVNAWIDEGFIPQTAEYPYDVGFSYINELIDRCLL

EVSKVGGDGRVKYCKMHDLLHDLAHAESHKKTKCLLNPAKELKEFPACESRLRRISLIEN

NIPIIDEPIKCPGIRTLLLCNNLSLVSIKAIFFADMKYLAVLDLSCTSIDSLPDTVGNLK

HLKFLNLSQTKITKLPRSLAELRSLQFMDVSQCEHLREMHSGIGEHKSMLHLNVKGSGKL

ESLPVGISKLIYLHTLKGPVFKREREPTANALQLCALVKREKAVNAKVLQFGDLKRLTLL

QHLSLTLNAPSSNGIFQLEEGIFGSLTNMRALSIKYIDSSEDASGSLCFPEEIQFMRRLE

ILHLKNCALQDWIFELENLMELELYGDNGSTSVYRGLANIPNLRKLKLSTNDNDKCVEFP

QEFGKSGAFPKLENFVIKRFNKMENFPSLQDGALPMLKCLQMKYCYKLKDITKALQRPSS

LLEEIRVKGCPVWQNGIWTDKVTWKILKDHPIKLTIDGYTISWALDQYQNSKLFGNDLPR

KVLRSSMLIFQTAFRFKEAGTSSAGGVAVDRTLQNVSSVECRQQQITMDEHKEESVAGVT

GMTKGEPVSSSNSDEIPNQGNFQAQTETLAHA

>LL_018575_T.1

MKLIDEPFVVGQQKAFPTLESLVIDEAEAKNIGVLGKGGSGKTLILKTVFNSEKVRNHFS

DGLLLWLTVSQSPCFKSLRNELRTQIALQNGVDLGHNLDEEGVKLWLNDRLQKSGNFIFF

LDDVWGEHAAELLEQLGVQRPVRDHSKSKVIVSARDRTALLKMGVADRYTITMEDLTGDE

SWKLFAYHAFPYNHGNTPANIDED

>LL_018576_T.1

MFSENFAESQWCLDELLLMQESGAPILPIFYGVKPAELRWTDQNKSGVYAKALQQLEGKT

TFNPQTQQIEPRYGSATVRKWRTVLRTVSDLHGFDLEEGPFNHDEGQMLNKVVQRVGEIL

AERTRLYVAKHPTGLDDKVKDLETRVLRGKAQVVGIVGPGGMGKTTLAKEFFNRKRSQYT

KSYFLSDVRENATSLHSLQSKLLYGLTQL

>LL_018577_T.1

MLKRHLTSSHVFFILDDIDHADQLDAFLPVKDVLSSNSLILITSRDKDVLASSGVEQLSI

YKLTGLPRQHSLELFCSYAFHQPYPVKGFENLVAKFLQVCHGMPLSLKVLGASLYGNNDK

SVWGDILLKVPTGIQKILKISYDALSKEEQHIFLDIACFFIGKKKNGAITLWDGSGWNGS

SGFKNLEGKCLVEVDDENVIHMHDHLRDMGRDIAEEKLPRRLWRPTENIDNWLHQQSSAI

TEARGISIRHRDSSDKSVIINSSMLSWYQTKIEKVLGNRMMNILYTLYRFLGIGRLMKLM

YRCCRFLGIRNRV

>LL_018580_T.1

MAGLNLEHSHELFCLHAFHRPSLVVGFEEVVREFLDACQGLPLSLKVIGALLYSERPDLR

YWKTQLLKISAVLPSDIQSRLKISYDSLDEQERNIFLDIACFFIGEDKDK

>LL_018581_T.1

MAQPTSAQRASGVAASSSSFAPFDVFINHRGPDVKNTLASLLYHELKSRGLQVFLDRPEL

EPGHIIISQIKAAIEGASVHVAIFSPKYAESKWCLDELVHMIDSTGKILPVFYKVKPSEL

RRTDKGAYADALRKHEEQQRHDFQKIVEWKKALSHVADISGFELDACNGDEVELVDKVVQ

RVLKEVPKPPLHVAKYQIGLQEKLEDFERKVLSQLEEEGRQEVKVVGIVGLGGIGKSTLA

RNFFNRKRSNYSHSSFLADVRENEARKSITSLQSQLIKDLRGRDISIRSPEEGIGELEKN

LKSCHSLIILDDVDHLHQLDALIFPIRYVLHPKSLILITSRDKHVLRSAEITEPSIYQLK

GLDGPHSLELFCCHAFFHPHPHPLPQSKDLVDGFVRACQGLPLSLQVFGALLCQENDRSY

WEEQLNGLQTLPLDIQKRLKISYNSLNREEQQIFLDIACFSIGHDKDKWIRIWGGSRWKG

SVGLRNLENKCLIEVDAKNKIRMHDHLRDMGRNIAAE

>LL_018588_T.1

MGLPLALKVIGSSLRNEPPAVWENAKKKLSRGESISKHHREDLLHCLETSIDVLDDESKE

CFLDLGAFPKGRKFSVDSLLDIWVHVRGMEWQYAFVVLLELASRHLLNLTSDPG

>LL_018591_T.1

MASTSTSTSHGNRVYDVFLNHRGPDNRKTLASHIYHRLTDRGLRVFLDTEEMQPGEELTA

QIIHAIRTASVHVAIFSEGYASSPWCLDELVEMVKTKKPIIPIFYDVNPSELRWTQAGNG

RYARDLHMLERRTTSDPQTNKKAQRYGSDTIEKWRTALYSAAEISGFELNKFNGDDGLLV

NEVIQRVLKTVPKTLPNVAMYPTGLDVKVKELEAIVSSQQQSGSLARAVGIVGLGGVGKT

TLAKQFFNSHTSNFERSCFLSDVREKAARNSLISLQAQLLKELAEQNKQISSVYEGEDML

RCYLPSSRSYLICIDDVDHLDQLSVLFFPLKYTKSLILITSRNKDVLRSAGIEESSIYMV

KGLNTQHSKELFCWHAFKQSYPVKGFEQLVDNFLVACGGLPLSLEGIGKLLYGKNDLNYW

KAQSHKISKILPRYIKSTLEISYNGLDKEERQIFLDIACFFIGNIQRDTAIRIWDGSGWQ

GRLGFRNLENRCLVEVDSENCITMHNHLRDLGRELAENAEYPLRLWRMEDHLHNRPNHSS

VRGIHMVYRNDAEHFFENLADSCNMSTLQLLRAEGDFVERLFNVNGHFRHLLYLHWDNCP

YSSIP

>LL_018600_T.1

MAESSSAAAPSDYDVFINHRGPDVKKKFASHLYRRLLSYNLRAFLDLPELKRGDDFDPQI

KNVIRSASVHVAIFSSTYAESVWCLNELLLMLESKAPIIPVFYDVKPHELRWTWGKDGEY

AQAIQRLEKKMNYDPQTHEEKLRHDSDTVENWRKALSRVAGISGFELEEYNGDEGELVDM

VVKEVLKKVKK

>LL_018624_T.1

MVYDVFINHRGPDTKKTFAAALYRRLLSQGFRAFLDQNELQEGSSFTSQIAGAIRAASVH

VAIFSPTYSESSWCLQELVLMLESTAPIIPVFYKVTPSKLRYGEELRKLEMKMTCDPQTG

MSRPRYDSATVQNWRNALSSVADKSGFDLEGAFNGDEMEMLDKVVERVVKMFKKTQIFVA

KYPIGLDDKVQDFETAVLKQQHRGEVQIVGIVGLGGVGKTTLAKELFNRKRSSYNKSYFL

ADVRENARSSSLHSLQSKVMKGIIQVDLKIDSIVEGIEILRRYLQSSHVLLVLDDIDHVN

QLDAFLPVKDVIHSDSLILITSRDKDVLISSGLKAPCIYKLTGLPVQRSRELFCSYAFCH

PYPLRGFEDLVDKFLMASDGLPLVLKVFGTLLFGNNDKSFWEDRLYQGQVPSDLQKSLKI

SYDALNEEEKQIFLDIACFFIGEKKNTAVTVWDGSGWKGLLGIQNLESKSLVEVDSDNYI

HMHDHLRDLGRKIAEDKLPRRLWRPSQIIDDLSQQSSVSAQSFNISNFP

>LL_018626_T.1

MAYTTPSCSSFPQTHSQIEMENQTSRPATSVGASSSSTAADTNILYDVFISHRGEVKKTI

ASHLYHGLLSRGLRPFLDQQELQEGLNFPSQLEAAIRSASVHVAIFSPNYADSQWCLHEL

VLMLESRAPIIPIFYRVKPAELRWTDQNKDGVYAKALHKLQMKKNVRPAN

>LL_018634_T.1

MVKPKTPESLYVAKFPTGLDEKLKDFEDRVLWQQQQRGRPQLLGIWGLGGVGKTTLAKEF

FNTKKSDYHRSCFLFDVKANAQKGSLTSLQRELLKSLSGLDKPIKNVDEGTGMLQEHIKK

LISSNASFHSLTGLDEPVHGVDEGKEMHINRLKSSDTLASNNNILVILDDVDHVDQVSAL

LPVDLPSGSLIVITSRDKHILIASKVENSSIYNLTGLKEQHSLELFCSYAFSQTYPRPGF

ESIVDSFLDACNGLPLSLKVFGANLYGRDISYWKDELGSLPTEIKERYKVSYDALDKEEK

QMFLDIACFLIGENRDAAICIWEASGWRARRGFQNLQDRCLVEVDSSNLISMHDHLRDLG

RDVAEESSP

>LL_018937_T.1

MAMAASSSTSASTSTSANLNSNSNSSYDIFISHRGPDVKNTFASYLYRRLHSHRLKVFLD

REELQQGDYIHSQIEGAIQTASVHVAIFSPRYAESTWCLNEVLSMLKSGAPIIPVFYNVK

PAELRWTQGKYAQALQEKEKRHDPATVEQWKKALSNVAGISGFELEACNGDEGELLEKVV

ESVLRKIKKTILHVANYPTGLDEKVRDFENQVLSRHQQSGSRVLGIVGLGGAGKTTLAKQ

LFNRKLSYYKDKISCFLSDVRETVDKGSVTSLQSKLLTALTRSTVQIDHKDEGMEKLKMH

LSLSEILLILDDVDKVDQVDALLPRETSVHSNSFILITSRNKDVLRMSGVGESSIYKLDG

LSEQHSLELFCCYAFSRRDPLAGFEVLTQKFLKACQGLPLSLKVLGALLFGKNDMSYWHD

QLAKLENILPDEIQERLKISYDTLDREEKQIFLDIACSLIGHDMNKAIRIWDASGWKGRL

GFQNLQNKCLVEVEDFSYRRAKNINMHDHIRDLGRDIAGSVLPCRLWRWTENVIDDLLQQ

NIVLTVRG

>LL_018974_T.1

MQAGDRVISQIEGAIATASVHIAIFSPRYAESIWCLDELLLMLKSGATIIPIFFNVKPSD

LRWADKDGVYHQALRKHEEKRRYDSQTLENWSRALADVSSLSGFELDGCKGDEGELVDKI

VQRVLKKGEKGLLNVAKYPTGLNAKLEDLESTVLLQQRQQRGNAQVVGIVGLGGVGKTTL

AKELFNRKSSDYGGSCFLFDVREAAANCSLNSLQSKLLKDLIRLDRQIDSIAEGIEMLRT

NLKSFHALVILDDVDHVDQLDAFLSVKDTLRSDSLILVTSRDKHVLTSSGIGESSIYKLT

GLNRQHSRELFCSSAFCHPYPLSGFEYLVDEFLTACDGLPLSLKVFGSLLCGENDKSCWK

DQLDRLRQVNLPTEIQKTLKISYDALNAEEKQIFLDIACFFIGEDRDMALRIWNGSGWKG

SLGLRNLKNKGLVEVDTKNKLRMHDHLRDLGRDIVEQEKSSGLSHRLWRPTKNITDLFQG

SSNVQAITNVRGVRMVPRVDPFDVYSDLHLHSEMSGDKSWNYKNFGNRVKRLHSRHHNIL

GVRMLQLLITEDSYLESILRRVQSPNLIWLCWYKCPHSSLSSWIPMENLRVLEVAGSKLK

MLWRHESQAPLKLQELNICAPLLKFPKSIGQLKLLEKIVVDYPPMWIDLKTLPEESFQLK

TLPEEFCDLQSLKHLELKRWSTMKSLPDSFGNLTNLEYIDLSESYNLEMLPNSFGNLIQL

KHLCLQSCYKLTMLRETLGNINTLEYLSFSDCPNMEVLPPQVTRQSLEELYLLGTSFKEL

PSAIGELGNLEILELGSPFLEMLPPSFGYLRSLQRLRLYNCQELKYLPDSVGLLTKLTTL

NIECCGLCSLPSDVMKMNNLESLQVVDCPLHEFPFSRALTKLGSIDKCMFQLKFLRLYNT

RLSEVSFHEGVCPNLQYLKITSCYELVEVGALPASLITLDLSSCPALRRIRGLCGLAKLQ

ILNMNGCKEVEELPSLETLISLEDFRSDIYYKLIKACTVRVVDKAEV

>LL_019043_T.1

MDLIQTALGALTELGGVIQFIYSSKASGKELKEKLLLLKPNIDEAIQIISSSDSTTHKQK

QFKEFQAVLEDGLKLVQEGDNLHIFDIYRKWRYGRRIREFQEKIKDLIDIQGSPILAHDL

QKLGADVTDLRRRFESAAIAEQPISYTSHEVPDMPNFVVGLENRINTVKQIILQNDVDRL

GITGMGGSGKTTLASALCNNPQVKDSFTNIIFIPVKQSNPNVKGLLETMWDKIIGVQRPD

FQSIEDAHNKLQKQLRLKSYRPILVILDDVWSTSNLENLLFEAEGYKTIITTRQDSTIPK

TKSTRFYNMPMLEDANALSLFCFWTFGQPSIPTTEKEDLVKQVAAECKGLPLALKVIGSS

LRGKPRPIWKNAERKLSKAESISEYHREALLKRLETSIDFLDDIHKQCFLDLGAFPKGRK

FSVESLLDIWVYVRGMEWTDAFEVLLELATRNLLNLTGDLGSAAIDYNCASDLSFSQHDV

MRDLALHLASLDNINSPKRLFMPRKEDKIPTDWRSILKDQSSRAQFVSIHTGAMQEQDWF

QIDFPEVEALALFFDANHYCLPTFLHNTPKLKVLILCNYSSTRAILSGMPIFPSPVQIRS

VLLNKLIVPSALYERCRSWERLEKLSVCLCEGLGNITLMDMELQPLNFPNIMEINFDHCS

DLGELPNKLCNLSSLQRLSVTNCHLIQNLPDDIGRLKSLRVLRLSACPSLTRLPASICKL

GQLEYFDISLCRRLEDLPTEFDQLSNLETLDMRECSGLKKLPAIKQRSLKRVIISDSDKE

ESEGAWLYIKASAIPNLTIDVVQEKFSLDWLDD

>LL_019124_T.1

MELRVGDKFPSQIEDAIRSASVHVLIFSQRYAESEWCLKELVYILASGRPVIPVFYHVEP

AVLRWENAKGVYSEALRKLEEKRTHDPETHEEKPRYDPAAIQKWREALSSASLTTGIDLQ

GTCNGDEGELVKKIAKRVLEIVKNKPVGISEFYCSQQYGCPGGLIDWSDGTY

>LL_019130_T.1

MLRGLNPQDSQELFCRHAFDQPCPFIGFEQVVEKFLNACDGLPLSLKVFGELIRGKDLEY

SKAQVDKISRILPDNLLKRLKVSYDSLDKEEKQIFLDIACFFIGEDRDTATRIWDASSWD

GWLGLRNLENKCLVEVDSENCITMHDHLRDMGRDIAKNEPPEWTLRLWLPSNNLLHLSYQ

SHVRGIIMDPNGREWAEQSFKQLAEFAGSGFVSNMSGLQLLRATGYCIESVFSVVQPQQL

IWLCWYTCPYSSLPSWIPIKNLRVLQLKGTNLETLWSNESQAPLQLRELEVYGGLLNIPK

SIGKLEHLEKFALHRSMPWPVDDLKLLPDEFCLLLSLKHLELKCCRGMKSLPDSFGNLRN

LQHIDLSYCLNLQMLPDSIGNLVNLQYINLFSCTNLETLPDSFGNLTNLQHINLSYCSHF

QILPCSFGNLRNLQHINLVSCTNLEMLPDSFGNLTNLQHINLSYCSHFHFQTLPYAFGNL

RNLQYISLSRCSHLQMLPDSFGNLTNLQHIDLSDCSHLKMLPESFGNLTNLQHISLSRCS

SLNTLPDSFGNLRDLQRIDFSSCSRLQMLPNSFGNLGNLEHINLSNCSNLQMLPPTFGSL

SNLKILHLKSKLLKMLPPSFGYLRRLEELELYVCHGLKGLHDSIRMLPQLRKLKIREMNN

ELLEKEDCSDLVEVGTLPTTLIKLELIFCVGLRKIGGLCGLVELQILNISRCIDLEELPT

IETLVSLEELCASMCLKLKSIQGLGQLTKLRRLDVKECSELKELSGVEHLKSLEILDSSQ

CPKLQWGGGVVEQLRQGLKEGFIT

>LL_019176_T.1

MAGASVVGGLTQSLTSMLADQLLQEASLITSFKEDFEFLCDELVSIKCLLNDAGEKRNSA

SICSWLDELEYFVYDTVDVVKECSRARKFGNPIFRYRIGRRIRGLKERISNIHKSAKYLK

YLMSVMQLHAFNADNLEDQRERSSALIKESQIVGMEHDICYITDLILQKDGPQVISVMGM

GGLGKTLLAQHVFKSERVQQGFDYLIWLAFSHSFVVKQVLVEMCRQIKLSVHGQQTDEII

TKIHQHLKERRCLLVLDDVWHRDVLEKIGFSLETMSKIKILLTTRDRKVAEAMPHPHHIQ

PINYLSLEHSMTLFCIHAFPERCEKSPPNELSSFAYRIVEKCSGLPLAIKTIAASMAKVR

RLPNDWERTLNRLMEVHAMSNSVMASLRLSYQALPNHLKLCFVYFSVFPKNTQIKFDYLL

HAWISEGFTPEEEAYDVARSYIDELIDRCLLEVSKVSGDGRVKYCKMHDLLHDLALSESQ

KQTKCLLKPGKEIKEFPVNECVGLRRISLIKNDISQMNKVIQCPGLRTLLLWNNTHLTSI

SASFLTNVKYLAVLDLSQTSIESLPESVQNLKHLRFLNLSQTKIKKLPESLTSVRSLQFL

DVSWCKNLCELHSGIGEHKSMEYLNVKGCRNLKSLPVGISKLICLQSLKGAELKRGKATN

AKALQLKDMKGMTLLRQLTLIIDASSSEGTVQLEEGTFGGMTKMRDLSFKYINSCSLLHL

PKDILVMQRLEILHLGGCVVPKWIFQLQNLMELKLLGDNDSADYKGLERIPNLRKLQLSG

KEKCVEFPLEFGERGAFPKLVKFILEDFTSLENFPSLQDEAMPMLRHLEMKNCRELKDMS

EALERLSSNLKNIEVKCCPRWEDSLWKGERTWHLLKDHTIKLTVNGHKICWEMNELENLR

EIEALKAKSVQEMVFL

>LL_019475_T.1

MADPGFLPGCIGVAIDIAIGQILNHDVIAVSCKRELESLRDLVTSIKRTVTQIHLCRDIN

ASEINTWIIKLDALLQGALTVVQKCSIPRFALVSRHKTSRRITALISEIEKHLKWVSLVQ

LQQQMQMQMEMRRESAQPSSSTSATTSEGLSPSTGGFSIQEPLIVGQEEAFTTLQMWLLD

ATVISIFRIGIVGKGGSGKTLLLKRLFSNQEVRDFFSDGFLLWLTVSQSPSLTSLRSELC

RQVAMQTNVVFSQNMMEEDAKNWLNQRMQGKRFSLFLDDVWGEGAILLEELGVALLTHHS

DSKIIVSSRDRRALLEMGVSETSTMAVGDLVEEKSWDLFAHHAFPYSKGNLPANIDEMTA

KLVCAKCGGLPLAIKVVGRSMAGITDAQEWELAVRRLPNEKKQDHQALYDRLKWSYDSLG

NYHVNLQLCFLYLAAAFIEDQIVDVEVELISLWVGEGLLAHNKLGDQAGRKED

>LL_019579_T.1

MALDLATGTAGNTISNTITSMIINPLIQQINDAIHLDENRNILESQLNRMKSLLLDISNQ

FQDQQRTLPETVSNCLGRMETEVVKARDLIDRSLQPQHCLDFLIFKPNLPRQIREWKADF

EGLFRELQTDISVFYTAREIVSSAPHQAELLLQDAPDYGFVGLGIKSAEIQLKEWLTETP

QVGLIGVWGMGGVGKTTLLKKVYNYYKVSNIFDVVIWVTVSQFSIIELQSCIAQTINLDL

TVFNSNIDMRKMKLSAQLKIKKFLLILDDMWIPLNLKQLGVEFGNDRGSKVVFSTRNGDL

VRQMNTGKSMQLQPLSPEEAWELFRNVAFKDSPLSPGLEEIAKDVANECKGLPLAINVIA

STMIGIKGVDQWNLALSQMQTMDSNFPSTHPRVEYELYQILRWSYDRLPNANFKNCFLYC

AMFPEDKEIEVDKLVGMWIAEGIVESKNPAILMETAKSYINLLVDRCLFEVAHFPQGIVF

LKAHDVLRDLAIYIGEKEENCFFRAGQMHEHFPLEIREDCKRISLYENNITQLPPQGLKC

TNLVSLILRGNKGLEEIPEAFLVNFPCLKVLDLSRTKLKTLPKSLWQLTQLEFLDLSYTE

IEDVPEGIKNLSRLQFLDLSGCSKMRSLPRHIRELKNLKSLQASQYNVVL

>LL_019832_T.1

MIISVPQDVTDMAEQAERLNNVIHGINEKLAQEQKPPQRIVKAWLDKAGEAIESSGSIRS

DYWEHKNCLSCCPNCLCRYKISKRIRDWKGNVAQLHSQRQSDFPPTGEYGDPTPRTQVPI

HENPESSSAANTRRSAQTKLVERWLTDEDSNVRRIGLNGMGGVGKTSLLDTINNSQKVKN

SFELIIKVTVSKNHILDMQDCVASRLNLTQFPDKSNFEERKDMLCRSLVNKKFLLLLDDM

WAEDM

>LL_019876_T.1

MDPDAASFVSSSHGNTNSSVFDVFLNHRGPDVKKGLASHIYRRLIVHGFTVFLDQEELQR

GENITPQIEGAIRTASVQVAIFSPRYAESPWCLNELLLMLESGKPIVPVFYNVNPSELRW

TRSGNGLYAQALRRLENKATVDQRRQKKPRHDSSTVEKWSNALSDVSKISGFELNACNSD

EGQLVDEVVEQVLKMVSKTPLNVAKYPTGLDDKVKDVERMVLLQQQSQKARVVGIAGLGG

VGKTTLAKEFFNRHRSRYVQSCFLFDVRENAAQGSLKSLQSTLLKELVQKNEQINSTDEG

IGKLKKHLSEKREAVIVVLDDIDHIDQLDALLPPLKGTIHSTSLILVTSRNKDVLTSSGI

VESLIYKQTGLNPQHSRELFCSHAFDQTCPLTGFEP

>LL_019966_T.1

MADPCSSSSMAGSASTSYSQNFQVFINHRGPDTKDTFATPLYHSLRQSGLLVFLDKPEMQ

LGHDIPSQIRAAIQDASLHVAILSPHYAESEWCLNELVLMIQSGKTIIPVFYSVKPSDLR

WTGEEKNGPYARALYAHEKKKRYDPQTIQQWRTA

>LL_020006_T.1

MAASSSTAAPISSSTPAHSSHNQPLPWCNVFINHRGVDVNKTFAGSLYLRLIQKGLRAFL

DKEEMQPGYNFSCQIAQPIGTASVHVAIFSPG

>LL_020045_T.1

MVEKIVERVMKMSEKPGLYVAKHPTGLNEKVTDFETTILKQQQLSGNVQVVGIVGLGGAG

KTTLSLELFNRKRSNYNKSYFLSDIREKANKNSLHSLQSELLNGLTQLDLHINNINEGIG

MLKRHLTSSHVFFILDDIDHEDQLEAFLPVKDVLSSNSLVLITSRDRDVLARSGVETLSI

YNLRGLPRQHSLELFCSYAFHQPYPVKGFEDLVNRFLDVCDGLPLSLKVLGALLYGNNDK

SLWGNILYKAKVPTDIQKRLQISYDALSKEEQDIFLDIACFLIGEKKNSAITLWDGSGWN

GSLGFQSLQGKCLVEVDAKNVIHMHDHLRDMGRDIAEEKLPRRLWRPTENIDNNWLHQQS

SVITEARGISIRHTDSSDESVISICCRFLDIRNRVMELLYTCYRFFGVGAMKLRLLSTEG

YLLERFFRRVHSPNLIWLSWSDCPYSSLPSWIPMRNLRVLRVQGGALKTLWQDKCEVSNS

FGNFTNLYHLDLSGCNKLERLPDSFGSLTNLRHVDLSNCKNLFRLPDSLGGLTNLHHMDL

RRCRKLERLPDSLGGLTNLHHMDLRRCRKLERLPDSLGGLTNLHHMDLSECEKLERLPDS

LGGLTNLHHMDLSFCGN

>LL_020356_T.1

MSFTRYNPLERTVSYSPRKYDVFLSFRGEDVRKTFVDHLYASLENAGVNAFLDSEKLEKG

DEISPTIEIAIENSAIRIPIFSQNYATSHWCLEEVSHMCKSGGHIIPLFYDVTPTEVRYP

DRGAYAEAFQQKKRRYSKAKINEWKKALAKVVSFSGWSRDDTFGFEAGLVKLVVQDVFKT

LQNVIPEEEDYPDKPQDVAEEIPLEAMPLEIRNEPLRISKSIFGMEERMNEVIHLLKTDS

EDSLITIAICGMVGVGKTSLAKAVHNHIYHKFDAACFVYDVHHKAQHTNGIAKMQRQILK

DLIKFKDKVNSEAHGKSLMRDRLRSIKALVILEYVGDSKELEALRGDWYGSGSRVLVITI

DPQLLNERQVDFVFEMQGLDSDHALQPFSWHAFLRDRPDEGYEELSQRAVNICNGLPFLL

EVLGAHLYNKDLSYWVEACQILEDLAHQDKYAILRLCFDGLNSEQKEMFLDMACLFIGRK

RENVLSFWEASDFDPNKGLMDMVLKSLISLVEDDKFAMHSELRDMGRAIVTEESEELGQR

SRLWKTHEAELVVMEGKG

>LL_020501_T.1

MLESGKTIVPVFYNINPSELRWTRGGDGLYARALSILHCILFCTRDENGGYARALRMLEK

KTTVDPRTNKKKPRHDSSTVEKWSNALSDVSKISGFELNACNSDEGQLVDEVVEQVLKMV

SKTPLNVAKYPTGLDDKVKDVERMVLLQQQSQKARVVGIAGLGGVGKTTLAKEFFNRHRS

KYVRSCFLFDVRENAAQGSLKSLQSTLLKELVQVNEQINSTDEGIGKLKKHLSCLRGAVI

VVLDDIDHIDQLDALLSPLKDTIHSTSLILVTSRNKDVLTSSGIDESLIYEQTGLNPQHS

RELFCSHAFDQTCPLTGFGRLVEKFLDACAGLPLSLKVIGALIRGKDFEYWKA

>LL_020586_T.1

MESLPDSLGNLTNLQTIDLSGCNGLQMLPDSLGNCTSVQTIDLSGCSGLQMFPHSLGNCT

SLQPIVIAHTFASTSASASASASAHMYDVFLNHRGTDVKKNLASHMYYRLPFYGLKVFLD

QRELQKGECLTRQIEGAIASASVHVAIFSPQYAQSRWCLDQLVLMLESSKKSGATIIPVF

YNVHPSDLRWPDKGRYARSLLAHESKGRYNKETLADWRAALAEVSHISGFELRSCNGDEG

ELVEKVVQQVMKKVKKPPLHVAVHPTGLDNKLNDFERKVLLQQQHSGQARSGNVEN

>LL_020590_T.1

MEYINPVIELMRDVINEEEKREVLQTDFQPMLNLLRDLTTLFPTVFPDQRKEAQEALKIW

MQLLHNCLREAISLLHRPQPGRPRRCLDRLICNPRTLSQEINEWKVLFHCLYQELQSDLS

IFVTACQSLLPLSEDEDEDEAASEDEDEAASEDEDEDEDEAASEIPLSEAEVSEQPLCEG

EAWMLFQRIAFKDGPDPTDNIQLCARNIVDACKGSRSAISVVAAAMIGKSSLLDWQNSLD

SMKIADPSFRPENHPQLYQLLRWSYDALPTYLKNCFLSSAIYPKDANINVTELVQMWIAE

GLVRSKQETHLMDMAIARTYVNLLLDRRFFHSATDEEIQDDEEIQDDEKTQYIRVDGAIR

EMAMYIAEKDHNYLLKAGQSLQHFPTVHSEDCKRISLRWNNIEDLPSEFRCPKLVSLILS

Y

>LL_020638_T.1

MALEVGTGAAAGVAAEVVNKHIVAPLMQRIDDLIHLDRNTRSLQVNLERLKRLLQDISTA

FENDKKRPADSIAYCLQRMDQTFGEATRLTATQGKQSFLGSLKFPRQVTEMNTKFDQLFQ

DLRTDLQIFLAAQQIVSASPPQGTNVLLQPVPEGGFVGSVIESAKNQLQTWITGKAPQDR

VIGVYGMAGVGKTSLLKSVYNFYKVSHLFDVVMWVTVSQDYKISGLQRQIAEAINLDLSS

TSNTDTQTMKLSASLKNKKFLLFLDDIWNALDLQELGVAFGTDKGSKVVFSTRNRDLASA

EADKSTEVKPLSMEQGWKLFQRVAFKGGPVPEDLKDCARQIADECEGLPLAITVVAAAMR

GKKKEVEWNTSLSLMKIADPSFPDTHPRVDKELYQKLRWSYDDLPTHLQNCFLYCALFPE

DHQIHVEQLVLMWIAEGVAKSKETTYSMDMELGLSYVKILIDRGLFQNFDFEKQSNDIIV

NRPVNVWKWRGITVHDVIRDMAIYIGEKEDNCKRMSLHSNQIEHLPNDLVCTQLVSLILS

GNIPESFLMNLTSLRVLDLSFTSIQSVPTSLGQLRLLEYLSLRRTQIKELPEEICNLSKQ

SIIIAMYDRKAYTTAVPSVVGLS

>LL_020739_T.1

MANQASSSSSPSASVSTSYPRYDVFINHRGPDVKNTFAAHLYRRLLDHGLRVFLDKPELL

QGHRITPQIEAAIQVASVHVAIFSPSYAESKWCLEELVLMLKSKATILPVFYKVEPSQLR

WTVENGEYAKALRKLEEKTTVDSKTGEKKPRHNSETIQNWRSALSQAADISGFNLDCDEE

ELLNKVLQGVLKSVSKLPFYVAKYPTGVEDKLQDFEREVLQRHEQQVEAKVVGIVGFGGV

GKTTLAKAFFNRNRSDYKESCFLFDVREQSINYLQTKLIEDLKHTTIKIQSPDEGIELLK

KQLSSCEALIILDNVDKADQLEKLLPMKDILSSQSLVLVTTRDKHLLKQWGVLESSIYNM

TGLNMPHSKELFCSYAFFKRFPPPEFAELVDKFVTTCGGLPLSLKVIGAFLCGENDEHYW

KEQFDRLQQSLPEEIQKTLRISYDSLVKEDQEVFLDIACFFLGDRDRAIRIWGLVGIRNL

ENKCLLEVDS

>LL_020909_T.1

MWVKLFQRKKPEFQNVEDAHRQIQEQLLRQAKPILVVLDDVWSRANLEKLLFERKGYKTL

VTTRVRSTIPTAASTQLYELPLLDDADALSLFCFWAFGQRSIPNSADEQIVKQVQAECKG

LPLALKVIGSSLHGEPRPAWERAKNKLSNGEPISDYHREGLLRCLETSIDSLDEEARECF

LDLASFPEDRKISVDALLDIWVYVRKMEWQDAFVTLLELASRNLLNLTSNLRSQAINYGS

ASELYFSQHDVMRDLALYLASRDSIVRRKRMLMPRKEDSLPGKWELFKDQAFDAQIVSFH

TGAMEEDQWCEMNFRQAEALVLHFSASKYSLPSFLSSMKKLKVLMVFNYGSKRATIDGLP

ALSSLTQLKTVRLERLHVPSLQDHSLILQNLKKLSLSLCEGLGNMSSFNNTKSSLRLPTM

LDFNLDHCCDLEELPPGICDTSSVENWSITNCHLLRNLPDDLGKLSSLRMLRLSACLGLK

ELPASIGKLGKLEYLDLSLCECLKELPEEVGQLRKLEELDMRECSRLRKLPKSVGGMKSL

KRVICDEKIGQQWNRVKSLAIMELR

>LL_020933_T.1

MRFLACKFYRYNPCITNHFAAPSKFLFLSINTSIFRIISIPMANTCSSSSAASCSASTSY

SGYQVFINHYGEDVKKGFASHLYRCLRDRGGFRVFLDKPELEEGDEISSQIKDAIREASV

HIAIFSENYAKSRWCLDELVLMKEARATILPVFFNVEPSMVRHAVKEGKYAEHLRVLQKE

TTHDPITGEEKLRYEFSTIQSWRDALYDVANRKGFELKAFNSDEGELVDKLLVKVLEKVP

KTELDVAKYPTGLGKKFEEFEEMLSQHHDEPEKTNVVGIVGFRGVGKTTLAKEIYNRKKS

CYSKVSFLSDVREKAATRSLTSVQSQLIKDLQDIPIEIENTAQGIGILKQHLSFCHALII

VDDVDKQDQLDVILPIKDILKPGSLIVVTSRHKDVLRSSGVSSIYNLEGLNTLHSKQLFC

LYAFSQPFVPRKFAEFEDVVDQFVNACDGLPLLLKVFGALVCGHNDKGYWEGRLDRLRTT

LHDDFSDEITWAF

>LL_020944_T.1

MLKDHLSSHDILLILDDVDRVDQVDALVPNQTSIHPNSLILITSRDKDVLTRSGVENSSI

YKLNGLNEQHSLELFCRHAFRRSDPLTEFENLTEKFVNAGKGLPLSLKVFGALLCGNTDI

SYWQQQLDKLVKILPTEIQERLRISYEALDSQEKQMFLDIACFFLGEDRDMAITIWNGSG

WAGELGFQTLQNKCLVEVVEQNRGWNREIRMHDHLRDLGRAEARVSQPLRFWRIDDLLQQ

SSVITVRGIRMVLSEYLDDEEFDDIQMRRLQLVDTEGILMDMALLEDGEIPRSDMALLER

VSILFPA

>LL_020948_T.1

MAMAFVTGTAPNIAASAISTHIIDPLIQQIDDVIHVDENRRLLRDQLNHMKGLLRDISYQ

FYDRQKATPESLRNCLERMEGEVRKARELIDRSQSPQQSCMGIHCVCLFELSLSRQIRKW

TTSFNGLFDELRNDFQLFSSAQLIAFFSPQQAEMKIERLLTESPHVRRVCVYGSSGVGKT

TLLRKIYNAHKVSDAFDAVIWVTVAQFPILELQGAIASELNLELPETDVVERMMKLSAYL

KAKNFFLVLDDMWTEIDLEKLGVEFNNHRVSKVIFSTRNRDLIEEMKAEESMKIEPMSTD

EEWDLFSKVAFKDAGESDVPEEIVRIAREVSSECKGLPLAINVIASSMIGKSDVNEWKHA

LRQMQKSRDFIDPIVHHPHIDRDLFQRLRWSYDCLPDSNLKNCFLYCAMFPQATQFPVKP

LVQMWIAESLVNTKDDGHEFVQFLQDRYLFQLQSTENVITVHDAVRDMAIYIGETSKEKC

VFRARQMLQQFPLSRGINDCKRISVVGSNIKSLPAWELGCQQLVTLFLGKLELKEIPEAF

LGGLTSLRVLDLGGTQVKSLPLSLWELRELKFLNLSWTQIENLDERIGNLSSLQFLNLSH

CMKLKSLPSQIVKLKNLRYFDFSECDNLRLIPKEITILPNCEVIVQ

>LL_021116_T.1

MDMITQSNVFIAPVMQLMKLVIDDEESRDILQTDWSPINNLLLDIASCCKLIGDQRKEVL

EALKVWMQLLHNSFTEARSLLHRSKQQRQRRHRQRCLDCLICNPRPLSQEIKEWKVLLTY

LFQSLQTDFEKFVSARHIVKSSPILEDVAKSAQSKVETWLTEADHVRTIAVYGMPGVGKT

SLLKRVYNNYIKVTREVFQHVIWVTVPNNYTILDLQRQITEAIGRYYRSSAHSGTIKHWL

SKTLKGKKFLLLLDELRSPLDLQEVIGVAFSEEKQSKVLFTTRNRDLIPLMSAEESIEVQ

PLSTDEAWMLFRRIAFKDSRVSMDIEGYARNMVDECKGLPLAISLVAAAMTGKTRVDEWK

TCLSSINA

>LL_021188_T.1

MAGDIATGCAGNVLSDVFCNVWNKVVEKGKMVISLPRAFTQMKEEVGTLDNEIHDINAEL

NRKQWPAKRRVQTWLDKAGELKLTSETIVTEYETYQQHKSCLGCCPNCVRRYRISNDIRD

WTRKFSELYSEKAQPDFPRIGEYGDPTPRKQILIQDSDSGFVADAKRSTHTELLQSWLTD

EDSNIQRIGIIGMGGVGKTSLLNIINDCQKVKTSFELIIKVIVSRNNISDLQRQVAERLN

LELPGYSEARKAVLRNFLKNKKILLLLDDMWADDMWTSSKLQEIGASLNDTGSKIVLTTR

DKEVCERMNVQETIEVEPLVQEEGWQLFRSRAFENRNGNVPLELEEVVREIANECKGLPL

AITVVAAAMKAHSTVLRQWENALYQMRNVPDTFDGLYSIVDKELFRRLQWSYDFLNSDNL

KACFISFAAFPEDRVIDCEEVIDIWVAEGLVKGTGDSYLPDTADSFIIHLRNRCLIEVVE

TDGVGKISHIKIHDILRDLAIRIGEKKHGCYFKAGQGVSHFPLEEVDSERRAKLSLMSNN

IQSLPQTFTSSTLSVLLLSQNPHIKEVPESFLNELPSLRVLDLSETGITCLPPCIENLKH

LVSLQLKKTGICELPETIGDLKELQFLNVRECKKLRCLPER

>LL_021194_T.1

MASLFHEWLLLPLRRSLNFLQSIVQINPIAVSATTTPIAEGLSSSSNPIAVSATTTSDYY

DAFLNHRGDVKEILATPLHQRLTDCGLRVFLDKKELQEGDNIPSLLKAAIRSAAVHIAIF

STNYAESPWCLNELLLMFESGKPIIPIFFGVKPSELRDARGQTGRHLDLLEKKKAIKSES

QLQNRRYDPETVKEWRDALHKASNITGFVVEAEACNSYDMHLLDKIVKRVLEKLPKTLLH

VSKYPTGLDDKIEDFERTVLMPPQSGKTVGIVGLGGVGKTTLAKEFFNRERSKYDRSCFL

PDVREKAASGSLNSLQSILLEQLTGSKVQINSIDEGIGELKKRLSSSRPRALIILDNVDH

IDQLNALLFPLNKASLLGCVILVTSRNKDVLTSFGIVETSIYKLEGLHRRDSRELFCSHA

FNQPYPVKGFEQLVECFLDTCHGLPLSLEVIGALFLGNKDLKDWEAQLRKIKKILPEDIQ

GKLRISYDSLDEEEKQIFLDIACFFIGEDCDTAIRIWDKSGWEGRLGLRKLKNRCLVEFD

SENCITMHDHLRDLGRYLGEKEPPGRELRLWCPSDDSLQNLSSQSPVRAISLVHVNGSQQ

SFENLAGCCNLSRLKLLRVQGDFAGLPDSFGLLTSLQSLDLSQCSTLQAIPDSVGSLTSL

QSLDLSECSTLQAIPDSVGSLTSLQSLDL

>LL_021203_T.1

MAHLQKLISSSAASPSTAATPNSNHNYDVFVNGHRQEVKKTLASHLHHLLLLDGFRVFDQ

QEILEEGVSLNSQIRGAIATASVHVAIFSPRFAESKWCLEELVLMLESGKSIIPVFYHVK

PSDLRIRAGNGEDGVYGRALLDLEKNSRNEPNSHRVWREALLRVADISGLELEACNGDEG

ELVDRVVQLVEEKVIETQLLAAYPIGLDEKLEAFKNMVLTQQQQQGVQTTIVGIVGSGGV

GKTTLAIEFFERNRSDYNWSCFLYDVKGAAGRNSLHSLQNQLVKDLTCMDFEIINVSDGK

EKLSKIFFPYQALVILDDIDDVEQFFAFFPRGNDHLVSGSLILVISRYRHVLEKSGIDDL

SIYDLTGLNETHSQELFCLHAFHKRLPVLGFNEVVTQFLEACNGLPLFVKVFGALVYQKD

LKYCETQLRKIAKILPATNIQGLKICYDSLDEQEKDAFLDIACFFINEDKDMAITIWDAS

GWEGRLNLENLQNYCLVDINSKNCIRMHDLLRDLGRDLAEKERFNCRLWRPTDNLAFHGA

FQQSTVRGVNMVQGSISNHSFVDMSRLQLLRAEGDCLGSISKHLTHSDILWLCWNHCPYT

SLPSWIPVWNLRVLQVAGGELKMLWMDESEAPMELRELYISADVLRFPKSIGQLGNLEKI

ETKAAIIEPLPEEFCRLRSLKHLSLRRSLRCRPFYGDYSDLKMLPELFGNLRNLSHLDLF

GCSSLEMLPDSFGNLGNLSHLD

>LL_021270_T.1

MADASGASSSSAPSAYPYYDVFINHRGPDVKNKFASRLYRSLNSFGLRVFLDKPEMQPGH

SIPRQIEAAIGVATVQVAIFSRTYAQSKWCLDELVLMLSSGKTILPVFYKVKPSDLRYGA

YAEELCDLEQKKTHDPETHQQKKPRYASDIIKAWRDALSRAADISGFELNDRYGDEERAE

EELLDKVVQTVLKKIPKRLDVAEYPTGLGEKLQDFEFKVLSQQEQQPVEAK

>LL_021345_T.1

MDPSPASSSTASHSIGSGHDVFINHRGPDTKNTFASCLYYGLLSRGLRPFLDREELEEGV

EFPSQIEDVIKNASVHVAIFSPAYADSRWCLHELVLMQETRATILPVFYNVEPAELRGID

HNKTGRYAEALNKLQEKKTVDSQTNQEKPRYDPAIIQKWRNALSKVADTSGFDLKGKFNG

DEGLMLHKVIERVLKIMATKTRLHVAKFPTGLDDKVKDFETVLRQQPTGKAQVVGIVGCG

GVGKTTLAKEFFNRKHSDYSRSYFLSDVRENAKISLHSLQSELLKGLGQLDGHVNNIHQG

TQMLLEFLSSAKVLLVLDDIDHQNQIDALLPNRDALLPNRDALLPNRDALHKDSLIVITT

RDKDVLAGSRVAEPSIYKLTGLTAQHSRELFCSYAFCYPHPLPGFVDVVNEFVDASDGLP

LSLKVFG

>LL_021371_T.1

MDSAEKSESQAPSNSSAVALGGVQTCSPSKTKPRLKPLTVKFSDFHPKNSSVSSPKVSSP

LPPNLSDSSLYNSPTNFSPASSAFVSASQSPFLSPRLLQLPNDWASQSPLLSANAAGLLL

QLPHGSASESPFLSPNSSGFLQLRNDVEQQQQKQQQQQQQELEYSPFSQSGSHSDDIPSS

SYTPPAENDSIHLANCVQSHSIHLTNYVSEDFSDDIQRSKAQAASDHAPPRVSFSFPAPR

VSFAKGPMSPPSSSKLRSCDVYIGIHGQNSKLLRFSKWLHAELELQGIACFAADRARYSD

SQSHDVADRIISSATFGVVIVTRSTFFNPFGIEEIRVFMQRKNLVPIFFDLGQSDCSAND

IIEKKGELWEKQGGELWRLCGVQEKEWREAIEGLGRIQEWKLEANKVNWRNCILRAVSLL

GSRLGRKSVAERERVKKERVDGQEFPFLRNIGFVGREKELTEIENILFGRDADDEEDFQV

CCFGGYEKDRGQESAESFMPRRRSDVDMWTNMKGLGFTEGYAERRKSDADRFAWLEGKKK

EADALKNKSRGHSETKRCKEPTLEAWIEPPLELVNRGRSPQKLRMKAKSAHRSRHGHKHK

DDNSRAEPRNLSYGSGIACIHGVSGIGKTELALEFAYRNSQRYRMVLWVGGETRYFRQNF

LNLSLLLGLDVSTETQMCPEKGRIKTFEEQEHEALQRIRRELLRDVPYLLVIDNLESEMD

WWDGRDILELLPRINAATHVIITTRLPNVMSFKPLELSYLSVVE

>LL_021401_T.1

MGGAGKTTLAKELFNRKSSNYGKSYFLNDVRESKRSLHSLQSKLLKGLTQMDLSVDSVDA

GIEILKDHLSSFHSKLIILDDVDSVDQVDALFPDQTVLCSGSLVLITSRNKDVLTSARVQ

ESSIYHLTGLNPEHSRQLFCLHAFSQPHPQPEFEYMVDMFLKACDGLPLSLKVFGALFYG

QTDIEYWEQQLDRLQHLPSEIQEKLKISYEALNKEEKKIFIDIACFFIGENKDMAIRVWD

ASAWKGLMGFRNLKNRCLVDVDRENKIQMHDHLRDLGRQIAEEESTSQLCLWNPTQNIDD

LLQQTSAEVIAEVRGMRLRPSEYNDDDEDASFSCCDCGKLQRLNAGKLQIVDIEDDLLER

ILRRVHFPNLVWLRWKECPCSSLPSWIPTKNLTVLEVDGNNLETLWETQVPLQLRELMIT

APLSKFPKSIGKLKHLQRIHVYLTDEDNVKTFPEEICNLRSLTTLELLRCSKMKSLPESF

GNLKNLQHISLSGSRSLERLPNSFRNLIRLKYLDLSDCINLTISMGTFRNITTLECINLR

SCEKMEVLPPQIAHQRSLEKLCLNGTNLKELPDAIGKLQDLEVLELGSPLLETLPLSLGE

LRNLQQLALSGCRKLKYLPDSILQLNQLTEMTVTGCVKLCDLPFKRVEGEREALKESEGQ

RTLSNFDSSIEKCMFSLQRLKLYNTGGLSEVSFQEGVCPNLQHLDIRGCHDMVEIGKLPN

TLLTVELTDCFNLREIKGLCGLEKLQSVLFSRCSELELPNIETLVSLEKLVANGCMKLKS

IRGLEQLTKLRKLECSDLEELLGVEHLRSLNELWTYNCVKPKSIRWLEELTQLRILHLNM

CHDLEELPSVEHLRSLEKLEAPMCKKLKTIGGLEHLEKLRQLSVVHCSDLEELPGVENCA

SLEILYANGCPKLQWGLGVLEQLGQQLERLVI

>LL_021417_T.1

MPVHIFLEVKSLIAELNNLRQLYELGSMDESKMNEAIFKHVSKLTNDPQQNAIMLQQMGA

DDMFEGAFVEVPCNYKGLGKSDFEVGLQKNIENLKSTLLRRDVSVVGVQGMGGLGKTTLA

LALSNDGEIK

>LL_021424_T.1

MAMTNLTTVASSSSPASSFTSTYHVFISHRGPDVKNTFASHLYRRLSHCGLRAFLDKEEL

QKGDTIFSQIELGIRGASVQIAILSPTYAESPWCLDELVVMKNSGATILPIFFNVVPSEV

RHTEKNGAYAKALRELEEKTTFDPQTCEKKPRYTSQTIQSWR

>LL_021425_T.1

MAMTNLTTVASSSSPASSSTSTSHTPHHVFISHRGPDVKTTFASHLYRRLSHCGLRAFLD

KEELQKGDTISSQIKLAIRDASVQIAVLSPTYAESPWCLDELVLMKNSGATILPIFFKVD

PSHVRHTEKGVYAEALRKLEEKTEEKTEEEKTEEKTEEKKTEEKTEEKTTFDPQTREKKP

RYTSATIQSWRAALSSVAALSGFVHSNQDDEGELLDKVVERVLKEVPKTPLDVAKYPTGL

DIKLEELKRTVLFDQHQEPERVETKVVGIVGIGGLGKTTLAKEFFNSERLSYKTSSFLSD

VREAAARMSLNSLQSKLIMDLQHRDVTVDRHNGIELFQMHLKLCHALIVLDNVDHISQLD

AFLPLKGILSPGSLILVTSRDKHVLISSGVSQQSIYHLKGLDPPDSQTLFCFHAFHKPLP

PPQFADLVYGFVGACNGLPLSLKVFGALVCGENEISVWEEVLDKLHNYMTEIYDTLKISY

DSLEPEVKKTFLDIACFFLGEDGNKAMRIWGKKEIRDLQDRCLVELDGQNKIQMHDHLRD

MGRLIAQEGSMPLRLCDRTTNSIEELLEQQTNLINGVRGINMLSHCNDFFHYMPNSWCAW

SGAEMRHFSFSKEVIFKCLRLVACKGGYLENILRAVQSPDLIWLRWYGCYYSSLPDWIPM

KNLRVLEVQGSKLNVPWPHESKAVPFQLRELNIYAPLSEIPKSIGKLKYLESIAICNSDP

LCISDPFESIAICNSDPHSQGCQSTLTGLPDEFCNLLSLKYLFFRGFSRMGSLPFRFGEL

TNLQRIDLIGAASLLMLPDSFGNLQQLEHLSLGDASSLWELPDSFGELTNLTELYISGAA

SLQSIPGVGQLTKLIDLDISGCHEIRELPGLEHLTSLKRLDVGRCKKFQSIRGVGQLTQM

WYLDVSECYELQELPGVEDLTLLEILNAGECPKLQWSEGVVEQLRQRLNDGFIYEKENEN

ERHRKRKRCGENV

>LL_021431_T.1

MYCNPGRLSEEINEWKVLFDYLSQGLQRDFSLFVTAPIIHRDQIEESEQPLSEDEAWMLF

RRIQFCATNIVEACKGSRSAISVVAAAMIGKTSLLDWQTSLDSMKIADPSFRPENHPPID

LQLYQLLRWSYDALPTSDLKNCFLSSAIYLEDANINVTELVQ

>LL_021438_T.1

MASASSSSSEFFELFISHHFPVIKNTFVSRLYRLLISHGFGVFLNKPETQVGHDIPSQIE

ATIRNASLHVPIFSPTYAESQWCLNELLLMVESVKSGRTTILPVFYNVKPSDLRHGAYAE

ALSCHEQKHQVHDLQTIRNWRDVLCSVAEITGIDLDNGDEDELLDKVVQNVLKIVPPQLH

VAKHPTGLEDVLIDFEKWTLMHRQGERRDEAKVVGIVGSGGVGKTTLVTKFFNRRKADYA

VSSFLP

>LL_021458_T.1

MAASASFNSTAAPSNTNSKYNYQVFVNHRGEDCKKTLASHLYHRLRLHGFRVFVDKPEMQ

PGDSLTSQIRTSFVPIA

>LL_021487_T.1

MLESGAPIIPVFYHVRPNELRREDGKYAEDLRNLENMKTHDNQEKPVYNSTTIKKWKDAL

SRVSFITGLDLLDTCNGDEGKLVEMIVQSVLKNVKMPGLENSCLVNLDTENDTHMLDRHR

DMGIISKAPKSDISQKDNDDSLQQSNDHHTAIPKLPNCGQKRKPKW

>LL_021529_T.1

MAMAASHSASTSIAADSNTNNCYDVFINHRGPDVKKTFASHLYRRLISYGLRVFLDYKEL

QEGEDLTSQIKGAIKNASAHVAIFSPTYADSQWCLDELVLMVESSTPIIPVFHNVKPAEV

RRTRGEGVYARSLYNLEKKTAYDSQPRYSSITTEKWRDALSRVADISGLDLEAFNGDEGE

LVDKVVDVVLKNGVKTQLSVARYPTGLDDKVKDFDRKISLQNEHSGRIQRKIALQNEHEQ

YSGRVQIVGITGLGGVGKTTLAKQLFNRESSNYKKSYFLSDIREKTKSSLHSLQSEVLRG

LFQYDRHVDSRHQGTEILRKHLSSSKILLVLDDVDDVDQVEALLPVDDLCHDSLILITSR

DKDILTRSRVPESSIYKLTGLNTERSRELFCSHAFCQPLPPSGFENLVDKFLRACGGLPL

SLKVFGGLLYGKSDICYWEDELSSLEQTLPGEIQKRLQISYNALPSDEQQMFLDIACFFI

GQTRDTAIQIWEVSRWNGRQGLRSLLNKCLVEVEVENENKIRMHDHLRDLGRDLAAKDSK

WPRLVWRGTENIDDLSQQSFGLTDLRGISMLRSEYRPEDDKEDEEYKHHVVVYRDDHDEE

EYRDDDALHQFFSSDEMSKLQLLHIDIKSDLRGLHSRNLIWLCWHNCPCSSLPTWISMKK

LRVLQVRGCQLETLWQDELQAPSQLRQLQIDAPGLTNIPKSISHLKHLKHLRLSSQCMRY

LPDSFGNLTNLEHIDLSNCGQLERLPSSFGNLIKLKYLYLRWCTNITLSSGSFGNITTLE

YIYLWDCHKIVEWPSQFAHQRSLKILSLYLSNLKEWPTAIGEPGRLEKLVLKTPLLKTFP

SSLGHDLMNLKSLELLDCRSLKSMLEICNFTKLQILKIGGCFEVRELSVETLVSLEEFLV

DECVQLKRIRGLAQLTKLRKLSVKGCSELEETPGVENLKSLEKLQAYDCMKLKSIWCSAQ

QIH

>LL_021554_T.1

MAMAASSSTSASASTSSNPNSNSNSSYDIFISHRGPDVKNTFASYLYRQLHSHRLKVFLD

REELQQGDSINPQIEGAIQTASVHVAIFSPRYAESTWCLNELLSMLKSGAPIIPVFYNVK

PAELRWTQGNYAQALQQKRQKRHDSATVELWKKALSSVAEISGFELEACNGDEGELLEKV

VDSVLRKVKKTPLNVAKYPTGLDEKVRDFENKVLLRHQLGGIKGQVVGIVGLGGAGKTTL

AKEIFNRKSSHYKHSCFLSEVRENVGKGSLTLLQSKILKELTGSAQIDNKDEGIEKLKIH

LSSSEILLILDDVDKVDHVDDLLPDRTSVHSNSFILITSRNKDVLRMSGVGESSIYKLDG

LSEQHSLELFCCYAFSRPDPLPGFKDLTQKFLKACQGLPLSLKVLGALLLGKNDMSYWHD

QLARLESILPEEIQERLKISYDTLDREEKQIFLDIACFLIGHDMNKAIRIWDASHWKGRL

GFQNLQNKCLVEVVEDRWCYDGPLYGISKKINMHDHIRDLGRDIA

>LL_021560_T.1

MEIKMWEKGVGILQKNLKSCHALIILDDIDDADQLDAFLPIKGVLDPKSLILVTSRDKHV

LGSVGIAKSSIFHLKGLNNPHSQQLFCSHAFFQPHPRPGFENLVVKFVKGCDGLPLSLKV

FGALLCEKDESYWEEKLLELHKLPSKITARLKISYDSLSQKEKQVFLDIACFAIGEERDK

WIRIWGGLVEFQTLEDRCLVDVDSGRMIRMHDHLRDLGRDIAEEESMPRRLWRSIEDVND

LFEEQSSSVIPEVRGIMMSYPWIVTEDSRKVEISLNESSSHEAFKIRKLQLLDSKGDFVQ

RILSRVRSPNLIWLRWNDCPCSSLPPWVPMDHLRVLEVTSFKLNRLWQSESQAPLQLREL

YVNAPLLEFPKSLGKLKYLEKIDFFDVDLPTFPKEFGDLCSLKYLRLNKFDKLQILPNYF

GNLTNLQYIYMYNCESLKKLPNSLGNLTNLQHIDMYGSRQLQSLPNSLGNLTNLYHIKLS

RCKSLKKLPNSLGNLKNLQQIDLDECRKLQILPNSLGNLTNLHRINLSGCKSLKMLPSSL

GNLTNLQHIDLDDCNNLKMLPDSFGNLTNLKHIDLYRCSSLKMLPDSFGNLTILPRIHLV

RCRSLEMLPDSFGNLTNLQHIDLSWCSNLQMLPDTFGNLRDLQSITMAGCKRLEMLPDFF

GNLTNLREIDLSDCSRLEMLPDFFANLTNLREINLCGCSNLQMLPDTFGNLRNLQSIRMS

GCKSLEILPDSFGNLRNLQSIHMSGCKSLEILPDSFWNLTNLQLINLSNCSSLEMLPVYL

GNLINLQRINLSACSSLKMLPDSLGNLTSLRHINLSACSSLEMLPDSLGNLTNVQCISLG

GSSNLKMLPNSFGNLLQLKHLHLSDCCSLTSETLVNITTLECLDLSGHSQMRLLPPEVGR

QQNLKRLHLLDTNLKYLSSTTTECLSNLEVLIIGSPLLKELPRSFGSLTSLKELRLHGCP

ELKCLPNSVGLLTQMTLLDISDCGIQYLPAAVMKMNSLQTLRVRSCPLQELPFKKGERER

EKLLTESTPGRRRRKRKRRASREMVPSIDKGLFQLKELELYCTEISEVSFPEGTCPNLEY

LMIRCCDNLKEVRALPVTLRVLGFRGCVSLMKIKGLSDLVNHRYFRCLEIIDCPRIEVDK

LPGIEALKHSLVPPYYYNRQMEREREEDEMIELQEGNLWTPNSAGFFYSPGPYPRHPDNL

FIWFCISSLF

>LL_021581_T.1

MASPSNTASAASTPHGIGNNYPKYDVFINHPAPDIQYTIAGQVYSRLNTHNLSVFLDRQA

LQTGVNLASKAASVHIAIISPGYAVSAWCLRQLVQMLESGKTIIPVFYKVCRAELWWPDG

VYAQALRKHERQDQYDSHIIEGWRKALSAVAKINGVEMLLYNSDKEFLVDKVVEAVVKMF

GIPLPASRAYEVKGADSDTIALLHRKGANGPTVEMALQQPPVYPMASAYGIGNNVSPFDV

SINHRGLDVKRGFASYLYKRLSAHGLRVFMNREVLQTGVNLASQNEGAIKPASVHIATIS

PGYAESTWESTWCLRELVKLLKSGKRIVPVFYKVYPSQLRQTDGDYAEALRKHERETRYD

SHTIEGWRTALSALAHRRGFDLMAYNGDEGLLVDKVVEGVVKTFGIPLPASQAFRQDFTV

ISKGCKWSSY

>LL_021717_T.1

MANKICCSCSRFFSCLRNSPDAISSAEGAEHSSDVPAVGSSSAAVEEGNVDVDLDALIEY

ISQVLDLIKEGKSSQKKTKTAQQYLPTTSKIFNKLKDFLSESTNFFKDIIPSDGIKTVAS

VILQGMGMGQLVTTGLVVVADILERFDDVAANKIECLRLLKEMIFLAKLVKQFKERPQLL

EGMDDVIKESTELIVASSIECLSQMKSSKFSKFFSTKANQEDLERSTKLLDDQYKHIYAQ

MGISLFDAQAFKKAPLLSRGYPEHAVGLEEPIKEVIDLLQWGSEENAMAIMLNGIGGMGK

TTLADAVFSRLNLKGCKYSKVQLFKYIGSIPDIVDLQRLILKDLMEPQESNPDIRTPEDG

QRVLGYALENVTAFIYIDNVLGENELRQLLPRDMKKAKKVRLLLTARDINVRRGCPVTVQ

IYRMRSISSAEAMNLLNREMSNSMAGELNSSQLRILTDICGGIPLMLTLVAGVIRFEGDK

QKAYRIVMQDRQKLKGQTFGDKEHYLFAYDSLPEMCKDPFLDICSFFNGWDWNTVADIVG

ESELDMLEKRALVTKDLDGVAWVHDVILTIGLQKTESVRFKFTSASEVEELLYHNEEKVP

NH

>LL_021739_T.1

MADFGIGVASNVTGIIIGEVIQKMKDIIEMEENVGDLKTDLTSMKLSLGNIETQFEEQQR

AIPESVRNCLERMHNALGVVHNLILRAEPQPRCLGCWRLRNPNLSTQIRDWKTGFDRLFQ

ELEMVISTSANAVELVSSAAPRPEVLLQPVPDSGFVGSVIQSHQMQLQAWLTQPHRQARM

IGVYGMGGIGKTSLLKLVYNHYKEVSGVFDVIIW

>LL_021742_T.1

MRDRLRSIKALVILEYVGDSKQLEALRGDWYGSGSRVLVTTLDSQLLIDGHVDFVYEMRG

LDSDHALQSFSWHSFMRDKPDEIYEELSQTAVNICNGFPLLLEVLGAHLYNKDRSFWVET

LENLVDQDKYAILRLCYNGLNSEQKEMFLDMACLFIGRKRESVISFWEASHCDPNKGLTG

MILKSLISLDEDDRFAMHSELRDMGRAIVAKESEEAGERSRLWKPQEAELVVMEGKGTEK

VRFLTYLQQEVILQTNNLQTMCNLQLLWLDGALVEGDFAQMPPEIKWLRWENCPLKCLPC

EWNMKHLAVLDLSFSKDIEAVWTTFSDEEGPKNLKVLKLNHCTNLQLLPDLRNHTSLIRL

ELCGCTELKELPECVGLLKNLKHLDLSNCYNLTHLPDTITNLSSLEVLFLSNCHNIRKLP

ESFGELTVLKKIRMNGMPLKELPGTFRWLSCLEKLTLHGCYKLASLPQSIGELNCLRYLD

IHGCSSLRSLPDSIYELESLRHLDMTDCKKVDLGEELCNLVCMERLILSNCPAMRSLCTS

IGRLNCLRHLNMSNCASLFRLPEETGNLVCLEDLLLNKCHNLCELPQSIGNLSKLRNLEM

EENYSLSLLPPSFSRLTSLKVLKSGGCRLPQNIGKFSSLEILYLKSYKVSFLPPSFSRIP

RLTKLHLHHCTELVELPSLPKKLLQLCIQDCLGLRKISSLSHMKKLKLLRIHNCRELVEL

PDFASLQSLQDLSISICEKVKRITGMEGLKSLRKLQVAGCRTSVVRPLQLIKETLCLELF

SFSANGVPENLQHKMRDSGDYLLNVILEINEPCTGVILCVMVRFNNSVTSLSIEMSIVRD

GKEIFNTKLPIHSKNVVGDRVFVHILRQNHPLVMMLQSGDVVRAKAEGDEERMLIRSGGM

QFFYKGEDGRTEDLILGRLGKDVTLLMKKYNEDEDDICEIPAQKI

>LL_021797_T.1

MDEPAAAQQSHGYTSQVPDNPVGLDNPIDDVRQILLRDDVHIVGVTGMGGSGKTTLASAL

SNDPKVKASFHEIIFIAVSELNGNTNSILNIIETMWDKIRCGPKPRFQSIEDAHNQLQEN

LMRKTNLRTLVILDDVWSPLNAKQLLFKAKGYKTVITTRHDSTIPKTSYSRLYKMPMLEE

AHGLSLFCQYAFGQSSIPTTEKEVLVKQVAAECHGLPLAIKVIGSCLRDQPWPVWESAKD

DLSRAEYLNDLHKEELLNCLETSVNILDDELKQCFLDLGAFPQGGKFSVDSLLDIWEYVR

GMKRSYAFVVLLELASRNLLYLRDDLGRGAITYKCAAELSFSQHNVMRDLALHLASQNSE

RLFMPVKDDRIPAKWQILGDQSSRAKFLSIHTGAMGEQDWCQIDFGEVEALGLFFASSKY

CLPTFLHTMSKLKVLIIYNYGSHRAILSGMPSFPSLGQIRSLLLNKLTVPPLYKSYKSWE

RLEKLYVCLCEGLGSITLVDNELEA

>LL_021838_T.1

MLKSGKPIIPVFYNVKPEDLRWTGWTGTERDGPYAKALHKHEEKLRHDIQTIHQWRKALS

DVANISGLELEAFNGDQDELLDKVVQTVLKMVPPQLDVAKYPTGLGEKLEDFESTVLSQQ

QGERRVEAKVVGIVGLGGVGKTTLAKDFFNRKRSSYQGSSFLFDVRENATRGNLISLQCK

LIKDLKNQDIKFDRVEEGIEIVKKNLSSFHALLIIDDVDHVDQLDAFLPIKHVLDPRSLI

LVTTRDKAVLRSASIVQSSIYHLSGLNRSQSQQLFCSYAFSQSYPPVGFEKLVNGVLKIC

DGLPLSLKVFGALLCGESDKSYWEEVLNGLNILPADIQKILRISYDSLNEEEQQIFLDIA

CFSTGEDRDMWIRVWGGSGWKGLVGFRNLQNKCLVEVDHENKIRMHDHLRDMGRNIADSS

TLMAPRLWRPMKNLHDLLDQLSPSSVNNFQDFLFKMILHIPCCNCLMFLKHTQTEVRGIK

MASQSIQRDNWHEVLPLNESSSNEVFEIKKLQLLEAEGDFVERILTWVRFPKLIWLRWNK

CPCPSLPSWIPMHHLRVLEVSGRNLERLWPCGSEVPLQLRELNIAAPLLEFPKSLGQLKH

LEKIEARYYVVAGVPLTTLPEEFCDLCSLKYLTLRGYNNLQILPNS

>LL_021888_T.1

MANKSCSSCCSTFFPCFPCFSARKSPPSSFLPNLSDSDAISSSANVNIKADSPTVDTEAA

VQRHDTQTLQSSPQASTSHVASSTAEGASVPNAAADAMFSPSANVAFPAVANQSALLTSH

IPQTRPNHPDASTSHAAASSSSAEKASSTVVTAVEKLDDHKAPSGGSSSASTVQTMSNAI

HNISKDVTAAVEGENVNVDLDALIKHISELLKLVKEEGKKSSVPKMVQRYLPTSKSWFKL

REFLSEATNFVKDIIPGDAKNIVHGLLQNMGTAHLATSGLLVVANMLERFEDVSNNREEC

LRLMKRMAFLAKLVKQFEERSQLKEGMHDEIEAAIGLIVESSIMCCSQMGSSAFCRLFST

SVNKEKLAEFRQKLDDMNTRIYIQIGICIYDASQREKAPLSKLYPDHAVGIEESVKEVID

LLEWGSEESVKEVIDLLESVKEVIDALTVIVHGFGGMGKTTLADAVFSLVHVEGCKYSQV

QLFKDIKSTPDILELQKLMLKDLMESEKIPDIRKHEEGQRQLGCMLEKVQAFIYIDNVLN

ANELRELLPKDMNKAKKVRLLLTA

>LL_021891_T.1

MQVDDIDGESIETLLKQHLASSEIFIILDDVDHVDQVDALLPDQAVIRSDSLILITSRYK

NILTSSGIEDSSIYKLSGLNEQHSRELFCSHAFNQPYPLSGFESLVDKFSKACGGLPLSL

KVFGALHRGQKNPSYWEEQLDKLGRILPNEIKQRLQISFEALDVEEREIFLDIACFFIGE

KRDMAVRIWDGSHWKGFQNLEDRCLVEVDGENVIHMHDHLRDLGRDIAKAPGLVRGTDNI

DDLLQQPSPVITDVRGIRTVYGRSEMSWHRRILQVPESEGGLLERILTRVHSPNLIWLRW

YRCPFSSLPSWIPVKNLRVLEMRGKEWTSLWEGEWQ

>LL_021927_T.1

MADPCSSSSMAGSASTSSSQNFQVFINHRGPDTKDTFATPLYHSLTQSGLLVFLDKSEMQ

LGHHIPSQIQGAIKVASLHVAILSPRYAESEWCLNELLLMIESKKTIIPVFYNVKPSDLR

WTGEEKYGPYARALYAPETKKRYEAKTIQQWRTALSHVANISGLELEAFNGEEEKLQRQE

ELLGKVVQSVLKIVPPPLDVAKYPTGLGEKLKEFERTVLSEHQREHRVGAKVVGIVGLGG

IGK

>LL_021966_T.1

MAASTAASSNTNSDYNYQVFLNHRGEDCKKTLASHLYRRLIRHGFRVFLDKPEMKVGDDI

EPQIREAIATSSIHIAIFSRGYAESKWCLKELDLMLKSKVARATTIIPVFYDVSTSLSEV

YGQALCNLENEPNIQVWKEALSRVSKISGLELKAYNGDEGELLDRVVQLVKEKIIREIPP

LYVADYPTGLDKKLEDFEQTVLLQQQEQHSESTKIFGIVGACGVGKTTLARELFNRNRLG

YDRSCVLFDVRDQPLSSLQNKLVKDLKSLNPDIDNIHEATGKGCQALVILDDVGNDEQLN

ALFSTINGVLRSDCLILVTSCNKEALKCSGIREASIYKLKGLDREHSRELFCRHAFHQPQ

PKAGFEEIVEQFLNISDGLPLSLRQIGAQLFEKELRYWKAQLRKILGSTLPAVIQSRLKI

GYDDLDDQEKEVFLDTACFFIKQDKDMAIRIWDASNWEGWLNFHNLENKFLLAIDDKNCI

IMHDHIRDLGRNLAEKELPKFHRRIWRPADKLSLNASLQPIVRGINRVEGPSGQSFVNVE

LNDRYVDMTQLQLLRAEGDCLASMSNLLRQSDLRWLSWDKCPYASLPSWIPTRNLRVLQV

AGRELKLCHGESELPLMLEHNENIETKDTIQLYRGKRFLNSARCSTS

>LL_021979_T.1

MAHVPFASAPSSYTACSVSTSSYPVYDVFISHRGPDVKKTFAGHLYQRLRSRGLLVFLDK

QELQAGHQISFQIAEAIKVASVHVVIFSPHYAESTWCLEELVLILKSRATMLPVFYSVKP

LDLRWTTGLYAEALRKHEEKRRCDIEKIQGWRNALSTAANISGFELEACNGDEVELLEEV

VQRVLKEVPKPPLYVAKYKIGLQEKLEDFEKTVLSQLGEKGRPEAKVVGIVGVGGIGKTT

LVKEFFNNRRSNYKVSSFLFDMRENATAKSINLLQSKLIKDLKDIDIKIDRWEEGIGMIE

KNLKSCHALIILDNVDHIDQLDAFLPIKPVLDPNSLILVTSRDKHVLRSARIPESSIYHL

KGLNRSHSLELFCCHAFFQPHPVPEFEDLVNGFINACDGLPLSLEVFGALLCQENDKSYW

EEQLNELQMLPPEIHRRLKISYDSLNPEEQQIFLDIACFSIGENKNKWIRIWIGSGWKGS

VGLRNLENKCLVEVDGENIKMHDHLRDMGRSIAQEGSMPRRIWCGTTKTVYDLLEQPASV

TEVRGIKMVPRSTLTDTGEEALFRSKSKMPLSKSFKGEFFSISKLQLLEAEGDFVKALLA

KVRSPNLVWLRWNKCPLPFLPSWIPMHDLRVLEVDGSKLKTLWRSESQAPLQLRELNIDH

APLLEFPNSLGQLKHLEKITVDYSWTGLWPSLNDIPQLATLPEEFCLLRSLKYLKLAFCQ

KLQILPTCFGNLTNLQHIDLYECQSLKMLPNSLGNLRNLHYIRLAYCGELQILPDSLGNL

TNLQEINLEGCTSLEMLPDSLGTLTNLHGINMSGCLNLKTLPASLENLTNLQHIRLDDCK

SLKMLPNSIENLTNLKNIDLSGCCELQRLPHSFGNLTNLQEINLEECKCLKMLPESVGNL

TNLKNINMNACQSLNMLPDSFSNLTNIQYITMSDCESLEMLPDSFGSLTNLRHITLSHSK

RLEMLPNSFGNLTNLQHIDLYGCESLKMLPDSFGNLRNLRYVTMSHCKSLQKIPSSFGNL

TNLQSINMYGAEKLEMLPTSFGNLINLQHIDMQGCQSLKMLPDSFGSLTNLKYIRLCRCE

SLEQMLPNILENFINLQYIDISHSSKLQMPSNSLGNLLQLKHLHLSGCTLIISEETLGNI

TTLECLDLSSCLQMQVLPSQVVHQKYLQKLNLLGTNIKELPSAPIVCLSNLEVLNLGSPL

LKVLPQWIGDLISLRKLQLRGCRILERLPDSVGQLAQLKVLRLHGSQELKCLPDSVGLLN

QLRVLDISNCGIQYLPPAVMKMNNLEILRVCGCPLRDLPFKNIQGEGEPESAPRRELDSS

IHTAMIHLEVIHLYLTEISKVSFPQGVCPNLKSLSIRRCNSLVEVGALPTTLIRINLRGC

GALKKIDGLYRLAKLAALDMTGCCALCLEAVPGLETLKSLKILKSDFETCYPNDEGEEEM

EMEDANLQGVLWCDDFCVSREFYPKFH

>LL_021980_T.1

MLESGAQIIPVFYHVRPNDLRRKDGKYAEDLRNLENMKTHDPETNQEKPVYDSTTIKKWK

DALYRVSFITGLDLLDTCNGDEAKLVEMIVQSVLKNVKMPGLENSCLVNLDTENDTHMLD

RHRDMGIISKAPKSDISQKDNDDSLQQSNDHHTAIPKLPNCGQKRKPKCKRYLASKISKS

PRRKKVYVRVETKYRDPGKS

>LL_021993_T.1

MDSFNGKNACSRLDATLTGLIEIFRPICLSNLRKPQNEQNFVYEKFLNTLMKGIEFLKKW

ENTRCFNFLHSLRYGFQIRQLEKEISDFLHYQMPVHIFLEVKSLISELNNLRQLYELGSM

DESTMNETIFKNVSKLTNDPQQNAVMLLQQMGADDMLDGSFVECPCNYDGPGKSDLVVGL

EKNIWNVKRTLLQSEVSIVGVQGMGGLGKTTLALALSDDKEIKGVFQNNIIFITVSESPN

LKVILETMWEKIIRKKRPEFQNVEEAHRQLQQQLLLQAKPTLVVLDDVWSRAKLEQLLFE

GVGYKTLVTTRDRSTIPKTTSTQLYELPLLDDADALSLFCFWAFGQRSIPSTADEHLVKQ

VQAQCKGLPLALKVVGSSLHGEPRPVWESAKKKLLNAESISDYHKEGLFKCLETSIDVLD

EEARECFLD

>LL_022011_T.1

MDSVGCGSLILITSRDKNLLRRSSPKIVLYHVRRLSRQHARELFCQHAFRQSIPSQEFDD

LVEDFLYICEGLPLALKVLGGQLLGFSDREQWKRQLEMFSTTLHNDIIKTLKVGYDALDH

ALDKEAFLDVGCFLMGEETELAMRVLNGLYGSSIVHSLERLHQKCLLDFHYDVQSNDEIE

CRKQENRNIGYNDLSLQYRGYKLTMHDQLRELARHIAREQFRVFQKPHTPLRLSSSDDIL

QLCYQDKSNTPSIWGIRNTKEKPLDFSLQYIRGERVRLLVLEFPKEFPLRYLVSPEISGD

LVWLRLRNFRFDSMRFLLSTSVRSLTVLELESYGESIEEFFKILMDQFMGNPDKAPSRLN

ELNINILYEPLLTASSNRSTFDAGPSTKSSESYISSPVQQGGVTPIFLLSFLKWIGLENL

GRMVLTNLTFLQSLRIDSGSLPSLKHLDLSGCTNLKELPKSFSKLMQLQHLALRECKNLS

IPLQILGEISTLEYVDFEGCDKLVALPQGIPYQ

>LL_022091_T.1

MSSPGRVSKDINEWKALFDCLFQDLQRDFSIFVTAAHQIIHRDPIPAFSAKGSEQLYQSY

DALPTSDLKNCLLYSAIYEKDAKINVTELVQMWIAEGLVRSKQETHLMDMAIGRTYVNLL

LDRRFFHSASNEENQYYSGWNSSDYQSIRVDGDIREMAMYIGEKERNCLLKARQGIQNFP

TVHNADCERISLHQNQIANLPSELPACPKLVSLILSGNRISVVPSGFLVGLTSLKVLDLS

YNHRISSIPSSVGQLQQLQLLRLENLFNIKELPEEIGHLSSLQFLNLKCCLSLESLPSQI

GQLQNLKYLNINNTRLRVIPHEISQLTALTTLDIEDVELRSICRLKGLINLTMLRINVKE

GNTSSGIEGGIMGTWLDMRHLYLRFTSYLGYVKDDYVMDDLPGDMQNMRKLQSLEMWRYR

GLSLPEYSCKFQHLQYLELNWCPHLKELFPLERLPSLRYLRLYHCSNLKELGISGKASGG

FLMLEKLVLSGLEKLESMAGPSNHGEWNERTLPKLSVLKITVCPYLKRLPVGMDKLLNLK

TIKATSDWWKQIIWENDSTKLHIANIFKA

>LL_022094_T.1

MGVAKYTITIQDLNQEDSWELFKHHAFPYHINGNLPSNIDDEKAKLVCNKCAGLPLGIKV

VGAAMAGSTHPQQWEWALHRLENADNLDAYLKLSFDAMGNEDFNMQLCFLHIATAFPEDA

IIEAQEAIPLWMGGGLLARKKVEHVPRHVLADHCLIEPMIRNTEGCPVCLRMQDPFQIGK

LYLDLLAERCLIQPTARDADGLVLCFRMLNIVRDWAILIAKDEENFYRCVGNSNGYGLEH

LQNNKESSKIKRIFLSDNKLSSLPQFSSSSEMRSLLIFENTGLTKIPKKVIGSMLSLKVL

DLSGTSARLLPNVACLKELICLKLSGTRIKELPKSVTGLGHLEILDVSFTDINELPSDIH

KLRSLRYLGLRGCTYLQYLPSNISSLLSLQYLYMDGCSSMWTKPSGNKHKNVMDGCKKVA

PLQSLGDLIQLKQLALQTNGEQQLQHLPDGNRQQLEQLLKSMTEMDTLLLVLPEMVSLPL

HISDMSNLRSLSLKCSKLIKMDSFICSSLTETPFSMKNDVTHLKHLSYLKFYECDVLKEF

QHLHKLPYLR

>LL_022104_T.1

MAASTDAPSNSNSRSNYQVFLNHFGKDSKKTLASHLYRRLRRHGFRVFLDNQEMKVGDDI

DPQIRGAITTASVHIAIFSPGYAESKWCLKELDLMLKSKFARATTIIPVFYHVSTSLSKV

YGQALCNLENEPDIQIWKEALSRVSNITGLELMKACNGDEGELVDRVVQQVEGIINREIP

PLYVAAYPTGLDKKLKDFQKTVLLQQQEQHSERTKIFGIVGPCGVGKTTLAREFFNRNRS

CYDRSCVLFDVRDQPLISSLQNKLLEDLNSLNPNIDNIHEATGKGCKALVILDDVGNAEQ

LKALFSAVNDVLRSDSLILVTSCDKKTLKCSGIREASIYKLKGLDREQSRELFCWHAFHQ

PHPETGFEDIVTQFLNISDGLPLSLQELGAQLSGEEWWYWKAQLRKILGSTLPAEIQRRL

KIGYDDLDNQEKEVFLDTACFFIKQNKDMAIRIWDASSWEGRLNLQNLENKFLLEIDSTN

CIRMHDHIRDLGRYLAEKELPRFHRRIWRPTDKLSLNASLQPIVRGINRVKGKSDQSSYS

YPQVDMTQLQLLRAEGDCLASISKLLEQSDLQWLCWDEFPYASLPPWIPVKNSTVLQVAR

CEPKLRHAETEITMEHQWILCIAILCRPDPGQGTIAYVLYDKVQDITIKKMRSIHGETTN

NKAYYIALMEGLEVALENGVNEIAVYMNSKLIIDQMTGRCKVRSKNLVPLCNSAHELARK

FLSFTMYHDEMDGDHMNASRMCKDLLSGVMSSPVKNVN

>LL_022124_T.1

MMYSSITGVQMLKKFASHMYHRLLPHGLKVFLDERELEQGECLTPQIKGAIASASVHVAI

FSPRYAQSRWCLDELVLMLESGKKSGATIIPVFFNVQPSDLRWTDKGRYARSLLSHESKG

RYNKETLADWSAALAEVSDISGFELKSCNGDEGELVDKVVQQVVKKVKKPPLRVAEHPTG

LDNKLNDFERKVLLQQQHSGQAKVVGIVGLGGVGKTTMAKEFFNRKRSSYGRSSFLAEVR

GAAARSSLTSLQSQLYEDLTGCAGVMNSVDEGTGMLRRLLSCSSVLIILDDVDHADQVLA

FVQPGNDVLHSGSLIIVTSRNKQVFTVLGIEVSSIYKMEGLDKEHSQLLFCLHAFNKDLP

VVEFEEVVGKFLKLCAGLPLSLIVLGSLLRGKKDLGYWEGQLQKILKSGLPKDIQSRLRI

SYDSLEEEEKNIFLDIACFFIGTKKDTAIRIWDGSGWAGSVGFLNLESKCLVEIDSKNCI

RMHDQLRDLGRYLAEKEPLGSGLRLWRPTDSLFRNVPPQSTVRGIKIGLGSRIGEECSNH

IVDMRGLQLLSAPLAFLESISEVLRKADLVWLSIQSISPIPCLPSWIPIRNLRVLQLGGL

DGYDFLEDEVHQLVRNLRVLALGGYDILEDEEEIDQLLKTWRGLQIDQLLKTWRGLQPEG

PLETLWETENAAPVNMRELIIGADLLNIPKSIGNLKHLENFSLYGNRATRVSLKTLPDEF

CNLQSLKHLALTQCSAMESLPDSLGNLTNLQTIDLSGCNGLQMLPDSLGNCTSVQTIDLS

GCSGLQMFPHSLGNCTSLQPIVIAHTFASTSASASASASAHMYDVFLNHRGPDVKKNSQP

YVLSSSLLWIESLSRPAGIAERRMLNSSNRRSHCQRLRPRSYFL

>LL_022135_T.1

MASASTSTSSSVFPRNYHVFINHRGPDVKKTFASHLYHCLLSHGLQVFLDQPELERGENT

PPQIERAIDTSSVHVAIFSPGYADSDWCLNELVLMLKSDAVIIPVFYKVKPSHLRFADKG

YTGAMRIFAYLWEYLCESMPKRLLMADKSYTGAMRNHEKRYDQQKITNWKRALHDVSLIS

GFELCNGDEGELVHEVARQVLKKVRKEPLPVATHPTGLDVKLQDFEVILERQQHRDGPNV

VGIVGLGGVGKTTLAKYFFNSKTSDYQKSCFLPDVRDAAARHSLTSLQSKLFRDLTGRDQ

SIDNVVDGIAMLKDRLSCRQALVILDDVDDVVQLNALFSPVKKALPSGSLVLLTSRNMDV

LMKSAQIAESSIYNMTGLGFEHSQELFCSHAFLQPHPPAVEYNKLVEKFLDACQGLPLSL

EVFGALLYLQNLEVWEAQLLKLSQVLPEKIQSSLKISYDSLDKQEKQIFLDIACFFIGTD

RDTAIRVWDGSGWGGSLGLMTLQSKCLVTVDSENRIRMHDHLRDLGRDLA

>LL_022155_T.1

MGKTTLADAVFSLVHVEGCKYSQVQLFKDIKSTPDILELQKLMLKDLMESEKIPDIRKHE

EGQRQLGCMLEKVQAFIYIDNVLNANELRELLPKDMNKAKKVRLLLTARDENVGRVCPME

TPTKIYHMKGISTREAASLLKKDIKEEIDSSQFDEIIEICGGIPLKLTLVGPFISKAKNK

QQAYRRLMQEKGRLKIEPFDEIERYVFAYDDLPDMCKDPFLDICLFFKGWDWDTVADIVG

DIYLEMLESRALVTKDTNGVTSLHDVILTLGCQKTDRITRFKFTSARKM

>LL_022173_T.1

MKKTHDSTTIEKWRNALSRVSEISGFDLEVFNGDEGMLLERVVDDVLKSMKTHLNVAEYP

TGLDEKVEEFESTVLSELHSPKINVVCIVGMCGVGKTTLAKEFFNRKSSKYMKSYFLFHV

GENSLISLQSELFRGLFQLDMHINTIDEGLGMLRRHLSSSKVLLVLDDVNHVEQLDALLP

VDVLHPDSLILITSSHKNVLTHSRVRVSSLYNLTGLNIQHSVELFCWHAFRQPHPKSGFE

YLVQKFSRACSGLPLSLKVFGGLLYGNTDKDHWDSIQSMLPLEIQQNLIVGFDSLDSKEQ

QVFLDIACFFIGQDRDTAIRIWNGSGWKGLFCFRALQDKYLVEVDNENKIRMHSHLRQMG

RDLANVQEPLRLWSGTKYSDILLRKFRVSGQSVDLLLLFNKNLCTAFVIFHCFYRINH

>LL_022187_T.1

MAKKSCCSCCTFLSCFSCVSARNTTDDIQTPPLNPAGSGLVDIQTTPLNPDASFSHHPPA

AASSSVEGTSSAAAPKASLDSNAANGNTNAPKASLDSNAANGNTNVASPLIATQEALLIR

NDPQITPSNPQAIPSHVPEKEASTSEILTIVKQDDPQSSGSSSSSTLQTVSVDVDNIPKA

AAAVEGDNVKVDLDGLIKHISELLEHIKDEGKSSSLPKLVHRSYMPTSKTFSKLKEFLSE

ATNFVKDIVPGDTKTIVRGLLQGMGTAHLATTGLLVVANILERFEDVSNNRDGCLHLLKE

MIFLAKVVKQIKEHSQLQERMHDEVKDATELIVEGSIKCCSQMDSSRFSKFFSSSVNKKE

LGELAQHLDAMYRHIYIQMGIEIYDVSHCQMANLSRGYPEKAVGIQEAIREVVDLLEWGS

EKNAIAVILHGFGGMGKTTLADAVFAVVNIEGCQYSMVRLFDNIESRPNIVELQKWILKD

LMGSENIPQIRKHEDGQRELSRMLEEKPAFIYIDNALSKRELAQLLPEDTSKAKKVRVLI

TARDLNVRKACPLKTVLKEYRMRGISSMEATNLLNIEMFNDNIEEILHSYKVNQIDYIIE

RCGGIPLMLKLVAKALHVAEDKQEVDAVLDELEKLKGEDFGPDNIESYFFAYDKLPTECK

DPFLDICSYFEGWDWDTVANIMGESELNMLARRALVTKDTNGVVSVHDVILTLGRRKSHG

VRFIFTKASQIKKELDQEGEKHVQKIKGIWLSENRDLFPISATILDSMHKSLRILKLGNL

TRIEGKCNKIFENLIFFQGAVPYLPFDTKELKYLSYQPKDLKLQEIPRSLKHMEFDGRLH

SHAPEISARELQEFQNLRVLRLTRFARLKKLGDLVEGLRELTLSYCQSIEELPSSISMLP

FLRVLRMDYCSNLKHLPYDIGSLNSLQELNLQGCTSLEMLPDSFEKLSLLKSLDLSSCKK

L

>LL_022193_T.1

MASPSNTASASTPHGIGNNYPKYDVFINHPAPDILYTIAGQVYSRLNTHNLSVFLDTEAL

QTGVNLASQIEEALKSAFVHIAIISPGYAESAWCLTQLVQMLESRKTIIPVFYKVCSSEL

WWPYAQALRKHERQAQYDSHTIEGWRKALSAVAMINGVEMLPYNSHKELLVDKVVEAVVK

MFGIPLPASRAYELKGADSHTLALLHPNGANGPHIEMALQQPWVYPMASAYGIGNNVPLF

DVSINHRGLDVMRCFASFLYHRLSAHGLRVFLNREILQMGVNLASQKEGAIKSASVHIAI

ISPGYAESTWCLRELVKLLKSGKIIIPIFYKVYPSELRRTDGVYAEDLRKLERKTRYDSH

TLEGWRTALSALAHIRGFELMAYNGDEELLVDKVVEGVVKMFDIPLPASRAHELGWADSD

TISLLHPKDANGPHIEMGVRQPRSNSNC

>LL_022211_T.1

MGSCSNSVSTSTAYGGNNNYVYDIFISHRGRDVKKTLATDLYHRLHGHQLRVFLDIYEMQ

KGERINPQIEGAIRTASVQVAIFSPTYAESDWCLNELLSMLDSGSTIIPVFYHVKPSELR

WTLGTNGVYAQALRMLEEKKTFDIQTQQEKRRYESDTIAKWRKALSDVSEIIGFHLETYN

GNEEQLLEEVVEEVLKKVKRTPLDVSKYPIGLDETVDDLRRIMSLEKQNGQARVIGIVGL

GGVGKTTLAKEIFNRERSNYKHSSFLFDIREKPLNSLHTDLLKDLTPLNEQIRSTDEGKE

KLRNYLSSQKVLIILDNVDHAEQLDALLLPAKDVLHSSSLIFVTSRNKQVLINSDIAEQS

IYKLECLSQQHSEELFCLHAFGQRHPVAGFEGLVKKFKDACGGLPLSLTVLGANLRGNNL

TYWTAQLRKLSKILPNDIQSRLKVSYDSLDEEEKQIFVDIACFFIGEDRDTAIRIWDGCG

WEGWLGIRNLENRCLVEVDSANCIKMHDHLRDMGRDLAEKEPPGCLRRLWRPTDNLLDLP

GQSPVRGINLVQQRSGQSFENTLALGSPFLGLSRLQLVRAEGDCLDRSIFSRVQSPNIIW

LCWYSCPYYFLSRWFAMESLRVLHVEGSRLKRLWGERDFQVPLQLRELNIIDAPLSKIPK

SIGKLKHLEKFVLHHGCMETLPDEFCHLGCLEHLELKSCSKLKSLPDSFGKLTKLQYIDL

SQCINLQMLPHSFGNLVQLQNLYLNKCYKLTISKETFGKISKLQCLDLSCCSKIEVLPPQ

VENNWLLRELILKGTQLREFPRSIRELIHLEVLILDSQRLKMLPPSIGSLRNLQQLELLS

SQLKRLPKSIGDLSSLKLLWIQSLKRLECLPESIGYLTSLEELVIWNCTELKCLPSCVKE

LNNLARLEMEYCGLTELPFRKDEGERGTIAGSKSSELDTASSFKCMLGLKYIKLRGISIS

EVSFSEGVCPNLQYLKIRDCCSLVKVGALPTTLTELVLWNCFSLRKIAVLAQLTRLRVLN

VSRCFGLEELQGIEHLTSLKSLNAGHCIELQWLPSLETLKSLERLDTSSCYNLKSIKGLA

QLTKLRTLEVATCIQLEELDGVEHSQSLKRLNASACPKLHWDGKIMGQLRQQLNEGLII

>LL_022214_T.1

MADPGLLSGFTGLCMNTAAQGILNYIKIAVRCKKELNSLGVLVKRIDPIIARIQQYRIPN

KAPAVDEWLQELYALLKKGSAVAQECTIPRFNLITRYQLSRRITRLISDIKQHIDSAATL

MAWSSILDEFGEIKESVQALASSQASTSAATSQTLIASTGFFIKEPLIVGQENDLARLEE

LVIDEAAKSPFLRIGMVGKGGSGKSLLLRTFFNSKKVRDLFSDGLLFLWLTVSQSPSFTS

LRYELSTQIAAQTKADFKENMNQEAAQIWLHEKMQEKRFVLILDDVWEDGPQLLEELSLV

RLTSNSTVIVSSRNRRTLAEMGIAENSIIPIGDLAQDDSWRLFSSHAFPYNDGKIPAIVH

EETARLVCARCGGLPLAIKAVGRAMAGTTDRQEWDLAMRILPTANRQDKEAVYDRLRLSY

DALGSYDVNLQMCFLYLAAFLEDEMVAVETEAIPLWIGEGLLAGKMLKQDQCGLDPFEMG

RIYAKVLEDRCLIETIRRDVDGRVLSFRMHDVLRDLAIQIAEHEESFYCRAGKGLTKLNE

DECSGRARFLLNNNNLTSLPQSWSAPQICSLTVRDNNIIEIPGSLFG

>LL_022233_T.1

MDQAIYQVFINHRGKDVKRGLASLIYNRLIEFGLRVFFDKKELRMRDRLKPVIRGAIRSA

SVHIAIFSENYAESRWCLDELFWIFKSSDHKPKIITVFCDVEPGDLRNFEKGPYAKAFEK

HEGRHARRVAKWKYALIEASNISGPKFKKDTSDQGEFLRIIVNSVLEVVKWDQLEVAKHP

VGLDQALAEFESKIDIKELATKVVVIAGLAGAGKSSLAKHLFNSKRSDFSRSCYLSNVRE

KDLTVLQRQLLHDLVGNIMDISDTGVVKRILRATVRRSKILIVLDDVDDGRKLESLLYMD

AVGPGSLILITCRDKNILIDSSAKPLFYDVKPLPAKHAQELFCRHAFSQSDPVEEYKALL

EKFLKICEGLPLCLEVLGKGLAYNPDITYWTGQLANYRPGLPLPQADRVINTLKVSYSAM

DDEEKDFFLDIGCFFLGEDTELAVRVLEGLGYNRDPRQLLKILHQKCLVDFDNYVEYHHE

IESSGQDLHPFLIPTRLMYRNAYVDVESYLPAVLPRGSSKIIMQNQVRELARYIASEHLF

KNMCPLRLSHSIDIQKMLQKDESDLCIIRGIRIGEGQDPPQLSNKIQGVRLLVLEHLMER

SFFGSNIKGDLVWLRLRNSGFTSIPSTISQRSLRVLEFHGPIDHVEGLFNQLICKYDKIP

TELRELNVDPGTSNSCQHVDHSMESSDQNITSTYRRLMPSFYCFLGYIGTQLKYLNKIVL

KNIPFVQSLPIDFSNLKCLRHLDLTGLTHLTELPTSLTLS

>LL_022246_T.1

MSQNPEQQKPMPELGFQVFLSHRGPDAKNHIASLIHNQLTQFHQLRTFIDKKGIRAGHYI

IDEIQKAIKSASVHVAIFSQGYAQSEWCLNELVSMLESTRPIIPVFLDVNVSDVRYIKGT

YEQAFEYYKTKGRVSEVDMDKWKKALNTASQISGIEFKMKENDHGEILNKIVDAVLEALK

CVSPDVAKVGNVSKVEDFPANRDELEVYKEFITEEFKLGKDEGGPDLCELFGTAKVGPDL

TELFEADEGRPDLSELFEDWAL

>LL_022283_T.1

MSGFICLNIDTAIKQILRHISNAVRYEDELKPLKELVTSMEATMRQIQHYRLLITWKIKD

SESAANDRNSESDDWVKDFNKRLDELNASLQDASQVAKDCAVSTHQVILRDQMSVRITEL

TSKIAENLQSMSCWVGDARAKGIVADARHITPAWQTVTPSSGVRDILAPSTTSATISQAA

LDKLPTGRMKLMDEPFIVGQEKYLKRLQNLVTNEKAPTTTKIGVLGQGGSGKTLLLKTLF

NNEKVRNYYRSGFLLWLTVSESPSFTSLRNKLWTQIKIQDKQGAVKNINEQHVEIWIAMQ

NNVDALKNTEKQQVKNGVHETLKLNCRFLLFLDDVWDMSAEKLLNELGIPESVINNPDSK

VIVSSKEETALSSMGIAADYVIEMKGLNEHNSWKLFSHHAFPNDNRNPPGKEEAKLVCQM

CQGLPLAIKVVGKAMAGSTDRKQWRWALQRLSNIKSVYDCRLRFSFEALGNEGVKMQCCF

LLVAATSLEHEILFARHVIVLWAGEGLLSGKTVQEEGYDPFEMGWNYLSLLADRYLIEPM

MRDHDGRVVCFRIHDELRTLAIQIAEKQENFYCSAGKESPVLNVGDQYSGCTRIFWSHEE

SLDQSPNASQVCSLSISESEDSVLKVIPRGLLGSMSSLKFLDLSWTGVESLPDTLAKLKQ

LVCLRLMGVPIKIL

>LL_022285_T.1

MGASSSTPAAAAASASTPTSTTASASTSTYAAANPNYKSYSVFINHRGVDVKKTFARSLY

LRLLDNGLTAFLDEEEMQTGYNITSQLEQVVRAATVHVAIFSPRYADSPWCLDELVMMLE

SEAPIVPVFYRVTPAHVRRTLGDYGRALRTHEKKGRHHLDTIRKWRNALFQVANNSGFEL

ETCNGEQLVLELLDK

>LL_022308_T.1

MASSSTSTSTSTSHGNRVYDVFLNHRGPDAKKTLASHIYHRLTDRGLRVFFDTEEMQPGE

ELTAQIIDAIRTASVHVAIFSKDYASSPWCLKELVEMVDTKKTIIPIFYDVDPSELRWTQ

ARDGVYARALNIIRCILQCKWGEDRGYARALRMLEKKTKLDPQTNKQTPRYESKTIENWR

RALSYAAEISGFELNKFNRDEGLLVNEVIQRVLKTVPKILPNVAMYPTGLDVKVKELEAI

VSPQQQSGSLARAVGIAGLGGVGKTTLAKQFFNSYSHRSNFERFCFLSDVREKAARNSLI

SLQAQLLKELAEQNEQINNPAEGIEMLRRYLPSSCRALIVIDDVDNIDQLDVLFSPVKHT

SSLIIVTSRNKDVLTNAEIEESSIYMVKGLNTQDSQKLFCWHAFKQPYPVEGFEQLVEKF

VDACDGLPLSLKVIGALLRGKRNFNFWKEQLAKISKILPAEIQSRLKISFDGLDEEEKGI

FLDIACFFIGIDRDTAIRIWDGSGWQGWLGFSNLENRCLVEVDSENCITMHDHLRDLGRD

LAKKEYPALRLWRMEDDLHNRPNHSSVRGIHMVYRNDAEHFFENLADSCNMSRLQLLRAD

GDFVGRLFNVNGHFRHLLYLHWDNCPYSSLPPSIPIKNLRVLYIKGGILETLWQEESQAP

LEMREMVIHEELKMIPKSIGKLKHLEKIDLSSEYLESLPDSVGDLENLKSLHLCGCSSLQ

MLPDSVGNMTNLQSLDLVRCSALQMLPDSVGNMTNLQRLEGYML

>LL_022313_T.1

MADLYISTSAASSSTAATPNSNHNYDVFVNGHRQEVKKTLASHLHHLLLLDGFRVFDQQE

ILEEGVSLNSQIRGAIATASVHVAIFSPRFAESKWCLEELVLMLESGKTIIPVFYHVKPS

DLRIRAGNGEDGVYGRALLDLEKNSRNEPNSHTVWREALSRVADISGLELKECNGDEGEL

VDRVVQLVEKKVIETQLLAAYPIGLDEKLEAFKNMVLTQQQQQGVQTTIVGIVGSGGVGK

TTLAIEFFERNRSDYNRPCFLYDVKGAAGRNSLHSLQNQLIKDLTSMDLEIINVSDGKEK

LSSFPFPCQALVILDDIDDVEQFFAFFPPGNELLCSGSLILVISRYRHVLEKSGIDDLSI

YDLTGLNKTHSQELFCLHAFHKRLPVLGFNEVVTQFLYACNGLPLFLKVFGALVYQKDLK

YCETQLRKIAKILPATNIQALKICYDSLDEQEKDTFLDIACFFINEDKDMAITIWDASGW

EGRLNLENLQNYCLVDINSKNCIRMHDLLRDLGRDLAEKERFNCRLWRPTDNLAFPGAFQ

QSTVRGVNMVQGSSDSSFDNPVNPNNSFVDLSRLQLLRAEGDCLGSISNHLTHSDILWLC

WNSYPSANLPPWNPIRNLRVLQVAGSELKMLWMDKNEAPLELRELYIRADVLRFPKSIGQ

LWKLEKIEAKAAIIEPLPEEFCLLLSLKHLSLRTRMNLEGHPLYGDYSHLEMLPESFGTL

SNLSHL

>LL_022417_T.1

MAALVDPLTNKLLDKAFTEMGRVVNIILYSKTWAKELKIMLLSLKPTIDQISKKMFDSDS

SPDRGKLFKTFQADLQDGLRLVENLEKIHSFDIYRKYRYGKRVLKFQKKVYEFLLIHGPP

NVTLEKQKLNEYVMDLGQRLRPFERLIENVKMTTDPISNSLILQQINTIPVFQSSIDGMP

QAAVVTCTYQVPDMPNFVVGLDNPIHAVKKILLQNGVNIVGVTGMGGSGKTTLASALCND

TQVQDFFRSNIIFITVSQFPNVKGLLDTMWDKIIGRQRPDFQSIEDAHNQLQKNLSLKPN

HPTLVVLDDVWSVPTVEQLVFQAKGYKTIITTRQNSTIPKRSDTRLYSMRMLEAANSLSL

FCFWAFSQTSIPTTENEDITKQVVVECKGLPLALKVIGSCLNGEPQPVWESAKAKLSRGE

HISDYHRDNLINRLEMSIDVLDDEQKRCFLDLGAFPK

>LL_022452_T.1

MDITTSVVHLTRNVINEEEKRKLLQTDFQPMLNLLLDLTTVFPDQRKEAQEAFKIWMQLL

HNSFTEARSLLQRPQPGRARRCLDRVICNPRTLSIEINEWKVLFDCLFQDLQSDFSIFVT

ARQIIRYSLIASFKTDPIPAFSAEGSEQLYQSYDALPTSDLKNCFLYSAIYGKDAYINVT

ELVQMWIAEGLVRSKQESHLMDMAIARTYLNLLLDRRFFHNATNEDIRYYSAWDSSDDQS

IIVDDDIREMAMNIAEKEQNCLFKAGQGIQNFPTVHTEDCERISLRRNQIADLPSELPCP

KLVSLILSHNSIRRVPSGFLVGLTSLKVLDLSNNDRVSSIPSSVGQLQQLQLLRLDQLWE

LKDLPEEIGHLSSLQFLSLKKCDSLESLPSQIGQLQNLKYLDISFCKRLRMIPHEISQLT

ALTTLDAWGVSLSGEAESERSISSLKGLINLTMLRIDVEVDVKEGNTSSGIEGGIMGTWL

DMRHLELSFDYSRGDVMDDLPDDMQNMRKLQSFIVWRYRGLSLPEYSCKFQHLQNLELRW

CQHLKELSQLERLPSLTYLELYECPNLKELGIFGKASGGFLMLEELALSWLEKLESMAGP

SNHGVWNERTLPKLRILKIRECPNLKRFPVGMDKLLNLKTIKADSNWWQQIIWENDHMKL

HFANFFKER

>LL_022476_T.1

MGGLGKTTLAKAIFNNICQRFEASCFLSDVRVNDLRDLQEQILTELSGENFPVRNVHRGK

ATMKARLGNIRVLVVLDDISHKNQLDALNSEGCFGPGSRVIVTTRDQQILKLGQAKIYEM

KEFENDEALQLFSWHAFLRPCPDQEYKDLSLGIVEACRGLPLSLEVLGASLYGKTDRILW

IEALKKLQSVKYGDIRKSLEISYEALEDDEKDIFLDIACFFLKQGNPWFLETARLHVISF

WESLYAAPNDALENLILKSLVRVRHVMPRYGVDGLQISNKGYFHFAMHDLIRDMGRAIVA

NESRELGERSRLWNSEDALEVLKGGLGTKRVRGLSFQTSNRNKITLPDGCLSCMTNLNLL

WLDGATIEGDLGQISSNLRWLRWRSCPLERLPLNWNTRHLALLDLTGIGIGAADIGSLTE

ELWNERTAQAKPKNLRILVLNMCLNLRELPNLSDHTSLLSLELERCENLQSLRIEDLPPL

GRLFSLEKLILSYCTSLTCLPKAVGEFKYLRYLDMRGCSSLVFLPDEFGKLASLEELNMS

GCEKLSELPQTFGNLRRLKILKILKCFALSRLPPSFSNLESLAQLNAGHSNLINGLPDQI

GNLRMLEFLNLGDSFASRLPPSISNLGKLATLLLNTCVYLSELPALPEGLVKVDVGNCRQ

LKSISSMSHLIKLETLVLYQCEGLTELPQVRSCQSLRLLDLYGCKNITNLTGTEGLKSLQ

TLYLSGSGISVLNLGQLMKDMCCLQVLSISAKQLPQCLEHKLPNVHSVKVMFNDVICSPL

KKNRKCTAVVVCLVLNLFHSANFNVGGSVSINIISKQSDKEIFGSEITVDTKRVGGRRTD

GLCVLVYRENHPLIMSLEIADAVLVQACSSDDIIIKGSGMQLLYRKEDNGSGDEDVIFEE

LERDLNSLLQLSSGKEKEAEPLKSISSGKEKDVESLKPTFPKGEIKNDGDVDVEEDSKVN

RPFQAGSGSFGSSFNTHTGNAKGGCDPTEKFIRHRQPWQPRVMVSRRYVAIYLILLLILF

NNFKLCKP

>LL_022489_T.1

MANNSPSLRDDMVQVATGAAGQVTADIINKGFVAPIIEQINALIQMDRNNQFLQDKLERT

KDLLQHISIAFENQQAAPHKGIACCLAKMRDVIQEGKDLSVQYKEKQKSPFWLLYKVGIS

TQITELNAKFDRLLQDLQNDFSIFANAQRFISALPPSDALLQPVPDEGFIGSGFEFAETQ

LRTWITAEAPQVRRIGVYGMPGVGKTSLLKRIYNFYKVSKAFDVVIWLTVSRVYEIRDLQ

GRIAEQLKLDLSHVSVLDIDTPKMRLSERLSKMKYLLVLDDIWNALDQQALGVGFGTDKG

SRVVFSTRSRELTLTEAEESIKVEPLSRDEGWELFQRVAFKYGNESGHVPEELKQCARKI

ADECHGLPLAITVVAAVMRGKGAVVDDWNDCLSLMEKADPSFTETHPRVDEELYRKLRLS

YDDLLKTPNLQNCFLYCALYPEDEQISVDELVRMWIAEGFFNSCGATYSMNMKTGHRYVK

LLIDRCLFQSVNFQKQSNKPVKIWKKRFITVHDVIRDMAIHIGEKEENYLCRAGSQLKDF

SQRQTQDCKRMSLHLNQIDSLPEDFLSRELVSLIISKNPLAYDVRGSFLMNLNSLRILDL

SNTQIESVPTSLTQLRVLDLSNTQIESVPTFL

>LL_022512_T.1

MANLTTAASSSMASSAPSTSYTRYDVFISHRGPDVKNTFASHLYRRIFNSGLSVFLDREE

LEEGHNISSQIKQAIHGASLHIAILSLNYAQSSWCLDELVLMKDSGTTILPVFFKVDPSV

VRWTDKGGAYAEALQGLQQKTTPDSETGEAKPRYDSNTIQNWRNALSYVADLSGFVHGDQ

DDEGELLDNLMESVFKKVPRPPLDVAKYPTGLDLKFEEFEKTVLSQKQDEKPVEAKVVGI

VGVGGVGKTTLAKEFFNNRRSDYNSSSILFDVRENARTISDLQYKLLIDLKPEIKEIKIK

SRDEGIEILKRHLSSCQALIILDDVDNMDQLQAFFPIKKVLRPDSLILVTSRDQHVLRIS

GVLESSIYTL

>LL_022573_T.1

MGTAHLAATGLLVISNVLQRFEDVSKNRDECLRLLKEMFFLAKVVKQIKDHPQLKLKMRD

EVQDATELILDGCIRCYSQMTSSKISGFFSTTVNKEELAKLKQELDYIYKHLCLRLGIEI

YDANQCKTGSLSRLYPQHAVGIEEATREVIDILEWGSEQNAHAVVLHAFGGMGKSTLAQA

VFSKLEIKECKYSKIELFKDINSSPNILELQKQILKDLMVKELPEMRNHQDGQLELARTL

ENVPAFIYIDNALGVNELQQLLPEDMSRAKKVRLLITARDKNVRKACPLKTPPKLYAVGC

MSSTESTSLLKNEMFDDPARILDCKQVNYIVDKCGGIPLMLKLVARALRVAKDKPQEVGE

VMKELENLKGEDFGANKIEPYFFVYDKLPEECKDPLLDICSFFHGWDRDTVADIVGDSEL

KMLESRALVTQDTNGDISVHDIILALGCEKSKGERYRFSNASQVKKLLDEMEDKDIQAIK

GIWLTSDYKNTLDISASKLDLMYDSLRVLAFESLTRVKGKCTNIFKKLIFFKAEITHLPF

DVSKLKGGLKYLNYQPKDLKLSEIPSSLRHVEFDGILHNPGFQISSTDIQQLKNLRILKL

INFAMLINLPENMGDLVNHLEELCLSGCRSIEQLPKSISNLRSLRILRLERCYSLKELPT

DLGLLFSLQELHLRYCGSLKELPNSIVNISSLKILDLYHCALLSLPPGLGNLTGLVKIDL

TGCYRLESIPESIRELKLLQPTLEMSFCQRIKELPHGLCNLTYISHISLGHCIRLENLPD

RFGELASLRTLNLGQCRTLKKLPESFGQLKCLEELVLENCCHLRELCNDFHCLLSLQILD

LSGCFKLQKLPADFHSLKSLQRLNIGSCHKLGGKWMDHVLKIKSLQAVDIERSQQLVNRW

TEIQKEGQQSWSFAVSAGQDLSEDQKETIEKTLLSDFDRLLTNGRGEPFCLSDLPPNTLF

LFVCDPYPYFRYWRLIEETVDEIKKQLKIIYIGPHFSELPVKVKEGIFMRDMSVTRVDDV

LKKVSLISDTDEGVRANGSLCKCFRMHTTMGDGEGDKYVKSWKLLTDSEVEEYVVSNCRR

YIRLKKLAETSQESNMELLRELFTPGKKEITNVFQRYHSEQDGTEDSEPFICGVDKLEGK

NIILEMRPVHLCNDIRLSSLEEMYLMEKERLELEIISIPIVEPRTQMDPIVEPRTQMDLF

DVHTQLSRKVPWLVLQNSWDKITRVVKYFLLKQCGWEGGDELEKCYTSMIWIIEFNGKIT

IYKPSLFHKLDKWKILFNYLESLAYQVKWGTKVRGGEKIEELRREEWKQLTSMSTLEFIF

NHLGSVSNQVKEKMFQGKMICLLYNGAHRRDNEKVTTFLLNSMTEVRDNIHIIYISGNME

RFRLTDTITKDCYNSSGKTVKMDGMSLLNVLGDEAVKFWMRLLQWKDEINENDYYADHKQ

FCEMRRLLLSFALDDEQMDEQIGCMIFMHEDGNMFATCRNAILRRLFRFNNKGEDKLKGW

MQLLVKVLMEEKKERHHESSSFCNVTNSIQTAATAVKPETVDLDLDLDSLIQYTSDLLEQ

EELYQLLEFNKEGKRSSRPKARHHWPTSEGFSMLKNFLSEGISATWVEDIVSGHPEEDKW

KSVTKRLLKELGEAQWAAAGLLIVAEVLERFDTVSTNRNECLFLLYQLNRLAIYVKQFKE

RSGSWTVMMREATGSIVVGSMMCCAQIDPSKFGMFVENSMNHGYMNSFDWELKRKCNIMD

GSITKGVGSERGSHLEIMCNDVFFDFVTGDLANKNENIGGPSAEDENATHGNPDAQPHIA

PSVAGSSS

>LL_022607_T.1

MADILTGVAGNTISSLAIAPLIRLLDEVIHLDENRQLLQDKLSSTRILLQDISNQFEHEE

RSPPGTVTYWSQRLQQSLEEAKSVIDRSQKQNWCLDCLICKPRVSRQIRDWTTNFDELLS

NLQRGFQLFRDAQQIALSTPQRPETLLQPVPDSGFVGLGIRDAEKRVQTWLTMEDHQFRV

IGIYGLGGIGKTALLKTVYNTHRTGNVFQAVLWITVSQSFNILNMQNDIAEAININLGGS

SNAEMRKMKLFTSLKAKKFLLILDDMWSPLDLDEEVGIPVGNDKGSRVIISTRSRDVIRR

MGTNDFSIQIKPLSEEEGWELFCRKAFKDDGRAPTKSIEDVARLIAGECKGLPLAINVVA

AAMIGNSTVDEWDRALSHMKSVDPVFLNTSGIEEDLYRKLKWSYDRLPDRNFQNCFLYCG

AFPEDAEILVETLVEMWAGEGLLQSRETSYLIDTGREYVKLLVDRCLFEDVFGDMRLIKA

HDIVRDMAIYIAEKEENCFFRTGQHLQRFPVENGMDSGKRIAMGYNAIPFLPTDLTCPRL

LALLLPRNQSLTEVPNGFFLSLTSLRVLDMSGTKIESLPGSMWHLTQLEYLALCKTPIKN

LPGEICNLGRLQFLHLSGCSGLKSLPSTIGELKTLKHLDLEGCWMLTEIPREISQLTSLT

KLNLWHSILNTEEGSSGVCSLKDLRNMPNLMELYVRVKAGSEVEAGVKSGIKVGTMGTWV

EMRRLTLDFHVDGLDDDDVVEDLPEDMHKMKKLQSFYLSRYYGRSLPNCICDINQLEYLN

LIGCWQLRELPLLDRLPNLKSLWLQNCRQLR

>LL_022608_T.1

MALSATQRPEALLQPVPDSGFVGLGIKDAEKMVQTWLNTEDHQFRVIGIYGLGGIGKTAL

LKTVYNTYKTGNVFQAVLWITISQTPNILNMQKVIAGPGNLDLHAEGISDSEMLKMRIST

SMKNMKFLVVLDDMWKSLDLEEVGIPVGNDKGSRVIISTRSRDVIRRMGTNDFSIQIKPL

SEEEGWELFCRKAFKDDGRAPTKSIEDLARLIAGECKGLPLAINVVAAAMIGNSTVDEWD

RALSHMKSVDPVFVNTSSIEEDLYRKLKWSYDRLPDRNFQNCFLYCGAFPEDANILVETL

VEMWAGEGLLQSRETSYLIDTGREYVKLLVDRCLFEYVYSDMSCIKAHDIVRDMAIYIAE

KEENCFFRTGQHLQRFPVENGMDSGKRIAMGYNAIPFLPTDLTCPRLLALLLPENQRLTE

VPNGFFLNLTSLRVLDMSSTKIESLPGSMWHLTQLEYLALSSTPIKNLAREICNLGRLQF

LHLFSCKELESLPSTIGELKTLKHLDLEFCSRLTEIPREISQLTSLNRLNLRNSSTLKME

EDSSGVCSLKDLRNMPNLMELEISVKGGSEVDAGIKSGIKVGTMGTWVEMRHLSLQFHVD

HKDDVVEDLPEDMHNMKKLQSFDLWDYCGRSLPNCICDFNQLEYLELIGCSQLRELPLLE

RLPNLKSLWLQSCRQLRELGIGRWGSAGVGFPMLERLRLDNLPRLESMRSSSSNIVWSEG

LMPKLQLLRIRGCPSLKRVPEEVAKLPNLREIDAREAAWWEGLIWEDDDVKKTFEDKFRK

PRLKKKFRKPVL

>LL_022635_T.1

MNEEDVKRWLSQRMQTKRFVLFLDHVWGEGGKLSVELGFSRLTEHSNSKIIVSSRNRRAR

IEMGVVDTSTFTMGDLNEDKRRAEIECDKCGGLPLAIKVTGRAMESITYAHEWELAILRL

SNANSQDHQALHDHLRWSYDALGSYDVNLQVCFLYLAAFTEDESIEVDQEEWITFWVGEV

LLKRNERGLEKGCLKLMSQPTTHLRWEKFMSIF

>LL_022649_T.1

MLLPMKDMLSPESLVLVTTRDKHLLKQWGVLESSIYNMTGLNMPHSKELFCSYAFFKRFP

PPEFAELVDQFVTACGGLPLSLKVIGALLCGENDELYWNEQFDRLQQSLPEEIQKTLRIS

YDSLVKEDQEVFLDIACFFLGEYRDKAIRIWGLLGIRNLENKCLLEVDSESKIRMHDHIR

DCARNIAEEGSMPRRLWHPTTNIIDDFLEQSPSIITEVRGITTVSSSDGFFRGNKGHQML

HYISEMPQKKKKTHIHGMKNLQFVATEDGYLEGIVRGMRSPHLTWLRWYGCSDSSLPDWI

PMKNLKVLEVQGRKFNSLWPPQSQGPLQLRELNIEAPLSEFPKSIGQLKYLESISINAIH

DFSYSNSPIQIQVETLPQEFCQLCSLKYLVLKGFSRLMSLPFSFGKLTNLQYIDLDGASR

LQMLPNSFGDLSRLKHLSLEECSSLTISNGTLGNITTLEYLNLRGCRNVKELPPQVAHQR

SLEKLYLRHTNLKELPSGLGDLCNLEVLEIGDNSSELLPSSVGYLKSLKYFSIYGWPKLK

CLPDSFGPLTQLTELRLVRCGIEFLP

>LL_022693_T.1

MANLQISSVDTPSTSGHYTGCYFKVFINHRGPDLKTPFAIPLYHLLHSGGLQTFLDKEEL

EKGGLLSPQIEGAIRTASIHITVFSPGYADSTWCLDELLLIWKKFKSGDTIIPVFYDVKP

SELRWTTGRYGEALSNHEQKGRYDPKIVKKWRKALSRVSHIIGFELEDYNGDKEKLLDAV

VKCVLKKGRKIGLDVAMHPTGLEEKVEEFEKTVRLQERQSGKVKILGIVGMGGSGKTTLA

KYFFNEKSSNFGSNFGKSCFLFDVRENAARGSLNSLQTQLIKDLAHRDVQINHVDQGIEM

LRTELRTNLGSSQQALVVIDDVDHAEQLHAFLPIKDVLPSDSLILVTSRYKDVLTRSGIA

ESSIYKLTGLNEQYSQQLFCLHAFCQPHPLSGFEDLVHEFCRACNGLPLSLKVFGGLLCE

KSDKSYWKGLLNDLILRGKLHNDILQTLKLSYDALGVEEKQIFLDIACYFIGENMDMVIR

ILDGSGWSGLVGLQNLQVKCLVEVDGGNTLKMHDHLRDMGREIADQEKSSGVIPHRLWPL

IQDIPDLWQRSSVITEVRGIRTVPCFVHVFWKSKEEWSIWLKIWCDEVFGNCLRKLNDPR

PYCFSNLQLVATKDDHLKSILRRVMSPNLIWLRWNDCPYTCLPSWITMKNTRVLEVSGNK

LKTLWRSESQAPLELRELTISSPRFLKFPKSIGQLKHIEKIVVKRSKLKSLPEEFCQLRL

LKYLDLSQSYSLQMLPKSFGYLANLQHVD

>LL_022707_T.1

MNALIVVVDYMHTSFTPYIACDCLGLNTFCFTSFSFATMAMEAIAIGAAANLTSSAIIEL

LTYVKNATESEKNLQFLTTDLTRMEGILLDIRSQFEDQQKMLPPLLQNSLESMKSALQEV

SDLIDGAKRQRQRCLGLGWCLFCKPKLVTQITEWKTNRCNQLLQQLDSDLSRAADIAQLL

ESADTQTDVLLQPIPDCGFVGDGIRAAQTKLQIWLNEPNRVIGVYGMGGVGKTSLLQLIH

NDCKEKVSTNFDFVIWNTVSGSQTSKIPLPNEEFDRLKIESLQDTIAESVGLDIKGISID

TRKMKLYASLKNKRFLLILDDVWRPIDLDQVGVNFGHDNSSRVLISCRNKFVVETMAANY

QYRVMEIKPLSTKDGWDLFRRKAFRNGPAPDNRVEAIAREIAEECQGLPLALNTVAAAMA

PKTGAVEWRRGLDFMRNVHPSLSSSHPTIHDQLYQRLKWSYNDLSSDLKMCFLYCAAFPE

DELIPVETLVEMWTAESLVSQKGTSYYMDVGREYIDALVDRCLFQYDYMYGDKKAFIRVH

DVLRDMAIYVGEGEEKGLYLSSQHLQHFPTEEETRDRKRISLLNNQISYLPTTDLRCQTL

VSLVLAKNEELKQIPDRFFENLISLKVLDLSETAIEALPASVGQLGQLQCLHLERCTELK

ELPDRIGELKHLQFLNLSYCWSLKYLPDRIGELKHLKHLNLGGTRLLRANQICQLTSLNR

LVLTEVLTPMSLEDLTNLSNLIVLRTKIKPEIKGGSMSSWSEMRELSLIFSEDDVEDVAA

LDILPQNMQSMKKLQSEDDVDDVEDDAALDILPQNMQKLERLVLGNYRGVSLPNCISNFQ

NLKELELFSCRKLKELPGLEIGSDAASDGFPMLERLSLDGLDSLESIVWNEKTMLKLQHL

SIEGCRVLKSFRTEELPNLRELFIGHCDELSNVSQISSLPMLERLRLWYLDSLESIVWNE

NTMLKLKHLTIHRCPVLKSFRTEELPNLRELWIGFCGELSNVSQIGSLPMLERLNLSYIE

KLESIAHWNDKTMPNLQNLRVNECPMLKRLPKGLDKLSNLRKIEGKLDWWNGIVWENHHT

VYSKVSQFFRE

>LL_022728_T.1

MNEEDVKRWLSQRMQAKRFVLFLDHVWGEGGELSIELALSRLTEHRNSKITVSSRNRRAR

IEMGVVDTSTFTMGDLSEDKRRAEIECDKCGGLPLAIKVTGRAMECITNALEWELAILRL

PNANSQDHQALHDHLMWNYDALGTYDVNLQLCFLYLAAFTEDESIEVEQELITFWVGEVL

LERNVLGLEKGCLKLMNQAT

>LL_022750_T.1

MAGEAIATGAAANVLSSVFMKFVAEVKMVISLPQAVRDMQTEVQRLNPVISEIQGELNQE

QRNPKQVVKNWLDEADEVKKGSQSITNEYEQRKNCVGCCPNYLCRYRIGKDIRNWKQRVA

KLDSEKRSNLPPSGEYGDFTPPTQVPIEIPKSGFTGNAIRSAQSRLEAWLTADSSNTGIM

GVYGMGGVGKTSLLHTINNSHKVSQSFDLIIWVTVSREYKISDLQDCIADRLKLKNFPDK

SNRQGREVMLCSYLKKKKFLLILDDMWESLELQSLGVSGNERGSKIILTTRNKAVCTRMD

VEEMIPVDPLLEDEGWQLFCSRAFRNGDVPPAIEEVAREIAGECKGLPLAIIVVAAAIKH

YEYPHEWELALHQMRTIDESFYDIHDEIEKKLFQRLKWSYDVIPKYLKTCFVYFAAYNED

RIINCEEVIEMWIAEGLEGLVKSGGQSYLHDTAYSFISFLRDRCLIEATKTDIVGRIEQV

KIHDVLRDLATLLAQKEHKCFFKA

>LL_022778_T.1

MIMAAEIIEKMEEISELQKDGSVLNTDFSRMKGLLIHIRNVFQSRQKRVPEALERSLEKM

KDALKEAKDLIDEDKKQRERQCRFLFGNSKHSSKIRVWKAGFYALLQELEMAFSTSSNTG

QIAFSATPPAHRRLQPVPDSGFVGSGIQFAREQLQTWLQAEPHGQVRVIGVHGTRGVGKT

SLLKIIYNTYKQEVSAFFDVIIWITASPGNQIEQLQASIAKALNLNLEGSSNADARKTQL

NASLRRRRFLLILDNMDQQIDLNEVGVKTGDDTGSKILISGRDKNVVEGMGSRDYSRIIE

PLSPEEAWELFRRGAPFTNGVAPALERTAKEIASTCGGIPLAINAVAAAMSEDYDQWSSA

LDSIRELKSSLPRDLETIDPDLYPVSRWCYDKLSDSKLKICFLHCAAFLPDKEIAVETLL

EMWEAEGLLKVQDARRRYIDELVSRCLVERVEYIVDDNYHSVEYLKIHGALRGMAIYIGQ

KEEKCLFVAGQQSQNFPNERDTRDCKRISVGHTAIKDLPSDLKCSNLESLILASNIRLEE

VPKGFLSKSINSLKVLDLSRTSIKTLPTSVDQLEQLVFLNLTECIKLVDLPDSLCKLSSL

QVLNLNSCSALLSLPSNIAELKNLKHLRLDACYSLNGIPQNLSQLTSLNTLILPLMESIC

ALSVVDLSKLSNLMELTTVVKSKDLSKQNEVGTKATWLQMRNLTLEYDDDAGADRDTIAR

EHVILKNMGDMESLQNFHLIKYQGETLPNSVCQFQNMKMLLLHSCYQLKAFLAGDNSSSA

STGDSFPKLEKLVLRNLLKLESLARPLWKEGTMSELQLLVISNCPLLKELPSGMKLPHLK

ELHITMCNEVQKLDIGSGGFPMLETVILDELNMLESISDPSTVWTDSTMSKLQMLEFIDC

PLLKKLPTGMETLSGLRQINGELDWWQSIIWENDDKKTKLSQLFMII

>LL_022795_T.1

MAGVSTGTGFAANVGSNVISDVWKEIFQVVQMVRALPTDVGNMNEEVQRLENLRDEINTE

LAREQRNAKIIVQNWLTKAGRAIDSSVSINTQYDQQKNCLGCWPNYLCRYRISKDITAWN

LTVNQLHSERQADFPPRGEFGDPTPRTHISIHHPEVAGNGIQAAQLELERWLTHKDSNIQ

RIGIHGMAGVGKTLLLDIINNSQIVRNSFQLILKATVSRNKMLDLQDCIARRLKLREFPD

KSNSEERVEMLRSSLEAKQNYLVLLDDIWGDSALWTIDDLQNLGVSLNNTGNKIVLTTRD

KDVCARMNVQEIRTVPLLSEEDGWQLFRSRAFQNRNGDVPEDIEEFARQIAKECKGLPLA

ITVIAAAMTKHTDLHDWEVALGQLRNLPAMFYDLHPGVDERLLQRLKWSYDALTLPDLKT

CFLYFAAYPEDAIISCTEVIDTWIADSLVKGNEDSYLQDTGRTYINQLRDRCLIEVAKKD

DRGRISSVRMHDVLRDLAILIAEKEHSCYFKAGQGVTRFPVAEDADRMVCARLSLMYNRL

DSLPTTFACSSLSVLLLGQNPRIKEISGSFLSELPSLKVLDLSYTGITSLPSSIGDLKHL

ASLQLGHTQIRELPETVGDLNNLQFLGVQYCKQLLCLPERISELKRL

>LL_022830_T.1

MERQESIPEIRTPEDGQRALGDALEKVTAFIYIDNVLGENELRQLLPEDMKKAKKVRLLL

TARDINVRRGCPVTVKSYRMRSMSSGEAMDLLKKGMPNGMAGKLNSGQLRRLIDICGGVP

LMLTLVVGVIRSEDDKQKAYRIVMQDRQKLKGQTFGDKEHYLFAYDSLPETCKDPFLDIC

SFFNGWDWNTVADIVGESELDTLEKRALVTKGIDGVASVHDVILTIGLQKTEGERFKLTS

AREVEELLCHNNEEKCIQAIKGLWLTENNDPLRMDTPKLDLMHSSLRVLALGNSIRVEGK

CKKVFEKLVFFQAGIPHPPFDMSKAKELKYLSYGPETLDLSKIPSNLKHIEFHGGLHSHA

SEISSRGLRQLNNLRTLRVISFANLRKLPTELGDLVKGLEELSLSDCKSLEELPKSISRL

QYLRVLRMDHCSGLKQLPEEFSKLQRLRVLRMDYCSSLEHLTEDFGLLSSLQELNLQGCI

ALQALPTSFETLSSLKLMNLSSCTSLEELPHGLDNLTSLVSLELKGCNKLRSIPESIGQL

KLLPSSMDISDCSSLTELPDEFCNLSFITHLLLPNCKSLRRLPDRFGELASLKVLNLRNC

KSLKKLPEGFRLLQFLEVLDLGGCNKLEELCNDFHCLSSLQDLSLSGCQCLKKLPENFHV

LVSLQRLLLCNCKSLKQLPEDFHLLASLQHLHLHDCPMLEAKCMDNVIKAKTLQAVDIKR

STMLLERWAEIQREGDRSWSFTVFTGQDFSQEEAIKIQKKFLSSLDGLLTYGQGEPFHLC

DCPPNTSILVMFDDRPDFKYDYPWPLIEDTLNDIQIKFEMIYIGKYFDKLPTKVANKILG

HATYNAEVVLLFKSWFETLNSDAFEDEHFQLSAKIMGNGKDDKYLSPGKLIAKCEIEEFV

LGKCGKYIRLKQLETSQQSDKEILREMFATGEESTDHIFQSISTSHKIQTENSKPFTCGM

DELKGKTVLLEIRHIEVCYDIRLESLTQMYNTAKERLGFEIISIPIVQPSTKFDVFDIFQ

RFSMEVPWLVLQKPWTIAATVKYFLCKQFSDEEEDEEEDEELKTWYPSVIWIIEPNGRIT

TYKPFVFSMLDRWGEKSYPFTDEKIEELRKEEWEKQKNISSLEFLFLHLEEVSMQVKDML

LQGKKICLYGGNEG

>LL_022836_T.1

MASASTSHGNYDIFLNHRGPDAKKTLASHIYHGLTQRGLRVFFDTEEMQPGEELSAQIID

AIGTASVHVAIFSKDYASSPWCLKELVEMVDTKKTIIPIFYDVNPSELRWTQAGDGRYAQ

ALHMLKSKKTLDPQTNKETPRYNSTTIENWRKALSYAAEISGFELNKFNRDEGLLVNEVI

QRVLKTVPKTLLNVAMYPTGLDVKVKELEAIVSSQQQSGSLARAVGIVGLGGVGKTTLAK

QFFNSHTSNFERSCFLSDVREKAARNSLISLQAQLLKEL

>LL_022892_T.1

MKTAANLYPMKGMSSTDAMSLLQKEMFDDPERILDSKQVDHTIKKCGGIPLMLKLVAGAV

RVAKDKPQEVGEVMEELENLKGEDFGPGKIESYFFAYDKLPEDCKDPFLDICSFFHGWDR

GTVADIMGESELKMLESRALVTQDTNGVVSVHDIILALGCRNSQELRFRFTNAGQVQELL

DHKEEKDIQAIKGIWLSKNKNPLDISASKLNLMCNSLRVLALGSLTQVKGECSKIFEKLV

FFQAEITHLPFDVSKLKGLKYLNFKPKDLKLSEMPCSLRHMELDGSLHSDALQVTSTDLQ

QFGDLRKLRLIIFEKFKILPKEMGYQVNVLRELSLSGCTSIEQLPESVSKLRSLQILRMD

MCVSLTELPADFGSLNSLQELHLQLCFNLKALPNSVEKLSSLKLLNLSFCHNLLNLPPGL

GNLACLVKLNFNQCLKLESIPESIRELKLLQ

>LL_022907_T.1

MSSLPLASGGNPILHTLDVFNCKKSSSRLDELLQGLIQTVQPLCLSNLKAPKNEQNSIYE

QFLCILMDGVELVKNCEKTSLYNVYQNLRYASKIHQLEKEICHFVHYQMPVQLLQDVKNV

ITELKSLRHLYESRSVDERKVNETIVPKLTEDPFQNATMLQRMGPDGVFDGAFGEAPPCR

YDDSIKDDFVVGLEKNIWDLKKILLQKEVSVVGVQGMGGVGKTTVALALSSDRDIKGAFR

NNIIFITVS

>LL_022945_T.1

MLLSASLKSKKFQFCLVLDNLWSPLNLQELGVKFGHNKYSKIVFSTWKKDLIPAMRVEKY

IEIQSLSQDEAWMLLISEYRFQ

>LL_022982_T.1

MPPPLVDPITGAIIEQALSKLLDVVKIIASSKIRGKELKEKLESLKPIIDQICQKLSDPD

RANQFKDFQVVLQDGLVLVEKLEKIHSFDIIKKYQYGKKIRKFQKKVSEFLHTHGPPSLI

LDLQKLDADFNGFCQRFEGLIESLIQNVIARMTNDPISNTIRLQQMNNTTQVFPTSMEGM

NESTAAQQAISCTYQVPDMPNFVVGLDKPIDAVKKILLQNGVNIVGITGMGGSGKTTLAS

AVCNDTQVRDRFKTNIFFIPVSQLERNANGLLDILEVMWDKIIGGHRPHFQSIEDAHNQL

LTNLRLTLCQPTLVVLDDVWPKPNVEQLLFEVKGYKTIITTREESNIFITGTSRQLYKIP

VLKDADALSLFCFWAFSQKSIPATEKEDLVK

>LL_022983_T.1

MFQGQQNITPQMQAAIQVVSVRMAIFPSSYAEWERCLDELVLMVKCLVDATATTPPIFYE

VEPSTTYDPQTSEEKPRYDSDTIRGLRNALSYAARICRFELDGDEEELLSWVVQSVLKNV

SKPPFSVAK

>LL_022985_T.1

MAASTAASSNTNSDYNYQVFLNHRGEDCKKTLASHLYRRLIRHGFRVFLDKPEMKVGDDI

EPQIREAIATSSIHIAIFSRGYAESKWCLKELDLMLKSKVARATTIIPVFYDVSTSLSEV

YGQALCNLENEPNIQVWKEALSRVSKISGLELKAYNG

>LL_023110_T.1

MVQHLSEMIGMDERRKDFEMIGFDEKVGLDKMVEDFERNFLVNHRQGANPLVVRIVGVGG

VGKTTLAKELFRRKRSDYNKSCFLSDVREYARRGLLTSLQSQLLKSLNPSVEIDTIDDLR

GRQELIRRHISDFSVLLILDDVGDLNQIDALLPVRTDDLFRNSLIVITSRPSSPDEGRVI

TRSGVENSAIYELNGLNIQHSQELFCAHCFSQPSPPKSFECWVDKFFDACAGLPLCLKVI

GAHLYGDFKPHHWEEIYDKLIFNPDKIEESIKISYESLTMEQQYIFLDIACFFIGQKRNT

AIIIWNGSGWNGLLGFQNLHNKCLVEVDNENNIHMHDRLRDFGRAQAATLSPSRLLNCMN

ILEQLFVSAQSLLY

>LL_023131_T.1

MMVDCMSKSKSTIIPVFYDVEPSELRRTRDNNGVYGQALFKHEEEKKFNSSTIEEWRNAL

FEASKIQGFDRETYYRGACTGVLQCLERIFELCNLPLVFRAKAFMGDIVEGVLKNVERRL

HVSEYPKGLEQKLKDFETRVSLQQESGKVRVIGIAGLGGVGKTTLAKEFFNRQRSKYKRS

CFLFDIRENSLTTLQSILLKDLTQSSVQIRSPEEGKGMLRRYLRSSHALIIFDDVDNIQQ

LDALLLPAKNVLNPGSLILVTSRNKDVLISSDIPETSIYGLTGLNPLHSEELFCSHAFGQ

SHPIVGFEEVVGKFLDVCHGLPLSLKVLGALVHGENDLKHWEDQLRKIIKILP

>LL_023147_T.1

MITSPSPYLLSCPPALTIFIVAYVGRTLVLLRLPSWGVPHRLFDDSASSLAMATAIELGR

SRYKKSEIYDVFLSFKGRDVRRGFADHLYHALTDSGFRVFLNIHHLGQSGEDIFASLQHV

IDEVSRMLIPIFSPN

>LL_023230_T.1

MLKEHLSSQQILLILDDVDKVDQVDALVPDQTSIHSNSLILITSRDRDVLTKSGVEKSSI

YELNGLNKQHSVELFCWHAFRRSYPLPEFEDLSEKFVNACNGLPLSLKVLGALHYGNTDM

SFWHQQLNKLVKILPGEIHERLRISYETLDSEEKQMFLDVACFFLGENRDTAIRIWNGSD

WAGELGFRTLQNKCLVEVVEGNRGSEIRMHDHLRDLGRAEARVSQPLRFWRFDDLLQQSS

VSINHSIF

>LL_023245_T.1

MANLTTASHHGDNASSAATSSSMASASTFCSDFDVFINHRGPDVKHSFASHLYRRLLDRG

LRVFLDKPEMEAGQNIRAQIEDAIRGASVHVAILSPRYAESNWCLDELVLMRNSGATILP

VFYKVHPSVLRWTVEGGVYAESLRYLEQKTTHDSQTGEEKQRYAPDTIQKWRSALSYVAD

LSGFLLSGDKGDKGKILDNVVEHDEGELMDHIVKSVLKNIPKPLLDVAAYPTGLELKLED

FEKTVLSQQQEERKHEETKIVGIVGIGGAGKTTLAKKFFNTKRSHYDACSFLSDVREVAA

RRSLNYLQSDLISDLKHEDIKVGRHEGLGLFQKYLKSCHAVIILDDVDHISQLEAFLPLK

HMLNPQSLILVTSRDKHVLVSSGISEKSIYNLKGLDPRNSQMLFCSHAFHKPLPPPEFAD

LVDRFVRTSHGLPLSLKVFGALVCGENERSFWEEILEKLDKILPAEIHNRLKISYDSLSH

E

>LL_023272_T.1

MAQVSEILPVAAPYHVFINHRGPDVKETLARLINNRLTKCHGLCVFLDEKEIRIGDSLSP

AIIGAIKLASVHVAIFSRRYAESSWCLEELHLILRSYHEGKAKIVPVYFGVEPTDLRHIE

SGLYAEAFEKHQRKGRVAMEVIENWKTALNQASMISGVLFKTDESVDGDIFENIVDTVLK

EVFTKEAIEVAKYPVGLEQAAEDFQKEILKHSDPNATTIVGITGLGGIGKSTLAKHLYNL

RRPEFSRSSICEVGQMGLRSSQKKLLRDLLGIYDSRYIYSISQGKRILNDCLPRLPPVFI

VLDNVDNSDQIDSLLDVDSVASRSLILITSRDTNVLRCSSAKTIIYDVKTLHQNHARELF

CRYAFCQSKPSEELDYFVEEFLKICGGLPLCLKVLGGLFVGRCDKEYWKRQLEKLSTTLP

HEIINSLRVSYEALDPEEKEVFLDIGCFLVGEEKELAIRVLEGLHDNSRIGDYLESLRQK

CLLDFHYDVQSEDEIEIRKREYPYRSHKLTMHDQLRGLARHIAREEIANTSTLNPLRISS

SYDLRDMLKLRAHGSKAMAYISSLHIRGIRCSKDVNRPFFREDIKDIKDIREDIKDIRVE

GVRGVRLFVVEHLWCASFIFPFRQWDMYGELVWLRLRSAISNVFSVDFLRTMSLRQLRVL

EIQGSGDTIESIFQNLDHDLPHLNVLNIVYCTAPKFSYNPGRSSHQTSSGKRKLSSISQF

DPKISDKPGSLKSSHQTSSGKAKSSESSLISQSDTKKSWFFDYLKNWGTGMKTLTRLVLK

NIRFLQSLPIDFSQLQNLRHLDLSGCSELTELPNTFSELLQLQYLALRGCFNLSMPPDML

GEISTLEYVDFKGCSKLTLLPEKIPHQRHLRYLNLLGTNLIQLPDNLDNLENLEQIRIGS

PALNEIPLNVANLRGLTELFLLDCDGLLQISPEGIAAPNIKILVVDGCSIDNFSFQEQEI

LDVGSIYLRVGMRFLRDLTLKKTSIAEICFPEGVYSSLETIDLSENACLMRVNGLPSALV

SLNMQNCRELKTVTSLSKLVNLKYLNINRCCNLKTLDIMEGLASLEEFNAEGCWELQTTE

VLSRLSKLNCLNISTDNQSFWEDIREFLRSPTQMPSTAIFSGRNAIFSGRTSLDTMLRVR

HITEKFKLKMLNIVFEGQPRVYKRLEKLDAHGGILVFLITNYAKSPQYSVKFGRSSRHST

GDLEYSTMSGDRGGRRVHVFMWTEGSRLFKEYSECGEFYGSFNMKEEEANKKTVGWMIMV

DKKLDVSKFCIEFLNAFV

>LL_023312_T.1

MDSLCSDSLILITSRNIDVLTRAGVEESFIYKLKGLTPQHSQELFSLYAFRKPYPLPGFY

ELVEKFLIKCDGLPLALKVFGALLFKQKETFWEKTLSQLEVPFGIKQKLKISYDALDENE

QQIFLDIACFLIGEKRDIAIAIWDEHRFQNLESRCFVEVDSNNDIRMHDQLRDMGQEIAL

ERGLWHPAEDIDDLAKQSSVITKLRGFKMFPRCQRESEERRRDRLYNTFCPSRTMKWQFL

SSGGYLLGRILRRLHY

>LL_023413_T.1

MAELTSSVVSTSSRNNHAYHVFINYCGSDHVKKPLATDLYHRLCEHELRVFPDVEKLQEG

KDPAKDQIHQTEAAILTASVHIAILSPAYAESRACLTELLLMLKTGATTIPVFYDVKPSQ

LRGTELCAEALYWKENNERVNSELIGKWRKALSEVSNKDGFALELKSYNSDAEHLVNAIV

EKVETNIGHVFINRCSDVQKTFASHLHRRLLFCGQRVFIDKEELLERDEEDSLTPQTKDA

IRKASLHIAIFSKKYVESSCCLNELVRMVESKAIIIPVFYGVQPSDIRWQNTDNTFGRTL

SKVADIIGFEERWSDDGLLVNDIVERVLSKGKTPMSYDVFINHRGPDTKKTLASHLYHRL

SALGLSPFLDRENLEKGHELTPQLKQAIGIVSVNIAIFSGKYADSTWCMKELEFMLLSEA

AIIPVYYGVEPSDLLETSRDSERVYARALCNLEQKRTIDPATNQPTQRYDSRIIEKWRQA

LSNVAAKRGFNLNDYNGDEGVLLDKVVHEVDRIVRIKRAGQRSNSSWFRAGLEGTALSR

>LL_023458_T.1

MPGFISGFMSVGIHNAIQKIFYSTNTAVRCREELASLKDLIMKMEPIIKEIQQNRLELNR

KKDMKPSAVNAWLEQLDALLQQASKMVDRCTIPTCDIVSRYQMRIMITRLISDINEHLKL

SPLVHMAQTQEVLADALGQMRQTVEAPMDSVSTSSASTSLQSVHSSTGITARKKLIDEPL

IVGQDQAIANLHKLVIEDEDQARNIGVLGKGGSGKTLLLKTVFNSQKVRSHFSDGLLLWL

TVSQSPSVTTLRTELYTQITLQKIVDLSKNIKEEDIQIWLNETLQQSRRLALFLDDVWDR

NAAELLEDLGIQLPVTVDSKVIVSSRSRTALQKMGIGDKYTIVMEDLMEEDSWKLFAGHA

FPYNHMKPPGNMDVEKAKLVCHKCGGLPLAIKVVGKAMAGSIDPRQWEWAAQLLPEADSV

YDCLRLSYDALGKEDFYLQLCFLYVAAAFLEDKIISVYEVIQRWVGEGLLARKMLQDHNQ

LQLGYDQFEMGKIYLNLLADRCLVEPIMRGVDGQVEYFRVHDVIHDLGIRIAEEEEHFYC

RVGCNLATLNQNECSSRTRILLSDNHLTALPESWRAPQVCSLLLPGNIHLTKVPSTVIRS

MISLKFLDLSGTSVQSLPKSVGDLKQLVCLALIAMPAIKKLPRSVTNLVRLEILDVSFSA

IRALPSGLHKLTSLRYLDITCCKDLQYLPASISRLTSLQYLLMYGCTTLWRKDQHKRFKR

VASINNVATLRQLKMLSLTNNGDIISEGTLGNMVQMDSVQISLTNMESLPFDMSNMLKLR

RLVLECSHLVKLESNFCNFQNMSNLELSDCGRLEELSYLHKLKSLRQLRILRCSKLRKFP

QEFGEIGAFPLLEIFSLLGLDNLEELPKIEEGAMISLKLFTLMECPALRILPPSFLKLKT

LEKIRIYGCSMVAENLKEVKGENTMVKVMTMSIQDTIEVKNKYLQVKGHMAGWLYGEFWC

NELFLFLKGLEAF

>LL_023546_T.1

MAGVSIGTGIVTNVGSNVISDVWKEIFQVVKMVRALPTDVGNMNEEVQRLENLRDEINTE

LAREQRNAKTIVQNWLTKAGEAIDSSVSINIQYDQQENCLGCWPNYLCRYRISKDIRAWN

LTVNQLESERQTNFPPIGELGDPTPRTHISIQHPEVAGDGIQAAQLELESWLTDKDSNIQ

RIGIHGMAGVGKTLLLDIINNSQIVRNSFQLILKVTVSRNHILDLQDCIARRLNLREFPD

IKSNFEERIEMLRSSLEAKQNYLVLLDDIWGNSALWTIDDLQKLGVSLNNTGNKIVLTTR

DKDVCARMNVQEIRTVPLLSEEEGWQLFRSRAFQNRNGDVPEDIEEFARQIAKECKGLPL

AITVIAAAMTKHTDLVRDWEDALGQLRNLPEVFYGLHDGVDERLFKRLKWSYDALTLPDL

KTCFLYFAAYPEDAIISCTEVIDMWIADSLLKGNEYSFLHDTGRTYINQLRDRCLIEVAE

KDYGGRISYVKMHDVLRDLAILIAEKEHSCYFKAGQGVTRFPVAEVADRMACARLSLMYN

PLDSLPTTFACSSLSVLLLGQNPRIKEISGSFLRELPSLKVLDLSFTGITYLPSSIGNLK

HLASLQLHDTEICELPETVGDLNNLQFLGVQSCRKLLCLPERISELKRLRALDIEECGRL

SQLPRGIAELVSLERLSMQFSVPLDFDDGNAERKHACLKDMQSLGCLRQLKVSMNSPVQE

GVIGNWSKMRLLSLKFIDRVNQDYLPQDMQAMKDLEFLALDGCNVKRLPSWVSEFRRLEC

LTLYECQQLEELELGMGFPKLQQLTLNRLESLQCVRAVAAASGSGGLGDGASSLPMLKSL

YEIRCTELKTLCGEWDKLKCLEKIVGEKEWWDALQWEDPSMKASLESKFRYWS

>LL_023585_T.1

MASPLPCICISMTNLQISSVDTPSTSGDYTGCYYQVFINHLKNLKTPFPIPLYHLLHSGG

LKTFLDKEELEKGGLLSPQIEGAIRTASIHITIFSPGYADSTWCLDELFLIWNKFKSYGD

TIIPVFYDVKPSELRLTKGGGYAQALSTHEQSGTYNPQTLQKLRIALDDVSRIVGFELEA

YNGDKEKLLDAVVKCVLKKGRKIGLDVAMCPTSFEEKIEEFEKTLLLQERQSGKVKILGI

VGMGGSGKTTLAKYFFNQKSSNFGNSCFLSVRHNGKTLNLLQHKLIKDLAHRHVQVDHVD

QGIELLRTLLRYSQHALVVIDDVDHADQLDAFLPIKDVLPSDSLILVTCRDKDVLRRSGI

EESSIYKLTGLNEQYSQQLFCLHAFCQPHPPSGFKDLVHEFCRACNGLPLYLTVFGGLLC

EKSHKSHWNGLLNDLRRAKLQNEILQTLKLSYDVLGVEEKQIFLDIACYFIGENMDMAIR

IWDGSGWSGLMALQNLQAKFLVQVDGKNTLKMNDHLRDMGREIADQEKSSGVIPLRLWPL

IEDIPDLCQRSSVITEVRGISTVPRFVDYYKSKSEEEWSMWLKNWCDEVFGNCLRKLNDP

TPYCFSNLQLVATKDGHLKSILRRVKSPNLIWIRWNSCPYTCLPSWITMKNTRVLEVSGN

KLKTLWRSESQAPLELRELTISSPLFKFPKSIGQLKHIEKIVVTRSYLKSLPEEFCQLRL

LKYLDLSQSYRLQMLPKSFGYLANLQHVDLSESYSLHMLPDTFGNLIHLKHLNLRFCFSL

TLSKEAFANITTLEYLNIDYCSNVGALPPEVAHLTSLEYLCVC

>LL_023628_T.1

MEIGTAVATNAIDKVIVDPLLQQIDDVIHLVQNRGLLQAKVERMELLLQDTRNVFENQQR

TLPASIQHCLDSTRDAIREAKSLIDRSQLKQQSLGCLFCKPRVSRHIRKCNSSFHQNLTE

LQTLFSTFASAHQMGLTSEQPPAKELLQPLPEWGLVGVDIKSAEKQLQTWLAEGPHLRKI

GVFGMAGVGKTTLLKWVYNFYKVGDLFDVVVWVTVSQDYKIWDLQHRIAKALNLDLSGIS

DFDTDALKMMLSASLIKKKFLLVLDDIWSALDLPQKLGVTFGDDKDSKVVFSTRDKNLAI

KEANKYLKVEPLSNQEGRTLFNRVAFKDDHVPEELKEFARDFADECEGLPLAITVVAAAM

CGETSVDEWNTSLSLMKNADPSFPETHPRVDQELYRRLRWSYDHLPNANLKKCFLYCAMF

PEDKRIHVDFMILMWIAEGFVKSKEATHSKDMDLGHRYVTILIDRGLFQSFNFEKQSKDI

IVNKPVNIWKERYITVHDVLRDMAIYIAEKEENFICSAGQQLKDFPRNPSQDCKRISLAY

NRIEYLPRDFECPKLVNLMLYGNPLTCAVPENFLINLSSLRVLDLSQSPIESVPTSLTQL

TSLEVLDLNSTGIKELPQDICSLSKLLYLDLSICKNITSLPCTVGKLPQLQYLLLKGCNN

LVLPAEISQVTSLRRLSLDIGTSEEALKDPTNVVSLKGLTILKELSLSIFLDFEAHELMG

TWFELRNLRLKYKNYVNDDLPQGMQNMKRLESFRLDRYEGVNLPNWICQFQHLQRLELFH

CSKLEDLFSLERLPNLKFLKLNICNELQGLGIGCSGKPGGFPMLEMLVLKNMPKLQSITE

TSSEGGVLKEGTLLKLRVLKIFQCPLLRKLPMGIEMLPNFIALYGSKSWWKHIIWEDSNM

EIYLQNRFKTKNFYNYFDEEEAS

>LL_023722_T.1

MYILAKHIKHLKNRPQLNNCMEDTINEGTDLLVEALMCTTQIDRSKFRMFLSTSSDRSKL

DKFKNGIATVYRHFQDQISLATNDLAFANKADLSVIKAVVMPPRKPRERPYPDHAEGIEE

QLKKVNDLLEWESEKKALAVILWGIGGMGKTTLADAVFAQHTIEGCKYALVKLSEDDTST

PNIVKLQNSILNDLTIGTEDEGHATFEGSASDGQRAISRILKKVEAFIYIDNVVTAESLR

DLLPRRDTFKKLRVLITTRNKNVRVALKMENRDYPVGLLPTAEGLKLLKKEIYPPAEGLN

VAGDSNRKLSDSQLDDLVKICGGFPLLLILIGSYIYSEEDKEKAYSIAKEDTEHWNGDSL

EGLDKRMFSYDNLPEECKDAFLDICSFFKGWEWETVANIVGQSALDMLEKRALVSRDKNV

TVSVHDVVLSIGKLKAKNTRFDLTEASTSDIEEFFKKDIKAITGLKFSDQKIQKSCKIPF

EPMSGSLRIFAHKMEWEGQCGNAFEKLLCYNGETPQFPFNWSQLKALRFLNYQPQNLDLL

QQISSKTLKHIELDGRLCSVSEISASDIQEFQQLQVLRLINFEKLENLPEDWGPHLRENL

KELTLSHSTSMKELTGSISQLKNLKILKLDNCWNLERLSKDCELLRSSLQELNLWNCLSL

EALPSSLGEALDSDSRRLFIYKGVLLDPPQAESEVLSDPPQ

>LL_023751_T.1

MPGVGKTTLLLTVYNSHKVGKDSFTDVIWVTVSERCEISDLQDQIAQEIGLDLPSIRTSA

TRKAMLCESLKNKKFLLILDDLWSALNLQDVGVEFGEGSKVVFSTRFKDLALRDADKSIQ

VETLSKEEGRDFFNRLAFQGGDIPKELEDYAREFADECNGLPLAISAMAKAMRHKTEVED

WNKFLSMMKTADRHFPNMHPTVDRELYQILRTSYKCLPRDDPNLQHCFLYCAMYPEDKNI

DVDALVRLWISEGLVKSEEETYSMDMDLGRTCVEKLIERSLFQDVSFKRRSKYNVQKPVN

VWKGRYIKVHDVIRDMAIYVGKEENFLCRAGRQLKVFPHSGTQDCERISLFRNQIEKLPD

DFKCPKLVSLILSENPLISSVPESFLFNLHSLRVLDLSHTQIESVPTSVTRLQLLEFLAL

SSTNIKELPIATCTLSRLRFLDLSSCKALSSLPLEISQLQLLEFLTLSSTNIKELPIAIC

SLSRLQFLDLSDCRVLTSLPPEMSKLTSLKRLHLSMGMEKALNDPKNVMALKGLTNLIEL

HLVLFSKFDALATIMGTWIEMRHLYLRHIEMNNVKYNLPDEMQKMNKLQSLVLDGYIGVK

FSNWICDFQQLERLELDGSDNVKELPPFEKLSNLKFLKLRNYSNLRDLRIGSSGKLDGFP

MLEMLHLSNLSKLRSIAEEGMLKEETLLKLHVLKITKCPVLRKLPQGMEKLSMLTAIYGH

RSWWENITWDDYAMKMHLLKLFKEI

>LL_023752_T.1

MDCARKTADECKGLPLAITVVAAAVRGKTVDEWNLFLSLMKIADPSFPNTHRNVDEGLYQ

ILRTSYKDLPRAHPNLQHCFLYCAMYPEDKIIDVDALVRLWIAEGLVQSREATYSMDMKL

GRSYVKDLIDRGLFQDATFERRRKKNIQKPVNVWKGD

>LL_023754_T.1

MENLATQNQDVASSSSTASSSALTSSYFQYDVFLNHRGPDVKNHFASHLYRRFLDRGLRV

FFDKEELEEGGNISSQIVQAIRGASVHIAIFSPRYAQSSWCLNELVLMNNSGATILPVFY

KVDPSVVRWTVKDGAYAEALRGLEQKTIHNSETGEENPRYGSDTVQNWRNALSHVADLSG

FVLHGDEDDEGELLDKVVQRVLNEVPKTPLDVAKYATGLAEKLQDFEKTVLFQQNETERV

EAKVVGIVGVGGVGKTTLAKELFNSIRSSYNATSFISEVREVAGRMSLNYLQSNLIRDLK

HIDITVGRHEGIGILKKHLSLCHALVILDDVDHVSQLEAFLPIKDILSPHSLILVTSRDK

HVLISSGISKQSIYNLKGLDTPDSQKLFCSHAFHKPLPPPEFADLVDRFVTACDGLPLSL

KVFGAQVCGESERSVWQEILDELDKILPTTEIYDTLKISYDSLRPNVKEIFLDIACFFLG

EDGDKAMRIWGKAGVRNLQNRCLVEVDSENLIKMHDHIRDMGRMIAEEGSMPRRLWHWTA

NIIHDLSQQPSQLMTEVRGISMVSSSFDFFHYQHSDHSDSTSSQTSQRNLESRYLFHNFY

F

>LL_023784_T.1

MAEAIVSTLSEQLTSMLAHKIFQEVSLVTNFREDVESICDELYSIKILLNGVMVMTNSHL

TTNWLHKVQDFLYRAVDIVEECEARKFSNPLFRYRMGRRIKTLKTCISKIHSSAKYLKYL

KSVFDVNAHVHALNANSEDKRKKSSADLIEISIVGMEAKIKDITGWILQPGFTVIAVVGS

GGLGKTLVVQKVLDNPSVQQRFDHVVWLAVSQKYIERELLLEMGKRIKLPTSEEDLSFLE

VESLKGIIQKHLEYKRCLLVLDDVWAQDGLEDIVSQMNSGRNNKIVVTTRNKQVGISMGA

HECCEMEYLSDEKSLQLFNIHAFPDCQLQNHPPSEEIQLLSKSIVQKCGGLPLAIKTIAA

SMARVERIPNLWVSTLQRLNQAEALTSTVMPSLSLSYHSLPYHLKSCFMYCSVFPKNTAM

RSQYLVYSWIAEGFIS

>LL_023862_T.1

MCSTQIGSSKRWKCSFWSASGDKKELVDFQQNLQRMYRHMDSEMDICIYDGQMRSQKPRE

RQYPDYAVGIEEQLKEVTKLLEWESENNAVAVILHGFGGMGKTTLAQAVFARLNIEGCKY

STVKLFDDINSTPYTSDILKLQTYILKDLQMGTLGEDIRTVEEGKGKIASILQREKAVIY

VDNVLLPHPFEQLLPRDMVNAKKLRLLITTRDIDACSECTKMITKFHDVKGIPHDDAKSL

LKTGMKSQTDDMDMEDQLNSSQLDRIVEICGGIPKLLEVVAGFIRGQKNKQRAYNKVIEE

KEKNWKGKIGKDIASYVFAYQYLDEMLKDPFLDICAFFNGWDWDKVANIVGDDELEMLEQ

RALVKKDPRRCVTVHDVILAMGLQQTEGTRFTITNQSQIQNLEDEDIDIKRIKGIRLLSG

NKAPLCISASIIDFMCSSLRVLELEDNIKVEGMCEKTFTKLIYFVDKGFHLRFNVSKSKE

LKYFYGAAKDFNLFQMPSSLVHVELNGKCFNSHPFQISSQCLGQLRNLRVLTLTSFSQLT

QLLQKLVDVAEGLQYLRLTSCEHLEELPEFDSKLRCLKELEISSCNSFRKLPEDVGSLNS

LQKLTLSDCVSLEVLPSSLDKLSSLQELHVRECGRLEVLPNGLDKLLSLKVLDLSWCENL

KNLTFDLGKLTSLVQLRLRGCEKLSSIPQSISQLKLLDSTMDLSRTGLKELPDGFCNLSF

LTNLQLGWCRSLEKLPDRFGELSNLVKLSLQYCESLKELPESFGRLKTLEHLDLSECESL

EELCRDFKGLLSLRVLSLDRCTMLKTLPEDFHCLTSLEILYLRGCSMLEGKWMESVVKMK

TLQMIVNIEGSPMLEERWAEIQRQGDQSVHPIRDNFAVHTRQGLANDESKENVLKRLQAG

FDSILTNGRGEPFHASHIPLNTLLLVMFDGHEQFRDYYPLNLIEENIDDIESNVNMIYFG

NHFNKLPKKVADRILGHAPANANVQPLFQKVFATLGIEEDGRMQADGFVFRSQFEMIMKI

ASDQKGDKYFSSWKRLRWQSVEEFVMKKCGRYQRLKKLAESNTELLRELFATGEGEDIHC

CFVKNDGSDKVGVDELKGKTILLDIRPVNLGYELPLQALTEMYVAEKERLNFEIITIPVN

GRYEKWDAIDKFSSDIPWLVLQSPWQLANVTKYFLVEGCALLKERDDEWDEWYPNIMRVI

DSNGRIAAHNSVGVLRMVNTWGAEAYPFT

>LL_023937_T.1

MDIVTGAASNTINIVISSLIQQIDDAIRMEERRRILEAQLNRMKVLLIDISDQFELRRKT

PPQSLKNCLQRMQDSVLKANNLIDRSQQPQAQLCIDFFFCKPRLSREIKDWNTSLNHLIR

ELNTDFTLFCNAQKVAAATAQAAEKKLNSFVKEDPQSRIIGLYGNGGVGKTTLLRKMFNN

YQVSDAFDDVIWATADQFSIGELQNVIASAVQLDLSGCHNDVELQRAKLCAYLKMKNFFL

VLDNMKKKIDLKELGVEFSNDKDSKLLFSTENRNLIEEMKADVCMKIQPMTTEDTWKMFR

NVAFKDSHVPGDIEHVARQIASECEGLPLAIKLIGSAMIGNNSLNEWQLVLSQMQRMDFS

FPISQPRIEPVLFQRLRFCYDCLPHANLKEGFLFCAAFAECEQIPLKALVHIWIAEGLLQ

ARDLDNLMKMGYNYVKLLEKRCLFQMKGDVITVHNVVRSMAICLGENEENCVFMAGRQLQ

YFPHIQNHEDCKRLSVYDNNLKSLPTKELRCPKLVSLFLGKNEELKEIPERFLLGFTSLR

VLELGIPVKSLPASLWKLIHLVFLDLSWTEIEDVPEDIVNLSHLQFLYLGYCRNLVSLPY

QLGDLENLKYLYLKGSYNLREIPEEINCNIEL

>LL_024007_T.1

MLFRRVAFKADQVSMDIEERARRFADECKGMPSAINIVAAIIGQTNVDQWDTCFSLMKTV

DPSFQATHPLLDAELYRLLRWSYDALPNSNVKNCFLYCAFFMEKKWAILPSSCTANQEVD

SRELVQMWIGEGLVKSKQGTYLMDMALARKYLQLLLDRCFFQIVHESTRVFRKKSLKSTE

PAHDVIPSIRVHAAIREMAKYIGQKEENFLFIGSQNLRNFPEVRNNNCRMISVANNFIEH

LPTDMRCAELASLILKGNTNLKELPSDFLLSQTSLRVLNLSG

>LL_024012_T.1

MAAEIIEKMEEISELQKDGSVLNTDFSRMKGLLIHIRNVFQSRQKRVPEALERSLEKMKD

ALKEAKDLIDEDKKQRERQCRFLFGNSKHSSKIRVWKAGFYALLQELEMAFSMSSNTGQI

AFSATPPAQRLLQPVPESDFVGSGIQSARKQLQTWLQAEPRGQVRVIGVHGTRGVGKTSL

LKIIYNTYKQEVSDLFDVIIWITASPGNQIEQLQASIAEALNLNLEGSSNADARKTQLNA

SLRRRRFLLILDNMDQQIDLNEVGVKTGDDTGSKILISGRDKNVVEGMGSRDYSRIIEPL

SPEEAWELFRRGAFKNGVAPAPDQNNIEGTAREIASTCGGIPLAINAVAAAMSFIKDHDQ

WSSALASITKLKSSLPRDLETIDPDLYPVSRWCYDKLPDSKLKICFLNCAAFLPDKEIAV

ETLLEMWEAEGLLEVQMDARRRCIKELVSRCLVERVEYIVDDNYHSVEYLKIHGAFRGMA

IYIGQKEENCLFAAGQQSQNFPNERDTRDCKRISVGHTAIKDLPTDLKCSNLESLILASN

IRLEEVPKGFLSKSINSLKVLDLSRTSIKTLPTSVDQLEQLVFLNLTECIKLVDLPDSLC

KLSSLQVLNLNSCSALLSLPSNIAELKNLKHLRLDACYSLNGIPQNLSQLISLNTLILPL

MESICALSVVDLSKLSNLMELTTVVKSEDLSKQNEVGTKATRLQMRNLTLEYDDDAGADR

DTIAHEHVILKNMGDMENLQNFHLIKYQGVTLPSSVYQFQNMKMLLLHSCNQLKAFLDWD

NSTGDSFPKLEKLVLRNLLKLENLAGPLELENLAEPLELESIGGPSCMWKEGTMSQLQLL

VIFNCPLLKELPSGMKLPHLKELHITMCNEVQKLDIGSGDFPMLETVILDELNMLQSISD

KVWTDSTMPKLQMLEFIDCPLLKRLPAGMETLSGLRQINGELDWWQSIIWENDDKKTKLS

QLFMII

>LL_024024_T.1

MASLVGASASSSMASASSSSYDVFINHRGPDVKNSFASHLYRRLSENGLRAFLDRDEMEK

GGEISSQIDKAIRGASVHIAIFSPRYAESDWCLHELVLMMKKYSLEYPRVTILPIFYKID

PSVVRWTNEKGAYFEALRALEQKKTKDSENGEAKPRYGVETVQRWRDALSFVADLSGFTL

GPEDDEVVLLDSLMEEVRIKVPRTPLDVAKYPTGLDLKLEEFGKMVERESVKVKVIGIVG

VGGVGKTTLAKHFFNNRRSSFSASSFLWDVREAAGRMSLNDLQSKLIKDLKPKDKDIKID

RHEGIEILKKHLSLCEALIILDDVDHVSQLDAFLPIKDVISPTSLILVTSRDKHVLKRSG

ILESSIYNLEGLDLPQSKELFCSHAFNQPLPSPEVADLVDEFVTACKGLPLSLKVIGALL

CGQYDRCYWQDQLERLNQTLPDEIFTTLRISYDSLTKQDQPIFLDIACFFIGEDIDKGIR

IWGMAGVRNLLNKCLLEVDTKNKIKMHDHIRDMGRSIAEEPTMPLRLWDSTINIIDNLSV

QQSTVIREVRGINVVSSSYYGWFRDSENVWSRNRRGSQISPSRYLFNNSHFPAMTNLQVL

VTEEDQLDDMLRRVQPPHLIWLRWYRISHSSLPNWIPMKNLKVLEVEGGRLNMLWPQQSQ

GVPFDLRELNIWAPLSEIPKSIGQLKYLERIAIYNADSHGAHGALERLPDEFCRLRSLKY

LVFKGFSKMVSLPDSFGDLTNLQHIDLHYAHSLQTLPDSFGELTNLQHIDLDSARSLQTL

PDSFGELTNLQHINLASANSLQTLPESFGKLTRLKHLSLRGCKSLTISSGTLGDITTLEY

LDLSYCEHVNELPPQVADQLSLQTLYLEGMKLKELPSVACNLKVLKLGGDLLEMLPPNLG

DLKSLKSLSVSDSPQLKSLPDSVGLLTQLTELEIKWCGIEYLPGSLLQMNMLESLVVWNC

PVQEHPFKKEVEGERETEGETGCSRCMFGLKCLILSFTKIREIYVDEDVCPNLQQLWLNN

CSVLRQIGGLCGLPKLKDLKIYDCTQVELSGLETLTSLEKLTFWQLDKLQSIPWLAQLTT

LRKLNVIRCPEIQALPDVGHLTCLESLSFEGCKKLQSIPGLWQLTKLRELDVSQCHEIPE

LPGVEHLTSLEKLNISGLWKLESIQGLGQLSKLRRLNVSECREISELPGVEHLTSLKRLN

ISGLSKLESIQGLGQLTQLRKLNVGRCCELRDLPGVEYLMSLEIVNACECPKLRWRQGIL

EQLRQQLEEGFIYEEQRNCLEFLLWSFCYCLDQS

>LL_024029_T.1

MDGVELVKNCEKTSLYNVYQNLRYASKIHQLEKEICHFVHYQMPVQLLQDVKNVITELKS

LRHLYESRSVDERKLNETIVPKLTEDPFQNATMLQRMGPDGMCDGAFDEAPPCRYDDSVK

DDFVVGLEKNIWELKKILLQKEVSVVGVQGMGGVGKTTVALALSSDRDIKGAFRNNIIFI

TVSESPNLKGILETMWVKLFQRKKPEFQNVEDAHRQIQEQLLRQAKPILVVLDDVWSRAN

LEK

>LL_024052_T.1

MASPTFASSSTAASPTSDNSYHVFISHRGVDVKKTFASHLYRGLLSHGLRTFFDKQELQV

GESLTCQIKNAIRSASVHVAIISPGYSESKWCLQELLLMLESRAPIIPVFYHVKPTDLFW

TSAEGVYGKALHLLENKEADSSHKPLRYSSTTIENWTNSLSRVTEISGLDLEAACNGDEG

ELVEKIVKHVFKKLKKPGLHVAKYPTGLNEKLDDFENRVLRKHQNEKTLVVGIVGMGGAG

KTTLAKELFNRRCSNYSKSYFLNDVRESKRSLHSLQIKLLKGLTQMDLAIESVDAGIEML

KEHLLSSCQSQLIVLDDVDGVDQVDALLPDQTVLCSGSLVVITSRNKDVLTSAGVQDSSI

YHLTGLNPRHSRHLFCLHAFSQPHPLPGFEYMVDMFLKACDGLPLSLKVFGSLLYGKNDL

SYWQDQLDRLQQLPGEIQERLRISYEALNKEEKKIFLDIACFFIGENRDMVTRIWDGSGW

KGLLGFRNLQNRSLVEVDTENNIRMHDHLRDLGRDIAEVDSGSQLCLWSPTENIDDLLQE

ASVIAGVRGIRMCPSEYNDDADLIFPSEYGDNDDQYPSFCTERRLQPVNTRKLQLVDIED

YLLERILRRVQSPNLIWLRWKECPCSSLPPWVPMKNLRVLEVDGNSLDTLWESESQAPLQ

LRELMIT

>LL_024121_T.1

MTMANFVTAAASSFSASTSASGRSHYDVFISHREADVKNTFASHLYRRLRDCGLRAFLDQ

DELKKGYNFPSQRELAIRGAKVQIAILSPAYAESRWYLDELVLMKNSGATILPIFFKVDP

SEVRDTLKGVYAKFLRELEKTTTSDPQTPEKKPRYAPETIQNWRDALSYVANLSGFVHRD

QDDEGELSENVVEGVLKELLKTPLDVAKYPAGLDNKLEDLKKTVLYHQQQQQESERLETK

VIGIVGIGGVGKTTLAKSFFNSQRSSFKESSFLSEVRESAARMSLNSLQSKLIKDLLHTD

EITVDSHNGIELFQRHLKSCHAMIVLDDIDHISQLEAFLPLKNILSPGSLILVTSRDKHV

LISSGVSEQSIYNLKGLDTPNSQELFCFHAFHKPLPPPEFADLVDRFVTACVGLPLFLKV

FGAQVCGLNERSLWEEILDELGKTLSIEIYDRLKISYDSLEPGVKEIFLDIACFFLGEDG

DKAMRIWGKVGIRNLQLRCLVELDGQNKIQMHDHIRNMGRKIAEEVSRPLRLWHCSTASI

EDLLVQRTSVINEVRGIKMDIPKELRREMGHLSFCEGLQMRLRHLRLLPVTEGYLEDILR

RVQLPHLIWLRWYGCNYSSLPDWIPMKNLRVLEVEGSTLNTLWAHQSQAVPFQLRELNIY

APLSEIPKSIGQLELLERIVIRCPSPKSTLKSLPDEFCNLLSLKYLVFDEFSGMLLLPDR

FGELTNLQHIDLDGAASLQRLPNSFGNLQQLKHISLNGASSLQWLPYSFGNLANLERLDI

GGCEKLQFIPGLGQFTKLTHLDVHGCREIRELPGVEHLTSLQLLNVGECQKLQSIPWLGQ

FTELTHLYITELTHLNIKGCSEILELPGEEHLTSLERLDIGGCEKFQSIPGLGQLTKLTH

LDVHGCREIRELPGVEHLTSLEKLDVGGCEKLQFIRGLGQLTKLTHLDVHGCRDIQELDV

HGCREIRELLGVEHLTSLERLDIGGCEKLQFIPGLGQFTKLTHLDVHGCREIRELPGVEH

LTSLEYLNVGGCKKLQSIPGLVQLVQIRQLNERHRKRCSCM

>LL_024187_T.1

MASASTSTAVNSCYEIFLNHRGPDTKKTFTSHLYRRLVLHGFRVFWDKEEMQVGDSLTSQ

IHEAIKAASLHVAIFSPGYAESKWCLEELVLMLESKKPIIPVFYGVHPTDVRQTQGDGVF

AQALRNLQKKRRHDSSQPRYNSTAIENWRTALSTVAEISGLELDKFSGDEGELLDKVVDS

VLKKMGKTRFDVAEHPTGLDQKIEDFEEKVLSVQREHTGKPQVIGIEGFGGIGKTTLVKE

LFNRKSSQYQKSYFLFDVRENARKASLESLQQKLLKGLTQIDQNIESVSEGIQLLKTRLS

SCDALVILDDVDHINQLDALLRPIRDVLHPHSLILITSRDKGVLRSSKIEESSIYKLTGL

NRPQSQELFC

>LL_024256_T.1

MADPGFVPGFIGVGIQMAIDQIIHHINIAVSCRKELASLKEMVKSMQPIITQIQQNRVQT

EASAVNEWLTQLDEILRQASNMARHCTVPRFDFVSRYQTSKRITGLISNIEKHLKLVQLI

NLAQTQGLHAMQREIAEDAGSSSASASTSTAATAGFFIEEALIVGQEEALESLEKSVMAD

CESLLSIGVVGKGGSGKTLLLRRLFNSQKVQNLFRNDLLLWLNVCYPLSFPSLRNELCAQ

IAIQTHVDLDKNMSQGDVRIWLKENMGRKKFALFLDNVWREGGKLLEELGLVHLINHSNS

KVIVSSRDTTTLLQMGVAETSIVRMEDLMDKQCWELFGHHAFPFNHGKPPANIDEETAKL

VCANCGGLPLAIKVVGRAMAGITNPQQWKFAVGRVPKVHSSYEQSSDLYKCLRLSYDALD

INLQLCFLYLAAASQKSQILSGQSHVVQLCIGEGLMATKELQNEPGYDPFEMGRSYLHSL

ADRCLIEPIARDLEGCVLWFRMHDVLYDLALQIAETEENCYFRPGRGFPELRENECSGFT

RISLNYNTLSSLPKSFRAPEICSLLLRNNKDLTEIPKRVIGSMVSLKVLDLSWTSLHSLP

DSLGCLKQLVCLRLSGVPLRRL

>LL_024299_T.1

MQEKYIVADERGNVNVNLDELMEHISLLLKLIEKDNGPHKKPTIPNIWTYSLDWKNLYDL

LSSGVERLSALKDVISGPVKEILKGMGYASLATTGLLFVVYSLERYEELSSNKEECLSLL

KEMCRFVDVVKRLKESPEGLQNIIEDATALIIEGAIMCCSQIDSSPWKNFFKAPINKADL

SGINQKLHEMCSKIDLQVDLLVFKDVRTYGPVLKNIQTYGPVLNDVQNTTRRILTEIEHK

TPPLSRKYPTDAVGIEESVKEVLDLLECESEQKAVTVILHGFGGMGKTTLADAVFSQVGD

GGRKYSKVELFTDIDSKPDIKKLQRKILEELMGPGKTIPEFTTDRDGQRELAEVLREVHA

FIYIDNALKRDALTKLLPQNMDTGTNVRLLVTTRDENVKFACPNPKIYHMKGIQGAEAIL

ILKNKIKEGINDNQFNDIVRICGEIPLMLTLAAAFIGGADDKLKAYDRLMQDQGKWENES

FHEIHCAFAYDAIPKNCRDPFLDICSYFKGRDWDTVADIVGQEVLESLASRALVTKDANG

NASVHDVILSLACQKSKEESQGKEVRLTFTTETQLEEFLEAKDTEGIKGIWVSADDIWHQ

LSTLGTSSSRRDAVDISSSTLDTMHSSLRVLHLGYVKTVSGNCSKIFKKLVYFEAEITQL

PFDVSKLEQLRYLCYKPTELKVLPKMPSSLRHIEFGDMPSDLSFMLHDRLSMYTFQISST

YLQQLGHLRILRLNGCDNLKNLPKDLGDLVNGLQELSLSSWRHIEKLPESISRLQSLRIL

KIENCSSLTELPANIGLLSSLQELSLRRCTKLVALPESVKNLSSLTLLNLNGCGNLVNLP

DGLGNLTGLVKLSLNYVSRLESIPESIRELKLLQTTSLSMCTFKKELPHGFCNLLFLKSL

SLELCARLRKLPEAFGQLKYLEDLVLKDCHQLKELCNDFHCLSSLRNLDLSNCYSLDKLP

EDFHRLPSMENLYLRSCKRLKGKWMDRVVNMKRLQVVHIEKSKQLFKRWAEIQRDSDPSW

SFSVISGQALWMSEVQKENIEKNILSDFDRLLTDVQGELFHISHLPLNTLILVICDIHPI

IPLKTCFPWKLLEEKIDEIQTNFKIIYLGPYCNLLAEKVKNITLVGDLPTVRVREVFEKV

WSTLDTGTFAEIEGQDGYFQLVGNGKSDEYRPCWKHLTATQLEELVLDNCGKYIRLKKLL

ETTQNSNIELLTELFAPEAHQTTDVFQKNFQDCGVDELKGKTILLEIRHTKLSYDIRVQS

LKEMYDVVKKEKLALEIISIPIYEPGRWESFEESRESGAYEQFLKEIPWLVFIERNSRKE

VIRRVLFLAKKCGWKECDEYENWYPSINLIIDPCGKVIIYNSSTIMPTMGEVGKFLSNYL

ESVSYQVRWGAKVCPIMDEKIEKLNKEEWKQLKNMSTLEFLFCHLERVSSEVKKLMFQKN

IICLCSHMGLFCLQSLITKNDDVHIICIPEYDARNMKRFQVTYATYKYFYYSLPDWNYIG

LRNICLLRRDEALRFWMRILYLKEEINGMDESNEQLWKLKCFLQSFQFLLDKDKFKAFSL

PLDTENNFMTFMNKDGKIIATCDGEIVYWIYNSVTAMDKREQLLQQLIKNVEKGNETHGE

DSSISHSLTKNIQTAVDASEQKNVDVDLDLDILGNYIHELLLEEQLYHFFKINKERKKWS

QPKAIWPTSEGFRMLKDFLLKGINVNWVQDIISGNEEDKWKTVTKRLLKDIGQTQWAAAG

LLMVADVLERFNTVSTNQNLCLFLLDEMYRMALYLKKKFNEKYRLPTWMKREATGSIVIG

SIMCCAQIDPSKFAVFIDEPVDEKMLRAFLEELELKCNGMVHALSKGLKKFIYEEYSTGQ

IRKDPRRVFNLLADFIIGHLEGESEDEGGSSFESEDEGGSSSEGESGDEGGSSSEGESGD

EGGSSSKGKGCPTVTIREE

>LL_024308_T.1

MAQLTSAAQHKFTSIDLPALILLLILLFLILLLIRSTNSFILLPVTAQVTTPLDHPLASA

SSSSSSTCSASKTSYPQYHAFINHRGPDVKNTFARNLYKILKSCGVRVFLDKQEMVEGHP

ITSQTKSAIETASVHIAIFSESYAKSFWCLDELVHMVNSGTTILPVFYKVEPSVVRWTGR

DSNGEYAKALRNHEEKQRYDFQTVQNWRKALSRVADISGFELKAHDGEEEENRLLNNVVL

GVLRNVPKTPLDVAKHPTGLEKGIENFERAVLSQQEEERVEAKVVGIVGVGGVGKTTLAK

EFFNRKRSEYGCSSFLFDVREKAATKSLNSLHKQLIVDLKHSDDIKFERCQESIGILKKA

LIILDIVDDVDQLNFFLPIKHVLDPKSLILVTTRDKHVLSSAEIAESSIYHLCGLDKSHS

QELFCRHAFFRQHPLPGFENLVDEILAACDGLPPSLKLFGALVRGHDKAYWEDVLKELRQ

>LL_024396_T.1

MASTVAGEAMASTAAGDAITNPVIRLMKDVIKDVIKEEDNRQILHTDLRSMESLLQYTDI

FFPYQQKEPPEIVKIWLQLLQNSLTEANGLFHRSGRRQSCLDGVVCKPGRLSKEIREWKA

FFDHLYGGFSNDLFLHISSDAIISQVIDLVIELLKDVSKEESSRPILRTDLRRMETLLGD

ITLFRDEPNITTLFQPLKLLQKSLSEAKGLRDLCEPRQSFLDRVVCDSWRLSKERREWSA

SFDKIYHGFQSDLSILVTSPQFLSPKALHKDEALVQGVAAPGFVGSGIIVAQAQVERWLS

ERDRFHVIGVYGLPGVGNTSLLRMLYNKVNHVFEERIWVTVSEYYKMFDLQSAIQRQCQG

NLNLPDSLDERKVKLSAYLKKKNFLLVLDGLWSRLDLQELGVAFGDHKHSKVLFSTRRKD

LIPEMSADESTEIQPLSKEEGWMLFRRVVFKDDKVSMDIEDRARKIADQCRGVSLAIHVV

AAAMIGQTSLHEWDICLSSIQNVDPSFPANGSGLDAELYAALRFSYDALPNSDVKKCFLY

CALYNEEQEIDTLELVQMWIGEGVVKSKQGTYLMDMAVGRKYLKLLVDRCLFQSVKDSWN

VFTQNKIRVQSAIRAMAIYIGQKEENCLFMATQNQRNFPDVPADENCRRISVASNFMEDL

PSQLRCPKLATLTLTDNSHLRELPSEFVRSLTSLRVLNLSGCTQMASVPTSIEKLRMLEF

LSLTHLPIQDLPKEVCRLSKLQFLDLSNCESLKSLPSEIGEIKNLKSLNLYGCRSLKTIP

REISELTSLSRLVLPWQMYLSMETEAPASIWSLKGLKNMTELTVSIKRGQQLKDGIMGSW

SEMRHLMLRYYGGHEDLPEDMKSMKKLQSFMLWGYRGSSLPNCKFEHLEVVSLGNCDNIS

DLSPLEWMPNLKLLKLRSYFGLRELGIGSRGSGSPSGYQRLQKLVLKELPHLESLGGASK

RGVWDERTLPHLRVLKIRGCDSLRRFPVGMEKLPNLCALMGEDFWWRSIMWEDEDMKTKL

EKNFKSY

>LL_024553_T.1

MLKLEGKTTSDPNKPRYASETIQKWRAALSYVANLVGFVHSDRDDEGELLDNVVERVLKE

IPKTPLDVAKYPTGLDNKLEYFKKTVLYQQQQESERLETKVIGIVGIGGVGKTTLAKSFF

NSQRSSFKESSFLSDVRDSAARMSLNSLQSKLIKDLLHTDKITVDSHNGIELFQRHLKSC

HAIIVLDDIDHISQLDAFLPLKKILSPGSLILVTSRDKHVLISSGVSEQSIYHLKGLDTP

NSQELFCFHAFHKPLPPPEFADLVDRFVEACDGLPLSLKVFGAQVCGLNERSLWEEILDE

LDKTLPTEIYDKLKISYESLDQQDKEIFLDVACLFLGEEAEKAMRIWGKGRFRNLQDRCL

VEVDEYGYGNRNKIKMHDHIRDMGRKIAEEGLPLRLWDCKTNNIDDWLESRTTLINEVRG

IKMVCPRGDGFRHTQFWHFWWPNTEMGQLSFFKCLEMRVRHFRHLRLVAAEKGDLEDILR

RVQSPHLIWLRWYGCNYSSLPDWIPMENLRVLEVQGSTLNTLW

>LL_024638_T.1

MDIDSVDQGKGMLRHYLKDYRVLLVLDDVDDLDQVDALLPVDVLHRDSFILITSRNRNVL

EKSGIRESSIYQLRGLDAQHSRELFCSYAFCQPHPLSGFESLADQFLKACNGLPLSLKVF

GGLVWGNTDKSYWEDQLDRLQQTLPDEIQKILQISYDALHREEKQIFLDISCFFIGLNRE

TAIRIWDGSGWRGSLGFRTLQEKCLVEVGIDNKIIMHDQLRDMGRELAKESALPRRIWHG

LEDIHDGAELSDRIWRWMQQSSVITDVRGIKLFHSDYEYASFSCSKIRNLQLLDASRVPF

LQLPRLCYETLLRRLGSETLLERILSSVLSPNLIWLRWHNCPYSSLPSWIPVKNLRVLEV

DTMTFQTLWQSESQIPLELRELQIDGPAYQWPPKLQSDAPLLNIPKSIGQCKHLERIVVN

DHSNFENLPEEFGLLRSLKVLVFKSCKKLRFLPDSFGNLTNLEHIDLSQCSGLETLPKSF

GNLKNLEHIDLSHCGVLEKLPNSFRNLTNLEHFDLSHCSGLETLPNSFGNLTNLKHIDLS

YCSGLETLPNSFGNLTRLEKLNLTETNLEELPGAIGKLCALEKFTLQSPLLETLPPSIGD

LKSLRYLMLWRCEKLKCLPDSMRLLNQLTEMTVYVCSLGDLPFKRVEGESETLTRLQQLE

LTMIQKLDKVSFGEGVCPHLQDLVIWSCNDLAEIGKLPNALIKLELRKCNKLRKIEGLCG

LAKLQELNINFCTKVEELSSIETLVSLETLLVIHCWNLKSIRGLGQLTKLRFLNMSGSFL

LEELPDLEQLILLERLETTECFKLKSIRGLAQLKKLQGLIVDHCRELEVFPGVEHLRSLS

MLSASGCPKLQWAGGAVEQFKRQCRYFSLK

>LL_024656_T.1

MAKEIETGTTSQVLGEIINKGFVAQIIQEIDALLINEDQTNQFLQDKLERMKALLQDMST

AFEHQNTGAPSESIEYCLKRMREAIQKAKALIDAQSQPQKRSRYSILNAPIDAQSQPEEG

SSSSISNALIDAQSQPQQWGRYSISNLLNAENIRKFNAELDQPFQDLKIPREISKLNAEL

DQLFQDLKNAFDMVANARLAMMPLQSPAWPQSSASAPPQETDVLLQPMTEAGLLGPRSIE

SAETRLQIVLTTPLQSSAMPQSYETQLQIVPATAPQSSLMPQSSETQIRIVPATPPQSSA

MPQFYETQTQHQIVPATPLQSSAMPQSYETQVQIVGRAIRSAETQLKTWIETPRVQVIAV

YGMPGVGKTTLLDRVYKFYKHTNVFDSVIWVTVSRIYEIRDLQGHIAKKLELNLSGISDS

DADTAKRMLSESLIEKKFLLILDDIWSALDLKQLGVAFGTDKGSKVVFSTRHRDLALSEA

DESMNVESLSKDEGWELFARVAFKGGHVPEELQECARKIADECESLPLAITVVASVMRGK

GTKVDDWNSCLFLMKNADRSFADTHPSVDHLLYQRIRWSYDDLPTNLQNCFLYCALFPED

EQIWVDELVRMWIAEGFCKSNDRTYSMDMAIGYSYVKLLIDRGLFQSGNFGKQVMDKIVI

KPLNVWKKRFITVHDVIRDMAISIAEGEQDKKHLCRAGVQLKCFPRNPTQDCKRMSLRRN

QIESVPEDFPCKQLVSLILSQNPLVSNVRGSFLKNLSSLRVLDLSNTQIESVPTSLTQLR

LLDLSNTQIKSVPTSFTELQFLTLRSTCIKELPREIGNLSDLQFLDISECKYLTSLPTEI

SQLTSLKRLHLSMGMENAVKDPTNIMALRGLKHLIEFHLYMFFKFENHEIMGTIMGSWVE

M

>LL_024660_T.1

MITSPSPNLLSCSPALPVFIVVYVGKTLVPLRLPSWGVPHRLFDDSASSLAMATAIELGR

SRYKKSEIYDVFLSFKGRDVRRGFVDHLYNALTDSGFRVFLNIHHLGQSGEDIFASLQHA

IDEVSRMLIPIFSLN

>LL_024696_T.1

MSGFICLNIDTAIKQILRHISNAVRYEDELKPLKELVTSMEATMRQIQHYCLLITWKEDT

AVNDWVTNFNRWLNELDGFLQEASEVAQNCTHVSTHQGILCDKMIKRIADSTSKIAERLQ

KMQPSSSDKSDKLIDEARAKGIVADARHITPAWQTVTRSSGVRDILAPSTTSATISQAAL

DKLPTGRMKLMDEPFIVGQEKYLERLQNLVTNESPTTKKIGVLGQGGSGKTLLLKRLFNS

EKVRNHFKDGFLLWLTVSKSPSFTSLRNKLWTQIKIQDNKGAVKNINEQHVEI

>LL_024697_T.1

MSAENLLDTLGILESVTNNPNSKVIVSSRDTAALSKMEIADVDIEKIKSLNEHDSWKLFS

SHAFPNDNRNPPGKEEGKLVCQMCQGLPLAIKVVGKAMARSTDRKQWRWALQRLSNIKSV

YDCRLRFSFEALGNEGVKMQCCFLLVAATSLEDEVLFARHVIVLWAGEGLLSGKTVQEEG

YDPFEMGWNYLTLLADRYLIEPMMRDHDGRVVCFRIHDELRTLAIQIAEKQENFYCSAGK

KSPVLNGAEYSGCTRIFWSYKESIDQSPKASSGSEDSVQVCSLSISESEDSVLEKIPGKM

LGSMSYLKFLDLSWTGVKSLPDTLGKLKQLVCLRLMGVPIKILPQSLTTLVNLQILDLSF

SSVTELPSGLHMLKSLRYLGLSHCKDLQYLPHNILALTSLQYLHMIECKSLWTNAEQYKW

KKVASISDLGKLSKVKWLELQNNGVTVDEKAIGKIVEVETLRLTLTGMKSLPSGISEMSK

LKRLALKCPDMDNMGLSHVKNKKFDDYFQNLNCLILFKCDKLVELPDFHKLRGLQRLEII

TCRKLKKLPEAFGEAGAFSSLKIFSLVRLEKLEKLPEIKEGAMPSLQIFTIMECPVLEGL

SRDEYLKLKTLQKFRIYSCPKVAEEFEKWKKVTEPNKMVKLMSIKDTEEIKKWHFQLCVK

HGSWVYGELWCNELFLFLGGLDTLV

>LL_024760_T.1

MAESSSSSTSTYDVFINNCGPDVQKIFVCYLYRLFLSYGLKVFLDPEDLKRGDYSNSQME

GVIRTASLHVAIFSSEYPGPTWCLKEFILMKYDPQTPQETPRHDPATLGQWRNALSIFAG

ESGFELEDCNR

>LL_024815_T.1

MSERIRDLTAKIAQHLQLMSSMVGDARAEGIVADARHITPAWQTVTPSSGLRDILAPSTT

ISQAVLDMLGTERMKLIDEPLIVGQRNTLERLERLVTNEELLTTNKIGVGVHGKGGSGKT

LLLKTLFNSEKVRNYFSGGFLLWLTVSESPSFTSLRNKLWTQIKIQDKKGTVKNINEQHV

EIWIAIQNNVDVIQNTEKDEVKKRVNEALQHSSGFVLFLDDVWESDAGNLLNELGVQQET

VTKNPNAKVIFSSRNRQALPNMEIVEVEDLNENDSWKLFSHHAFPHDNKNPLGKEEGKLV

CQICQGLPLAIKVVGKAMAGSTDRKQWRWALQRLSNAKSVHDCRLGFSFEALGNEGVKMQ

CCFLLVAAASLEDEVLFARHVIVLWAGEGLLSGKMVEEEGYDPFEMGWNYLTLLADRYLI

EPMMRDHDGRVVCFRIHDELRNWAIQIAERQENFYCFVSRNSTVLKGNECSDSTRIFLGH

NESLDQSLEAPQFCSLSISENSVLKEIPRRVLGSMSALKFLDLSWTGVKSLPDTLGKLKQ

LVCLRLMGLPIKILPASLTTLVNLEILDLSFSSIEELPSGLEMLKSLRYLGLNHCKVLQH

LPGSISALTSLQYLHMIECNRLWTNAERKYKKVARISDLGKLTKVKWLELQNNGETVDEK

TLGKMAEVETLRLTLTVMKSLPSSI

>LL_024856_T.1

MANVPRVSGNAMVEPVMEMMKYLIQKEDNRQILQTDLRPYMETLLQYTHIFFGYEQKDLP

EMVKIWLQLLQNSLTEANGLFHRSRPRQICLDCVVRSPGRLSKEIGEWNALFNHLYYGFL

SDLIQHIALPGNAMISVLVNPAANLLKDVVEKEDSRQILQIDLRRMESLLRDFTTLFQVP

EKKALREIMKTWLPLLLNSLVEAKGLLDSSATRQSFLDCLSIDRSGPRQRRLSKEIGDWS

KFFDHLYHGFQSDLSILVTSLQFLSSNPLHIEEKAEEPAVIQPLSRDKAWMLFRRVAFKD

DQVSMDIEERARRFADECKGMPFAINIVAAMIGQTNVDQWDTCFSLLKPVDPSFQATHSL

LDAELYRLLRWSYDALPNSNVKNCFLYCAFFMEYQKVGSRELVQMWISEGLVKSKQGTYL

MDMALARKYLQLLIDRCFFQIVKDSTNVFRFTGYQAHDVIPSIIVHAAIREMAMYIGQKE

ENCLFIGSQNLRNFPEVHNNNCRRMRISVADNFIEHLPTDMRCAELASLILKGNTNLKEL

PSDFLLSLTSLRVLNLSRCTEITSLPTSMGQLKLLEFLSLSGLRNIKYLGEEICNLSRLQ

FLNL

>LL_024864_T.1

MKLYSSLENKRFLLILDDVWRPIDLEQVGVNFGHDNSSRVLISSRSKDVVETMAANEYCM

MVQTLSREEGWELFRGGAFRNGAAPDNNVEAIARGIADKCQGLPLAIIVVAAALARKDSA

DEWRRALDLMRNVYPSFASTHSCTIDVELYQRLRWTYNDLPSYNLKMCFLYCAAFPEDAW

IPVETLVELWTAVSLVPQRRATYYMDVGREYIKALVDRCLIQYVEEEDVMDKKAYIRVHD

VLRDMAIYVGKGEEKCLFLSSQHLQHFPDEEHTRDRKRISLFDNQISHLPTDLKCQTLLS

LVLARNRELEEIPESFFRNFTSLKVLDLSYTSIEALPTSVGQLGQLEFLNLECCYFLTDL

PNSICNLSHLQFLNTRGCHRLNYFPDIIGQLKNLKRLEHSTDVQLNPHQICQLTSLNRLV

LPEGSPMTLEDLTNLSNLIELHVKVKPEIKGGSMSSWSEMRKLTLIFTSDDVEDDAALDI

LPQNMQSMNNLQYLSLVQYRGVSLPNCISNFQNLKELQLCSCTNLKELPGLEIGSDAASD

GFPMLERLHLENLDSLEIIVWNEKTMLKLENLWISRCPVLKSVRTEELPNLRNLQIEYCD

ELSNVSQIGSLPMLERLTFQNLEKLESIAHWNDKSMPNLQNLEVIDCPMLKGCQMDWINY

SI

>LL_024868_T.1

MDIFNCKNACCRLDGLLMGLIQTLQQMCSLNLEAPQNEQNPSYEELLSTLMKGVELVKEC

EKISHFTILHNLGYASRIHQLEKDICGFVQYQMPPQVLLSVKNLITELQTFHHLYELGSL

DESETNETIFRYVADLTNDPHKNAMMLQQMGADDMFDGAFDVAPCSYYLSVKSDFVMGLE

KNIRNLKRILLQGEKSIVGVQGMGGLGKTTLALALFNDQEIKGVFQNNVIFITVSQSPNL

KGILETMWEKIVCKKKPEFQNVEDAHTQLQQQLLRQATPTLVVLDDVWSRADLENLLFEG

EGYKTLVTTRDCSTIPQTHCTELYQLPLLDDADALSLFCLWAFGQRSIPCTAAENLVKQV

QAECKGLPLALKVIGSSLHGEPLPAWESAKRKLLNGEPISDYHKEGLLRCLETSIDALNE

ETRECFLDLGSFPEDRKICAESLLDIWVYVRKMEWQDAFVILLKLASRNLLNLTSNLRSP

AINYGSASELYFSQHNVIRHLAVYLASR

>LL_024951_T.1

MSSILFDVKDVRENAARISLDSLQRELIKELKNRDIPIKRWEEGKEILKKNLSSSDHALI

VVDDVDHIDQLDAFLPIKDVLHPDSLILVTSRDKGVLRRAKIVESSIYHLRGLTRAHSQQ

LFCCYAFSQQVPKPGFKNLVEGFIETCHGLPLSLKVLGALLCGEDDHSYWKELLDGLNIV

LPDDIEKSLRISYNSLNEEEQQIFLDIACFCIGRDRD

>LL_025057_T.1

MKKHGTALRQWKNALYQMRNVPETFDGLHSIVEKELFRRLQWSYDFLSDNLKACFISFAA

FPEDTVISCQEVADIWVSEGLVKGTGDSYLLDTADSFIIHLRNRCLIEVVRTDPVGRISY

VKIHDILRDLAIRIGEKKHGCYFKAGQGMSYFPLEEVDSERCAKLSLMSNNIQSLPQTFT

CSNLSVLLLSQNPDIKEVPESFLNELPSLMVLDLSETGITCLPPSIGNLKRLVSLQLKKT

GICELPETI

>LL_025069_T.1

MEKKSCRCRCPLFSCFACFSDENTTPSTAVPNAANEALTHPYHDSQTPVAPPAASSSEAT

TSTDVRSASLSSSSSQPIVPSAAPLSSSHSQPNTSPSSSNAASSSSGSKADLDGHIQNIR

TCANALHTLNVDADLDGLIESVSQFLEVIKEEKSTGNESSKTKLPAKNLWQSPSESLKKF

KEVLSEGINVFKDIIPGEAKTAVQGLLKGMGTAHLATTGLLIVALVLERLETVSANRKEC

LCVLDEICILAQHVKKLQQRPQLKEGMEDSMKEAMGLIVEAAIMCSTQIGSSKRWKCSFW

SASGDKEELVDFQQKLQRMYRHMDSEMGICIYDASDCRQMRSQKPRERQYPDYAVGIEEQ

LKEVTKLLEWESENNAVAVILHGFGGMGKTTLADAVFARLYIEGCKYSMVKLFDDINSTP

STSDILKLQTYILKDLQMGTLGEDIRTVEEGQREIGSILQREKAFIYVDNVLLRYPLEQL

LPRDMVNAKKLRLLITTRDIDACSECTKMITHFYDVKGIPHDDAKSLLKTGMKSQTDDME

EQLNSSQLDRIAEICGGIPKLLEVVAGFIRGQKDKQKAYNKVIQEKEENWKGKIGEGITH

YVFAYQNLPEKLKDPFLEICAFFNGWDWDTVANIVGDDELEMLEQRALVKKDPRGRLTVR

VHDVILAMGRQQTQGTRFTITNQSQIQNLEDEDVEKIKGIRLLSGKKGPMSISASIIDLM

CSSLRVLELGDSIKVEGKCEKTFKKLIYFVDEGFHLRFNVSKSKELKIFIGAAEDFNLFQ

MPSSLVHVELNG

>LL_025129_T.1

MKNLTSSAGSISSRNNYDYQIFINCRHPDNVLETFRTSLYESLCSHNLRVFLNGQEQPEG

KSEDAIRTASLHIAIFSPGYAESTECLDQLLLMLETGQQIIPVFFRVEGSKLRRTRDLEP

YDEALDFKEKNGVGIEITGKWRKAIHDVSNKKGLKRGSYNGDEGHLINQIVERVTKIFCA

YDVFINNAPDPDIQKKFASHLYSRLLFHGRRVFLDKKKFEGGDNLTPRITGVIENASVRI

AILSTKYVDSSCCLNELLSMVKSGKTIIPVFYRVKPSEVRWQIGAYSKLRRKIRAFSKLL

SQKGLCDLLLRRLKWQQSENGHGLLATKTVDSDTVENWEKALFKVAEISGFELDECNGDE

GLLVNKIVQEVLSSENATGKTAISYDVFICHRGPDSKNTLASHLYHRLVASGLRPFLDRE

SLQKGQELTSQIKKAIKNASVQIVIFSKYFGESEWCLKELEDMLKTKADIIPVFYNVDSS

YLRETYRDGDLVYSRALLKLKETYEERPRFPDLTGLYNRLIKGVKSGPIVPRYTPSTIVN

WSKAIFDVAGKPGGFHLQNCDGDEGVLLDNVVKNVVNRVNLLRKKRSNRRRRS

>LL_025137_T.1

MHRQGERRDEAKVVGIVGSGGVGKTTLVTKFFNRRKADYAVSSFLPHVRETAAKMSLNSL

QSKLIKDLQDMDIKIESVDEGRGILETNLKSCHALIVFDDVDHIDQMDALLTNHVLDPKS

LILITSRYTNVLRRLGIPEILVYKMTGFNLQRSQQLFCTYAFFQPYPPPGFAILVYNFAK

ACNGLPLCLKMFGALLCGVNDRSQWEEVLDKVGRILPEYTMKMLKITDRSQWEEVLDKVG

RILPEDTMKMLKITYDSLNEEELQIVLDIACFCIGKDRDLCTEIWHGSGWRGLVGFRNLE

DKCFVEVDTENKIRMHDHVRDIGRDISAFPYFPRRVLRATIEDLCDLLKQSSVSIQPS

>LL_025193_T.1

MVAYTLVIARAIFSRYYFHHPSIIQNLISMADLTIASASSSEASTSTAASTPYPRYDVFI

NHRGRDVKNTFASHLYRQLHDRGLQVFLDREELVEGHLFSHQIEEAIRGASVHVAIFSPN

YAESEWCLKELVYMVNSQAKILPVFYNVEPSELRRTRGSYGVALQNLQQKSTRDHQTGED

KPRYDADIIQSWREALSYVAGISGFELRGDESDLLEEVVRGVLKKVPKTLLQVATYPTGL

ANKLEEFERTVLAAEEDVKTVGAKVVGIVGHGGVGKSTLAKHFFNLKKSQFYESSFLFDV

RENARTMGLSDLQSQLIHELKHRRIEIKSDDLKSDDQGIEILKKHLLSCHALIILDDIDR

INQMEAFLPVTDILSPDSLIVVTSRDKHVLRSAGILESSIYYMKGLNKQDSQVLFCRHAF

HQHPPPREFEDLVGRFVTECDGLPLSLKVFGALVCGKEESYWNGILVELHERLPGEIQER

LIISYESLDKNDQSIFLDIVFFFIGEAIDEARRIWGTVGVVHLEDKCLLEVDSKNKIKMH

DHIRDMGRGIAQGGSIPRRLSEMRHLSHIDEVLERSSAGTTQVRGISLVHSTTPGLHKSH

PQMVWDSKMSRFKRWMHEVMRNCEWKLFNNNRYYATSLKILVTEDGYLKSILRRVRSPEL

IWLCWHRCPYSCLPSWVPMNNIRVLEVFGSRLTMLWQSEKAPLDLRGLTIAAPLSHFPKS

IGQLQYLEKIVVKSEDGVPLEALPEEFCNLRSLKYLELRWCKRMISLPESFGKLTNLQHI

DLLGAFTLQSLPYSFRNLTRLKHLNLSLCMSLKMSSGILGSGCPVEYLSLEYCDNVEELF

SQVALQSSLETLILRRLDFKEIPSAIGGLSNLEFLWIGGSIYERCNMMEEFPPSHFCLEN

LKTLYVESCPKLKCLHAFSEAFPQLTSLRISGCAILEYLPEDLLRLKNLRKLQVSFCPLR

ELPLNKLDSKDKCVSGLEELSLQEVNISEVSFWVCPNLQYLDLSECQQLRHIGELCGLAK

LRQLRIGDCRNLEELPSLETLMSLAYIEVYKCPKVKSIQGLAELTKLQRVEVHDLNELIE

LPGVERVKSLEFLDVQ

>LL_025200_T.1

MASTPTNSCYQIFLNHRGPDTKLTFTSHLYRRLVLHGFRVFWDKEEMRVGDTLTTQILDA

IKTSSVHVAIFSPRYAESHWCLDELFLMVESGKPIIPVFHHVKPSELRQTQGEGVYAQAL

HNLEEKKTNDGQPRYNSTTIGNWRIAFSTVAEISGLELDKFNRDEGELLDEVVDSVLQKL

RKSRLDVAEHPTGLDEKIKDFTNKVLLVQPQESGKAQVMGIVGLGGIGKTTLAKEFFNRK

SSLYHRSCFLFDIRENARKASLESLQKNLLKGLTQMDIKIGSVSEGIELLKTHLLSCQAL

IILDDVDDVNQLDALLRPIRTALQSDSLILITSRDKDVLTRCRVAESSIYKLTGLNTRHS

RELFCLHAFTQGHPVPGFEDLVGKYLNACDGLPLSLKVFGALLYEKDRSFWEDQLERLEE

ILPDEIQKRLQISYDALQKDEKQIFLDIACFFIGENRDTALRIWDGSRCKGRVGFQSLQN

KSLVDVDIKNEITMHDHLRDLGRDLAKDPGLPCRLWHWTENIDDLLEQSSVVTEVMGIRM

RLNNVVDNLYGSFSRDKLRKMKKLQLLDIDMYKFNLDNQDSEDDLLESILSVVHLPNLIW

LRCDNSHHDSLPSWIPMMKLRVLTVTSMNLETLWKEASQAPLQLRELQIASPLLRDIPKS

IGLLKHLEQIVLRCLYLEELPEEFYDLISLKHLTIKSCGIRSLPESFGKLTNLQYIDLSD

STQLEKLPDSFGNLTNLQHINLSRCENLERLPVSFRNLTGLKYLHLLACYNLTMSSETFG

NICTLEYIDLSFCERIEILPSQLAHQRSLEILDLELENLIELPSDIGELRSLRNLNLRNC

HRLKYLPDSLGRLNQLANLTIYNCLLRQLSFKMVEGEGGSICPNLQRLHINGCENLVEVG

TLPHGLIKLELIDCPKLRKIEGISSLAKLQMLNLRDSIEVQQLPSLETLVSLEELDVSGC

VKLRSIWGLAHLTKLRSLDLSGCSELVELIGAEQLTSSTWLNAYACPKLQLGEGVISRCI

FYKR

>LL_025247_T.1

MQDKKGAVKNINEQHVEIWIAIQNNVDDIKNTKQEKVDDIKNTKQEKVDDIKNTKQANVK

KWVNEALQQSSGFVLFLDDVWESDAVKLLNDLGIQQETVTNKPNSKVIVSSRDTAALSKM

EIADIEKIKSLNEHDSWKLFSSHAFPNDNRNPPGKEEAKLVCQMCQGLPLAIKVVGKAMA

GSTDRKQWRWALQRLSNIKSVYDCRLRFSFEALGNEGVKMQCCFLLVAATSLEDEILFAR

HVIVLWAGEGLLSGKTVHEEGYDPFEMGWNYLSLLADRYLIEPMMRDHDGRVVCFRIHDE

LRTLAIQIAEKQENFYCSAGKESPVLNVGDQYSGCTRIFWSHKESLDQSPNASQVRSLSI

SESEDSESEDSESEDSVSEDSVSVSKVIPRGLLGSMSSLKFLDLSWTGVKSLPDTLGKLK

QLVCLRLMGVPIKILPESLTTLVNLEILDLSFSSITELPSGLHMLTSLRYLGLSHCKDLQ

YLPRSISALTSLQYLHMAECNSLWTNAGRHKWKKVASISDLGKLTKVKWLELQYNGETFE

EKMAEVETLRLTLTKIKSLPSSIGEMSKLKRLALKCPDLNKMELSRNKNDYFQNLNCLIL

FNCNMLVRLPDFHKLRNLQRLEIIACRKVKKFEAEAFDAGAFSSLKIFSLVRLEKLEKLP

EINEGAMPSLKIFTIMECPALQKLPEGYLELKTAEKIRIYSCPKVVEEVEKWKERKPNMD

EMVKTMSIKDTEVIKKWHFQLCVKHGSWVYGELWCSELFLFLGGLDTLV

>LL_025258_T.1

MANLTTFSHHGDNASAAAASSSTACSSASTFCSDFEVFINHRGPDVKYTFASHLYRRLLE

RGLRVFLDKSEMEEGHTISSQIEDAIRVASVHVAILSPRYAESSWCLNELVLMIKTGSTI

LPVFYKVHPSVLRWTVEDGAYAKALRHLEQKTT

>LL_025299_T.1

MAASSSTAASTSTSSDLNSNTSFDVFINHHGLDVKKTFASHLYERLHSYGLKVFLDQPES

QRGDYFTSQIKGAIGAASVHVAIFSIRYAESTWCLNELLWMLESKTPIIPVFYHVEPADL

RWTLGKDGVYAQALRKLEEKTEEKTRGEDGVCPQKTQKTLRHKPETVAQWRDALHKVSLI

SGFEVKECNGDEGILLTKVIESVLNNVKKPLDVAKYPIGLDEKRTDFENNVLLPHLQSGT

RKPQ

>LL_025315_T.1

MEQEVVPATYQALINHRGPETKKNFASLIYHRLISCGLRLFLDKDELRTGDIVYPSIRKA

IRSVFIHICMFYEFYAELRLCLAKLCWILESSHERAIIPVFCDVEPHDLLYSDQGHYAVA

FGEHQRKGKVAVAVVKEWKEAFRKPSKISGLVFKTNESEHGECLEEIVKIVLREVKSDPL

EVAKYPVGLDQALEDFQNVLGGLKVLIVLDDVDDGRKIRSLLHLDGLGLGSLILITSRDK

DILKGTSAYPLVYEVKPMKRTVAQELFCQHAFRQSKAFEGY

>LL_025330_T.1

MASASTSTSSSVFPRNYQVFINHRGPDVKKTFVSHLYCRLRSDGLQVFLDQPELERGENI

PEQIEQAIDIASVHVAIFSPRYAQSKWCLDELVLMLKSRATRGATILPVFYGIEPFVLRL

RGKDDEVYAEAMRIHEKKNRYEQQIITDWKRALYEVSLISGFELKAYNGDEGELVDEVAR

QVLKKVRKEPLPVATHPTGLDVKLQDFEVILERRQHREGPNVVGIVGLGGVGKTTLAKYF

FNSKTSDYQQSCFLPDVRDAAARHSLTSLQSKLFRDLTGRDQSIDNVVDGIAKLKDRLSC

RQALVILDDVDDVVQLDALFSPVKDALPSGSVVLLTSRNKDVLRKSARIAESSIYNMTGL

GFEHSQELFCSHAFL

>LL_025413_T.1

MSLNSLQSKLIKDLLHTDKITVDSHNGIELFQRHLKSCHAIIVLDDIDHISQLDAFLPLK

KILSPGSLILVTSRDKHVLISSGVSEQSIYHLKGLDTPNSQELFCFHAFHKPLPPPEFAD

LVDRFVEACDGLPLSLKVFGAQVCGLNEKSLWEEILDELDKTLPTEIYDKLKISYDSLEP

KVKEIFLDIACFFLGEDGDKAMRIWGKLGIQDLQYRCLVELDGENKIQMHDHIRDMGRKI

AEEGSIPLRLWQCTTNSIEDLLEQRTNLINEVRGIKLVFSVNALRYESFQGWGSSQSLRN

>LL_025418_T.1

MRGKGAVVDDWNDCLSLMEKADPSFTETHPRVDEELYQKLRWSYDNLPKHLQNCFLYCAL

YPEDEQISVDELVRMWIAEGFFNSCGATYSMNMKKGHRYVKLLIDRCLFQSVNFQKQSNK

PVKIWKKRFITVHDVIRDMAIHIGEKEENYLCRAGSQLKDFSQRQTQDCKRMSLHLNQID

SLPEDFLSRELVSLIISKNPLAYDVRGSFLMNLNSLRILDLSNTQIESVPTSLTQLRVLD

LSNTQIESVPTFLTQLECLTLRCTCIKELPKDIGNLSNLQVLDISECKYLTSLPAEISKL

TSLKRLHLNMGMENAVNDPANVMALIGLKNLMEFQLFMFFKFETHEIMGTIMGSWIEMRH

LYLHYNHMNYVKCNLPQGMQNMKNLQSFVLFDYRGTNLPNWICEFQQLERLELDSCDNVK

ELPPLERLPSLKFLKLRKYSNVRDLGIGNFGIRSGFPKLEILHLSDMPKLRSIGVLEEGT

LLKLHVLKVKKCPLLKKLPTGMEKLPNLSALYGHRISWWEKIIWEDHNMKICLLKLFKEV

>LL_025450_T.1

MAELTAASHHHDDFASSSTASCSTSTSYVHYDILINHCGVDKDACTFATKLYSQLISRDL

KVFLHQLEYMFKLEEEVTSQMEAAFSLASVHIAIFSPSYAQWKWCLDALVRMTKPGVTIL

PIFYKVEPFQVRWTGKDNHGPYAQWLHDHEEKERYDSHTIQNWRDVLSHVSEISGFELKA

CNGDEEELLSEVVKGVYKDFPCLRYHVFLNHRGPDSKTTLASQLYDRLTSCGLRVFLDKR

ELVEGFKIDGQIEVAIQFASVHIAIFSPKYAQSSWCLDELYLMTKSGAIILPVFYKMKSS

DLDGEYAKDLVNHENLKRYKSDNIQRWRSALSEVAKIKCFFEMDGNDADVLDEIVQSVLK

NVPDRSQVVHSSEVQTVVKGVLKQNVPRNSIVVVESDPEGYFGLAERLIDFENAMLLQQK

ERVKVKVVGVVGAAGIGKTALANLFFTRKRKDYKEYAFLSYVKDKGFTISLDHSHSDLLE

IFKHSPHQSAMLRKHLSFSHALIIVDADKLDDFMPIESVLDPLKSVLDPKSLILVTSRDK

DLLRKSGIVDSSIYHLDGLTRAHSRKLFCFYAFSQLHPEPRFADLVDEFLTACDGWPLWL

KAFGGLLCDKDISFWEKRLSELDNCKLPPEMCRTFGISYDSLNQEEQQIFLDIACFFTGQ

DRDKATRIWGVSGVSSLENKCIVQVDHQNKIKMHNHLRVLGRNIAQGLSPGRLWHQTTNN

IEVLLEQSSFELSRFRGITMIPHVEFRGLSNKTESQMRYHFQHLHRAHDHEVFHGPKNLK

FLAAQDGYVEDILSRVGSPGLIWLHWYNCPYSNLPSEIPMENLRVLEVEGSQLTKLWRQE

SQAPLQLRELNICAPLSVFPESIGQLKHLEKIVVECYARSLETLPEELCHVLSLKHLELR

YCSRMTSLPDSFGELTNLQYIDLEGASSLHMLPNSIGKLSLLKHLCLKDCLDIRDLPGVE

HLTSLEMLDVSGCEKLHNIPGLGQLAKLQTLNASRCRDIQELPGLEHLTSLERLDVSGCE

KLHSIPGLGAARKAANTKC

>LL_025467_T.1

MQPGFSIKSQIAHAVNTATVHVAIFSPRYAESRWCLDELVDMLKSKAPIIPVFYRVTPAQ

VRHAQGSYGEALQTHADNGRHDSNTIDKWRNALEEVANNSGFELKTGSGTSEEWELADRM

VQHLSEMIGMDERRKDFEMIGFDEKVGLDKMVEDFERNFLVNHRQGANPLVVRIVGVGGV

GKTTLAKELFRRKRSYYNKSCFLSDVREYARKGLLTSLQSQLLKSLNPSVEIDIMDERRG

REELRKHISDLSVLIILDDVGDLNQIDALLPVRTVELSSNSLIIITSRDEGRDEGVITRS

GVVTRSRSGVITRSRSGVITRSRSGVGNSPTYKLNGLNYQHSRELFCAHCFSQPRPRRRF

ECLVDKFLVACAGLPLCLKVIGALLYGDFRRPHWKNICNKLISSPDKIEDSIKISYESLT

MEQKYIFLDIACFFIGQKRNTAIVIWNGSGWNGLLGFQNLHNKCLVEVDNENNIHMHDRL

RDFGRAQAATLSPSRLLNCINILEQLSVQPVRGIRMVLGEYINLPSLQLLDTEGVPSLQL

LDTEGDLMERILKNLMSPSLLWLRWNKCPNSSLPSWIRMESLRVLQVSGSELSTLWEDEQ

APLQLRELEIRAPLSDIPWSIWRLDHLEHIVIGKFLSGQVNLTKLREEFCYLRSLKTLVL

TECSIMKSLPDSFGHLRNLRHIDLSFCRNLERMPDSIGFLSLVEHINLSDCHDLKSLPDS

IGLLRRLQHIDLQGCRKLETLPNSFAHLTDLRHINLSGWHHLERLPDCFGNFRLLEHIDL

KECHILKELPQSFGDLNDVQHINLSHCRDLERLPDSFANLRNLKYMDLSGCHNLERLPRY

LENLIHLKYLDVKGCSNLSIGTLDDMPPHLEVAHRLQLEQLLEI

>LL_025500_T.1

MLLQRELLKELFGWDNPLKNVDEGTGMLEKHIKRLRSSNPHDVSNPHDVDDVSNILVILD

DVDHLNQVDALLPVDLPPGSSLLITSRNKHVLLGSNVEDSSIYLLTGLNYQDSQELFCSY

AFSQTYPRPGFESLVDSFLKACEGLPLSLKVFGANLYERDISYWQDELRGLPREIEERLK

TSYNSLDFQEQQIFLDIACFFTGKDRDTALSIWYESGWQGRRGFQNLQDKCLVEVVNKNK

IRMHHHLVDLGRDVAEESSPRRVCRWTEKTIDDLFHPSPGTTVRGIRMVQEGGYNSDTDD

EDDTLVEPQRILKWLRILDTESSLFERIFKVKLQKLTWLCWRRCPHSFLPSWVTMKNLRF

LQVSDSSLNTLGEREGESQALLPLPVCLEELLADGCVQLKSIGGFEHSTKLRHLTVSGCS

ELEELPSMERLACLETLCTERCVKLKHIRGLAQATKLRVLHVSRCSALEELPRMESWVSL

EFLLADYCLRLKTISGFGQSTKLRTLHLNGCSALEELPSMETLVSLVELRAEGCVKLKNI

RGLEQATKLRHLHVIGCSQLEELPSMETMVSLEELCAAGCGKLKNVQGLAQARKLQFLDV

SGWSELEDLPSMETLVCLEVLQAEGCVKLKNIRGLEQATKLRHLHVIGCSQLEELPSMET

MVSLERLCAARCGKLKSMGGLGQLTKLQRVNVNGCCELEELPSMETLVCLETLSAEGCVL

LKSIRGLAQCTNLINLNVGGWLELEELPSMETLVSLVELRAEGCVKLKNVRGLQQASKLR

HLRVIGCSQLEELPSMETMVSLEELCAAGCGKLKSIGVLGQLTKLQKVDVDGCCELQELP

SMETLVSLVELRAEGCVKLKNIRGLEQATKLRHLHVIGCSQLEELPSVETMVSLEGLCAA

GCGKLKSIQGLAQATKLRFLNVSGCFELEELPSMETLVCLENLRAKGCVQFKSITGLVQC

TKLRFLNVTGCSELEELPSMETLVSLEELRVQGCVRLKRIRGLGRLTKLQHVNVMSCYEL

QELEGVDEHCTWLDKLKTFSNLKLQCSVWQ

>LL_025593_T.1

MAASASTSTLADPVDTYSCDVFINHRGPDSKKTLASHLYRRLLSHGVRAFLDQEELQVGV

NFNSQIKEAIRTASIHVAIFSPRYAESKWCLDELVLMLESGAPIIPVFYHVKPAELRWTG

VKDRGYAQALQKLEMKKTYNSETHQENPRYDSTTIENWRNALSSVADISGLDLEAFNGDE

GELLDKVVERVMKKGRKTGLYVAKYPIGLDDTVKDLETTVPLQQHSGRVQIVGITGLGGV

GKTTLAKELFNRKSSNYSKSYFLFDVRENAKISLQSKLLKAITQMDLHVDSIDHGIEMLR

KHLSSSQVFVVLDDVDHVDQAESLLPVDVLRPGSLVLITSRDKTVLARSGVQESSIYKLS

GLNIVRSRELFC

>LL_025612_T.1

MITRLISDIRGHLELSPLVQLAQIPELAEELKQTIKESVEAAFASSSSSSSATTSHPLAS

GGVYISEPLIVGQDKAFARLEKLVTDAECSRIGVIGMGGSGKTLLFRRVFNSKVVQNIFS

DGLLLWLTVSHSPSFTSLRNDLCSQISMQTKAD

>LL_025630_T.1

MVFLLDPVTGAVVEKALSALMGAAKIIITSKKCGKELDEMLSWLKPIIDDLLKLEIPDSD

SSSHVVMQFKDLLKAGLEVVEKIARTSWFDLINRFRYGRRIQKFLKKFTKFIEINGSPGA

KLDMAKLHADLKVLDADLRASFQRLVEIVNAIRMTNNPVINNAILQQMINANTIQVVNTQ

VVTTHVVNNISVVETHTATSSQESIGSSYQGTEISKQAVDLNNRINDVKQILLQNDVKVV

GVVGMGGIGKTTLASTLCNEVKGFFQNIIFIPVSSSPNVKALLDTMWNKITGRLENPGFP

SIEYAHSQVQRALSSKTDRQILMVLDDVWSRSDLQHLLFEAEGYKTIFTSRENNIIPMSR

DGPYREYPMPMLNVEDSLKLFCFWAFGQPSITTTEHKDLVCQVAAECNGLPLALTVIGSC

LRDRPRVVWRSAMTKLSRAEPINSDHTKQLLDRLKLSIDVLDDEESKQCFYDLAAFPKGK

KFSVDALLDIWVYVRGIEWCDAFVNFLELAKRNLLNFTSDPRSQAISDYDCASGLCFSQH

DVMRDLAFHLASQDSTKPCSRFFMPGKEKNIPADWISQKEKTSKAQFVSIHTDAMEEQDW

GQIDFPEVEALALFFGAREYCLPTFLKTMPKLKVAIIHNYSSKRAILHGLPSFPLFNQIK

SVVVEGLNVSPFYEYCRTWESLEKFSVCLCEGLVNMPLWDRVGIWEKSHPSKMTVSTIYT

HILQVALFFMMVSKKCKQYLTGKEQVLKFPNIVEINFDHCSDLEELPVKICNSTNLQKLS

VTNCHLFQKLPDDLGNLRSLRVLRLSACPGLSMLPPSIRQLQQLEFLDISLCRSLKDLPM

EFEQLSKLKVLDMRECSGLKMLPKALAKLKSLGRVICDENAGRQWLAIKASAMPNLTVEV

VEECFNLEWLDY

>LL_025645_T.1

MAELTDAPRHDDSASSPTASELTSDPGYDDFDSGHDDFDPGHDDFASSFTASGSASTPYV

HSDVFINLHDYNDCGRDVMTFATRLCNRLKSHEFQVVLDKPNFLYEEDNIPSKIQDSIFA

APVHIAIIPPNYVHSKWCLDELVLMIKTSAMLRTTIYPVFYSVKPNELRYDGAYAKALST

YEQLYGSRTIQDWKDALFHIANISGFVLDEGPGGEEELLANVVKSVRNDFPHGPRYDVFL

NYRGIDHVKDNFAKSLYDGLVSCGLRVFLDKGELVPGFDISQQIEVAIQLAHVNIAIFSP

KYAESSWCLRELVLMGRSRATILPVFCQMRRSDLLDGVLYAEALLNHEKKKRYDSTTIKD

WRDALSKVAQLPGYELGGDEKELQHKIEQSVLDSLAKRSADVRNSEVELQRVLKIVLKED

VPKRVSQEVEDEKELQPEVELQRVLISALKEDVPKRVSQEKEDVPKRVSQEKEVPK

>LL_025652_T.1

MGVYGKDGIGKTSLLKLIHNHYKKVSCIKFDLVIWFLVCSSECEENTNKDSIVRGSSAID

MRKMKLYACLEKKKVLLILDDMWSPMDLNTMGVKNFKLSFMSKKGRDFF

>LL_025664_T.1

MGVAHKYIITMENLFKDESWELFSYHAFPYNNRRIPSNVNEEKAKMVCQKCGGLPLAIKV

VGKAMAGCTFPQEWKLAARRLPIDHSLDACLRLSYDALGKEDIYLQLCFLYIAAAFSEDQ

IIHANQVIPIWAGEGMLTRKTDHQVSHSSFPKGSVYLHLLADRCLIEPTLRSMNGRVVFF

KMHDVLRDLGIRIAEAEGTFYCRVGQGLRTLNENECSGHSRILFSRNELRSLPGSLRAPE

LCSLLMDGNKDLTKIPKKVMGSMFSLKVLDLSGTSVQSLPESVGSLKQLACLLLSRTPIK

LLPVSFTNLVNLEILDLSRSSITELPTGLHNLKTLRHLAMDKCVHLHYLPCSFSRLTTLR

VLSMYGCATVWEKGEQKGWKKVSTMASINNLASLNNLSRLHLTNNGDVITEGTLGNMIEM

HTLMLQLTNMKFLPSDMNKMSRLRRLYLECPDLVKVERNFCDFQSMTTLTLYKCDMLEEL

PHLHKLQSLKRLEIIECSRLKKVPQEFGEEGAFSLLEMFSLVGLHELEELPMVKGGAMPL

LKIFIIVECSALKIFPKSYFNLKTLQVIKVYGCSSMIMENLEEIQESNTMIKVKTMSIED

TRDARKRYSQVGGEIKSWLYGEFWNNEIFIFLRSIYTLE

>LL_025685_T.1

MASPSNTASAASTPHGIGNNYPKYDVFINHPAPDIQYNIAGQVYSRLNTHNLSVFLDRQA

LQTGVNLPSKAASVHIAIISPGYAVSAWCLRQLVQMLESGKTIIPSFYKVCRAELWWPDG

VYAQALHKHERQDQYDSHTIEGWRKALSAVAKINGVEMLLYNSDKELLVDKVVEAVVKMF

GISLLASRAYELKGADSDTIALLHRKGANGPHIEMALQQPPVYPMASAYGIGSNVSPFDV

SINHRGLDVKRGLASLLYKRLSAHGLRVFMDGEVLQTGVNLASQNEGAIKPASVHIAIIS

LGYAESTWCLRELVKLLKSGKRIVPVFYKVYPSELRQTDGVYAEALRKHERETRYDSHTI

EGWRTALSALAHRRGFELTAYNGDEGLLVDKVVEGVVKTFGIPLPASQAFRQDFTVTSKG

CKWSSY

>LL_025708_T.1

MASSSTSASTSHGNCVFDVFLNHRGPDVKKTLASHIYHGLTQRGLTVFFDIEEMQEREAL

TPQIINAIRTASVHVAIFSKGYASSHWCLDELVEMVKTKKPIIPIFYDVNPSELRWTQAE

NGRYVRDLHMLERKTTWDPQTNKEAQRYGSGTIENWRKALSSAAEISGFELNKFNGDEGL

LVKEVIQRVLKTVPKKLLNVAMYLTGLDVKVKELEAIVSSQQQSRSLARAVGIAGLGGVG

KTTLARQFFNSYISNFERHCFLSDVREKAARNSLISLQAQLLKELAEQNKQVNSLSEGAE

MLKRYLPSSCRALICIDD

>LL_025791_T.1

MAASTSAPLDTNYNVFINYLGADVGKTFVSYLYCRLLSLGMRVFPDPHELQEGEADYVIS

QREGIIQTASLHIAIFSGRYAESKCCLNELVLMLESGTLIFPVFYHVSPADLRGTHGKYG

LNLEMKKTHDSTTIEKWRNALSRVSEISGFDLEVFNGDEGMLLERVVDDVLKSMKTHLNV

>LL_025827_T.1

MAASSSTAASTSTSSDLNSNTSFDVFINHHGLDVKKTFASHLYHRLHSYGLKVFLDQPES

QRGDYFTSQIKGAIGAASVHVAIFSVSYAESTWCLNELLWMLKSKAPIIPVFYHVEPADL

RWTLGKDGVYAQALRKLEEKTEEKTRGEDGLYPQKTQKTLRHKPETVAQWRDALHKVSLI

CGFEVKECNGDEGILLTKVIESVLNNVKKPLDVAKYPVGLDEKRTDFENNVLLPHLQSGT

R

>LL_025900_T.1

MVDPGFVPGLIGVGIQITIGEILRHINIAVNCRKELTALKDTLMRIEPFIKEIRLALNKH

KDEAPTVDNWFEELDALLKHASEIVQLCIPRWGIVSRYQTCRKITDLTQKIDKLLTRSDL

VLMVQLVQVQKLTRKILEGQCQIIENIETLASSSSSASTSQAVHAPIGTINSKKEDKTVT

VTASRPSNLSKVSSFQDEQLDSHGFVSHDVEEEEDPTENQRFGKALDDFPADCDDELNLT

AGEDVEILGEQDKWLYVRKKQPGTDSKVAGWVPMSFVKSHSSFPLATTRYLQEDKRVTGT

ASGLSNLSKFSPFQDQHLDFHGLVSHDVEEEVLGENQRFGKALYDFPAGSEDELNLTAGE

EVEILGQQTEWFYVSKKQPGRDGKEAGWVPVFYLSPSYSSF

>LL_025927_T.1

MDLLNTNRIVNLIEQIDDVIQLDDHGLLESQLNRMKSFLLSIRTQLSYGQTAPIETFIKG

FERMENEFRSARILIVKVSDQRDSWQRRFYRPLSNRNVDEFYKKLREDIELKELKEFIRP

VILLIGEILDQQDSWPFYCIPCNREVFEFYKELKQLKELMQFEKLKEHTKLNELTELEEN

KLLQDLKELKEIKQFEKPKEHTELIDHTELTELEELKLLNELQKLKRLEKYTERKELTEL

KKLIQLIQLKEIKKHTEHTELIQLIIKWKIYVDEIYKELKNHTELKLIQIIIKWKKYVDE

LYKELKKLSEHTELTEPVQTGPKNGEVVVESWLREAPHLRIIGVYGGAGVGKTRLLQNVY

KSYKVSDFFDVVIWVTIGQSGILELQDCIAQAVNLDLGAQHSIDLDMRKIKLSEYFRAKK

FLVIFDDISVPLNLKKLGVEFGDDKGSKVICSSRTKDVIEKTWAKCAIYIEPLTRGEAWE

LFCNVAFKDGHLPQDSQHIARKVADECRGLPLAIHVVAATMRGNNSVNDWNLALRQMQTT

VFDHKFPTSNLSIDDHLYQILRWSYERLPDANFKSCFLYCAMFPRDHEIEVDKMVGMWIA

ESLVDSMDTAHSYLKLLVDRCFFQVTRIPHGKVFIKTCNPLRDVAIYIGNREEHCLFMAG

GQVQNFLFIADRQVENFPGEIERNDWKRITFYGNNIKCIPPQRLNCRNLVSLILGANKEL

EEVSMSFLEQLPSIRVLDLSRTNINILPITLWSRKHLQYLDLSYTQIEYLSEYIANLCEL

QFLNLSGCSKLESLPEAIGHLSQLQFLNLSGCSRLTSIPSVIYELKSLKHLYISQYKVLL

>LL_025990_T.1

MASGASSSSPMACFASTSYAQTYDVFINHRGPDVKEGFATRLYHTLHSSYGLQVFLDKQE

MQVGHKILSQIEAAIGKASVHVAIFSPTYAESEWCLDELLLMKKSGATILPVFYKVKPSE

LRRTGKDKDGTYAKDLRKHEEKQRRGSQTIQDWRDALSEVANISGCELEADEEELLDKVV

QSVLKTIPKTPLDVAKYPTGLEAKLQDFENRVLRQHEKEPLEVKVVGIVGLGGVGKTTLS

TDFFNKKRSDYKGSSFLFDVREHSARAKLNSLQTKLIKDLKFRDVNIESIVEGKGILERN

LSSCHALIILDDVGDLDQLDAFLPILRRVLDPKSFILVTSRNKHVLVSAKIPETSIYQLT

GLNRLHSKELFCKYAFFEPDPLRGFESLVDRFVTASCGLPLSLIVFGALVCGEKEPYWEE

QLDGLHGQLPEKIEKSLKISYNSLNSEEKQIFLDIACFSIGEDRDKWIRIWGGSRWKGLV

GFRNLQNKCLVEIGSENEIKMHDHLRDLGRNIAEGLIPLRLWGSTTQDINDLLEQSCDGV

RQVRGIRMAPRSTLADTNCEDQLFLSKRRSGDELFGISKLQLLEAEGDFVESILTWVRPP

NLIWLSWNKCPCSSLPSSLPMRDLRVLEVNGNELKGLWQSQSKAPMQLRELNIKALSVEF

PKSLGQLNLLEKFVAEETNLGTLPKEFCYLPHLKYLKLKDYKMQILPNSAEKVTNLQLSQ

RRGLQNLANLQISDLQQCRSLEMFLTVVAWRRSQTPSRN

>LL_026093_T.1

MEVVVVKLLAEKLSSVLFAKLKKQVWLVVKFKEEIESIYYELAQINLMLNDVGDTRNSQL

KAHWLQNLRYVLTEGIDLLEESEEADNDFKEADNVFISFFNNWLMGQKFFFLRRMDKKIQ

VLKKRISDINDNGNKYVRYLKSLVDENAISAEDGRKKSSPHLDEKDQTVGIQEDIEVLAD

EILKENGSRVIAIVGMGGRGKTNLVKHVFNTQKVKDGFENRVWLYVSQKVDIEHLLQHIA

HQIHLPGDEIPIHIPGFEYLLHHIGSQIRLPEDETTKYCSQVRHRSPSIELPKEYHHRLN

EVSMDRLRDNIYTHLKAEKKPSIFVFDDLWHKDVLKRIGLRLLSHNSKNKIVVTTRDKGI

SNALGAEYTYHMKDLSEKESMELLCIHAFSKRKLPKDLESCGKDILNKCTRLPMAVEAMA

TQLNGVDKIPNHWKAALGRVNTEDVIKEKVMPILRPSYNEMPPLLKACFLSFASFPPNTP

IHVEWLVYSWLATFIEIVSAGGDAVDVARSYIDQLVDLCVIQALHVSADGRVKSCQMQEF

FHGLAFSESENVAKCLLKPKKDLPYLPVEDCKDARRISLVKNEIIKITETIQCPKLRILL

LFENVNLQSISKTFFKELRYLVVLDLSQTGIKSLPESIQYLKRLRFLNLSQ

>LL_026116_T.1

MAYPTPSCSSFPQTHSQIEMENQTSRPATSVGASSSSTAADPNILYDVFISHRGEVKKTL

ASHVYRGLLSRGLRPFLDQQELQEGLHFPSQLKAAIRSAWVHVAIFSPKYAESQWCLDEL

LLMQESGAPIIPIFYHVKPAELRW

>LL_026228_T.1

MRTIDESFYDIHDEIEKKLFQRLKWSYDVIPKYLKTCFVYFAAYNEDRIINCEEVIEMWI

AEGLEGLVKSGGQSYLHDTAYSFISFLRDRCLIEATKTDIVGRIEQVKIHDVLRDLATLL

AQKEHKCFFKAGQNLKDFPVEEDSKGWARMSLMCNPIRTLPTTFACSSVLVLTLRQNPGL

GVVPGSFLQGMSSLRVLDLSDTHITSLPPSVGDLKHLSSLQLFRTDIRELPESIGDLNKL

QFLNVGFCGQLQSLPKRITELKCLTALDIWGCRKLSHLPRGISELFSLERLRMKMSIPLA

FEEAPDTNAERSYACLKDLHSLRRLRNFAVEIKSPVKDGVMGNWSNMRDLWLDFNLANQD

NLPQDLKEMKDQLERFCLCRCDVELLPSWIGEFRKLSYLLLKSCKRLKELPAELHELPGL

RGLSIEDCDALKEMELGRRGCFPKLEKLTLQNLKSLECLQGATSSSSSSGLGEGALPMLK

SFTVKKCEKLKALPLGWDKLKCLEEIRGERDWWDAIQWQDVNLKTSLESKFRSL

>LL_026314_T.1

MNKSFLFLLDDMWAEDMWTCDKLQNLGVPLNGTGFKLVLTTRDQPVCATMNVQQRIRVEP

LLEDDGWELFRSRAFQNGNVPEQLEEVARKIAKECKGLPLAIIVVAAAMTNHSYLYEWEL

ALNQLQNVDKTFYDLHPGVDTELFQRVKWSYNVLPDYLQACFLYFAAYPEDRVIFCRKVI

HMWIADGLLKSSGDSYLEDIGHSYISHLRDRCLIEMVSTDSVGRISHVKIHDVLRDLAIF

IAETEPYRCYFKAGQRVSHFPVEESWARMSLIYNHLHSLPTSFACSSLSVLLLSENPDIT

EVPGSFLSELPSLKVLDLSRTRITSLPPCIGNLKHLAFLQLEGCGGICELPETICDLNNL

QFLNVSWCTQLRCLPERISELKRLRVLDIR

>LL_026353_T.1

MADPQTSTAYSSVASASTSTNYEVFISHRGPDVKKTFAGHLYYRLRSHGLQPFLDKEELR

AGEVGSLQIERAIRTASVHIVIFSPGYADSRWCLDELVLIMNKSGDTKIPVFYNVKPSEL

RWTGRYVEALSNHEQNGRYDRQTLEKWRKALYDISYIIGFELEAHNGDEGKLLDIVVQWV

FKKGRKISLNVATYPTDPQEKVEESETTVSTPQRQNRKDKVPRIVGMGGLGKLTTLAKKL

FKRPSSNVGTLISQDRQIQQIEMFKREILVAATENFHDNNKIGEGGFGRVYKGTTQDGRQ

IAVKKLSLYSKQGKEEFQNEVNVLPKIQHRNIVNILGYYSDESEIMLIYEYLPKSLDKIL

FDPNKRVQLDWKKRHNIIVGIVRGLLYLHEDSQLRIIHRDIKPSNILLDQKLNPKIADFG

LAKVLSDETHNGAEVADIYEGEIHITSQDDTYSTTGIVGTVGYAAPEYLIHGNLSVKADV

YSFGVLLLELVTGRKNTDFNLPIERQILLEWAWRSYTEQNIHLMIDPSIIETCDMEEASR

CIHVGLLCTQYDSSLRPPMSTAIFMLSSRSAILPEPIVASFVGSYSRCVSTSSERIYSSS

SGDFNINPSYN

>LL_026366_T.1

MRENARTKSLAYLQNKLLEDLTQIKDKYIEIESRDEGIGILKRKLSSRHVLIVLDDIDHM

EQLEAVLPIKKILRPGSLILVTSRDKNLLTASGVLESSIYIMKRMNTPHSKELFCSYAFQ

FHEPNPLQEFADLVDRFVKACDGLPLSLKVFGALVSGENERSAWLEILDELDNIMPIEIH

DRLKISYNSLSSKVKEVFLDIACFFLGEDGDKAIRIWGKREVQNLQNKCLMELDKGNKIK

MHDHIRDMGRKIADEGSMPRRLCYQMTNFNDDLFQQSSPVVTEVRGIKMLTCPYDLFRDI

NASNWQYRYMFRSPKLSPYNSLEMRHPLAMRNLRLVSTDLSDLEGILSRVQSPHLIWLRW

DGCSHTSLPHWIPMQNLRVLEVGGWRLNTLWPHSQAAPSDLRELNICAPLSDIPKSIREL

KNLERIVISHSDRQASITLKRLPDEFCHLKSLKYLVFNGFSRMVSLPDSFGELINLEHVD

LEGATSLQMLPHSFGNLSRLKYLCLSGCEKLTISNGTLRDISMLEYLDLSSCKQLKELPP

QVSHQRSLQTLYLWGTKLKELPSLIGDFCNLEVLKLGSNLLEVLPLRLGDIKSLKILSIF

DSPQLKSLPDSIGLLNQLSRLEIVCCGIEYLPQQLLKMNSLEVLEVSGCPLRGHPFKKEV

EGERKQLSSLERE

>LL_026379_T.1

MLESGAPIIPVFYHVRPNELRREDGKYAEDLRNLENMKTHDNQEKPVYNSTTIKKWKDAL

SRVSFITGLDLLDTCNGDEGKLVEMIVQSVLKNVKMPGLENSCLVNLDTENDTHMLDRHR

DMGIISKAPKSDISQKDNDDSLQQSNCLYNKWRILFGWSANWWALFGGSLKEQVTTSVRK

SICGYM

>LL_026509_T.1

MAMPNFNTAAASNSGADYDVFISHRGPDVKNTFASHLYRRLCHCGLTAFLDKEELQKGDT

ISSQIELAISSASVQIAILSPNFAESSWCLNELVLMKNSGATILPVFFKVDPSEVRYTEK

HGVYFEAMLKLEGKTTSDPNKPRYASETIQKWRAALSYVANLVGFVHSDRDDEGELLDNV

VERVLKEIPKTPLDVAKYPTGLDNKLEYFKKTVLYQQQQESERLETKVIGIVGIGGVGKT

TLAKSFFNSQRSSFKESSFLSD

>LL_026615_T.1

MANFHGSVAATSSSTASSSASTSCSYFDVFINHRGPDVKHTFASHLYRQLLNRGLRVFLD

KSEMEPGHNISSQIQQAISGASVHVAIFSPSYAQSNWCLDELVLMKNSGATLLPVFYKVH

PYVLRWAVKGVGAYAEALGELEQKTTYDSETGKEMARYGPDTVQKWRDALSYAADLTGFL

LPGDKDDKRKPLDNVVEHDEGELLDLLVKSVLKNVTKPQLDVAKYPTGLELKLQAFEKTV

FTQHLAERELVEAKVVGIVGVGGVGKTTLAKEFFNRRRSDYNSSSFLFDVRENARTMSLA

SLQKKLLEDLTQIKDTKISSCDEGIGILKRNLSSCHVLIVLDDVDNIDQLESILPIKKIL

DPNSLILVTSRDKHVLKASEVLKSSIYILEGMNTQHSKELFCSYAFRQPDPLPGFADLVD

QFVTASGGLPLSLKVFGAQVCGEDERSFWQEILDKLDKITGEIHDRLKISYDSLRPDVKE

IFLDIACFFLGEDGDKAMRIWGKPEIRNLEDRCLVEMDDENKIKMHDHIRDMGRK

>LL_026687_T.1

MVDEFLEFCSGLPLFLKVFGALLSDKYKQSEWQEVLNRLQKEFPSEIEKSLQISYDSLNT

EQREMFLDCACFFRGLKRDTAIRIWYGSGWEALLGFQTLQNRCLLEVDIENNIQMHDHLR

DFGRALGVTMLPLRLWHCTDIKDMLKPFTVPSVRGIRMVLGDDVDAFGDIDMSNLQLVDT

ENAMLERILRRLMHFPSLLWLRWNKCPNSSLPSWIPMESLRVLQVSGSVLRTLWEDESQA

PLQLQELEISAPLRNIPKSIGRLKHLEWIAVSRFLSGGRVNFTELPKEFCELQSLKTLVL

TEC

>LL_026735_T.1

MAEAAVVSTLMQTLTSMLADKLLQEVSLATSFKNDFEFICDELVSIKMLLNDAGKKRNTR

SMSNWLDKLEDFLYDALDMVKECGVVRRFGNPILRYRMGCKIRGLKDRIIKIHKSAKYLK

HLTCVLDLNAFNEENAEDKRERSSAFLKEIQHVGIDNGIEQITDLILSEEDCHQVIAVLG

MGGMGKTFLVQHVFKSQKVEQCFDYMVWLALSPSFMVKQLLVEMCRQIKLPVPSDEIQGS

GAEDLVTEIHEYLKEARCLFVLDDVWERDVWSKIGLPFQNKYKIVITARHKKVVESLPRY

HIHYMVELSYENSMKLFCIHAFPDREENSPPEELTSLAQEIVKKCSGLPLAVKTIGASMT

RVRRIPNDWESTLNRLNEAEAMNGRVMPSLRLSYEDLPYHLKLCFLYCS

>LL_026768_T.1

MAASSSTAASTSTSSFDVFINHRGCDVKKNFASHLYQRLRSYGLKVFLDQEELQRGDSFS

SQIRGAIRAASVHVAIFSVSYAESTWCLNELLWMSESEAPIIPVFYHVEPAELRWTLGKD

GVYAQALHKLEEKTTDDPQTQTQGEMSPHEATSSIINWRNALSIQHWRNPLKKYDPQAHQ

KTLRHNPATVAQWRKALHTVSLISGFELKECNGDEGILLTKVIESVLKNVKKPLDVAKYP

TGLAEKITDFENTVLLPHLQSGRKPQIVGIVGLGGAGKTTLAKEFFNRKSSEYNKCCFLA

DVRENVNNACLISLQSHLLMKLTGTDLQIHNK

>LL_026780_T.1

MEQVSEILPVAATYHVFINHRGPDVKRTLASLIYNRLCVFLDEKEIRTGDILSPAIIGAI

KLASVHVAIFSRHYAESSWCLEELHLILRSYQEGKAKIVPVFFGVEPKDLRHIESGLYAE

AFEEHQRKGRVAMEVIENWKTALNQASMIRGELFKMDESVDGDIFENIVDAVLKEVFTRE

AIEVAKYPVGLEQAAEDFQKEILKHRDPNATTIVGITGLGGIGKSTLAKHLYNLRDPKFK

RSSICEVRQMGLRSLQKKLLRDLLGNSQHIAEEAPTYFK

>LL_026788_T.1

MAESSSSISTSTHDVFISHRGPDVKKTFASHLYHRLISFGLEVFLDQEELQRGGSINSQI

EKAIRAASVHVVIFSENYAGSTWCLNELVWMVDSKAPIIPVFYRVKPSELRWTEDKDNGV

YAQDLHRLKKKMNYDPQTQQETPRHDSATIKQWRNALNIVGGVSGFELAACNGDEGILLE

KVVESVLRDVKKPLHVAKHPTGLEEKVTDLENTVLLQHQQNGRKPQVVGIVGLGGAGKTT

LAKELFNRKRSQYSRCCFLSDVRDTVGKGSLISLQSSLLTKLTGSDLKINNKYEGIKMLE

KHLSSNQILLILDDVDTVDQVDALVPNQNSIHSNSLILITSRDSGVLTRSGVKQSLIYKL

NGLRKRYSLELFCYHAFNRPYPLPGFEDLTEKFVNACDGLPLSLKVIGALLCGNKDMSYW

HKQLRKLEKILPSEIQERLRISYESLEREEKQIFLDIACFFIGKNKDTAIRIWNGSDWEG

WLGFQTLQNKCLVELENRYL

>LL_026802_T.1

MAASSSTAASIFTAEPNISYPACDVFINCGVDVKKTFARSLYYRLRENELTAFLDIEELQ

PREEILPRTKAAIRSASLHVAIFSPRYAESQWCLDELILMLETKLDKPIIPVFYHVKPSD

LLWTLGKHGNYAQALQRLEQETTDEGNLRYDSGIIERWKKALHYVSDISGFELEACYGGE

GKLLDKLEQCLLKLVENKKYPTGLDEKVKDFEETVQHSTKPEPIQEKRYDGEDRWENLSI

HYDPLSEEEKHIFLDIACFFIGKNRDMAIRIWDCSGWKGRLGFENLQNKCLVEVDSWNMI

QMPYRLRDFGRAVAEEATSQRRLWRWTENVSDDLVQKLSEITVRGIRKVLSEDNDYDEFS

AIGNMSLQLPDTQHSHLDRIWRRVKFSNLIWLRWRECPYVSLPSWIPMKNLRVLHVSGNS

LEILWQDGSEAPSQLEELWADGCMQLKSVRGLSKCTELRKLIVNKCWELEELPSVETLVS

LEELRVDECLMLKSIKSLAKCTKLRYLKVSGCSELKRLPTMESLVSLEEFCASECVMVKS

SIRGLEHCIKLRHLDVSGCLELEALPIMKTLKSLEELWANRCVKLKSITGLEHATKLRLL

NVSDCFELQELPNMEKLECLEELWADNCVKLKSIRGLEQATKLRILNVSDCSALKELPIM

RQLLSLKILRATGCVKLNNIKGLEQCTELSIVSVSRCCKLEELPSMERLAYLNEFYASEC

VKLKSIKGLEKLTKLRHLNVSNCPDLKELPSMERLKSL

>LL_026816_T.1

MLHKVIERVLKIMATKTRLHVAKFPTGLDDKVKDFETVLRQQPTGKAQVVGIVGCGGVGK

TTLAKEFFNRKHSDYSRSYFLSDVRENAKISLHSLQSELLKGLGQLDGHVNNIHQGTQML

LEFLSSAKVLLVLDDIDHQNQIDALLPNRDALLPNRDALHKDSLIVITTRDKDVLAGSRV

AEPSIYKLTGLTAQHSRELFCSYAFCYPHPLPGFVDVVNEFVDASDGLPLSLKVFGALLF

RKEKYYWEDTLDRLHQVHELPSDIKDTLKISYDALNGEDKQIFLDIACFLIGKKRNIGIG

LWNESRWKSGFQNLESKCLVELDSDGDIHMHDHLRDLGRQIGEEELPRRLWRPTENIDDW

LQQQSSVSVQSFKHLKY

>LL_026856_T.1

MASTSSAASSTSTAATSDYDVFINHRGPLVKKTLASHLYRRLLSHGVIAFLDQHELQRGD

DLTSQIVGVIGSASVHVAILSEGYAESTWCLNELLLMLESKAPVIPVFYGVKPHELRSGQ

AIQRREKKMKHDPQTEEEKRRHDSATIENWRKALSLVAGISGFELEACNGDEEELVDMVV

REVLRKVKKPALNVAEYPTGLDDKVKDFENTVLLQLQESGKPRMLGIVGLGGIGKTTLAK

EFFNRKSSDYSKTCFLADVRDTASKRDLILLQKKLLRSLNASDNIDGVDEGIGMLSKHLS

SSRALIILDDVDHWNQVDALLPHQSVHSDSLVVITSRDKDVLTRSKVENTSIYKLTGLDT

QQSQELFCFYAFNQLYPQPGFESLVLEFLKRCNGMPLSLKVFGALLYGKDISEWEDELES

LRQILPKDIHHSLMISYNSLNKDEQQIFLDIACFFTGENMDRAIRIWDGSGWQGRRGFET

LRNKCLVEVDSENNIQIQDQLRDMGRYIAEEPGLPR

>LL_026902_T.1

MAHLQTSTTHLSNDTPSTSTSYPVYDVFINHRGPDVKNSFASHLYRELYVHGLQPFLDRE

ELRAGELLSPQIEAAIRTAYIHIAIFSPGYADSTWCLDELLLILKKSRDTIIPVLYNVKP

SEVRWVSKERQYAQALKKLEDKGRYDRQTLENWRKAFNDVSYRVGFELDAYNGDERKLLD

NVLQCALKKGRKIGLNVARYPTGLEDKV

>LL_026915_T.1

MASTSSASASASAFGSPSPSTSSASASASAFGSTSSASASGSAFASPAPYPSANSNHDVF

INHRGPDTKKTFASHLYHRLLSHGITAFLDQNELQQGEGLTCQIENAIHSASVHVAIFSS

KYAESAWCLRELVLMQESKSPIIPVFYHVKPADLRWTKGTYAQDLEKLKNSQRYDAQDLE

KWTKALSNVPETSGFELEAYNGDEGELLNKVVERVLKKVRKPPLDVGKYPTGLDDKVNDL

ENTVLLHPQHSGEAQILGIVGLGGVGKTTLAKQLFNKKCSNYSKTCFLPNVRDHAGKGSL

ISLQRTLLKILTGSDVNSINSVINSVDEGIGMLRKHLSCANALIIIDDVDHEDQVNALWP

HQTVLHSKILILITSRNMDVVRRRVENTSIYKLTGLKTQHSRELFCCHAFSQSYPLPGFE

SLVEKFLKTCNGLPLSLKVFGSLLYGKYNDKSYWQYVLDKLKVPSEIQNKLKISYDSLTE

GEQQIFLDIACFFIGEDRDMASRIWESGLCGFQNLQDKCLVEVNSENKIEMHDHLRDLGR

DIAKEPGLPRRLWRWTDEDIDDLTLQQSSLSAIPVRGIKLLQKEYNEFADDDALGGIRMT

RLQLLDTEGVLLERILTKQSPNLLWLRWIECAHSCLP

>LL_026918_T.1

MEMFNNCKQSCSRLVALLANLKHKVLTLCATNIEKAQNEQNPIYARCLSILNKGDQLVKK

YEKTSRFNIDQNLKYASEIDQLEKDLSDFVQSQVEAQLSLELKNLISEFKSLRHLYELGS

VDERKANETIVPQLTNDPNENAMMLQHMGPDSMFDGAFDEAPASTYNGSGKSDFVAGLDK

NIFNLKRILLQREVSVIGVQGMGGIGKTTMATALCNDQEIKGAFGNNIIFITVSQSPSLK

GILEAMWEKIVRRKKPEFQNVEDAHRQLQQQLLRQAQRTLVVLDDVWSRANLEKLLFKGE

GYKTLVTTRDRSTVPTTTSTRLYDLPLLDDANALPLFCFWAFGQRSIPSNADEQLVKQVQ

AECKGLPLALKVIGSSLHGQPRPAWDSAKNKLLNEESISDYHREGLLRCLETSIGALDEE

ARECFLDLGSFPEDRKISVDALLDIWVYVRKMEWNDAFAILLELASRNLLNLTSNLSGSK

AINYGSASELYFSQHDVIRDLAIYLSSQGSLVHRKRLLLPKQE

>LL_026926_T.1

MTTPPKGYLMRGISSAYAKSLLEEQLPKSLLANEMPSNKEGILNSNQFNRIIDKCGGIPL

MLNLVVEELSSSKHEKEVCDVIEELEKLEGEEFGVKVEYCFFIYDKLPKECKETFLDICA

FFQGWDWDLVADMVGKAALERLERRALVAKGPNGIVTVHAVILEVGRRMAKAMRIRFLER

SELQNFLQEDEKLVEELRYLRYKP

>LL_026945_T.1

MANVPRVSGNAIISLVLDPVMDMMKDLIQKEENGQILETDLRPMESLLQYTGIIFRDQLT

DPPEMVKIWLQLLQNSLTEANGLFHRSRPRLQSCLACVVCNPGRLSKEIREWNALFNHLY

HGFQSDLFLHIALSSNAMISVAVDPVMELLKDVIEKEDNRQILQIDLRPMESLLRDFTTL

FQPQKEALLEIMIKAWLPLLLNSLIKAKGLLDSSAPRQSFLDSVSKEIREWSASFERYLF

QHIVLSGNAIIRHVVDPVMELLKDVIKEEDNRQILQTDLSRIETLLLDITTLFRDEPDIT

TLFQSQNKAFLEIMKTWLPLLLNSLTEAKRCLLIDRVVAEASFDKIYHGFQSDLSVLVTS

LQFLSSNPLHIQEKAKEPTVIQPLSTDEGWMLFRRVVFEDDKVSMDIEDRARKIAEQCRG

VSLAIHVVAAAMIGQTSLHEWDICLSSIQNVDPSFPATASGLDAELYAALRFSYDALPNS

DVKKCFLYCALYNEEQEIDTLELVQMWIGEGVVKSKQGTYLMDMAVGRKYLKLLVDRCLF

QIVKERKNVFKEKTIRVESLIRAMAIYIGQKEENCLFMATQNQRNFPEVPADANCRRISV

ASNFMEDLPTQLRCPKLATLTLKDNPHLRELPSEFVRSLTSLSVLNLSGCTQMASVPTSI

EKLRMLEFLSLPHLPIQDLPKKVCRLSNLQFLDLSNCESLKSLPSEIGEIKNLKSLNLYG

CGFLKTIPREISELTSLSRLVLETKLSMETEAPASIWSLKGLKNMTELRVRVDWRQQLKD

GIMGSWSEMRHLMLDYHGDEDLPEDMKSMKKLQSFMLHYYDGSSLPNCKFEHLEVVSLLG

CTSISDLSPLEWMPNLKLLKLEFCQNLRELGIGSRGSGSPSGYQRLQKLVLKDLPRLESL

GGASKRGVWDERTVPHLRVLKIRYCPSLKRFPVGMEKLPNLCALVGSSDWWERIRWEDEN

MKIKMQKIMKQYYP

>LL_027078_T.1

MLLPMKDILSSQSLVLVTTRDKHLLKQWGVLESSIYNMTGLNMPHSKELFCSYAFFKRFP

PPEFAELVDQFVTTCGGLPLSLKVIGALLCGENDEHYWKEQFDRLQQSLPEEIQKTLRIS

YDSLVKEDQEVFLDIACFFLGGDRDRAIRIWGLVGIRNLENKCLLEVDSQSKIRMHDHIR

DCAKIIAEEGSMPRRLNNIDDLLEQSPSVITEVRGIATVSCWSALYQAFFDYARQKENNC

HVHIIRNLQLVATGGGYLEGILRRVRSPHLIWLRWVRCSEFSLPDWIPMENLRVLEVQSR

RKLNFLWPHQSQGPLQLRELNIEAPLSEFPKSIGQLKYLESISIIDFDSTSENTLETLPQ

EFCQLCSLKYLVLSGFSRLMSLPFSFGKLTNLQYINLNGASRLQMLPNSFGDLSRLKHLS

LVLCSSLTISNGTLGNITTLEYLNLFGCGNVKELPPQVAHQRSLETLFLVDTNLKELPSG

IGELCNLEFLHIGGKSLEVLHPSVGHLKSLKYFSVIRSPKLKCLPDSFELLTQLTDLALE

RCGIEFLPEGLLKLNNLNGLHVHFCQELRQMPGVEHCMSLRYLRARECPKLQWDGGVLQQ

LRRRLGESLIIDVKQYNSSSRLQEL

>LL_027080_T.1

MAASSSSAVHYPYDVFISHRGPDTKKTLASHIYRGLSNNGLRTFLDQPELQQGDSLTSQI

EDAVRTASIHVAVFSPRYAESSWCLNELLLMLESGKTIIPLFYHVAPAELRRTRGRYGEA

LQKLAEKRTYEGECRYDSNTIEKWRDALSEVGCISGFELHQCNGDEGELVDKVVRFVVKK

MKKPGLCSTCGHLNVSITLMAASSSSAVHYPYDVFINRGRDTEKTLASHLYHGLSNNGLR

TFRDQPELRLGELTSQREDVVRTASIHVAVFSPLYAESSWCLNELLLMLESGKTIIPVFY

HVAPAELRWTRGKAGRYGEALQELAEKTTFDFESGKVFYQSVTIENWGKALSRVADISGF

ELDGDEGELVDQVVRYVLKKMKKPGLCSTCGHLNV

>LL_027092_T.1

MSLNGLQIKLVKDLKGIQINIEAGEDGVGVIKKNLSGCHTLIILDDVDHRDQLNAFLSIR

DVLDQRSLIVVTSRDKHVLRSAGIVESSIYLLTGLEMHYSKELFCSYAFFQRHPRPGFED

LVNRFLRACCGLPLSLKVFGALVCEHDPSYWEDQLLGLHRTQTLPTDIEKSLKISYDSLN

HEEQQMFLDIACFSIGKDRDTWITIWGASRWKGLAGFRNLEDKCLVVVDSEKKILMHDHL

RDLGRNIANFPGSMPRRLCGMRRDVLDLLEQSSAVMTEVRGIRMDYDWEKRMGRGIRMAH

DRDERMVPFLSEMPLNGSSSQHEVFEIRKLRLLEVEDEDDFVKNILSCVRAPQLLWLCWK

FCSFSSLPSWIPMHDLRGLEVDGINLRRLWQCESQAPLQLRELNLGHALSLLEFPKSIGQ

LEHLQKIVVQDAHSLASLPEEFCYLRSLKYLQLNGCTNLQTLPNSFGNLTSLQHIGLDSF

KKCKMLPDSFGNLTNLQHLYLGFCESLEMLPNTFGNLTNLLHLDLRFCRSLKMLPNTFGN

LTNLQHLNLYFCKSLRMTLDSCQNLTNLPFQCAIDIPTITLNGCTSLKRLEFLINVRRIN

IYNCPSLKMIPYSFGNLSNLEEITIKGCTSLKLLPDSFCNLTNLQRINLEGCTSLEMLPN

SIGRVRYLTVCNSPKLKCLPDSVGLLVHLKELRIWDCGIQYLPQDLIRMKDFRSLDVNNC

PLRGRPFEKVGEEIRGTLQLRELMLKRTEISKLFIPDSVCPDLQYLSVDCCNNLVEITAL

PTALRMLDLRKCGALTKIGGLSNCTKLLSLDMRGCNNLQVEELPDVLASMATLGA

>LL_027095_T.1

MEVVTGFGANVASNVFSGLWNNCFSGLWNRGVQVVKTAICFRQAVTDMEVQAQSLDDVVH

DIKSELAQEQKPAKRTVQAWLNRADNAIECSQSIRNRYQQPKNCLICCPNCHSRCTISKD

IVDWKLTVVQLHSERHSDNFPSSGEYGDHAPAAQIPIEIPKTGFVGNAIQSGQLKLEQLL

TEDTNIRIIAVHGMGGVGKTSLLQTINNSLQVRNSFDLIIWVIVSKDCEISNLQDIIARR

LNLGNFPYGS

>LL_027155_T.1

MKEVFRRDAHEMPDKYEVGLEEQAKDFQNEILTESHSNATTIVGIAGMSGIGKSTLDRYL

YRLRRSDFTRSCILCDVRNTDLLSLQKKLLWELLGNYSLPVFSDRYEGKRILQAFLRRLP

RIFIVLDDVDRMDQLDSLLDLDAVPSGSLIFITSHDKVLLNQFSANTRLYDVKVLPNGHA

RELFCRYAFRQCKPPEELKDLVDECLKICGGLPLSLRVLGGLFVGQYDRDYWEQELAKFR

LPQGIIDPLKVSYEALNPEEKEVFLDIGCFLAGEKKDLAVQVLEGLHGRDYLRSLHQKCL

VYFHFDVQSDDEIDCSQWYNQDQMIRYNDSCWSLKIRMHPQLRELARQIVRDEFYVLPTR

RPLRLSSLQDMLYMMRSPVARSNLEIRGIRVPGFVKPKYSIRDIREYLTRDIRVEGVCLF

VVEGLSMFPYAFRWWTVLGELVWLRLYNVSSFHFLRFINLRRLRVLEIRGDGDSLEELFE

ELFGRLDSEGTPSLNELNIEESSSPWNLNKLNIDTQASASTSGGKNLEIQKQVDETGTSI

QAEQRGSSLFRYLDSSVTKESLTQLKLKNLAFLPSLPIEFSRLQNLRHLDLSGCSSLTEL

PNSFSQLLQLRYLALQDCKNLSIPDDLLGEISTLEYVNFKGCAKLVALPHGIPYQRHLRY

LNLLYTGLLQLPENLELLHNLEHLTIGSQVLTELPLSISNLRGLKKLFLIQCSSLLRISP

EGIAAPNMQVLAIDGKVGMSSLRDFTLKNTQISKICIPEIFYPILETIDLSLNSGLMEVN

SLPSTLASLSLQGCTALKTLRSLSNLVNLKFLNVNCCCALKILIVEGLASLKELEASECW

ELGKIEGLRKRNRLNNLYMSTENRDTWNDICEYLRSPFQKPSIAIFSGPANDDAKVVMKY

LQGYRVCKEMQLKFNLQMHNIRKSSRSIKLENVCSYGAILMFFVANDYHEEMSTTSIDIT

FELFNGSKLCYKSLTGWARARGSVPKCEPTLILPPRRYRQSFHVFMWTEGSPLFKEKNTA

QVNFSFGDALVSTGWIAMVDKDTNVSKVCKELFKLPD

>LL_027225_T.1

MADPGLLSGFTGLCMNTAAQGILNHINIAVRCKKELNSLGVLVKRIDPIIAQIQQYRLAL

NQKKGIPNKAPAVDEWLQKLDALLKKGSAVAQECTIPRFNLITRYQLSRRITRLISDIKQ

HIDSAATLMAWSSILDEFGEIKESVQALASSQASTSAATSQTIIASTGFFIKEPLIVGQE

NDLARLEELVIDEAAKSPFLRIGMVGKGGSGKSLLLRTFFNSKKVRDLFSDGLLLLWLTV

SQSPSFTSLRYELSTQIAAQTKADFKENMNQEAA

>LL_027226_T.1

MLIKDLTGSNVQISSADEGPEKLRRYLSSSQALIILDDVDHIKQLDALLLPAKAVLHTSS

LILVASRNKDVLISSDIPESSIYKLTGLDPQHSRELFC

>LL_027228_T.1

MTEAAVVGAGALTEKFIAMVAQKLFEEVSLFTSFREEFEFFCDELISIKFLLNYTAERSN

STLMSNWLDSLEDFLVDAEDIVEECGTPPKFCNLIIKCRMARKIKILKERISKINRSAKY

VKYLSSVLHVNALNAYNSEDVREKSSAFIRESHTLGMERDIDIITKWILEEDGYGVIAIL

RMGGLGKTLLLQHVFKSEKVREHFDHFIWLSVSRKFVAKQLLPEIGRQIKLSPIMVKLPP

TPTPKSNESRAEEVKDKKDQNISKQVSFSKHISFRSPQISFRCRQSENQNLNEVSEEDLR

YRNAAELRENLHKYLQGKRSIFALDDVWDTDVFEKIGLPFATQNKIVFTSRDKRVADATR

AHHTHHKTCLSEENSWKLFCIHAFPDLAKPPHELTNVARLVVNKCGGLPLAVKIIAASMA

RLGRLRNHWESTLHSLNQTNFMGDRVMQSLTLSYHALPFHLKHCFVYCSAFPRNSEIKSE

VLVYLWIAQGLVSTQDVAHDVYDVGCSYLKELINRCLWKIHEVVIAKCMI

>LL_027276_T.1

MNSSSSGAPILPIFYGVKPADLRWTDQNKTGVYDKALQDLETKTTFNPQTQQMEPRYGSA

TLRKWRTVLRTVSDLHGFDFEEGPFNHDEGQMLNKVVQRVGEILAERTRLYVAKHPTGLD

DKVKDLETTVLRGKAQVVGIVGPGGMGKTTLAKEFFNRKRSQYNKSYFLSDVRENATSLH

CLQSKLLYGLTQLEQHIDDIHQGTEKVKNVLSSFQVLLILDDIDHIDQVDALLPEMDSLC

RDSLILITSRNIDVLTRAGVEESFIYKLKGLTPQHSQKLFSLYAFRKP

>LL_027327_T.1

MLENHLSSHQILLILDDVDGLDQVDALVPNQTSIHPNSLILITSRDKDVLTRSGVEHSSI

YKLNRLNEQHSLELFCWHAFRRSDPLTEFENLTAKFVNACNGLPLSLKVFGALLYGNTDI

SYWQQQLDRLVKILPTKIQESLRISYEALDCEEKQMFLDIACFFLGEDRDMAITIWNGSG

WAGELGFRTLQNKCLVEVEWNRRTGCNQIRMHDHLRDLGRAEARVSQPLRFWHIDDLLQQ

SSVSSQSFDIFV

>LL_027340_T.1

MGDPGFVSGFIGVSIGYAIHQIFHHVSIALRCKKELRSLRDLLMRIEPVIRDIQQYRLAL

NRKRGTPISKSDINMKPSAVNEWLKRMDSVLRQASTMAEECTVPSCDAISRYKTSRRIIR

RTSDISLHLESVTLMGWASVLEGLGQIKDSVESLAASSSATTSAITTSEPVTSTGIIINE

PLIVGQANDLGRLEKLATDVEVNPPFSRIGVVGKGGSGKTLILKTLFNSQKVRDLFSDGL

FLWLTVSQTPSIPSLQNDLKTQIAMQTLKLDPNRNMNIPLNESLQGKRFIIFLDDVWDHG

AELLEELGVLRVIHLFDSKII

>LL_027341_T.1

MGGIGKTTLAQEFFNRNSANYWRSYFLSDVRENAGKSSLHSLQRKLLKGLTQIDRQIESV

GEGKEILKGYFPSSHTLVILDDVDHVNQLDALLRPVQTVLGSDSLILITSRDKDVLTCSG

VKESSVYKLTGLNTHHARELFCLHAFSQPDPLPGFEYLVDNFLKVCDGLPLSLKVFGARL

FGKNNKSYWQDQLDRLQQIDVPAEIQHRLKISFDSLNKEEQEMFLDIACFFIGENRDMVI

RIWDGSGWKGLLGFQNLQNKCLVEVGTENDIHMHGHLRDLGKDIADASRSRRRLWHRA

>LL_027369_T.1

MISSSDSSTHKHRQFKEFQAVLEDGLKLVQEVDNLHIFDIYRKWRYGKKILEFQDKIKDL

IGIQGSPILAHDLQKLGADVTDLRRRFEAATIAEQSISHTSHEVPDIPNIVVGLDNRINA

VKQILLQNDVDRLGITGMGGSGKTTLASALCNNTQVKASSEKIIFIPVNKLIQM

>LL_027377_T.1

MASTSSTAADSNHDVFISHRGPDVKKNFASHLYHRLRFYGITAFLDKDELQAGEKLTSQI

EGAIRSASVHVAIFSSGYADSEWCLNELLEMLESKSPIIPVFYKDVEPADLRWTLDKKRT

YAQALEKLEKKTSPDPITQELKLRYASPTIEKWRDALYRVSMLSGFELAAFYGDEGELLN

KVVDGVLKMVGKPALDVADYPTGLDDKVKDFENSVLLQHQSEKPHPFGIVGLGGVGKTTL

ATELFNRKKSDFSRSCILFNVRENAGNNTLNLLQTELCKCLTGLDKPIVSVPEGKGILRK

HISSCKVFLILDDVDHIDHIYAFLPHQAVLHPNSLVLITSRNKKVLTSAGVEESRPYIT

>LL_027441_T.1

MANVRNNVFGEVVQKMQEIIIELERNLGILNTDFTSMKDLLNHISNQFQSRQRRAPEPVE

KCLGRMKDALAEAKNLIDGVNQQRERGWCLPSRNLFPGSKIRAWKARFNALLQELEMVFS

ISSSPEQIVSSSAPRLQRIHLLPVPNFVGSGIQSHLKELQTWLAGQARVIGVYGAAGVGK

TSLLKLIYNTYKYTKEVSGFFDIVIWFTVSRNFEIEQLQASIAEALNLSLEGTVETRKMK

LSASLERKRCLLILDNVSSKIDLNEVGIRTGDDNGSKILISSRSIEETEQMGERDFSVKL

QPWSSEESWELFKRGAFTNGVVPENIEDIAREMASACKGSPLVISVVAAAMSFKKTQKDW

RDASTSMGRTDPASSSRTHGNIDAELEQLLRWSYHDLSDTSLKICFLTCAAFLEDQEATV

ETLLEMWYAEGLLNPGAETPYLMDAGRQFIKKTGR

>LL_027448_T.1

MSISLSNTMPPNFSALLYFPPSQFLLLNCRSFFPSSPLLIAISIFQMAMAGSSSTAIHYS

YDVFISHRGPDVKKTFASHLYRALSNHGFRVFLDQPELQQGDSLTSQIRNAVTTASIHVA

VFSSRYAESSWCLDELLLMLESEKTIIPVFYQVTPAELRWTVCKAGKDGQPLQKLAKPLT

PVIAGKYAEALQKLAKKKTYDPQSCTWKPRHESATIEKWRNALSHVAGISGFELEACNGD

EGELLDKVVRCVCKKVNKIPLHVAKYPTGLDEKVNDFENKVLLEQQHSGKPPVLGIVGLG

GAGKTTLVKELFNRKSSQYIKSCFISDVRENAVKGSLISLQRKLLKSLTGMQVDDIDESI

ETLLKQHLASCEIFIILDDVDHVDQVDALLPDQALIRSDSLILITSRYKNILTTSGIEDS

SIYKLIGLNEQHSRELFCSHAFNQPYPLS

>LL_027482_T.1

MAESSSSSTSTYDVFINNCGPDVQKTFACYLYRLFLSYGLKVFLDPEDLKRGDYNNSQME

GVIRTASFHVAIFSSEYAGSTWCLKDLLLMKYDPQTPQETPRHDSATVGQWRNALSIVAG

ESGFELEDCNR

>LL_027511_T.1

MDIIISSKSSSKELRQKLLGLKPTIDEISRLSSDSDPSSYRVQLLKDFQAQLQDGLHLVK

KLERMSSFNLYRRYRYGKQILKVEKNVNDFLLTQGLAGLVLDVHKLNVDSKGCDKRLERI

EEMGRHIIDSANAKMTKDASSNSLVLHQMSTTQLFETSIDESYDTLMAEKSTSSCKSLVP

DMPNFVVGLNNLV

>LL_027559_T.1

MASASTSTSSSVFPRNYHVFINHRGPDVKKTFASHLYHCLRSHGLRVFLDRPELESGENT

PPQIERAIDTSSVHVAIFSPGYADSKWCLDELVLMLKSLKSGAVIIPVFYKVKPSHLRFA

DKGYTGAMRNHEKRYDQQKITNWKRALHDVSLITGFELESYNGDESELVLNVARQVLKKV

RKEPLPVATHPTGLDVKLQDFEVILERQQHREEPNVVGIVGPGGVGKTTLAKYFFNSKTS

DYQSCYLPGVRDAAARHSLTSLQSKLFRDLTGQDQSIDNVDDGIAKLKDRLSCHKALVIL

DDVDDVVQLDALFSPVKDALTSRDKDVPSGSVVLLTSRDKKVLRKYARIAESSIYNMTGL

GLEHSLELFCSHAFLQPHPAVGYEDLVEKFLDACQGLPLSLQANGALLYSENREFWEAQL

LTLASNGKQCWNLDFIVPVKLFFF

>LL_027589_T.1

MASASTSTAVNSCYEIFLNHRGPDTKKTFTNHLYHRLVLQGFRVFWDKQEMQVGDTLSSQ

IHGAIKAASLHVAIFSPRYAESHWCLDELLLMVESGKPIIPVFHHVHPTELRRTQGEGIY

AQALRKLENKTTYDSQPRYNSTAIENWRTALSTVAEISGLELDEFNGYEGDLIEEVVKVV

DSVVKKMGKPRLDVAEHPTGLDEKIEDFEKKVLFVQREHTGKPQVIGIVGSGGMGKTTLV

KELFNRKSSQYQKSCILFDVRENARKASLESLQKKLLKGLNEIDKNIESVSEGIQLLKTH

LSSCQALVILDDVDHTDQLDALLRPIRDVLHPHSLILITCRDKGFLGTSRVEESSIYKLT

GLNRQQSQELFCLHAFTQGHPLPGFEDLVRRYLEACDGLPLSLKVFGALLYKNDISFWQD

QLDRLEEILPKDIQKSLKISYDALQSDERGIFLDIACFFIGKNRDTAIRIWDGSGWKGSL

GFQSLHNKNLVEVDIENNIRMHDHLRDLGRDLAKNPELPRRIWRTEDYEDLLQQSSETDV

RGISIRWTKRAHPSFPRCRIRNLQLLDFNARNIHPLESIRNAMQSPNLIWFSWYNYDGYG

DSDHSSPPSWIPLKKLRVLELRGRKLRTLWEEESEAPLQLRELIVGCYAITSLPHWFGNL

TNLEHIELSMSDLESLPDSFGNLTRLQHLELSGCKRLESITLRNLTRLKYLHLAECTILT

SITLGTICTLEHLHLSFCTSIEVLPSLGRLNQLTKLTIDRCSRLREVSFKMVEGERESIY

LNLQHLELRGCNKLKSITLGNLTRLKYLELSGCDELKSITLGTICMLEHVRLSLCESIEV

LPAQLARQQSLEVLHLQNLWKLRELPSDIGRLNQLSRLTIHDCSLREVSFNIWTSRAVGI

WWRLEHCRMHSQH

>LL_027624_T.1

MDPSPASSSTASHSISSGYDVFINHRGPDTKNTFASYLYHALLSRGLRPFLDREELEEGL

EFPSQIVDVIQNASVHVAIFSPGYADSRWCLHELVLMQETTATIVPVFYNVEPAALRWTD

HNKTGRYAEALNKLQRKKTVNSQTVGSKKRKRSTPRYDPAIIQKWRDALSRVADTSGFDL

KGKFNGDEGLMLEKVIERVLKIMAKQTQLYVAKYPTGLDDKVKDFETTVLRQQPTGKAQV

VGIVGWGGV

>LL_027648_T.1

MANHQTSATGSSSTGASPNGYYNFDVFINHRGPDTKKTFASHVYRRLLLDRFRVFLDKQE

MQAGDDLTCQIKGAIAAASVHIAIFSPKYADSKWCLEELVLMLESGTTIIPVFYHVKPSD

LRIKTGKGEDGVYGRAFAI

>LL_027668_T.1

MASASTSNAVNTATKSCYQIFLNHRGPDTKNTFASHLYRRLAVLHGFRVFWDKDEMRVGD

TLTSQIHDAINTASVFVAIFSPGYAESKWCLDELHMMVESGKPIIPVFYHIQPGELRQTH

G

>LL_027712_T.1

MLVQRTQLDIQILEELERRRCLLVLDDIWNRNDFNQFNWLLSKAKIVLTTRDTGVLSTIS

PDLLKVYNLKKLCDENSKKLFRIHAFPDRGDTQQCPADTDQPPKDFTQHSTANTDIEPPK

ELVAFADQIVQKCGGLPLALKTIGASMARVRRLPNDWESILKSLNEAEAMSGKVWASLRI

PIRCRAFLH

>LL_027716_T.1

MALKLFQSASSPSSLGFAFASPLTTSSESVYYDVFINHHGVDVKKTFASFLYRSFRRMGL

RAFLDLEEFDMGDSLPDQIQHAILSSSVHIVIFSKNYAQSPRCLNELVFMLKTKARIHPV

YYHVQPAEVRFCIKGAYADAFSKYENQGRYSEKEIGDWKKALREASEIPGWDLDDFKGDE

GKLLEKIVDRVFRDVKTREVAKYPVGLYEAVEDFERKLGEFAHDRKKNAKLVAIVGMSGS

GKTTLAKKWFNLKHSEYDASCFLFHVRKTSARNELPILQRKLLKDLLHLDHKIDSTSHGK

EILRSCFATVGTASRFLIILDDVDHNNQ

>LL_027882_T.1

MPFAINIVAAMIGQTNVDQWDTCFSLMKTVDPSFQATHPLLDAQLYRLLRWSYDALPNSN

VKNCFLYCAFFMENQEVDSRELVQMWIGEGLVKSKQGTYLMDMALARKYLQLLIDRCFFQ

IVKDSTNVFRFTGYQAHDVIPSIIVHAAIREMAMYIGQKEENCLFIGSQNLRNFPEVHNN

NCRRMRISVADNFIEHLPTDMRCAELASLILKGNTNLKELPSDFLLSLTSLRVLNLSRCT

EITSLPTSMGQLKLLEFLSLSGLRNIKYLGEEICNLSRLQFLNLSQCGLLQSLPSKIAQL

TNLKFLDLDMCDSLMVIPHEMSQLTSLSRLVLPREMGLSMETEAPASIWSLKGLKNMTEL

RVSIHYLEQLKDGIMGSWSEMRHLMLSYRGDEDLPEDMKSMKKLQSFMLHYYDGSSLPNC

KLEHLEVVSLWDCHQISDLSPLEWMPNLKLLKLRSCFGLRELGIGSRGSGSPSGYQRLQK

LVLKYLYSLESLGGASKRGVWDERTLPHLRVLKIRNCASLKRFPVGMEKLPNLCALMGKD

SWWRSIMWEDEDMKTKLEKNFKSYYI

>LL_027888_T.1

MGATASNTAFTSTPSGIGNDDSQFHVYIHHRSPDVKKTLASHIYRRLRAHGLRVFLDTEE

LQVGDNLPAQIVGAIRTASLHIAIFSPRYAESSWCLDELVQMLESGSIIIPVYYHVKPSE

LRSTSGRTGVYAEALHRLEMKRSFDPQTQEEKPRYDSGTIEGWRSFGPQTQEEKPRYDSG

TIEGWRNALSHVADISGFDLHACNGDEGVLVDLVVLCVLKKLGIPLAAQELEGAASDRIS

LLTRDNANGRDIEMGLQQPPEGNNNTQQESGIVATPHYVQSIATFFATCHGAILAFVALV

MMTAPVSSAVRIFILFVSALVSVAIIRDLVLKSYNEFSGMRGGRSLRKSLVANQWWVIER

IALFLIFIMFSLGLLLVSNRVLKPQHH

>LL_027901_T.1

MFESGSPIIPVFYHVKPSQLRWTQGEGGVYSQDLRKLEEKKTYDTETHEEKPRHDSTTIQ

KWRNTLSSVAEISGFELDANTDEGVLLDKVVECVVKKKREARLNEEAQSYENTVLLEEQQ

PKGKLEVISMWSERYDVICGGIERILGPCIAVVFFIFWVIGFLNSMKKADEMEKGFPQGF

P

>LL_027911_T.1

MSGFICLSIDKAIKQILRDRNIADRYKDEWKPLKELVTSMEDTMRQIQHYRLLITWKIKD

SESAANDRNSESDDWVKDFNKRLDELNASLQDASQVAKDCAVSTHQVILRDQMSVRITEL

TSKIAENLQAMSSWVGDARAKGIVADARLITPAWQTVTPSSGVRDILAPSTTSATISQAA

LDNLPTGRMKLMDEPFIVGQEKILKRLQDLVTNESPTTKKIGVLGQGGSGKTLLLKRLFN

SKEVRNHFKTGFLLWLTVSKSPSFISLTNKLWTQIKMQDKKGAVKNINEQHVEIWIAIQN

NVDDIKNTKQEKVDDIKNTKQEKVDDIKNTKQANVKKWVNEALQQSSGFVLFLDDVWESD

>LL_028012_T.1

MTKSGATIIPVYYNVTRSDLQLTRENRDGEYAKALRRHQKKQRYDLNTLQKWRKALSEVA

ELPGLKLHGNPEKDLEIWVNDIETRVKSVLNSLGKRPEVRSSELRSSELQRVVEIVVNDP

NPCNAVQSGAISMEIVVSMENYYFGLAEQLEEFEKAVLSHKIEERGE

>LL_028170_T.1

MLKDLMESEKIPDIRKHEEGQRQLGCMLEKVQAFIYIDNVLNANELRELLPKDMNKAKKV

RLLLTARDENVGRVCPMETPTKIYHMKGISTREAASLLKKDIKEEIDSSQFDEIIEICGG

IPLKLTLVGPFISKAKNKQQAYRRLMQEKGRLKIEPFDEIERYVFAYDDLPDMCKDPFLD

ICLFFKGWDWDTVADIVGDIYLEMLESRALVTKDTNGVTSLHDVILTLGCQKTDRITRFK

FTSARKMKECLDHMKEKDIQNIKGIWLSDNKDLFLISATKLDSMYESLRVLNLGNFAKVE

GKCNEIFKKLIFFQGAVPDLPFDVTDLDLKYLSYQPTNLKVSEMPPSLRYMEFAGRLYAG

AFDISSRDLERLKHLRILRLTGFAKLKKLPDQLGDLVNGLQELSLSYCKSIEELPPSTSK

LQVLRVLRMDGCSSL

>LL_028175_T.1

MLDSDALVIPVYFDVTPDDCESIDSGPFAEAFRKHHEQGRIDRSILKQWIEALKKVTLRP

GKRLDDYNGRYGLLAMEVVAEVLKEVNKVTLEEAEHPVCLKQKVEELYTLIRSHKTSTRA

ATIVGIVGIAGIGKTTLAKALYNRVHSDYKAASFLSNMSETIRNKGLQALQTILLKDLLH

VDKNVRSPSEGKELLRERLNVVEDILIVLDDVGQHEQLADLLDLNALHPTNVILVTTRDR

GMLQRFRGCLEYEVKRLEATEAEKLFCFHVFGREEADEDLRDLVVKLVGMCHGVPRLLEQ

CGKRLFGKDKRSYWEFQLANLSEQLEYHAEDVQIADGNSEQEGFFTYEI

>LL_028182_T.1

MAHFESSSSTENTSSSAYDVFINYYDRNVEKTLASHVNRRLREHGLRVCFNRLELQGGDN

ITPEMEGAIRKASVHITVFSPGFVESSLCLNELMLRLESMKDRESGSTIIPVFYGVKPSE

LRWTRPGTDMGVYAKALLNFEEKKTFDSNTIENWRKAFYDVAEKSGFELDAFNGDKGKLV

NQIVKRVLKKLENHLYYDVFISYRAPDVKKTLASHLYHRLLAHGLRVFLDNQELQKGDGI

FFQIKRAIATSSVHIAIFSQNYAQSNWCLDELVLMLESGSRFIPVFHDVEPSKLLGQKDC

YGEALDMLHRKKIHDYKANEEKPRYDVDTIAKWRKALRDATNINGFTLDSCNGDNGQLVH

RVVQEVLSIYA

>LL_028189_T.1

MAGDIGINFAGNVLSNVFCNVWNKGVEKGKMVIYLPQAFNQMKEDVGTLDNEIHDINAEL

NRKQWPAKRRVQTWLDKAAELKLTSETIVTEYETYQQHKSCLACCPNCVRRYRISNDIRD

WTRKFSELFSEKAQPDFPRIGEYGDPTPRKQILIQDSDSGFVADAKRSTHTELLQSMLTD

EGSNIRRIGIIGMGGVGKTSLLDIIHNSPKVEASFEQFIKVIVSRNNISHLQKQVA

>LL_028245_T.1

MANLQISSVDTPSTSGHYTGCNFKVFINHRGPDLKIPFAIPLYHLLHSGGLQTFLDKEEL

EKGELLSPQIEGAIRTASIHITIFSPGYAASTWCLDELLLIWNKYKSCGDTIIPVFYDVM

PSELRWITGRYAEALSKHERKGRYDPKTLEKLRVALHDVSNIVGFELKAYNGDKEKLLDA

VVKCVLKKGRKIGLDVAMHPTGLEEKVEEFEKTVRLQERQSGKVKILGIVGMGGSGKTTL

AKYLFNKKSSNFGKSCFLFDVRENAARRSLNSLQTQLIKDLAHRDVQIYHVDQGIEMLRT

NLESSQHALVVIDDVDHADQLHAFLPIKDVLPSDSLILVTSRYKDVLTRSGIAESSIYKL

TGLNKQYSQQLFCLHAFCQPHPLSGFEDLVHKFCRACNGLPLSLKVFGGLLCEKSDKSYW

IGLLNDLILRAKLQNDILQTLKLSYDALGEEEKQIFLDIACYFIGEDMDRAIRIWDGSGW

SGLVGLQNLQAKCLVEVGRKNTLKMHDHLRDMGREIADQEKSSGLIPHRLWPLIEDIPDL

WQ

>LL_028254_T.1

MDQVPETVAKLAAYQIFINHRGPDVKQNLASLIYHRLTSMGLSVFLDKTEFQTGDTLSPA

ILGAIQSAYIHIVIFSEKYAESHWCLKELCWILESSKERDIKIIPLFCDVEPSDLRYIKK

GLYAKSFEQHEQKGRVTMDDIQSWKTALEEASHISGIPFTKNESDYGETLEKIVKIVMKE

VFRRDAHEMPDKYEVGLEEQAKDFQNEILTESHSNATTIV

>LL_028262_T.1

MVQPCLIPAKSLVNFSVRQSKKKICSLIKAIREHLKLSPLLVRIPEDQMKDTQKVLEGLG

KTKDSLEPLASSATTSQEAMKNLVVGQNQAFQSLQRLVIENVESLQRLVIENVEAKNIGV

LGKSGSGKTLHLRTLFNSQEVRDHFKGGLLLWLTVPQRLSITDLCQQIATQKGIDLKKEN

KEVDEKEWVSHQLEQSNRLALFLDDVCEGNGTELLDGLGIVQIVRKNPNSKVVVSSRDRT

ALSKMEVLDEYTVTIDETDMVELDNTFADLEKFVGAQEAHKIVVLGECGAAKSLLLKTIF

NRQKVRTYFSDDLVLWLSLSQRPSVTSVVNDLCQQIAEQTLLFLIPTMEEEAKKIWLKME

LQQRRFELFLDDVWERDAGILFEELGIWGAASSKVIVSSTDLTALSEKGVTENYTITIQE

LNQTKSWDLLKDHAKFPCDIGNLPLNIEIPLEKPVCAGLPQAIKVVGRAMAGSTHPQQWE

WALDRLQNAQSLDDCLRLSFDALGNDDVNMQLCFLHIAAAFPKDAIIEAQEVIPLWMGEG

LLARKKVEHVPRHVLADHCLIEPMIRNTEGRPACLRMQDPFQIGKLYLDLLAERCLIQPT

ARDADGLVLRFRMLSVVRDWAMPIAQDEENFYCCVDNGNGLKHLQNNNEPSKIKRLFLRG

NDLSSLQQFSSYKEMRSLLISENTGLTKIQKKVIGSMLSLKVLDLSGMSARSLPNVACLK

ELICLKLSGTPIKELPKSVTGLGHLEILDVSFTDIKELPSDIHKLRSLRYLGLRGCTHLQ

YLPSSISSLLSLQYLYMDGCSSMWTKPSGNRRKNVMDGCRKVAPLQSLGDLIQLKQLALQ

TNGEQQLQHLPDGKRQQLEQLLKSMTEMDTLLLVLPEMVSLPLHISDMSKLRSLTLKCSK

LIKMDSFICSSLTETPIPMKNDVTHLKHLSYLKFYECDELKEFQHLHKLPYLRQLEIILC

PKVENFTEFGVNEGFPSLKTFSLVGLPKLQVLPEIGGGAMPSLKIFTMMDCEILRMPKSY

LNLETKIRVYRCPKVEEEHMGMVHQVLPQPKSTKSIIEEYWQQHREKKKWLYGECWCNEL

FQSITDMRTLRG

>LL_028276_T.1

MLFKRIAFKDGRAPMEIQLCARNIVDACKGSRVVIRVVAAAMIGKTSLVDWQTSLHSINN

ADHPSFPHNQLLRWSYDALPTSDLKNCFLSSAIYPKDANINVTELVQMWIAEGLVRSKQE

THLMDMAIARTYVNLLLDRRFFHNATDEEIQDDEETQYIRVDGAIREMAMYIAEKDHNYL

LKAGQSLQHFPTVHSEDCKRISLRGNNIEDLPSEFRCPKLVSLILSYNPISVVPSGFLVG

LTSLKVLDLSYNQDISSIPSSVGQLQQLQLLRLDFFTYLKHLPEEIGHLSSLQFLSLSYC

SNLQSLPSQIGQLQNLKYLNISACWSLGVIPHEISQLTSLTTLDADGVKLSVEAESERSI

SRLKGLINLTMLKGLINLTSSGIEGGIMGTWLDMRHLELCFTGSDYAMDDLPDDMQNMRK

LQSLRMFNYSGLSMPAYSCKFQHLQHLKLESCEHLKELPPLERLPNLEELDLVGLNQLES

MAGASNSGVWNERTLPKLRILRI

>LL_028337_T.1

MLFQGIAFKDGPVPTDNIQLCARNIVDACKGSRSAISVVAAAMIGKTSLLDWQTSLDSMK

IADPSFRPENHPHIDPQLYQLLRWSYDALPTSDHKNCFLSSAIYEKDATIDVTKLVQMWI

TEGFVRSKQETHLMDMAIARTYLNLLLDRRFFHNPNPSSPYNEIQYSGWHCSDIQCITVD

GAIREMAMYIAENEQNCFLKAKNITSQRMQRMQPFPTIHDNEDCKRISLAGNEIEDLPSE

FRCPKLVSLILSWNTISVVPSGFLVGLTSLKVLDLSHNQDISSIPSSVGQLQQLQLLTLQ

GLLIKHLPEEIGHLSSLQFLSLKGCVYLESLPSQIGQLQNLKYLNICHCRRLRVIPHEIS

QLTALTTLDAWDVELSVEAESERSISRLKGLINLTMLRITVKVDVKEGNTSSGIEGGIMG

TWLDMRHLWLSFEHSRDDVMDDLPDDMQNMRKLQSLTMENYRGLSLPEYTCKFQHLQHLE

LNDCRHLKELSPLERLPNLKQLYLECPELKELGIGN

>LL_028340_T.1

MVVKEVLKKVKKPALHVAKHPTGLDDKVKDFENTVLLQLQQSGKPQMLGIVGLGGVGKTT

LAKEFFNRKSSDYSKTCFLADVRDNASKRDLILLQKKLLRSLNASDNIDGVDEGIGMLSK

HLSSSRALIILDDVDHLNQVDALLPHQSVHSDSLVVITSRDKDVLTRSKVENTLIYKLTG

LDTQQSQELFCSHAFNQLYPQPGFESLVLEFLKRCNGLPLSLKVFGALLYGKDISEWEDE

LESLRQILPGEIQQSLKISYNSLNKDEQQIFLDIACFFRGENMDSAIRIWDGSGWQGRRG

FETLRNKCLVEVDSKNKIQIHDHLRDMGREIAEEPELPCRLWRWTKKTIEPLLQQSLVGA

>LL_028345_T.1

MIFRGIFFLSTQSTCVSNRASSCQSTCVSNLHQRTQNPSPLTRHTHSFRPSLVSQSHSIM

ALTGIATNVAADIASTIVAAVVQQIKDVVDLEENIQLLINTDFKRMEVFLRYIDKQFQEQ

QRRVPEPVELCLTRMNDALTQAKLLVDRVQRQRRRCFGCCLLCNAKLTTQVTNWETRFAQ

LFQDLQNDFAITANAGQIVSTAAPQEGGLLQDVAPGDIVGSTFESAQTKLQTWLEPHCKA

HVVGLYGMGGVGKTSVLKAIHDNYKQKVSPIFDVIIWFTVSKYKTEEEDNKITALQFSIA

HTLHLDLKDCPTLEIRKLKLSVSLENKRFLLILDDLWSNMDLDKVGVKFGHDKGSKVLVS

SRSTDVIKAMG

>LL_028390_T.1

MPVHCLVLLFFSCQSILPTPMAQSTTAPASASKPFAVFINHRGPDVKNHFAQYLYRRLKS

RGLEVFLDQPELQAGKSIISQIREAVQVSSVQVAIFSENYAGSKWCLRELCLMVEAEKSR

AAAILPVFYKVKPSELRWTGTDDKGLYAQALRHHEEKQRYNSEIIQSWRDALSYVADISG

FELDACNGNEAELLDKVVERVLTVVPKPALHVAKYATGLREKIEDFEKTVLLQQELEPVE

ANVVGITGGGGVGKTTLASEFFNCKRSQYNFSSFLPDVREKASSSSLNSLQRKLLKDLTG

IDKEIGSWQEGIEILETNLSSCNALIILDDVDDADQLEAFLSIKRVIDRKSLILVTSRDK

QVLRSVGMAESSIYHLSGLSKEHSQELFCSHAFFQRHPPRGFENLVVRFVNGCDGLPLSL

KVFGALLCKKDQSYWEEKLKELHKLDRKILASLKISYDSLRPKEQKVFLDIACFAIGEDR

EKWVRIWGGSAALQTLEDRCLVELEDRIMSPTSHGFFGEDRLLITLPVRIRMHDHLRDMG

REIAKEVSIPRRLWRSSKYVYDIFEEQSTSVIPEVRGIKTPSTHDSEDLLTHSKLIVALR

KSSSHEAFRIRKLQLLDTEGEFVQGILTRVQSPNLRWLCWNCCPCSSLPSSLPMDHLKVL

EVN

>LL_028462_T.1

MAGVGKTTLLKLVYNFYKVSPVFDVVIWLTISQDYKISDLQGRIAKALNLNPDNDVDTLK

MMLSARLIKKRFLLIFDDMWGALDFQELGVAFGSENLGSKVVFSTRNRDLALMEAQQSIK

VEPLPRDEGWALFEKVAFRGGHVPEELKECAREIADECEGLPLAITVVAAAMSAKTSMDE

WNTSLSLMKIADPSFPSTHPRVDKELYRKLRWSFDDLPTYLQNCFLYCALFPEDHQIHAD

ALLQMWIAEGVVKSKEATHSMDMELGFSYVKLLIDSGLFQNGNFEKQSNDIIVNKPVNVW

KWRGITVHDVIRDMAIYIGEKEDNHVCRAGQGLTDFPHSPSRDCNKHTVSANISQSTQTP

GVSFIEKHSDHGIAGRDMQSFQAPIFVSWRLQQIIIIAMYDRKAHTTAVPFFVELS

>LL_028463_T.1

MWYPKSKINEWKKALAKVASFAGWSRDDTFGFEAGLVKLVVQDVFKTLQNVIPEEDDGPD

NPPDMAEEVPLEAIPLEIRNEPLRISKSVFGMEERMDEVINLLKTDSEDSLITVAICGTA

GMGKTSLAKAVHNHIYHKFEAACYVYDVRHKAQHTNGIAKMQRQILKDLVKFKDKVNDEA

HGKSLMRDRLRSIKALVILEYVGDSKQLEALRGDWYGSGSRVLVTTLDSQLLID

>LL_028475_T.1

MQEGENITSHIKEAIATTSIHVLIFSLGYAESDWCLNELLLVRDSVAPIIPVFYNVRPAD

LQWTEGKDGVYAHALHNLQKKKTHDSETHEEKPRYDSDTIESWRKALHSVAEISGFELEA

CSNGDEGELLDKVVLSLPKR

>LL_028568_T.1

MAYPTPSCSSFPQTQSEIEMENQTSRPATSVGASSSSTAADPSDSCSTILYDVFISHRGE

VKKTLASHLYRGLLSRGLRPFLDQQELQEGLHFPAQLKAAIRSAWVHVAIFSPNYAESQW

CLDELLLMQESGAPIIPIFYHVKPAELRWTDQSKSGAYAQALNQPRMKSRYGSATIQNWR

DALSRVANISGFDLDGPFNGDEGHMVEKIVERVMKMSEKPGLYVAKHPTGLNEKVTDFET

TILKQQQLSGNVQVVGIVGSGGAG

>LL_028623_T.1

MAQLTSAAQHKFTSIDHPLASASSSSSSTCSASKTSYAHYHAFINHRGPDVKNTFAHNLY

KTLKSCGVRVFLDKPEMIEGHPITSQIKSAIETASVHIAIFSESYAKSSWCLDELVHMVN

SGTTILPVFYKVEPSVVRWTGKDSNGEYAKALRNHEEKQRCDFQTIQNWRKALNRVADIK

GFELKAHDGEEEEKRLLYNVVLGVLRNVPKTPLDVTKHPTGLEKGIENFERAVLSQEEEE

GVEAKVVGIVGVGGVGKTTLAKEFFNRKRSEYGCSSFLFDVREKAATKSLNSLQMQLIVD

LKHRDDIKFERCEEGIGILKKHLSSCQPLIILDNVDDVDQLNFFLPIKHVLNPKSLILVT

TRDKHVLSSAEIAESSIYHLCGLDKSHSQELFCRHAFFWQHPLPGFENLVDEILAACDGL

PLSLKLFGALVRGHDKAYWEDVLKELRQTYTQSDEKQNIFESSYCSLSTEEQHMFLDIAC

FFIGKNRDTVIRIWEGSHWKGLAGLQNLEDKCLVEPADSENMIRMHDQLRDFGRNKATSL

GSMPRRLCCSIKDVHDLLEQSSSSVIQEVRGIRMPHRSAIEDSSDQELFPSKPEMLLNNS

SSHEKFQIGNLQLLEAEDSFVECILSMVEPPQSLIWLCWNDCPDSSLPLWIPMEHLRVLE

VAGSKLTRLWQHESQAPLQLRELNIKAPLLELPKPLGQLKHLEKIVVEYPVILQEKGLAT

LPDEICALCSLKYLELKEFHDLRILPNSFGNLTNLQHINLCSSFNLQFLPDSFGNLTMVK

CINLSYCSNLAMLPDSFGNLRSLQHINLYRCKNLKVLPDSFGNLRNLQGIDLGSCRRLHM

LPTSMENLTNLQRISLRDCRRLKMLPNSMGNCTNLEIINLHGCCDLEMLPDSFGDLENLK

>LL_028663_T.1

MESALRDFITLFEPEKKALREIIAAKFWLALLLNSLIEAKGLLDSPAPRQSFLDCLSKEI

REWSASFNNIYDYLSILVTSLQFLSSNPFHIEEKAEEPTVIQPLSGDEAWMLFLRVAFKD

DQVSVDIQDNARRFADECKGMPLAINIVAAMIGQTNVDQWDTCFSLMKTEDPSFQATHPL

LDAQLYRLLRWSYDALPNSNVKNCFLYCAFFMENQEVDSRELVQMWIGEGLVKSKQGTYV

MDMALGCKYLQLLLDRCFFQIVHESTRFFRKKYPWDRTKR

>LL_028760_T.1

MAGVGAGAVKFLAEKVGTALLEKLQDDVSVVVAFKEDFELIHRELIHIKFLLTDAGDNMK

SQSMTNWQQSLRDVLTQAMDLIEHCDELDNSNVSSHHWIQGKIIFLRQMVSRIYSKLSLG

REIQALKKSIDQINDTGSKYVTYLKSLGDVNESADQEEKVISCSRLLKNDQSVGIQNDID

VITELILQRDGPGVIVVVGTGGQGKTRVLQHVFNSKAVQNGFKHRVWLPISRKFNIDELL

QVVADQIKLQGDELAIKDRTYGHEELTIQLPNDYHNKLTQVSTEGLKARIQRYLGEIHNL

EHEMASPFVSLAAWISRYFGESKKPSLFVLDDVWEKNILQQIVSVVLHCQDNQNKIVVTT

RDRGVADAIGGHTQYRMEYLSDQDSLELFCIHAFSKDLPVRKLPEDVKFFERLILDKCTR

LPLAVEAIAKRMADASRISHQWETALHSLDKAAVIREDVLPTLRSSYNALPYHLKACFHS

FASFPPNIPIKS

>LL_028791_T.1

MAQYQTFASSSSASTPAICNANYNITSASTSTAANPNTNYNYDVFINHRGPDVKKTFASH

LYRRLLSYGFRVFLDQHELDEGENLTSQIEAAIATASVHVAIFSKRYAESKWCLDELLLM

LKSKATTIPVFYDVRPHELRWTTGESRGTYAEALGNLEKKTTYDSQTHKEKPRYDAKTIE

NWREALSSVAEISGLELEACNWDEGELVDKVVQLVQKKARKPPFDVATYPTGLDEKLDDF

EQTVLLQNDSGEAKVTGIVGLGGVGKTTLAKYFFNRRRSDYHRSSFLFDVREAADMSTLT

SLQSKLYKDLTGQDQLIYSTDEGIGMFRHRISPSEALIVLDDVDSLEQMRVLFSPVKDAL

NSSSFILVTSRNKDVLTQSGIKESSIYKLTGLKRDYSQELFCSHAFHRPYPVVGFEEVVG

QFLDACQGLPLSLKVIGALLFLENPNLDYWKAHLSKISKVLPIDIHRRLKISFDSLDEQE

KEIFLDIACFFIGEDMDKAIRIWDGSGWGGSISFRNLENKCLVEVDRKNCIRMHDHIRDL

GRALADKESRLRLWRRTDLDVFHQSSVRGISIVKGQTFKEPVEVSNRYVDINGLQLLRAH

GDCLGSISNLLTKSDLLWLRWNNFPNASLPSWIPLKNLRVLEVRMASGELKTLWKRKSEA

PVQLREMIIGPRLLKFPNSIGKLKHLQKLTVYYIEKLREEFFHLRSLKYLRMRTGVKLLP

KAFGNLTNLQHLNLTNCNCLQMLPNSFGNLTNLQYLDLSGCSNLQMLPKSFGNLLRLKHL

CLTDCSKLTLSNVTLGNISTLESLDISNCQKMDVLPLQV

>LL_028880_T.1

MAASASNTASTSTPSGIGNEHSRYSVFINHRGPDAKITLARPIYRRLLAHGLRVFLDPEE

IQAGEDRTSQIEGAIRTASLHIAIFSPRYAESSWCLNEIVLMLESGSIIIPVYYHVKPSD

LRWTSHRNGVYAEALHGLERKTSFDSQTWQEKLRYDSGTIEGWRKALFDVADRSGFDLDA

CNGDEGLLVDRVVLCVLEKLGIPLAAQESEGAASDRISLLTRDNANGRDIEMGLQQPPEG

NNNTQQESGIVATPH

>LL_028903_T.1

MAASSSTAASIFTAEPNISYPACDVFINCGVDVKKTFARSLYYRLRENELTAFPDIEELQ

PREEILPRTKAAIRSASLHVAIFSPRYAESQWCLNELILMLETEAPIIPVFYHVKPSELR

CAKAPIIPFFYHFIPSQLRWTQGKHGNYAQALQRLEQEPTDEGNLRYDSGIIEGWKNALY

TVACMSGLELEACYGDEGELLDKVEQRLLKLVEKEKYLTGLDEKLKDFEETVQHSTKPQP

IQEKFKIKYDWKDQLKILQPIQENLSYDGKDQLETLQSIQEKPNINVKPDMIFNPNMSYK

PNISVKPDMIDNPNMSYNPKISSGKKDGRENVSIQPIQEKPNISSYDWEDRWENLRIHYD

SLSVEEKQIFLDIACFFIGKNKDMAIRIWDGSGWKGRLGFENLQNKCLVEVNSWNMIQMP

YRLRDFGRVVAAEATSQRRLWRWTENVSDDLVQKLSVSSQSFSPILLNKN

>LL_028904_T.1

MAASSSTAASTSTSSDLNSNTLFDVFINHHGLDVKKTFASHLYQRLHSYGLKVFLDQPEL

QRGEDFTSQIEGAIGAASVHVAIFSVSYAESTWCLNELLWMLKSKA

>LL_028950_T.1

MAFESAVVSGFLANVASAAIVGGVKAVKDAFGWEYPKHQDLQIVPDSGFVGRGIQSARKK

LQIWLNEPEARVIGVSGMGGVGKTSLLQLIHNDCKEKVSTNFDFVIWNTVSGSQSSKIPL

PNEEFDRLKIESLQYTIAESVGLDIKGIPIDTRKMRLNACLKNKRFLLILDDVWRPIDLD

QVGVNFGHDNSSRVLISCRNKFVVQTMAANYQYCVMEIEPLSTEDGWDLFRRKAFRNGPA

PDNRVEAIARDIAEECQGLPLALNTVAAAMAPKTGAVEWRRALDFMRNVDPSLSSTHPTI

HDQLYQRLKWSYNDLPSGLKMCFLYCAAFPEDEWIPVETLVEMWTAESLVPQRRATYYMD

VGREYIKALVDRCLIQYDEGKDAIRVHDVLRDMAIYVGEGEEKGLYLSSQHLQHFPTEEE

TRDRKRISLLDNQISYLPTTDLRCQTLVSLVLAINEELKQIPDRFFENLISLKVLDLSYT

AIEALPASVGQLGQLQCLHLKGCYHLKKLPDRIGELKHLQFLNLSYCARLKELPDRIGEL

KHLKHLNLEGVELDRVNPHQICQLTSLNRLVLPPHLTPMSLEDLTNLSKLIVLEVRVKPE

IKGGSMSSWSEMRELTLYFRDLDDVDDVDLDDDVGLDDFYDVDLDDDDDVGLDDLNDVDL

DDDDDVDLDDVEYDIVEDDAALDILPQ

>LL_029003_T.1

MMLESNADIIPVFYHVKPTDLRWTLGKNGVYAEALQKLEEKTTCDAQTNQRKSRYDSATI

EQWRNALSMVSGISGFDLEACNSDEGVLLNKVVESVLRKVKKTPLNVAKYPTGLNEKITD

FENKVLLKQHSGRKLPHMVGIVGLGGVGKTTLAKELFNRKSTDYSRSCFLHDVREYASKG

SLTLLQSKLLRNLTESVEQIDNKDEGVEKLKEPLSSYKSLLILDDIDNVDQVDALLPDQT

IIHSNSLVLITSRNKDVLTTSGVEESSIYKLNELDTQHSLELFCSHAFGRPYPLPKFKYL

AEKFANACHGLPLSLKVFGALLHGKNDMSYWHDQLDRLKKILPHEIQERLNISYEALNEE

EKQIFLDIACFLIGQDKHMAMRIWDVSGWKGRLGFQNLQNKSMVEVEPGNRIQMHDHLRD

LGRYVAGSSSPCRLWCWTENVIEDLLQQSYNGQVITVRGIKMALSEDRHDDEFDGIQMRR

LQLVDTEGSLLESILKRVKSPNLIWLCWKKCPYSSLPYWIPMKNLRILQLSGSTLETLWQ

HDSQLPVCLEELRVDGCKQLKSLRGLAQLTKLQRLFVCECSELEELPSMEALLSLEEFVA

VGCVKLKSILGLAKLTKLRYLNVGCVKLKSILGLAKLTKLRHLNVSECSELEELSSMETL

LSLEVLEAQQCVKLKSIPGLAQLRKLRHLNVSNCPELEELPSMETLLSLEWLWADGCVKL

KSIP

>LL_029056_T.1

MLGRYLPSSCRALICIDDVDDLDQLSALFFPVKHTNSLILITSRNKDVLRSAKIEESSIY

MVKGLNTQDSKELFCWHAFKQSYPVKEFEQLVDNFLVACGGLPLSLKVIGALLYIGNKDL

NYWKEQSHKISKILPKDIKSTLEISYNGLDNEEKQIFLDIACFFIGNIQRDTAIRIWNGS

GWQGWLGFRNLENRCLVEVDSENRITM

>LL_029074_T.1

MQEGENITSHIKEAIATTSIHVPIFSLRYAESDWCLNELLLMRRPADLQWKEGKDGV

>LL_029127_T.1

MAGDANTALLRFIYNKYKKQVSGSFDSVIWFTVSQKYKIEDMQAFIVDKLNLDFNEISNI

DTRKMKLSASLEKKNILLILDEMWSPIDMDEGVKFGDHNGSRVLITSRCRDVYEIMAAND

Y

>LL_029143_T.1

MACLATVTGFAANVSSNVLSGLWNKVVEAVKMVISLPQAVTDMEAQVQRLKDVRDNINAD

LAREQSDKPKTIVKNWLENAGKAIDSSVSINNEYQEHRNCLACCPNCVCRYRISRRIRDL

ILRIDRLHLEKQSDFPQSGQYGDPTPLTQIPIRTPIFVGDGIQSAQSELERLLTEDTDIQ

VVGVYGMGGIGKTSLLQAINNSNKVCESFQEIIWVTVSKDHDILKMQNCIGERLKINNFK

EISNFEQRKYVLYSKLKDMKFLLILDDIWRVVELESLGVSLHDTTSKILLSTRSRVVCTQ

MKAGVIRSVEPLSEEEGWQLFCSRAFLNGNAPHEIEDVARKIAGECKGLPLAINVVAAAM

TEHGTDLHLWEVALRQMQNIGRTFDDLHPEVDEQLFQRLRWSYDALKSDHLKICFLYFAA

YPEDSVIYCGEVIDMWISESLVEGNGESYLLDTAHSYINHLRDRCLIEVVHTDQVGRISR

VKIHDVLRDLAIRIAEKEHRCYFKAGQGVTDFPSQEDSEGCARMSLMRNDLRSLPTTFAF

SSLSILLLRENRGIEEVPESFLRELSSLKVLDLSGTGITSLPPSIGNLKHLASPRAYVNC

RKAFAT

>LL_029162_T.1

MAASSSTAASTSTSSFDVFINHRGCDVKKNFASHLYQRLRSYGLNVFLDQEELQRGDCFS

SQIRGAIRAASVHVAIFSVRYAESTWCLNELLWMSESEAPIIPVFYHVEPAELRWTTLGK

DGVYAQALHKLEEKTTDDPQTQTQGEMSPHEATSIINWRDALSIQHWRNALKKKYDPQAH

QKTLRHIPATVAQWRKALHNVSLISGFELKECNGDDGILLTKVIESVLKNVKKPLDVAKY

PTGLAEKITDFENTVLLPHLQSGRKPQIVGIVGLD

>LL_029168_T.1

MGIVDTSTFTMGDLSEDMRRAELECDKCGGLLLAIKVIGRAMESITDSHEWELAILRLPN
[truncated: 1,339,168 more chars]
